# METTL14 promotes neuroblastoma formation by inhibiting YWHAH via an m6A-YTHDF1-dependent mechanism

**DOI:** 10.1038/s41420-024-01959-8

**Published:** 2024-04-22

**Authors:** Jianwei Wang, Hongli Yin, Gen Li, Di Wu, Yunyun Xu, Yanling Chen, Xiaodong Wang, Yujiao Xing, Ting Zhang, Danhong Fei, Pengcheng Yang, Fang Fang, Yanfang Tao, Xiaolu Li, Juanjuan Yu, Yang Yang, Zhiheng Li, Lei Shi, Zimu Zhang, Jian Pan

**Affiliations:** 1 Institute of Pediatric Research, Children’s Hospital of Soochow University, Suzhou, China; 2 Children’s Hospital of Soochow University, Suzhou, China; 3 School of Medical Technology and Information Engineering, Zhejiang Chinese Medical University, Hangzhou, China; 4 Department of Pediatrics, Municipal Hospital Affiliated to Taizhou University, Taizhou, China; 5 Department of Pediatric Surgery, The Affiliated Hospital of Yangzhou University, Yangzhou, China; 6 Department of Medicinal Chemistry, Jiangsu Key Laboratory of Drug Design and Optimization, China Pharmaceutical University, Nanjing, China

**Keywords:** Paediatric cancer, Cancer epigenetics

## Abstract

Neuroblastoma (NB) is a common childhood tumor with a high incidence worldwide. The regulatory role of RNA N6-methyladenosine (m6A) in gene expression has attracted significant attention, and the impact of methyltransferase-like 14 (METTL14) on tumor progression has been extensively studied in various types of cancer. However, the specific influence of METTL14 on NB remains unexplored. Using data from the Target database, our study revealed significant upregulation of METTL14 expression in high-risk NB patients, with strong correlation with poor prognosis. Furthermore, we identified ETS1 and YY1 as upstream regulators that control the expression of METTL14. In vitro experiments involving the knockdown of METTL14 in NB cells demonstrated significant inhibition of cell proliferation, migration, and invasion. In addition, suppressing METTL14 inhibited NB tumorigenesis in nude mouse models. Through MeRIP-seq and RNA-seq analyses, we further discovered that YWHAH is a downstream target gene of METTL14. Mechanistically, we observed that methylated YWHAH transcripts, particularly those in the 5′ UTR, were specifically recognized by the m6A “reader” protein YTHDF1, leading to the degradation of YWHAH mRNA. Moreover, the downregulation of YWHAH expression activated the PI3K/AKT signaling pathway, promoting NB cell activity. Overall, our study provides valuable insights into the oncogenic effects of METTL14 in NB cells, highlighting its role in inhibiting YWHAH expression through an m6A-YTHDF1-dependent mechanism. These findings also suggest the potential utility of a biomarker panel for prognostic prediction in NB patients.

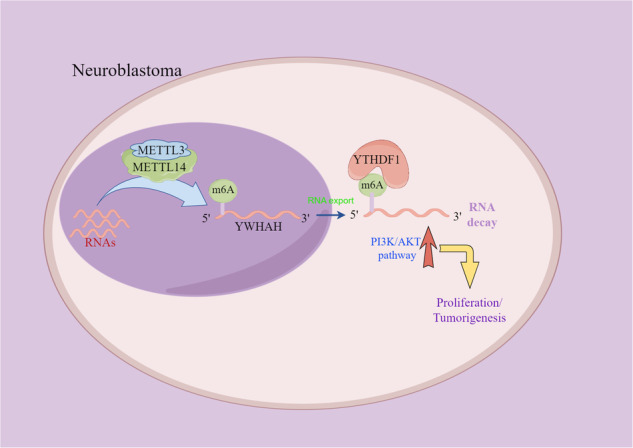

## Introduction

Neuroblastoma (NB) is a pediatric tumor that typically originates from the neural crest in the adrenal gland region [1]. It is the most common solid extracranial neoplasm in children, primarily affecting infants and young children [2]. Although significant progress has been made in NB treatment over the years, which has led improved survival rates, effective treatment options for refractory and recurrent patients are still limited [3, 4]. Therefore, a comprehensive understanding of genetic alterations that contribute to NB risk is crucial for revealing the underlying causes of this disease. N6-methyladenosine (m6A) is a prevalent posttranscriptional modification of eukaryotic mRNA. It plays a critical role in mRNA metabolism and translation within cells, as well as in cellular differentiation, embryonic development, and tumorigenesis [5–9]. M6A methylation is catalyzed by the m6A methyltransferase (writer) complex, which includes methyltransferase 3 (METTL3), methyltransferase 14 (METTL14), and WTAP [10]. This modification is reversible and can be removed by m6A demethylases (erasers), such as FTO and ALKBH5 [11]. In addition, m6A can be recognized by various reader proteins, including YTH domain family proteins, HNRNP family proteins, Igf2bp family proteins, and eIF3, triggering downstream biological reactions [12]. For instance, YTHDF1 functions as a promoter of translation, YTHDF2 facilitates the degradation of mRNA, and YTHDF3 functions as a cofactor in target mRNAs, collaborating with these proteins [13, 14]. Understanding the roles and interactions of these m6A-associated proteins provides valuable insights into the regulatory mechanisms involved in gene expression and cellular processes.

A mounting body of evidence suggests that m6A modifications and their regulators play crucial roles in the pathological mechanisms of various cancers [15]. Numerous studies have consistently demonstrated the involvement of m6A writers, particularly METTL3 and METTL14, in promoting the survival, proliferation, and invasion of tumor cells [16]. The overexpression of METTL3 in hepatocellular carcinoma (HCC) leads to the degradation of the oncogene SOCS2 through m6A modification, which is associated with poor patient prognosis [17]. Abnormalities in the METTL14 gene have been implicated in various aspects of gastrointestinal tumorigenesis, progression, drug resistance, and metastasis [18]. Furthermore, m6A modifications have been identified in pediatric tumors such as gliomas [19], NBs [20], retinoblastomas [21], and acute lymphoblastic leukemia [22]. In glioma development, the involvement of HUR in METTL3-mediated m6A modification of the lncRNA MALAT1 has been observed, leading to increased MALAT1 transcript expression and the promotion of glioma development through the ERK/MARK pathway [23]. A correlation between single nucleotide polymorphisms (SNPs) in the METTL14 gene and NB has also been reported [24]. An epidemiological case study evaluating the relationship between SNPs in the METTL14 gene and the overall risk of NB was conducted on a cohort consisting of 898 patients and 1734 controls. Moreover, a study conducted in 2020 identified METTL14, WTAP, and YTHDF1 as components of a risk prediction model, suggesting their potential contribution to the treatment and prognosis of patient with NB [25]. However, our understanding of the involvement of m6A modification in pediatric cancers is still limited, and the specific mechanisms and targets of its impact remain unidentified, and require further investigation. These findings indicate a possible association between the METTL14 gene and NB susceptibility, although the underlying mechanisms remain unclear.

YWHAH, a member of the 14-3-3 protein family, acts as a negative regulator of the protein kinase PDK1 and mediates signaling through phosphoserine-binding proteins [26]. Namkoong H et al. reported that YWHAH may play an oncogenic role in various tumors, including cervical cancer, lung cancer, ovarian cancer, and breast cancer [27]. However, YWHAH has also been implicated in hepatocellular carcinoma, where its deletion activates the PI3K/AKT pathway, accelerating epithelial-mesenchymal transition (EMT) and cell cycle progression [28]. The aberrant activation of the PI3K/AKT signaling pathway, a critical survival pathway, has been observed in several human cancers, including NB [29]. However, the specific contribution of YWHAH to NB remains unknown.

The results of our study demonstrated that the upregulation of METTL14 expression had a negative prognostic impact on patients with NB. Furthermore, we found that METTL14 expression was regulated by the transcription factors YY1 and ETS1. Importantly, dysregulated METTL14 expression significantly influenced various cellular processes in NB, such as proliferation, colony formation, migration, invasion, and tumorigenicity. Through RNA-seq and MeRIP-seq analysis, we identified YWHAH as a target gene of METTL14. Mechanistically, METTL14 downregulated YWHAH expression in an m6A-dependent manner, facilitating the recognition and subsequent degradation of YWHAH mRNA by YTHDF1. In addition, activation of the METTL14/YWHAH axis stimulated the PI3K pathway. Furthermore, activation of the METTL14/YWHAH axis promoted the progression of NB by activating the PI3K/AKT pathway. In summary, our investigation successfully elucidated the significance of the ETS1/METTL14/YWHAH axis in NB, revealing a potential therapeutic target for NB.

## Results

### METTL14 overexpression is observed in NB patients and is correlated with poor prognosis

**Fig. 1:**
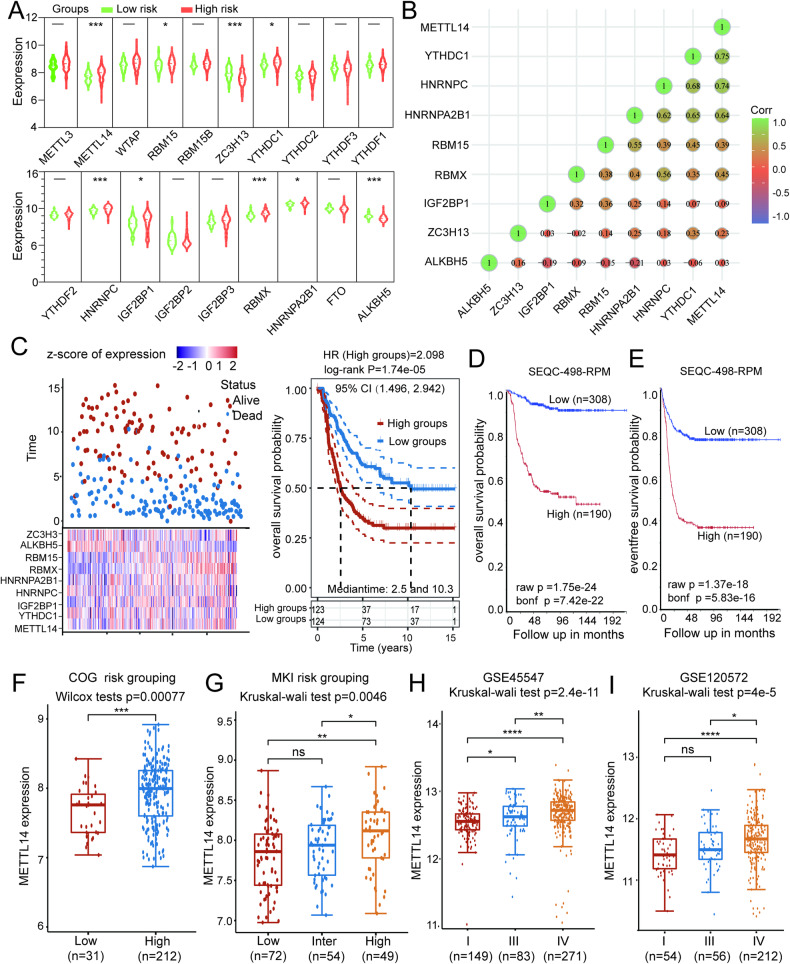
Expression, correlation, and prognostic information of m6A methylation-related genes in Neuroblastoma. **A** Expression profiles of m6A methylation-related genes in a high-risk group and a low-risk group of neuroblastoma. **B** Correlation matrix of interaction in m6A methylation-related genes with differential expression. Correlation coefficients are plotted with negative correlation (blue) and positive correlation (green). **C** Prognostic analysis of nine-gene signature in the training set. Left: Survival status of the patients; Right: Kaplan–Meier survival analysis of the ten-gene signature. **D**, **E** Kaplan–Meier overall and event-free survival probability analysis of METTL14 in NB. **F**, **G** Expression of METTL14 in neuroblastoma MKI and COG groups. **H**, **I** Expression of METTL14 in two neuroblastoma cohorts (GSE45547 and GSE120572) at different times, y-Axis represents the normalized log2 expression value. Data are represented as mean ± SD. *P < 0.05, **P < 0.01, ***P < 0.001, and ****P < 0.0001.

Numerous studies have investigated the correlation between m6A-related genes and tumor progression [7]. By utilizing the TARGET database, we created expression violin plot of 19 m6A methylation-related genes in the low-risk (31 cases) and high-risk (213 cases) groups of neuroblastoma patients. Notably, nine genes (METTL14, YTHDC1, IGF2BP1, HNRNPC, HNRNPA2B1, RBMX, and RBM15 were upregulated, and ALKBH5 and ZC3H13 were downregulated) exhibited significant variations in their expression levels (Fig. 1A and Supplementary Table 1). Pearson correlation analysis revealed a significant positive or negative correlation among some of these nine genes (Fig. 1B and Supplementary Table 2). Specifically, METTL14 and YTHDC1 exhibited the most substantial positive correlation, with a correlation coefficient of 0.75, while ZC3H13 and ALKBH5 displayed the most significant negative correlation, with a correlation coefficient of −0.02. Subsequently, we constructed a prognostic model for NB using these 9 genes (Fig. 1C and Supplementary Table 3). Based on this model, patients were divided into high-risk and low-risk groups, with the latter consistently showing a superior prognosis. The Kaplan–Meier (KM) method was used to generate survival curves.

Among the nine genes that were significantly differentially expressed, METTL14 was identified as the most crucial m6A methyltransferase. To investigate the correlation between METTL14 expression and the prognosis of NB patients, we conducted a comprehensive analysis using the R2 database (https://hgserver1.amc.nl/cgi-bin/r2/main.cgi?open_page=login). Our findings revealed a noteworthy correlation between METTL14 expression and overall survival (OS) (Fig. 1D) and event-free survival (EFS) (Fig. 1E) in patients. Notably, individuals with high levels of METTL14 expression displayed considerably lower OS and EFS rates than did those with low levels of METTL14 expression. Moreover, METTL14 expression significantly increased in high-risk patients in both the MKI group and the COG group (Fig. 1F, G, Supplementary Tables 4 and 5). Furthermore, our analysis of two separate datasets, namely, GSE45547 and GSE120572, demonstrated a substantial increase in METTL14 expression (Fig. 1H, I, Supplementary Tables 6 and 7).

These analyses provide evidence suggesting that genes associated with m6A modification play crucial regulatory roles in NB. Given the prominent role of METTL14 as a methyltransferase, its increased expression is expected to modulate gene expression through m6A modification, thereby influencing the onset and progression of NB, which is the central focus of our investigation.

### METTL14 regulates the activity of NB cells

To evaluate the expression of METTL14 in NB cells, we utilized the Cancer Cell Line Encyclopedia (CCLE) database to compare METTL14 levels in various tumor cell lines. Through the compilation of CCLE data, which can be accessed at https://sites.broadinstitute.org/ccle/, information on more than 20 tumor types was gathered. The findings indicated that METTL14 mRNA expression was high across all tumors (Supplementary Fig. 1A and Supplementary Table 8).

**Fig. 2:**
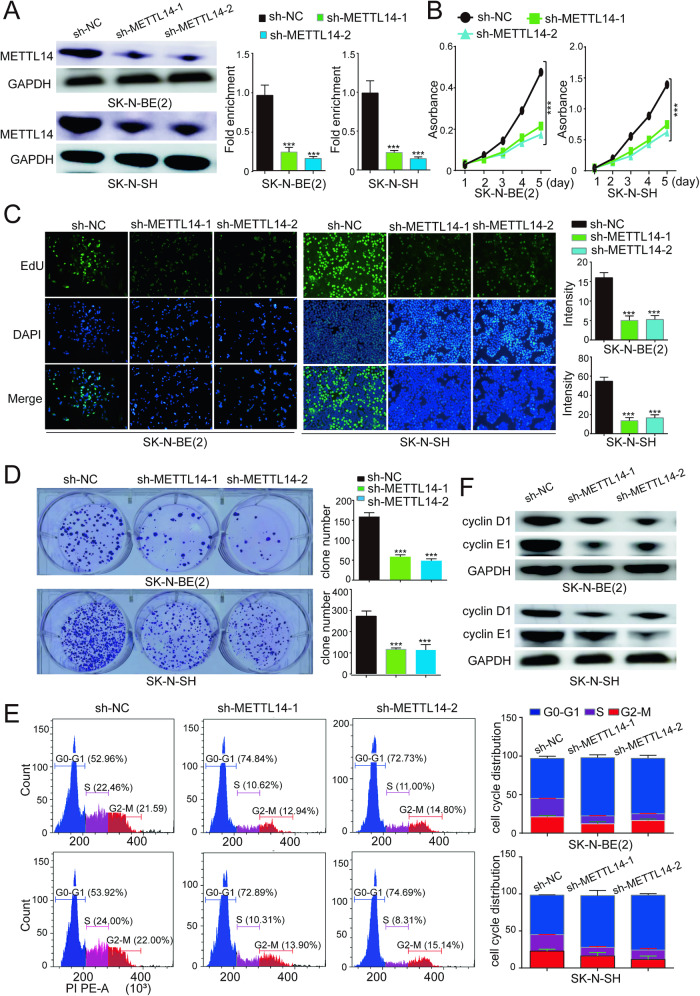
METTL14 knockdown inhibited the survival and proliferation of NB cells in vitro. **A** The METTL14 expression in SK-N-BE(2) and SK-N-SH cells is analyzed by western blotting. The internal parameter is GAPDH. **B** Cell proliferation of NB cells transfected with sh-NC or sh-METTL14s was detected by CCK-8. **C** EdU staining of SK-N-BE(2) and SK-N-SH cells. Images showing DAPI-stained nuclei (blue) and EdU-stained proliferating nuclei (green). **D** The clonogenesis ability of NB cells was weakened after transfection with shRNA. **E** The cell cycle distribution was calculated by flow cytometry using PI staining. *P < 0.05; **P < 0.01; ***P < 0.001; n.s: not significant.

To investigate the impact of METTL14 on NB, we suppressed its expression in two NB cell lines, SK-N-BE(2) and SK-N-SH, by constructing shRNA vectors. Transfection of the two vectors led to a significant reduction in the protein level of METTL14 (Fig. 2A). The CCK8 assay revealed a noticeable inhibition of cell proliferation after METTL14 knockdown (Fig. 2B). In addition, compared with control cells, METTL14 knockdown cells displayed significantly lower EdU fluorescence intensity (Fig. 2C). Furthermore, we observed a decrease in the ability of NB cells to form colonies following METTL14 knockdown (Fig. 2D). Collectively, these results strongly indicate a substantial decrease in cell viability upon METTL14 knockdown. Conversely, the overexpression of METTL14 (Supplementary Fig. 1B) enhanced cell proliferation (Supplementary Fig. 1C) and colony formation (Supplementary Fig. 1D). Notable changes were observed in the distribution of the cell cycle, particularly a significant increase in the duration of the G0-G1 phase, along with a marked reduction in the expression of cyclin D1 and E1 (Fig. 2E, F). These preliminary findings suggest that METTL14 inhibition hinders cell cycle progression.

**Fig. 3:**
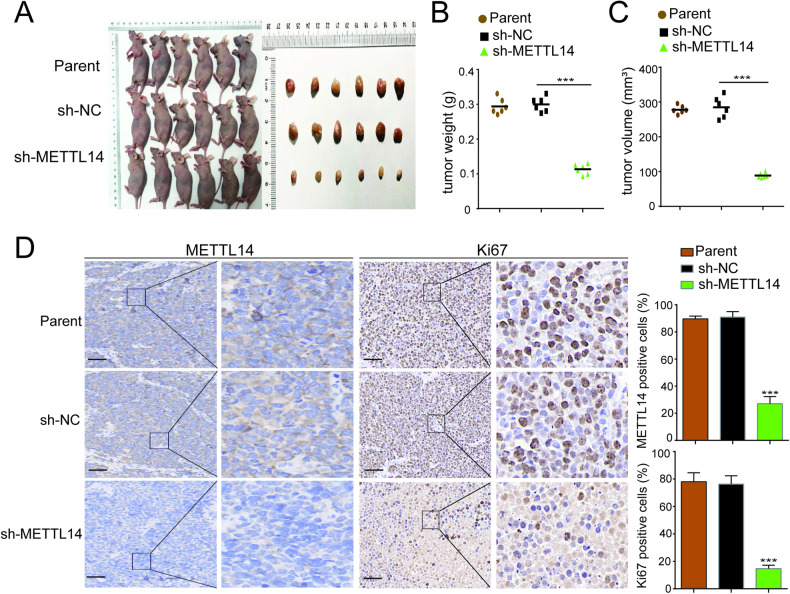
METTL14 knockdown inhibits tumor formation in vivo. **A** Photos of nude mice and xenograft tumors formed by subcutaneous injections of sh-NC or sh-METTL14-transfected SK-N-BE(2) cells. **B**, **C** Tumor volume and weight at the end of xenografts (*n* = 6). **D** IHC detection for METTL14, Ki-67, expression was conducted in sections of tumors harvested from xenografts. All data are shown as mean ± SD (scale bars of D = 50 μm). NS, not significant; *P < 0.05; **P < 0.01. The data are based on three separate experiments.

To further explore the connection between METTL14 and tumor development in NB, we utilized a transwell assay to evaluate changes in the migratory and invasive capabilities of SK-N-BE(2) and SK-N-SH cells after METTL14 knockdown or overexpression. The results indicated a substantial increase in the migration and invasion ability of both cell lines following METTL14 overexpression (Supplementary Fig. 1D). Conversely, cell migration and invasion were notably inhibited after METTL14 knockdown (Supplementary Fig. 2A). Subsequently, SK-N-BE(2) cells with METTL14 knockdown were subcutaneously injected into nude mice, resulting in a marked decrease in the weight and size of the tumors (Fig. 3A–C). Immunohistochemical analysis of tumor sections revealed a significant reduction in METTL14 expression (Fig. 3D) and a corresponding marked decrease in Ki67 expression in METTL14-knockdown tumor tissues compared to those in the control group (Fig. 3D). These findings provide compelling evidence of the impact of METTL14 on tumor growth and proliferation in NB.

### ETS1 and YY1 regulate METTL14 expression in NB cells

**Fig. 4:**
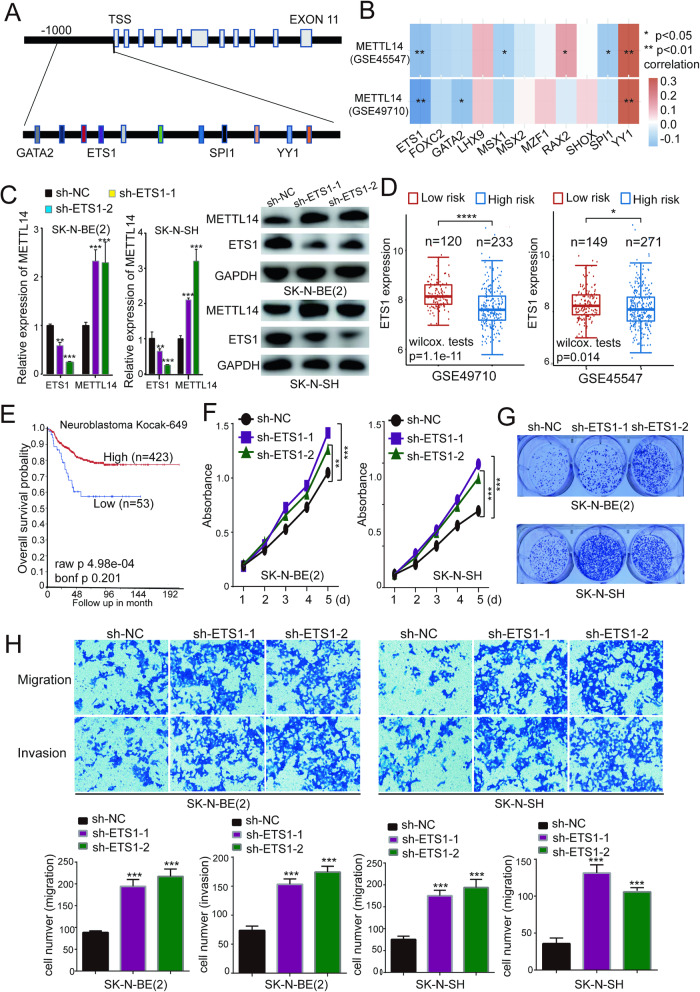
Identification of ETS1 as a potential transcription regulator for METTL14. **A** AnimalTFDB3 and JASPAR were used to predict transcription factors that might bind to the METTL14 promoter sequence, and 11 more likely transcription factors such as ETS1, SPI1, GATA2, and YY1 were found. **B** Two cohorts, GSE49710 and GSE45547, were used to analyze the correlation between METTL14 and 11 transcription factors. **C** ETS1 was knockdown in NB cells and its impact on the METTL14 RNA and protein levels were evaluated by qPCR and western blot. **D** ETS1 expression was remarkably lower in patients with high risk than in patients with low risk of neuroblastoma. Two datasets GSE49710 and GSE45547 were used for this analysis. **E** Kaplan–Meier overall survival probability analysis of ETS1 in NB. **F** Cell proliferation was detected by CCK-8 assays in NB cells transfected with sh-NC or sh-ETS1s. **G** The capacity of colony formation was decreased by shRNA transfection. **H** The migration and invasion of SK-N-BE(2) and SK-N-SH cells transfected with sh-NC or sh-ETS1s. All experiments were in three replicates. *P < 0.05; **P < 0.01; ***P < 0.001; n.s: not significant.

The expression of METTL14 in NB is regulated by transcription factors. To identify the specific transcription factors that control METTL14 expression, we initially conducted bioinformatics analyses using the JASPAR and AnimalTFDB3.0 databases. These analyses were performed to predict potential binding sites for transcription factors within the region spanning from −1000 to 0 nt of the hMETTL14 promoter. By integrating these predicted results, we identified 11 transcription factors with a high likelihood of involvement. These factors included YY1, SPlI, GATA2, and ETS1 (Fig. 4A). Furthermore, we performed a correlation analysis between these transcription factors and METTL14 in NB patients. This analysis revealed that YY1 and ETS1 exhibited strong correlations with METTL14 in NB patients (Fig. 4B and Supplementary Table 9). Specifically, YY1 expression was positively correlated with METTL14 expression, while ETS1 expression was negatively correlated with METTL14 expression. Through data collection from the cistromeDB database, we revealed that YY1 can bind to the METTL14 promoter region in NB cells (SK-N-SH). In addition, YY1 binding to the METTL14 promoter was also found in HepG2, K562, and A549 cells (Supplementary Fig. 3). Although information regarding ETS1 binding to the METTL14 promoter in NB cells was not unavailable, ETS1 binding to the METTL14 promoter was found in Jurkat, K562, and A549 cells. To verify whether YY1 and ETS1 can bind to the METTL14 promoter region in NB cells, we performed ChIP-qPCR experiments and found that YY1 and ETS1 were indeed able to bind to the METTL14 promoter sequence (Supplementary Fig. 4).

To confirm the role of YY1 and ETS1 in regulating METTL14 expression, we utilized shRNA constructs to suppress their expression. Our findings demonstrated a significant increase in METTL14 expression following ETS1 knockdown (Fig. 4C), while YY1 knockdown inhibited METTL14 expression (Supplementary Fig. 5 and 5B). Therefore, our results suggest that ETS1 and YY1 are two transcriptional regulatory factors involved in the regulation of METTL14 expression. In addition, a previous study showed that YY1 can promote the proliferation of NB cells [30], and in our study, its expression was significantly greater in high-risk NB patients (Supplementary Fig. 5C and Supplementary Table 10). We also found a significant positive correlation between YY1 and METTL14 in NB (Supplementary Fig. 5D and Supplementary Table 11), and patients with high YY1 expression have a poorer prognosis (Supplementary Fig. 5E). ETS1, on the other hand, is known to regulate the expression of several genes and is often considered an oncogene [31]. However, it has also been reported to have apoptosis-inducing activity [32]. In other types of cancer, such as hepatocellular carcinoma, ETS1 has been found to act as a tumor suppressor [33] and inhibit tumor cell proliferation by targeting RYBP [34]. A direct correlation between ETS1 and NB has not been previously reported. Therefore, we aimed to further analyze the effect of ETS1 expression on the activity of NB cells.

Furthermore, we examined two cohorts, GSE49710 and GSE45547, and found a statistically significant reduction in ETS1 expression in the high-risk group compared to the low-risk group (Fig. 4D and Supplementary Table 12). A prognostic correlation analysis of NB demonstrated a positive association between high ETS1 expression and favorable overall survival (OS) (Fig. 4E). Subsequently, we investigated the impact of ETS1 knockdown on the activity of NB cells, and the CCK8 assay results indicated a significant increase in the proliferation of SK-N-BE(2) and SK-N-SH cells following ETS1 knockdown (Fig. 4F). Moreover, the colony formation assay results showed a marked increase in the clonogenic capacity of NB cells after ETS1 knockdown (Fig. 4G). In addition, the transwell assay results indicated a significant increase in the migratory and invasive capacities of NB cells following ETS1 knockdown (Fig. 4H).

Based on these preliminary findings, it can be concluded that ETS1 acts as an upstream negative regulator of METTL14, thus influencing the activity of NB cells through the regulation of METTL14 expression.

### YWHAH is a downstream target of METTL14

**Fig. 5:**
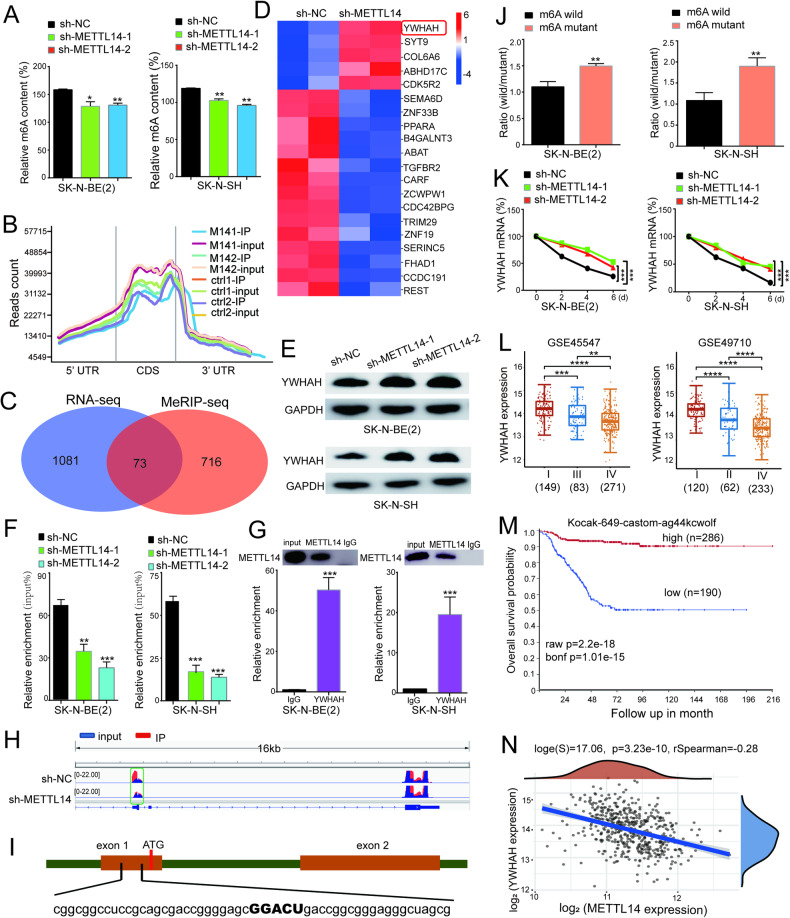
YWHAH is a downstream target gene of METTL14. **A** The total m6A level of NB cells decreased significantly after METTL14 knockdown. **B** MeRIP sequencing results showed that m6A modification sites in NB cells were mainly concentrated in the CDS region and the stop codon region. **C** There were 73 gene overlaps between RNA-seq and MeRIP-seq. **D** The heatmap shows 20 genes with significant differences in expression. **E** The protein level of YWHAH upregulated after METTL14 knockdown, determined by Western blotting assay. **F** The m6A modification level of YWHAH at the 5 ‘end decreased significantly after METTL14 was knocked down, detected by MeRIP-qPCR. **G** Rip-qPCR results showed that METTL14 protein could bind to YWHAH transcript. In this experiment, METTL14 antibody was used for RIP, and IgG antibody was used as negative control. **H** The change of m6A modification level on YWHAH gene was mainly in the 5 ‘non-coding region by IGV visualization analysis. **I** The 5 ‘non-coding region of YWHAH has a highly probable m6A modification site. **J** The activity of luciferase increased significantly after m6A mutation. **K** The degradation rate of YWHAH transcripts decreased significantly after METTL knockdown. **L** The YWHAH expression was remarkably increased in neuroblastoma patients in stage III and IV. Two cohorts GSE45547 and GSE49710 were used for analysis. **M** There was a significant correlation between YWHAH and the OS probability of NB patients. **N** The YWHAH and METTL14 expression showed a significant negative correlation in NB. *P < 0.05; **P < 0.01; ***P < 0.001; n.s: not significant.

The impact of METTL14, a prominent m6A methyltransferase, on the overall level of m6A methylation in cellular systems is profound. Our investigation of m6A levels revealed a significant reduction in m6A methylation in SK-N-BE(2) and SK-N-SH cells following METTL14 knockdown (Fig. 5A). To gain a deeper understanding of the underlying mechanism by which METTL14 contributes to tumor development and to identify its downstream targets in NB, we conducted RNA sequencing (RNA-seq) and methylated RNA immunoprecipitation sequencing (MeRIP-seq) analyses using sh-NC and shMETTL14 SK-N-BE(2) cells. RNA-seq analysis revealed that 739 genes were downregulated and 415 genes were upregulated in the METTL14 knockdown group compared to the control group (Supplementary Table 13). Consistent with previous studies, the MeRIP-seq findings indicated that m6A modification sites were predominantly localized in the stop codon region (Fig. 5B and Supplementary Table 14). By comparing the two sequencing outcomes, we identified a total of 73 genes, that overlapped between genes showing significant disparities in RNA-seq results and those demonstrating substantial reductions in m6A modification (Fig. 5C and Supplementary Table 15). A heatmap was constructed to visually represent the expression patterns of the top 20 genes showing noteworthy differences in expression (Fig. 5D and Supplementary Table 16). Among the identified genes, YWHAH, an adapter protein involved in mediating multiple signaling pathways, consistently exhibited reduced m6A levels. In addition, YWHAH mRNA was greater in METTL14-knockdown NB cells than in control cells. Therefore, YWHAH was selected as a potential target gene for further investigation. To validate this finding, we assessed the expression of YWHAH at the protein and RNA levels after METTL14 knockdown. The data demonstrated a clear increase in YWHAH expression upon METTL14 knockdown at both the RNA (Supplementary Fig. 6A) and protein levels (Fig. 5E and Supplementary Fig. 6B). These findings suggest a potential regulatory role of METTL14 in modulating the expression of YWHAH through m6A methylation, shedding light on its involvement in the tumorigenesis of NB.

To investigate the potential m6A modification of YWHAH transcripts, we used m6A RNA immunoprecipitation (RIP) qPCR. The findings revealed significant enrichment of YWHAH transcripts through m6A antibodies, with a notable reduction in m6A modification levels after METTL14 knockdown (Fig. 5F). To confirm that YWHAH is a target of METTL14-mediated m6A modification, as determined by MeRIP-seq, we conducted RIP-qPCR in NB cells using a METTL14 antibody to assess the binding of METTL14 to YWHAH mRNA. The results demonstrated the binding of METTL14 to the 5′UTR sequence of YWHAH mRNA (Fig. 5G). These data indicate that METTL14 can regulate the expression of the YWHAH gene through m6A modification.

Visual analysis using IGV software revealed that m6A modification of the transcription product of the YWHAH gene was primarily concentrated in the 3′ UTR (Fig. 5H). However, m6A modification level did not significantly change after METTL14 knockdown. Conversely, a notable decrease in the m6A modification level in the 5′ UTR was observed after METTL14 knockdown. Further analysis of the 5′ UTR sequences using RMVar identified a prominent m6A modification site (Fig. 5I). To examine the impact of the m6A modification site at the 5′ UTR on YWHAH expression, we introduced a 1000-bp promoter sequence and 5′ UTR sequence of the YWHAH gene into the pGL3.0 vector. Subsequently, we assessed dual-luciferase activity and observed a significant increase in luciferase activity upon mutation of GGACU to GGCCU in the 5′ UTR sequence (Fig. 5J). These findings indicate that METTL14 modulates the expression of YWHAH by modifying m6A sites in the 5′ UTR, highlighting the importance of m6A modification in the 5′ UTR. To investigate the potential regulation of YWHAH mRNA stability by m6A modification, cells were treated with mitomycin C. The results suggested that METTL14 knockdown significantly impeded the degradation of YWHAH at the RNA level (Fig. 5K). Therefore, it can be inferred that m6A modification in the 5′ UTR possibly accelerates the degradation of YWHAH transcripts. Furthermore, the expression of YWHAH trended lower in high-risk NB patients than in low-risk NB patients (Fig. 5L and Supplementary Table 17).

Prognostic correlation analysis revealed significantly greater overall survival in NB patients with high YWHAH expression than in those with low YWHAH expression (Fig. 5M). The data analysis also indicated a clear negative correlation between METTL14 and YWHAH expression in NB patients (Fig. 5N and Supplementary Table 18).

### YWHAH act as a potential tumor suppressor by regulating the PI3K/AKT pathway

**Fig. 6:**
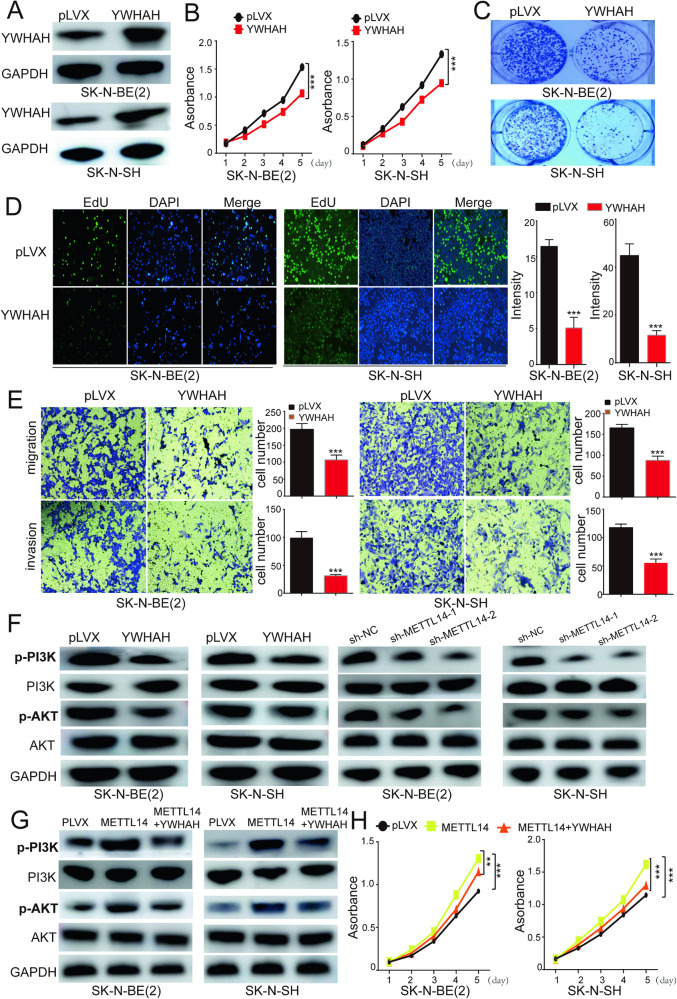
YWHAH overexpression inhibits the activity of neuroblastoma cells by regulating the PI3K/AKT signaling pathway. **A** The overexpression of YWHAH in NB cells was detected by WB. After YWHAH is overexpressed, the cells were harvested for colony formation assay (**B**), CCK-8 colorimetric assay (**C**), EdU staining test (**D**), migration and invasion (**E**). **F** YWHAH overexpression or METTL14 knockdown significantly reduced PI3K and AKT phosphorylation levels. **G** Overexpression of METTL14 increased the phosphorylation level of PI3K/AKT, while overexpression of YWHAH significantly decreased the phosphorylation level. **H** CCK8 detection showed that METTL14 overexpression could increase the NB cells proliferation, while YWHAH overexpression could significantly decrease the increased level. ns, not significant; *P < 0.05, **P < 0.01. The data are based on three separate experiments.

To investigate the potential role of YWHAH in the progression of NB, we overexpressed YWHAH in NB cells (Fig. 6A). Subsequently, we conducted cell proliferation (Fig. 6B) and colony formation assays (Fig. 6C), the results of which demonstrated a decrease in NB cell growth following YWHAH overexpression. This trend was further supported by the results obtained from the EdU test, which indicated that the overexpression of YWHAH significantly inhibited cell proliferation, as evidenced by decreased fluorescence intensity and density (Fig. 6D). Overall, these findings suggest that YWHAH may act as a potential tumor suppressor, exerting inhibitory effects on NB. Transwell assays revealed that YWHAH overexpression significantly inhibited cell migration and invasion (Fig. 6E).

Previous studies have reported that YWHAH can inhibit the PI3K/AKT signaling pathway [28]. Therefore, we investigated the impact of YWHAH overexpression on the PI3K/AKT pathway in NB cells. At the protein level, we observed a significant decrease in the phosphorylation levels of PI3K and AKT following YWHAH overexpression, indicating the inhibition of PI3K/AKT pathway activity (Fig. 6F), similar observations were made after METTL14 knockdown. However, METTL14 overexpression led to a notable increase in the phosphorylation levels of PI3K and AKT, while YWHAH overexpression resulted in a reduction in the phosphorylation levels of PI3K and AKT (Fig. 6G). Notably, when both METTL14 and YWHAH were overexpressed, the proliferation capacity of cells was significantly lower than that of cells overexpressing METTL14 alone (Fig. 6H).

To further validate that YWHAH can be regulated by m6A modification, we chose a small molecule inhibitor of m6A methylation [35], SAH, and then assessed cells viability. Treatment of the cells with SAH significantly inhibited the proliferation of both SK-N-BE(2) and SK-N-SH cells (Supplementary Fig. 7A). Detection at the protein level revealed a significant increase in the expression level of YWHAH, which further suggested that YWHAH can be regulated by m6A modification. Moreover, the phosphorylation of PI3K and AKT was significantly inhibited (Supplementary Fig. 7B).

These findings suggest that YWHAH serves as a regulatory gene targeted by METTL14 in NB.

### YTHDF1 recognizes the m6A site of YWHAH

**Fig. 7:**
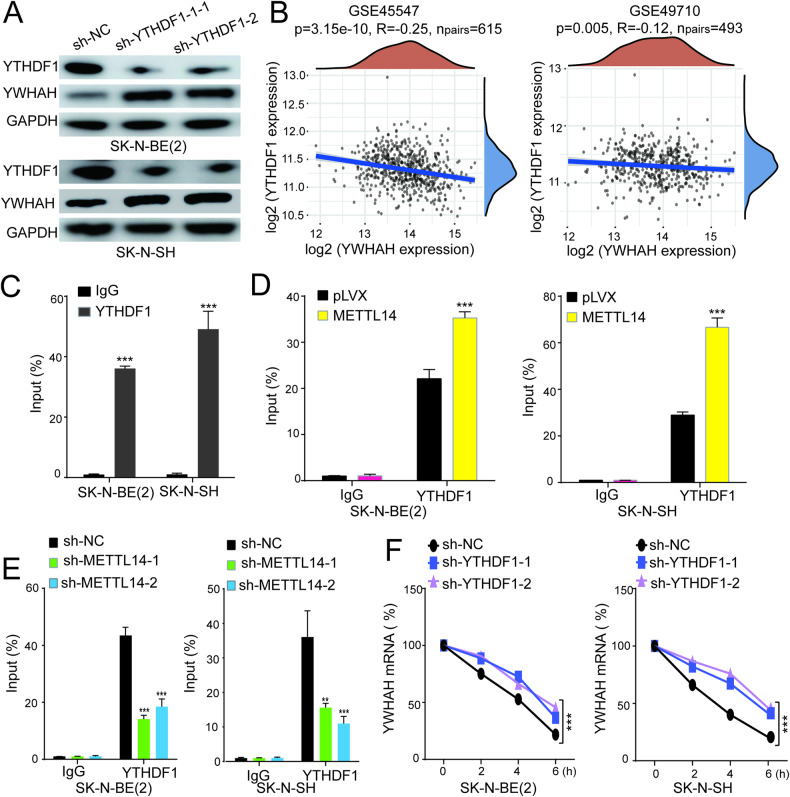
YTHDF1 facilitates YWHAH mRNA decay in an m6A-dependent manner. **A** The level of YWHAH protein was increased after YTHDF1 knocked down. **B** The YWHAH and YTHDF1 expression showed a significant negative correlation in NB. **C** Results of RIP assays of SK-N-BE(2) and SK-N-SH cells followed by qPCR demonstrated direct binding of YTHDF1 and YWHAH mRNA in NB cells after inhibiting (**D**) or overexpressing (**E**) METTL14. **F** Knockdown of YTHDF1 remarkably decreased the degradation rate of YWHAH mRNA. All experiments were in three replicates. *P < 0.05; **P < 0.01; ***P < 0.001; n.s: not significant.

The recognition of m6A methylation indeed plays a crucial role in modulating gene expression, and identifying the specific reader proteins that bind and regulate the m6A modification site on YWHAH mRNA is of highly important. Among these reader proteins, YTHDF1/2/3 are recognized as primary proteins for m6A recognition and have the ability to regulate mRNA stability and function. In our investigation, we utilized an shRNA vector to knock down the expression of the three YTHDF proteins to investigate their binding to YWHAH mRNA. The findings demonstrated a noticeable increase in YWHAH protein levels following YTHDF1 knockdown (Fig. 7A). However, the knockdown of YTHDF2 and YTHDF3 did not affect the expression of YWHAH (Supplementary Fig. 8). Through thorough data analysis, it was discovered that the expression of YTHDF1 in NB was significantly negatively correlated with YWHAH expression (Fig. 7B and Supplementary Table 19). To confirm the potential binding of YTHDF1 to YWHAH mRNA, a RIP-qPCR experiment was conducted, which yielded results indicating that the YWHAH transcript can indeed bind to YTHDF1 (Fig. 7C). Importantly, the knockdown of METTL14 significantly diminished the binding between YWHAH mRNA and YTHDF1 (Fig. 7D), while the overexpression of METTL14 led to an apparent increase in binding (Fig. 7E). These results suggest a direct regulatory relationship among METTL14-mediated m6A methylation, YTHDF1 binding, and YWHAH mRNA expression, shedding light on the intricate mechanisms underlying the regulation of gene expression in NB.

To further investigate the regulatory role of YTHDF1 in the stability of YWHAH mRNA, we analyzed RNA degradation after treating cells with actinomycin D. Our findings demonstrated a significant decrease in the degradation rate of YWHAH mRNA following YTHDF1 knockdown (Fig. 7F). These results provide evidence that YTHDF1, which act as a recognition protein for the m6A modification site on YWHAH mRNA, exerts regulatory control over mRNA degradation.

## Discussion

Recent studies have demonstrated the significant impact of RNA modifications, specifically m6A modifications, on cancer development [18]. These modifications influence post-transcriptional gene expression through epigenetic mechanisms, ultimately determining the functional properties of proteins. For example, METTL3 has been found to play a role in the progression of colorectal cancer by targeting the m6A-BHLHE41-CXCL1/CXCR2 axis, resulting in the inhibition of antitumor immunity [36]. These findings underscore the regulatory effects of RNA m6A modifications and their associated factors on tumor initiation and progression. Therefore, investigating the biological functions and potential molecular mechanisms of m6A and its associated factors has significant implications and prospects for medical advancements.

Indeed, exploring the role of m6A modification in pediatric cancers holds great potential. m6A modification is present in various pediatric cancers [19–23], indicating its potential as a therapeutic target in pediatric cancer treatment. The interaction between the RNA N6-methyladenosine reader IGF2BP3 and MYCN plays a crucial role in promoting NB cell proliferation [37]. Similarly, blocking the FTO/m6A/MYC/CEBPA-associated signaling pathway using R-2HG has shown promise in inhibiting the rapid development of glioma [38]. In osteosarcoma, WTAP inhibits the expression of its potential target gene HMBOX1 through participation in m6A modification, significantly accelerating osteosarcoma development by regulating the PI3K/AKT pathway [39]. These findings highlight the importance of m6A modification and its associated factors in pediatric cancers, suggesting potential avenues for targeted therapies.

In our study, a strong correlation was found between METTL14 expression and both disease-free survival (DFS) and OS in NB patients. Functionally, cell proliferation assays were performed, and demonstrated that METTL14 knockdown markedly decreased the proliferation and colony-forming abilities of NB cells. Conversely, the overexpression of METTL14 led to a significant increase in both the colony-forming and proliferation capacities of NB cells. These findings demonstrate that METTL14 is a potential NB promoting factor. Mechanistically, METTL14 knockdown significantly influenced the expression of 1154 genes. Furthermore, through the integration of RNA-seq and MeRIP-seq results, we confirmed that YWHAH is a shared target gene of METTL14. We conducted an analysis using data from the DEPMAP database on the expression of METTL14 and YWHAH across all cell lines. Surprisingly, the correlation analysis indicated a positive correlation between the expression of these two genes, contrary to the expected negative correlation (Supplementary Fig. 9A and Supplementary Table 20). This positive correlation is likely attributed to the intricate nature of tumorigenesis. However, upon examining multiple gene expression datasets of NB patients, we consistently observed a significant negative correlation between METTL14 and YWHAH in all these datasets (Supplementary Fig. 9B–F and Supplementary Table 21). Therefore, we suggest that while METTL14 and YWHAH may not exhibit a negative correlation in all types of tumors, there is indeed a notable negative correlation in NB tumors. Further research is warranted to explore their relationship in other tumor types. These findings elucidated the potential therapeutic targets and mechanisms involved in NB development.

YWHAH exerts bipartite regulatory effects on tumors. Numerous studies have reported a correlation between elevated YWHAH expression and various cancer types [40]. Specifically, YWHAH has been shown to exert a positive regulatory influence on large B-cell lymphoma [41] and papillary thyroid cancer [42] and to enhance the activity of gastric carcinoma (GC) cells via PI3K/AKT pathway activation [28]. These findings demonstrate that YWHAH may function as a tumor promoter. Conversely, in miR-660-5p-regulated hepatocellular carcinoma (HCC), YWHAH appears to inhibit HCC by suppressing the level of PI3K/AKT phosphorylation [43]. Studies by *P Nava* revealed that proteins belonging to the 14-3-3 family exert a remarkable inhibitory effect on the phosphorylation of AKT [44]. However, the effect of YWHAH on NB remains unexplored. Our study demonstrated that the expression of YWHAH was markedly lower in the high-risk group than in the low-risk group. Moreover, YWHAH overexpression significantly suppressed the phosphorylation of PI3K and AKT and strongly inhibited NB cells proliferation. Consequently, our study revealed that YWHAH is an inhibitor of the PI3K/AKT pathway in NB, thereby exerting a negative regulatory effect on the development of NB.

Recent reports have shown that METTL3 and WTAP, two components of the functional methyltransferase complex, can influence cell proliferation in different cancer types through the AKT signaling pathway [45]. In ovarian cancer cells, METTL3 was found to inhibit GC cell proliferation by inactivating the AKT pathway, while WTAP stimulated AKT pathway activation, leading to enhanced proliferation and migration [46]. Based on these findings, it is proposed that methyltransferase complex components may regulate downstream pathways by targeting the same molecule: AKT.

In our investigation, we aimed to examine the underlying mechanism through which METTL14 modulates biological processes in NB cell lines. We assessed the levels of phosphorylated proteins associated with the PI3K/AKT pathway. Our findings suggest that METTL14 plays a crucial role in NB by affecting the PI3K/AKT pathway. Aberrant activation of the PI3K/AKT pathway has been observed in NB and is associated with unfavorable prognostic outcomes [3]. Therefore, targeting the PI3K/AKT pathway is a potential approach for developing molecularly targeted therapies for NB. In recent years, various strategies have been reported to disrupt different components of the PI3K/AKT signaling pathway. Exploring the potential interactions between METTL14, YWHAH, and the PI3K/AKT signaling pathway may lead to innovative breakthroughs in NB treatment.

m6A-mediated modification of the 5′ UTR can regulate both translation and degradation processes. Specifically, YTHDF2 has been identified as a regulator of OCT4 mRNA degradation through its binding to the 5′ UTR of OCT4 mRNA. This interaction ultimately facilitates the development of the CSC liver phenotype and promotes tumor metastasis [47]. However, given the high similarity in amino acid sequences among the YTHDF family proteins, some researchers have suggested that they may possess functional redundancy and collectively contribute to the promotion of RNA degradation [48]. The participation of YTHDF1 in the process of RNA degradation has been extensively documented. For instance, YTHDF1 interacts with AGO2 via the YTH structural domain, potentially facilitating the interaction between AGO2 and miRNAs. Subsequently, YTHDF1 polymerizes additional components of miRISCs through LLPS, resulting in the formation of P-bodies and subsequent degradation of mRNAs [49]. Furthermore, YTHDF1 plays an important role in promoting the degradation of m6A-modified EVB viral transcription products [50]. In the context of NB, our findings indicate that YTHDF1 primarily mediates the degradation of YWHAH mRNA. In addition to the role of YTHDF proteins in RNA degradation, other recognition proteins, such as YTHDC2 [51], have been reported to regulate RNA degradation and translation during sperm maturation. Hence, we acknowledge the possibility that other recognition proteins might be involved in the degradation of YWHAH mRNA in NB. Further investigation is warranted to explore this aspect.

In conclusion, our study supports the oncogenic role of METTL14 in NB progression. We identified the involvement of the “METTL14-YWHAH-PI3K/AKT” axis, in which METTL14 promotes tumor growth and invasion in NB cells by activating the PI3K/AKT signaling pathway. These findings highlight the importance of METTL14 as a potential therapeutic target for NB treatment.

## Materials and methods

### Cell lines and mice

We purchased SK-N-BE(2), SK-N-SH, and normal cell lines 293T from the Chinese Academy of Sciences cell bank. As complete media, 10% fetal bovine serum (Dongling Biotech, Soochow, China) and 1% penicillin-streptomycin (C0222, Beyotime, China) were added to DMEM and DMED/F12 (BasalMedia, China). The cells were incubated at 37 °C in a humidified cell incubator with 5% CO_2_. Before the experiment, the cells were confirmed to be free of mycoplasma contamination.

All the animal studies at the Children’s Hospital of Soochow were approved and licensed by the Animal Care and Use Committee. BALB/c-nude mice were purchased from Cavens Model Animal Research Inc.

### Lentivirus preparation and infection

Gene overexpression is accomplished by introducing the coding sequence (CDS) region of either the METTL14 or YWHAH transcript into the pLVX-EF1α-Puro plasmid. The pLKO.1 lentiviral vector (IGE Biotechnology Co., LTD., China) is employed to knockdown the target genes. Following sequencing, purified plasmids are transfected into 293FT cells along with packaging plasmids psPAX2 and pMD2G. The packaging plasmids are combined with psPAX2 and pMD2G in a 4:3:1 ratio using the PEI (49553-93-7, Sigma-Aldrich, USA) reagent, following the protocol. After 48 h, the supernatant is collected to concentrate the virus. The cells were subsequently infected with a concentrated virus in a complete culture medium for a duration of 24 h. Following this, the cells were subjected to passaging and culturing in a medium supplemented with puromycin (60210ES25, Yesen, China) at the final concentration of 1 µM for 48 h, resulting in the establishment of a stable NB cell line expressing shRNA. These cell lines were utilized for conducting q-PCR assays, assessing cell proliferation, examining the cell cycle, as well as investigating invasion and migration capabilities. The shRNA sequences were as follows:

METTL14-shRNA-1: 5-CCGGGAACCTGAAATTGGCAATATACTCGAGTATATTGCCAATTTCAGGTTCTTTTTG-3

METTL14-shRNA-2: 5-CCGGAGGATGAGTTAATAGCTAAATCTCGAGATTTAGCTATTAACTCATCCTTTTTTG-3

ETS1-shRNA-1: 5-CCGGGTGCAGATGTCCCACTATTAACTCGAGTTAATAGTGGGACATCTGCACTTTTTG-3

ETS1-shRNA-2: 5-CCGGGACCGTGCTGACCTCAATAAGCTCGAGCTTATTGAGGTCAGCACGGTCTTTTTG-3

YTHDF1-shRNA-1: 5-CCGGATCGGTCTAAAGTGCTAATTTCTCGAGAAATTAGCACTTTAGACCGATTTTTTG-3

YTHDF1-shRNA-2: 5-CCGGCCCTACCTGTCCAGCTATTACCTCGAGGTAATAGCTGGACAGGTAGGGTTTTTG-3

YTHDF2-shRNA-1: 5-CCGGCGGTCCATTAATAACTATAACCTCGAGGTTATAGTTATTAATGGACCGTTTTTG-3

YTHDF2-shRNA-2: 5-CCGGCCACAGGCAAGGCCCAATAATCTCGAGATTATTGGGCCTTGCCTGTGGTTTTTG-3

YTHDF3-shRNA-1: 5-CCGGTAAGTCAAAGAAGACGTATTACTCGAGTAATACGTCTTCTTTGACTTATTTTTG-3

YTHDF3-shRNA-2: 5-CCGGGATAAGTGGAAGGGCAAATTTCTCGAGAAATTTGCCCTTCCACTTATCTTTTTG-3

### Real-time quantitative PCR

A total RNA extract of NB cells was prepared using TRIzol reagent (15596026, Invitrogen, USA). The reverse transcription of RNA was performed using the HiScript® II Q Select RT SuperMix for qPCR (+gDNA wiper) (R233-01, Vazyme, China) as the provided protocol. The cDNA was used for qualitative real-time PCR using the Hieff® qPCR SYBR Green Master Mix (11202ES03, YEASEN, China). The usage of primers for RT-qPCR were as follows: GAPDH forward, 5-TGCACCACCAACTGCTTAG-3, reverse, 5-GATGCAGGGATGATGTTC-3; METTL14 forward, 5-AGAGAAACTGGCATCACTGCT-3, reverse, 5-TCGTAAACACACTCTTCCAAGG-3; YTHDF1 forward, 5-TGTGGAATGAGGGACCGTTG-3, reverse 5-GGATCCTCTACAAGGGCACG-3; YTHDF2 forward 5-GCGACACATTCGCCTAGAGA-3, reverse 5-GGAAGTGGTGTGCTTGTAGC-3; YTHDF3 forward 5-TTTTCATCAGCGCATCTGCC-3, reverse 5-TCTGTGCCTTAGCTAGGATGC; YY1 forward 5-ACGGCTTCGAGGATCAGATTC-3, reverse 5-TGACCAGCGTTTGTTCAATGT-3; YWHAH forward 5-GACATGGCCTCCGCTATGAAG-3, reverse 5-ATGCTGCTAATGACCCTCCAG-3.

### Western blot

After digestion with trypsin (C0201, Beyotime, China), the cells were washed twice in phosphate-buffered saline (PBS), then lysed with protein-loaded buffer (20315ES05, YESEN, China) and boiled at 100 °C for 10 min. The separation of same amounts of proteins was in sodium dodecyl sulfate-polyacrylamide gel electrophoresis (SDS-PAGE) and transferred to nitrocellulose membranes, and subjected to immunoblotting analysis with anti-GAPDH (60004-1-Ig, Proteintech, China), anti-METTL14 (26158-1-AP, Proteintech, China), anti-ETS1 (12118-1-AP, Proteintech, China), anti-YY1 (22156-1-AP, Proteintech, China), and anti-YTHDF1 (17479-1-AP, Proteintech, China), anti-YTHDF2 (24744-1-AP, Proteintech, China), anti-YTHDF3 (25537-1-AP, Proteintech, China), anti-Cyclin D1 (26939-1-AP, Proteintech, China), anti-Cyclin E1 (11554-1-AP, Proteintech, China), anti-PI3K (20584-1-AP, Proteintech, China), anti-phospho-PI3K (4228T, cell signaling, USA), anti-AKT (60203-2-Ig, Proteintech, China), anti-phospho-AKT(28731-1-AP, Proteintech, China). The polyvinylidene difluoride (PVDF) membrane was incubated with the secondary antibody at room temperature for one hour, followed by visualization using the ECL luminescent solution (P1050, Applygen, China) and the AI600 image gel imaging analyzer.

### Clone formation assays

Tumor cells at the logarithmic phase were subjected to digestion using a 0.25% trypsin solution to get a single-cell suspension in the presence of culture medium. Using an inverted microscope, a cell counting chamber was used to determine how many cells were in a 10 µl single-cell suspension. The resulting cells were then seeded into 6-well plates, with each well receiving 500–1000 cells. It was necessary to replace the culture medium every three days until visible clones of the cells were formed.

### Cell-cycle assays

The samples utilized for cell cycle experiments were subjected to overnight fixation with a 75% alcohol solution prior to the introduction of RNase A (ST578, Beyotime, China) and PI. After 30 min incubation in the dark environment, the cells were evaluated using flow cytometry.

### In vivo experiments

The animal studies were performed according to approved protocols and guidelines provided by the Institutional Animal Care and Use Committee. To investigate the impact of METTL14 on the growth of subcutaneous xenografts, BALB/c nude mice (3 × 6) (Beijing Vital River Laboratory Animal Technology) were subjected to subcutaneous injection of 0.1 mL cell suspension with 2 × 10^6^ cells. At the conclusion of a 30-day period, the mice were euthanized, and the experiment was concluded. The tumors were subsequently isolated, weighed, and photographed.

In one animal experiment, we selected female nude mice of the same sex, age, and similar weight to minimize the impact of sample differences on experimental results. The animal experiment in this study did not use a blind design.

### Cell proliferation assay

The Cell Counting Kit‐8 (CCK‐8) assay (C0037, Beyotime, China) was utilized to test the cell viability. Cells were initially seeded into a 96‐well plate and incubated for 24, 48, 72, 96, and 120 h. Subsequently, 10 μL of CCK‐8 solution was introduced into each well and incubated at 37 °C for a duration of 2 h. The absorbance at wavelength of 450 nm was measured by the microplate reader (SpectraMax® M5, Molecular Devices).

### EdU test

After culture in a 6-well plate, the NB cells were stained using the BeyoClick^TM^ EdU‐488 kit (C0071S, Beyotime, China) as the instructions. Fluorescence microscopy was then used to examine the stained cells. The Image J software (National Institutes of Health) was employed to quantify the proportion of positive cell nuclei to the total number of cell nuclei.

### Dual luciferase

The YWHAH promoter and wild type/mutant 5′UTR sequences were amplified and inserted into pGL3 vectors. SK-N-BE(2) cells were transfected with 1600 ng of YWHAH-Luc and 20 ng of Renilla luciferase plasmid pRL-TK using M5 Hiper Lipo2000 Transfection Reagent (MF135-01, Mei5bio, China). Using a double-luciferase assay kit (11402ES60, YEASEN, China) according to the instructions, luciferase activity was tested after 48 h.

### RNA m6A quantification

Following the provided instructions, the m6A modification extent of the total RNA was detected using the EpiQuik m6A RNA Methylation Quantification Kit. In brief, a quantity of 200 ng RNA was introduced into an analytical well, together with the m6A standard solution, and subsequently subjected to treatment with the capture antibody solution and determination antibody solution. The quantification of m6A level was accomplished through colorimetry, by measuring the absorbance of each well at a wavelength of 450 nm, followed by calculation based on the standard curve.

### RNA immunoprecipitation (RIP)-qPCR

The RIP assays were conducted using the RNA Immunoprecipitation (RIP) Kit (P0101, Geneseed, China) in accordance with the protocols. Briefly, a total of 2 × 10^7^ NB cells were harvested and cleaved using RIP lysis buffer. After centrifugation at 4 °C, the precipitation was abandoned and then collected the supernatant, followed by incubation for 1.5 h at room temperature with specific antibodies and the negative control, IgG. Subsequently, after washing the protein magnetic bead-antibody complex, the immunoprecipitated RNA was purified and tested by RT-PCR.

### m6A-RNA immunoprecipitation assay (MeRIP-qPCR)

The m6A modification level of mRNA was detected by the MeRIP-qPCR method with the Magna MeRIP Kit (17-10499, Merck, Germany) following the protocols. In brief, m6A antibody was incubated with magnetic beads, then 100 µg total RNA was incubated with magnetic beads in RNase-inhibiting immunoprecipitation buffer overnight at 4 °C. RNA bound to m6A antibody is digested with protease K, precipitated, and the enrichment of m6A RNA is quantified using qPCR and normalized to the input.

### ChIP-qPCR

SK-N-BE(2) and SK-N-SH cells were cross-linked with 1% fresh formaldehyde for 15 min at room temperature, neutralized with glycine for 5 min, and lysed in lysis buffer. The cross-linked DNA was then sheared into fragments ~200–1000 bp in length. Sheared chromatin was immunoprecipitated with anti-YY1, anti-ETS1 antibodies using protein A/G agarose (Pierce, 20421, USA). Mouse IgG (Cell Signaling Technology, 5946, USA) was used as a mock antibody for negative control. Finally, the immunoprecipitated DNA was de-cross-linked and isolated. ChIP-qPCR were performed with the Hieff® qPCR SYBR Green Master Mix (Vazyme, R233-01, China). Antibody binding signals were calculated as a percentage of input chromatin precipitated for each region examined. ChIP-qPCR primers were designed for the METTL14 promoter based on the YY1 and ETS1 binding site. The usage of primers for ChIP-qPCR were as follows: YY1-F 5-ATCCTATGAAGACAGAATCAC-3, YY1-R 5-ATGTCCTAGGAGTCAGAAGCTA-3; ETS1-F 5-ACGAAGGTGGTTAACTAGCC-3, ETS1-R 5-AGATGACAGTCCTGACGTTT-3; ACTB-F 5-CATGTACGTTGCTATCCAGGC-3, ACTB-R 5-CTCCTTAATGTCACGCACGAT-3.

### Migration and invision

The cells were subjected to trypsinization, followed by resuspension in a serum-free medium, and subsequently seeded into chambers with the 8-mm pore size polycarbonate membrane (3422, Corning, USA). Matrigel (356234BD, Biosciences, USA) was used to coat the chambers in order to facilitate cell invasion assays. The concentration of cell suspension was diluted to 1 × 10^5^ cells/mL, subsequently 100 µl of the cell suspension was evenly distributed into each well through gentle shaking. In the lower chamber, 750 ml of medium containing 20% FBS was introduced. Following an incubation period of 48 h, the cells residing on the upper surface of the insert were cleared using the cotton swab. The migration of cells to the lower surface of the insert were then stained with 0.1% crystal violet for a duration of 30 minutes and were rinsing with PBS and subsequent microscopic examination. Triplicate assays were performed for each experiment.

### Establishment multi-gene prognostic signature

Therapeutically Applicable Research to Generate Effective Treatments (TARGET) is an open database for childhood cancers. The gene expression and clinical data of neuroblastoma from the TARGET database. Log-rank test was used to compare differences in survival between these groups. The time ROC (v 0.4) analysis was used to compare the predictive accuracy of risk score. The least absolute shrinkage and selection operator (LASSO) regression algorithm for feature selection, using 10-fold cross-validation, the above analysis uses the R software package glmnet (v 4.1-1). Lasso: The least absolute shrinkage and selection operator (LASSO) regression algorithm was used for feature selection, 10-fold cross-validation was used, and the R package glmnet was used for the analysis. Cox: Multivariate cox regression analysis was used to construct a prognostic model, and the R package survival used for the analysis. Step: First, the multi-factor cox regression was used to analyze the data, and then the step function performed the iteration. Finally, the optimal model is selected as the final model. For Kaplan–Meier curves, *p*-values and hazard ratio (HR) with 95% confidence interval (CI) were generated by log-rank tests and univariate cox proportional hazards regression. All the analysis methods and R packages were implemented by R (foundation for statistical computing 2020) version 4.0.3. *p* value < 0.05 was considered statistically significant.

### Gene correlation analysis

All microarray data was downloaded from the GEO database (http://www.ncbi.nih.gov/geo). The raw data were downloaded as MINiML files. Box plots are drawn by boxplot; the R software ggord package was used to draw PCA plot, the R software ggstatsplot package was used to draw two-gene correlation, the R software pheatmap package was used to draw multi-gene correlation. Used Spearman’s correlation analysis to describe the correlation between quantitative variables without a normal distribution. *P* values less than 0.05 were considered statistically significant (*P < 0.05).

### Statistical analysis

All data are presented as mean + standard error of the mean (SEM) derived from at least three independent biological replications unless otherwise indicated. To test for differences between two groups of samples, a student’s t-test was used. *P*-values less than 0.05 were considered statistically significant for all two-sided tests.

### Supplementary information

Supplementary materials

Dataset 1

## Data Availability

Raw meRIP-seq data of all samples were uploaded to the Gene Expression Omnibus (GEO) of the National Center for Biotechnology Information (GSE244350). RNA-seq has been deposited in GEO database under the accession number (GSE244657). The mRNA expression data can be obtained from the Cancer Cell Line Encyclopedia (CCLE) database (https://portals.broadinstitute.org/ccle/).
