# Supplementary material for: METTL14 promotes neuroblastoma formation by inhibiting YWHAH via an m6A-YTHDF1-dependent mechanism
Source: Cell Death Discov. 2024 Apr 22;10:186. doi: 10.1038/s41420-024-01959-8 (PMC11035551; doi:10.1038/s41420-024-01959-8)

Figure 2A

METTL14

GAPDH

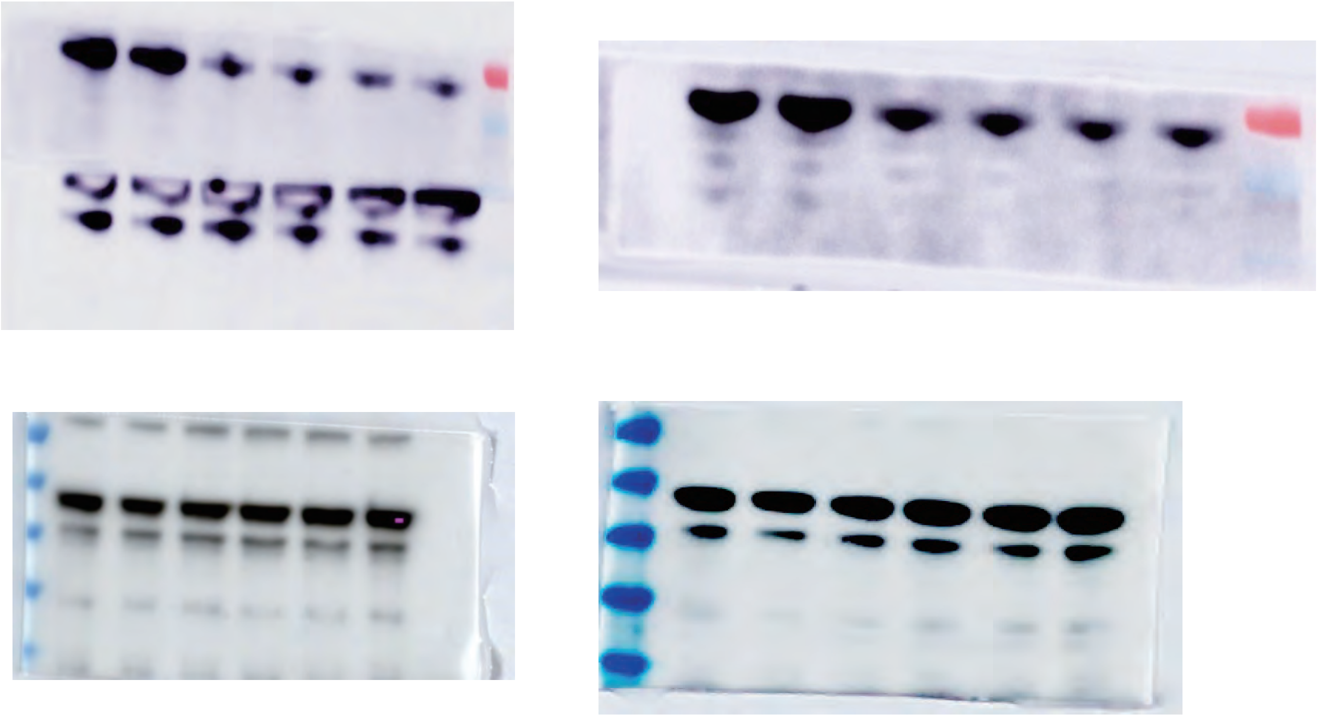

Figure 2F

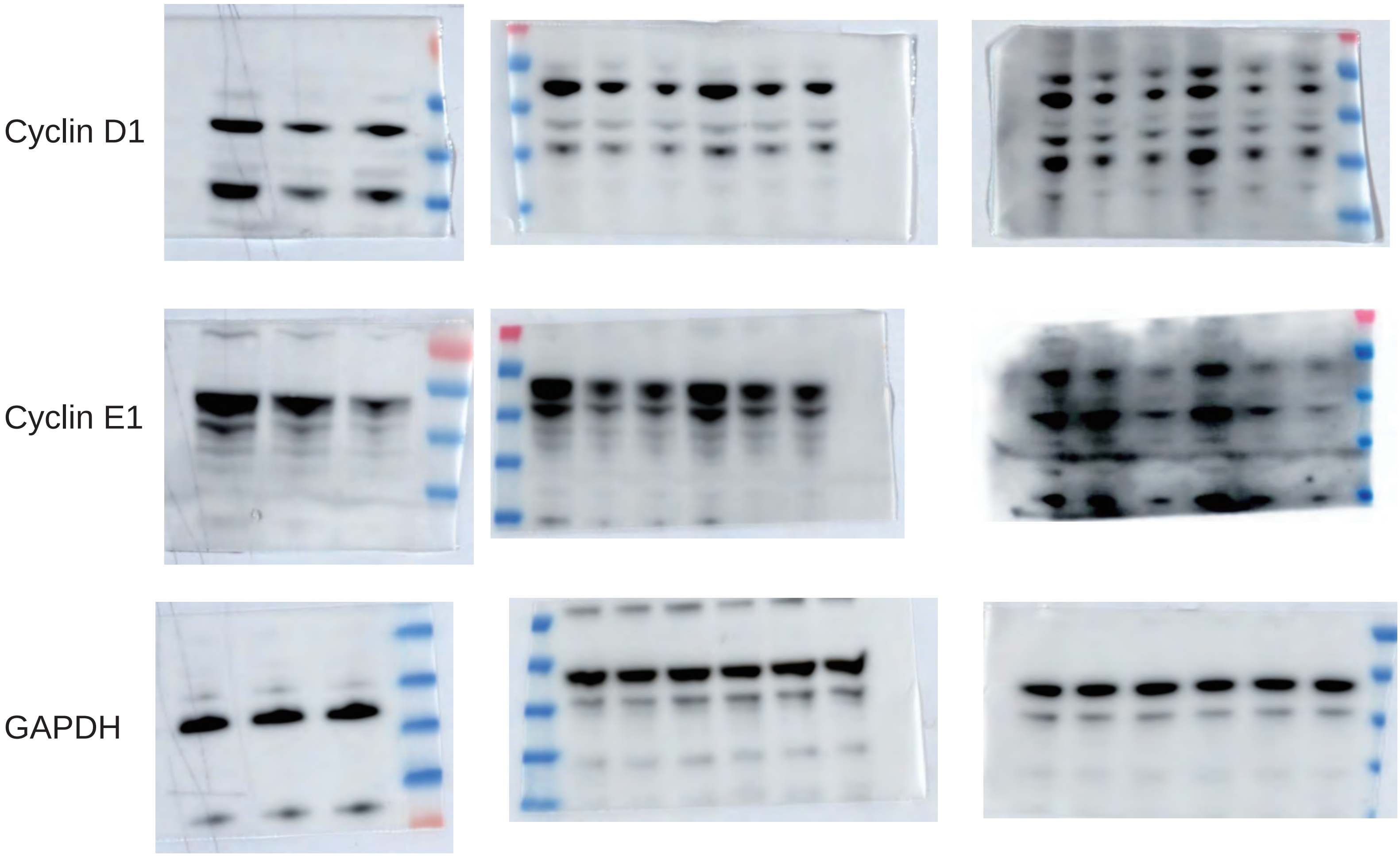

Figure 4C

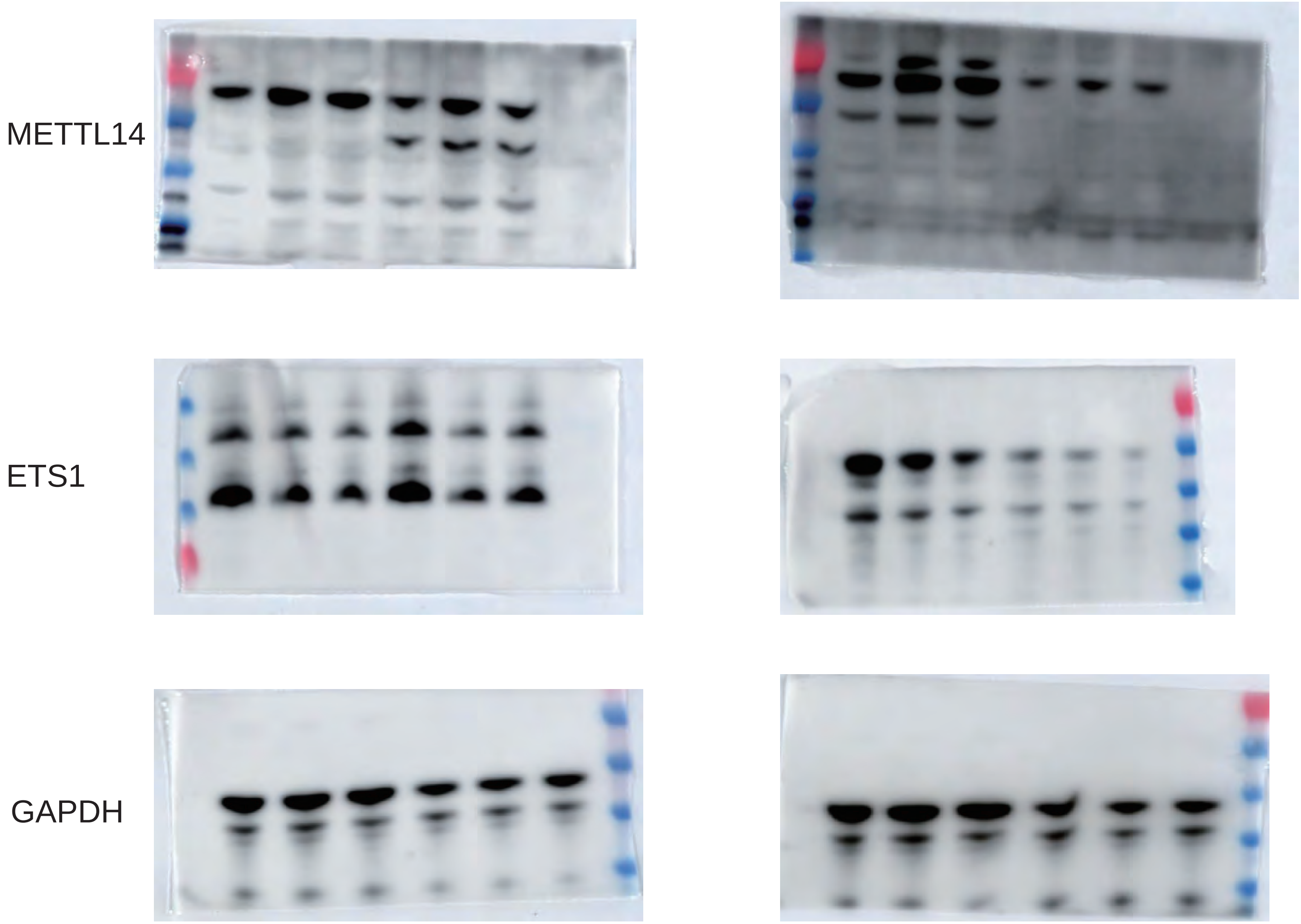

Figure 5E

YWHAH

GAPDH

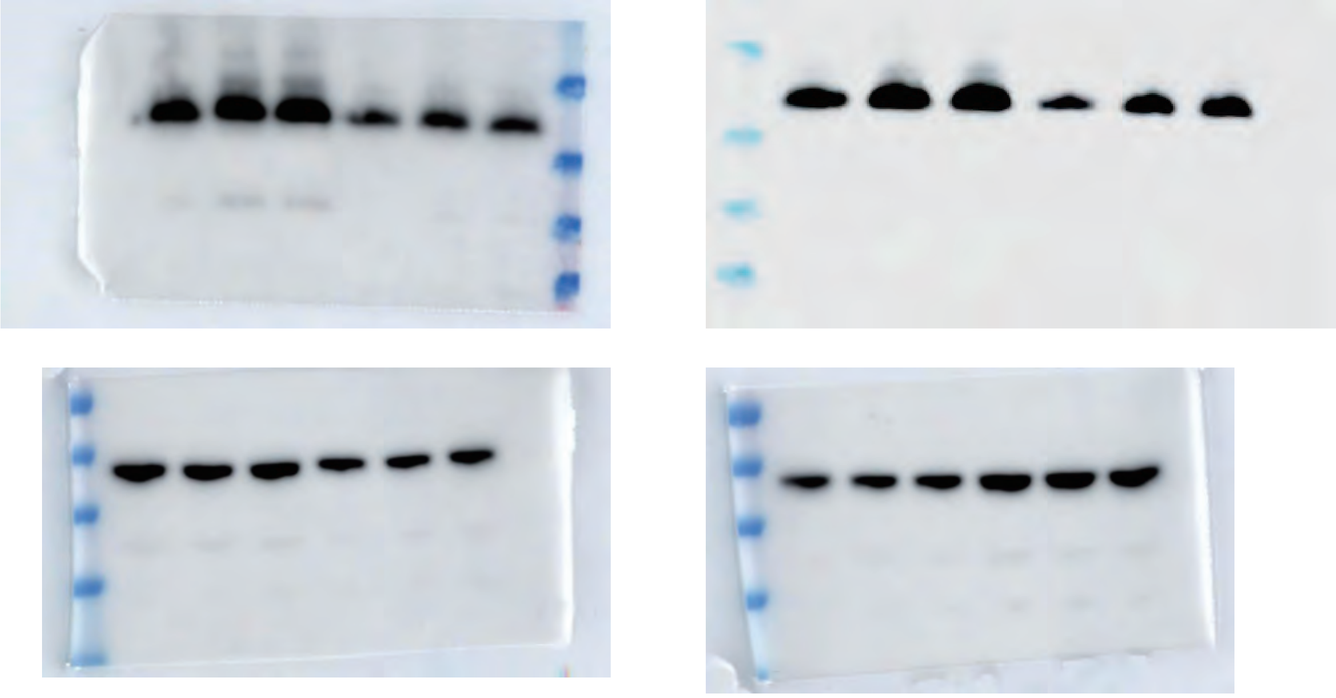

Figure 6A

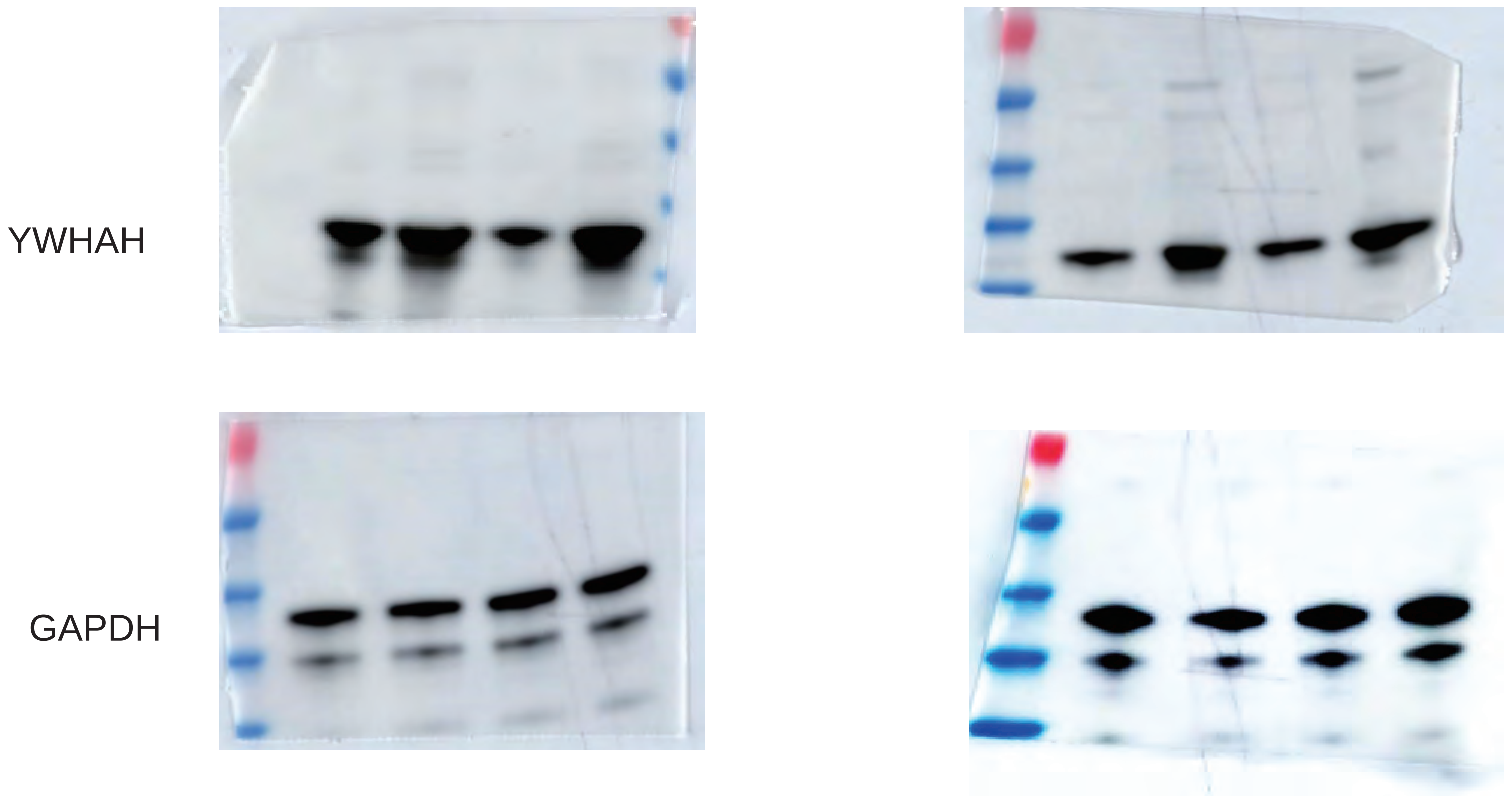

Figure 6F

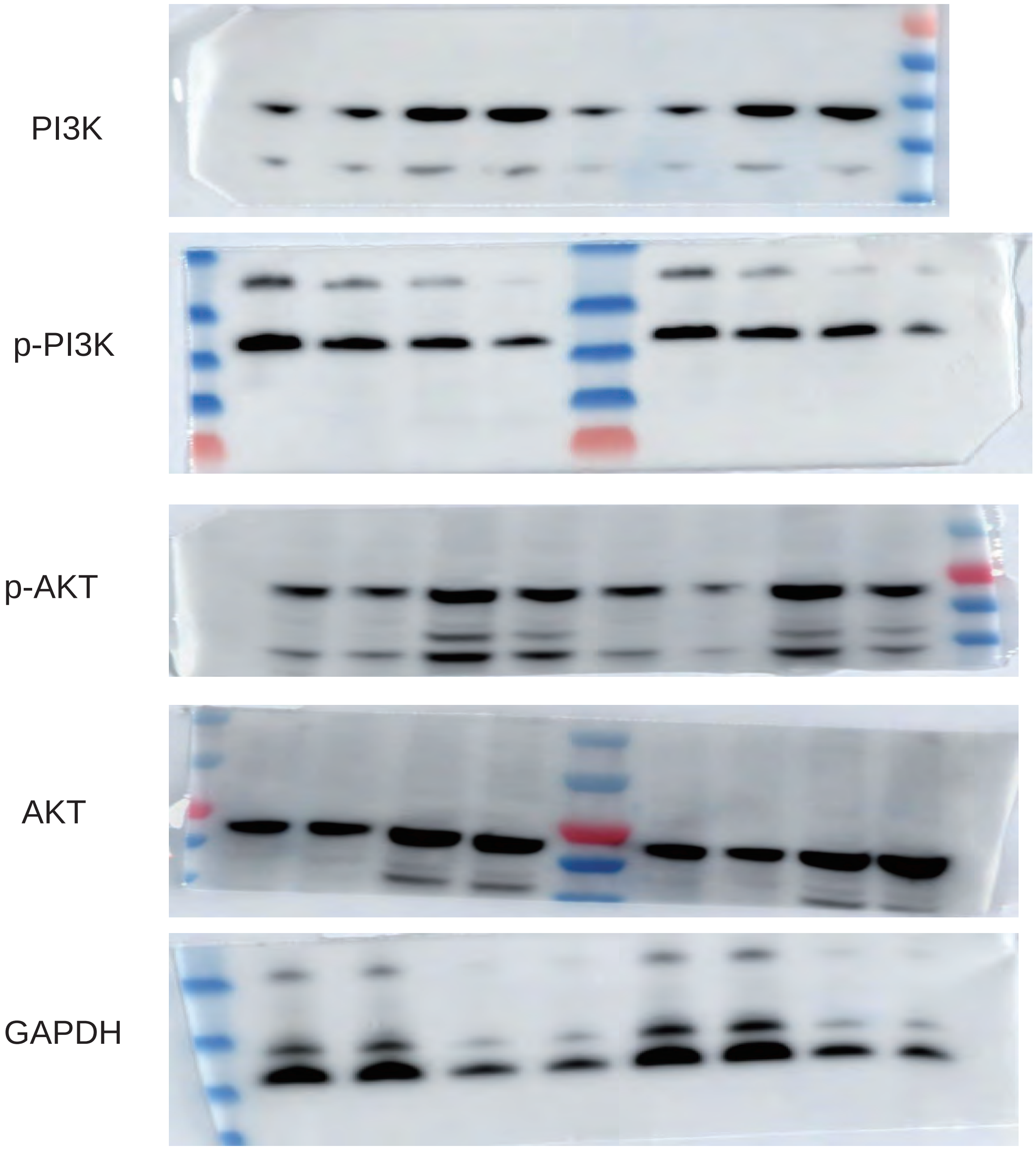

Figure 6F

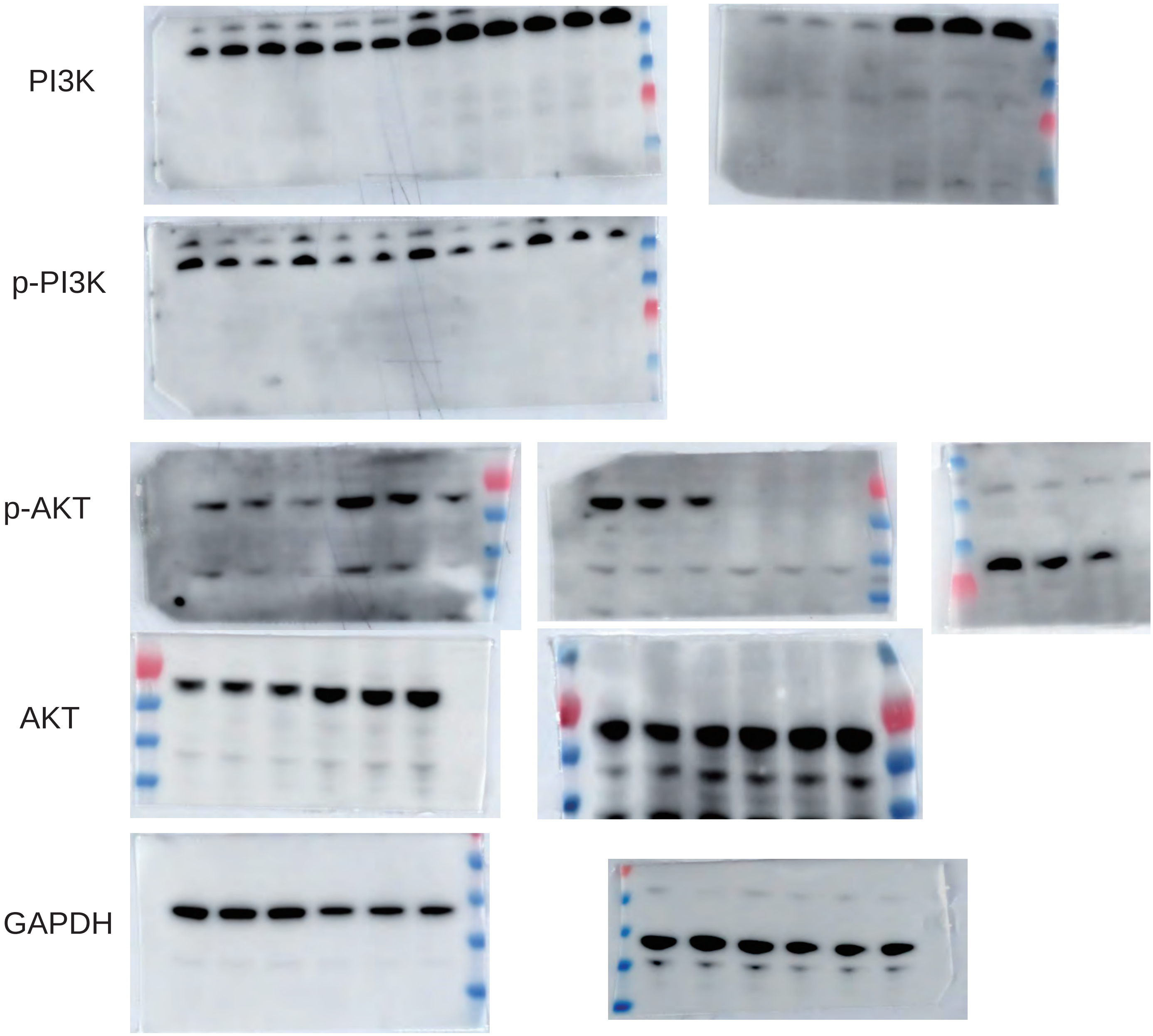

Figure 6G

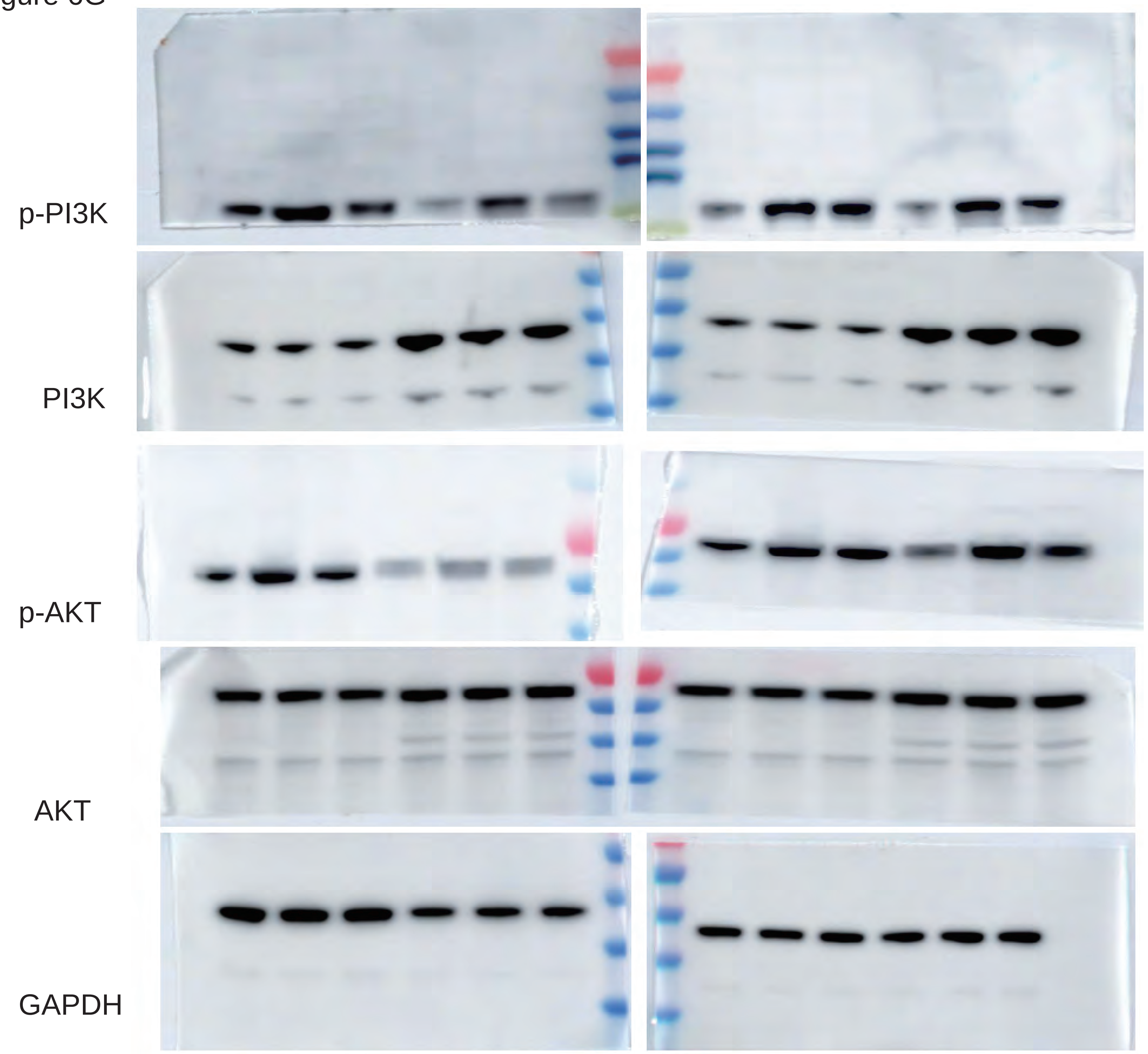

Figure 7A

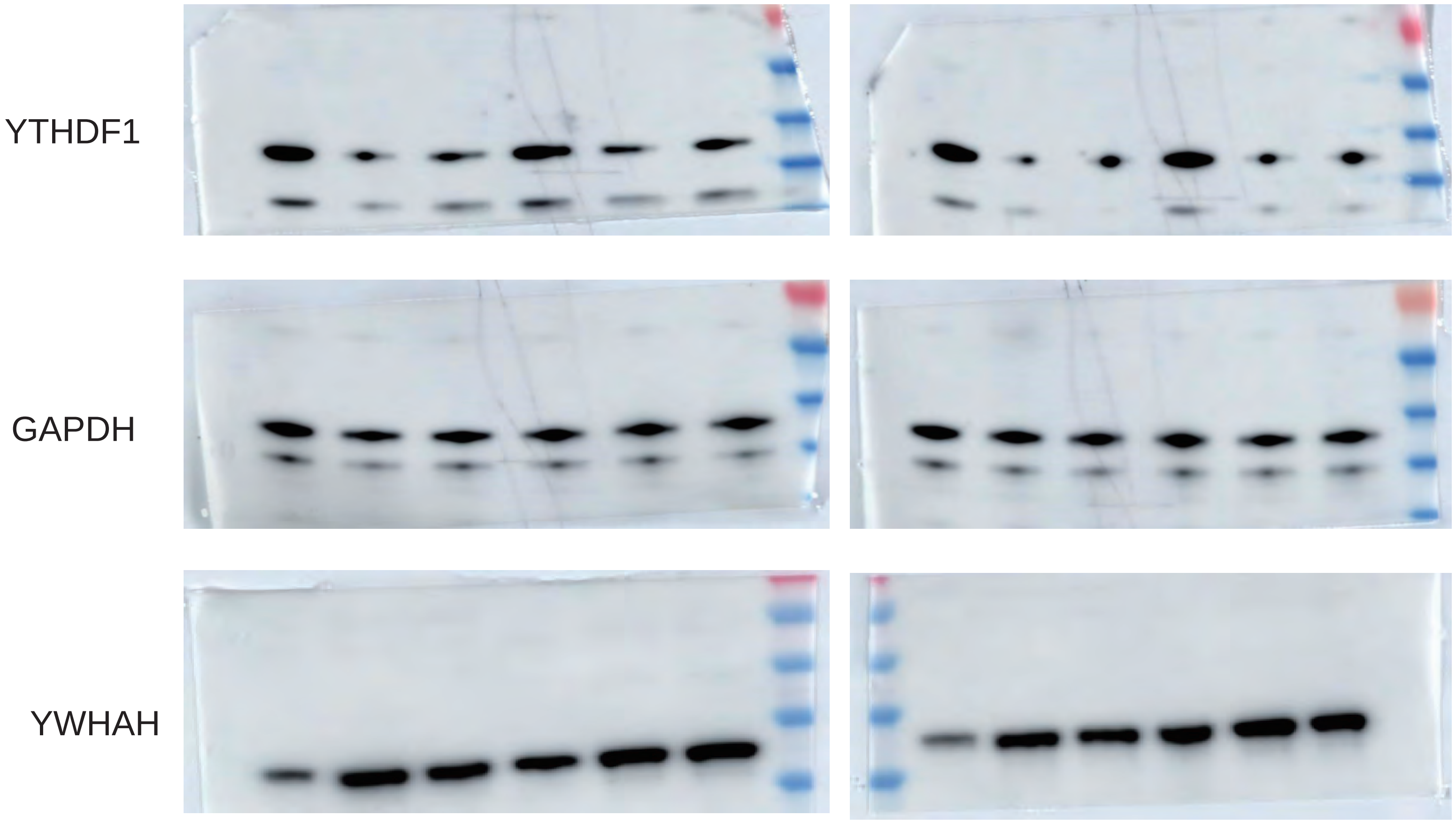

supplementary figure 1

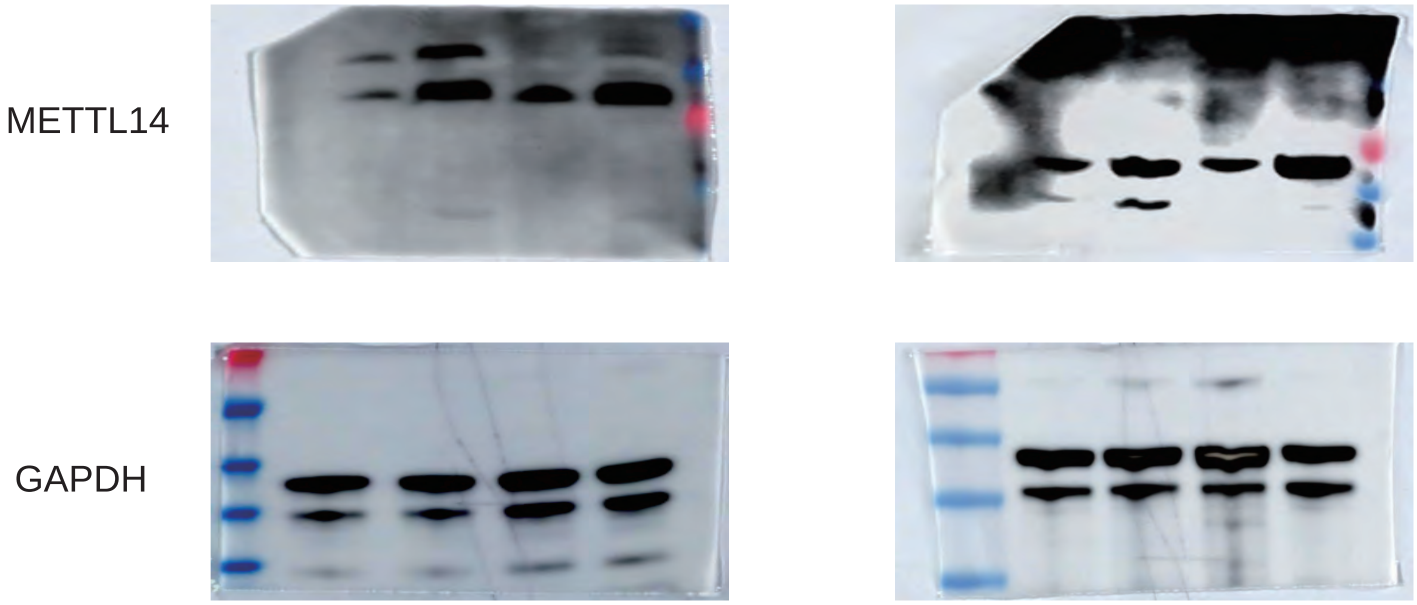

supplementary figure 7B

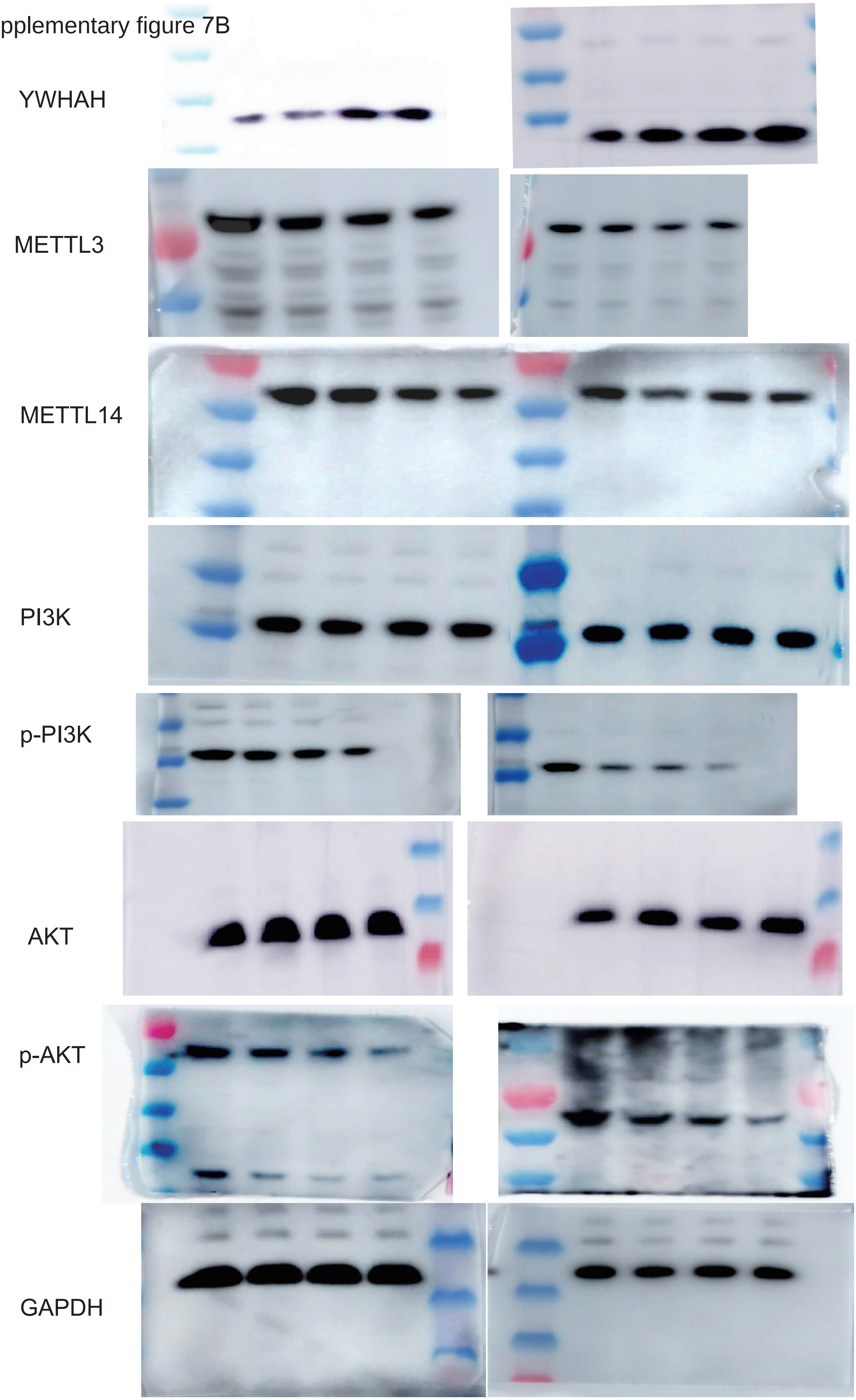

supplementary figure 8A

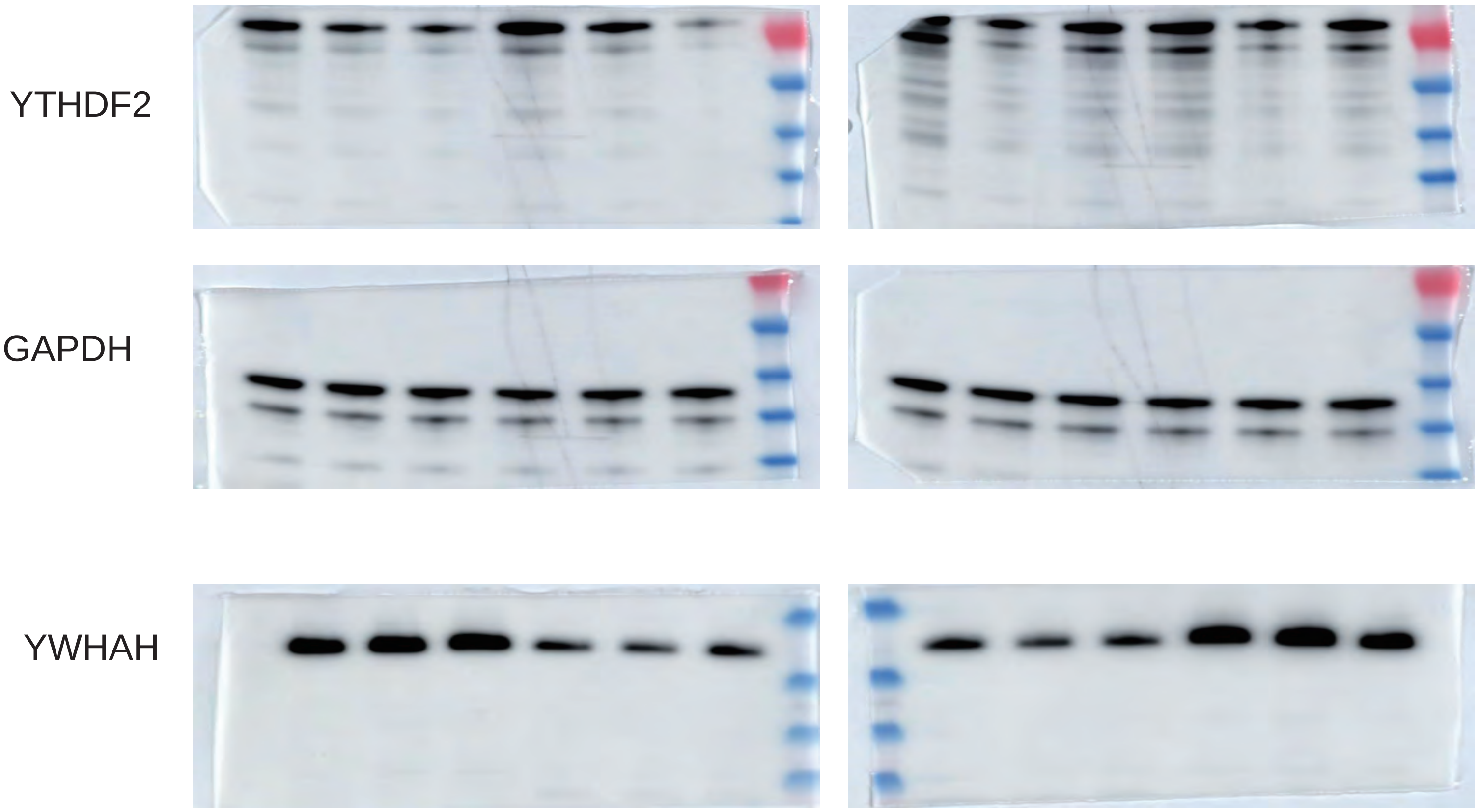

supplementary figure 8B

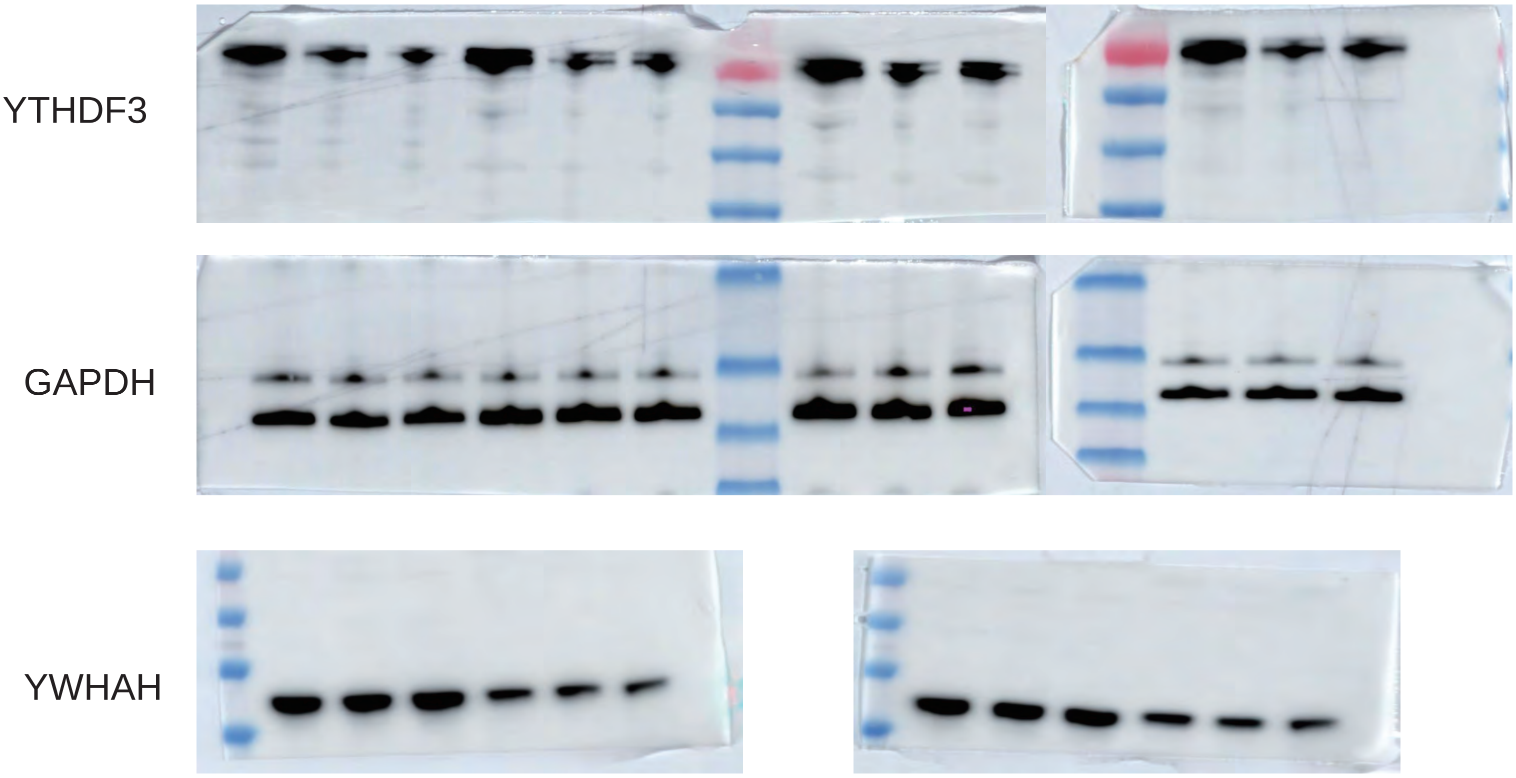

Supplement: Supplementary file 2 — Dataset 1 [file 41420_2024_1959_MOESM2_ESM.pdf]
