# Supplementary material for: METTL14 promotes neuroblastoma formation by inhibiting YWHAH via an m6A-YTHDF1-dependent mechanism
Source: Cell Death Discov. 2024 Apr 22;10:186. doi: 10.1038/s41420-024-01959-8 (PMC11035551; doi:10.1038/s41420-024-01959-8)
Supplement: Supplementary file 1 — Supplementary materials [file 41420_2024_1959_MOESM1_ESM.pdf]

Author contribution Statement

Jian Pan and Zimu Zhang designed and directed the study; Jianwei Wang did most of the experiments, analyzed the data and wrote the paper; Hongli Yin, Gen Li, Di Wu and Yunyun Xu provided assistant in the statistical analysis and supervised the writing of the paper; Xiaodong Wang, Yanling Chen and Ting Zhang took part in animal experiments; Yujiao Xing, Danhong Fei and Pengcheng Yang helped with the statistical analysis; Fang Fang helped with western blot experiments; Yanfang Tao and Xiaolu Li supervised the the paper revision; Juanjuan Yu and Yang Yang took part in the preparation and transfection of lentivirus; Zhiheng Li and Jianmin Wang helped and supported with cell apoptosis and cycle analysis; Lei Shi and Tiandan Li took part in CCK8 test and Western blotting detection. Final draft read and approved by all authors.

March 20, 2024

Dear Jianwei Wang,

Thank you for choosing Springer Nature Author Services. This manuscript, titled "METTL14 promotes neuroblastoma formation by inhibiting YWHAH via an m6A-YTHDF1-dependent mechanism," is very interesting. The paper was edited for grammar, phrasing, and punctuation. In addition, many edits were made to further improve the flow and readability of the text. Below, we highlight the areas of this paper that we focused on in our edit.

In cases where the meaning of the text was not clear, revisions were made to convey the information with increased clarity and reduced ambiguity.

Some edits were made to improve conciseness by trimming unnecessary words and streamlining the flow of your manuscript.

Certain edits were made to remove redundant, repetitive or unnecessary phrasing and to present the information in a more straightforward manner.

Comments were left if further clarification would be helpful or confirmation of the meaning of the text was necessary. Please review these comments and all our changes carefully for more detailed suggestions, as well as to ensure that the final version of the manuscript is fully accurate.

Thank you again for using our editing services; we wish you the best of luck with your submission.

Best regards,

Philip M.
Senior Editor
Springer Nature Author Services

Cell Line Authentication Report

Cell Line Authentication Report

Customer: Zimu Zhang

Quotation Number: 80-982599547_R3

Completion Date: 07/29/2022

1. **Sample ID: BE2**
2. **Original Material: Cell pellet**
3. **Methods:**

1).Genomic DNA was extracted from the cell pellets provided by the customer.

2).Samples, together with positive and negative control were amplified using GenePrint 10 System (Promega).

3). Amplified products were processed using the ABI3730xl Genetic Analyzer.

4). Data were analyzed using GeneMapper4.0 software and then compared with the ATCC, DSMZ, JCRB and RIKEN databases for reference matching.

4. **Results:**

1) 10 Loci STR Profile：

Genetic Site	Cell Bank information		Customer sample	
(Locus)	SK-N-BE(2)		BE2	
Amelogenin	X	Y	X	Y
CSF1PO	10	10	10	10
D13S317	11	11	11	11
D16S539	9	11	9	11
D5S818	12	12	12	12
D7S820	9	10	9	10
THO1	6	7	6	7
TPOX	8	11	8	11
vWA	18	18	18	18
D21S11			30	32.2
Percent match between the sample and the database profile：100%				

Summary:

Your cell line is considered to be “identical” to the reference cell line in the Cell Bank STR database, as the STR profile yields a 100% match.

Notes：

1. P=100% x (2xM)/N; M=18, N=36 P=100% x (2x18)/36=100%

M: number of the matching peaks; N: number of all peaks

2. Based on the ANSI Standard, cell lines with ⩾80% match are considered to be related; i.e., derived from a common ancestry. Cell lines with between a 55% to 80% match require further profiling for authentication of relatedness.

3. The short tandem repeat (STR) profile generated by Azenta is indicative only of the sample sent to Azenta at the time it was sent. This data and analysis are for research use only.

2) Electrophoretogram

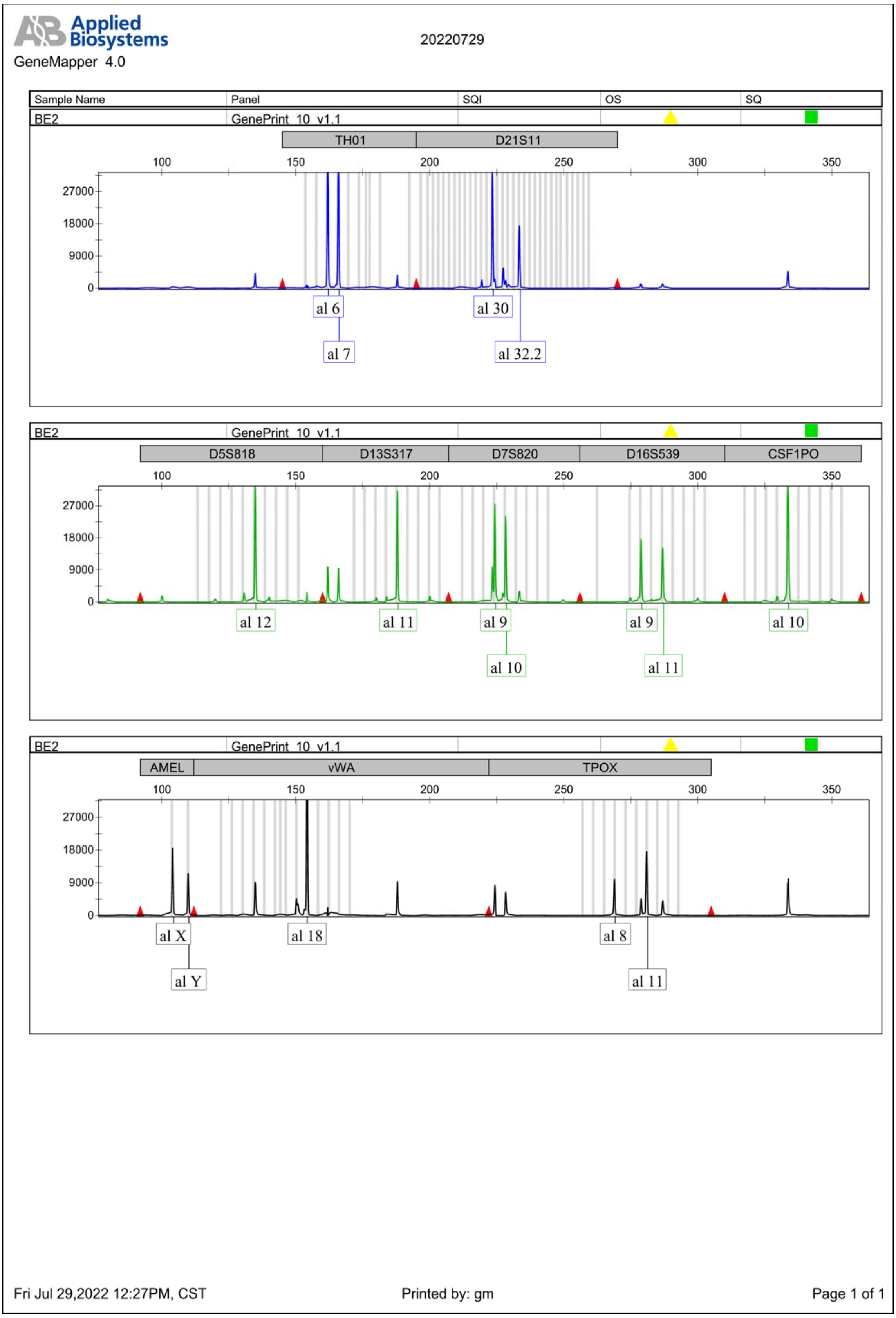

Note：Raw data in appendix

GENEWIZ®

Solid science. Superior service.

Cell Line Authentication Report

GENEWIZ, Inc.

C3 Building, 218 Xinghu Road

Suzhou Industrial Park, 215123

Suzhou, China

Tel: 400-8100-669

www.genewiz.com

GENEWIZ®
Solid science. Superior service.

苏州金唯智生物科技有限公司
中国苏州工业园区
星湖街218号纳米科技园C3楼 215123
电话:0512-68731001
传真:0512-62629530
www.genewiz.com.cn

Cell Line Authentication Report

Customer:Zhang zimu

Institution: Children's Hospital of Soochow University

Quotation Number: 80-222202365

Completion Date：11/09/2018

1. **Sample ID: SK-N-SH**
2. **Original Material: Cell pellets**
3. **Methods:**

1).Genomic DNA was extracted from the cell pellets provided by the customer.

2).Samples, together with positive and negative control were amplified using GenePrint 10 System (Promega).

3). Amplified products were processed using the ABI3730xl Genetic Analyzer.

4).Data were analyzed using GeneMapper4.0 software and then compared with the ATCC, DSMZ or JCRB databases for reference matching.

4. **Results:**

1) **10 Loci STR Profile：**

Genetic Site	ATCC		Customer sample	
(Locus)	SK-N-SH		SK-N-SH	
Amelogenin	X	X	X	X
CSF1PO	11	11	11	11
D13S317	11	11	11	11
D16S539	8	13	8	13
D5S818	12	12	12	12
D7S820	7	10	7	10
THO1	7	10	7	10
TPOX	8	11	8	9.3 11
vWA	14	18	13	14 18
D21S11			31	31.2
Percent match between the sample and the database profile：94.75%				

Summary:

Your cell line is considered “related” to the reference cell line in the ATCC STR database, as the STR profile yields matches that are ≥ 80% but less than 100%.

GENEWIZ

Notes：

1. P=100% x (2xM)/N; M=18, N=38P=100% x (2x18)/38=94.75%

M: number of the matching peaks;　　N: number of all peaks

2. Based on the ANSI Standard, cell lines with ≥80% match are considered to be related; i.e., derived from a common ancestry. Cell lines with between a 55% to 80% match require further profiling for authentication of relatedness.

3. The short tandem repeat (STR) profile generated by GENEWIZ Inc. is indicative only of the sample sent to GENEWIZ Inc. at the time it was sent. This data and analysis are for research use only.

GENEWIZ

2) Electropherogram

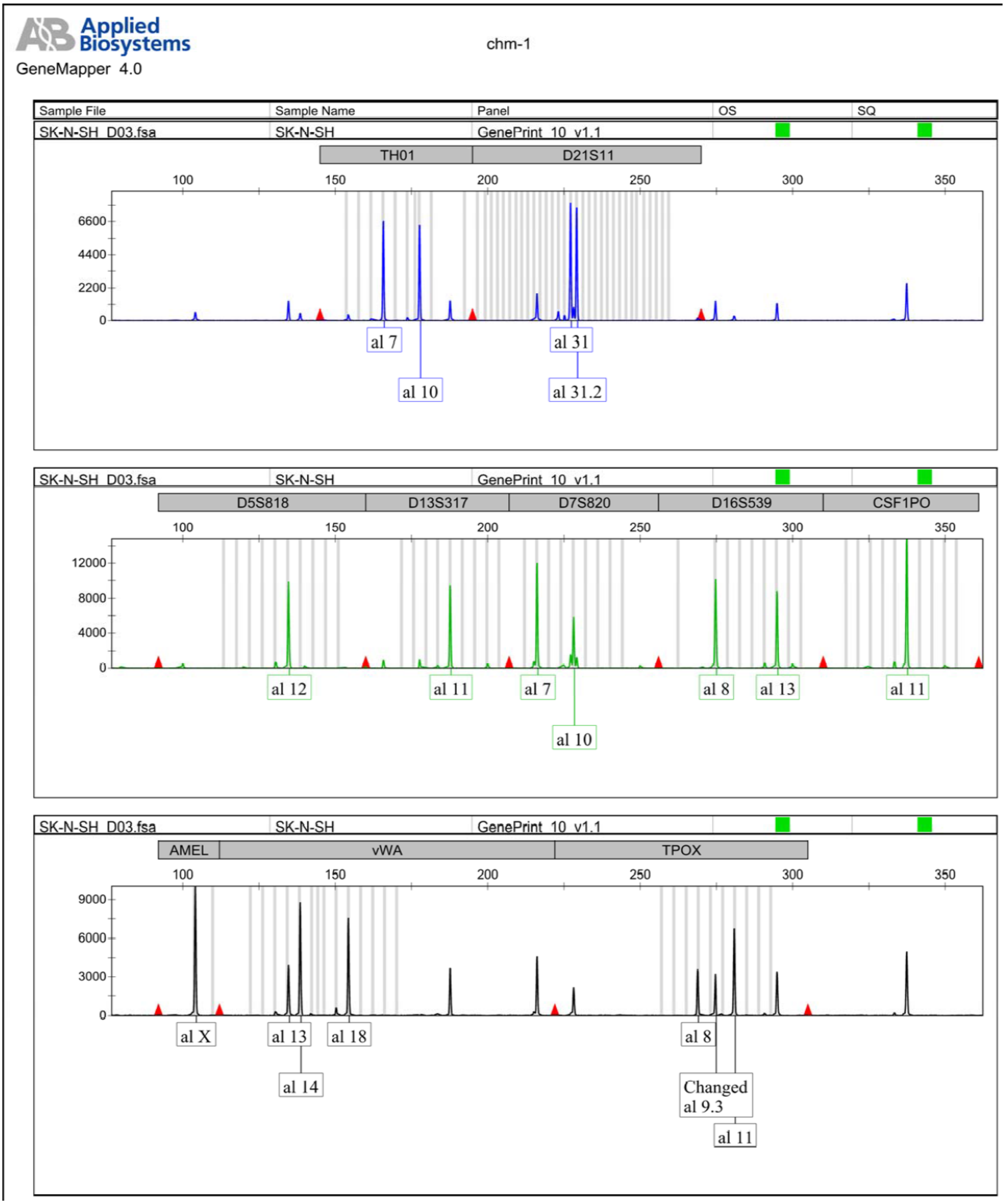

Note：Raw data in appendix

Supplementary Figure Legends:

Supplementary Figure 1. Overexpression of METTL14 promotes the neuroblastoma cells activity. (A) The comparison of METTL14 expression in different tumor cells. Data from CCLE datasets. (B) The overexpression of METTL14 in NB cells was detected by WB. After METTL14 is overexpressed, the cells were harvested for colony formation assay(C), CCK-8 colorimetric assay (D), migration and invasion (E). *P < 0.05; **P < 0.01; ***P < 0.001; n.s: not significant.

Supplementary Figure 2. METTL14 knockdown inhibited the migration, invasion and tumor formation in nude mouse. (A) The migration and invasion of SK-N-BE(2) and SK-N-SH cells were inhibited after METTL14 was knocked down. (B) Nude mice's weight growth curve after injection. (C) IHC detection for METTL14, Ki-67, expression was conducted in sections of tumors harvested from xenografts. All data are shown as mean ± SD (scale bars of D = 50 μm). NS, not significant; *P < 0.05; **P < 0.01. The data are based on three separate experiments.

Supplementary Figure 3. The cistromeDB database analyzes the binding of YY1 and ETS1 to the METTL14 promoter sequence. The data download link is http://share.suda-lab.com/?5KEfV.

Supplementary Figure 4. ChIP-qPCR was used to verify the binding of YY1, ETS1 and METTL14 promoter sequence.

Supplementary Figure 5. The expression of METTL14 was significantly decreased at the RNA level after YY1 was knocked down in SK-N-BE(2) (A) and SK-N-SH (B) cells. (C) The expression of YY1 in neuroblastoma. (D) The YY1 and METTL14 expression showed a significant negative correlation in NB. (E) Kaplan–Meier overall survival probability analysis of YY1 in NB. *P < 0.05; **P < 0.01; ***P < 0.001; n.s: not significant.

Supplementary Figure 6. (A) After METTL14 was knocked down, the expression of YWHAH at the RNA level was significantly increased; (B) The gray value analysis of protein expression.

Supplementary Figure 7. Regulation of NB cells by m6A inhibitor SAH. (A) SAH treatment inhibited the proliferation of NB cells; (B) SAH treatment increased YWHAH expression and inhibited PI3K and AKT phosphorylation levels.

Supplementary Figure 8. YTHDF2 and YTHDF3 cannot mediate the degradation of YWHAH mRNA. After YTHDF2 (A) and YTHDF3 (B) were knocked down, the YWHAH expression did not alter significantly at both the RNA and protein levels.

Supplementary Figure 9. Correlation analysis of METTL14 and YWHAH. (A) The correlation between METTL14 and YWHAH was analyzed using all cell lines in the DEPMAP database; (B) Different neuroblastoma datasets were used to analyze the

association between METTL14 and YWHAH.

Supplementary Table Legends:

Supplementary table 1. Expression statistics of 19 m6A-related genes in neuroblastoma high-risk and low-risk groups.

Supplementary table 2. Correlation analysis of 9 m6A-related genes in neuroblastoma.

Supplementary table 3. Prognostic models of neuroblastoma constructed by 10 m6A-related genes.

Supplementary table 4. Expression data of METTL14 in COG risk subgroups.

Supplementary table 5. Expression data of METTL14 in MKI risk subgroup.

Supplementary table 6. Expression data of METTL14 in GSE45547 dataset.

Supplementary table 7. Expression data of METTL14 in the GSE120572 dataset.

Supplementary table 8. Expression analysis of METTL14 in different tumor cells.

Supplementary table 9. Correlation analysis of METTL14 and 11 potential transcription factors in the GSE45547 and GSE49710 datasets.

Supplementary table 10. Expression of YY1 in low and high-risk patients of neuroblastoma from TARGET dataset.

Supplementary table 11. Correlation analysis of METTL14 and YY1.

Supplementary table 12. Expression analysis of ETS1 in GES4447 and GSE49710.

Supplementary table 13. RNA-seq differential gene statistics.

Supplementary table 14. M6A peak distribution.

Supplementary table 15. RNA-seq and MeRIP-seq intersect genes.

Supplementary table 16. Data analysis of 20 genes with significant expression differences.

Supplementary table 17. Expression analysis of YWHAH in GES4447 and GSE49710.

Supplementary table 18. Correlation analysis of METTL14 and YWHAH in neuroblastoma.

Supplementary table 19. Correlation analysis of YTHDF1 and YWHAH in neuroblastoma.

Supplementary table 20. Cell gene expression data from the DEPMAP database were used to analyze the correlation between METTL14 and YWHAH.

Supplementary table 21. Correlation analysis of METTL14 and YWHAH in neuroblastoma datasets.

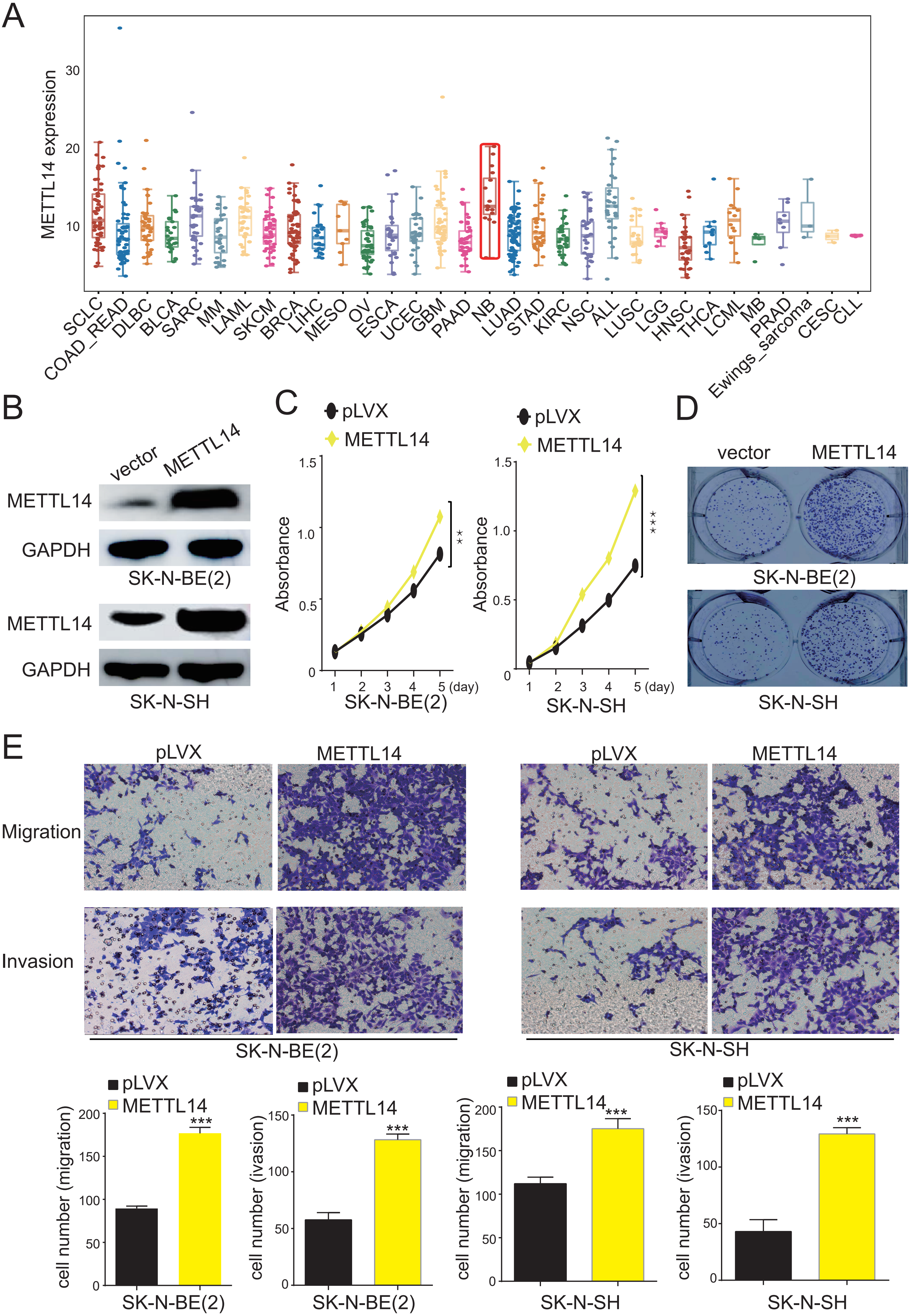

Supplementary Figure 1

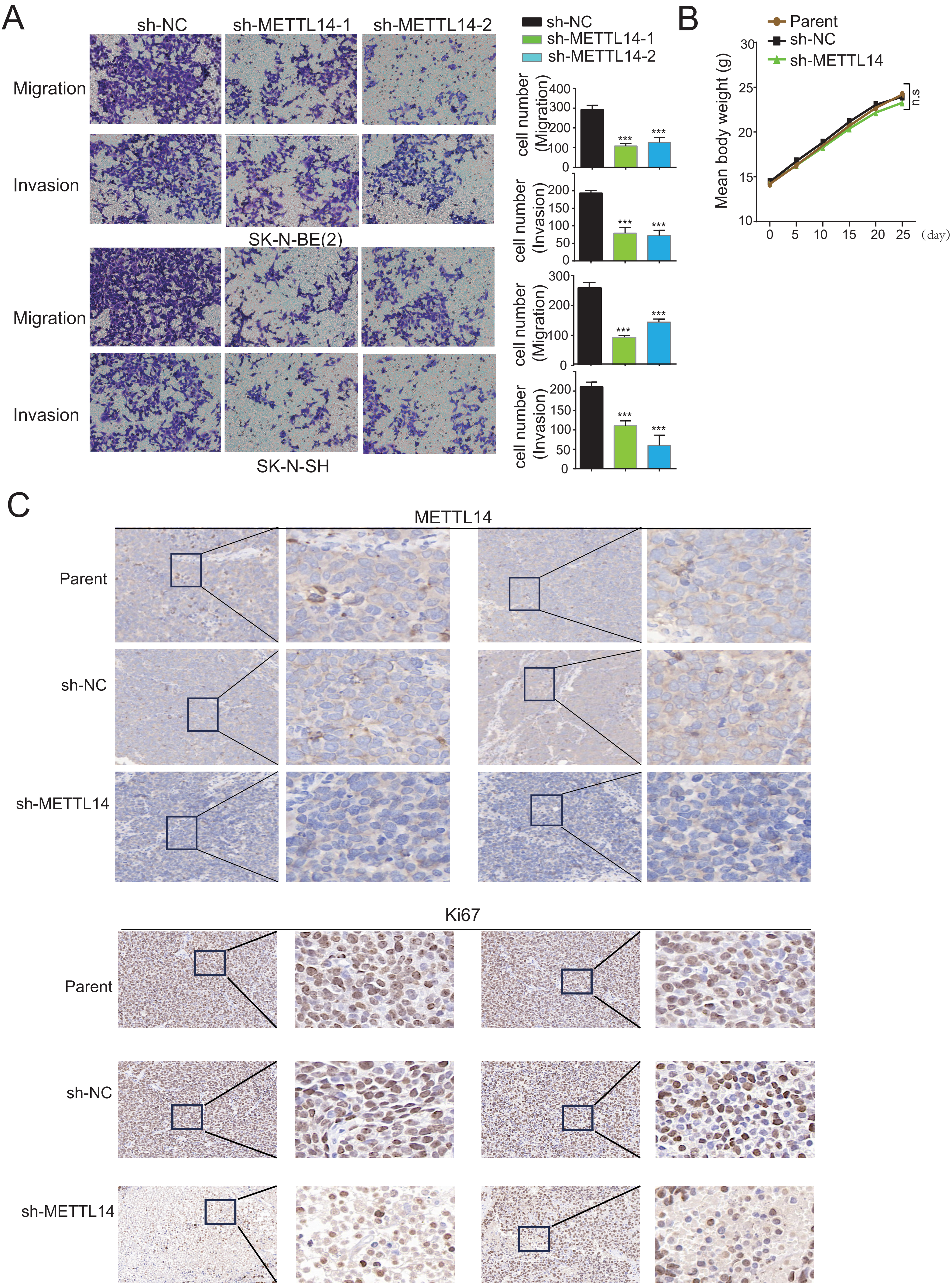

Supplementary Figure 2

A

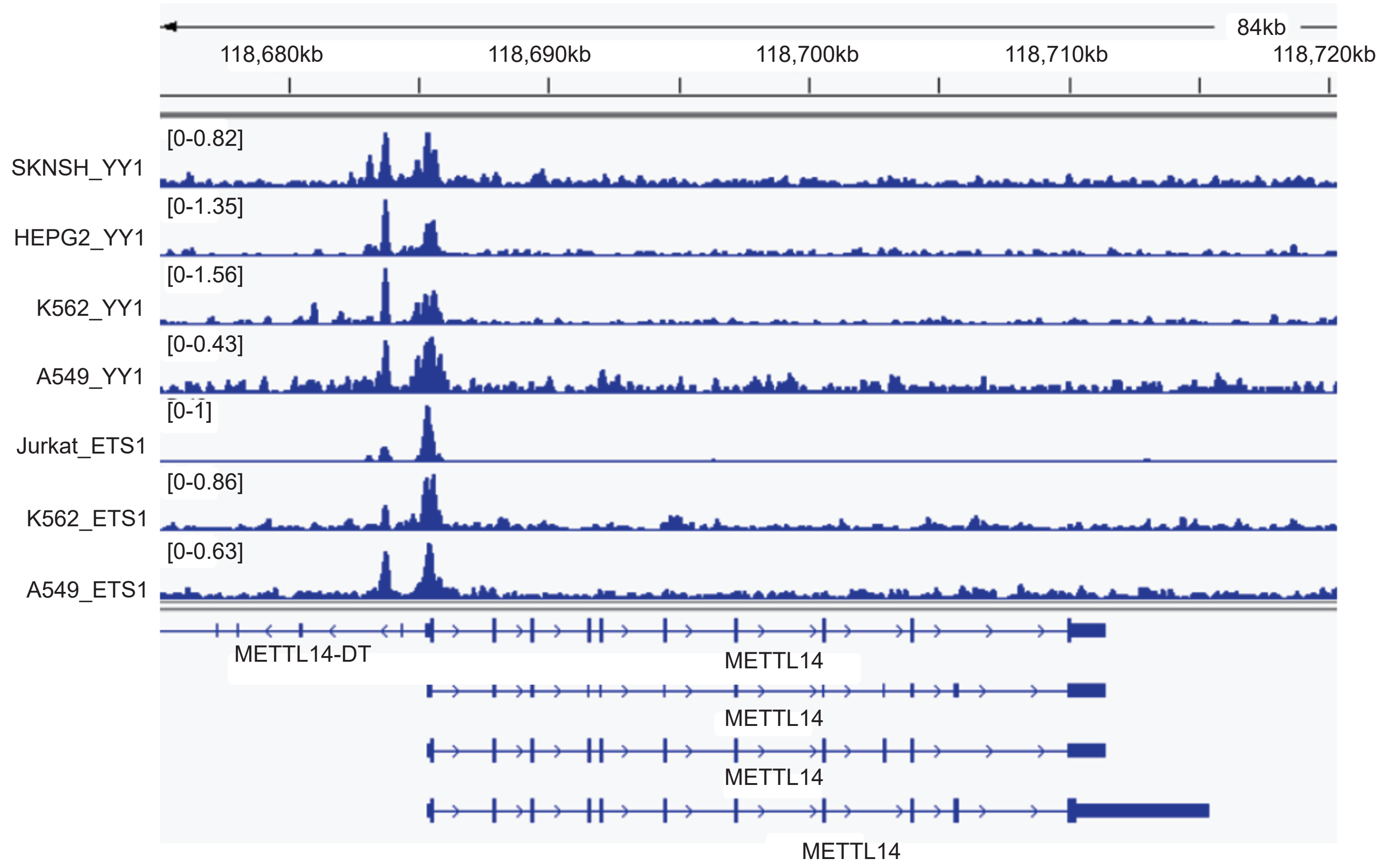

Supplementary Figure 3

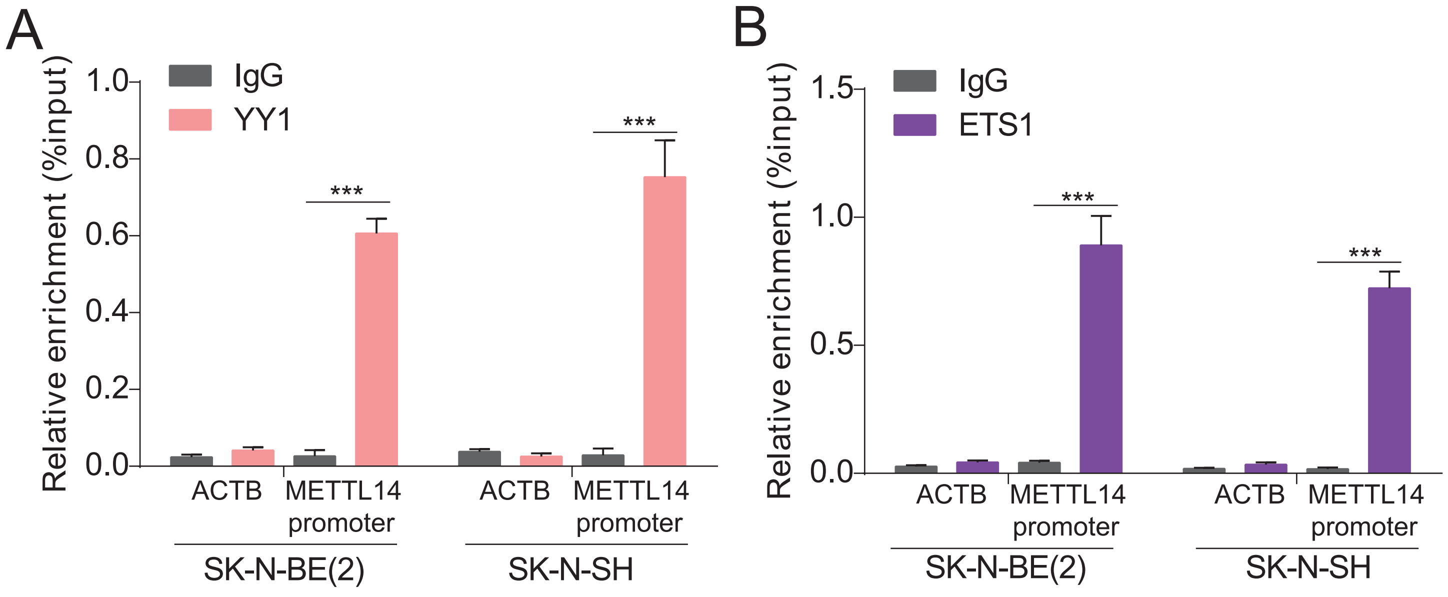

Supplementary Figure 4

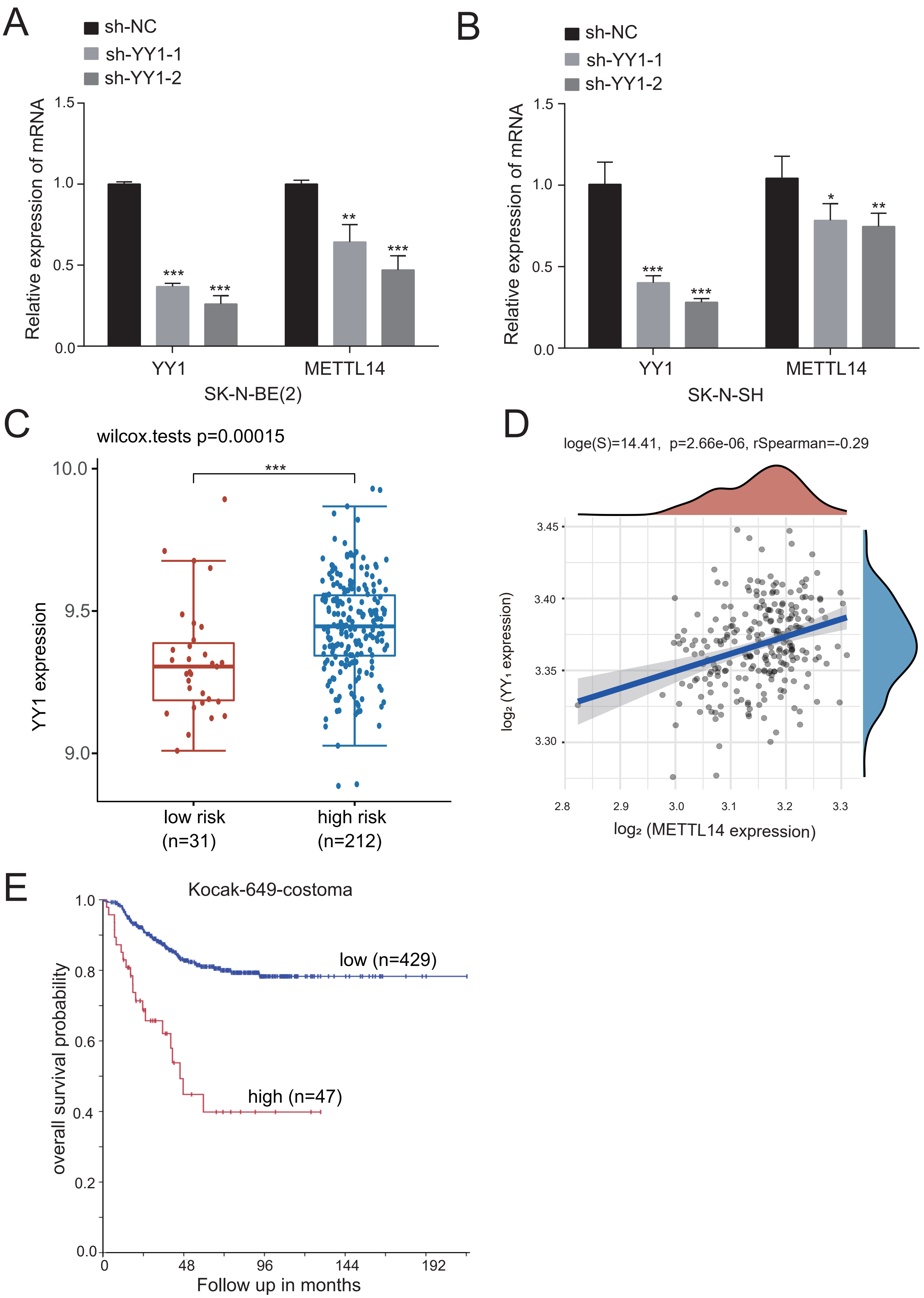

Supplementary Figure 5

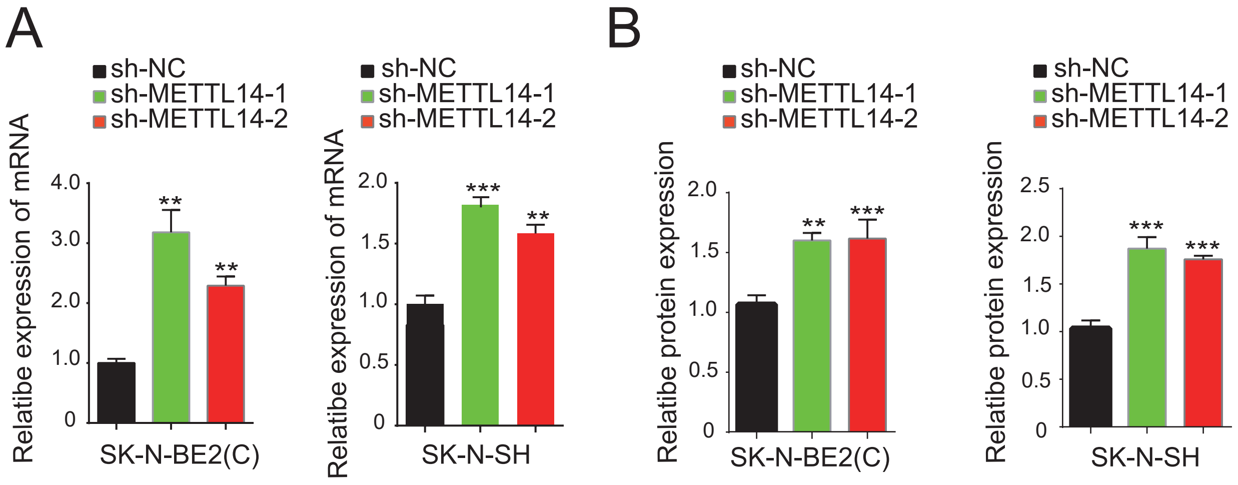

Supplementary Figure 6

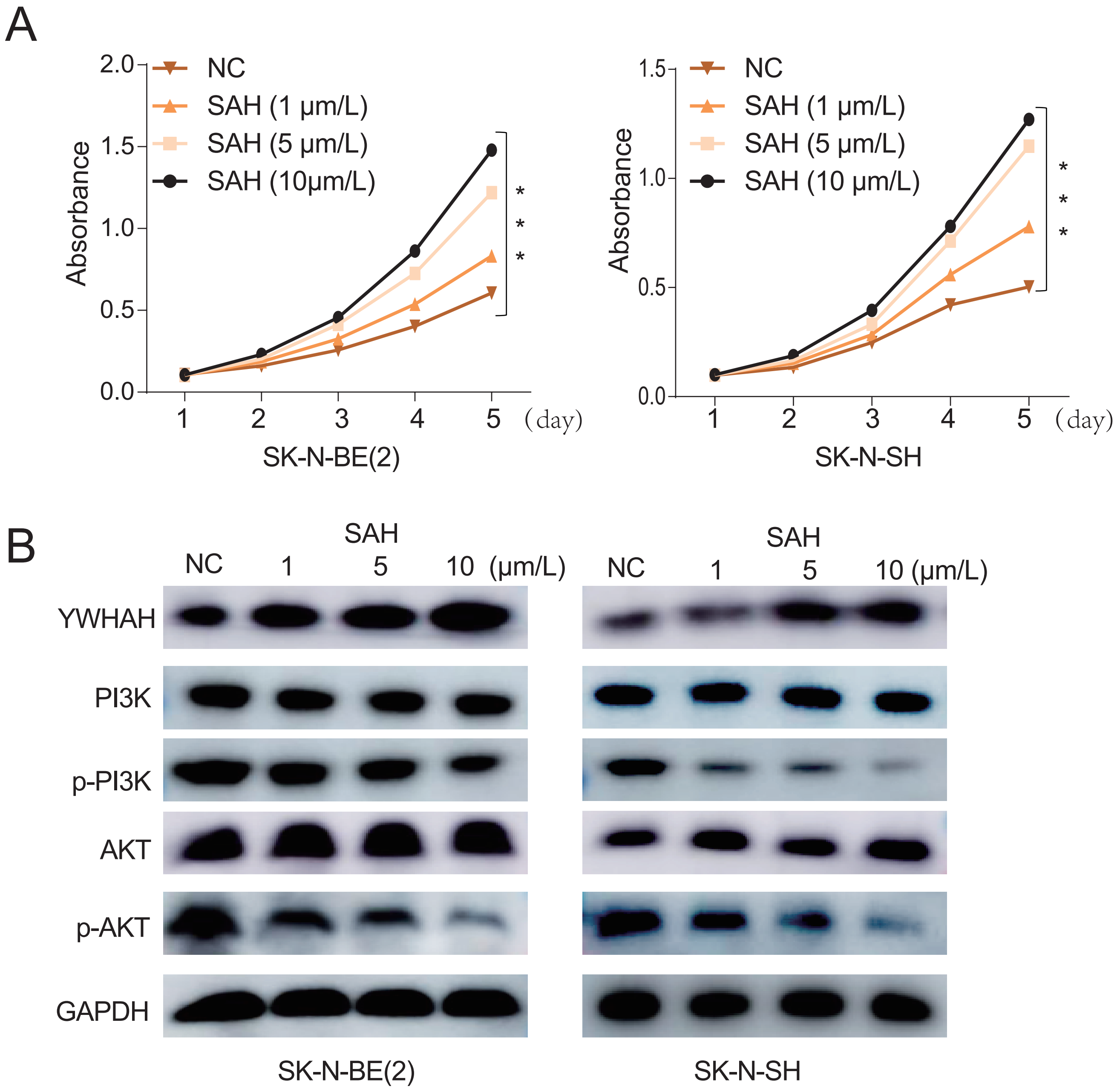

Supplementary Figure 7

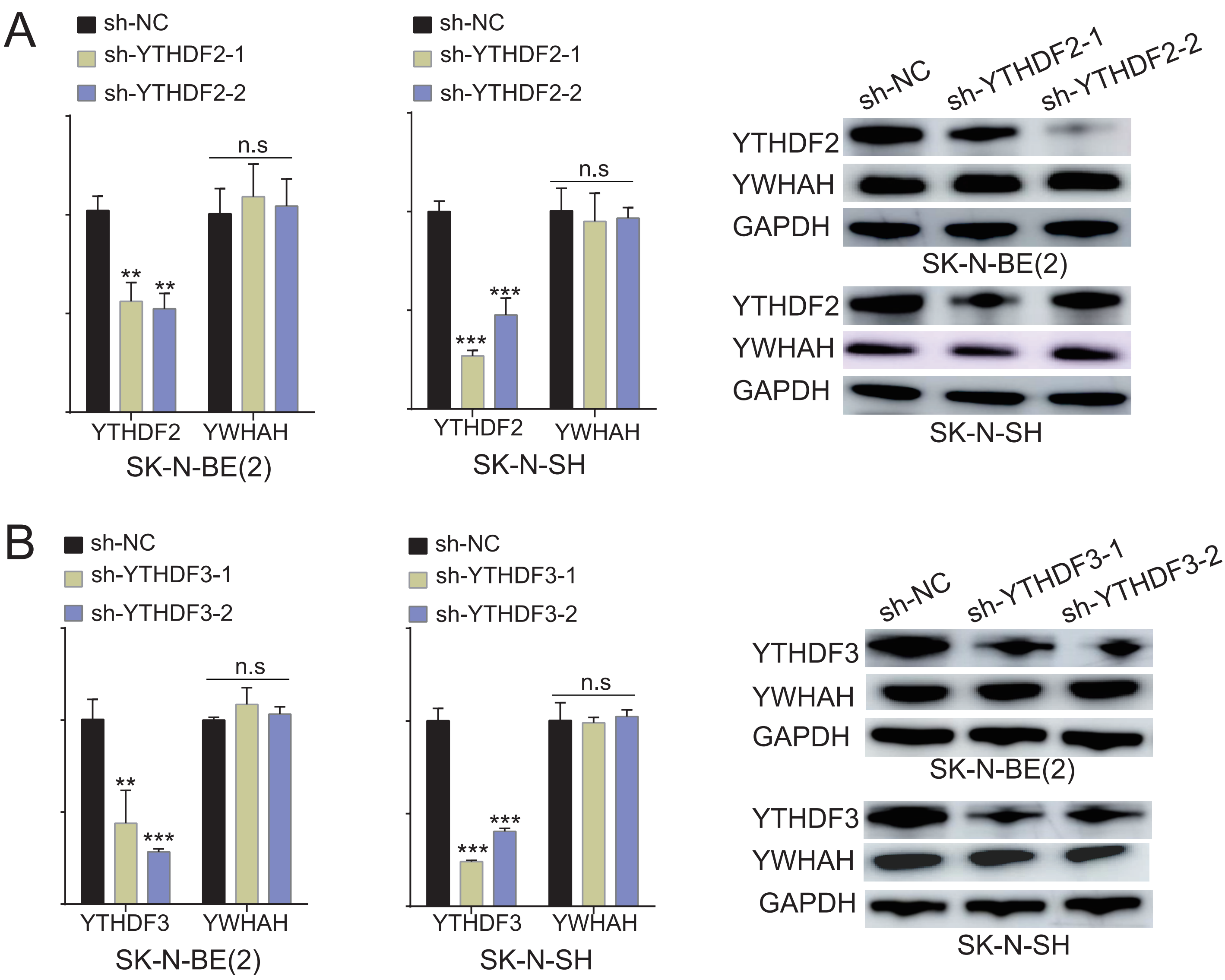

Supplementary Figure 8

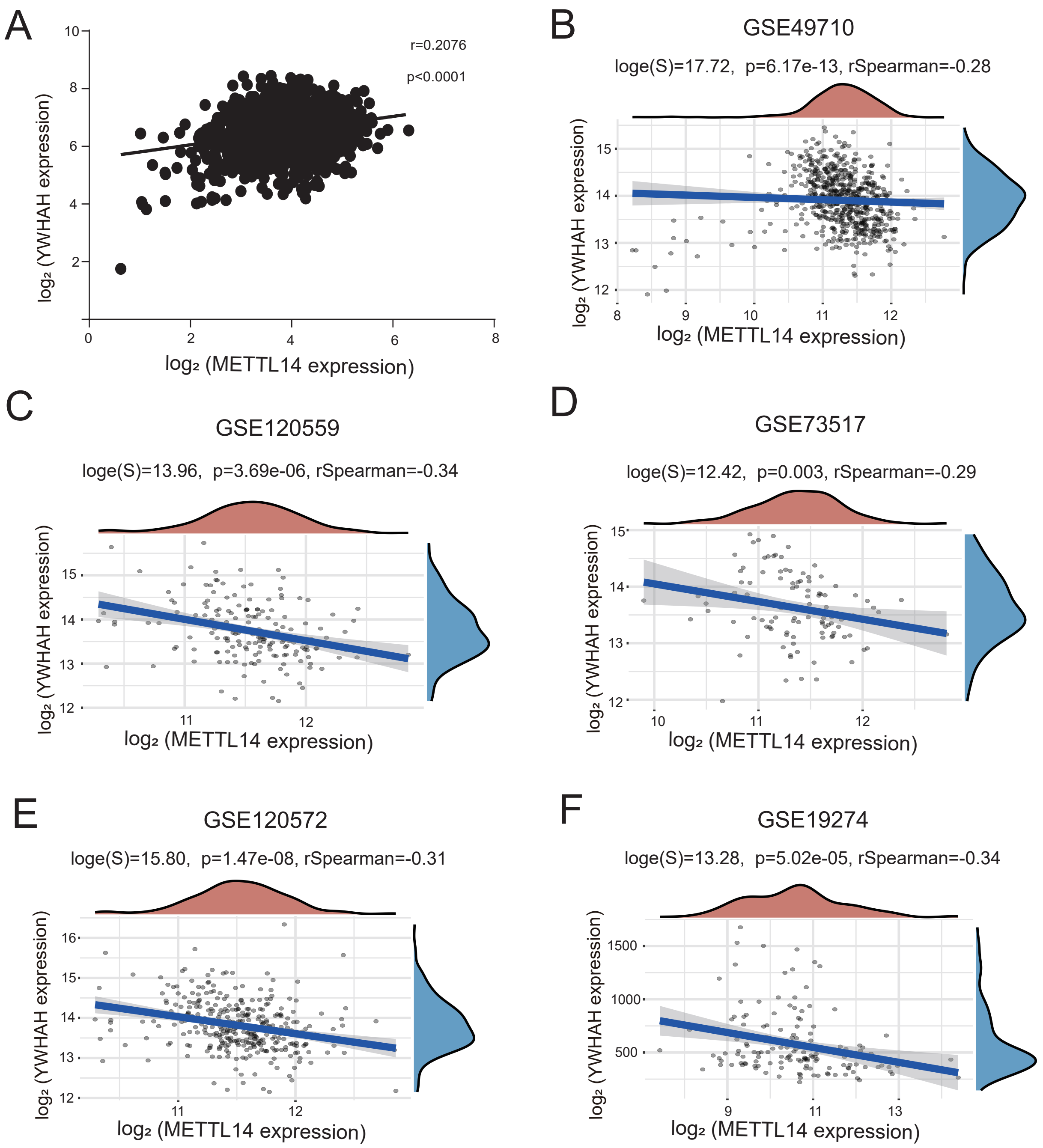

Supplementary Figure 9

Supplementary Table 1

	METTL3	METTL14	WTAP	RBM15	RBM15B	ZC3H13	YTHDC1	YTHDC2
TARGET-3	8.61836	7.3033	8.25715	8.43367	8.194	7.73483	8.49346	7.41595
TARGET-3	8.88345	7.86023	9.0252	8.65091	8.43176	8.59437	9.1565	8.0229
TARGET-3	8.47862	7.36593	7.98687	8.42556	8.30673	6.96182	8.33548	7.69198
TARGET-3	8.77171	7.79965	8.35769	8.49532	9.00335	8.30273	8.8459	7.93666
TARGET-3	8.5137	7.2822	9.01224	8.38952	8.62137	7.58598	8.62724	7.93352
TARGET-3	7.67693	7.76037	8.2273	8.19239	9.14224	7.84585	8.31775	6.8094
TARGET-3	8.84744	8.17601	8.59889	8.8444	8.5327	8.53997	8.83996	8.10543
TARGET-3	8.86674	7.88618	8.71781	8.87284	8.48482	8.19994	8.57885	7.97451
TARGET-3	8.25219	7.26121	8.32601	8.68654	8.39249	7.79123	8.44993	7.54233
TARGET-3	8.37561	7.76633	8.22298	8.57122	8.47598	7.463	8.49919	7.73676
TARGET-3	8.34988	7.94033	8.57323	8.78069	8.43037	8.40717	8.81602	7.88235
TARGET-3	8.74944	8.42465	8.87799	8.08036	8.82255	8.31911	8.93838	7.90028
TARGET-3	7.99759	7.35317	8.81383	8.24336	8.08948	7.90391	8.30037	8.24713
TARGET-3	8.53257	7.46327	9.06552	7.91369	8.66409	8.70347	8.55787	7.63183
TARGET-3	7.60707	7.77668	8.72739	7.66844	8.66908	7.79854	8.44639	7.23704
TARGET-3	7.63483	7.45216	8.03816	8.34739	8.46713	8.12157	8.3109	7.63222
TARGET-3	9.04841	7.0377	8.07786	8.19389	8.46816	8.13818	8.40564	7.55136
TARGET-3	8.46618	7.78143	8.54187	8.83685	8.48125	8.13013	8.68662	7.71233
TARGET-3	8.42043	7.14799	7.94253	8.62897	8.45415	7.49041	8.12685	6.87804
TARGET-3	8.4923	7.64017	8.47224	8.94205	8.63117	8.43436	8.5235	7.93884
TARGET-3	8.25688	7.1994	7.69589	7.76093	8.24201	7.4898	7.89508	7.08681
TARGET-3	8.71224	8.03172	9.12521	8.51722	9.16278	8.21549	8.80869	7.6971
TARGET-3	8.39142	7.96317	8.91548	8.78034	8.75734	7.81996	8.47894	8.12274
TARGET-3	8.32744	7.77198	8.4542	7.99615	8.50851	7.98342	8.6494	7.61569
TARGET-3	7.74194	8.07611	8.61819	8.54064	8.28476	7.94522	8.94494	7.82581
TARGET-3	8.8175	7.51091	8.2293	8.30526	8.37532	8.42762	8.56621	7.54075
TARGET-3	8.35129	7.74478	8.62924	8.67878	8.70906	7.83725	8.6462	7.55627
TARGET-3	8.4312	7.36118	8.71061	8.0989	8.40538	7.24851	8.21507	7.25714
TARGET-3	9.05072	8.08029	8.50401	8.26362	8.85292	7.50938	8.54278	7.99629
TARGET-3	8.69342	7.44297	8.61377	8.69908	8.76936	8.44067	8.64271	7.66866
TARGET-3	8.90034	8.16011	8.49773	9.54746	9.03328	7.78017	8.94534	8.02828
TARGET-3	8.76404	7.93695	8.5737	9.06814	8.58572	7.46999	8.9132	7.72422
TARGET-3	8.35534	7.78644	8.77666	8.82506	9.31335	8.37419	8.81789	8.42406
TARGET-3	8.5324	8.48396	9.18238	8.87471	9.11211	7.63456	9.43768	7.96662
TARGET-3	9.53233	7.85604	8.97964	8.36089	8.97635	7.53358	8.66669	7.21524
TARGET-3	9.86154	8.805	9.25311	8.87993	8.63289	8.17585	9.34679	8.12402
TARGET-3	9.03082	7.88024	7.44116	8.59864	8.01346	8.9769	8.04543	7.00507
TARGET-3	9.13425	8.30731	9.0065	8.80708	8.89998	7.93324	9.08548	7.53112
TARGET-3	8.2642	7.39381	8.89262	8.1105	9.06787	6.95288	9.05302	7.66283
TARGET-3	8.26538	7.41424	8.75608	8.37203	8.56931	7.12939	8.68925	7.02755
TARGET-3	8.51781	8.31635	9.06294	8.59919	8.45185	8.07144	9.62503	8.28809
TARGET-3	8.68795	8.05388	9.23225	8.97736	8.21983	7.59385	8.79525	8.54129
TARGET-3	8.6897	7.99366	8.37695	9.12783	8.93292	7.81222	8.77222	7.88748
TARGET-3	8.59552	7.99528	8.50787	8.31812	7.82897	7.5471	8.56386	7.99463
TARGET-3	8.90391	7.6401	8.81557	9.24636	8.38366	8.24609	8.62345	7.59899
TARGET-3	9.12179	8.02303	8.73371	8.83725	8.49455	6.98454	8.53535	7.65418
TARGET-3	8.27115	8.0571	8.7906	8.64119	8.32801	7.23681	9.11375	7.85799
TARGET-3	8.90194	8.59227	9.03604	9.05945	8.9246	7.93091	8.89868	7.74838
TARGET-3	8.60796	7.9189	8.80529	8.69575	8.81137	7.40877	8.75658	7.8888
TARGET-3	8.83416	7.61279	8.89734	8.85364	8.81014	7.35002	8.42659	8.20149
TARGET-3	9.28856	8.59269	9.29499	8.23949	8.66847	7.6257	8.70992	8.16091
TARGET-3	8.68398	8.05916	8.88512	8.4528	9.0704	7.40871	8.51367	7.78848
TARGET-3	8.32277	7.43599	8.28718	8.69312	8.19632	7.75743	8.06981	7.09586
TARGET-3	8.46616	7.42422	8.62202	8.44641	8.01004	8.28176	8.918	7.62636
TARGET-3	8.78118	8.24315	8.96549	9.27074	8.84778	6.3142	8.5923	7.58557
TARGET-3	8.71054	7.77141	8.57006	8.14652	8.46861	6.40741	7.76863	7.44313
TARGET-3	9.25932	8.14646	8.50144	8.25001	8.51363	6.76973	8.32057	7.44445
TARGET-3	8.1965	7.53679	7.97941	8.29349	9.2482	7.65998	8.51024	7.07589

TARGET-3	8.9992	8.42711	9.15495	8.86889	8.60115	8.28115	9.1942	7.94208
TARGET-3	8.75717	7.39985	8.84211	8.63242	8.68101	6.63523	8.33959	7.51156
TARGET-3	8.15144	7.43276	8.66289	8.26327	8.7214	7.61614	8.72874	7.18976
TARGET-3	8.20259	8.01931	8.74323	8.0946	9.02143	7.73127	8.77161	7.36781
TARGET-3	8.81905	8.1642	9.23315	8.59048	9.18456	7.57179	8.54845	8.58036
TARGET-3	8.58642	7.73073	8.37963	9.16998	8.76188	6.70077	8.53831	7.17286
TARGET-3	7.63498	7.56174	8.27018	9.00446	8.14466	8.02353	8.60667	7.31087
TARGET-3	8.7998	8.27069	8.54501	8.30296	8.54742	7.76604	8.89126	8.00423
TARGET-3	8.56204	7.91549	8.64491	8.11999	8.20166	7.89152	8.69456	7.80268
TARGET-3	8.58809	8.23907	8.69861	8.60615	8.82966	8.24815	8.88095	7.72616
TARGET-3	8.75159	7.52105	8.07095	8.70361	8.66557	7.39717	8.23726	6.91856
TARGET-3	9.02354	8.13648	9.33162	8.52145	9.16338	7.40573	8.99855	7.95413
TARGET-3	9.60006	8.01798	8.59563	8.65305	8.83823	7.17538	8.50722	7.96133
TARGET-3	9.3296	8.2181	8.96328	8.8103	8.61253	8.03243	8.67266	7.82253
TARGET-3	8.45516	7.66111	8.50331	9.00167	8.86553	7.87262	8.81611	7.93149
TARGET-3	8.26464	8.33747	8.34673	8.58105	8.43744	7.41657	8.47056	8.06864
TARGET-3	8.10468	8.14777	9.18312	8.73263	8.84733	8.3437	9.30496	7.72843
TARGET-3	8.70488	7.94385	8.50249	8.58877	8.59012	7.70645	8.76617	7.8986
TARGET-3	9.40152	8.05025	8.9967	8.32559	8.86573	7.88243	8.74614	7.5617
TARGET-3	8.89895	7.57717	8.64245	8.15155	8.74395	7.6973	8.35429	7.12795
TARGET-3	8.50564	8.30145	8.94467	8.99909	8.53387	7.26638	8.71192	7.85793
TARGET-3	7.62097	7.36958	7.63199	8.13029	8.28196	6.59079	8.07675	6.84689
TARGET-3	9.24973	7.98455	8.40008	9.07262	8.76324	7.45023	8.64029	7.75061
TARGET-3	8.26466	7.43371	8.31945	9.03856	8.4216	7.26587	8.39438	7.58801
TARGET-3	8.54709	8.25778	8.93621	8.84765	9.07889	7.55483	8.83558	8.10812
TARGET-3	9.15815	8.05518	8.62054	8.34551	8.73119	7.55569	8.34239	7.59153
TARGET-3	7.51432	6.97472	7.7686	8.14608	7.62556	7.84822	8.33148	7.05644
TARGET-3	8.52075	8.08507	9.02872	8.65379	9.20328	7.7327	8.83732	8.064
TARGET-3	8.64768	7.42028	8.00727	8.47853	8.07266	8.10178	8.78582	7.70594
TARGET-3	7.6438	7.41019	8.48329	8.87164	8.04817	8.47445	8.44846	7.29728
TARGET-3	9.10354	8.56412	9.16966	8.27781	8.98454	7.51936	9.01395	7.83466
TARGET-3	8.31408	8.10993	9.01744	8.28372	8.95913	6.40068	8.65481	7.98528
TARGET-3	8.16289	6.07873	7.82033	8.1438	8.4365	7.5918	7.29509	6.40642
TARGET-3	8.92651	8.19229	9.12258	8.31702	9.0613	7.80577	8.76525	8.11336
TARGET-3	8.80286	7.7986	8.37023	9.23885	9.40243	7.04633	9.12552	7.45214
TARGET-3	9.1121	8.02696	8.56148	8.41731	7.90023	7.31483	8.88995	7.87793
TARGET-3	9.03416	8.40829	9.2686	8.58989	8.6848	7.84108	9.11384	7.77454
TARGET-3	8.78112	8.5769	8.8866	9.0742	8.80177	7.71637	9.17604	7.89818
TARGET-3	8.55607	7.79179	8.2253	9.06266	8.88481	7.84189	8.8523	7.80072
TARGET-3	7.38614	7.00228	7.78412	7.53256	7.74052	6.28706	7.80527	6.25327
TARGET-3	9.61001	8.30711	8.57945	8.03423	8.64674	8.20374	8.92323	8.14814
TARGET-3	7.98842	8.04851	9.67684	8.58627	8.76167	7.41473	9.325	7.84887
TARGET-3	8.64995	8.11829	8.69258	8.32152	8.29953	8.14364	8.93275	7.85248
TARGET-3	8.62783	7.91723	9.08209	9.17134	9.14686	7.92182	8.9059	8.12903
TARGET-3	8.10082	8.38271	8.89058	8.49194	8.37605	8.07678	9.01721	8.00814
TARGET-3	9.04027	8.2504	8.68979	8.86001	8.99017	8.08062	8.99523	7.6297
TARGET-3	8.15572	7.98046	8.37153	8.37907	8.69193	7.2587	9.06366	7.30117
TARGET-3	8.78764	7.87003	9.34939	8.75855	8.38299	7.32993	8.952	7.61462
TARGET-3	8.30071	7.73226	8.4613	9.04274	8.45722	7.64013	8.68112	7.32559
TARGET-3	9.03162	8.0263	8.14247	8.53825	8.72283	7.91604	8.26851	7.61321
TARGET-3	8.16957	7.7747	9.43807	8.45851	8.39116	7.1638	8.90716	7.65318
TARGET-3	8.92982	8.35247	8.97956	8.78767	8.2901	8.25708	9.09398	7.79496
TARGET-3	9.13152	7.5172	8.96052	9.10288	8.44092	8.29901	8.60535	7.28776
TARGET-3	8.94433	8.37847	8.68975	8.96732	8.68863	7.92676	8.95351	8.22524
TARGET-3	8.32889	7.78422	9.11749	8.6707	8.63412	7.13599	9.0066	7.7034
TARGET-3	7.71588	8.55678	8.95993	9.24913	9.00865	7.28712	9.48279	8.09403
TARGET-3	7.59584	7.02268	7.9461	8.6556	8.94098	7.30396	8.14942	6.82296
TARGET-3	9.64792	7.46975	8.19044	8.69545	9.43136	7.78356	8.54798	6.89892
TARGET-3	8.53307	7.95655	8.75484	9.02246	8.66289	7.74335	9.0333	8.41303

TARGET-3	9.0848	8.15103	8.5581	8.64903	8.81558	7.44818	8.61998	7.70184
TARGET-3	8.48531	7.77435	8.15761	8.58178	8.63706	7.40319	8.31155	8.07975
TARGET-3	8.38486	6.99383	7.83975	8.15625	8.52104	7.75362	8.11553	6.4503
TARGET-3	7.63349	7.8815	8.53741	8.58832	8.23701	7.3845	8.46999	7.6736
TARGET-3	8.07643	8.82909	8.77795	9.01249	8.4307	7.01561	9.23492	8.34824
TARGET-3	8.27544	7.94388	8.91884	8.59745	8.61089	8.57465	9.31662	8.09171
TARGET-3	8.90825	8.12314	9.05341	9.0744	8.58887	7.29388	8.6353	8.04646
TARGET-3	9.55993	8.01022	7.62032	9.36875	8.56497	8.23506	8.69508	7.9779
TARGET-3	8.64066	8.44992	9.097	9.46356	8.83347	8.60089	9.17435	7.8106
TARGET-3	8.2078	7.83362	8.35824	8.18302	9.04098	8.23851	8.66142	6.92099
TARGET-3	8.37841	6.98459	7.61427	8.80484	9.01886	7.12751	7.68068	7.28268
TARGET-3	8.76118	8.84062	8.9951	8.97412	9.44138	8.20522	9.28127	7.61583
TARGET-3	8.86466	8.11066	8.6946	9.22883	8.58693	8.23652	9.0412	7.99011
TARGET-3	8.95282	8.04093	8.72937	8.0869	8.58952	7.51526	8.89284	8.47491
TARGET-3	8.13127	7.24902	7.85096	7.96079	7.98391	7.27256	8.20236	7.34856
TARGET-3	8.72649	7.85587	9.24532	8.50509	8.67043	7.91343	9.16193	7.72366
TARGET-3	9.31884	8.48692	7.90899	9.22678	9.31884	8.26885	8.97014	7.88398
TARGET-3	8.36611	8.12883	8.39438	8.28555	7.95435	8.27206	9.18315	7.92272
TARGET-3	8.54572	8.23291	8.50918	8.94283	8.42678	7.56385	8.89068	7.66669
TARGET-3	8.14589	7.6444	9.09069	8.09067	8.60193	7.55473	8.42011	7.3924
TARGET-3	8.68287	7.73449	8.0465	8.70182	8.80757	7.72287	8.38646	7.34189
TARGET-3	9.01001	8.34898	8.88079	8.88704	8.61753	8.34512	8.79311	7.39207
TARGET-3	7.78167	7.08764	8.25843	7.98451	8.81589	6.56331	8.01967	6.72295
TARGET-3	9.4776	8.49741	8.419	9.01594	9.34535	7.8276	9.02192	7.30077
TARGET-3	8.69326	8.40893	8.86237	8.35617	8.59961	8.22797	8.9474	7.70753
TARGET-3	8.54425	7.5937	7.54237	9.14056	8.59509	7.45058	8.47655	7.18859
TARGET-3	8.3032	8.25531	8.29903	7.97192	8.76826	8.18475	8.29074	7.88582
TARGET-3	9.01562	8.32992	8.81398	8.48585	8.70861	7.1959	8.83826	7.67554
TARGET-3	7.97937	7.93784	8.00118	8.65807	8.97848	7.54219	8.31178	7.37911
TARGET-3	8.44337	7.46728	7.85212	8.1029	8.34226	7.66242	8.47586	7.38853
TARGET-3	9.34651	7.85844	8.6012	8.76398	8.69976	7.8668	8.70752	7.45192
TARGET-3	8.78454	8.28727	8.51921	8.17264	8.14382	7.97597	8.85532	8.08417
TARGET-3	8.89456	7.81883	8.42586	8.91649	8.97937	7.81121	8.68548	7.41129
TARGET-3	8.78406	8.06527	8.18701	8.17829	8.3754	7.67756	8.88016	7.54195
TARGET-3	8.72741	8.12781	8.45414	8.90453	9.23612	7.97727	8.73769	8.04028
TARGET-3	8.22388	7.94284	8.57294	9.28986	8.85926	7.02972	8.66748	7.51707
TARGET-3	8.93653	8.00981	8.21511	8.7902	8.84468	7.79963	8.62163	7.53015
TARGET-3	8.55119	7.63915	7.84086	9.04564	8.91013	6.8158	8.26812	7.39833
TARGET-3	9.08439	8.19444	8.83274	8.99053	8.90198	8.22673	8.83273	8.17945
TARGET-3	8.82081	8.35266	8.04627	8.75957	8.59539	7.6277	8.81172	7.99164
TARGET-3	7.62308	8.25538	8.25169	9.62235	8.94391	7.12921	9.20185	8.06303
TARGET-3	8.36542	8.28049	7.83148	8.31682	8.51238	7.50467	8.16919	7.87293
TARGET-3	7.73824	7.34179	7.83678	8.46165	8.50302	7.28109	8.17507	7.36543
TARGET-3	7.1313	8.34572	9.13991	9.27065	9.45977	6.20044	9.04113	7.22801
TARGET-3	8.26993	8.23015	8.78773	9.37407	9.31067	7.79738	8.63417	7.05622
TARGET-3	8.91718	8.25893	8.68223	9.15765	9.34792	8.3514	9.10322	8.11659
TARGET-3	8.80237	7.71163	7.95303	8.93297	8.77013	7.42989	8.80004	7.92278
TARGET-3	9.10872	7.47074	8.0022	8.82455	8.62866	8.06746	8.5045	7.81018
TARGET-3	9.32149	7.3255	7.55962	8.23636	8.30154	8.29604	8.40994	7.3092
TARGET-3	8.95682	7.8985	8.43561	9.08628	8.65403	8.67077	9.10105	7.86911
TARGET-3	9.24024	7.3531	8.349	8.6661	8.12497	7.18747	8.42896	7.61109
TARGET-3	8.05494	7.4123	7.8906	8.34284	8.61899	7.1805	8.32288	7.23802
TARGET-3	8.08442	7.29317	8.22018	8.09978	8.11947	6.64509	8.42422	7.43412
TARGET-3	8.76115	7.50362	8.47275	8.76263	9.09964	8.01451	8.6181	7.66273
TARGET-3	8.71937	8.40479	9.0753	9.17802	8.44981	7.81867	9.0723	7.94311
TARGET-3	8.49699	7.52104	7.73879	8.87762	8.39216	7.7501	8.5073	8.02378
TARGET-3	8.23405	7.50386	8.6497	7.8822	8.28271	7.11977	8.49158	7.56328
TARGET-3	9.92414	8.869	8.66839	8.56616	8.77658	7.96477	9.03028	8.31902
TARGET-3	8.96185	7.89437	8.91321	8.66517	8.79378	7.42899	8.82545	7.99222

TARGET-3	8.29295	7.26709	8.02633	8.32533	8.3327	7.16607	8.50508	6.85081
TARGET-3	8.26686	8.16592	8.33355	8.937	8.42182	7.90084	9.09312	7.95169
TARGET-3	8.75885	7.76126	7.97233	8.56866	8.65093	7.17046	8.52117	7.62813
TARGET-3	8.53871	8.25054	8.32212	8.68939	8.33315	7.52035	8.67414	7.44841
TARGET-3	8.56061	7.76178	7.9695	7.92558	8.68588	7.2956	8.5188	7.6051
TARGET-3	8.17268	8.16947	8.47168	8.37842	8.30566	7.75528	9.03467	7.93574
TARGET-3	8.13922	8.55402	8.70314	8.35928	9.44397	7.70603	8.67235	8.35967
TARGET-3	8.12876	8.07696	10.1463	8.85919	8.90014	7.63034	9.06015	7.90618
TARGET-3	8.22177	7.5726	8.12973	8.37949	8.39544	6.58422	8.55349	7.32405
TARGET-3	9.47477	7.91519	8.96014	8.49624	8.58276	8.0073	8.58039	7.61175
TARGET-3	8.64912	7.88826	8.72752	8.42589	8.42561	7.27729	8.64302	7.46007
TARGET-3	8.74372	8.09805	8.98065	8.49266	8.73064	7.73648	8.81421	8.19157
TARGET-3	7.59567	7.10663	8.60502	8.52976	8.60303	6.32793	7.99426	6.76499
TARGET-3	9.21674	8.25877	8.70888	8.69308	8.35369	7.51057	9.01995	8.05265
TARGET-3	9.81257	8.60989	9.15138	8.89806	8.80488	7.84009	8.78858	7.3678
TARGET-3	8.49206	8.25564	8.33011	9.01027	8.79332	7.09956	8.77721	7.91229
TARGET-3	8.74071	8.01204	7.95398	8.97873	8.8046	7.12816	8.57473	7.82042
TARGET-3	8.29598	7.39434	8.39582	8.27117	8.3516	6.81588	8.09062	7.10713
TARGET-3	8.12288	7.78095	8.64889	8.02848	8.31153	7.41752	8.38855	7.64866
TARGET-3	8.89837	8.21045	8.88484	8.58498	8.8055	8.1165	8.50285	8.1549
TARGET-3	8.64585	8.13282	9.06312	7.84997	8.20694	7.42058	8.73499	8.00899
TARGET-3	9.09934	8.11901	9.10282	8.27725	8.99631	7.46284	8.61325	8.12029
TARGET-3	9.09699	8.10323	9.12359	8.54568	8.79562	7.13849	8.60285	7.55268
TARGET-3	8.83954	8.65283	9.33269	9.10939	9.16027	7.1668	8.96683	7.96329
TARGET-3	8.41347	7.19466	8.54828	8.41641	8.76013	7.58042	8.20035	6.96599
TARGET-3	8.34653	7.61585	9.14059	8.74688	8.4335	7.08739	8.5128	7.97664
TARGET-3	9.16199	8.35549	9.40578	8.86509	9.18712	7.44202	8.8293	8.27444
TARGET-3	8.42053	8.26073	8.76569	8.82639	8.28611	7.2579	8.86916	7.8231
TARGET-3	8.82047	8.53678	8.67971	9.23705	8.65181	7.60225	9.05681	7.89518
TARGET-3	8.68545	8.91843	8.82527	8.2069	8.4213	7.69076	9.18862	7.9955
TARGET-3	8.45918	8.16116	8.09141	9.20094	9.13946	7.20616	8.60481	7.9545
TARGET-3	9.23026	8.25725	9.37055	8.54864	8.52477	7.62223	8.963	8.14083
TARGET-3	8.76065	8.77459	8.94177	8.76722	8.89081	7.60788	8.94845	8.2116
TARGET-3	8.48589	7.2176	8.16565	8.18138	8.76174	7.33074	8.06567	7.22514
TARGET-3	8.67891	8.44909	8.84965	8.49163	8.10412	7.55995	8.9729	8.08272
TARGET-3	9.1174	8.13081	9.23497	8.63838	8.71778	8.21734	8.69764	8.05566
TARGET-3	9.1421	8.18962	9.30148	8.72478	8.27868	8.04524	9.10374	8.20516
TARGET-3	7.93817	7.68844	8.79917	8.55231	8.23326	6.83352	8.63896	7.31564
TARGET-3	8.52719	7.3619	8.92117	8.07762	8.52749	7.25971	8.42017	7.51407
TARGET-3	8.05501	6.87112	8.31605	7.93107	8.28369	6.86384	7.77478	7.18982
TARGET-3	8.41834	7.32842	8.90216	8.10294	8.53297	7.58906	8.09635	7.61393
TARGET-3	8.87381	8.15119	8.6495	9.13415	8.61683	7.2815	8.93936	8.05702
TARGET-3	8.50222	7.96473	9.15023	8.41557	8.79441	7.57985	8.80342	7.87696
TARGET-3	8.53563	7.96038	9.01087	8.81046	9.15688	7.67162	8.88522	8.20609
TARGET-3	8.25397	7.19984	8.55014	7.51015	8.39147	7.28065	7.62537	6.99567
TARGET-3	8.23827	7.07745	8.43753	8.45569	9.12005	6.5057	8.02874	6.93729
TARGET-3	8.34327	7.94934	9.59338	8.87899	8.31186	6.96226	9.0914	7.86505
TARGET-3	9.08932	8.35173	9.23114	9.43111	9.10899	7.39386	8.65857	8.0408
TARGET-3	9.00925	8.24429	9.14062	7.96688	8.2505	7.38733	8.76361	8.15536
TARGET-3	9.4068	7.53384	8.66251	8.34782	8.45162	7.93521	8.25191	7.74966
TARGET-3	8.23248	7.60035	8.996	8.32163	8.08498	7.09814	8.36973	7.28749
TARGET-3	8.59551	7.17324	8.75414	8.56992	8.61503	7.58929	8.33777	7.40162
TARGET-3	8.8291	8.1187	9.13146	8.73966	9.17993	7.06814	8.75022	8.32254
TARGET-3	8.20776	7.06729	8.80522	8.52175	8.33729	6.67018	7.773	7.70267
TARGET-3	8.39853	7.77643	8.77671	8.60507	8.43732	7.15158	8.89213	7.66624
TARGET-3	8.4527	7.03863	8.30501	7.96511	8.55475	7.55462	8.26184	7.07746
TARGET-3	8.86057	7.51336	8.7977	8.68015	8.78471	7.55313	8.49437	7.63523
TARGET-3	8.95094	7.87299	8.92798	9.03491	8.95021	7.42736	8.86702	8.26258
TARGET-3	8.23763	7.22368	8.59335	8.60186	8.47693	7.62075	8.0284	7.27933

TARGET-3	8.05401	7.40854	8.3356	8.86844	8.29901	7.36921	8.5582	7.44813
TARGET-3	8.80799	7.93709	9.01478	8.58564	8.74696	6.91585	8.64474	7.76466
TARGET-3	9.00406	8.0821	9.12867	8.7272	8.70278	7.51394	8.78917	7.95332
TARGET-3	8.9276	7.9925	9.25821	8.73749	8.48019	7.43745	8.76099	7.98308
TARGET-3	8.54711	8.6694	8.91834	9.04078	8.65759	7.79621	9.22909	7.5531
TARGET-3	8.8207	8.43161	8.64077	8.71027	8.83039	7.18378	9.02925	7.99247
TARGET-3	8.98876	8.31327	8.94207	8.84022	8.39101	8.02774	9.08086	8.03836
TARGET-3	9.50899	8.38383	8.74972	9.23225	8.84442	8.15714	9.20608	8.17612
TARGET-3	9.1044	8.41612	9.13127	9.35579	9.06849	8.36026	9.08152	8.33056
pvalue	0.06886	0.000769	0.069695	0.01704	0.154324	2.64E-05	0.022956	0.795837

YTHDF3	YTHDF1	YTHDF2	HNRNPC	IGF2BP1	IGF2BP2	IGF2BP3	RBMX	HNRNPA2
8.16489	7.83406	9.24124	9.35319	7.5522	4.66696	7.42599	8.81236	10.4526
8.62822	8.48957	9.15395	10.2459	8.54526	6.77039	8.26407	9.11854	10.9252
7.85181	8.0282	8.82415	9.69891	6.52889	4.87276	7.99449	8.71099	10.4249
8.24462	8.6327	9.02999	9.9023	9.07122	5.12697	8.11084	9.03463	10.7612
8.25809	8.59719	8.83554	9.55507	7.94158	6.09455	8.40799	9.40132	10.7858
8.73523	9.1482	9.96232	9.84347	8.07245	5.60306	8.35101	9.48468	9.84786
8.32529	8.58824	9.08673	9.69874	5.83168	4.51136	8.01935	8.92188	10.8533
8.49868	8.09054	9.52235	9.95659	7.11826	5.53627	8.06815	8.54371	10.625
8.00251	8.36834	8.68968	9.62619	7.7951	5.05961	7.98986	8.79347	10.3804
8.40807	8.20224	9.12683	9.8791	7.98693	5.96591	8.86119	8.94551	10.7154
8.70089	8.36883	9.22784	9.83689	7.95358	6.16678	8.77524	9.02206	10.5924
8.71445	8.79797	9.43812	10.2293	7.68168	6.52858	8.17965	9.36284	10.7258
8.02941	8.42501	8.9059	9.28922	6.96179	5.36639	8.13774	9.08262	10.6141
8.25721	8.67652	9.00507	9.87504	5.9011	6.14306	7.62104	9.11938	10.6719
8.34991	8.51267	9.45484	9.46755	7.38323	6.33963	8.00901	8.91005	9.86852
8.049	8.54313	8.64285	9.08937	7.58666	4.46411	7.33547	8.48346	10.2975
8.3428	8.33038	8.67666	9.30334	7.43435	6.54931	7.47859	8.97034	10.2758
8.31972	8.54643	9.07827	9.54317	8.65647	5.90248	8.53834	8.8078	10.3898
7.99794	8.05888	8.86607	9.32282	8.388	5.23736	7.80266	8.7217	10.4424
8.17906	8.76301	9.50912	9.58274	8.62788	5.61784	8.59091	8.95598	10.6561
7.67625	8.15582	8.86644	8.87615	5.97527	5.63008	7.25224	8.71823	10.0468
8.46259	9.18822	9.52957	9.96695	9.19829	4.9037	9.26037	9.76583	10.4977
8.7098	8.83582	9.08725	9.92787	8.55228	5.38937	8.8936	9.46655	10.7104
8.51323	8.61965	8.63723	9.32705	8.42603	5.81097	8.75627	8.55037	10.6632
8.66783	8.40305	9.2505	9.6874	8.26217	6.3925	8.95635	8.68983	10.8254
8.35928	8.5365	9.02067	9.70972	8.2181	5.78573	7.82017	9.29453	10.3343
8.07489	8.2092	9.07975	9.39017	8.56135	6.53707	8.48146	9.07697	10.4049
7.86752	8.40673	8.80021	9.16809	7.57303	4.86331	7.93243	8.72016	10.4899
8.99222	8.75477	9.24089	9.92361	6.99118	8.16987	8.0469	9.11764	10.8178
8.20971	8.54155	9.37624	9.71058	7.58225	5.11853	7.71404	9.3677	10.6686
8.22491	8.82525	8.78572	10.1422	9.52266	5.27194	9.02199	10.1526	10.8041
8.15956	8.77635	9.41574	9.83043	5.52877	5.34678	8.37472	9.07414	11.1997
8.50131	9.31564	9.51312	9.98402	5.95958	6.49864	8.1814	9.52646	10.2305
8.69986	9.08425	9.33902	10.3409	9.40404	6.04841	9.05	9.53106	11.1121
8.44035	8.73416	9.26776	10.3458	8.68185	5.28358	7.54662	9.63631	10.3743
9.16303	9.03967	9.64619	10.4522	8.86046	8.03325	8.34366	9.32584	10.8448
5.90103	7.57496	8.46622	8.23561	8.65489	5.79732	8.92545	8.92645	10.7838
8.51028	8.40183	9.55058	10.0928	9.37592	5.39035	8.65283	9.35885	11.0269
8.07451	8.47416	9.47887	10.0585	5.67746	5.28563	8.68934	9.38678	10.5666
7.965	8.88296	8.90973	9.29801	7.75075	5.08927	7.72002	9.39121	10.446
7.87615	7.82144	9.43122	10.145	8.13616	6.1034	8.3064	9.14288	11.2315
8.55603	8.6477	9.52604	10.1097	8.32418	4.97788	8.56828	8.90503	10.9721
8.4498	8.89061	9.43085	10.7117	10.0252	5.08117	8.59535	10.0748	10.8614
8.4379	8.25844	9.08985	9.60849	6.54611	5.25835	5.99215	8.60664	10.5444
8.01777	8.43913	8.87287	9.65511	8.45623	5.58142	8.15028	9.45577	10.702
8.29933	8.83512	9.16345	10.0959	9.24427	4.7907	8.37686	9.42014	10.5966
8.53491	8.5435	9.46932	9.57839	8.36073	5.57672	8.06437	9.41032	10.7176
8.61879	9.41606	9.90734	10.3226	8.99378	6.69672	8.77538	9.61727	10.7398
8.42077	8.20206	9.33278	10.0385	9.01625	5.67343	7.43734	9.08801	10.8527
8.60455	9.22574	9.2086	9.90084	8.48505	5.44364	7.97564	9.61733	10.7779
8.54627	8.36584	9.15884	10.4621	5.6441	5.3128	7.80226	9.45101	10.9093
8.55199	9.43799	9.44624	10.3019	9.23348	5.67693	8.67061	9.9268	10.7048
7.88634	8.32655	8.28632	9.06495	7.73493	5.11454	7.10665	8.57165	10.5468
8.14611	8.5783	9.33115	9.77204	6.73439	5.29666	7.32223	8.76293	10.6017
8.35975	8.66508	9.44893	10.4518	5.76289	4.90892	8.68825	9.98704	10.6167
8.3526	8.44803	8.75609	9.91385	6.58767	5.69115	7.99456	9.55648	10.3538
8.33563	8.23519	9.19556	10.0236	7.74396	6.24277	7.66929	9.16702	10.3068
7.83882	8.56877	8.73672	9.79218	9.07997	6.4635	8.49719	9.6429	10.5399

8.75251	9.05717	10.0322	10.3414	8.95858	5.7463	8.70343	9.29474	10.6987
8.50861	8.60489	9.22528	10.1129	8.51726	5.30129	7.45237	9.08725	11.03
7.798	8.65905	9.08032	9.74441	8.88825	5.02672	7.40874	9.24142	10.3835
8.769	8.84692	9.05628	10.0375	9.00154	6.24754	8.3084	9.76574	10.6395
8.59213	8.87159	9.27415	10.5626	9.28361	6.28672	9.32017	10.3101	10.9535
8.3648	8.69464	8.75173	9.68789	9.29501	6.25765	7.69579	9.15432	10.8522
7.99255	8.35794	9.03163	9.58518	7.35271	5.58949	8.1159	9.41161	10.5193
8.09584	8.43035	9.09277	10.2047	8.50408	5.07243	8.26346	9.56226	10.7749
8.67666	8.19299	9.30358	9.59494	6.34933	5.76045	6.01145	8.70501	10.189
8.85863	8.97453	9.51783	10.27	9.51936	6.41534	9.76005	9.82515	11.1046
7.75861	8.0183	8.97643	9.43071	9.00158	5.38177	8.00852	8.99682	10.3242
8.94428	9.08667	9.37689	10.3201	8.6633	5.13311	8.74289	10.2143	10.8681
8.52342	8.6382	9.3264	9.77023	7.11637	4.5889	8.17466	9.12903	10.7918
8.95344	8.56951	9.67741	10.2382	8.94032	4.9901	8.29596	9.43522	10.8479
8.23837	8.33329	9.25411	9.71	9.07714	5.39058	7.81195	9.30493	10.5295
8.30491	8.55983	9.05573	9.87756	9.05072	5.71289	8.57405	9.65524	10.7478
8.55734	8.25735	9.80171	10.2099	8.28509	5.57739	8.86517	9.2459	10.7118
7.76978	8.05292	8.8415	9.63129	8.20336	4.97107	8.50874	9.23718	10.5625
8.88323	8.76289	9.46161	10.3138	7.82452	6.4891	8.33057	9.20448	10.7434
8.25039	8.76663	9.47643	9.76942	8.69097	5.15855	8.11471	9.62542	10.2905
8.05852	8.84898	9.61659	10.1732	9.06197	5.23305	8.87335	9.19118	11.1441
7.22689	7.91567	7.37553	8.98387	8.40793	5.19818	7.66589	9.41499	10.122
8.2461	8.78649	9.4075	10.4434	9.10927	5.32874	8.45214	9.49436	10.8913
8.06148	8.66223	9.31715	9.54049	8.96987	5.2145	7.89975	9.17984	10.5187
8.65934	9.06785	9.31234	10.3575	8.46769	5.00132	9.24137	9.25	11.2202
8.67822	8.54976	9.09509	10.0117	6.94783	6.21411	7.90037	9.26174	10.6377
7.72324	8.05444	8.24944	9.8191	6.02203	5.20231	7.44644	8.64695	9.72733
8.32437	8.84095	9.35057	10.2806	9.10999	6.46943	8.98918	9.74136	10.6118
7.77685	8.00467	8.83776	9.21504	7.21342	5.90465	7.70986	8.56531	10.4831
7.92675	8.0092	8.32199	8.76514	5.68305	5.16784	7.16111	8.70363	10.0862
9.04182	8.98423	9.61244	10.3577	5.60339	5.77805	8.67442	9.64093	10.269
8.61906	8.90029	9.04627	9.99453	8.9441	4.91911	8.72279	10.0544	10.7647
7.40657	8.35668	8.91415	8.39584	8.59562	5.05321	7.65594	8.40985	9.5095
8.9804	8.99403	9.6262	10.3479	7.94049	5.21138	8.40725	9.45508	10.9651
8.61249	8.65834	9.78905	10.1665	5.68616	5.0721	8.83013	10.1726	11.0233
8.24407	8.62552	9.3184	9.73442	7.89209	5.50718	6.68515	9.14604	10.3695
8.60017	9.05262	9.70854	9.95621	5.67731	4.8328	8.75679	10.0387	10.6556
9.12308	8.05212	9.41719	10.4846	9.55888	4.92976	7.23912	9.04934	11.0021
8.06258	8.43851	9.29292	9.80247	8.32418	5.02142	7.80757	9.24935	10.716
7.38335	7.7892	7.93971	8.42499	7.91844	5.31507	6.64473	9.02972	9.92316
8.56885	8.61214	9.64639	10.3123	5.56568	4.4808	7.44653	9.21077	10.6718
8.61107	8.87878	9.84519	10.2336	7.52199	5.72016	7.47416	9.2308	10.7086
8.28032	8.47563	9.24939	9.98074	9.02356	5.31068	6.87557	9.24833	10.8277
8.29663	8.72773	9.34311	9.6236	8.3796	5.26278	8.35521	9.34579	10.5456
8.51979	8.52517	9.6687	9.50274	7.74792	7.08059	7.73587	8.90475	10.5631
8.13315	8.14972	9.71406	10.5875	8.43292	4.94282	8.80426	10.3869	10.7089
7.87597	8.33744	9.35081	9.93787	8.44644	4.72052	8.00275	8.98118	10.5157
8.23587	8.59851	9.63986	10.2003	8.38836	6.88541	8.93174	9.66325	10.8691
7.65271	8.6684	9.25064	9.72787	9.49667	5.15095	8.74201	9.58311	10.6385
7.87088	8.76567	9.41652	9.48678	9.00234	5.30708	8.83711	9.30324	10.5071
8.83031	8.48696	9.83282	9.95716	6.06542	7.04476	7.15579	8.88371	10.7423
8.23043	8.49949	9.35545	9.91501	9.52172	5.05547	8.38126	9.43612	10.864
8.24276	8.59242	9.80376	10.0711	8.60395	5.36862	9.19818	9.75577	11.0137
8.24194	8.42283	9.06314	10.4828	8.98607	4.95778	8.79699	9.85284	10.7352
8.12447	8.70239	9.54977	10.0589	8.71767	5.31996	7.46953	9.07942	10.5483
8.43724	8.59957	9.54488	10.2293	8.80577	4.98689	8.36492	9.68878	10.6989
7.59264	8.55719	8.9875	9.22872	8.86656	5.8035	7.95225	9.17072	10.4196
8.46837	8.4788	8.89917	10.1925	9.02391	9.18797	9.18815	9.63785	10.7782
8.04527	8.90378	9.02316	9.94374	7.90493	5.49364	9.07691	9.11936	11.0839

8.2098	8.36228	8.9268	10.3247	8.35011	6.21706	7.76098	9.21058	10.5608
8.20965	8.59936	8.47939	9.61151	8.99459	5.33933	8.40932	9.68606	10.7765
7.11766	7.80479	8.18787	9.32899	8.3299	7.2869	8.35987	9.35614	10.158
8.43568	8.65262	9.04657	9.33912	8.90617	5.92559	8.50312	9.31728	10.7528
8.51232	8.44117	8.69199	10.2087	7.83542	4.56936	8.63088	9.2972	10.6985
8.38961	8.4679	9.52951	10.4173	7.50007	4.7323	8.36254	8.81221	10.754
7.99798	8.93442	9.25266	10.2288	9.1444	4.83019	8.14573	9.02338	10.9228
8.17549	8.75563	9.18346	10.1203	8.96785	5.25805	8.20078	9.30284	10.9126
8.39168	8.34768	9.39152	9.98302	8.73368	7.05884	8.5856	9.21085	11.1309
8.39223	8.92563	9.70085	10.1506	9.38422	5.06139	7.96962	9.65377	10.1812
7.54882	8.19577	8.82513	9.30209	8.91669	5.18492	8.42252	9.58877	10.1756
8.69163	8.90842	9.73768	10.3896	8.66898	5.38845	9.93944	10.0178	10.7108
8.13427	8.54336	9.25869	10.1511	8.29338	5.12983	9.09512	9.00623	10.9151
8.35464	8.70239	9.20599	10.0111	9.19934	5.11386	8.86188	9.8197	10.957
7.56631	7.99486	8.59513	9.28974	6.42792	5.38184	6.60936	8.66508	9.99069
8.5547	8.91246	9.7097	10.563	5.92888	6.47083	8.12496	9.20531	10.5177
8.28829	8.99553	9.38233	10.5401	9.47268	4.68544	9.43415	10.0632	10.973
8.56985	8.42086	9.25208	10.2571	6.84752	5.43476	7.35525	8.89435	10.8386
8.72276	8.57545	9.24003	9.81114	8.94686	5.2455	9.18312	9.11139	10.8698
8.04076	8.63102	9.03075	9.37567	6.37468	5.16882	6.27278	9.27773	10.165
7.79171	8.32621	9.15633	9.53187	9.36665	6.2384	8.95883	9.15288	10.6514
8.57295	8.47726	9.13162	10.5956	9.22966	4.71594	9.64124	9.15255	11.2435
7.84568	8.62702	9.04036	9.22754	7.67655	6.02423	7.17999	9.41561	9.86668
8.01452	9.50604	9.8857	10.156	10.0417	4.81272	9.74352	9.86809	10.9017
8.17836	8.59149	9.52477	9.81423	5.59494	4.8273	6.00665	9.40197	10.4688
8.04958	8.65879	8.42656	9.49607	9.09659	5.33539	8.60117	9.13931	10.5455
8.56629	8.6986	9.40439	10.3513	6.09677	5.94937	5.9894	8.90869	10.1436
8.3607	9.35744	9.5464	10.3173	9.19761	4.83028	8.19655	9.77867	10.6995
8.13609	8.69837	9.27272	9.76152	8.96768	5.99489	9.24444	8.84902	10.7676
8.32882	8.16506	9.08595	9.49536	8.46154	5.61961	7.09926	9.16495	10.4357
8.40877	8.89542	9.32746	9.93709	9.09956	6.3626	8.37382	9.60279	10.725
8.65959	8.77018	9.06493	10.1352	7.47923	5.48235	8.06517	8.98597	11.155
8.16241	8.36813	9.19958	9.93652	9.70718	7.56042	8.85862	9.73619	10.8309
8.09745	8.32223	9.02983	9.72753	6.01184	5.06876	5.63941	9.09552	10.4712
8.06357	8.62503	9.43791	10.173	9.35661	5.97957	9.59419	9.72261	10.9115
8.15883	8.80875	8.95924	9.91089	9.28126	4.96164	9.26645	9.95023	10.7091
8.43729	8.24639	9.40265	9.87122	6.50107	5.96434	8.44534	9.39333	10.5437
7.12894	8.33944	8.37112	9.75063	7.36191	5.09995	8.34584	9.57161	10.3388
8.53601	8.78411	9.16861	10.1736	8.27694	5.46901	8.9533	9.51111	11.0131
8.49321	8.43347	8.88199	9.95872	9.00178	5.39666	9.44873	9.48455	10.8166
8.58022	8.82572	9.87539	10.1633	8.11403	5.98109	8.8144	9.38102	10.9994
8.70817	8.37249	9.3985	9.6248	5.704	5.0699	6.68755	8.83298	10.3295
7.77697	8.42738	8.80326	9.44963	7.13847	5.48415	8.01433	9.1235	10.3039
8.81252	9.21377	9.61852	10.0297	8.82433	5.25456	8.14458	9.66738	10.2922
8.34066	8.57876	9.76796	10.283	9.60842	6.41305	9.32403	9.6867	10.6043
8.28385	8.76187	9.13577	10.1847	6.25022	4.8051	9.32276	10.0392	10.7475
8.14089	8.51838	9.06882	9.40379	6.95268	5.85785	8.07772	9.40951	10.7602
8.29174	8.5252	9.13672	9.67749	8.86896	6.63001	8.78795	9.14963	11.0724
7.60758	8.20662	9.0872	9.05958	8.74507	5.25525	8.4856	9.32913	10.7029
8.10181	8.8259	9.62195	10.0453	9.10575	4.96442	8.54409	9.45865	10.6973
7.56041	8.16473	8.60666	9.45783	7.92751	5.37477	7.93952	9.01908	10.6938
7.96489	8.33301	8.7905	9.37566	8.84449	5.60937	7.95945	8.58743	10.4338
7.78421	8.05422	8.04239	9.52129	8.38701	4.74593	8.22387	9.56134	10.5617
7.82332	8.83031	8.90701	9.90115	9.74183	5.02806	8.72162	10.0061	10.637
8.68138	8.48764	9.25763	10.3619	8.66348	4.92623	8.99307	9.41013	10.9541
7.97548	8.3966	9.03404	9.51589	5.63602	4.75482	8.36178	9.24492	10.3117
7.8704	8.10107	8.82769	9.3301	6.71703	5.00333	6.42494	8.98838	10.4593
8.76771	8.33709	9.3559	10.4485	9.15232	8.01437	9.51375	9.65697	11.2734
8.22335	8.57849	8.817	9.92915	7.50764	5.04358	8.59357	9.65676	10.9025

7.91628	8.43526	8.19633	9.23012	7.84135	5.60276	7.29522	9.38725	10.4671
8.40074	8.17558	9.35238	9.96799	8.51222	4.88226	8.78081	9.1384	11.08
7.88255	8.31739	8.75773	9.57439	6.91376	5.2022	8.36525	9.12499	10.682
8.23897	8.11869	9.25884	9.54985	8.19284	5.52389	8.61462	9.30532	10.6367
8.1	8.33308	8.46661	9.73981	8.28208	6.58364	8.53141	9.52379	10.6799
8.65442	8.32196	9.33423	9.81168	10.2275	5.07452	5.30414	9.22595	10.5297
8.49498	9.1134	9.16397	10.2699	8.66745	4.62549	9.41083	10.049	10.8699
8.98998	8.92262	9.78108	10.5499	8.66601	7.02579	8.31059	9.44856	11.0472
8.23248	7.92357	8.96475	9.64139	7.35645	5.39219	7.2323	8.86765	10.4813
8.60029	8.6774	9.27697	9.97413	9.80527	5.16667	8.75241	9.87218	10.7872
8.36696	8.42235	9.04346	9.93028	9.41589	5.29737	8.45698	9.2518	10.8875
8.2345	8.41372	9.30708	9.94818	10.0414	5.31872	7.37042	9.32208	10.8559
7.39715	8.06144	8.47426	8.80573	7.79649	6.10439	7.63412	9.20985	10.2932
8.17519	8.98389	9.10855	10.4721	5.79888	5.22873	8.91092	10.3264	10.9524
9.11688	8.31301	9.35312	10.052	5.88982	5.62661	8.81263	9.65892	10.8318
8.20095	8.35841	9.27944	9.74209	8.85778	5.06254	8.99	9.42215	10.7715
8.02809	8.22676	8.73489	9.94983	8.33898	5.25493	8.46548	9.53759	10.721
7.49593	8.41824	8.43401	9.31439	8.26554	5.20926	6.34583	8.97259	10.144
8.36675	8.41451	8.72817	9.64504	6.09416	5.59469	6.5003	8.92086	10.3602
8.27591	8.61397	9.29293	10.0259	6.58031	6.44603	9.37633	9.65976	11.131
8.80719	8.63204	8.96282	10.066	5.51695	5.22758	6.4508	8.729	10.3705
8.38669	8.66455	9.10478	9.86036	9.44092	5.06551	8.7241	9.83214	10.5531
8.24374	8.72229	9.2757	10.0957	9.59339	5.69299	8.31441	9.19964	10.7552
8.76086	9.00098	9.36625	10.5395	9.36228	6.46132	9.07197	9.87	10.871
7.78525	8.54037	8.70636	9.3949	9.72789	6.22285	8.23661	9.19058	10.395
8.20726	8.48934	8.85307	9.69754	8.68665	5.79902	8.54605	9.46673	10.5338
8.83598	8.96119	9.40035	10.5264	8.98267	5.45576	9.64607	10.0277	10.9047
8.55476	8.3747	8.97806	10.4245	8.26784	4.92296	9.13689	9.5866	11.0813
8.8263	8.63468	9.40055	10.4439	9.06414	4.6819	9.53582	9.57909	11.2612
9.3154	8.73027	9.2688	10.668	5.62805	4.68019	8.79743	10.1084	11.0459
8.14524	8.38542	9.51795	10.077	8.85477	4.72336	9.04477	9.5779	10.6557
8.86011	8.77202	9.45525	10.0474	7.549	6.00139	7.64397	9.08328	10.8667
9.04459	8.84058	9.10863	10.5452	9.58172	6.93447	9.44703	9.96102	11.0379
7.3379	8.3204	8.80845	9.45829	9.08341	5.46434	8.7002	9.22929	10.4212
8.83948	8.66577	9.06124	10.3408	7.25076	5.24508	7.62125	9.29331	10.7783
8.68143	8.95661	8.97968	10.3271	7.48463	5.44062	8.34176	9.39157	10.6996
8.9197	9.07194	9.51411	10.5442	5.43813	5.04704	8.51526	9.1759	10.8626
8.44263	8.22757	9.34565	9.25391	8.31189	5.45449	7.43764	8.78362	10.4592
8.42024	8.55938	9.16945	9.50463	7.83043	6.36387	7.25128	8.90447	10.5063
7.51882	8.33586	7.98691	9.11274	6.29704	5.32487	6.61658	8.66709	9.91268
8.49326	8.72819	9.18886	9.4458	7.0191	5.97603	6.09609	8.84663	10.1868
7.96182	8.97091	9.30346	9.41567	8.69273	5.12681	8.58883	8.96506	10.7021
8.67421	8.86641	9.51071	10.1688	7.88721	5.69816	8.33715	9.33851	10.7571
8.41255	8.70515	9.25174	10.4873	9.23435	4.89699	8.98523	9.58867	10.7572
7.90939	8.44551	8.92338	9.23612	6.61443	5.24113	6.00194	8.49284	9.35795
7.3717	8.48352	8.9025	9.54649	9.86663	5.59785	8.29947	9.75309	10.1456
8.98372	8.87551	9.58608	10.2627	7.6452	5.78749	8.60136	8.88692	10.9891
8.50284	9.12509	9.84026	10.504	9.68378	5.15778	9.26155	10.0926	11.0204
8.85891	8.61916	9.3335	10.1566	6.51866	6.16945	6.79566	9.20527	10.4728
8.12321	9.01644	9.30745	10.244	9.45026	5.50596	8.94014	9.35236	10.6867
8.09472	8.70179	8.44077	9.76175	5.80526	4.85919	8.77747	9.29448	10.74
8.10077	9.00157	8.52811	10.0128	9.15059	5.2141	8.61832	9.2648	10.3391
8.4761	8.8511	9.18859	10.4231	9.57322	6.81747	9.57167	10.0652	10.9537
8.04517	8.49493	9.01227	9.43441	9.23969	4.85362	7.4313	8.74314	10.1022
8.3635	8.58723	8.59143	10.0926	8.83164	4.82803	7.95692	9.38942	10.3627
7.74228	8.0984	8.79559	9.09845	7.90658	4.63478	5.68377	8.84514	9.56254
8.30877	8.60108	9.1253	9.66266	5.54896	5.35731	8.84561	9.17963	10.7733
8.23588	8.40489	9.01859	9.93247	8.50886	5.58559	8.5127	9.73362	10.7684
8.10662	8.44491	8.8038	9.30952	9.08722	5.38682	8.10346	9.18463	10.3019

7.82258	8.64756	9.12654	9.62995	9.12841	4.97989	7.87592	9.28696	10.5912
8.27078	8.64715	9.43767	10.4927	5.61754	5.04561	9.16148	9.33428	10.6402
8.36722	8.78216	9.18717	9.85034	7.89281	5.04868	7.54896	9.31281	10.7852
8.59672	8.70967	9.35572	10.119	8.45942	5.27439	8.29117	9.55924	10.7106
9.05708	9.08857	8.84388	10.6482	8.60333	4.64927	9.42375	10.0586	10.8851
8.81996	8.9493	9.62379	10.5115	7.41803	5.67095	8.15989	9.28793	10.7849
8.60066	8.59669	9.30605	10.2628	8.73055	6.11261	9.16452	9.53618	11.1919
8.51316	8.65274	8.84137	10.3902	9.61526	4.95033	8.97913	10.3404	10.8329
8.84364	9.02804	9.42764	10.6526	9.99207	7.32651	9.93718	10.0776	11.368
0.95439	0.127211	0.074878	0.000245	0.021215	0.166491	0.296891	7.65E-06	0.018076

FTO	ALKBH5	Group
9.66137	8.82816	Tumor_G1
9.99146	8.85251	Tumor_G1
10.0588	8.87848	Tumor_G1
9.96206	8.60752	Tumor_G1
9.53594	8.54264	Tumor_G1
10.5418	9.56486	Tumor_G1
10.2937	9.29278	Tumor_G1
9.88029	8.90249	Tumor_G1
10.0689	9.11564	Tumor_G1
9.91572	9.05411	Tumor_G1
10.4094	8.78537	Tumor_G1
10.062	9.17749	Tumor_G1
9.9518	8.62025	Tumor_G1
9.86429	8.82483	Tumor_G1
10.2861	9.79465	Tumor_G1
10.0441	9.10336	Tumor_G1
9.99875	8.76836	Tumor_G1
9.83914	8.98408	Tumor_G1
9.81007	8.97475	Tumor_G1
10.1256	8.84396	Tumor_G1
9.83807	9.52681	Tumor_G1
10.5431	9.37842	Tumor_G1
10.1873	8.9458	Tumor_G1
9.7888	8.70032	Tumor_G1
10.5124	8.96416	Tumor_G1
10.0589	9.05177	Tumor_G1
10.347	9.0562	Tumor_G1
9.61173	8.7742	Tumor_G1
9.85171	8.98633	Tumor_G1
9.6509	8.8611	Tumor_G1
9.83313	8.28936	Tumor_G1
9.79354	8.6238	Tumor_G2
11.2016	8.93592	Tumor_G2
10.1388	8.65295	Tumor_G2
10.0047	9.05572	Tumor_G2
10.1266	8.61564	Tumor_G2
8.29008	8.32456	Tumor_G2
9.73585	8.4798	Tumor_G2
9.56413	8.60033	Tumor_G2
10.2083	8.7521	Tumor_G2
9.28855	7.93259	Tumor_G2
9.26584	8.43047	Tumor_G2
9.72108	8.41689	Tumor_G2
9.91248	8.94746	Tumor_G2
10.1971	8.35478	Tumor_G2
10.0633	8.61894	Tumor_G2
9.51402	8.63279	Tumor_G2
10.4731	9.31484	Tumor_G2
10.0874	8.72131	Tumor_G2
10.3624	8.61304	Tumor_G2
9.2782	8.83918	Tumor_G2
10.101	8.87128	Tumor_G2
9.47944	8.38982	Tumor_G2
9.57742	8.67445	Tumor_G2
10.1856	8.95508	Tumor_G2
9.54644	8.82211	Tumor_G2
8.61925	8.63773	Tumor_G2
10.3477	8.77648	Tumor_G2

9.60143	8.8379	Tumor_G2
9.89095	8.57498	Tumor_G2
9.60181	8.66978	Tumor_G2
10.1273	8.8895	Tumor_G2
10.5462	8.71724	Tumor_G2
9.76681	8.497	Tumor_G2
10.6749	8.76296	Tumor_G2
9.5896	8.57823	Tumor_G2
9.38629	9.15748	Tumor_G2
9.83893	9.02203	Tumor_G2
8.52959	8.60051	Tumor_G2
10.5724	8.74075	Tumor_G2
9.79722	8.83933	Tumor_G2
10.1961	8.3356	Tumor_G2
10.5262	8.69432	Tumor_G2
10.3596	8.92628	Tumor_G2
9.95381	8.53479	Tumor_G2
9.705	8.49639	Tumor_G2
10.0758	8.89121	Tumor_G2
10.6718	8.88033	Tumor_G2
10.0258	9.11395	Tumor_G2
9.5874	8.19251	Tumor_G2
9.07244	8.71662	Tumor_G2
10.0251	8.8839	Tumor_G2
10.3367	8.8976	Tumor_G2
10.3855	8.76294	Tumor_G2
10.0297	8.53515	Tumor_G2
9.94095	8.72783	Tumor_G2
9.46276	8.26245	Tumor_G2
9.81138	7.75379	Tumor_G2
10.2688	8.9608	Tumor_G2
10.2889	8.64111	Tumor_G2
10.1764	8.27803	Tumor_G2
10.8502	8.10053	Tumor_G2
9.4523	8.70176	Tumor_G2
9.46241	8.567	Tumor_G2
10.8517	9.04582	Tumor_G2
9.6995	8.34721	Tumor_G2
10.0164	8.83524	Tumor_G2
9.06867	7.89187	Tumor_G2
10.3198	8.54934	Tumor_G2
9.68404	8.90601	Tumor_G2
10.2312	8.50387	Tumor_G2
9.75586	8.53888	Tumor_G2
10.1232	8.36768	Tumor_G2
9.97433	8.24664	Tumor_G2
9.92494	8.32026	Tumor_G2
9.68443	8.51753	Tumor_G2
9.64989	8.38237	Tumor_G2
10.1415	8.78127	Tumor_G2
9.69384	9.06549	Tumor_G2
9.92443	8.54056	Tumor_G2
10.067	8.82236	Tumor_G2
10.1145	8.11319	Tumor_G2
9.95806	8.60942	Tumor_G2
9.7055	8.56829	Tumor_G2
9.38201	9.02163	Tumor_G2
9.42536	9.16948	Tumor_G2
9.80101	8.18868	Tumor_G2

8.91922	8.52691	Tumor_G2
9.97556	8.3134	Tumor_G2
8.94838	8.48944	Tumor_G2
9.89566	8.70859	Tumor_G2
9.78786	8.41958	Tumor_G2
10.2544	8.52027	Tumor_G2
10.0294	8.70223	Tumor_G2
9.16914	8.5419	Tumor_G2
9.71494	8.15024	Tumor_G2
10.8714	9.45516	Tumor_G2
9.76529	8.93031	Tumor_G2
10.2804	9.58921	Tumor_G2
10.1922	8.94396	Tumor_G2
10.0972	8.62206	Tumor_G2
10.2125	9.21359	Tumor_G2
9.88319	9.27595	Tumor_G2
10.4888	9.08154	Tumor_G2
9.91359	8.7788	Tumor_G2
9.42221	8.71792	Tumor_G2
9.60173	8.92321	Tumor_G2
9.88428	9.01851	Tumor_G2
9.80655	8.97442	Tumor_G2
10.1553	9.13739	Tumor_G2
9.57283	8.77135	Tumor_G2
11.0509	9.52713	Tumor_G2
9.82771	8.61685	Tumor_G2
10.8246	10.1511	Tumor_G2
10.2625	8.85403	Tumor_G2
9.48062	9.1067	Tumor_G2
10.0545	8.94232	Tumor_G2
10.0569	9.02442	Tumor_G2
10.238	8.54885	Tumor_G2
9.55534	8.19777	Tumor_G2
9.98936	8.94629	Tumor_G2
9.8328	9.23782	Tumor_G2
9.62984	8.99112	Tumor_G2
9.95074	8.51373	Tumor_G2
9.95197	8.76822	Tumor_G2
10.2226	8.93723	Tumor_G2
9.83801	8.248	Tumor_G2
9.69955	9.09318	Tumor_G2
10.7159	9.06323	Tumor_G2
10.4038	9.13767	Tumor_G2
8.77766	9.14962	Tumor_G2
9.3925	9.04988	Tumor_G2
10.4028	8.70035	Tumor_G2
9.71221	8.93119	Tumor_G2
9.64613	9.14678	Tumor_G2
9.7992	8.44431	Tumor_G2
9.89335	9.26529	Tumor_G2
9.20988	8.24036	Tumor_G2
9.51584	8.36953	Tumor_G2
9.11278	8.20836	Tumor_G2
9.63222	8.27958	Tumor_G2
9.28732	8.44597	Tumor_G2
9.8773	8.82017	Tumor_G2
9.41188	8.47802	Tumor_G2
9.99823	8.2359	Tumor_G2
9.80764	8.34913	Tumor_G2

9.00095	8.74618	Tumor_G2
9.63752	8.44688	Tumor_G2
9.82286	8.24801	Tumor_G2
10.0845	8.66507	Tumor_G2
10.0042	8.53384	Tumor_G2
9.88687	8.60343	Tumor_G2
10.4895	8.88446	Tumor_G2
9.52882	9.26259	Tumor_G2
9.10082	8.60765	Tumor_G2
8.838	8.64315	Tumor_G2
9.53263	8.5879	Tumor_G2
9.9282	8.4747	Tumor_G2
8.78161	8.14154	Tumor_G2
10.3752	8.75583	Tumor_G2
9.29776	8.14944	Tumor_G2
9.30569	8.55326	Tumor_G2
9.91278	8.63354	Tumor_G2
9.93654	8.75696	Tumor_G2
10.0556	8.99904	Tumor_G2
10.0682	8.83198	Tumor_G2
10.2319	8.931	Tumor_G2
10.2532	8.39284	Tumor_G2
9.7775	8.22018	Tumor_G2
9.95726	8.40325	Tumor_G2
10.1013	8.44814	Tumor_G2
9.62508	8.19827	Tumor_G2
9.93909	8.45765	Tumor_G2
9.58107	8.34946	Tumor_G2
11.4616	8.16802	Tumor_G2
10.12	8.36853	Tumor_G2
9.44601	8.71387	Tumor_G2
9.34315	8.3779	Tumor_G2
9.88137	8.34351	Tumor_G2
9.32185	8.11494	Tumor_G2
10.3315	8.48617	Tumor_G2
10.1808	8.43166	Tumor_G2
10.274	8.52885	Tumor_G2
10.1082	8.35212	Tumor_G2
9.77016	8.587	Tumor_G2
9.95379	8.84332	Tumor_G2
10.5533	9.02433	Tumor_G2
9.90627	8.67569	Tumor_G2
9.88463	8.743	Tumor_G2
10.3778	8.16316	Tumor_G2
10.2039	8.74967	Tumor_G2
9.59235	8.3819	Tumor_G2
9.6803	8.61782	Tumor_G2
9.84766	8.51093	Tumor_G2
10.2937	9.01294	Tumor_G2
10.0537	8.34665	Tumor_G2
9.81108	8.11827	Tumor_G2
9.92625	8.10649	Tumor_G2
10.0286	8.38898	Tumor_G2
9.82914	8.39399	Tumor_G2
8.65439	8.02894	Tumor_G2
10.1609	8.52923	Tumor_G2
10.0948	8.33017	Tumor_G2
9.95992	8.17988	Tumor_G2
9.69504	8.32794	Tumor_G2

10.1923	8.11245	Tumor_G2
9.51698	8.3101	Tumor_G2
9.68944	8.45505	Tumor_G2
9.88704	8.4456	Tumor_G2
10.0748	8.20111	Tumor_G2
9.64585	8.11371	Tumor_G2
9.78307	8.39895	Tumor_G2
10.2892	8.32868	Tumor_G2
9.87609	8.32589	Tumor_G2
0.082036	8.71E-07	NA

Supplementary Table 2

Genes	ALKBH5	ZC3H13	IGF2BP1	HNRNPA2	YTHDC1	METTL14	HNRNPC	RBMX
ALKBH5	1	0.15838	-0.18828	-0.20615	-0.06437	0.030416	0.029701	-0.09358
ZC3H13	0.15838	1	0.031885	0.246422	0.347239	0.23127	0.175404	-0.0153
IGF2BP1	-0.18828	0.031885	1	0.254195	0.066154	0.08997	0.141938	0.318164
HNRNPA2	-0.20615	0.246422	0.254195	1	0.649345	0.641299	0.619112	0.404161
YTHDC1	-0.06437	0.347239	0.066154	0.649345	1	0.747627	0.681674	0.345539
METTL14	0.030416	0.23127	0.08997	0.641299	0.747627	1	0.742038	0.453042
HNRNPC	0.029701	0.175404	0.141938	0.619112	0.681674	0.742038	1	0.560942
RBMX	-0.09358	-0.0153	0.318164	0.404161	0.345539	0.453042	0.560942	1
RBM15	-0.15322	0.138204	0.359788	0.551797	0.446354	0.393221	0.392569	0.377028

RBM15
-0.15322
0.138204
0.359788
0.551797
0.446354
0.393221
0.392569
0.377028
1

Supplementary Table 3

	times	status	METTL14	YTHDC1	IGF2BP1	HNRNPC	HNRNPA2	RBMX
TARGET-3	1.334247	1	7.93695	8.9132	5.52877	9.83043	11.1997	9.07414
TARGET-3	15.20548	0	7.78644	8.81789	5.95958	9.98402	10.2305	9.52646
TARGET-3	14.50959	0	8.48396	9.43768	9.40404	10.3409	11.1121	9.53106
TARGET-3	2.526027	1	7.85604	8.66669	8.68185	10.3458	10.3743	9.63631
TARGET-3	0.882192	1	8.805	9.34679	8.86046	10.4522	10.8448	9.32584
TARGET-3	0.556164	1	7.88024	8.04543	8.65489	8.23561	10.7838	8.92645
TARGET-3	1.279452	1	8.30731	9.08548	9.37592	10.0928	11.0269	9.35885
TARGET-3	0.671233	1	7.39381	9.05302	5.67746	10.0585	10.5666	9.38678
TARGET-3	1.484932	1	7.41424	8.68925	7.75075	9.29801	10.446	9.39121
TARGET-3	15.24384	0	8.31635	9.62503	8.13616	10.145	11.2315	9.14288
TARGET-3	13.32329	0	8.05388	8.79525	8.32418	10.1097	10.9721	8.90503
TARGET-3	7.868493	0	7.99366	8.77222	10.0252	10.7117	10.8614	10.0748
TARGET-3	3.073973	1	7.99528	8.56386	6.54611	9.60849	10.5444	8.60664
TARGET-3	12.91781	0	7.6401	8.62345	8.45623	9.65511	10.702	9.45577
TARGET-3	12.59178	0	8.02303	8.53535	9.24427	10.0959	10.5966	9.42014
TARGET-3	1.120548	1	8.0571	9.11375	8.36073	9.57839	10.7176	9.41032
TARGET-3	10.56164	0	8.59227	8.89868	8.99378	10.3226	10.7398	9.61727
TARGET-3	3.369863	1	7.9189	8.75658	9.01625	10.0385	10.8527	9.08801
TARGET-3	10.59178	0	7.61279	8.42659	8.48505	9.90084	10.7779	9.61733
TARGET-3	7.243836	0	8.59269	8.70992	5.6441	10.4621	10.9093	9.45101
TARGET-3	1.610959	1	8.05916	8.51367	9.23348	10.3019	10.7048	9.9268
TARGET-3	1.70137	1	7.43599	8.06981	7.73493	9.06495	10.5468	8.57165
TARGET-3	1.19726	1	7.42422	8.918	6.73439	9.77204	10.6017	8.76293
TARGET-3	0.934247	1	8.24315	8.5923	5.76289	10.4518	10.6167	9.98704
TARGET-3	2.967123	1	7.77141	7.76863	6.58767	9.91385	10.3538	9.55648
TARGET-3	2.167123	1	8.14646	8.32057	7.74396	10.0236	10.3068	9.16702
TARGET-3	0.035616	1	7.53679	8.51024	9.07997	9.79218	10.5399	9.6429
TARGET-3	3.676712	1	8.42711	9.1942	8.95858	10.3414	10.6987	9.29474
TARGET-3	12.79452	0	7.39985	8.33959	8.51726	10.1129	11.03	9.08725
TARGET-3	3.219178	1	7.43276	8.72874	8.88825	9.74441	10.3835	9.24142
TARGET-3	4.791781	0	8.01931	8.77161	9.00154	10.0375	10.6395	9.76574
TARGET-3	1.246575	1	8.1642	8.54845	9.28361	10.5626	10.9535	10.3101
TARGET-3	1.216438	1	7.73073	8.53831	9.29501	9.68789	10.8522	9.15432
TARGET-3	3.860274	1	7.56174	8.60667	7.35271	9.58518	10.5193	9.41161
TARGET-3	1.306849	1	8.27069	8.89126	8.50408	10.2047	10.7749	9.56226
TARGET-3	11.74521	0	7.91549	8.69456	6.34933	9.59494	10.189	8.70501
TARGET-3	8.863014	0	8.23907	8.88095	9.51936	10.27	11.1046	9.82515
TARGET-3	1	1	7.52105	8.23726	9.00158	9.43071	10.3242	8.99682
TARGET-3	5.621918	0	8.13648	8.99855	8.6633	10.3201	10.8681	10.2143
TARGET-3	1.213699	1	8.01798	8.50722	7.11637	9.77023	10.7918	9.12903
TARGET-3	1.99726	1	8.2181	8.67266	8.94032	10.2382	10.8479	9.43522
TARGET-3	1.367123	1	7.66111	8.81611	9.07714	9.71	10.5295	9.30493
TARGET-3	3.767123	0	8.33747	8.47056	9.05072	9.87756	10.7478	9.65524
TARGET-3	0.657534	1	8.14777	9.30496	8.28509	10.2099	10.7118	9.2459
TARGET-3	10.37808	0	7.94385	8.76617	8.20336	9.63129	10.5625	9.23718
TARGET-3	0.6	1	8.05025	8.74614	7.82452	10.3138	10.7434	9.20448
TARGET-3	1.668493	0	7.57717	8.35429	8.69097	9.76942	10.2905	9.62542
TARGET-3	3.254795	1	8.30145	8.71192	9.06197	10.1732	11.1441	9.19118
TARGET-3	1.912329	1	7.36958	8.07675	8.40793	8.98387	10.122	9.41499
TARGET-3	1.821918	1	7.98455	8.64029	9.10927	10.4434	10.8913	9.49436
TARGET-3	8.213699	1	7.43371	8.39438	8.96987	9.54049	10.5187	9.17984
TARGET-3	11.74247	0	8.25778	8.83558	8.46769	10.3575	11.2202	9.25
TARGET-3	0.906849	1	8.05518	8.34239	6.94783	10.0117	10.6377	9.26174
TARGET-3	2.90137	1	6.97472	8.33148	6.02203	9.8191	9.72733	8.64695
TARGET-3	7.506849	0	8.08507	8.83732	9.10999	10.2806	10.6118	9.74136
TARGET-3	10.25205	0	7.42028	8.78582	7.21342	9.21504	10.4831	8.56531
TARGET-3	1.693151	1	7.41019	8.44846	5.68305	8.76514	10.0862	8.70363
TARGET-3	14.29041	0	8.56412	9.01395	5.60339	10.3577	10.269	9.64093

TARGET-3	1.139726	1	8.10993	8.65481	8.9441	9.99453	10.7647	10.0544
TARGET-3	7.027397	0	6.07873	7.29509	8.59562	8.39584	9.5095	8.40985
TARGET-3	9.761644	0	8.19229	8.76525	7.94049	10.3479	10.9651	9.45508
TARGET-3	1.126027	1	7.7986	9.12552	5.68616	10.1665	11.0233	10.1726
TARGET-3	4.356164	1	8.02696	8.88995	7.89209	9.73442	10.3695	9.14604
TARGET-3	6.252055	0	8.40829	9.11384	5.67731	9.95621	10.6556	10.0387
TARGET-3	7.605479	0	8.5769	9.17604	9.55888	10.4846	11.0021	9.04934
TARGET-3	10.11233	1	7.79179	8.8523	8.32418	9.80247	10.716	9.24935
TARGET-3	4.276712	1	7.00228	7.80527	7.91844	8.42499	9.92316	9.02972
TARGET-3	11.48219	0	7.3033	8.49346	7.5522	9.35319	10.4526	8.81236
TARGET-3	1.205479	1	8.30711	8.92323	5.56568	10.3123	10.6718	9.21077
TARGET-3	2.09589	1	8.04851	9.325	7.52199	10.2336	10.7086	9.2308
TARGET-3	2.775342	1	8.11829	8.93275	9.02356	9.98074	10.8277	9.24833
TARGET-3	1.194521	1	7.91723	8.9059	8.3796	9.6236	10.5456	9.34579
TARGET-3	5.049315	0	8.38271	9.01721	7.74792	9.50274	10.5631	8.90475
TARGET-3	1.123288	1	8.2504	8.99523	8.43292	10.5875	10.7089	10.3869
TARGET-3	7.928767	0	7.98046	9.06366	8.44644	9.93787	10.5157	8.98118
TARGET-3	7.747945	0	7.87003	8.952	8.38836	10.2003	10.8691	9.66325
TARGET-3	1.153425	1	7.73226	8.68112	9.49667	9.72787	10.6385	9.58311
TARGET-3	0.887671	1	8.0263	8.26851	9.00234	9.48678	10.5071	9.30324
TARGET-3	2.224658	1	7.7747	8.90716	6.06542	9.95716	10.7423	8.88371
TARGET-3	5.030137	1	8.35247	9.09398	9.52172	9.91501	10.864	9.43612
TARGET-3	5.882192	0	7.5172	8.60535	8.60395	10.0711	11.0137	9.75577
TARGET-3	1.926027	1	8.37847	8.95351	8.98607	10.4828	10.7352	9.85284
TARGET-3	4.39726	1	7.78422	9.0066	8.71767	10.0589	10.5483	9.07942
TARGET-3	6.463014	0	7.86023	9.1565	8.54526	10.2459	10.9252	9.11854
TARGET-3	2.156164	1	8.55678	9.48279	8.80577	10.2293	10.6989	9.68878
TARGET-3	10.07671	0	7.36593	8.33548	6.52889	9.69891	10.4249	8.71099
TARGET-3	4.449315	1	7.02268	8.14942	8.86656	9.22872	10.4196	9.17072
TARGET-3	13.83014	0	7.79965	8.8459	9.07122	9.9023	10.7612	9.03463
TARGET-3	1.723288	0	7.46975	8.54798	9.02391	10.1925	10.7782	9.63785
TARGET-3	0.520548	1	7.95655	9.0333	7.90493	9.94374	11.0839	9.11936
TARGET-3	1.008219	1	8.15103	8.61998	8.35011	10.3247	10.5608	9.21058
TARGET-3	0.838356	1	7.77435	8.31155	8.99459	9.61151	10.7765	9.68606
TARGET-3	2.164384	1	6.99383	8.11553	8.3299	9.32899	10.158	9.35614
TARGET-3	1.136986	1	7.8815	8.46999	8.90617	9.33912	10.7528	9.31728
TARGET-3	3.742466	1	8.82909	9.23492	7.83542	10.2087	10.6985	9.2972
TARGET-3	6.369863	1	7.94388	9.31662	7.50007	10.4173	10.754	8.81221
TARGET-3	5.547945	0	8.12314	8.6353	9.1444	10.2288	10.9228	9.02338
TARGET-3	3.383562	1	8.01022	8.69508	8.96785	10.1203	10.9126	9.30284
TARGET-3	4.454795	0	8.44992	9.17435	8.73368	9.98302	11.1309	9.21085
TARGET-3	10.2	0	7.2822	8.62724	7.94158	9.55507	10.7858	9.40132
TARGET-3	4.917808	1	7.83362	8.66142	9.38422	10.1506	10.1812	9.65377
TARGET-3	0.005479	0	6.98459	7.68068	8.91669	9.30209	10.1756	9.58877
TARGET-3	4.545205	0	7.76037	8.31775	8.07245	9.84347	9.84786	9.48468
TARGET-3	10.06027	0	8.17601	8.83996	5.83168	9.69874	10.8533	8.92188
TARGET-3	5.375342	0	8.84062	9.28127	8.66898	10.3896	10.7108	10.0178
TARGET-3	13.69315	0	7.88618	8.57885	7.11826	9.95659	10.625	8.54371
TARGET-3	2.00274	1	8.11066	9.0412	8.29338	10.1511	10.9151	9.00623
TARGET-3	13.07671	0	7.26121	8.44993	7.7951	9.62619	10.3804	8.79347
TARGET-3	3.054795	1	8.04093	8.89284	9.19934	10.0111	10.957	9.8197
TARGET-3	9.827397	0	7.24902	8.20236	6.42792	9.28974	9.99069	8.66508
TARGET-3	2.619178	1	7.85587	9.16193	5.92888	10.563	10.5177	9.20531
TARGET-3	0.863014	1	8.48692	8.97014	9.47268	10.5401	10.973	10.0632
TARGET-3	2.939726	1	8.12883	9.18315	6.84752	10.2571	10.8386	8.89435
TARGET-3	11.67945	0	7.76633	8.49919	7.98693	9.8791	10.7154	8.94551
TARGET-3	10.38904	0	7.94033	8.81602	7.95358	9.83689	10.5924	9.02206
TARGET-3	0.687671	1	8.23291	8.89068	8.94686	9.81114	10.8698	9.11139
TARGET-3	4.556164	1	7.6444	8.42011	6.37468	9.37567	10.165	9.27773

TARGET-3	7.871233	0	8.42465	8.93838	7.68168	10.2293	10.7258	9.36284
TARGET-3	3.961644	0	7.35317	8.30037	6.96179	9.28922	10.6141	9.08262
TARGET-3	1.186301	1	7.73449	8.38646	9.36665	9.53187	10.6514	9.15288
TARGET-3	3.821918	1	8.34898	8.79311	9.22966	10.5956	11.2435	9.15255
TARGET-3	7.158904	0	7.46327	8.55787	5.9011	9.87504	10.6719	9.11938
TARGET-3	12.05205	0	7.08764	8.01967	7.67655	9.22754	9.86668	9.41561
TARGET-3	9.191781	0	7.77668	8.44639	7.38323	9.46755	9.86852	8.91005
TARGET-3	12.66301	0	7.45216	8.3109	7.58666	9.08937	10.2975	8.48346
TARGET-3	0.860274	1	8.49741	9.02192	10.0417	10.156	10.9017	9.86809
TARGET-3	13.52329	0	8.40893	8.9474	5.59494	9.81423	10.4688	9.40197
TARGET-3	5.90411	0	7.0377	8.40564	7.43435	9.30334	10.2758	8.97034
TARGET-3	7.339726	0	7.78143	8.68662	8.65647	9.54317	10.3898	8.8078
TARGET-3	1.79726	1	7.5937	8.47655	9.09659	9.49607	10.5455	9.13931
TARGET-3	0.635616	1	8.25531	8.29074	6.09677	10.3513	10.1436	8.90869
TARGET-3	11.79726	0	8.32992	8.83826	9.19761	10.3173	10.6995	9.77867
TARGET-3	3.517808	1	7.93784	8.31178	8.96768	9.76152	10.7676	8.84902
TARGET-3	2.830137	1	7.46728	8.47586	8.46154	9.49536	10.4357	9.16495
TARGET-3	11.96712	0	7.14799	8.12685	8.388	9.32282	10.4424	8.7217
TARGET-3	5.980822	0	7.64017	8.5235	8.62788	9.58274	10.6561	8.95598
TARGET-3	7.224658	0	7.1994	7.89508	5.97527	8.87615	10.0468	8.71823
TARGET-3	11.33151	0	8.03172	8.80869	9.19829	9.96695	10.4977	9.76583
TARGET-3	2.2	1	7.85844	8.70752	9.09956	9.93709	10.725	9.60279
TARGET-3	6.876712	0	7.96317	8.47894	8.55228	9.92787	10.7104	9.46655
TARGET-3	12.66027	0	8.28727	8.85532	7.47923	10.1352	11.155	8.98597
TARGET-3	7.989041	0	7.77198	8.6494	8.42603	9.32705	10.6632	8.55037
TARGET-3	4.487671	0	8.07611	8.94494	8.26217	9.6874	10.8254	8.68983
TARGET-3	0.435616	1	7.81883	8.68548	9.70718	9.93652	10.8309	9.73619
TARGET-3	8.446575	0	7.51091	8.56621	8.2181	9.70972	10.3343	9.29453
TARGET-3	6.967123	1	8.06527	8.88016	6.01184	9.72753	10.4712	9.09552
TARGET-3	1.589041	1	8.12781	8.73769	9.35661	10.173	10.9115	9.72261
TARGET-3	5.29863	0	7.94284	8.66748	9.28126	9.91089	10.7091	9.95023
TARGET-3	0.665753	1	8.00981	8.62163	6.50107	9.87122	10.5437	9.39333
TARGET-3	11.14247	0	7.74478	8.6462	8.56135	9.39017	10.4049	9.07697
TARGET-3	7.517808	0	7.36118	8.21507	7.57303	9.16809	10.4899	8.72016
TARGET-3	13.07945	0	7.63915	8.26812	7.36191	9.75063	10.3388	9.57161
TARGET-3	1.345205	1	8.19444	8.83273	8.27694	10.1736	11.0131	9.51111
TARGET-3	0.909589	1	8.35266	8.81172	9.00178	9.95872	10.8166	9.48455
TARGET-3	1.967123	1	8.25538	9.20185	8.11403	10.1633	10.9994	9.38102
TARGET-3	2.558904	1	8.28049	8.16919	5.704	9.6248	10.3295	8.83298
TARGET-3	0.90411	1	7.34179	8.17507	7.13847	9.44963	10.3039	9.1235
TARGET-3	10.71233	0	8.34572	9.04113	8.82433	10.0297	10.2922	9.66738
TARGET-3	7.10411	0	8.23015	8.63417	9.60842	10.283	10.6043	9.6867
TARGET-3	5.150685	0	8.08029	8.54278	6.99118	9.92361	10.8178	9.11764
TARGET-3	0.539726	1	8.25893	9.10322	6.25022	10.1847	10.7475	10.0392
TARGET-3	3.20274	1	7.71163	8.80004	6.95268	9.40379	10.7602	9.40951
TARGET-3	12.32603	0	7.47074	8.5045	8.86896	9.67749	11.0724	9.14963
TARGET-3	2.293151	1	7.3255	8.40994	8.74507	9.05958	10.7029	9.32913
TARGET-3	6.539726	0	7.44297	8.64271	7.58225	9.71058	10.6686	9.3677
TARGET-3	4.753425	1	7.8985	9.10105	9.10575	10.0453	10.6973	9.45865
TARGET-3	2.726027	1	7.3531	8.42896	7.92751	9.45783	10.6938	9.01908
TARGET-3	14.2137	0	7.4123	8.32288	8.84449	9.37566	10.4338	8.58743
TARGET-3	13.92055	0	7.29317	8.42422	8.38701	9.52129	10.5617	9.56134
TARGET-3	2.090411	1	7.50362	8.6181	9.74183	9.90115	10.637	10.0061
TARGET-3	9.824658	0	8.40479	9.0723	8.66348	10.3619	10.9541	9.41013
TARGET-3	2.520548	1	7.52104	8.5073	5.63602	9.51589	10.3117	9.24492
TARGET-3	10.34521	1	7.50386	8.49158	6.71703	9.3301	10.4593	8.98838
TARGET-3	1.805479	1	8.869	9.03028	9.15232	10.4485	11.2734	9.65697
TARGET-3	4.646575	0	7.89437	8.82545	7.50764	9.92915	10.9025	9.65676
TARGET-3	2.249315	1	7.26709	8.50508	7.84135	9.23012	10.4671	9.38725

TARGET-3	10.76438	0	8.16592	9.09312	8.51222	9.96799	11.08	9.1384
TARGET-3	0.564384	1	7.76126	8.52117	6.91376	9.57439	10.682	9.12499
TARGET-3	2.441096	1	8.25054	8.67414	8.19284	9.54985	10.6367	9.30532
TARGET-3	2.586301	1	7.76178	8.5188	8.28208	9.73981	10.6799	9.52379
TARGET-3	5.967123	0	8.16947	9.03467	10.2275	9.81168	10.5297	9.22595
TARGET-3	1.106849	1	8.55402	8.67235	8.66745	10.2699	10.8699	10.049
TARGET-3	5.29589	1	8.07696	9.06015	8.66601	10.5499	11.0472	9.44856
TARGET-3	12.77534	0	7.5726	8.55349	7.35645	9.64139	10.4813	8.86765
TARGET-3	0.775342	1	7.91519	8.58039	9.80527	9.97413	10.7872	9.87218
TARGET-3	6.926027	1	7.88826	8.64302	9.41589	9.93028	10.8875	9.2518
TARGET-3	3.454795	1	8.09805	8.81421	10.0414	9.94818	10.8559	9.32208
TARGET-3	7.608219	0	7.10663	7.99426	7.79649	8.80573	10.2932	9.20985
TARGET-3	1.980822	1	8.25877	9.01995	5.79888	10.4721	10.9524	10.3264
TARGET-3	1.178082	1	8.60989	8.78858	5.88982	10.052	10.8318	9.65892
TARGET-3	0.410959	1	8.25564	8.77721	8.85778	9.74209	10.7715	9.42215
TARGET-3	1.073973	1	8.01204	8.57473	8.33898	9.94983	10.721	9.53759
TARGET-3	1.287671	1	7.39434	8.09062	8.26554	9.31439	10.144	8.97259
TARGET-3	7.339726	1	7.78095	8.38855	6.09416	9.64504	10.3602	8.92086
TARGET-3	1.273973	1	8.21045	8.50285	6.58031	10.0259	11.131	9.65976
TARGET-3	2.821918	0	8.13282	8.73499	5.51695	10.066	10.3705	8.729
TARGET-3	5.632877	1	8.11901	8.61325	9.44092	9.86036	10.5531	9.83214
TARGET-3	2.791781	1	8.10323	8.60285	9.59339	10.0957	10.7552	9.19964
TARGET-3	4.087671	1	8.65283	8.96683	9.36228	10.5395	10.871	9.87
TARGET-3	7.339726	1	7.19466	8.20035	9.72789	9.3949	10.395	9.19058
TARGET-3	10.79726	0	7.61585	8.5128	8.68665	9.69754	10.5338	9.46673
TARGET-3	10.85753	0	8.35549	8.8293	8.98267	10.5264	10.9047	10.0277
TARGET-3	10.37534	0	8.26073	8.86916	8.26784	10.4245	11.0813	9.5866
TARGET-3	7.594521	0	8.53678	9.05681	9.06414	10.4439	11.2612	9.57909
TARGET-3	9.079452	0	8.91843	9.18862	5.62805	10.668	11.0459	10.1084
TARGET-3	0.778082	1	8.16116	8.60481	8.85477	10.077	10.6557	9.5779
TARGET-3	9.956164	0	8.25725	8.963	7.549	10.0474	10.8667	9.08328
TARGET-3	6.536986	0	8.77459	8.94845	9.58172	10.5452	11.0379	9.96102
TARGET-3	2.265753	1	7.2176	8.06567	9.08341	9.45829	10.4212	9.22929
TARGET-3	10.23562	0	8.44909	8.9729	7.25076	10.3408	10.7783	9.29331
TARGET-3	6.115068	1	8.13081	8.69764	7.48463	10.3271	10.6996	9.39157
TARGET-3	6.909589	1	8.18962	9.10374	5.43813	10.5442	10.8626	9.1759
TARGET-3	11.06849	0	7.68844	8.63896	8.31189	9.25391	10.4592	8.78362
TARGET-3	10.14247	0	7.3619	8.42017	7.83043	9.50463	10.5063	8.90447
TARGET-3	10.92603	0	6.87112	7.77478	6.29704	9.11274	9.91268	8.66709
TARGET-3	2.139726	1	7.32842	8.09635	7.0191	9.4458	10.1868	8.84663
TARGET-3	5.120548	0	8.15119	8.93936	8.69273	9.41567	10.7021	8.96506
TARGET-3	10.53151	0	7.96473	8.80342	7.88721	10.1688	10.7571	9.33851
TARGET-3	7.69863	0	7.96038	8.88522	9.23435	10.4873	10.7572	9.58867
TARGET-3	4.676712	1	7.19984	7.62537	6.61443	9.23612	9.35795	8.49284
TARGET-3	0.29589	1	7.07745	8.02874	9.86663	9.54649	10.1456	9.75309
TARGET-3	8.216438	1	7.94934	9.0914	7.6452	10.2627	10.9891	8.88692
TARGET-3	5.238356	1	8.35173	8.65857	9.68378	10.504	11.0204	10.0926
TARGET-3	10.06849	0	8.24429	8.76361	6.51866	10.1566	10.4728	9.20527
TARGET-3	4.717808	1	7.53384	8.25191	9.45026	10.244	10.6867	9.35236
TARGET-3	2.663014	1	7.60035	8.36973	5.80526	9.76175	10.74	9.29448
TARGET-3	0.871233	1	7.17324	8.33777	9.15059	10.0128	10.3391	9.2648
TARGET-3	2.484932	1	8.1187	8.75022	9.57322	10.4231	10.9537	10.0652
TARGET-3	10.47945	0	7.06729	7.773	9.23969	9.43441	10.1022	8.74314
TARGET-3	10.10137	0	7.77643	8.89213	8.83164	10.0926	10.3627	9.38942
TARGET-3	6.912329	1	7.03863	8.26184	7.90658	9.09845	9.56254	8.84514
TARGET-3	10.65753	0	7.51336	8.49437	5.54896	9.66266	10.7733	9.17963
TARGET-3	1.567123	1	7.87299	8.86702	8.50886	9.93247	10.7684	9.73362
TARGET-3	2.060274	1	7.22368	8.0284	9.08722	9.30952	10.3019	9.18463
TARGET-3	3.99726	1	7.40854	8.5582	9.12841	9.62995	10.5912	9.28696

TARGET-3	6.479452	0	7.93709	8.64474	5.61754	10.4927	10.6402	9.33428
TARGET-3	3.553425	1	8.0821	8.78917	7.89281	9.85034	10.7852	9.31281
TARGET-3	10.00822	0	7.9925	8.76099	8.45942	10.119	10.7106	9.55924
TARGET-3	10.04658	0	8.6694	9.22909	8.60333	10.6482	10.8851	10.0586
TARGET-3	2.282192	1	8.43161	9.02925	7.41803	10.5115	10.7849	9.28793
TARGET-3	0.90411	1	7.86297	8.87673	7.01538	9.84074	10.8671	9.27539
TARGET-3	1.090411	1	8.31327	9.08086	8.73055	10.2628	11.1919	9.53618
TARGET-3	3.939726	1	8.23738	8.85444	6.07526	9.37173	10.7144	9.3858
TARGET-3	3.865753	0	8.16011	8.94534	9.52266	10.1422	10.8041	10.1526
TARGET-3	1.868493	1	8.38383	9.20608	9.61526	10.3902	10.8329	10.3404
TARGET-3	4.608219	1	8.01202	8.90975	9.96665	9.91962	10.7429	9.31968
TARGET-3	2.273973	1	8.41612	9.08152	9.99207	10.6526	11.368	10.0776

RBM15	ALKBH5	ZC3H13	Risk	riskScore
9.06814	8.6238	7.46999	Low_risk	-0.4023
8.82506	8.93592	8.37419	High_risk	-0.39315
8.87471	8.65295	7.63456	Low_risk	-0.65989
8.36089	9.05572	7.53358	Low_risk	-0.76546
8.87993	8.61564	8.17585	Low_risk	-0.57312
8.59864	8.32456	8.9769	High_risk	0.985772
8.80708	8.4798	7.93324	Low_risk	-0.39562
8.1105	8.60033	6.95288	Low_risk	-1.0757
8.37203	8.7521	7.12939	Low_risk	-0.46024
8.59919	7.93259	8.07144	Low_risk	-0.66318
8.97736	8.43047	7.59385	Low_risk	-0.60264
9.12783	8.41689	7.81222	High_risk	-0.07633
8.31812	8.94746	7.5471	Low_risk	-0.83836
9.24636	8.35478	8.24609	High_risk	0.140672
8.83725	8.61894	6.98454	High_risk	-0.3375
8.64119	8.63279	7.23681	Low_risk	-0.42686
9.05945	9.31484	7.93091	Low_risk	-0.40833
8.69575	8.72131	7.40877	Low_risk	-0.75326
8.85364	8.61304	7.35002	High_risk	-0.09597
8.23949	8.83918	7.6257	High_risk	-0.3626
8.4528	8.87128	7.40871	High_risk	-0.19777
8.69312	8.38982	7.75743	High_risk	-0.1355
8.44641	8.67445	8.28176	Low_risk	-1.07652
9.27074	8.95508	6.3142	High_risk	-0.02409
8.14652	8.82211	6.40741	High_risk	-0.01002
8.25001	8.63773	6.76973	Low_risk	-0.45489
8.29349	8.77648	7.65998	High_risk	-0.36379
8.86889	8.8379	8.28115	Low_risk	-0.73191
8.63242	8.57498	6.63523	Low_risk	-0.76419
8.26327	8.66978	7.61614	Low_risk	-0.81913
8.0946	8.8895	7.73127	Low_risk	-0.43999
8.59048	8.71724	7.57179	High_risk	0.137353
9.16998	8.497	6.70077	High_risk	-0.31164
9.00446	8.76296	8.02353	High_risk	-0.15339
8.30296	8.57823	7.76604	High_risk	-0.39483
8.11999	9.15748	7.89152	Low_risk	-1.03669
8.60615	9.02203	8.24815	High_risk	-0.30488
8.70361	8.60051	7.39717	High_risk	-0.32199
8.52145	8.74075	7.40573	High_risk	-0.17017
8.65305	8.83933	7.17538	High_risk	-0.37928
8.8103	8.3356	8.03243	High_risk	-0.09245
9.00167	8.69432	7.87262	Low_risk	-0.45889
8.58105	8.92628	7.41657	High_risk	0.024431
8.73263	8.53479	8.3437	Low_risk	-0.76639
8.58877	8.49639	7.70645	High_risk	-0.296
8.32559	8.89121	7.88243	Low_risk	-0.83463
8.15155	8.88033	7.6973	High_risk	-0.33533
8.99909	9.11395	7.26638	Low_risk	-0.61772
8.13029	8.19251	6.59079	High_risk	0.202511
9.07262	8.71662	7.45023	Low_risk	-0.44209
9.03856	8.8839	7.26587	Low_risk	-0.43225
8.84765	8.8976	7.55483	Low_risk	-0.65046
8.34551	8.76294	7.55569	High_risk	-0.29084
8.14608	8.53515	7.84822	Low_risk	-1.15454
8.65379	8.72783	7.7327	High_risk	-0.37806
8.47853	8.26245	8.10178	Low_risk	-0.6765
8.87164	7.75379	8.47445	High_risk	0.328419
8.27781	8.9608	7.51936	Low_risk	-0.53223

8.28372	8.64111	6.40068	High_risk	-0.05921
8.1438	8.27803	7.5918	High_risk	-0.29839
8.31702	8.10053	7.80577	High_risk	-0.2176
9.23885	8.70176	7.04633	High_risk	-0.0562
8.41731	8.567	7.31483	Low_risk	-0.61478
8.58989	9.04582	7.84108	High_risk	-0.02189
9.0742	8.34721	7.71637	Low_risk	-0.67616
9.06266	8.83524	7.84189	Low_risk	-0.51322
7.53256	7.89187	6.28706	High_risk	0.162882
8.43367	8.82816	7.73483	Low_risk	-0.77078
8.03423	8.54934	8.20374	Low_risk	-0.61917
8.58627	8.90601	7.41473	Low_risk	-1.16764
8.32152	8.50387	8.14364	Low_risk	-0.53376
9.17134	8.53888	7.92182	High_risk	-0.15754
8.49194	8.36768	8.07678	High_risk	-0.33288
8.86001	8.24664	8.08062	High_risk	0.283509
8.37907	8.32026	7.2587	Low_risk	-0.90037
8.75855	8.51753	7.32993	Low_risk	-0.43969
9.04274	8.38237	7.64013	High_risk	-0.00234
8.53825	8.78127	7.91604	High_risk	0.062093
8.45851	9.06549	7.1638	Low_risk	-1.19765
8.78767	8.54056	8.25708	High_risk	-0.24101
9.10288	8.82236	8.29901	High_risk	-0.13741
8.96732	8.11319	7.92676	High_risk	0.087722
8.6707	8.60942	7.13599	Low_risk	-1.02976
8.65091	8.85251	8.59437	Low_risk	-1.04484
9.24913	8.56829	7.28712	Low_risk	-0.40303
8.42556	8.87848	6.96182	Low_risk	-0.98752
8.6556	9.02163	7.30396	Low_risk	-0.47406
8.49532	8.60752	8.30273	Low_risk	-0.73585
8.69545	9.16948	7.78356	Low_risk	-0.67488
9.02246	8.18868	7.74335	High_risk	-0.38514
8.64903	8.52691	7.44818	Low_risk	-0.53309
8.58178	8.3134	7.40319	High_risk	0.329584
8.15625	8.48944	7.75362	High_risk	-0.21697
8.58832	8.70859	7.3845	High_risk	-0.04597
9.01249	8.41958	7.01561	High_risk	-0.36889
8.59745	8.52027	8.57465	Low_risk	-1.27816
9.0744	8.70223	7.29388	Low_risk	-0.61616
9.36875	8.5419	8.23506	High_risk	-0.15128
9.46356	8.15024	8.60089	High_risk	0.035439
8.38952	8.54264	7.58598	Low_risk	-0.4139
8.18302	9.45516	8.23851	Low_risk	-0.85333
8.80484	8.93031	7.12751	High_risk	0.140986
8.19239	9.56486	7.84585	Low_risk	-0.69494
8.8444	9.29278	8.53997	Low_risk	-0.57313
8.97412	9.58921	8.20522	Low_risk	-0.40256
8.87284	8.90249	8.19994	Low_risk	-0.88921
9.22883	8.94396	8.23652	Low_risk	-0.79102
8.68654	9.11564	7.79123	Low_risk	-1.00426
8.0869	8.62206	7.51526	High_risk	-0.33002
7.96079	9.21359	7.27256	Low_risk	-1.06163
8.50509	9.27595	7.91343	Low_risk	-1.44198
9.22678	9.08154	8.26885	High_risk	-0.08119
8.28555	8.7788	8.27206	Low_risk	-1.15875
8.57122	9.05411	7.463	Low_risk	-0.82893
8.78069	8.78537	8.40717	Low_risk	-0.585
8.94283	8.71792	7.56385	Low_risk	-0.45253
8.09067	8.92321	7.55473	High_risk	-0.36972

8.08036	9.17749	8.31911	Low_risk	-0.76587
8.24336	8.62025	7.90391	High_risk	-0.25826
8.70182	9.01851	7.72287	High_risk	-0.39286
8.88704	8.97442	8.34512	Low_risk	-0.72884
7.91369	8.82483	8.70347	Low_risk	-0.71664
7.98451	9.13739	6.56331	Low_risk	-0.5327
7.66844	9.79465	7.79854	Low_risk	-1.23586
8.34739	9.10336	8.12157	Low_risk	-0.79591
9.01594	8.77135	7.8276	High_risk	-0.04839
8.35617	9.52713	8.22797	Low_risk	-0.57013
8.19389	8.76836	8.13818	Low_risk	-0.69604
8.83685	8.98408	8.13013	Low_risk	-0.74147
9.14056	8.61685	7.45058	High_risk	-0.2369
7.97192	10.1511	8.18475	Low_risk	-1.32693
8.48585	8.85403	7.1959	Low_risk	-0.42343
8.65807	9.1067	7.54219	Low_risk	-0.64336
8.1029	8.94232	7.66242	Low_risk	-0.68971
8.62897	8.97475	7.49041	Low_risk	-0.72316
8.94205	8.84396	8.43436	Low_risk	-0.43349
7.76093	9.52681	7.4898	Low_risk	-0.75316
8.51722	9.37842	8.21549	Low_risk	-0.49762
8.76398	9.02442	7.8668	High_risk	-0.39065
8.78034	8.9458	7.81996	High_risk	-0.21816
8.17264	8.54885	7.97597	Low_risk	-0.66001
7.99615	8.70032	7.98342	Low_risk	-0.87103
8.54064	8.96416	7.94522	Low_risk	-0.97513
8.91649	8.19777	7.81121	High_risk	0.128314
8.30526	9.05177	8.42762	Low_risk	-0.64901
8.17829	8.94629	7.67756	Low_risk	-0.73505
8.90453	9.23782	7.97727	High_risk	-0.35508
9.28986	8.99112	7.02972	High_risk	-0.00183
8.7902	8.51373	7.79963	High_risk	-0.04836
8.67878	9.0562	7.83725	Low_risk	-0.55723
8.0989	8.7742	7.24851	Low_risk	-0.64291
9.04564	8.76822	6.8158	High_risk	-0.02369
8.99053	8.93723	8.22673	High_risk	-0.28875
8.75957	8.248	7.6277	High_risk	0.034392
9.62235	9.09318	7.12921	Low_risk	-0.64906
8.31682	9.06323	7.50467	High_risk	-0.31314
8.46165	9.13767	7.28109	Low_risk	-0.55069
9.27065	9.14962	6.20044	Low_risk	-0.5724
9.37407	9.04988	7.79738	High_risk	-0.16907
8.26362	8.98633	7.50938	Low_risk	-0.59533
9.15765	8.70035	8.3514	High_risk	0.170077
8.93297	8.93119	7.42989	High_risk	-0.25122
8.82455	9.14678	8.06746	Low_risk	-0.59596
8.23636	8.44431	8.29604	High_risk	0.036315
8.69908	8.8611	8.44067	Low_risk	-0.40818
9.08628	9.26529	8.67077	Low_risk	-0.73738
8.6661	8.24036	7.18747	High_risk	-0.3126
8.34284	8.36953	7.1805	Low_risk	-0.71263
8.09978	8.20836	6.64509	High_risk	-0.22033
8.76263	8.27958	8.01451	High_risk	0.161216
9.17802	8.44597	7.81867	High_risk	-0.32257
8.87762	8.82017	7.7501	High_risk	-0.24612
7.8822	8.47802	7.11977	Low_risk	-0.56107
8.56616	8.2359	7.96477	High_risk	0.040221
8.66517	8.34913	7.42899	High_risk	-0.08499
8.32533	8.74618	7.16607	High_risk	-0.37457

8.937	8.44688	7.90084	Low_risk	-0.47642
8.56866	8.24801	7.17046	High_risk	-0.15547
8.68939	8.66507	7.52035	High_risk	-0.05175
7.92558	8.53384	7.2956	High_risk	-0.30553
8.37842	8.60343	7.75528	Low_risk	-0.67469
8.35928	8.88446	7.70603	High_risk	0.09413
8.85919	9.26259	7.63034	Low_risk	-1.04774
8.37949	8.60765	6.58422	Low_risk	-0.85032
8.49624	8.64315	8.0073	High_risk	0.014079
8.42589	8.5879	7.27729	Low_risk	-0.54728
8.49266	8.4747	7.73648	Low_risk	-0.41676
8.52976	8.14154	6.32793	High_risk	0.225487
8.69308	8.75583	7.51057	High_risk	0.058576
8.89806	8.14944	7.84009	High_risk	0.501311
9.01027	8.55326	7.09956	High_risk	-0.06231
8.97873	8.63354	7.12816	High_risk	-0.09643
8.27117	8.75696	6.81588	Low_risk	-0.50321
8.02848	8.99904	7.41752	Low_risk	-0.73359
8.58498	8.83198	8.1165	High_risk	0.150616
7.84997	8.931	7.42058	Low_risk	-1.17455
8.27725	8.39284	7.46284	High_risk	0.094797
8.54568	8.22018	7.13849	High_risk	-0.37545
9.10939	8.40325	7.1668	High_risk	-0.00462
8.41641	8.44814	7.58042	High_risk	-0.29562
8.74688	8.19827	7.08739	High_risk	-0.06077
8.86509	8.45765	7.44202	High_risk	0.033002
8.82639	8.34946	7.2579	High_risk	-0.24833
9.23705	8.16802	7.60225	High_risk	-0.02118
8.2069	8.36853	7.69076	High_risk	0.083401
9.20094	8.71387	7.20616	High_risk	-0.07903
8.54864	8.3779	7.62223	Low_risk	-0.52684
8.76722	8.34351	7.60788	High_risk	0.136275
8.18138	8.11494	7.33074	High_risk	-0.10524
8.49163	8.48617	7.55995	Low_risk	-0.51981
8.63838	8.43166	8.21734	High_risk	-0.26435
8.72478	8.52885	8.04524	Low_risk	-0.74482
8.55231	8.35212	6.83352	Low_risk	-0.53879
8.07762	8.587	7.25971	Low_risk	-0.75807
7.93107	8.84332	6.86384	Low_risk	-0.73563
8.10294	9.02433	7.58906	Low_risk	-0.72337
9.13415	8.67569	7.2815	High_risk	-0.39205
8.41557	8.743	7.57985	Low_risk	-0.6807
8.81046	8.16316	7.67162	High_risk	-0.38067
7.51015	8.74967	7.28065	Low_risk	-0.76944
8.45569	8.3819	6.5057	High_risk	-0.03194
8.87899	8.61782	6.96226	Low_risk	-1.13407
9.43111	8.51093	7.39386	High_risk	0.32721
7.96688	9.01294	7.38733	Low_risk	-0.87139
8.34782	8.34665	7.93521	High_risk	-0.38841
8.32163	8.11827	7.09814	High_risk	-0.06869
8.56992	8.10649	7.58929	Low_risk	-0.4635
8.73966	8.38898	7.06814	High_risk	-0.01295
8.52175	8.39399	6.67018	Low_risk	-0.49742
8.60507	8.02894	7.15158	Low_risk	-0.50392
7.96511	8.52923	7.55462	Low_risk	-0.71234
8.68015	8.33017	7.55313	High_risk	-0.15543
9.03491	8.17988	7.42736	High_risk	0.059199
8.60186	8.32794	7.62075	High_risk	0.013211
8.86844	8.11245	7.36921	High_risk	-0.18959

8.58564	8.3101	6.91585	Low_risk	-0.51863
8.7272	8.45505	7.51394	High_risk	-0.23433
8.73749	8.4456	7.43745	High_risk	-0.25767
9.04078	8.20111	7.79621	High_risk	0.094957
8.71027	8.11371	7.18378	Low_risk	-0.48167
8.57518	8.34141	7.36778	High_risk	-0.38831
8.84022	8.39895	8.02774	High_risk	-0.24202
8.52167	8.62871	7.462	High_risk	0.032325
9.54746	8.28936	7.78017	High_risk	0.446543
9.23225	8.32868	8.15714	High_risk	0.318218
8.71417	8.65233	7.74073	Low_risk	-0.54584
9.35579	8.32589	8.36026	High_risk	0.195431

Supplementary Table 4

rownames	Type	values	times	status
TARGET-3	G1(n=31)	7.3033	NA	NA
TARGET-3	G1(n=31)	7.86023	NA	NA
TARGET-3	G1(n=31)	7.36593	NA	NA
TARGET-3	G1(n=31)	7.79965	NA	NA
TARGET-3	G1(n=31)	7.2822	NA	NA
TARGET-3	G1(n=31)	7.76037	NA	NA
TARGET-3	G1(n=31)	8.17601	NA	NA
TARGET-3	G1(n=31)	7.88618	NA	NA
TARGET-3	G1(n=31)	7.26121	NA	NA
TARGET-3	G1(n=31)	7.76633	NA	NA
TARGET-3	G1(n=31)	7.94033	NA	NA
TARGET-3	G1(n=31)	8.42465	NA	NA
TARGET-3	G1(n=31)	7.35317	NA	NA
TARGET-3	G1(n=31)	7.46327	NA	NA
TARGET-3	G1(n=31)	7.77668	NA	NA
TARGET-3	G1(n=31)	7.45216	NA	NA
TARGET-3	G1(n=31)	7.0377	NA	NA
TARGET-3	G1(n=31)	7.78143	NA	NA
TARGET-3	G1(n=31)	7.14799	NA	NA
TARGET-3	G1(n=31)	7.64017	NA	NA
TARGET-3	G1(n=31)	7.1994	NA	NA
TARGET-3	G1(n=31)	8.03172	NA	NA
TARGET-3	G1(n=31)	7.96317	NA	NA
TARGET-3	G1(n=31)	7.77198	NA	NA
TARGET-3	G1(n=31)	8.07611	NA	NA
TARGET-3	G1(n=31)	7.51091	NA	NA
TARGET-3	G1(n=31)	7.74478	NA	NA
TARGET-3	G1(n=31)	7.36118	NA	NA
TARGET-3	G1(n=31)	8.08029	NA	NA
TARGET-3	G1(n=31)	7.44297	NA	NA
TARGET-3	G1(n=31)	8.16011	NA	NA
TARGET-3	G2(n=213	7.93695	NA	NA
TARGET-3	G2(n=213	7.78644	NA	NA
TARGET-3	G2(n=213	8.48396	NA	NA
TARGET-3	G2(n=213	7.85604	NA	NA
TARGET-3	G2(n=213	8.805	NA	NA
TARGET-3	G2(n=213	7.88024	NA	NA
TARGET-3	G2(n=213	8.30731	NA	NA
TARGET-3	G2(n=213	7.39381	NA	NA
TARGET-3	G2(n=213	7.41424	NA	NA
TARGET-3	G2(n=213	8.31635	NA	NA
TARGET-3	G2(n=213	8.05388	NA	NA
TARGET-3	G2(n=213	7.99366	NA	NA
TARGET-3	G2(n=213	7.99528	NA	NA
TARGET-3	G2(n=213	7.6401	NA	NA
TARGET-3	G2(n=213	8.02303	NA	NA
TARGET-3	G2(n=213	8.0571	NA	NA
TARGET-3	G2(n=213	8.59227	NA	NA
TARGET-3	G2(n=213	7.9189	NA	NA
TARGET-3	G2(n=213	7.61279	NA	NA
TARGET-3	G2(n=213	8.59269	NA	NA
TARGET-3	G2(n=213	8.05916	NA	NA
TARGET-3	G2(n=213	7.43599	NA	NA
TARGET-3	G2(n=213	7.42422	NA	NA
TARGET-3	G2(n=213	8.24315	NA	NA
TARGET-3	G2(n=213	7.77141	NA	NA
TARGET-3	G2(n=213	8.14646	NA	NA
TARGET-3	G2(n=213	7.53679	NA	NA

TARGET-3	G2(n=213	8.42711	NA	NA
TARGET-3	G2(n=213	7.39985	NA	NA
TARGET-3	G2(n=213	7.43276	NA	NA
TARGET-3	G2(n=213	8.01931	NA	NA
TARGET-3	G2(n=213	8.1642	NA	NA
TARGET-3	G2(n=213	7.73073	NA	NA
TARGET-3	G2(n=213	7.56174	NA	NA
TARGET-3	G2(n=213	8.27069	NA	NA
TARGET-3	G2(n=213	7.91549	NA	NA
TARGET-3	G2(n=213	8.23907	NA	NA
TARGET-3	G2(n=213	7.52105	NA	NA
TARGET-3	G2(n=213	8.13648	NA	NA
TARGET-3	G2(n=213	8.01798	NA	NA
TARGET-3	G2(n=213	8.2181	NA	NA
TARGET-3	G2(n=213	7.66111	NA	NA
TARGET-3	G2(n=213	8.33747	NA	NA
TARGET-3	G2(n=213	8.14777	NA	NA
TARGET-3	G2(n=213	7.94385	NA	NA
TARGET-3	G2(n=213	8.05025	NA	NA
TARGET-3	G2(n=213	7.57717	NA	NA
TARGET-3	G2(n=213	8.30145	NA	NA
TARGET-3	G2(n=213	7.36958	NA	NA
TARGET-3	G2(n=213	7.98455	NA	NA
TARGET-3	G2(n=213	7.43371	NA	NA
TARGET-3	G2(n=213	8.25778	NA	NA
TARGET-3	G2(n=213	8.05518	NA	NA
TARGET-3	G2(n=213	6.97472	NA	NA
TARGET-3	G2(n=213	8.08507	NA	NA
TARGET-3	G2(n=213	7.42028	NA	NA
TARGET-3	G2(n=213	7.41019	NA	NA
TARGET-3	G2(n=213	8.56412	NA	NA
TARGET-3	G2(n=213	8.10993	NA	NA
TARGET-3	G2(n=213	6.07873	NA	NA
TARGET-3	G2(n=213	8.19229	NA	NA
TARGET-3	G2(n=213	7.7986	NA	NA
TARGET-3	G2(n=213	8.02696	NA	NA
TARGET-3	G2(n=213	8.40829	NA	NA
TARGET-3	G2(n=213	8.5769	NA	NA
TARGET-3	G2(n=213	7.79179	NA	NA
TARGET-3	G2(n=213	7.00228	NA	NA
TARGET-3	G2(n=213	8.30711	NA	NA
TARGET-3	G2(n=213	8.04851	NA	NA
TARGET-3	G2(n=213	8.11829	NA	NA
TARGET-3	G2(n=213	7.91723	NA	NA
TARGET-3	G2(n=213	8.38271	NA	NA
TARGET-3	G2(n=213	8.2504	NA	NA
TARGET-3	G2(n=213	7.98046	NA	NA
TARGET-3	G2(n=213	7.87003	NA	NA
TARGET-3	G2(n=213	7.73226	NA	NA
TARGET-3	G2(n=213	8.0263	NA	NA
TARGET-3	G2(n=213	7.7747	NA	NA
TARGET-3	G2(n=213	8.35247	NA	NA
TARGET-3	G2(n=213	7.5172	NA	NA
TARGET-3	G2(n=213	8.37847	NA	NA
TARGET-3	G2(n=213	7.78422	NA	NA
TARGET-3	G2(n=213	8.55678	NA	NA
TARGET-3	G2(n=213	7.02268	NA	NA
TARGET-3	G2(n=213	7.46975	NA	NA
TARGET-3	G2(n=213	7.95655	NA	NA

TARGET-3	G2(n=213	8.15103	NA	NA
TARGET-3	G2(n=213	7.77435	NA	NA
TARGET-3	G2(n=213	6.99383	NA	NA
TARGET-3	G2(n=213	7.8815	NA	NA
TARGET-3	G2(n=213	8.82909	NA	NA
TARGET-3	G2(n=213	7.94388	NA	NA
TARGET-3	G2(n=213	8.12314	NA	NA
TARGET-3	G2(n=213	8.01022	NA	NA
TARGET-3	G2(n=213	8.44992	NA	NA
TARGET-3	G2(n=213	7.83362	NA	NA
TARGET-3	G2(n=213	6.98459	NA	NA
TARGET-3	G2(n=213	8.84062	NA	NA
TARGET-3	G2(n=213	8.11066	NA	NA
TARGET-3	G2(n=213	8.04093	NA	NA
TARGET-3	G2(n=213	7.24902	NA	NA
TARGET-3	G2(n=213	7.85587	NA	NA
TARGET-3	G2(n=213	8.48692	NA	NA
TARGET-3	G2(n=213	8.12883	NA	NA
TARGET-3	G2(n=213	8.23291	NA	NA
TARGET-3	G2(n=213	7.6444	NA	NA
TARGET-3	G2(n=213	7.73449	NA	NA
TARGET-3	G2(n=213	8.34898	NA	NA
TARGET-3	G2(n=213	7.08764	NA	NA
TARGET-3	G2(n=213	8.49741	NA	NA
TARGET-3	G2(n=213	8.40893	NA	NA
TARGET-3	G2(n=213	7.5937	NA	NA
TARGET-3	G2(n=213	8.25531	NA	NA
TARGET-3	G2(n=213	8.32992	NA	NA
TARGET-3	G2(n=213	7.93784	NA	NA
TARGET-3	G2(n=213	7.46728	NA	NA
TARGET-3	G2(n=213	7.85844	NA	NA
TARGET-3	G2(n=213	8.28727	NA	NA
TARGET-3	G2(n=213	7.81883	NA	NA
TARGET-3	G2(n=213	8.06527	NA	NA
TARGET-3	G2(n=213	8.12781	NA	NA
TARGET-3	G2(n=213	7.94284	NA	NA
TARGET-3	G2(n=213	8.00981	NA	NA
TARGET-3	G2(n=213	7.63915	NA	NA
TARGET-3	G2(n=213	8.19444	NA	NA
TARGET-3	G2(n=213	8.35266	NA	NA
TARGET-3	G2(n=213	8.25538	NA	NA
TARGET-3	G2(n=213	8.28049	NA	NA
TARGET-3	G2(n=213	7.34179	NA	NA
TARGET-3	G2(n=213	8.34572	NA	NA
TARGET-3	G2(n=213	8.23015	NA	NA
TARGET-3	G2(n=213	8.25893	NA	NA
TARGET-3	G2(n=213	7.71163	NA	NA
TARGET-3	G2(n=213	7.47074	NA	NA
TARGET-3	G2(n=213	7.3255	NA	NA
TARGET-3	G2(n=213	7.8985	NA	NA
TARGET-3	G2(n=213	7.3531	NA	NA
TARGET-3	G2(n=213	7.4123	NA	NA
TARGET-3	G2(n=213	7.29317	NA	NA
TARGET-3	G2(n=213	7.50362	NA	NA
TARGET-3	G2(n=213	8.40479	NA	NA
TARGET-3	G2(n=213	7.52104	NA	NA
TARGET-3	G2(n=213	7.50386	NA	NA
TARGET-3	G2(n=213	8.869	NA	NA
TARGET-3	G2(n=213	7.89437	NA	NA

TARGET-3	G2(n=213	7.26709	NA	NA
TARGET-3	G2(n=213	8.16592	NA	NA
TARGET-3	G2(n=213	7.76126	NA	NA
TARGET-3	G2(n=213	8.25054	NA	NA
TARGET-3	G2(n=213	7.76178	NA	NA
TARGET-3	G2(n=213	8.16947	NA	NA
TARGET-3	G2(n=213	8.55402	NA	NA
TARGET-3	G2(n=213	8.07696	NA	NA
TARGET-3	G2(n=213	7.5726	NA	NA
TARGET-3	G2(n=213	7.91519	NA	NA
TARGET-3	G2(n=213	7.88826	NA	NA
TARGET-3	G2(n=213	8.09805	NA	NA
TARGET-3	G2(n=213	7.10663	NA	NA
TARGET-3	G2(n=213	8.25877	NA	NA
TARGET-3	G2(n=213	8.60989	NA	NA
TARGET-3	G2(n=213	8.25564	NA	NA
TARGET-3	G2(n=213	8.01204	NA	NA
TARGET-3	G2(n=213	7.39434	NA	NA
TARGET-3	G2(n=213	7.78095	NA	NA
TARGET-3	G2(n=213	8.21045	NA	NA
TARGET-3	G2(n=213	8.13282	NA	NA
TARGET-3	G2(n=213	8.11901	NA	NA
TARGET-3	G2(n=213	8.10323	NA	NA
TARGET-3	G2(n=213	8.65283	NA	NA
TARGET-3	G2(n=213	7.19466	NA	NA
TARGET-3	G2(n=213	7.61585	NA	NA
TARGET-3	G2(n=213	8.35549	NA	NA
TARGET-3	G2(n=213	8.26073	NA	NA
TARGET-3	G2(n=213	8.53678	NA	NA
TARGET-3	G2(n=213	8.91843	NA	NA
TARGET-3	G2(n=213	8.16116	NA	NA
TARGET-3	G2(n=213	8.25725	NA	NA
TARGET-3	G2(n=213	8.77459	NA	NA
TARGET-3	G2(n=213	7.2176	NA	NA
TARGET-3	G2(n=213	8.44909	NA	NA
TARGET-3	G2(n=213	8.13081	NA	NA
TARGET-3	G2(n=213	8.18962	NA	NA
TARGET-3	G2(n=213	7.68844	NA	NA
TARGET-3	G2(n=213	7.3619	NA	NA
TARGET-3	G2(n=213	6.87112	NA	NA
TARGET-3	G2(n=213	7.32842	NA	NA
TARGET-3	G2(n=213	8.15119	NA	NA
TARGET-3	G2(n=213	7.96473	NA	NA
TARGET-3	G2(n=213	7.96038	NA	NA
TARGET-3	G2(n=213	7.19984	NA	NA
TARGET-3	G2(n=213	7.07745	NA	NA
TARGET-3	G2(n=213	7.94934	NA	NA
TARGET-3	G2(n=213	8.35173	NA	NA
TARGET-3	G2(n=213	8.24429	NA	NA
TARGET-3	G2(n=213	7.53384	NA	NA
TARGET-3	G2(n=213	7.60035	NA	NA
TARGET-3	G2(n=213	7.17324	NA	NA
TARGET-3	G2(n=213	8.1187	NA	NA
TARGET-3	G2(n=213	7.06729	NA	NA
TARGET-3	G2(n=213	7.77643	NA	NA
TARGET-3	G2(n=213	7.03863	NA	NA
TARGET-3	G2(n=213	7.51336	NA	NA
TARGET-3	G2(n=213	7.87299	NA	NA
TARGET-3	G2(n=213	7.22368	NA	NA

TARGET-3	G2(n=213	7.40854	NA	NA
TARGET-3	G2(n=213	7.93709	NA	NA
TARGET-3	G2(n=213	8.0821	NA	NA
TARGET-3	G2(n=213	7.9925	NA	NA
TARGET-3	G2(n=213	8.6694	NA	NA
TARGET-3	G2(n=213	8.43161	NA	NA
TARGET-3	G2(n=213	8.31327	NA	NA
TARGET-3	G2(n=213	8.38383	NA	NA
TARGET-3	G2(n=213	8.41612	NA	NA

Supplementary Table 5

rownames	Type	values	times	status
TARGET-3	G1(n=72)	7.99528	NA	NA
TARGET-3	G1(n=72)	8.02303	NA	NA
TARGET-3	G1(n=72)	8.0571	NA	NA
TARGET-3	G1(n=72)	8.59227	NA	NA
TARGET-3	G1(n=72)	7.61279	NA	NA
TARGET-3	G1(n=72)	7.42422	NA	NA
TARGET-3	G1(n=72)	8.24315	NA	NA
TARGET-3	G1(n=72)	8.14646	NA	NA
TARGET-3	G1(n=72)	7.43276	NA	NA
TARGET-3	G1(n=72)	7.73073	NA	NA
TARGET-3	G1(n=72)	7.91549	NA	NA
TARGET-3	G1(n=72)	7.94385	NA	NA
TARGET-3	G1(n=72)	8.05025	NA	NA
TARGET-3	G1(n=72)	8.30145	NA	NA
TARGET-3	G1(n=72)	8.05518	NA	NA
TARGET-3	G1(n=72)	6.97472	NA	NA
TARGET-3	G1(n=72)	7.41019	NA	NA
TARGET-3	G1(n=72)	8.56412	NA	NA
TARGET-3	G1(n=72)	7.3033	NA	NA
TARGET-3	G1(n=72)	8.38271	NA	NA
TARGET-3	G1(n=72)	7.98046	NA	NA
TARGET-3	G1(n=72)	8.0263	NA	NA
TARGET-3	G1(n=72)	7.78422	NA	NA
TARGET-3	G1(n=72)	6.99383	NA	NA
TARGET-3	G1(n=72)	7.94388	NA	NA
TARGET-3	G1(n=72)	8.12314	NA	NA
TARGET-3	G1(n=72)	7.88618	NA	NA
TARGET-3	G1(n=72)	8.11066	NA	NA
TARGET-3	G1(n=72)	7.26121	NA	NA
TARGET-3	G1(n=72)	7.85587	NA	NA
TARGET-3	G1(n=72)	8.12883	NA	NA
TARGET-3	G1(n=72)	7.76633	NA	NA
TARGET-3	G1(n=72)	7.94033	NA	NA
TARGET-3	G1(n=72)	8.42465	NA	NA
TARGET-3	G1(n=72)	7.35317	NA	NA
TARGET-3	G1(n=72)	7.46327	NA	NA
TARGET-3	G1(n=72)	7.77668	NA	NA
TARGET-3	G1(n=72)	7.45216	NA	NA
TARGET-3	G1(n=72)	8.40893	NA	NA
TARGET-3	G1(n=72)	7.0377	NA	NA
TARGET-3	G1(n=72)	7.78143	NA	NA
TARGET-3	G1(n=72)	7.5937	NA	NA
TARGET-3	G1(n=72)	8.25531	NA	NA
TARGET-3	G1(n=72)	7.46728	NA	NA
TARGET-3	G1(n=72)	7.14799	NA	NA
TARGET-3	G1(n=72)	7.64017	NA	NA
TARGET-3	G1(n=72)	7.1994	NA	NA
TARGET-3	G1(n=72)	8.03172	NA	NA
TARGET-3	G1(n=72)	7.96317	NA	NA
TARGET-3	G1(n=72)	8.28727	NA	NA
TARGET-3	G1(n=72)	7.77198	NA	NA
TARGET-3	G1(n=72)	8.07611	NA	NA
TARGET-3	G1(n=72)	7.74478	NA	NA
TARGET-3	G1(n=72)	7.36118	NA	NA
TARGET-3	G1(n=72)	8.08029	NA	NA
TARGET-3	G1(n=72)	8.25893	NA	NA
TARGET-3	G1(n=72)	7.44297	NA	NA
TARGET-3	G1(n=72)	7.4123	NA	NA

TARGET-3	G1(n=72)	7.50386	NA	NA
TARGET-3	G1(n=72)	8.869	NA	NA
TARGET-3	G1(n=72)	7.26709	NA	NA
TARGET-3	G1(n=72)	7.10663	NA	NA
TARGET-3	G1(n=72)	7.68844	NA	NA
TARGET-3	G1(n=72)	7.32842	NA	NA
TARGET-3	G1(n=72)	8.15119	NA	NA
TARGET-3	G1(n=72)	7.94934	NA	NA
TARGET-3	G1(n=72)	7.60035	NA	NA
TARGET-3	G1(n=72)	7.03863	NA	NA
TARGET-3	G1(n=72)	7.22368	NA	NA
TARGET-3	G1(n=72)	7.86297	NA	NA
TARGET-3	G1(n=72)	8.23738	NA	NA
TARGET-3	G1(n=72)	8.01202	NA	NA
TARGET-3	G2(n=54)	7.6401	NA	NA
TARGET-3	G2(n=54)	7.43599	NA	NA
TARGET-3	G2(n=54)	7.53679	NA	NA
TARGET-3	G2(n=54)	8.42711	NA	NA
TARGET-3	G2(n=54)	7.56174	NA	NA
TARGET-3	G2(n=54)	8.27069	NA	NA
TARGET-3	G2(n=54)	8.01798	NA	NA
TARGET-3	G2(n=54)	8.2181	NA	NA
TARGET-3	G2(n=54)	7.66111	NA	NA
TARGET-3	G2(n=54)	8.14777	NA	NA
TARGET-3	G2(n=54)	7.57717	NA	NA
TARGET-3	G2(n=54)	7.98455	NA	NA
TARGET-3	G2(n=54)	7.43371	NA	NA
TARGET-3	G2(n=54)	8.25778	NA	NA
TARGET-3	G2(n=54)	8.10993	NA	NA
TARGET-3	G2(n=54)	7.87003	NA	NA
TARGET-3	G2(n=54)	8.35247	NA	NA
TARGET-3	G2(n=54)	7.86023	NA	NA
TARGET-3	G2(n=54)	7.36593	NA	NA
TARGET-3	G2(n=54)	7.79965	NA	NA
TARGET-3	G2(n=54)	8.23291	NA	NA
TARGET-3	G2(n=54)	7.73449	NA	NA
TARGET-3	G2(n=54)	8.32992	NA	NA
TARGET-3	G2(n=54)	7.85844	NA	NA
TARGET-3	G2(n=54)	7.51091	NA	NA
TARGET-3	G2(n=54)	7.94284	NA	NA
TARGET-3	G2(n=54)	8.00981	NA	NA
TARGET-3	G2(n=54)	8.19444	NA	NA
TARGET-3	G2(n=54)	7.71163	NA	NA
TARGET-3	G2(n=54)	7.47074	NA	NA
TARGET-3	G2(n=54)	7.3255	NA	NA
TARGET-3	G2(n=54)	7.3531	NA	NA
TARGET-3	G2(n=54)	7.50362	NA	NA
TARGET-3	G2(n=54)	8.40479	NA	NA
TARGET-3	G2(n=54)	7.52104	NA	NA
TARGET-3	G2(n=54)	8.16592	NA	NA
TARGET-3	G2(n=54)	7.76126	NA	NA
TARGET-3	G2(n=54)	8.55402	NA	NA
TARGET-3	G2(n=54)	7.88826	NA	NA
TARGET-3	G2(n=54)	8.25564	NA	NA
TARGET-3	G2(n=54)	8.01204	NA	NA
TARGET-3	G2(n=54)	8.11901	NA	NA
TARGET-3	G2(n=54)	8.26073	NA	NA
TARGET-3	G2(n=54)	8.25725	NA	NA
TARGET-3	G2(n=54)	8.13081	NA	NA

TARGET-3	G2(n=54)	7.96473	NA	NA
TARGET-3	G2(n=54)	7.17324	NA	NA
TARGET-3	G2(n=54)	7.06729	NA	NA
TARGET-3	G2(n=54)	7.77643	NA	NA
TARGET-3	G2(n=54)	7.40854	NA	NA
TARGET-3	G2(n=54)	7.93709	NA	NA
TARGET-3	G2(n=54)	8.0821	NA	NA
TARGET-3	G2(n=54)	8.6694	NA	NA
TARGET-3	G2(n=54)	8.16011	NA	NA
TARGET-3	G3(n=49)	7.99366	NA	NA
TARGET-3	G3(n=49)	8.59269	NA	NA
TARGET-3	G3(n=49)	8.05916	NA	NA
TARGET-3	G3(n=49)	7.77141	NA	NA
TARGET-3	G3(n=49)	7.39985	NA	NA
TARGET-3	G3(n=49)	8.1642	NA	NA
TARGET-3	G3(n=49)	8.23907	NA	NA
TARGET-3	G3(n=49)	8.13648	NA	NA
TARGET-3	G3(n=49)	8.33747	NA	NA
TARGET-3	G3(n=49)	7.36958	NA	NA
TARGET-3	G3(n=49)	8.08507	NA	NA
TARGET-3	G3(n=49)	8.2504	NA	NA
TARGET-3	G3(n=49)	7.5172	NA	NA
TARGET-3	G3(n=49)	8.37847	NA	NA
TARGET-3	G3(n=49)	8.55678	NA	NA
TARGET-3	G3(n=49)	8.82909	NA	NA
TARGET-3	G3(n=49)	8.04093	NA	NA
TARGET-3	G3(n=49)	8.48692	NA	NA
TARGET-3	G3(n=49)	7.6444	NA	NA
TARGET-3	G3(n=49)	8.34898	NA	NA
TARGET-3	G3(n=49)	7.08764	NA	NA
TARGET-3	G3(n=49)	7.93784	NA	NA
TARGET-3	G3(n=49)	7.81883	NA	NA
TARGET-3	G3(n=49)	8.12781	NA	NA
TARGET-3	G3(n=49)	7.63915	NA	NA
TARGET-3	G3(n=49)	8.35266	NA	NA
TARGET-3	G3(n=49)	8.25538	NA	NA
TARGET-3	G3(n=49)	8.23015	NA	NA
TARGET-3	G3(n=49)	7.29317	NA	NA
TARGET-3	G3(n=49)	7.89437	NA	NA
TARGET-3	G3(n=49)	8.25054	NA	NA
TARGET-3	G3(n=49)	7.5726	NA	NA
TARGET-3	G3(n=49)	7.78095	NA	NA
TARGET-3	G3(n=49)	8.21045	NA	NA
TARGET-3	G3(n=49)	8.10323	NA	NA
TARGET-3	G3(n=49)	8.65283	NA	NA
TARGET-3	G3(n=49)	7.19466	NA	NA
TARGET-3	G3(n=49)	8.91843	NA	NA
TARGET-3	G3(n=49)	8.44909	NA	NA
TARGET-3	G3(n=49)	7.3619	NA	NA
TARGET-3	G3(n=49)	7.96038	NA	NA
TARGET-3	G3(n=49)	8.35173	NA	NA
TARGET-3	G3(n=49)	7.53384	NA	NA
TARGET-3	G3(n=49)	8.1187	NA	NA
TARGET-3	G3(n=49)	7.87299	NA	NA
TARGET-3	G3(n=49)	7.9925	NA	NA
TARGET-3	G3(n=49)	8.43161	NA	NA
TARGET-3	G3(n=49)	8.38383	NA	NA
TARGET-3	G3(n=49)	8.41612	NA	NA

Supplementary Table 6

rownames	Type	values
GSM11084	G1(n=149	10.88683
GSM11084	G1(n=149	11.37748
GSM11084	G1(n=149	11.56596
GSM11084	G1(n=149	11.65323
GSM11084	G1(n=149	11.36871
GSM11084	G1(n=149	11.01975
GSM11084	G1(n=149	11.02882
GSM11084	G1(n=149	10.93507
GSM11084	G1(n=149	11.18782
GSM11084	G1(n=149	10.92972
GSM11084	G1(n=149	10.68483
GSM11084	G1(n=149	11.81629
GSM11084	G1(n=149	11.21791
GSM11084	G1(n=149	11.40163
GSM11084	G1(n=149	11.57672
GSM11085	G1(n=149	11.45421
GSM11085	G1(n=149	11.27436
GSM11085	G1(n=149	11.72647
GSM11085	G1(n=149	11.09494
GSM11085	G1(n=149	11.70289
GSM11085	G1(n=149	11.40073
GSM11085	G1(n=149	11.12059
GSM11085	G1(n=149	10.91367
GSM11085	G1(n=149	11.16431
GSM11085	G1(n=149	11.33961
GSM11085	G1(n=149	10.98529
GSM11085	G1(n=149	11.1962
GSM11085	G1(n=149	11.06617
GSM11085	G1(n=149	11.35334
GSM11085	G1(n=149	11.39886
GSM11085	G1(n=149	10.90615
GSM11085	G1(n=149	11.00561
GSM11085	G1(n=149	11.13305
GSM11085	G1(n=149	11.303
GSM11085	G1(n=149	11.09135
GSM11085	G1(n=149	11.35104
GSM11086	G1(n=149	11.30932
GSM11086	G1(n=149	11.7019
GSM11086	G1(n=149	10.57513
GSM11086	G1(n=149	10.92336
GSM11086	G1(n=149	11.03372
GSM11086	G1(n=149	11.34002
GSM11086	G1(n=149	11.03246
GSM11086	G1(n=149	10.92413
GSM11086	G1(n=149	11.06885
GSM11086	G1(n=149	11.31874
GSM11086	G1(n=149	10.88088
GSM11086	G1(n=149	11.75395
GSM11086	G1(n=149	10.89071
GSM11086	G1(n=149	11.57875
GSM11086	G1(n=149	11.0587
GSM11086	G1(n=149	10.78577
GSM11086	G1(n=149	11.72386
GSM11086	G1(n=149	10.48695
GSM11086	G1(n=149	11.31727
GSM11086	G1(n=149	11.16755
GSM11086	G1(n=149	10.93699
GSM11086	G1(n=149	10.91287

GSM1108	G1(n=149	11.19381
GSM1108	G1(n=149	11.38484
GSM1108	G1(n=149	11.54186
GSM1108	G1(n=149	10.86123
GSM1108	G1(n=149	11.12819
GSM1108	G1(n=149	11.19015
GSM1108	G1(n=149	11.16755
GSM1108	G1(n=149	10.88643
GSM1108	G1(n=149	10.88125
GSM1108	G1(n=149	10.84312
GSM1108	G1(n=149	11.13623
GSM1108	G1(n=149	11.11925
GSM1108	G1(n=149	10.61288
GSM1108	G1(n=149	10.95736
GSM1108	G1(n=149	10.81068
GSM1108	G1(n=149	10.94211
GSM1108	G1(n=149	11.25809
GSM1108	G1(n=149	11.43849
GSM1108	G1(n=149	11.65893
GSM1108	G1(n=149	10.83342
GSM1108	G1(n=149	10.66696
GSM1108	G1(n=149	10.98163
GSM1108	G1(n=149	10.97952
GSM1108	G1(n=149	11.64988
GSM1108	G1(n=149	11.032
GSM1108	G1(n=149	11.30494
GSM1108	G1(n=149	10.7449
GSM1108	G1(n=149	11.27436
GSM1108	G1(n=149	11.05228
GSM1108	G1(n=149	10.79083
GSM1108	G1(n=149	10.87908
GSM1108	G1(n=149	11.10848
GSM1108	G1(n=149	11.28683
GSM1108	G1(n=149	11.4682
GSM1108	G1(n=149	10.74998
GSM1108	G1(n=149	11.08647
GSM1108	G1(n=149	11.05779
GSM1108	G1(n=149	11.12535
GSM1108	G1(n=149	10.91367
GSM1108	G1(n=149	11.33594
GSM1108	G1(n=149	10.57182
GSM1108	G1(n=149	11.25993
GSM1108	G1(n=149	11.37252
GSM1108	G1(n=149	10.96042
GSM1108	G1(n=149	10.38406
GSM1108	G1(n=149	10.66609
GSM1108	G1(n=149	11.30121
GSM1108	G1(n=149	11.18377
GSM1108	G1(n=149	10.43798
GSM1108	G1(n=149	11.97809
GSM1108	G1(n=149	10.44408
GSM1108	G1(n=149	11.12189
GSM1108	G1(n=149	11.25484
GSM1108	G1(n=149	10.66291
GSM1108	G1(n=149	11.14689
GSM1108	G1(n=149	11.32499
GSM1108	G1(n=149	11.91278
GSM1108	G1(n=149	11.74062
GSM1108	G1(n=149	11.48066

GSM11088	G1(n=149	11.27626
GSM11088	G1(n=149	11.53328
GSM11088	G1(n=149	11.22669
GSM11088	G1(n=149	11.02191
GSM11088	G1(n=149	10.87647
GSM11088	G1(n=149	11.05367
GSM11088	G1(n=149	11.38574
GSM11088	G1(n=149	11.32636
GSM11089	G1(n=149	11.52782
GSM11089	G1(n=149	11.88006
GSM11089	G1(n=149	11.56492
GSM11089	G1(n=149	11.81036
GSM11089	G1(n=149	11.23742
GSM11089	G1(n=149	8.221717
GSM11089	G1(n=149	9.941414
GSM11089	G1(n=149	11.17745
GSM11089	G1(n=149	11.07655
GSM11089	G1(n=149	11.21157
GSM11089	G1(n=149	11.51726
GSM11089	G1(n=149	11.6812
GSM11089	G1(n=149	10.84682
GSM11089	G1(n=149	11.49191
GSM11089	G1(n=149	11.20516
GSM11089	G1(n=149	10.42885
GSM11090	G1(n=149	11.70139
GSM11090	G1(n=149	11.00476
GSM11090	G1(n=149	10.75339
GSM11090	G1(n=149	11.39984
GSM11090	G1(n=149	11.52633
GSM11090	G1(n=149	11.2047
GSM11090	G1(n=149	11.69366
GSM11090	G1(n=149	10.27384
GSM11084	G2(n=83)	11.59877
GSM11084	G2(n=83)	11.60915
GSM11084	G2(n=83)	11.39162
GSM11084	G2(n=83)	11.63532
GSM11084	G2(n=83)	11.29211
GSM11084	G2(n=83)	10.89006
GSM11084	G2(n=83)	11.46027
GSM11084	G2(n=83)	11.25011
GSM11084	G2(n=83)	11.5723
GSM11084	G2(n=83)	11.24729
GSM11084	G2(n=83)	11.67532
GSM11085	G2(n=83)	11.44335
GSM11085	G2(n=83)	11.58782
GSM11085	G2(n=83)	11.93204
GSM11085	G2(n=83)	10.88788
GSM11085	G2(n=83)	11.03716
GSM11085	G2(n=83)	10.93992
GSM11085	G2(n=83)	10.9023
GSM11085	G2(n=83)	11.55633
GSM11085	G2(n=83)	11.03538
GSM11085	G2(n=83)	11.80163
GSM11085	G2(n=83)	11.1989
GSM11085	G2(n=83)	12.09316
GSM11085	G2(n=83)	11.01811
GSM11085	G2(n=83)	11.35556
GSM11085	G2(n=83)	11.62728
GSM11085	G2(n=83)	11.05566

GSM1108(	G2(n=83)	10.85056
GSM1108(	G2(n=83)	11.21052
GSM1108(	G2(n=83)	11.73942
GSM1108(	G2(n=83)	11.15188
GSM1108(	G2(n=83)	11.40975
GSM1108(	G2(n=83)	11.04873
GSM1108(	G2(n=83)	10.2109
GSM1108(	G2(n=83)	11.13518
GSM1108(	G2(n=83)	11.29493
GSM1108(	G2(n=83)	11.8654
GSM1108(	G2(n=83)	11.99739
GSM1108(	G2(n=83)	11.30562
GSM1108(	G2(n=83)	10.92334
GSM1108(	G2(n=83)	11.0302
GSM1108(	G2(n=83)	10.88823
GSM1108(	G2(n=83)	11.1754
GSM1108(	G2(n=83)	11.62682
GSM1108	G2(n=83)	11.22103
GSM1108	G2(n=83)	11.85285
GSM1108	G2(n=83)	11.4178
GSM1108	G2(n=83)	11.49768
GSM1108	G2(n=83)	11.2865
GSM1108	G2(n=83)	11.6188
GSM1108	G2(n=83)	10.80823
GSM1108	G2(n=83)	11.90889
GSM1108	G2(n=83)	11.07199
GSM1108	G2(n=83)	10.95572
GSM1108	G2(n=83)	10.75957
GSM1108	G2(n=83)	11.08041
GSM1108	G2(n=83)	10.62309
GSM1108	G2(n=83)	11.96717
GSM1108	G2(n=83)	11.34003
GSM1108	G2(n=83)	11.45411
GSM1108	G2(n=83)	11.47373
GSM1108	G2(n=83)	11.92634
GSM1108	G2(n=83)	11.4005
GSM1108	G2(n=83)	11.82938
GSM1108	G2(n=83)	10.90892
GSM1108	G2(n=83)	11.98813
GSM1108	G2(n=83)	11.04345
GSM1108	G2(n=83)	11.24395
GSM1108	G2(n=83)	10.00397
GSM1108	G2(n=83)	9.673136
GSM1108	G2(n=83)	9.00905
GSM1108	G2(n=83)	11.43766
GSM1108	G2(n=83)	12.03456
GSM1108	G2(n=83)	11.31138
GSM1108	G2(n=83)	11.23828
GSM1108	G2(n=83)	11.94786
GSM1109(	G2(n=83)	11.45411
GSM1109(	G2(n=83)	11.29493
GSM1109(	G2(n=83)	10.10851
GSM1109(	G2(n=83)	9.541605
GSM1109(	G2(n=83)	11.41885
GSM1109(	G2(n=83)	11.13157
GSM1109(	G2(n=83)	10.21574
GSM1108	G3(n=271	11.94028
GSM1108	G3(n=271	12.0502
GSM1108	G3(n=271	11.94028

GSM11084	G3(n=271	11.11665
GSM11084	G3(n=271	10.86374
GSM11084	G3(n=271	11.7591
GSM11084	G3(n=271	11.49214
GSM11084	G3(n=271	11.3996
GSM11084	G3(n=271	11.14912
GSM11084	G3(n=271	12.21143
GSM11084	G3(n=271	11.8209
GSM11084	G3(n=271	11.46379
GSM11084	G3(n=271	11.71655
GSM11084	G3(n=271	11.61329
GSM11084	G3(n=271	11.88736
GSM11084	G3(n=271	11.13965
GSM11084	G3(n=271	11.63952
GSM11084	G3(n=271	11.92877
GSM11084	G3(n=271	11.98206
GSM11084	G3(n=271	11.1832
GSM11084	G3(n=271	11.27192
GSM11084	G3(n=271	11.63165
GSM11084	G3(n=271	11.817
GSM11084	G3(n=271	11.22075
GSM11084	G3(n=271	11.62737
GSM11084	G3(n=271	11.4618
GSM11084	G3(n=271	11.43279
GSM11084	G3(n=271	11.60071
GSM11084	G3(n=271	11.48188
GSM11084	G3(n=271	11.54973
GSM11084	G3(n=271	11.28131
GSM11084	G3(n=271	11.32378
GSM11084	G3(n=271	11.81181
GSM11084	G3(n=271	11.72839
GSM11084	G3(n=271	11.63843
GSM11084	G3(n=271	10.99012
GSM11084	G3(n=271	11.50535
GSM11084	G3(n=271	11.8521
GSM11084	G3(n=271	11.28412
GSM11084	G3(n=271	11.76338
GSM11084	G3(n=271	11.20205
GSM11084	G3(n=271	11.04027
GSM11084	G3(n=271	11.45222
GSM11084	G3(n=271	11.4701
GSM11084	G3(n=271	11.44637
GSM11084	G3(n=271	11.38137
GSM11084	G3(n=271	11.14778
GSM11084	G3(n=271	11.51918
GSM11084	G3(n=271	10.98002
GSM11085	G3(n=271	11.15561
GSM11085	G3(n=271	11.83719
GSM11085	G3(n=271	11.91663
GSM11085	G3(n=271	11.59756
GSM11085	G3(n=271	10.99789
GSM11085	G3(n=271	11.77432
GSM11085	G3(n=271	11.79967
GSM11085	G3(n=271	11.31808
GSM11085	G3(n=271	11.72518
GSM11085	G3(n=271	11.57011
GSM11085	G3(n=271	12.01996
GSM11085	G3(n=271	11.81523
GSM11085	G3(n=271	11.49409

GSM11085	G3(n=271	11.6159
GSM11085	G3(n=271	11.42834
GSM11085	G3(n=271	11.00012
GSM11085	G3(n=271	11.10334
GSM11085	G3(n=271	11.01477
GSM11085	G3(n=271	11.03444
GSM11085	G3(n=271	10.44159
GSM11085	G3(n=271	11.4785
GSM11085	G3(n=271	11.59756
GSM11085	G3(n=271	10.74037
GSM11085	G3(n=271	11.34625
GSM11085	G3(n=271	11.11489
GSM11085	G3(n=271	11.24071
GSM11085	G3(n=271	11.10699
GSM11085	G3(n=271	11.3866
GSM11085	G3(n=271	11.3866
GSM11085	G3(n=271	11.5675
GSM11085	G3(n=271	11.06381
GSM11085	G3(n=271	11.2546
GSM11085	G3(n=271	11.48872
GSM11085	G3(n=271	11.64369
GSM11085	G3(n=271	11.85669
GSM11085	G3(n=271	11.36082
GSM11085	G3(n=271	11.35755
GSM11085	G3(n=271	11.62737
GSM11085	G3(n=271	11.51818
GSM11085	G3(n=271	11.69738
GSM11085	G3(n=271	11.39476
GSM11085	G3(n=271	11.25653
GSM11086	G3(n=271	12.05622
GSM11086	G3(n=271	11.9785
GSM11086	G3(n=271	11.69104
GSM11086	G3(n=271	11.57863
GSM11086	G3(n=271	11.32662
GSM11086	G3(n=271	11.63952
GSM11086	G3(n=271	11.60124
GSM11086	G3(n=271	11.84746
GSM11086	G3(n=271	11.12995
GSM11086	G3(n=271	11.59441
GSM11086	G3(n=271	11.27288
GSM11086	G3(n=271	11.36898
GSM11086	G3(n=271	11.26868
GSM11086	G3(n=271	11.0135
GSM11086	G3(n=271	11.5696
GSM11086	G3(n=271	11.49925
GSM11086	G3(n=271	12.09399
GSM11086	G3(n=271	11.33146
GSM11086	G3(n=271	11.28224
GSM11086	G3(n=271	11.78515
GSM11086	G3(n=271	11.64159
GSM11086	G3(n=271	11.95654
GSM11086	G3(n=271	11.41067
GSM11086	G3(n=271	12.32805
GSM11086	G3(n=271	11.01027
GSM11086	G3(n=271	11.20387
GSM11086	G3(n=271	11.79912
GSM11086	G3(n=271	11.86337
GSM11086	G3(n=271	10.73121
GSM11086	G3(n=271	11.49769

GSM1108(	G3(n=271	11.99965
GSM1108(	G3(n=271	11.03571
GSM1108(	G3(n=271	10.99962
GSM1108(	G3(n=271	11.27102
GSM1108(	G3(n=271	11.20474
GSM1108(	G3(n=271	11.82199
GSM1108(	G3(n=271	10.99612
GSM1108(	G3(n=271	11.13965
GSM1108	G3(n=271	11.21398
GSM1108	G3(n=271	12.77192
GSM1108	G3(n=271	11.62002
GSM1108	G3(n=271	11.60494
GSM1108	G3(n=271	10.75789
GSM1108	G3(n=271	11.80134
GSM1108	G3(n=271	11.20474
GSM1108	G3(n=271	11.28551
GSM1108	G3(n=271	11.72355
GSM1108	G3(n=271	11.51672
GSM1108	G3(n=271	10.90272
GSM1108	G3(n=271	11.39282
GSM1108	G3(n=271	11.6747
GSM1108	G3(n=271	11.09447
GSM1108	G3(n=271	11.46128
GSM1108	G3(n=271	11.56063
GSM1108	G3(n=271	10.87573
GSM1108	G3(n=271	11.52018
GSM1108	G3(n=271	11.87016
GSM1108	G3(n=271	10.23136
GSM1108	G3(n=271	11.75306
GSM1108	G3(n=271	11.27528
GSM1108	G3(n=271	11.45311
GSM1108	G3(n=271	11.49613
GSM1108	G3(n=271	11.83999
GSM1108	G3(n=271	11.3996
GSM1108	G3(n=271	10.85053
GSM1108	G3(n=271	11.23117
GSM1108	G3(n=271	10.32436
GSM1108	G3(n=271	11.34483
GSM1108	G3(n=271	11.66992
GSM1108	G3(n=271	11.53485
GSM1108	G3(n=271	11.51274
GSM1108	G3(n=271	11.16025
GSM1108	G3(n=271	11.78966
GSM1108	G3(n=271	11.31238
GSM1108	G3(n=271	10.95499
GSM1108	G3(n=271	11.96305
GSM1108	G3(n=271	10.6997
GSM1108	G3(n=271	11.08521
GSM1108	G3(n=271	11.37993
GSM1108	G3(n=271	10.95756
GSM1108	G3(n=271	11.2919
GSM1108	G3(n=271	11.74599
GSM1108	G3(n=271	11.20161
GSM1108	G3(n=271	11.19146
GSM1108	G3(n=271	11.64724
GSM1108	G3(n=271	10.87145
GSM1108	G3(n=271	10.76882
GSM1108	G3(n=271	11.34483
GSM1108	G3(n=271	11.90122

GSM1108	G3(n=271	11.88506
GSM1108	G3(n=271	11.47654
GSM1108	G3(n=271	11.56453
GSM1108	G3(n=271	11.71226
GSM1108	G3(n=271	12.03738
GSM1108	G3(n=271	11.75149
GSM1108	G3(n=271	11.83445
GSM1108	G3(n=271	12.01646
GSM1108	G3(n=271	11.27054
GSM1108	G3(n=271	11.53835
GSM1108	G3(n=271	12.05993
GSM1108	G3(n=271	10.95756
GSM1108	G3(n=271	11.7208
GSM1108	G3(n=271	12.18562
GSM1108	G3(n=271	11.19612
GSM1108	G3(n=271	11.41828
GSM1108	G3(n=271	11.55068
GSM1108	G3(n=271	11.62996
GSM1108	G3(n=271	11.84289
GSM1108	G3(n=271	11.45802
GSM1108	G3(n=271	11.68788
GSM1108	G3(n=271	11.61847
GSM1108	G3(n=271	11.73529
GSM1108	G3(n=271	11.00804
GSM1108	G3(n=271	10.70992
GSM1108	G3(n=271	11.93114
GSM1108	G3(n=271	11.58632
GSM1108	G3(n=271	11.98902
GSM1108	G3(n=271	9.168611
GSM1108	G3(n=271	8.715668
GSM1108	G3(n=271	8.549839
GSM1108	G3(n=271	8.44013
GSM1108	G3(n=271	8.782311
GSM1108	G3(n=271	8.272711
GSM1108	G3(n=271	8.822164
GSM1108	G3(n=271	11.29521
GSM1108	G3(n=271	11.60699
GSM1108	G3(n=271	11.40869
GSM1108	G3(n=271	10.88803
GSM1108	G3(n=271	11.86461
GSM1108	G3(n=271	11.00458
GSM1108	G3(n=271	10.82551
GSM1108	G3(n=271	11.35419
GSM1108	G3(n=271	11.48474
GSM1108	G3(n=271	11.61641
GSM1108	G3(n=271	11.47996
GSM1108	G3(n=271	11.14189
GSM1108	G3(n=271	11.5443
GSM1108	G3(n=271	12.34569
GSM1108	G3(n=271	11.75525
GSM1108	G3(n=271	11.75692
GSM1108	G3(n=271	11.68371
GSM1108	G3(n=271	11.46278
GSM1108	G3(n=271	11.34048
GSM1108	G3(n=271	11.20161
GSM1108	G3(n=271	10.84751
GSM1108	G3(n=271	11.03796
GSM1108	G3(n=271	11.13965
GSM1108	G3(n=271	11.71976

GSM11089	G3(n=271	11.89663
GSM11089	G3(n=271	12.61567
GSM11089	G3(n=271	11.79572
GSM11089	G3(n=271	11.38085
GSM11089	G3(n=271	11.51432
GSM11089	G3(n=271	11.78249
GSM11089	G3(n=271	12.3199
GSM11089	G3(n=271	11.15001
GSM11089	G3(n=271	11.27528
GSM11089	G3(n=271	11.61222
GSM11089	G3(n=271	11.53537
GSM11089	G3(n=271	11.66992
GSM11089	G3(n=271	11.58473
GSM11090	G3(n=271	11.16785
GSM11090	G3(n=271	11.71815
GSM11090	G3(n=271	11.20115
GSM11090	G3(n=271	11.07143
GSM11090	G3(n=271	11.50725
GSM11090	G3(n=271	11.87355
GSM11090	G3(n=271	11.51578
GSM11090	G3(n=271	10.18693
GSM11090	G3(n=271	11.14727
GSM11090	G3(n=271	10.45065
GSM11090	G3(n=271	11.03571
GSM11090	G3(n=271	11.88797
GSM11090	G3(n=271	11.47213
GSM11090	G3(n=271	11.2897
GSM11090	G3(n=271	11.62267
GSM11090	G3(n=271	10.32598
GSM11090	G3(n=271	11.61901
GSM11090	G3(n=271	11.52983
GSM11090	G3(n=271	12.05749

Supplementary Table 7

rownames	Type	values
GSM3402	G1(n=54)	11.59802
GSM3402	G1(n=54)	11.38543
GSM3402	G1(n=54)	11.49437
GSM3402	G1(n=54)	11.17397
GSM3403	G1(n=54)	11.4345
GSM3403	G1(n=54)	11.78827
GSM3403	G1(n=54)	11.60009
GSM3403	G1(n=54)	11.35877
GSM3403	G1(n=54)	10.37938
GSM3403	G1(n=54)	12.00651
GSM3403	G1(n=54)	10.61435
GSM3403	G1(n=54)	11.07612
GSM3403	G1(n=54)	11.58836
GSM3403	G1(n=54)	11.14299
GSM3403	G1(n=54)	11.30076
GSM3403	G1(n=54)	11.55261
GSM3403	G1(n=54)	11.98518
GSM3403	G1(n=54)	11.33442
GSM3403	G1(n=54)	11.51761
GSM3403	G1(n=54)	11.03577
GSM3403	G1(n=54)	11.25438
GSM3403	G1(n=54)	11.23326
GSM3403	G1(n=54)	11.51352
GSM3403	G1(n=54)	11.76014
GSM3403	G1(n=54)	11.68462
GSM3403	G1(n=54)	11.32301
GSM3403	G1(n=54)	10.89981
GSM3403	G1(n=54)	11.33094
GSM3403	G1(n=54)	11.76068
GSM3403	G1(n=54)	11.15635
GSM3403	G1(n=54)	11.90492
GSM3403	G1(n=54)	11.87488
GSM3403	G1(n=54)	11.60158
GSM3403	G1(n=54)	11.09494
GSM3403	G1(n=54)	11.09622
GSM3403	G1(n=54)	11.03028
GSM3403	G1(n=54)	11.41609
GSM3403	G1(n=54)	11.54929
GSM3403	G1(n=54)	11.30363
GSM3403	G1(n=54)	11.76565
GSM3403	G1(n=54)	11.16376
GSM3403	G1(n=54)	10.97778
GSM3403	G1(n=54)	11.65228
GSM3403	G1(n=54)	11.41943
GSM3403	G1(n=54)	11.30748
GSM3403	G1(n=54)	10.7293
GSM3403	G1(n=54)	10.95886
GSM3403	G1(n=54)	11.02363
GSM3403	G1(n=54)	11.05213
GSM3403	G1(n=54)	11.28029
GSM3403	G1(n=54)	11.14747
GSM3403	G1(n=54)	11.05682
GSM3403	G1(n=54)	11.75622
GSM3403	G1(n=54)	11.00003
GSM3402	G2(n=56)	10.98894
GSM3402	G2(n=56)	11.43902
GSM3402	G2(n=56)	11.91718
GSM3403	G2(n=56)	11.41803

GSM34030	G2(n=56)	11.50954
GSM34030	G2(n=56)	11.85828
GSM34030	G2(n=56)	11.20999
GSM34030	G2(n=56)	11.55938
GSM34030	G2(n=56)	11.96951
GSM34030	G2(n=56)	12.4163
GSM34030	G2(n=56)	10.69646
GSM34030	G2(n=56)	11.25379
GSM34030	G2(n=56)	11.17447
GSM34030	G2(n=56)	11.28071
GSM34031	G2(n=56)	11.80895
GSM34031	G2(n=56)	11.35171
GSM34031	G2(n=56)	11.05821
GSM34031	G2(n=56)	11.45028
GSM34031	G2(n=56)	12.01834
GSM34031	G2(n=56)	11.34531
GSM34031	G2(n=56)	11.80636
GSM34031	G2(n=56)	10.86455
GSM34031	G2(n=56)	11.35091
GSM34031	G2(n=56)	11.08011
GSM34031	G2(n=56)	11.72213
GSM34031	G2(n=56)	11.25974
GSM34032	G2(n=56)	11.75006
GSM34032	G2(n=56)	11.70249
GSM34032	G2(n=56)	12.05912
GSM34032	G2(n=56)	11.41122
GSM34032	G2(n=56)	11.08616
GSM34032	G2(n=56)	12.01953
GSM34033	G2(n=56)	11.2745
GSM34033	G2(n=56)	11.40898
GSM34033	G2(n=56)	11.68902
GSM34033	G2(n=56)	11.13439
GSM34033	G2(n=56)	11.66735
GSM34033	G2(n=56)	11.38444
GSM34033	G2(n=56)	11.51354
GSM34033	G2(n=56)	11.40125
GSM34033	G2(n=56)	11.39393
GSM34033	G2(n=56)	12.08838
GSM34033	G2(n=56)	11.9997
GSM34033	G2(n=56)	11.29656
GSM34034	G2(n=56)	11.48835
GSM34034	G2(n=56)	11.55025
GSM34034	G2(n=56)	10.9122
GSM34034	G2(n=56)	11.49719
GSM34034	G2(n=56)	11.55025
GSM34034	G2(n=56)	11.23509
GSM34034	G2(n=56)	11.59295
GSM34034	G2(n=56)	11.25217
GSM34034	G2(n=56)	10.32575
GSM34034	G2(n=56)	11.71319
GSM34034	G2(n=56)	11.17184
GSM34034	G2(n=56)	11.31779
GSM34029	G3(n=212	12.00805
GSM34029	G3(n=212	11.6013
GSM34029	G3(n=212	11.6244
GSM34029	G3(n=212	11.58714
GSM34029	G3(n=212	11.4226
GSM34029	G3(n=212	11.70583
GSM34029	G3(n=212	11.37622

GSM3402!	G3(n=212	11.48884
GSM3402!	G3(n=212	11.79902
GSM3402!	G3(n=212	11.67673
GSM3402!	G3(n=212	11.86955
GSM3402!	G3(n=212	11.04522
GSM3402!	G3(n=212	11.83885
GSM3402!	G3(n=212	11.39749
GSM3402!	G3(n=212	10.79092
GSM3402!	G3(n=212	11.35655
GSM3402!	G3(n=212	11.52791
GSM3403(	G3(n=212	11.07986
GSM3403(	G3(n=212	11.91503
GSM3403(	G3(n=212	11.9511
GSM3403(	G3(n=212	11.79166
GSM3403(	G3(n=212	12.04401
GSM3403(	G3(n=212	11.61727
GSM3403(	G3(n=212	11.64745
GSM3403(	G3(n=212	11.50473
GSM3403(	G3(n=212	10.29115
GSM3403(	G3(n=212	11.82746
GSM3403(	G3(n=212	11.57823
GSM3403(	G3(n=212	11.16877
GSM3403(	G3(n=212	11.79166
GSM3403(	G3(n=212	11.46389
GSM3403(	G3(n=212	11.82419
GSM3403(	G3(n=212	12.39459
GSM3403(	G3(n=212	11.70583
GSM3403(	G3(n=212	10.93167
GSM3403(	G3(n=212	11.08028
GSM3403(	G3(n=212	12.13191
GSM3403(	G3(n=212	11.51329
GSM3403(	G3(n=212	10.42522
GSM3403(	G3(n=212	11.5371
GSM3403(	G3(n=212	11.64343
GSM3403(	G3(n=212	11.81828
GSM3403(	G3(n=212	11.18492
GSM3403(	G3(n=212	11.64944
GSM3403(	G3(n=212	12.27417
GSM3403(	G3(n=212	12.12107
GSM3403(	G3(n=212	12.32923
GSM3403(	G3(n=212	11.41835
GSM3403(	G3(n=212	11.65449
GSM3403(	G3(n=212	10.91757
GSM3403(	G3(n=212	12.42869
GSM3403(	G3(n=212	11.58477
GSM3403(	G3(n=212	10.35616
GSM3403(	G3(n=212	11.26533
GSM3403(	G3(n=212	11.97422
GSM3403(	G3(n=212	11.49513
GSM3403(	G3(n=212	11.27069
GSM3403(	G3(n=212	11.61289
GSM3403(	G3(n=212	11.73851
GSM3403(	G3(n=212	11.16835
GSM3403(	G3(n=212	11.41368
GSM3403(	G3(n=212	11.65764
GSM3403(	G3(n=212	11.69508
GSM3403(	G3(n=212	11.71942
GSM3403(	G3(n=212	11.3647
GSM3403(	G3(n=212	11.64052

GSM3403(	G3(n=212	11.42075
GSM3403(	G3(n=212	11.27657
GSM3403(	G3(n=212	11.44781
GSM3403(	G3(n=212	11.61289
GSM3403(	G3(n=212	11.44736
GSM3403(	G3(n=212	12.09039
GSM3403(	G3(n=212	11.75376
GSM34031	G3(n=212	12.01101
GSM34031	G3(n=212	11.22617
GSM34031	G3(n=212	11.27156
GSM34031	G3(n=212	11.94997
GSM34031	G3(n=212	11.99249
GSM34031	G3(n=212	11.82046
GSM34031	G3(n=212	11.38532
GSM34031	G3(n=212	11.41459
GSM34031	G3(n=212	11.61679
GSM34031	G3(n=212	11.95839
GSM34031	G3(n=212	11.54819
GSM34031	G3(n=212	11.90077
GSM34031	G3(n=212	11.49224
GSM34031	G3(n=212	12.32206
GSM34031	G3(n=212	12.22176
GSM34031	G3(n=212	12.03931
GSM34031	G3(n=212	11.84309
GSM34031	G3(n=212	11.30535
GSM34031	G3(n=212	11.29161
GSM34031	G3(n=212	11.75591
GSM34031	G3(n=212	10.89646
GSM34031	G3(n=212	11.51228
GSM34031	G3(n=212	11.2266
GSM34031	G3(n=212	11.6894
GSM34031	G3(n=212	11.72687
GSM34031	G3(n=212	11.24083
GSM34031	G3(n=212	12.08496
GSM34031	G3(n=212	11.76583
GSM34031	G3(n=212	11.84416
GSM34031	G3(n=212	11.65345
GSM34031	G3(n=212	11.77745
GSM34031	G3(n=212	11.87772
GSM34031	G3(n=212	11.6244
GSM34031	G3(n=212	11.55159
GSM34031	G3(n=212	12.03406
GSM34031	G3(n=212	10.74305
GSM34031	G3(n=212	11.83122
GSM34031	G3(n=212	10.93932
GSM34031	G3(n=212	12.1693
GSM34031	G3(n=212	11.80663
GSM34031	G3(n=212	11.58334
GSM34031	G3(n=212	11.26095
GSM34031	G3(n=212	11.39749
GSM34031	G3(n=212	11.35323
GSM34031	G3(n=212	12.84414
GSM34031	G3(n=212	10.4233
GSM34032	G3(n=212	11.64991
GSM34032	G3(n=212	11.92568
GSM34032	G3(n=212	11.35472
GSM34032	G3(n=212	12.05674
GSM34032	G3(n=212	11.72091
GSM34032	G3(n=212	11.60035

GSM34032	G3(n=212	11.72635
GSM34032	G3(n=212	11.72091
GSM34032	G3(n=212	11.54723
GSM34032	G3(n=212	11.63207
GSM34032	G3(n=212	11.57437
GSM34032	G3(n=212	11.1178
GSM34032	G3(n=212	11.48263
GSM34032	G3(n=212	12.11435
GSM34032	G3(n=212	11.55989
GSM34032	G3(n=212	11.96574
GSM34032	G3(n=212	11.98072
GSM34032	G3(n=212	11.91781
GSM34032	G3(n=212	11.59745
GSM34032	G3(n=212	11.42829
GSM34032	G3(n=212	11.87772
GSM34032	G3(n=212	11.37392
GSM34032	G3(n=212	11.59305
GSM34032	G3(n=212	11.35123
GSM34032	G3(n=212	10.4233
GSM34032	G3(n=212	11.47931
GSM34032	G3(n=212	11.75267
GSM34032	G3(n=212	12.07256
GSM34032	G3(n=212	11.43441
GSM34032	G3(n=212	11.53522
GSM34032	G3(n=212	11.41882
GSM34032	G3(n=212	12.40813
GSM34032	G3(n=212	11.68284
GSM34032	G3(n=212	11.49801
GSM34032	G3(n=212	11.95839
GSM34032	G3(n=212	11.5376
GSM34032	G3(n=212	11.42682
GSM34032	G3(n=212	11.68736
GSM34032	G3(n=212	11.88156
GSM34033	G3(n=212	11.93188
GSM34033	G3(n=212	11.4784
GSM34033	G3(n=212	11.08569
GSM34033	G3(n=212	12.40161
GSM34033	G3(n=212	11.99191
GSM34033	G3(n=212	12.68755
GSM34033	G3(n=212	10.54767
GSM34033	G3(n=212	12.23689
GSM34033	G3(n=212	11.49128
GSM34033	G3(n=212	12.85655
GSM34033	G3(n=212	11.28978
GSM34033	G3(n=212	11.09901
GSM34033	G3(n=212	11.11608
GSM34033	G3(n=212	12.01385
GSM34033	G3(n=212	11.41093
GSM34033	G3(n=212	10.85105
GSM34033	G3(n=212	10.96697
GSM34033	G3(n=212	11.35697
GSM34033	G3(n=212	10.33448
GSM34033	G3(n=212	11.70028
GSM34033	G3(n=212	11.86732
GSM34033	G3(n=212	11.75484
GSM34033	G3(n=212	11.58226
GSM34033	G3(n=212	11.18225
GSM34033	G3(n=212	11.08487
GSM34033	G3(n=212	11.46389

GSM34033	G3(n=212	10.83352
GSM34033	G3(n=212	11.12199
GSM34033	G3(n=212	11.3684
GSM34033	G3(n=212	11.36978
GSM34033	G3(n=212	11.20097
GSM34033	G3(n=212	11.6439
GSM34033	G3(n=212	11.42976
GSM34033	G3(n=212	11.36749
GSM34033	G3(n=212	11.75111
GSM34033	G3(n=212	11.28978
GSM34033	G3(n=212	11.47008
GSM34033	G3(n=212	11.32773
GSM34033	G3(n=212	11.84529
GSM34033	G3(n=212	11.71204
GSM34033	G3(n=212	11.44421
GSM34033	G3(n=212	11.93413
GSM34033	G3(n=212	11.77856
GSM34034	G3(n=212	11.72924
GSM34034	G3(n=212	11.09949
GSM34034	G3(n=212	11.24758
GSM34034	G3(n=212	11.6439
GSM34034	G3(n=212	11.59596
GSM34034	G3(n=212	11.37796
GSM34034	G3(n=212	11.80554
GSM34034	G3(n=212	11.46486
GSM34034	G3(n=212	11.67673
GSM34034	G3(n=212	11.5539
GSM34034	G3(n=212	11.87938

Supplementary Table 8

	V1	V2	V3
DMS.53	SCLC	Tumor	12.36
NCI.H169	SCLC	Tumor	10.04
NCI.H118	SCLC	Tumor	12.86
NCI.H222	SCLC	Tumor	16.85
NCI.H510	SCLC	Tumor	10.24
NCI.H206	SCLC	Tumor	13.66
NCI.H134	SCLC	Tumor	12.68
NCI.H202	SCLC	Tumor	11.21
HCC.33	SCLC	Tumor	22.29
COLO.668	SCLC	Tumor	9.44
NCI.H196	SCLC	Tumor	16.2
SW.1271	SCLC	Tumor	10.55
NCI.H82	SCLC	Tumor	9.78
DMS.454	SCLC	Tumor	7.46
COR.L47	SCLC	Tumor	6.96
DMS.273	SCLC	Tumor	4.17
COR.L311	SCLC	Tumor	6.51
NCI.H228	SCLC	Tumor	13.21
NCI.H133	SCLC	Tumor	14.81
NCI.H208	SCLC	Tumor	8.67
NCI.H109	SCLC	Tumor	12.44
NCI.H110	SCLC	Tumor	9.04
NCI.H143	SCLC	Tumor	15.38
NCI.H187	SCLC	Tumor	20.08
COR.L24	SCLC	Tumor	7.6
COR.L88	SCLC	Tumor	18.8
COR.L95	SCLC	Tumor	8.85
COR.L279	SCLC	Tumor	8.05
DMS.153	SCLC	Tumor	20.44
NCI.H104	SCLC	Tumor	16.89
NCI.H161	SCLC	Tumor	18.41
NCI.H69	SCLC	Tumor	10.11
NCI.H193	SCLC	Tumor	18.86
NCI.H183	SCLC	Tumor	12.17
DMS.114	SCLC	Tumor	9.89
NCI.H524	SCLC	Tumor	7.19
NCI.H209	SCLC	Tumor	18.62
SBC.5	SCLC	Tumor	6.62
NCI.H196	SCLC	Tumor	14.43
NCI.H889	SCLC	Tumor	8.28
NCI.H211	SCLC	Tumor	6.52
NCI.H217	SCLC	Tumor	7.62
NCI.H146	SCLC	Tumor	6.91
DMS.79	SCLC	Tumor	13.78
NCI.H526	SCLC	Tumor	15.32
NCI.H219	SCLC	Tumor	10.54
NCI.H446	SCLC	Tumor	13.93
SCLC.21H	SCLC	Tumor	11.87
SHP.77	SCLC	Tumor	4.65
NCI.H841	SCLC	Tumor	9.64
SW1116	COAD_RE	Tumor	2.75
OUMS.23	COAD_RE	Tumor	8
NCI.H716	COAD_RE	Tumor	19.21
SW480	COAD_RE	Tumor	5
SNU.C2A	COAD_RE	Tumor	18.53
NCI.H508	COAD_RE	Tumor	5.8
LS123	COAD_RE	Tumor	4.79
SNU.1197	COAD_RE	Tumor	6.06

CW.2	COAD_RE	Tumor	9.99
SNU.61	COAD_RE	Tumor	6.19
HCC.1588	COAD_RE	Tumor	6.22
HT115	COAD_RE	Tumor	4.66
SNU.1033	COAD_RE	Tumor	3.79
SNU.C5	COAD_RE	Tumor	13.5
SNU.C4	COAD_RE	Tumor	15.64
HCT.15	COAD_RE	Tumor	13.17
SW1463	COAD_RE	Tumor	6.05
CCK.81	COAD_RE	Tumor	7.33
COLO.201	COAD_RE	Tumor	9.81
C2BBe1	COAD_RE	Tumor	11.16
LS513	COAD_RE	Tumor	7.35
SW837	COAD_RE	Tumor	22.44
GP2d	COAD_RE	Tumor	11.82
HT55	COAD_RE	Tumor	6.39
MDST8	COAD_RE	Tumor	5.31
SK.CO.1	COAD_RE	Tumor	5.16
SNU.81	COAD_RE	Tumor	38.96
SNU.1040	COAD_RE	Tumor	9.89
SNU.175	COAD_RE	Tumor	15.89
SNU.407	COAD_RE	Tumor	9.94
SNU.283	COAD_RE	Tumor	4.54
SNU.503	COAD_RE	Tumor	9
Hs.675.T	COAD_RE	Tumor	8.51
HCC.56	COAD_RE	Tumor	5.33
RCM.1	COAD_RE	Tumor	6.32
RKO	COAD_RE	Tumor	9.83
SNU.C1	COAD_RE	Tumor	5.65
SW620	COAD_RE	Tumor	5.76
HCT.116	COAD_RE	Tumor	16.38
LS411N	COAD_RE	Tumor	11.74
LoVo	COAD_RE	Tumor	9.75
LS1034	COAD_RE	Tumor	9.44
SW48	COAD_RE	Tumor	15.5
LS.180	COAD_RE	Tumor	9.3
SW403	COAD_RE	Tumor	8.13
NCI.H747	COAD_RE	Tumor	7.77
SW1417	COAD_RE	Tumor	5.73
SW948	COAD_RE	Tumor	7.9
HT.29	COAD_RE	Tumor	8.2
KM12	COAD_RE	Tumor	10.78
T84	COAD_RE	Tumor	5.65
CL.14	COAD_RE	Tumor	5.39
CL.40	COAD_RE	Tumor	6.89
Hs.698.T	COAD_RE	Tumor	9.84
COLO.678	COAD_RE	Tumor	11.19
COLO.320	COAD_RE	Tumor	9.18
CL.11	COAD_RE	Tumor	6.32
CL.34	COAD_RE	Tumor	7.34
NA.	COAD_RE	Tumor	8.19
P3HR.1	DLBC	Tumor	9.58
Toledo	DLBC	Tumor	22.56
Raji	DLBC	Tumor	7.91
BL.41	DLBC	Tumor	8.05
A4.Fuk	DLBC	Tumor	11.1
A3.KAW	DLBC	Tumor	7.94
MC116	DLBC	Tumor	8.69
CA46	DLBC	Tumor	11.96

BCP.1	DLBC	Tumor	10.71
DB	DLBC	Tumor	11.74
GA.10	DLBC	Tumor	10.73
EB1	DLBC	Tumor	15.38
Daudi	DLBC	Tumor	10.09
HT	DLBC	Tumor	14.56
JM1	DLBC	Tumor	13.39
NAMALW.	DLBC	Tumor	11.07
SU.DHL.8	DLBC	Tumor	9.2
OCI.LY3	DLBC	Tumor	8.42
BL.70	DLBC	Tumor	11.11
CI.1	DLBC	Tumor	9.81
U.937	DLBC	Tumor	9.69
ST486	DLBC	Tumor	15.41
RL	DLBC	Tumor	10.52
GRANTA.5	DLBC	Tumor	11.9
JeKo.1	DLBC	Tumor	9.43
NU.DHL.1	DLBC	Tumor	4.55
SU.DHL.1(	DLBC	Tumor	7.34
SU.DHL.4	DLBC	Tumor	5.9
OCI.LY.19	DLBC	Tumor	5.62
Mino	DLBC	Tumor	11.39
DOHH.2	DLBC	Tumor	6.2
RI.1	DLBC	Tumor	6.14
KARPAS.4:	DLBC	Tumor	18.44
Pfeiffer	DLBC	Tumor	8.01
NU.DUL.1	DLBC	Tumor	10.17
SU.DHL.6	DLBC	Tumor	8.56
REC.1	DLBC	Tumor	6.6
SU.DHL.5	DLBC	Tumor	14.5
WSU.DLCI	DLBC	Tumor	5.46
UM.UC.3	BLCA	Tumor	12.14
X5637	BLCA	Tumor	7.01
U.BLC1	BLCA	Tumor	11.21
J82	BLCA	Tumor	9.42
KMBC.2	BLCA	Tumor	11.08
T24	BLCA	Tumor	9.85
X253J	BLCA	Tumor	5.19
X253J.BV	BLCA	Tumor	6.76
HT.1197	BLCA	Tumor	5.3
UM.UC.1	BLCA	Tumor	7.27
TCCSUP	BLCA	Tumor	9.85
JMSU.1	BLCA	Tumor	10.68
SW.780	BLCA	Tumor	12.37
HT.1376	BLCA	Tumor	14.48
SCaBER	BLCA	Tumor	4.85
RT4	BLCA	Tumor	7.5
RT.112	BLCA	Tumor	6.7
SW.1710	BLCA	Tumor	7.58
VM.CUB1	BLCA	Tumor	11.19
CAL.29	BLCA	Tumor	7.55
BFTC.905	BLCA	Tumor	8.6
KU.19.19	BLCA	Tumor	7.97
BC.3C	BLCA	Tumor	6.28
X639.V	BLCA	Tumor	9.06
X647.V	BLCA	Tumor	8.25
HOS	SARC	Tumor	12.27
TC.71	SARC	Tumor	7.96
RKN	SARC	Tumor	10.2

HT.1080	SARC	Tumor	12.41
SK.UT.1	SARC	Tumor	11.53
T1.73	SARC	Tumor	14.62
Hs.870.T	SARC	Tumor	14.11
SJSA.1	SARC	Tumor	11.61
A.204	SARC	Tumor	9.02
A.673	SARC	Tumor	12.45
SK.N.MC	SARC	Tumor	13.78
RD.ES	SARC	Tumor	9.91
MES.SA	SARC	Tumor	8.59
GCT	SARC	Tumor	6.92
U.2.OS	SARC	Tumor	13.88
G.401	SARC	Tumor	12.24
Saos.2	SARC	Tumor	10.15
SK.ES.1	SARC	Tumor	12.6
RH.30	SARC	Tumor	8.07
KYM.1	SARC	Tumor	10.17
Hs.819.T	SARC	Tumor	7.49
MG.63	SARC	Tumor	6.45
TE.617.T	SARC	Tumor	16.38
G.292..clo	SARC	Tumor	11.31
Hs.729	SARC	Tumor	12.9
SW.1353	SARC	Tumor	14.13
G.402	SARC	Tumor	8.29
RD	SARC	Tumor	12.51
TE.125.T	SARC	Tumor	18.18
RH.41	SARC	Tumor	9.09
CAL.78	SARC	Tumor	26.64
TE.441.T	SARC	Tumor	13.84
Hs.863.T	SARC	Tumor	12.89
MHH.ES.1	SARC	Tumor	10.12
CADO.ES1	SARC	Tumor	5.24
S.117	SARC	Tumor	4.52
RH.18	SARC	Tumor	5.76
HuNS1	MM	Tumor	11.7
KMS.18	MM	Tumor	4.28
KMS.21BM	MM	Tumor	8.7
KMS.26	MM	Tumor	9.66
KMS.27	MM	Tumor	5.42
KMS.28BM	MM	Tumor	9.74
KMS.34	MM	Tumor	8.17
KHM.1B	MM	Tumor	4.72
KMS.11	MM	Tumor	13.21
KE.97	MM	Tumor	13.2
U266B1	MM	Tumor	7.8
KMM.1	MM	Tumor	10.56
KMS.20	MM	Tumor	12.71
NCI.H929	MM	Tumor	6.42
KMS.12.BM	MM	Tumor	6.83
MM1.S	MM	Tumor	9.27
RPMI.822	MM	Tumor	4.05
MOLP.2	MM	Tumor	7.52
JJN.3	MM	Tumor	10.94
SK.MM.2	MM	Tumor	7.04
LP.1	MM	Tumor	14.33
OPM.2	MM	Tumor	10.85
L.363	MM	Tumor	12.63
KARPAS.6	MM	Tumor	7.7
EJM	MM	Tumor	4.39

AMO.1	MM	Tumor	4.74
MOLP.8	MM	Tumor	5.01
NA..1	MM	Tumor	11.63
AML.193	LAML	Tumor	14.08
P31.FUJ	LAML	Tumor	15.65
HEL	LAML	Tumor	8.53
KO52	LAML	Tumor	14.15
HEL.92.1.7	LAML	Tumor	12.12
THP.1	LAML	Tumor	12.65
GDM.1	LAML	Tumor	12.85
KASUMI.1	LAML	Tumor	14.43
CMK	LAML	Tumor	12.58
SKM.1	LAML	Tumor	8.9
KG.1	LAML	Tumor	15.22
HL.60	LAML	Tumor	5.85
MV.4.11	LAML	Tumor	6.97
MOLM.13	LAML	Tumor	11.33
MONO.M/	LAML	Tumor	14.93
TF.1	LAML	Tumor	10.7
ME.1	LAML	Tumor	6.3
OCI.AML3	LAML	Tumor	5.87
Kasumi.6	LAML	Tumor	10.52
EOL.1	LAML	Tumor	12.76
MONO.M/	LAML	Tumor	12.25
PL.21	LAML	Tumor	9.71
OCI.AML2	LAML	Tumor	13.35
OCI.AML5	LAML	Tumor	8.45
NOMO.1	LAML	Tumor	10.46
M.07e	LAML	Tumor	11.42
NB.4	LAML	Tumor	12.42
OCI.M1	LAML	Tumor	8.96
SIG.M5	LAML	Tumor	9.79
MOLM.16	LAML	Tumor	9.43
F.36P	LAML	Tumor	7.34
NA..2	LAML	Tumor	12.39
PLB.985	LAML	Tumor	7.76
SKNO.1	LAML	Tumor	7.73
MUTZ.3	LAML	Tumor	20.03
RVH.421	SKCM	Tumor	8.55
Hs.895.T	SKCM	Tumor	11.54
Hs.940.T	SKCM	Tumor	10.49
SK.MEL.1	SKCM	Tumor	7.82
Hs.936.T	SKCM	Tumor	8.38
SK.MEL.28	SKCM	Tumor	6.18
SH.4	SKCM	Tumor	8.38
CJM	SKCM	Tumor	14.63
WM.115	SKCM	Tumor	15.56
COLO.800	SKCM	Tumor	6.78
UACC.62	SKCM	Tumor	4.23
RPMI.7951	SKCM	Tumor	11.73
COLO.783	SKCM	Tumor	9.65
COLO.792	SKCM	Tumor	5.36
WM.983B	SKCM	Tumor	11.03
WM.793	SKCM	Tumor	10.13
MDA.MB.4	SKCM	Tumor	11.52
WM1799	SKCM	Tumor	11.44
MeWo	SKCM	Tumor	7.74
WM.88	SKCM	Tumor	14.24
IGR.1	SKCM	Tumor	8.47

A2058	SKCM	Tumor	10.33
IGR.39	SKCM	Tumor	7.01
HT.144	SKCM	Tumor	7.82
SK.MEL.31	SKCM	Tumor	7.01
Hs.839.T	SKCM	Tumor	9.82
Hs.939.T	SKCM	Tumor	10.03
Hs.688.A.1	SKCM	Tumor	9.77
A101D	SKCM	Tumor	7.54
COLO.829	SKCM	Tumor	10.76
IPC.298	SKCM	Tumor	8.43
SK.MEL.24	SKCM	Tumor	8.92
K029AX	SKCM	Tumor	5.44
Hs.695T	SKCM	Tumor	6.96
SK.MEL.3	SKCM	Tumor	8.39
HMCB	SKCM	Tumor	11.71
Malme.3M	SKCM	Tumor	12.78
MEL.JUSO	SKCM	Tumor	9.5
COLO.679	SKCM	Tumor	6.76
MEL.HO	SKCM	Tumor	7.6
G.361	SKCM	Tumor	10.87
COLO.741	SKCM	Tumor	6.4
SK.MEL.30	SKCM	Tumor	6.98
C32	SKCM	Tumor	15.37
A.375	SKCM	Tumor	8.52
Hs.294T	SKCM	Tumor	12.12
SK.MEL.5	SKCM	Tumor	8.39
Hs.944.T	SKCM	Tumor	11.07
WM.266.4	SKCM	Tumor	9.21
LOX.IMVI	SKCM	Tumor	7.41
UACC.257	SKCM	Tumor	4.48
IGR.37	SKCM	Tumor	6.48
Hs.852.T	SKCM	Tumor	11.03
Hs.934.T	SKCM	Tumor	10.17
HCC2157	BRCA	Tumor	7.51
Hs.739.T	BRCA	Tumor	7.96
HCC38	BRCA	Tumor	15.85
DU4475	BRCA	Tumor	11.71
MDA.MB.1	BRCA	Tumor	8.88
T.47D	BRCA	Tumor	8.37
Hs.742.T	BRCA	Tumor	5.14
KPL.1	BRCA	Tumor	6.92
MDA.MB.2	BRCA	Tumor	12
HCC2218	BRCA	Tumor	5.39
HCC1954	BRCA	Tumor	13.73
CAMA.1	BRCA	Tumor	8.28
HMC.1.8	BRCA	Tumor	3.75
AU565	BRCA	Tumor	14.88
Hs.578T	BRCA	Tumor	7.65
BT.20	BRCA	Tumor	18.99
MDA.MB.3	BRCA	Tumor	11.14
MDA.MB.4	BRCA	Tumor	15.49
SK.BR.3	BRCA	Tumor	10.83
HCC1143	BRCA	Tumor	5.96
HCC1937	BRCA	Tumor	8.6
HCC1569	BRCA	Tumor	8.56
HCC1395	BRCA	Tumor	16.33
HCC1187	BRCA	Tumor	10.22
ZR.75.1	BRCA	Tumor	13.83
HCC70	BRCA	Tumor	6.47

MCF7	BRCA	Tumor	10.01
HCC202	BRCA	Tumor	9.13
MDA.MB.1	BRCA	Tumor	10.31
EFM.192A	BRCA	Tumor	7.16
MDA.MB.4	BRCA	Tumor	8.29
EFM.19	BRCA	Tumor	11.92
BT.474	BRCA	Tumor	9.14
Hs.606.T	BRCA	Tumor	9.27
MDA.MB.4	BRCA	Tumor	7.77
MDA.MB.4	BRCA	Tumor	9.11
BT.549	BRCA	Tumor	9.68
CAL.85.1	BRCA	Tumor	5.05
HMEL	BRCA	Tumor	10.29
HCC1806	BRCA	Tumor	5.78
UACC.812	BRCA	Tumor	3.28
CAL.148	BRCA	Tumor	7.35
MDA.MB.1	BRCA	Tumor	13.81
HCC1500	BRCA	Tumor	9.04
HCC1599	BRCA	Tumor	4.75
UACC.893	BRCA	Tumor	14.53
HDQ.P1	BRCA	Tumor	4.51
HCC1419	BRCA	Tumor	11.92
BT.483	BRCA	Tumor	13.57
Hs.281.T	BRCA	Tumor	9.34
HCC1428	BRCA	Tumor	9.01
Hs.274.T	BRCA	Tumor	12.15
ZR.75.30	BRCA	Tumor	9.25
Hs.343.T	BRCA	Tumor	9.11
CAL.51	BRCA	Tumor	11.42
CAL.120	BRCA	Tumor	11.44
JIMT.1	BRCA	Tumor	7.13
SNU.449	LIHC	Tumor	7.38
JHH.7	LIHC	Tumor	10.2
HuH.6	LIHC	Tumor	5.95
SNU.761	LIHC	Tumor	5.56
Li.7	LIHC	Tumor	8.35
HLF	LIHC	Tumor	9.84
JHH.4	LIHC	Tumor	6.65
JHH.1	LIHC	Tumor	6.79
JHH.5	LIHC	Tumor	9.44
HuH.7	LIHC	Tumor	7.33
SNU.182	LIHC	Tumor	10.1
SNU.398	LIHC	Tumor	11.97
SNU.423	LIHC	Tumor	8.67
SNU.387	LIHC	Tumor	5.37
SNU.475	LIHC	Tumor	9.38
JHH.6	LIHC	Tumor	7.32
SK.HEP.1	LIHC	Tumor	7.42
SNU.886	LIHC	Tumor	15.94
SNU.878	LIHC	Tumor	12.99
NCI.H684	LIHC	Tumor	9.59
Hep.3B2.1	LIHC	Tumor	13.18
PLC.PRF.5	LIHC	Tumor	9.11
Hep.G2	LIHC	Tumor	7.54
huH.1	LIHC	Tumor	6.11
JHH.2	LIHC	Tumor	6.44
NCI.H28	MESO	Tumor	4.4
MSTO.211	MESO	Tumor	13.65
IST.MES1	MESO	Tumor	9.37

ACC.MESO	MESO	Tumor	6.2
NCI.H2052	MESO	Tumor	13.21
NCI.H2452	MESO	Tumor	8.11
MPP.89	MESO	Tumor	11.47
IST.MES2	MESO	Tumor	13.39
JL.1	MESO	Tumor	7.5
OV56	OV	Tumor	8.24
JHOS.4	OV	Tumor	6.26
RMUG.S	OV	Tumor	6.31
COV318	OV	Tumor	5.51
JHOM.1	OV	Tumor	9.96
JHOC.5	OV	Tumor	8.96
OVK18	OV	Tumor	7.81
SNU.119	OV	Tumor	7.02
JHOM.2B	OV	Tumor	10.59
OV.90	OV	Tumor	5.07
OVTOKO	OV	Tumor	6.21
OVCAR.4	OV	Tumor	3.8
Caov.4	OV	Tumor	7.23
SK.OV.3	OV	Tumor	10.32
NIH.OVCA	OV	Tumor	12.77
COV362	OV	Tumor	4.1
COV434	OV	Tumor	10.91
COV644	OV	Tumor	6.25
OV7	OV	Tumor	3.94
OAW42	OV	Tumor	6.86
OAW28	OV	Tumor	7.28
OVCAR.8	OV	Tumor	12.86
OVMANA	OV	Tumor	3.06
X59M	OV	Tumor	7.06
KURAMOC	OV	Tumor	7.2
SNU.840	OV	Tumor	6.41
SNU.8	OV	Tumor	5.36
JHOS.2	OV	Tumor	5.47
OC.314	OV	Tumor	7.19
Caov.3	OV	Tumor	9.99
MCAS	OV	Tumor	6.52
EFO.27	OV	Tumor	8.22
A2780	OV	Tumor	9.94
IGROV1	OV	Tumor	8.79
TOV.21G	OV	Tumor	8.16
OVSAHO	OV	Tumor	5.67
FU.OV.1	OV	Tumor	5.39
Hey.A8	OV	Tumor	12.25
TYK.nu	OV	Tumor	7.18
OVISE	OV	Tumor	5.01
OVKATE	OV	Tumor	9.76
ES.2	OV	Tumor	4.8
TOV.112D	OV	Tumor	10.75
EFO.21	OV	Tumor	8.67
RMG.I	OV	Tumor	6.53
OELE	OV	Tumor	12.74
ONCO.DG	OV	Tumor	6.29
KYSE.450	ESCA	Tumor	14.72
TE.10	ESCA	Tumor	6.83
TE.6	ESCA	Tumor	11.16
TE.4	ESCA	Tumor	8.33
EC.GI.10	ESCA	Tumor	7.34
TE.14	ESCA	Tumor	9.7

TE.8	ESCA	Tumor	18.13
OE33	ESCA	Tumor	5.94
KYSE.410	ESCA	Tumor	8.39
KYSE.140	ESCA	Tumor	6.14
KYSE.180	ESCA	Tumor	9.34
TE.9	ESCA	Tumor	17.53
KYSE.520	ESCA	Tumor	7.95
KYSE.270	ESCA	Tumor	4.71
NA..3	ESCA	Tumor	10.56
KYSE.70	ESCA	Tumor	5.46
TE.11	ESCA	Tumor	3.2
TE.1	ESCA	Tumor	13.67
TE.5	ESCA	Tumor	8.5
TE.15	ESCA	Tumor	9.54
OE19	ESCA	Tumor	8.94
KYSE.510	ESCA	Tumor	8.71
KYSE.30	ESCA	Tumor	5.74
KYSE.150	ESCA	Tumor	11.37
JH.EsoAd1	ESCA	Tumor	9.18
COLO.680	ESCA	Tumor	9.38
OE21	ESCA	Tumor	3
KLE	UCEC	Tumor	6.55
SNG.M	UCEC	Tumor	8.08
HEC.151	UCEC	Tumor	8.63
HEC.1.B	UCEC	Tumor	4.74
SNU.1077	UCEC	Tumor	4.23
JHUEM.3	UCEC	Tumor	8.8
RL95.2	UCEC	Tumor	11.74
MFE.280	UCEC	Tumor	11.14
MFE.319	UCEC	Tumor	8.4
MFE.296	UCEC	Tumor	9.71
EFE.184	UCEC	Tumor	3.04
HEC.108	UCEC	Tumor	8.24
HEC.265	UCEC	Tumor	9.09
HEC.6	UCEC	Tumor	14.14
HEC.50B	UCEC	Tumor	6.88
TEN	UCEC	Tumor	7.91
JHUEM.1	UCEC	Tumor	8.71
HEC.251	UCEC	Tumor	11.74
Ishikawa..I	UCEC	Tumor	11.72
COLO.684	UCEC	Tumor	8.5
SNU.685	UCEC	Tumor	5.82
HEC.59	UCEC	Tumor	8.97
EN	UCEC	Tumor	9.18
ESS.1	UCEC	Tumor	15.78
HEC.1.A	UCEC	Tumor	10.3
JHUEM.2	UCEC	Tumor	11.89
AN3.CA	UCEC	Tumor	11.19
JHUEM.7	UCEC	Tumor	7.63
LN.229	GBM	Tumor	9.53
SF126	GBM	Tumor	12.6
TM.31	GBM	Tumor	28.89
SF.295	GBM	Tumor	8.72
M059K	GBM	Tumor	11.06
GOS.3	GBM	Tumor	13.11
KNS.60	GBM	Tumor	9.41
KNS.81	GBM	Tumor	10.5
KG.1.C	GBM	Tumor	13.98
AM.38	GBM	Tumor	10.55

CAS.1	GBM	Tumor	7.46
YH.13	GBM	Tumor	15.87
DK.MG	GBM	Tumor	14.84
U.118.MG	GBM	Tumor	18.06
GB.1	GBM	Tumor	9.33
SNU.489	GBM	Tumor	6.33
SNU.201	GBM	Tumor	8.34
SNU.466	GBM	Tumor	5.85
SNU.1105	GBM	Tumor	6.46
SNU.738	GBM	Tumor	6.87
SNU.626	GBM	Tumor	11.85
CCF.STTG1	GBM	Tumor	8.38
SW.1088	GBM	Tumor	17.19
LN.18	GBM	Tumor	15.42
T98G	GBM	Tumor	8.92
DBTRG.05	GBM	Tumor	9.98
A172	GBM	Tumor	10.96
GMS.10	GBM	Tumor	17.69
X42.MG.BA	GBM	Tumor	8.47
YKG1	GBM	Tumor	9.11
U.251.MG	GBM	Tumor	7.52
KS.1	GBM	Tumor	13.68
X8.MG.BA	GBM	Tumor	13.08
U.87.MG	GBM	Tumor	9.79
LN.443	GBM	Tumor	9.12
LN.235	GBM	Tumor	10.73
LN.428	GBM	Tumor	6.42
SF.172	GBM	Tumor	3.94
U343	GBM	Tumor	7.73
LN.215	GBM	Tumor	10.65
LN.464	GBM	Tumor	8.04
U.178	GBM	Tumor	13.44
A1207	GBM	Tumor	7.97
LN.319	GBM	Tumor	9.2
LN382	GBM	Tumor	10.01
LN.340	GBM	Tumor	9
SF268	GBM	Tumor	6.01
SNB75	GBM	Tumor	9.97
PA.TU.898	PAAD	Tumor	5.26
SW.1990	PAAD	Tumor	6.32
PANC.1	PAAD	Tumor	5.82
HPAC	PAAD	Tumor	7.86
SU.86.86	PAAD	Tumor	8.41
Panc.02.03	PAAD	Tumor	5.96
SNU.324	PAAD	Tumor	9.25
SNU.213	PAAD	Tumor	9.33
PA.TU.890	PAAD	Tumor	4.24
DAN.G	PAAD	Tumor	6.26
HUP.T3	PAAD	Tumor	8.45
KP.3	PAAD	Tumor	8.18
KP4	PAAD	Tumor	7
HPAF.II	PAAD	Tumor	7.52
Capan.1	PAAD	Tumor	6.43
CFPAC.1	PAAD	Tumor	11.77
Panc.02.13	PAAD	Tumor	7.59
BxPC.3	PAAD	Tumor	6.52
SUIT.2	PAAD	Tumor	11.32
YAPC	PAAD	Tumor	15.42
SNU.410	PAAD	Tumor	7.65

T3M.4	PAAD	Tumor	10.64
PK.45H	PAAD	Tumor	6.04
Panc.08.13	PAAD	Tumor	7.59
PK.1	PAAD	Tumor	8.57
Panc.05.04	PAAD	Tumor	10.41
MIA.PaCa.	PAAD	Tumor	3.38
Capan.2	PAAD	Tumor	13.24
Hs.766T	PAAD	Tumor	8.08
Panc.10.05	PAAD	Tumor	6.39
AsPC.1	PAAD	Tumor	6.72
L3.3	PAAD	Tumor	6.93
KP.2	PAAD	Tumor	10.63
Panc.04.03	PAAD	Tumor	13.49
PK.59	PAAD	Tumor	4.25
QGP.1	PAAD	Tumor	9.85
Panc.03.27	PAAD	Tumor	6.59
TCC.PAN2	PAAD	Tumor	8.25
HUP.T4	PAAD	Tumor	6.89
PSN1	PAAD	Tumor	8.19
PA.TU.898	PAAD	Tumor	9.45
NH.6	NB	Tumor	20.57
KP.N.RT.BI	NB	Tumor	16.75
KP.N.SI9s	NB	Tumor	5.43
SK.N.SH	NB	Tumor	12.44
SK.N.FI	NB	Tumor	18.85
SK.N.AS	NB	Tumor	21.67
IMR.32	NB	Tumor	13.68
KP.N.YN	NB	Tumor	11.94
SK.N.BE.2.	NB	Tumor	12.84
SK.N.DZ	NB	Tumor	11.01
KELLY	NB	Tumor	11.24
CHP.212	NB	Tumor	15.6
MHH.NB.1	NB	Tumor	17.84
CHP.126	NB	Tumor	13
NB.1	NB	Tumor	12.44
SIMA	NB	Tumor	10.62
RERF.LC.A	LUAD	Tumor	5.79
NCI.H2347	LUAD	Tumor	12.36
NCI.H2087	LUAD	Tumor	8.14
NCI.H2085	LUAD	Tumor	6.19
NCI.H1573	LUAD	Tumor	11.79
NCI.H1651	LUAD	Tumor	7.25
NCI.H1666	LUAD	Tumor	5.58
NCI.H1734	LUAD	Tumor	11.6
NCI.H2342	LUAD	Tumor	9.81
NCI.H322	LUAD	Tumor	7.88
Hs.618.T	LUAD	Tumor	10
NCI.H2291	LUAD	Tumor	7.41
COR.L105	LUAD	Tumor	9.34
HCC.2279	LUAD	Tumor	6.31
NCI.H838	LUAD	Tumor	11.3
NCI.H2228	LUAD	Tumor	15.18
Calu.6	LUAD	Tumor	8.03
SK.LU.1	LUAD	Tumor	5.89
PC.14	LUAD	Tumor	5.38
RERF.LC.M	LUAD	Tumor	6.9
MOR.CPR	LUAD	Tumor	5.82
NCI.H1568	LUAD	Tumor	10.48
NCI.H1563	LUAD	Tumor	9.73

NCI.H358	LUAD	Tumor	16.56
NCI.H165(	LUAD	Tumor	10.47
NCI.H179:	LUAD	Tumor	10.11
NCI.H143	LUAD	Tumor	5.04
NCI.H179	LUAD	Tumor	10.36
NCI.H135	LUAD	Tumor	13.34
HCC.2108	LUAD	Tumor	11.45
NCI.H596	LUAD	Tumor	10.86
NCI.H650	LUAD	Tumor	10.78
SW.1573	LUAD	Tumor	8.9
HCC.366	LUAD	Tumor	8.65
HCC.1833	LUAD	Tumor	6.48
NCI.H325	LUAD	Tumor	9.4
HCC.1195	LUAD	Tumor	3.67
NCI.H854	LUAD	Tumor	4.74
NCI.H202:	LUAD	Tumor	10.78
DV.90	LUAD	Tumor	8.68
NCI.H164	LUAD	Tumor	7.68
NCI.H23	LUAD	Tumor	2.94
NCI.H137:	LUAD	Tumor	9.32
NCI.H162:	LUAD	Tumor	6.62
Hs.229.T	LUAD	Tumor	12.73
NCI.H183	LUAD	Tumor	9.86
NCI.H175	LUAD	Tumor	11.2
RERF.LC.K	LUAD	Tumor	7.8
NCI.H169:	LUAD	Tumor	8.48
NCI.H441	LUAD	Tumor	12.8
NCI.H203(	LUAD	Tumor	6.38
NCI.H212	LUAD	Tumor	5.63
A549	LUAD	Tumor	7.65
NCI.H197	LUAD	Tumor	7.79
NCI.H647	LUAD	Tumor	8.91
HCC.44	LUAD	Tumor	5.32
NCI.H522	LUAD	Tumor	12.55
HCC.78	LUAD	Tumor	7.73
RERF.LC.A	LUAD	Tumor	12.4
ABC.1	LUAD	Tumor	9.72
NCI.H139	LUAD	Tumor	3.38
NCI.H178	LUAD	Tumor	9
NCI.H212(	LUAD	Tumor	12.62
HCC827	LUAD	Tumor	9.36
LXF.289	LUAD	Tumor	9.74
Calu.3	LUAD	Tumor	4.86
NCI.H143	LUAD	Tumor	6.48
NCI.H200	LUAD	Tumor	8.06
HCC4006	LUAD	Tumor	8.37
NCI.H194	LUAD	Tumor	12.11
NCI.H240	LUAD	Tumor	12.66
NCI.H207:	LUAD	Tumor	4.18
HOP.62	LUAD	Tumor	12.85
EKVX	LUAD	Tumor	9.02
NA..4	LUAD	Tumor	8.3
NA..5	LUAD	Tumor	5.92
RERF.GC.1	STAD	Tumor	10.94
Hs.746T	STAD	Tumor	11.07
LMSU	STAD	Tumor	7.77
ECC10	STAD	Tumor	8.16
TGBC11Tk	STAD	Tumor	16.71
SNU.520	STAD	Tumor	11.83

GSS	STAD	Tumor	7.32
SNU.620	STAD	Tumor	9.09
ECC12	STAD	Tumor	11
GSU	STAD	Tumor	6.56
FU97	STAD	Tumor	10.52
GCIY	STAD	Tumor	14.57
SH.10.TC	STAD	Tumor	6.8
MKN1	STAD	Tumor	16.32
MKN74	STAD	Tumor	18.53
KE.39	STAD	Tumor	8.61
HGC.27	STAD	Tumor	7.96
HuG1.N	STAD	Tumor	5.55
NUGC.4	STAD	Tumor	7.21
SNU.16	STAD	Tumor	7.03
NCI.N87	STAD	Tumor	9.91
OCUM.1	STAD	Tumor	8.35
SNU.5	STAD	Tumor	7.52
SNU.601	STAD	Tumor	7.52
SNU.668	STAD	Tumor	4.31
NCC.StC.K	STAD	Tumor	11.09
SNU.719	STAD	Tumor	9.23
SNU.216	STAD	Tumor	7.59
NUGC.2	STAD	Tumor	5.57
SNU.1	STAD	Tumor	10.65
AGS	STAD	Tumor	13.06
KATO.III	STAD	Tumor	9.06
NUGC.3	STAD	Tumor	11.15
IM95	STAD	Tumor	8.41
MKN7	STAD	Tumor	8.32
MKN.45	STAD	Tumor	13.98
X23132.87	STAD	Tumor	13.47
Caki.1	KIRC	Tumor	8.1
TUHR4TKE	KIRC	Tumor	12.36
RCC10RGE	KIRC	Tumor	4.61
OS.RC.2	KIRC	Tumor	6.3
SNU.1272	KIRC	Tumor	5.83
TUHR14TK	KIRC	Tumor	4.4
TUHR10TK	KIRC	Tumor	10.42
KMRC.20	KIRC	Tumor	9.47
VMRC.RCV	KIRC	Tumor	8.68
A.498	KIRC	Tumor	7.74
A.704	KIRC	Tumor	5.82
SNU.349	KIRC	Tumor	7.12
KMRC.3	KIRC	Tumor	14.46
X769.P	KIRC	Tumor	8.33
X786.O	KIRC	Tumor	9.95
VMRC.RCZ	KIRC	Tumor	8.13
ACHN	KIRC	Tumor	11.45
Caki.2	KIRC	Tumor	7.3
KMRC.2	KIRC	Tumor	7.08
KMRC.1	KIRC	Tumor	6.81
SLR.23	KIRC	Tumor	7.43
SLR.24	KIRC	Tumor	4.67
SLR.25	KIRC	Tumor	9.65
HEK.TE	KIRC	Tumor	11.15
SLR.26	KIRC	Tumor	11.17
SK.RC.20	KIRC	Tumor	7.86
SLR.21	KIRC	Tumor	9.21
SLR.20	KIRC	Tumor	9.53

UOK101	KIRC	Tumor	6.99
CAL.54	KIRC	Tumor	8.16
BFTC.909	KIRC	Tumor	5.42
UO.31	KIRC	Tumor	10.07
NA..6	KIRC	Tumor	9.2
LU65	NSC	Tumor	8.86
NCI.H1915	NSC	Tumor	4.18
HCC.1171	NSC	Tumor	12.25
NCI.H810	NSC	Tumor	10.54
NCI.H460	NSC	Tumor	5.36
LU99	NSC	Tumor	10.94
CAL.12T	NSC	Tumor	7.54
COR.L23	NSC	Tumor	4.84
IA.LM	NSC	Tumor	10.36
HCC.1438	NSC	Tumor	14.96
HCC.1359	NSC	Tumor	7.99
NCI.H211(	NSC	Tumor	7.61
ChaGo.K.1	NSC	Tumor	13.88
NCI.H2172	NSC	Tumor	9.69
NCI.H292	NSC	Tumor	8.49
T3M.10	NSC	Tumor	12.63
NCI.H661	NSC	Tumor	8.56
NCI.H1155	NSC	Tumor	14.31
NCI.H1581	NSC	Tumor	5.88
NCI.H2106	NSC	Tumor	14.75
NCI.H2444	NSC	Tumor	5.59
NCI.H1299	NSC	Tumor	2.35
HCC.2814	NSC	Tumor	5.89
HCC364	NSC	Tumor	10.1
HCC827.G	NSC	Tumor	6.82
BEN	NSC	Tumor	6.12
HCC2935	NSC	Tumor	10.09
LCLC.103H	NSC	Tumor	10.39
LCLC.97TM	NSC	Tumor	10.78
HOP.92	NSC	Tumor	4.96
NA..7	NSC	Tumor	8.64
SUP.T1	ALL	Tumor	22.41
SUP.B15	ALL	Tumor	17.08
TALL.1	ALL	Tumor	11.92
RS4.11	ALL	Tumor	2.31
HH	ALL	Tumor	5.37
PEER	ALL	Tumor	19.92
Loucy	ALL	Tumor	21.22
Reh	ALL	Tumor	13.46
SEM	ALL	Tumor	16.04
BDCM	ALL	Tumor	9.81
MOLT.13	ALL	Tumor	17.39
ALL.SIL	ALL	Tumor	10.76
KASUMI.2	ALL	Tumor	12.89
DND.41	ALL	Tumor	19.1
NALM.19	ALL	Tumor	12.96
HPB.ALL	ALL	Tumor	12.28
NALM.6	ALL	Tumor	14.26
PF.382	ALL	Tumor	22.9
MHH.CALI	ALL	Tumor	15.17
KE.37	ALL	Tumor	11.72
MHH.CALI	ALL	Tumor	13.59
MUTZ.5	ALL	Tumor	12.03
KOPN.8	ALL	Tumor	8.24

RPMI.8402	ALL	Tumor	12.96
P12.ICHIK/	ALL	Tumor	9.86
JURKAT	ALL	Tumor	14.72
X697	ALL	Tumor	7
MHH.CALI	ALL	Tumor	11.5
MOLT.16	ALL	Tumor	13.75
RCH.ACV	ALL	Tumor	7.64
MOLT.3	ALL	Tumor	11.61
NCI.H226	LUSC	Tumor	7.25
EBC.1	LUSC	Tumor	8.62
RERF.LC.A	LUSC	Tumor	11.58
SK.MES.1	LUSC	Tumor	11.51
NCI.H520	LUSC	Tumor	7.5
HCC.15	LUSC	Tumor	7.92
SW.900	LUSC	Tumor	7.42
LUDLU.1	LUSC	Tumor	7.59
HARA	LUSC	Tumor	14.09
KNS.62	LUSC	Tumor	4.72
EPLC.272H	LUSC	Tumor	7.67
HCC.95	LUSC	Tumor	7.54
LC.1F	LUSC	Tumor	5.92
LOU.NH91	LUSC	Tumor	5.05
LK.2	LUSC	Tumor	7.08
RERF.LC.S(	LUSC	Tumor	8.99
HLF.a	LUSC	Tumor	8.71
NCI.H1703	LUSC	Tumor	8.21
Calu.1	LUSC	Tumor	12.44
NCI.H217(	LUSC	Tumor	10.93
Sq.1	LUSC	Tumor	12.6
NCI.H1385	LUSC	Tumor	7.51
NCI.H1869	LUSC	Tumor	5.87
SW.1783	LGG	Tumor	10.46
GI.1	LGG	Tumor	8.36
KALS.1	LGG	Tumor	8.96
H4	LGG	Tumor	9.58
NMC.G1	LGG	Tumor	12.45
KNS.42	LGG	Tumor	7.84
Hs.683	LGG	Tumor	9.24
GAMG	LGG	Tumor	9.22
LNZ308	LGG	Tumor	9.7
SF539	LGG	Tumor	7.1
BICR.56	HNSC	Tumor	4.52
BICR.16	HNSC	Tumor	3.76
BICR.6	HNSC	Tumor	2.53
BHY	HNSC	Tumor	8.44
SNU.1214	HNSC	Tumor	14.28
BICR.22	HNSC	Tumor	2.96
BICR.18	HNSC	Tumor	7.12
YD.15	HNSC	Tumor	8.84
YD.10B	HNSC	Tumor	5.4
HSC.2	HNSC	Tumor	4.72
HSC.3	HNSC	Tumor	9.89
HSC.4	HNSC	Tumor	6.37
FaDu	HNSC	Tumor	9.23
CAL.27	HNSC	Tumor	6.98
SCC.9	HNSC	Tumor	9.16
SCC.4	HNSC	Tumor	8.25
SNU.1066	HNSC	Tumor	8.43
SNU.1041	HNSC	Tumor	11.7

BICR.31	HNSC	Tumor	6.1
PE.CA.PJ1!	HNSC	Tumor	5.11
PE.CA.PJ4!	HNSC	Tumor	6.02
PE.CA.PJ3	HNSC	Tumor	3.79
PE.CA.PJ4	HNSC	Tumor	3.73
A.253	HNSC	Tumor	6.89
CAL.33	HNSC	Tumor	11.16
SNU.1076	HNSC	Tumor	8.12
YD.38	HNSC	Tumor	7.6
SNU.46	HNSC	Tumor	8.41
YD.8	HNSC	Tumor	15.14
SNU.899	HNSC	Tumor	5.3
Detroit.56	HNSC	Tumor	6.26
SCC.25	HNSC	Tumor	3.84
SCC.15	HNSC	Tumor	6.45
FTC.238	THCA	Tumor	9.52
FTC.133	THCA	Tumor	5.23
SW579	THCA	Tumor	9.98
TT	THCA	Tumor	16.92
X8305C	THCA	Tumor	6.21
X8505C	THCA	Tumor	8.78
CAL.62	THCA	Tumor	7.58
BHT.101	THCA	Tumor	7.09
B.CPAP	THCA	Tumor	10.7
TT2609.C(	THCA	Tumor	10
ML.1	THCA	Tumor	9.17
NCO2	LCML	Tumor	12
MEG.01	LCML	Tumor	7.62
K.562	LCML	Tumor	9.34
KYO.1	LCML	Tumor	4.71
MOLM.6	LCML	Tumor	6.07
LAMA.84	LCML	Tumor	10.25
CML.T1	LCML	Tumor	12.69
BV.173	LCML	Tumor	9.76
NALM.1	LCML	Tumor	12.34
JURL.MK1	LCML	Tumor	13.07
JK.1	LCML	Tumor	8.36
KU812	LCML	Tumor	16.97
KCL.22	LCML	Tumor	11.56
EM.2	LCML	Tumor	15.49
D283.Med	MB	Tumor	8.4
Daoy	MB	Tumor	8.85
D341.Med	MB	Tumor	8.2
ONS.76	MB	Tumor	4.84
NCI.H660	PRAD	Tumor	11.6
VCaP	PRAD	Tumor	10.51
MDA.PCa.	PRAD	Tumor	14.01
DU.145	PRAD	Tumor	11.01
LNCaP.clo	PRAD	Tumor	6.91
X22Rv1	PRAD	Tumor	13.52
PC.3	PRAD	Tumor	4.42
PrEC.LH	PRAD	Tumor	9.87
EWS502	Ewings_sa	Tumor	8.4
EW8	Ewings_sa	Tumor	10.05
NA..8	Ewings_sa	Tumor	16.89
SF767	CESC	Tumor	8.56
NA..9	CESC	Tumor	9.42
NA..10	CESC	Tumor	7.63
EHEB	CLL	Tumor	8.67

JVM.3	CLL	Tumor	8.73
JVM.2	CLL	Tumor	8.49
MEC.1	CLL	Tumor	8.72

Supplementary Table 9

Genes	Gene1	Gene2	cor	p.value	pstar
cor.result.r	METTL14	SPI1	-0.05928	0.367701	
cor.result.r	METTL14	MSX1	-0.09108	0.165825	
cor.result.r	METTL14	MSX2	0.011648	0.859628	
cor.result.r	METTL14	LHX9	0.057142	0.385258	
cor.result.r	METTL14	RAX2	-0.0381	0.562851	
cor.result.r	METTL14	SHOX	0.041135	0.532111	
cor.result.r	METTL14	MZF1	0.054435	0.408208	
cor.result.r	METTL14	ETS1	-0.22976	0.000407	**
cor.result.r	METTL14	YY1	0.248865	0.000124	**
cor.result.r	METTL14	FOXC2	-0.07928	0.227974	
cor.result.r	METTL14	GATA2	-0.1311	0.0456	*

Genes	Gene1	Gene2	cor	p.value	pstar
cor.result.r	METTL14	SPI1	-0.10328	0.010384	*
cor.result.r	METTL14	GATA2	-0.02123	0.599307	
cor.result.r	METTL14	FOXC2	-0.05385	0.182273	
cor.result.r	METTL14	YY1	0.3203	3.87E-16	**
cor.result.r	METTL14	ETS1	-0.0913	0.002356	**
cor.result.r	METTL14	MZF1	0.013492	0.738443	
cor.result.r	METTL14	SHOX	-0.0202	0.617159	
cor.result.r	METTL14	RAX2	0.085548	0.033914	*
cor.result.r	METTL14	LHX9	0.110964	0.587393	
cor.result.r	METTL14	MSX2	0.008776	0.828048	
cor.result.r	METTL14	MSX1	-0.11443	0.044919	*

Supplementary Table 10

rownames	Type	values	times	status
TARGET-3	G1(n=31)	9.15499	NA	NA
TARGET-3	G1(n=31)	9.28712	NA	NA
TARGET-3	G1(n=31)	9.19001	NA	NA
TARGET-3	G1(n=31)	9.20485	NA	NA
TARGET-3	G1(n=31)	9.22739	NA	NA
TARGET-3	G1(n=31)	9.61371	NA	NA
TARGET-3	G1(n=31)	9.14768	NA	NA
TARGET-3	G1(n=31)	9.02851	NA	NA
TARGET-3	G1(n=31)	9.06764	NA	NA
TARGET-3	G1(n=31)	8.98418	NA	NA
TARGET-3	G1(n=31)	8.96496	NA	NA
TARGET-3	G1(n=31)	9.65431	NA	NA
TARGET-3	G1(n=31)	9.17787	NA	NA
TARGET-3	G1(n=31)	9.34139	NA	NA
TARGET-3	G1(n=31)	9.86771	NA	NA
TARGET-3	G1(n=31)	8.83076	NA	NA
TARGET-3	G1(n=31)	9.03367	NA	NA
TARGET-3	G1(n=31)	9.00875	NA	NA
TARGET-3	G1(n=31)	9.1197	NA	NA
TARGET-3	G1(n=31)	9.0436	NA	NA
TARGET-3	G1(n=31)	9.14732	NA	NA
TARGET-3	G1(n=31)	9.58387	NA	NA
TARGET-3	G1(n=31)	9.24664	NA	NA
TARGET-3	G1(n=31)	8.97371	NA	NA
TARGET-3	G1(n=31)	8.89632	NA	NA
TARGET-3	G1(n=31)	9.19332	NA	NA
TARGET-3	G1(n=31)	9.26206	NA	NA
TARGET-3	G1(n=31)	9.089	NA	NA
TARGET-3	G1(n=31)	9.39295	NA	NA
TARGET-3	G1(n=31)	9.20843	NA	NA
TARGET-3	G1(n=31)	9.35662	NA	NA
TARGET-3	G2(n=212	9.58247	NA	NA
TARGET-3	G2(n=212	9.64998	NA	NA
TARGET-3	G2(n=212	9.5601	NA	NA
TARGET-3	G2(n=212	9.59337	NA	NA
TARGET-3	G2(n=212	9.31482	NA	NA
TARGET-3	G2(n=212	9.19497	NA	NA
TARGET-3	G2(n=212	9.41798	NA	NA
TARGET-3	G2(n=212	9.22512	NA	NA
TARGET-3	G2(n=212	9.45056	NA	NA
TARGET-3	G2(n=212	9.25832	NA	NA
TARGET-3	G2(n=212	9.26717	NA	NA
TARGET-3	G2(n=212	9.32992	NA	NA
TARGET-3	G2(n=212	9.18237	NA	NA
TARGET-3	G2(n=212	9.39707	NA	NA
TARGET-3	G2(n=212	9.15186	NA	NA
TARGET-3	G2(n=212	9.19095	NA	NA
TARGET-3	G2(n=212	9.55604	NA	NA
TARGET-3	G2(n=212	9.63902	NA	NA
TARGET-3	G2(n=212	9.38458	NA	NA
TARGET-3	G2(n=212	9.54998	NA	NA
TARGET-3	G2(n=212	9.52726	NA	NA
TARGET-3	G2(n=212	9.16948	NA	NA
TARGET-3	G2(n=212	9.34361	NA	NA
TARGET-3	G2(n=212	9.58811	NA	NA
TARGET-3	G2(n=212	9.30625	NA	NA
TARGET-3	G2(n=212	9.52515	NA	NA
TARGET-3	G2(n=212	9.37347	NA	NA

TARGET-3	G2(n=212	9.2844	NA	NA
TARGET-3	G2(n=212	9.70453	NA	NA
TARGET-3	G2(n=212	9.63868	NA	NA
TARGET-3	G2(n=212	9.44373	NA	NA
TARGET-3	G2(n=212	9.33407	NA	NA
TARGET-3	G2(n=212	9.27754	NA	NA
TARGET-3	G2(n=212	9.23323	NA	NA
TARGET-3	G2(n=212	9.24659	NA	NA
TARGET-3	G2(n=212	9.3751	NA	NA
TARGET-3	G2(n=212	9.45425	NA	NA
TARGET-3	G2(n=212	9.4058	NA	NA
TARGET-3	G2(n=212	9.46497	NA	NA
TARGET-3	G2(n=212	9.31185	NA	NA
TARGET-3	G2(n=212	9.45104	NA	NA
TARGET-3	G2(n=212	9.1601	NA	NA
TARGET-3	G2(n=212	9.40928	NA	NA
TARGET-3	G2(n=212	9.62686	NA	NA
TARGET-3	G2(n=212	9.19143	NA	NA
TARGET-3	G2(n=212	9.39511	NA	NA
TARGET-3	G2(n=212	9.41628	NA	NA
TARGET-3	G2(n=212	9.47919	NA	NA
TARGET-3	G2(n=212	8.99486	NA	NA
TARGET-3	G2(n=212	9.49917	NA	NA
TARGET-3	G2(n=212	9.18782	NA	NA
TARGET-3	G2(n=212	9.31725	NA	NA
TARGET-3	G2(n=212	9.19123	NA	NA
TARGET-3	G2(n=212	8.6859	NA	NA
TARGET-3	G2(n=212	9.37597	NA	NA
TARGET-3	G2(n=212	8.69278	NA	NA
TARGET-3	G2(n=212	8.99129	NA	NA
TARGET-3	G2(n=212	9.30765	NA	NA
TARGET-3	G2(n=212	9.2649	NA	NA
TARGET-3	G2(n=212	9.02714	NA	NA
TARGET-3	G2(n=212	9.28931	NA	NA
TARGET-3	G2(n=212	9.40195	NA	NA
TARGET-3	G2(n=212	9.17283	NA	NA
TARGET-3	G2(n=212	9.61887	NA	NA
TARGET-3	G2(n=212	9.26176	NA	NA
TARGET-3	G2(n=212	9.26485	NA	NA
TARGET-3	G2(n=212	8.93056	NA	NA
TARGET-3	G2(n=212	9.51299	NA	NA
TARGET-3	G2(n=212	9.62362	NA	NA
TARGET-3	G2(n=212	9.30282	NA	NA
TARGET-3	G2(n=212	9.54217	NA	NA
TARGET-3	G2(n=212	9.29512	NA	NA
TARGET-3	G2(n=212	9.52366	NA	NA
TARGET-3	G2(n=212	9.22775	NA	NA
TARGET-3	G2(n=212	9.64461	NA	NA
TARGET-3	G2(n=212	9.47637	NA	NA
TARGET-3	G2(n=212	9.28417	NA	NA
TARGET-3	G2(n=212	9.59612	NA	NA
TARGET-3	G2(n=212	9.4085	NA	NA
TARGET-3	G2(n=212	9.6314	NA	NA
TARGET-3	G2(n=212	9.404	NA	NA
TARGET-3	G2(n=212	9.26443	NA	NA
TARGET-3	G2(n=212	9.68688	NA	NA
TARGET-3	G2(n=212	9.36484	NA	NA
TARGET-3	G2(n=212	9.3254	NA	NA
TARGET-3	G2(n=212	9.47064	NA	NA

TARGET-3	G2(n=212	9.53171	NA	NA
TARGET-3	G2(n=212	9.36082	NA	NA
TARGET-3	G2(n=212	9.55826	NA	NA
TARGET-3	G2(n=212	9.12049	NA	NA
TARGET-3	G2(n=212	9.04026	NA	NA
TARGET-3	G2(n=212	9.19676	NA	NA
TARGET-3	G2(n=212	9.19862	NA	NA
TARGET-3	G2(n=212	9.49666	NA	NA
TARGET-3	G2(n=212	9.45569	NA	NA
TARGET-3	G2(n=212	9.4376	NA	NA
TARGET-3	G2(n=212	9.33289	NA	NA
TARGET-3	G2(n=212	9.58592	NA	NA
TARGET-3	G2(n=212	9.29466	NA	NA
TARGET-3	G2(n=212	9.59136	NA	NA
TARGET-3	G2(n=212	8.99428	NA	NA
TARGET-3	G2(n=212	9.51433	NA	NA
TARGET-3	G2(n=212	9.44619	NA	NA
TARGET-3	G2(n=212	9.06633	NA	NA
TARGET-3	G2(n=212	9.33342	NA	NA
TARGET-3	G2(n=212	9.91127	NA	NA
TARGET-3	G2(n=212	9.1569	NA	NA
TARGET-3	G2(n=212	8.99661	NA	NA
TARGET-3	G2(n=212	9.39319	NA	NA
TARGET-3	G2(n=212	9.78312	NA	NA
TARGET-3	G2(n=212	9.25043	NA	NA
TARGET-3	G2(n=212	9.41789	NA	NA
TARGET-3	G2(n=212	9.26588	NA	NA
TARGET-3	G2(n=212	9.45261	NA	NA
TARGET-3	G2(n=212	9.08195	NA	NA
TARGET-3	G2(n=212	9.0209	NA	NA
TARGET-3	G2(n=212	9.57293	NA	NA
TARGET-3	G2(n=212	9.01264	NA	NA
TARGET-3	G2(n=212	9.21786	NA	NA
TARGET-3	G2(n=212	9.34449	NA	NA
TARGET-3	G2(n=212	9.09989	NA	NA
TARGET-3	G2(n=212	9.05317	NA	NA
TARGET-3	G2(n=212	9.19258	NA	NA
TARGET-3	G2(n=212	9.09475	NA	NA
TARGET-3	G2(n=212	9.22443	NA	NA
TARGET-3	G2(n=212	8.93373	NA	NA
TARGET-3	G2(n=212	9.83821	NA	NA
TARGET-3	G2(n=212	9.14371	NA	NA
TARGET-3	G2(n=212	9.01703	NA	NA
TARGET-3	G2(n=212	9.59756	NA	NA
TARGET-3	G2(n=212	9.90578	NA	NA
TARGET-3	G2(n=212	9.30227	NA	NA
TARGET-3	G2(n=212	9.22541	NA	NA
TARGET-3	G2(n=212	8.98098	NA	NA
TARGET-3	G2(n=212	9.314	NA	NA
TARGET-3	G2(n=212	9.33433	NA	NA
TARGET-3	G2(n=212	9.12949	NA	NA
TARGET-3	G2(n=212	8.96063	NA	NA
TARGET-3	G2(n=212	9.34345	NA	NA
TARGET-3	G2(n=212	9.34777	NA	NA
TARGET-3	G2(n=212	9.49171	NA	NA
TARGET-3	G2(n=212	9.19587	NA	NA
TARGET-3	G2(n=212	9.51552	NA	NA
TARGET-3	G2(n=212	9.53052	NA	NA
TARGET-3	G2(n=212	9.509	NA	NA

TARGET-3	G2(n=212	9.28155	NA	NA
TARGET-3	G2(n=212	9.09486	NA	NA
TARGET-3	G2(n=212	9.03075	NA	NA
TARGET-3	G2(n=212	9.20316	NA	NA
TARGET-3	G2(n=212	9.03981	NA	NA
TARGET-3	G2(n=212	9.28292	NA	NA
TARGET-3	G2(n=212	9.04348	NA	NA
TARGET-3	G2(n=212	9.63602	NA	NA
TARGET-3	G2(n=212	9.51479	NA	NA
TARGET-3	G2(n=212	9.80887	NA	NA
TARGET-3	G2(n=212	9.42984	NA	NA
TARGET-3	G2(n=212	9.39187	NA	NA
TARGET-3	G2(n=212	9.29858	NA	NA
TARGET-3	G2(n=212	9.49982	NA	NA
TARGET-3	G2(n=212	9.4945	NA	NA
TARGET-3	G2(n=212	9.29298	NA	NA
TARGET-3	G2(n=212	8.85164	NA	NA
TARGET-3	G2(n=212	9.24821	NA	NA
TARGET-3	G2(n=212	9.27078	NA	NA
TARGET-3	G2(n=212	9.39404	NA	NA
TARGET-3	G2(n=212	9.23673	NA	NA
TARGET-3	G2(n=212	9.44492	NA	NA
TARGET-3	G2(n=212	9.49205	NA	NA
TARGET-3	G2(n=212	9.45863	NA	NA
TARGET-3	G2(n=212	9.27604	NA	NA
TARGET-3	G2(n=212	9.33974	NA	NA
TARGET-3	G2(n=212	9.3939	NA	NA
TARGET-3	G2(n=212	9.29757	NA	NA
TARGET-3	G2(n=212	9.25011	NA	NA
TARGET-3	G2(n=212	9.23024	NA	NA
TARGET-3	G2(n=212	9.47035	NA	NA
TARGET-3	G2(n=212	9.44325	NA	NA
TARGET-3	G2(n=212	9.15538	NA	NA
TARGET-3	G2(n=212	9.17893	NA	NA
TARGET-3	G2(n=212	9.48899	NA	NA
TARGET-3	G2(n=212	9.34881	NA	NA
TARGET-3	G2(n=212	9.05301	NA	NA
TARGET-3	G2(n=212	9.39487	NA	NA
TARGET-3	G2(n=212	9.07679	NA	NA
TARGET-3	G2(n=212	9.25129	NA	NA
TARGET-3	G2(n=212	9.37809	NA	NA
TARGET-3	G2(n=212	9.45386	NA	NA
TARGET-3	G2(n=212	9.2695	NA	NA
TARGET-3	G2(n=212	9.34492	NA	NA
TARGET-3	G2(n=212	9.34608	NA	NA
TARGET-3	G2(n=212	9.2581	NA	NA
TARGET-3	G2(n=212	9.39578	NA	NA
TARGET-3	G2(n=212	9.40739	NA	NA
TARGET-3	G2(n=212	9.36185	NA	NA
TARGET-3	G2(n=212	9.44942	NA	NA
TARGET-3	G2(n=212	9.32099	NA	NA
TARGET-3	G2(n=212	9.37229	NA	NA
TARGET-3	G2(n=212	9.13158	NA	NA
TARGET-3	G2(n=212	9.39145	NA	NA
TARGET-3	G2(n=212	9.32938	NA	NA
TARGET-3	G2(n=212	9.12706	NA	NA
TARGET-3	G2(n=212	9.34882	NA	NA
TARGET-3	G2(n=212	9.47491	NA	NA
TARGET-3	G2(n=212	9.2056	NA	NA

TARGET-3	G2(n=212	9.52175	NA	NA
TARGET-3	G2(n=212	9.58887	NA	NA
TARGET-3	G2(n=212	9.49752	NA	NA
TARGET-3	G2(n=212	9.2296	NA	NA
TARGET-3	G2(n=212	9.11618	NA	NA
TARGET-3	G2(n=212	9.37216	NA	NA
TARGET-3	G2(n=212	9.40691	NA	NA
TARGET-3	G2(n=212	9.56187	NA	NA

Supplementary Table 11

Genes	TARGET-3	TARGET-3	TARGET-3	TARGET-3	TARGET-3	TARGET-3	TARGET-3	TARGET-3
METTL14	7.93695	7.78644	8.37832	8.48396	7.85604	8.805	7.88024	8.30731
YY1	9.58247	9.64998	9.63793	9.5601	9.59337	9.31482	9.19497	9.41798

TARGET-3	TARGET-3	TARGET-3	TARGET-3	TARGET-3	TARGET-3	TARGET-3	TARGET-3	TARGET-3
7.39381	7.41424	8.31635	8.05388	7.99366	7.99528	7.6401	8.02303	8.0571
9.22512	9.45056	9.25832	9.26717	9.32992	9.18237	9.39707	9.15186	9.19095

TARGET-3	TARGET-3	TARGET-3	TARGET-3	TARGET-3	TARGET-3	TARGET-3	TARGET-3	TARGET-3
8.59227	7.9189	7.61279	8.59269	8.05916	7.43599	7.42422	8.24315	7.77141
9.55604	9.63902	9.38458	9.54998	9.52726	9.16948	9.34361	9.58811	9.30625

TARGET-3	TARGET-3	TARGET-3	TARGET-3	TARGET-3	TARGET-3	TARGET-3	TARGET-3	TARGET-3
8.14646	7.53679	8.42711	7.39985	7.43276	8.01931	8.1642	7.73073	7.56174
9.52515	9.37347	9.2844	9.70453	9.63868	9.44373	9.33407	9.27754	9.23323

TARGET-3	TARGET-3	TARGET-3	TARGET-3	TARGET-3	TARGET-3	TARGET-3	TARGET-3	TARGET-3
8.27069	7.91549	8.23907	7.52105	8.13648	8.01798	8.2181	7.66111	8.33747
9.24659	9.3751	9.45425	9.4058	9.46497	9.31185	9.45104	9.1601	9.40928

TARGET-3	TARGET-3	TARGET-3	TARGET-3	TARGET-3	TARGET-3	TARGET-3	TARGET-3	TARGET-3
8.14777	7.94385	8.05025	7.57717	8.30145	7.36958	7.98455	7.43371	8.25778
9.62686	9.19143	9.39511	9.41628	9.47919	8.99486	9.49917	9.18782	9.31725

TARGET-3	TARGET-3	TARGET-3	TARGET-3	TARGET-3	TARGET-3	TARGET-3	TARGET-3	TARGET-3
8.05518	6.97472	8.08507	7.42028	7.41019	8.56412	8.10993	6.07873	8.19229
9.19123	8.6859	9.37597	8.69278	8.99129	9.30765	9.2649	9.02714	9.28931

TARGET-3	TARGET-3	TARGET-3	TARGET-3	TARGET-3	TARGET-3	TARGET-3	TARGET-3	TARGET-3
7.7986	8.02696	8.40829	8.5769	7.79179	7.00228	7.3033	8.30711	8.04851
9.40195	9.17283	9.61887	9.26176	9.26485	8.93056	9.15499	9.51299	9.62362

TARGET-3	TARGET-3	TARGET-3	TARGET-3	TARGET-3	TARGET-3	TARGET-3	TARGET-3	TARGET-3
8.11829	7.91723	8.38271	8.2504	7.98046	7.87003	7.73226	8.0263	7.7747
9.30282	9.54217	9.29512	9.52366	9.22775	9.64461	9.47637	9.28417	9.59612

TARGET-3	TARGET-3	TARGET-3	TARGET-3	TARGET-3	TARGET-3	TARGET-3	TARGET-3	TARGET-3
8.35247	7.5172	8.37847	7.78422	7.86023	8.55678	7.36593	7.02268	7.79965
9.4085	9.6314	9.404	9.26443	9.28712	9.68688	9.19001	9.36484	9.20485

TARGET-3	TARGET-3	TARGET-3	TARGET-3	TARGET-3	TARGET-3	TARGET-3	TARGET-3	TARGET-3
7.46975	8.0136	7.95655	8.15103	7.77435	6.99383	7.8815	8.82909	7.94388
9.3254	9.66942	9.47064	9.53171	9.36082	9.55826	9.12049	9.04026	9.19676

TARGET-3	TARGET-3	TARGET-3	TARGET-3	TARGET-3	TARGET-3	TARGET-3	TARGET-3	TARGET-3
8.12314	8.01022	8.44992	7.2822	7.83362	6.98459	7.76037	8.17601	8.84062
9.19862	9.49666	9.45569	9.22739	9.4376	9.33289	9.61371	9.14768	9.58592

TARGET-3	TARGET-3	TARGET-3	TARGET-3	TARGET-3	TARGET-3	TARGET-3	TARGET-3	TARGET-3
7.88618	8.11066	7.26121	8.04093	7.24902	7.85587	8.48692	8.12883	7.76633
9.02851	9.29466	9.06764	9.59136	8.99428	9.51433	9.44619	9.06633	8.98418

TARGET-3	TARGET-3	TARGET-3	TARGET-3	TARGET-3	TARGET-3	TARGET-3	TARGET-3	TARGET-3
7.94033	8.23291	7.6444	8.42465	7.35317	7.73449	8.34898	7.46327	7.08764
8.96496	9.33342	9.91127	9.65431	9.17787	9.1569	8.99661	9.34139	9.39319

TARGET-3	TARGET-3	TARGET-3	TARGET-3	TARGET-3	TARGET-3	TARGET-3	TARGET-3	TARGET-3
7.77668	7.45216	8.49741	8.40893	7.0377	7.78143	7.5937	8.25531	8.32992
9.86771	8.83076	9.78312	9.25043	9.03367	9.00875	9.41789	9.26588	9.45261

TARGET-3	TARGET-3	TARGET-3	TARGET-3	TARGET-3	TARGET-3	TARGET-3	TARGET-3	TARGET-3
7.93784	7.46728	7.14799	7.64017	7.1994	8.03172	7.85844	7.96317	8.28727
9.08195	9.0209	9.1197	9.0436	9.14732	9.58387	9.57293	9.24664	9.01264

TARGET-3	TARGET-3	TARGET-3	TARGET-3	TARGET-3	TARGET-3	TARGET-3	TARGET-3	TARGET-3
7.77198	8.07611	7.81883	7.51091	8.06527	8.12781	7.94284	8.00981	7.74478
8.97371	8.89632	9.21786	9.19332	9.34449	9.09989	9.05317	9.19258	9.26206

TARGET-3	TARGET-3	TARGET-3	TARGET-3	TARGET-3	TARGET-3	TARGET-3	TARGET-3	TARGET-3
7.36118	7.63915	8.19444	8.35266	8.25538	8.28049	7.34179	8.34572	8.23015
9.089	9.09475	9.22443	8.93373	9.83821	9.14371	9.01703	9.59756	9.90578

TARGET-3	TARGET-3	TARGET-3	TARGET-3	TARGET-3	TARGET-3	TARGET-3	TARGET-3	TARGET-3
8.08029	8.25893	7.71163	7.47074	7.3255	7.44297	7.8985	7.3531	7.4123
9.39295	9.30227	9.22541	8.98098	9.314	9.20843	9.33433	9.12949	8.96063

TARGET-3	TARGET-3	TARGET-3	TARGET-3	TARGET-3	TARGET-3	TARGET-3	TARGET-3	TARGET-3
7.29317	7.50362	8.40479	7.52104	7.50386	8.869	7.89437	7.26709	8.16592
9.34345	9.34777	9.49171	9.19587	9.51552	9.53052	9.509	9.28155	9.09486

TARGET-3	TARGET-3	TARGET-3	TARGET-3	TARGET-3	TARGET-3	TARGET-3	TARGET-3	TARGET-3
7.76126	8.25054	7.76178	8.16947	8.55402	8.07696	7.5726	7.91519	7.88826
9.03075	9.20316	9.03981	9.28292	9.04348	9.63602	9.51479	9.80887	9.42984

TARGET-3	TARGET-3	TARGET-3	TARGET-3	TARGET-3	TARGET-3	TARGET-3	TARGET-3	TARGET-3
8.09805	7.10663	8.25877	8.60989	8.25564	8.01204	7.39434	7.78095	8.21045
9.39187	9.29858	9.49982	9.4945	9.29298	8.85164	9.24821	9.27078	9.39404

TARGET-3	TARGET-3	TARGET-3	TARGET-3	TARGET-3	TARGET-3	TARGET-3	TARGET-3	TARGET-3
8.13282	8.11901	8.10323	8.65283	7.19466	7.61585	8.35549	8.26073	8.53678
9.23673	9.44492	9.49205	9.45863	9.27604	9.33974	9.3939	9.29757	9.25011

TARGET-3	TARGET-3	TARGET-3	TARGET-3	TARGET-3	TARGET-3	TARGET-3	TARGET-3	TARGET-3
8.91843	8.16116	8.25725	8.77459	7.2176	8.44909	8.13081	8.18962	7.68844
9.27169	9.23024	9.47035	9.44325	9.15538	9.17893	9.48899	9.34881	9.05301

TARGET-3	TARGET-3	TARGET-3	TARGET-3	TARGET-3	TARGET-3	TARGET-3	TARGET-3	TARGET-3
7.3619	6.87112	7.32842	8.15119	7.96473	7.96038	7.19984	7.07745	7.94934
9.39487	9.07679	9.25129	9.37809	9.45386	9.2695	9.34492	9.34608	9.2581

TARGET-3	TARGET-3	TARGET-3	TARGET-3	TARGET-3	TARGET-3	TARGET-3	TARGET-3	TARGET-3
8.35173	8.24429	7.53384	7.60035	7.17324	8.1187	7.06729	7.77643	7.03863
9.39578	9.40739	9.36185	9.44942	9.32099	9.37229	9.13158	9.39145	9.32938

TARGET-3	TARGET-3	TARGET-3	TARGET-3	TARGET-3	TARGET-3	TARGET-3	TARGET-3	TARGET-3
7.51336	7.87299	7.22368	7.40854	7.93709	8.0821	7.9925	8.6694	8.43161
9.12706	9.34882	9.47491	9.2056	9.52175	9.58887	9.49752	9.2296	9.11618

TARGET-3	TARGET-3	TARGET-3	TARGET-3	TARGET-3	TARGET-3	TARGET-30-PASLGS-01A
7.86297	8.31327	8.23738	8.16011	8.38383	8.01202	8.41612
9.37216	9.40691	9.31793	9.35662	9.56187	9.43924	9.56467

Supplementary Table 12

rownames	Type	values
GSM11084	G1(n=149	7.976306
GSM11084	G1(n=149	8.81762
GSM11084	G1(n=149	7.748515
GSM11084	G1(n=149	7.64364
GSM11084	G1(n=149	8.411873
GSM11084	G1(n=149	7.988971
GSM11084	G1(n=149	7.946041
GSM11084	G1(n=149	8.669923
GSM11084	G1(n=149	8.002658
GSM11084	G1(n=149	6.911677
GSM11084	G1(n=149	7.786424
GSM11084	G1(n=149	8.515457
GSM11084	G1(n=149	7.70725
GSM11084	G1(n=149	7.888056
GSM11084	G1(n=149	7.823052
GSM11085	G1(n=149	8.937046
GSM11085	G1(n=149	8.570031
GSM11085	G1(n=149	7.972847
GSM11085	G1(n=149	7.556325
GSM11085	G1(n=149	8.144472
GSM11085	G1(n=149	9.567911
GSM11085	G1(n=149	9.655367
GSM11085	G1(n=149	7.002624
GSM11085	G1(n=149	8.396277
GSM11085	G1(n=149	8.038046
GSM11085	G1(n=149	7.760252
GSM11085	G1(n=149	7.769689
GSM11085	G1(n=149	8.10285
GSM11085	G1(n=149	6.911134
GSM11085	G1(n=149	8.784109
GSM11085	G1(n=149	9.21742
GSM11085	G1(n=149	7.606449
GSM11085	G1(n=149	7.237743
GSM11085	G1(n=149	7.625811
GSM11085	G1(n=149	7.619966
GSM11085	G1(n=149	7.094558
GSM11086	G1(n=149	8.297618
GSM11086	G1(n=149	7.459593
GSM11086	G1(n=149	8.30234
GSM11086	G1(n=149	8.459744
GSM11086	G1(n=149	7.60829
GSM11086	G1(n=149	7.551586
GSM11086	G1(n=149	8.482136
GSM11086	G1(n=149	7.896403
GSM11086	G1(n=149	7.436617
GSM11086	G1(n=149	8.140729
GSM11086	G1(n=149	7.9755
GSM11086	G1(n=149	7.937102
GSM11086	G1(n=149	7.959579
GSM11086	G1(n=149	7.696203
GSM11086	G1(n=149	7.493168
GSM11086	G1(n=149	8.216568
GSM11086	G1(n=149	7.9755
GSM11086	G1(n=149	7.846308
GSM11086	G1(n=149	8.209557
GSM11086	G1(n=149	8.477036
GSM11086	G1(n=149	8.17361
GSM11086	G1(n=149	7.960458

GSM1108(	G1(n=149	8.269617
GSM1108(	G1(n=149	7.666664
GSM1108(	G1(n=149	9.303394
GSM1108-	G1(n=149	8.297999
GSM1108-	G1(n=149	8.148012
GSM1108-	G1(n=149	9.674408
GSM1108-	G1(n=149	8.474568
GSM1108-	G1(n=149	8.546009
GSM1108-	G1(n=149	8.117229
GSM1108-	G1(n=149	8.004015
GSM1108-	G1(n=149	9.863311
GSM1108-	G1(n=149	8.664579
GSM1108-	G1(n=149	8.909399
GSM1108-	G1(n=149	10.05024
GSM1108-	G1(n=149	8.216127
GSM1108-	G1(n=149	8.529679
GSM1108-	G1(n=149	7.576483
GSM1108-	G1(n=149	8.347477
GSM1108-	G1(n=149	7.600548
GSM1108-	G1(n=149	7.948763
GSM1108-	G1(n=149	8.027615
GSM1108-	G1(n=149	7.708602
GSM1108-	G1(n=149	8.853164
GSM1108-	G1(n=149	7.326164
GSM1108-	G1(n=149	8.908262
GSM1108-	G1(n=149	7.99995
GSM1108-	G1(n=149	8.863787
GSM1108-	G1(n=149	8.451925
GSM1108-	G1(n=149	8.570818
GSM1108-	G1(n=149	7.696203
GSM1108-	G1(n=149	8.5305
GSM1108-	G1(n=149	9.819639
GSM1108-	G1(n=149	8.687097
GSM1108-	G1(n=149	8.527352
GSM1108-	G1(n=149	8.051503
GSM1108-	G1(n=149	8.479137
GSM1108-	G1(n=149	7.917603
GSM1108-	G1(n=149	7.846824
GSM1108-	G1(n=149	7.860107
GSM11088	G1(n=149	8.50168
GSM11088	G1(n=149	8.152773
GSM11088	G1(n=149	8.290717
GSM11088	G1(n=149	8.746519
GSM11088	G1(n=149	7.583884
GSM11088	G1(n=149	8.251842
GSM11088	G1(n=149	7.846308
GSM11088	G1(n=149	7.302492
GSM11088	G1(n=149	7.429996
GSM11088	G1(n=149	7.652376
GSM11088	G1(n=149	8.417679
GSM11088	G1(n=149	7.521779
GSM11088	G1(n=149	7.907454
GSM11088	G1(n=149	7.759818
GSM11088	G1(n=149	6.85712
GSM11088	G1(n=149	7.908451
GSM11088	G1(n=149	7.559227
GSM11088	G1(n=149	8.463149
GSM11088	G1(n=149	9.397885
GSM11088	G1(n=149	7.831845

GSM11088	G1(n=149	7.755054
GSM11088	G1(n=149	8.587371
GSM11088	G1(n=149	8.465177
GSM11088	G1(n=149	7.597739
GSM11088	G1(n=149	7.371303
GSM11088	G1(n=149	7.846308
GSM11088	G1(n=149	8.827662
GSM11088	G1(n=149	7.587691
GSM11089	G1(n=149	8.384607
GSM11089	G1(n=149	7.729538
GSM11089	G1(n=149	8.105581
GSM11089	G1(n=149	8.594596
GSM11089	G1(n=149	9.254384
GSM11089	G1(n=149	8.316248
GSM11089	G1(n=149	8.189485
GSM11089	G1(n=149	7.783102
GSM11089	G1(n=149	7.884763
GSM11089	G1(n=149	8.187293
GSM11089	G1(n=149	8.541769
GSM11089	G1(n=149	8.07764
GSM11089	G1(n=149	8.267972
GSM11089	G1(n=149	7.60455
GSM11089	G1(n=149	7.938906
GSM11089	G1(n=149	8.941055
GSM11090	G1(n=149	7.553242
GSM11090	G1(n=149	9.242091
GSM11090	G1(n=149	8.458929
GSM11090	G1(n=149	8.652953
GSM11090	G1(n=149	8.935477
GSM11090	G1(n=149	9.285604
GSM11090	G1(n=149	8.605427
GSM11090	G1(n=149	8.727314
GSM11084	G2(n=271	6.753723
GSM11084	G2(n=271	7.280044
GSM11084	G2(n=271	7.100665
GSM11084	G2(n=271	8.011964
GSM11084	G2(n=271	8.322098
GSM11084	G2(n=271	10.70099
GSM11084	G2(n=271	7.237345
GSM11084	G2(n=271	7.744649
GSM11084	G2(n=271	8.22415
GSM11084	G2(n=271	7.883791
GSM11084	G2(n=271	8.878174
GSM11084	G2(n=271	7.255656
GSM11084	G2(n=271	8.729324
GSM11084	G2(n=271	6.993351
GSM11084	G2(n=271	8.196727
GSM11084	G2(n=271	7.151805
GSM11084	G2(n=271	6.584124
GSM11084	G2(n=271	8.437526
GSM11084	G2(n=271	8.295842
GSM11084	G2(n=271	8.888525
GSM11084	G2(n=271	6.874594
GSM11084	G2(n=271	9.3089
GSM11084	G2(n=271	8.830604
GSM11084	G2(n=271	7.73641
GSM11084	G2(n=271	8.801827
GSM11084	G2(n=271	8.26528
GSM11084	G2(n=271	7.471381

GSM11084	G2(n=271	7.622116
GSM11084	G2(n=271	8.405008
GSM11084	G2(n=271	7.027497
GSM11084	G2(n=271	8.280166
GSM11084	G2(n=271	8.66611
GSM11084	G2(n=271	8.073794
GSM11084	G2(n=271	6.990121
GSM11084	G2(n=271	9.485171
GSM11084	G2(n=271	7.371901
GSM11084	G2(n=271	7.339167
GSM11084	G2(n=271	7.362743
GSM11084	G2(n=271	7.186206
GSM11084	G2(n=271	7.385777
GSM11084	G2(n=271	9.771804
GSM11084	G2(n=271	8.587277
GSM11084	G2(n=271	11.25277
GSM11084	G2(n=271	11.06747
GSM11084	G2(n=271	7.714467
GSM11084	G2(n=271	8.39345
GSM11084	G2(n=271	9.054083
GSM11084	G2(n=271	7.29967
GSM11084	G2(n=271	7.429158
GSM11085	G2(n=271	7.689482
GSM11085	G2(n=271	7.755388
GSM11085	G2(n=271	7.037755
GSM11085	G2(n=271	8.183678
GSM11085	G2(n=271	9.899045
GSM11085	G2(n=271	7.686624
GSM11085	G2(n=271	8.250786
GSM11085	G2(n=271	9.531224
GSM11085	G2(n=271	7.756346
GSM11085	G2(n=271	7.548358
GSM11085	G2(n=271	6.977385
GSM11085	G2(n=271	6.611363
GSM11085	G2(n=271	7.345889
GSM11085	G2(n=271	6.958484
GSM11085	G2(n=271	7.139054
GSM11085	G2(n=271	9.05866
GSM11085	G2(n=271	7.484815
GSM11085	G2(n=271	7.601232
GSM11085	G2(n=271	7.439675
GSM11085	G2(n=271	9.657057
GSM11085	G2(n=271	8.152283
GSM11085	G2(n=271	8.066139
GSM11085	G2(n=271	7.9298
GSM11085	G2(n=271	8.017573
GSM11085	G2(n=271	7.642753
GSM11085	G2(n=271	7.339642
GSM11085	G2(n=271	8.209898
GSM11085	G2(n=271	8.84452
GSM11085	G2(n=271	7.319205
GSM11085	G2(n=271	8.984699
GSM11085	G2(n=271	8.143535
GSM11085	G2(n=271	8.687454
GSM11085	G2(n=271	8.702527
GSM11085	G2(n=271	8.211188
GSM11085	G2(n=271	8.075518
GSM11085	G2(n=271	7.736905
GSM11085	G2(n=271	7.157691

GSM1108!	G2(n=271	8.210758
GSM1108!	G2(n=271	7.723537
GSM1108!	G2(n=271	7.213556
GSM1108!	G2(n=271	7.554719
GSM1108!	G2(n=271	8.817795
GSM1108(	G2(n=271	6.616209
GSM1108(	G2(n=271	6.075037
GSM1108(	G2(n=271	7.986148
GSM1108(	G2(n=271	7.694278
GSM1108(	G2(n=271	8.342511
GSM1108(	G2(n=271	7.179235
GSM1108(	G2(n=271	7.735501
GSM1108(	G2(n=271	6.884278
GSM1108(	G2(n=271	8.03567
GSM1108(	G2(n=271	7.796757
GSM1108(	G2(n=271	8.718166
GSM1108(	G2(n=271	7.638931
GSM1108(	G2(n=271	7.946741
GSM1108(	G2(n=271	8.195341
GSM1108(	G2(n=271	8.679361
GSM1108(	G2(n=271	7.165496
GSM1108(	G2(n=271	7.268371
GSM1108(	G2(n=271	9.206785
GSM1108(	G2(n=271	8.368758
GSM1108(	G2(n=271	8.790052
GSM1108(	G2(n=271	7.513537
GSM1108(	G2(n=271	7.698159
GSM1108(	G2(n=271	8.700042
GSM1108(	G2(n=271	8.97463
GSM1108(	G2(n=271	8.695603
GSM1108(	G2(n=271	8.004226
GSM1108(	G2(n=271	7.877844
GSM1108(	G2(n=271	7.777438
GSM1108(	G2(n=271	7.995616
GSM1108(	G2(n=271	7.739796
GSM1108(	G2(n=271	7.692799
GSM1108(	G2(n=271	8.163263
GSM1108(	G2(n=271	8.55192
GSM1108(	G2(n=271	7.424152
GSM1108(	G2(n=271	7.598235
GSM1108(	G2(n=271	6.835139
GSM1108(	G2(n=271	8.317733
GSM1108(	G2(n=271	7.71537
GSM1108	G2(n=271	9.036298
GSM1108	G2(n=271	7.946741
GSM1108	G2(n=271	7.965448
GSM1108	G2(n=271	9.332861
GSM1108	G2(n=271	7.759095
GSM1108	G2(n=271	6.832352
GSM1108	G2(n=271	7.679944
GSM1108	G2(n=271	9.490217
GSM1108	G2(n=271	8.147485
GSM1108	G2(n=271	7.625917
GSM1108	G2(n=271	8.292891
GSM1108	G2(n=271	9.391522
GSM1108	G2(n=271	9.705427
GSM1108	G2(n=271	7.135763
GSM1108	G2(n=271	7.709584
GSM1108	G2(n=271	8.911228

GSM1108	G2(n=271	7.849067
GSM1108	G2(n=271	7.65461
GSM1108	G2(n=271	8.123671
GSM1108	G2(n=271	8.792101
GSM1108	G2(n=271	7.405974
GSM1108	G2(n=271	8.430067
GSM1108	G2(n=271	7.692799
GSM1108	G2(n=271	8.98906
GSM1108	G2(n=271	7.461808
GSM1108	G2(n=271	8.975804
GSM1108	G2(n=271	7.980082
GSM1108	G2(n=271	7.99245
GSM1108	G2(n=271	7.43415
GSM1108	G2(n=271	8.09474
GSM1108	G2(n=271	8.557613
GSM1108	G2(n=271	9.652952
GSM1108	G2(n=271	7.595338
GSM1108	G2(n=271	8.374799
GSM1108	G2(n=271	7.459738
GSM1108	G2(n=271	8.227302
GSM1108	G2(n=271	8.022918
GSM1108	G2(n=271	8.402448
GSM1108	G2(n=271	8.877072
GSM1108	G2(n=271	7.67463
GSM1108	G2(n=271	8.629767
GSM1108	G2(n=271	7.923363
GSM1108	G2(n=271	7.898752
GSM1108	G2(n=271	7.611341
GSM1108	G2(n=271	8.979684
GSM1108	G2(n=271	7.666761
GSM1108	G2(n=271	8.2172
GSM1108	G2(n=271	8.113502
GSM1108	G2(n=271	7.354027
GSM1108	G2(n=271	8.272711
GSM1108	G2(n=271	6.652183
GSM1108	G2(n=271	8.203446
GSM1108	G2(n=271	8.180239
GSM1108	G2(n=271	7.709067
GSM1108	G2(n=271	8.029323
GSM1108	G2(n=271	9.207551
GSM1108	G2(n=271	8.364141
GSM1108	G2(n=271	7.509084
GSM1108	G2(n=271	8.236044
GSM1108	G2(n=271	8.07997
GSM1108	G2(n=271	6.832907
GSM1108	G2(n=271	7.746092
GSM1108	G2(n=271	7.19523
GSM1108	G2(n=271	7.656076
GSM1108	G2(n=271	6.775839
GSM1108	G2(n=271	8.496207
GSM1108	G2(n=271	8.053093
GSM1108	G2(n=271	8.10852
GSM1108	G2(n=271	7.964963
GSM1108	G2(n=271	7.696205
GSM1108	G2(n=271	8.411981
GSM1108	G2(n=271	9.009268
GSM1108	G2(n=271	7.922434
GSM1108	G2(n=271	10.04457
GSM1108	G2(n=271	8.91359

GSM11089	G2(n=271	8.204685
GSM11089	G2(n=271	7.80662
GSM11089	G2(n=271	6.568481
GSM11089	G2(n=271	7.658967
GSM11089	G2(n=271	7.303801
GSM11089	G2(n=271	8.044513
GSM11089	G2(n=271	6.828333
GSM11089	G2(n=271	7.705269
GSM11089	G2(n=271	8.108103
GSM11089	G2(n=271	7.923363
GSM11089	G2(n=271	8.72495
GSM11089	G2(n=271	7.246846
GSM11089	G2(n=271	6.274098
GSM11089	G2(n=271	7.866938
GSM11089	G2(n=271	8.103556
GSM11089	G2(n=271	6.67667
GSM11089	G2(n=271	8.612105
GSM11089	G2(n=271	7.332956
GSM11089	G2(n=271	7.492745
GSM11089	G2(n=271	6.563684
GSM11089	G2(n=271	6.567825
GSM11089	G2(n=271	8.147034
GSM11089	G2(n=271	7.302216
GSM11089	G2(n=271	7.855434
GSM11089	G2(n=271	7.108716
GSM11089	G2(n=271	9.190887
GSM11089	G2(n=271	7.185151
GSM11089	G2(n=271	8.56924
GSM11089	G2(n=271	7.574467
GSM11089	G2(n=271	7.420544
GSM11089	G2(n=271	8.540947
GSM11089	G2(n=271	9.165088
GSM11089	G2(n=271	7.522108
GSM11089	G2(n=271	8.910409
GSM11089	G2(n=271	7.969065
GSM11089	G2(n=271	9.52577
GSM11089	G2(n=271	8.038243
GSM11089	G2(n=271	8.476545
GSM11089	G2(n=271	8.30572
GSM11089	G2(n=271	9.15882
GSM11089	G2(n=271	7.954079
GSM11089	G2(n=271	7.572081
GSM11089	G2(n=271	8.004678
GSM11089	G2(n=271	9.629199
GSM11089	G2(n=271	10.1233
GSM11089	G2(n=271	7.820572
GSM11089	G2(n=271	8.447798
GSM11089	G2(n=271	7.959447
GSM11090	G2(n=271	7.818659
GSM11090	G2(n=271	7.846238
GSM11090	G2(n=271	8.692671
GSM11090	G2(n=271	7.744649
GSM11090	G2(n=271	7.137418
GSM11090	G2(n=271	7.48682
GSM11090	G2(n=271	7.366282
GSM11090	G2(n=271	8.019835
GSM11090	G2(n=271	8.086828
GSM11090	G2(n=271	8.61665
GSM11090	G2(n=271	7.666761

GSM1109(	G2(n=271	8.584806
GSM1109(	G2(n=271	9.061068
GSM1109(	G2(n=271	8.61082
GSM1109(	G2(n=271	7.936182
GSM1109(	G2(n=271	8.389188
GSM1109(	G2(n=271	7.556672
GSM1109(	G2(n=271	7.565891
GSM1109(	G2(n=271	7.838143

Supplementary Table 13

gene_id	gene_nam	chr	Start	End	M142_FPK	C_FPKM	log2FoldC	pvalue
ENSG0000(	GREM1	chr15	32718004	32745106	31.21622	3.606052	3.127309	0
ENSG0000(	DDIT4	chr10	72273924	72276036	177.2651	532.3498	-1.57316	1.1857575
ENSG0000(	VDAC1	chr5	1.34E+08	1.34E+08	68.7843	230.9892	-1.73434	#######
ENSG0000(	VGF	chr7	1.01E+08	1.01E+08	73.58473	20.25131	1.874109	#######
ENSG0000(	PLXNA4	chr7	1.32E+08	1.33E+08	14.07273	2.448352	2.534504	#######
ENSG0000(	TOP2A	chr17	40388525	40417896	250.2325	610.3061	-1.27298	#######
ENSG0000(	TXNIP	chr1	1.46E+08	1.46E+08	33.25635	110.1177	-1.71421	#######
ENSG0000(	TNC	chr9	1.15E+08	1.15E+08	30.12559	7.974353	1.929979	#######
ENSG0000(	MFAP4	chr17	19383442	19387190	12.20094	62.57584	-2.34429	#######
ENSG0000(	PLK2	chr5	58453982	58460139	49.29991	6.110933	3.023025	#######
ENSG0000(	GFRA3	chr5	1.38E+08	1.38E+08	2.581112	23.1902	-3.15228	#######
ENSG0000(	SERPINF1	chr17	1762029	1777565	83.51953	229.081	-1.44226	#######
ENSG0000(	SP1	chr12	53380176	53416446	41.28083	97.23236	-1.22269	#######
ENSG0000(	KIF5C	chr2	1.49E+08	1.49E+08	78.2703	32.92207	1.262569	#######
ENSG0000(	PLXNA2	chr1	2.08E+08	2.08E+08	20.37022	3.62105	2.503183	#######
ENSG0000(	SLC7A11	chr4	1.38E+08	1.38E+08	19.06061	49.54724	-1.36492	#######
ENSG0000(	SLC5A3	chr21	34073578	34106260	3.139824	8.783994	-1.47082	#######
ENSG0000(	NEFM	chr8	24913758	24919098	403.1681	183.4003	1.149584	#######
ENSG0000(	DEPDC1	chr1	68474152	68497221	14.96489	38.04384	-1.33279	#######
ENSG0000(	ADAM12	chr10	1.26E+08	1.26E+08	16.29031	0.972162	4.071847	#######
ENSG0000(	PELI2	chr14	56117814	56301524	22.11995	56.30829	-1.33488	#######
ENSG0000(	DPYSL3	chr5	1.47E+08	1.48E+08	206.0015	100.7353	1.045402	#######
ENSG0000(	PALMD	chr1	99646113	99694541	12.39081	1.968743	2.667397	#######
ENSG0000(	VSNL1	chr2	17539126	17657018	5.501338	30.40342	-2.45213	#######
ENSG0000(	ATP8A1	chr4	42408373	42657105	33.72651	77.01569	-1.17793	#######
ENSG0000(	HSPA5	chr9	1.25E+08	1.25E+08	203.4295	101.9539	1.009876	#######
ENSG0000(	FABP7	chr6	1.23E+08	1.23E+08	0.105811	11.39774	-6.6785	#######
ENSG0000(	SLC39A10	chr2	1.96E+08	1.96E+08	21.03069	50.6583	-1.25486	#######
ENSG0000(	SSPN	chr12	26121991	26299290	7.483882	31.22431	-2.04688	#######
ENSG0000(	TMTC2	chr12	82686880	83134870	13.27791	31.57098	-1.23623	#######
ENSG0000(	BAALC	chr8	1.03E+08	1.03E+08	31.37368	4.915909	2.687216	#######
ENSG0000(	SEMA6D	chr15	47184101	47774228	22.09428	49.2183	-1.14226	#######
ENSG0000(	ANGPT1	chr8	1.07E+08	1.07E+08	3.347801	17.46714	-2.36958	#######
ENSG0000(	ITM2A	chrX	79360384	79367667	54.07368	13.59634	2.003671	#######
ENSG0000(	CMBL	chr5	10275875	10307902	3.971626	25.88053	-2.68946	#######
ENSG0000(	EFNB2	chr13	1.06E+08	1.07E+08	11.17431	2.930736	1.943084	#######
ENSG0000(	IER3	chr6	30743199	30744548	49.0824	16.3037	1.602927	#######
ENSG0000(	INA	chr10	1.03E+08	1.03E+08	56.09472	27.04887	1.065637	#######
ENSG0000(	MANF	chr3	51385291	51389397	121.4858	54.02557	1.18258	#######
ENSG0000(	RALGAPA:	chr14	35538352	35809304	34.07292	71.76865	-1.06131	#######
ENSG0000(	SGK1	chr6	1.34E+08	1.34E+08	21.38864	3.376845	2.675799	#######
ENSG0000(	EML6	chr2	54723499	54972025	31.34137	11.19029	1.497912	#######
ENSG0000(	ZIC2	chr13	99981784	99986765	31.98112	94.69356	-1.55296	#######
ENSG0000(	TRIM67	chr1	2.31E+08	2.31E+08	20.26641	6.963171	1.553669	#######
ENSG0000(	TSPAN11	chr12	30926428	30996602	4.243977	17.42007	-2.02392	#######
ENSG0000(	ARL4C	chr2	2.34E+08	2.34E+08	13.30731	3.946702	1.766621	#######
ENSG0000(	APLNR	chr11	57233577	57237235	11.08894	1.547559	2.853308	#######
ENSG0000(	ROBO2	chr3	75906695	77649964	11.15351	24.92461	-1.14666	#######
ENSG0000(	ELAVL3	chr19	11451326	11481046	113.0695	53.27653	1.098474	2.01E-99
ENSG0000(	SOCS2	chr12	93569814	93583487	5.10086	23.42662	-2.1845	4.13E-98
ENSG0000(	MBTD1	chr17	51177425	51260163	17.40825	39.16768	-1.15638	4.44E-95
ENSG0000(	SPARCL1	chr4	87473335	87531061	27.16486	69.09237	-1.33309	1.38E-92
ENSG0000(	VEGFD	chrX	15345596	15384413	1.48097	14.92643	-3.31638	4.31E-92
ENSG0000(	LRP1	chr12	57128483	57213361	18.31357	39.5114	-1.09603	3.38E-91
ENSG0000(	MAP2	chr2	2.09E+08	2.1E+08	66.95571	33.66359	1.005478	4.97E-91
ENSG0000(	IMPDH1	chr7	1.28E+08	1.28E+08	79.43979	39.1064	1.03528	1.14E-89
ENSG0000(	SPTSSB	chr3	1.61E+08	1.61E+08	30.02794	7.398822	2.035519	1.23E-89

ENSG0000(	ADGRA2	chr8	37784191	37844896	7.164693	17.54044	-1.27864	6.84E-87
ENSG0000(	CASP3	chr4	1.85E+08	1.85E+08	74.62521	36.89127	1.029791	1.43E-86
ENSG0000(	CPAMD8	chr19	16892951	17026815	1.748431	12.90176	-2.86679	9.45E-86
ENSG0000(	VAT1L	chr16	77788564	77980107	31.53687	12.86461	1.306058	1.54E-85
ENSG0000(	SIX3	chr2	44941702	44946071	19.91929	40.63394	-1.01506	6.51E-85
ENSG0000(	SEMA5A	chr5	9035033	9546075	7.557077	17.97605	-1.237	1.18E-83
ENSG0000(	PHLDA1	chr12	76025447	76033932	8.861498	3.364207	1.410676	1.21E-83
ENSG0000(	NDUFA4L:	chr12	57234903	57240715	1.571567	18.16974	-3.50959	1.25E-83
ENSG0000(	B4GALNT:	chr12	57623409	57633239	47.56818	22.08076	1.120497	3.12E-83
ENSG0000(	SYT9	chr11	7238778	7469043	12.20281	3.287035	1.904523	1.48E-82
ENSG0000(	SERINC5	chr5	80111651	80256048	20.92562	42.45465	-1.00732	7.23E-82
ENSG0000(	TNXB	chr6	32041153	32115334	1.171706	5.335815	-2.17405	6.61E-80
ENSG0000(	AMHR2	chr12	53423855	53431672	1.946137	13.11161	-2.73465	1.16E-79
ENSG0000(	ZIC5	chr13	99962964	99971909	4.519558	11.58906	-1.34502	2.81E-79
ENSG0000(	KIT	chr4	54657918	54740715	5.929135	20.14424	-1.75006	5.49E-79
ENSG0000(	NEFH	chr22	29480218	29491390	27.87836	12.59763	1.158523	5.66E-79
ENSG0000(	MYO10	chr5	16661907	16936288	1.136226	6.692761	-2.54493	1.25E-76
ENSG0000(	MCTP2	chr15	94231538	94483952	2.837961	10.36555	-1.8545	2.28E-76
ENSG0000(	RASGRP1	chr15	38488103	38565575	4.309614	15.99557	-1.87734	3.93E-76
ENSG0000(	SDF2L1	chr22	21642302	21644299	51.17473	16.48203	1.646959	7.30E-76
ENSG0000(	ELN	chr7	74027789	74069907	15.36959	41.5208	-1.42083	1.38E-75
ENSG0000(	FMOD	chr1	2.03E+08	2.03E+08	0.948819	8.452014	-3.13508	1.56E-74
ENSG0000(	PLAT	chr8	42174718	42207676	25.64638	8.514858	1.604702	6.11E-74
ENSG0000(	ETV4	chr17	43527844	43579620	68.97962	33.77402	1.042901	6.24E-74
ENSG0000(	SPOCK1	chr5	1.37E+08	1.38E+08	66.40143	137.4996	-1.03665	4.87E-73
ENSG0000(	ST8SIA2	chr15	92393881	92468728	25.23544	10.22044	1.316698	7.66E-73
ENSG0000(	ADM	chr11	10305073	10307397	3.993167	17.91351	-2.15063	1.91E-72
ENSG0000(	JPT1	chr17	75135248	75168281	274.0615	136.3353	1.020752	6.65E-69
ENSG0000(	FAXDC2	chr5	1.55E+08	1.55E+08	6.337177	23.3357	-1.86716	3.82E-68
ENSG0000(	DEPDC1B	chr5	60596912	60700190	21.28564	47.01667	-1.12962	3.80E-67
ENSG0000(	NPY	chr7	24284188	24291862	23.66763	2.985696	2.994478	1.63E-66
ENSG0000(	GRIP1	chr12	66347431	67069162	3.80325	14.88433	-1.95338	1.03E-65
ENSG0000(	NGFR	chr17	49495293	49515008	19.07945	39.03012	-1.0189	3.64E-65
ENSG0000(	ADAMTS1	chr21	26835755	26845409	31.21566	15.04844	1.066201	5.74E-64
ENSG0000(	MMP15	chr16	58025754	58046901	9.722872	2.407133	2.023851	1.14E-63
ENSG0000(	TIMP3	chr22	32801705	32863041	1.311105	4.832016	-1.86756	9.78E-63
ENSG0000(	FAM89A	chr1	2.31E+08	2.31E+08	31.77824	12.78617	1.326559	1.05E-62
ENSG0000(	CYBA	chr16	88643289	88651054	25.72699	62.88781	-1.2756	1.44E-61
ENSG0000(	ADAMTS2	chr5	1.79E+08	1.79E+08	8.318208	20.61008	-1.29521	4.77E-61
ENSG0000(	ARRDC4	chr15	97960703	97973833	3.40354	9.411343	-1.45396	8.39E-61
ENSG0000(	GNG11	chr7	93921735	93928610	4.873759	0.945846	2.376371	3.60E-59
ENSG0000(	PALM2AK.	chr9	1.09E+08	1.1E+08	1.690331	7.0748	-2.05153	4.38E-59
ENSG0000(	COL23A1	chr5	1.78E+08	1.79E+08	16.04334	36.28646	-1.16427	1.34E-58
ENSG0000(	MEGF6	chr1	3487951	3611508	0.490587	3.857764	-2.95595	1.86E-58
ENSG0000(	AEN	chr15	88621337	88632281	26.47426	12.33321	1.11537	1.91E-58
ENSG0000(	TMEM151	chr6	44270450	44307506	10.49275	3.624387	1.545446	2.37E-58
ENSG0000(	DIAPH2	chrX	96684663	97604997	0.737109	3.511736	-2.23617	9.55E-58
ENSG0000(	CDC42EP4	chr17	73283624	73312005	30.71907	64.35682	-1.054	1.13E-57
ENSG0000(	ATP2B4	chr1	2.04E+08	2.04E+08	7.160257	14.95299	-1.04884	1.89E-57
ENSG0000(	PKP2	chr12	32790755	32896777	13.97451	31.42963	-1.15616	7.90E-57
ENSG0000(	ZNF33B	chr10	42574185	42638570	13.98044	29.99942	-1.08772	9.30E-57
ENSG0000(	NR4A2	chr2	1.56E+08	1.56E+08	21.43234	8.561102	1.336767	1.14E-56
ENSG0000(	CCNE2	chr8	94879770	94896678	38.2315	17.12314	1.171433	1.28E-56
ENSG0000(	BEND4	chr4	42110853	42152878	0.885104	5.767174	-2.69057	9.74E-56
ENSG0000(	COL6A6	chr3	1.31E+08	1.31E+08	5.10083	1.682948	1.612518	2.63E-55
ENSG0000(	ADAMTS9	chr3	64515654	64688000	19.09559	7.904254	1.28681	3.32E-55
ENSG0000(	NR3C1	chr5	1.43E+08	1.43E+08	10.83968	23.02607	-1.07394	1.02E-53
ENSG0000(	PTPRO	chr12	15322257	15602175	11.48767	4.732257	1.29189	2.42E-53

ENSG0000(	METTL14	chr4	1.19E+08	1.19E+08	6.065055	15.01187	-1.29391	8.77E-53
ENSG0000(	TCEAL7	chrX	1.03E+08	1.03E+08	36.10778	13.97288	1.383791	8.10E-52
ENSG0000(	RGS16	chr1	1.83E+08	1.83E+08	22.29821	10.85069	1.052012	1.86E-51
ENSG0000(	CCNE1	chr19	29811991	29824312	22.01264	8.2378	1.431153	4.53E-51
ENSG0000(	PDGFRA	chr4	54229280	54298245	2.042866	9.223063	-2.16083	4.72E-51
ENSG0000(	APOLD1	chr12	12725917	12829975	5.193998	13.50491	-1.36527	6.45E-51
ENSG0000(	TUBB2A	chr6	3153666	3157544	51.46982	25.86063	1.005846	1.58E-50
ENSG0000(	CGNL1	chr15	57375967	57550727	2.735405	8.543796	-1.62812	2.40E-50
ENSG0000(	SCUBE1	chr22	43197280	43343372	5.947393	17.53989	-1.54737	3.54E-50
ENSG0000(	NRP1	chr10	33177492	33336262	17.34128	7.55599	1.211913	3.63E-50
ENSG0000(	SLC18A2	chr10	1.17E+08	1.17E+08	0.802032	3.57307	-2.14029	4.86E-49
ENSG0000(	PDLIM3	chr4	1.86E+08	1.86E+08	13.9043	30.62679	-1.12534	7.58E-49
ENSG0000(	SEMA3D	chr7	84995553	85186855	10.0959	3.634496	1.487529	2.79E-48
ENSG0000(	SVIP	chr11	22813799	22830299	32.82461	15.54824	1.090905	3.29E-48
ENSG0000(	CEMIP2	chr9	71683366	71816690	7.142435	1.758169	2.031375	4.18E-48
ENSG0000(	ENDOD1	chr11	95089846	95132645	8.659968	3.634734	1.264814	5.79E-48
ENSG0000(	NPR3	chr5	32689070	32791724	2.335631	7.201239	-1.61095	9.22E-48
ENSG0000(	DYNC1I1	chr7	95772506	96110322	9.306116	1.777572	2.398654	1.55E-47
ENSG0000(	RASSF2	chr20	4780023	4823608	3.953528	1.278543	1.639519	1.60E-47
ENSG0000(	CRABP1	chr15	78340353	78348225	38.20241	13.07404	1.560213	2.06E-47
ENSG0000(	TRIQK	chr8	92883532	93017673	13.87636	34.21528	-1.28816	3.52E-47
ENSG0000(	FAT3	chr11	92352096	92896470	0.408086	1.956768	-2.24409	5.00E-47
ENSG0000(	TENT5A	chr6	81491439	81752774	3.33349	0.439839	2.929397	1.81E-46
ENSG0000(	PCDH7	chr4	30720415	31146805	9.720867	3.719256	1.397833	2.22E-46
ENSG0000(	ITGA6	chr2	1.72E+08	1.73E+08	4.74556	0.8868	2.428889	2.66E-46
ENSG0000(	NEDD9	chr6	11183298	11382348	25.78672	12.40757	1.068305	2.77E-46
ENSG0000(	DGKB	chr7	14145049	14974777	7.882585	17.19042	-1.111	4.19E-46
ENSG0000(	TSPAN13	chr7	16753755	16784536	11.61824	2.57592	2.185957	5.03E-46
ENSG0000(	PPARA	chr22	46150521	46243756	2.574361	8.399001	-1.69111	5.35E-46
ENSG0000(	RMDN1	chr8	86468257	86514357	26.54632	55.59312	-1.05296	1.07E-45
ENSG0000(	FJX1	chr11	35618460	35620865	21.79903	9.13803	1.266484	2.89E-45
ENSG0000(	DBNDD1	chr16	90004871	90020128	24.02666	11.8142	1.03679	2.19E-44
ENSG0000(	MARCHF4	chr2	2.16E+08	2.16E+08	7.025405	2.833189	1.321743	2.33E-44
ENSG0000(	JAZF1	chr7	27830573	28180795	15.89094	32.80019	-1.03179	2.53E-44
ENSG0000(	SCN11A	chr3	38845764	39052157	1.676241	4.997158	-1.56118	5.28E-44
ENSG0000(	B4GALNT:	chr12	459939	563509	3.083466	8.802122	-1.50016	3.28E-43
ENSG0000(	RERG	chr12	15107783	15348675	6.828736	19.17638	-1.47558	3.75E-43
ENSG0000(	ZC3H6	chr2	1.12E+08	1.12E+08	3.722567	7.629256	-1.02186	6.81E-43
ENSG0000(	LRRN1	chr3	3799431	3849834	3.842667	11.03251	-1.5087	1.02E-42
ENSG0000(	RORB	chr9	74497335	74693177	7.380061	3.38447	1.138947	4.25E-42
ENSG0000(	C6orf141	chr6	49550646	49561907	10.29556	2.095427	2.303723	4.39E-42
ENSG0000(	FHAD1	chr1	15247272	15400283	1.788627	8.379063	-2.21422	1.38E-41
ENSG0000(	SPRY2	chr13	80335976	80341126	15.46303	7.197347	1.11574	1.38E-41
ENSG0000(	AIF1L	chr9	1.31E+08	1.31E+08	5.965319	14.50632	-1.26875	1.69E-41
ENSG0000(	ANKH	chr5	14704800	14871778	6.744004	17.11009	-1.33013	6.00E-41
ENSG0000(	LRATD2	chr8	1.27E+08	1.27E+08	5.614686	2.084336	1.441597	8.53E-41
ENSG0000(	EHD4	chr15	41895933	41972557	6.873396	2.74828	1.334586	8.78E-41
ENSG0000(	CORO1A	chr16	30182827	30189076	30.11376	13.8101	1.137732	9.00E-41
ENSG0000(	HLA-E	chr6	30489509	30494194	2.943759	10.71444	-1.84991	1.72E-40
ENSG0000(	MYLK3	chr16	46702282	46790407	3.10389	9.324848	-1.57272	1.92E-40
ENSG0000(	YPEL2	chr17	59331655	59401729	5.325165	13.88754	-1.36961	2.28E-40
ENSG0000(	BTBD11	chr12	1.07E+08	1.08E+08	3.474622	0.669068	2.389666	4.14E-40
ENSG0000(	PKIB	chr6	1.22E+08	1.23E+08	1.578114	8.366388	-2.38816	8.02E-40
ENSG0000(	STMN4	chr8	27235323	27258420	11.5175	3.312224	1.807257	1.31E-39
ENSG0000(	TAGLN3	chr3	1.12E+08	1.12E+08	52.96286	25.76087	1.053293	2.79E-39
ENSG0000(	LOX	chr5	1.22E+08	1.22E+08	12.8704	5.359718	1.276972	1.69E-38
ENSG0000(	XYLT1	chr16	17101769	17470960	4.655898	1.349237	1.798345	2.19E-38
ENSG0000(	STK26	chrX	1.32E+08	1.32E+08	4.922745	11.44657	-1.20328	2.27E-38

ENSG0000(	SHISA2	chr13	26044597	26052016	1.318099	0.060882	4.414868	4.76E-38
ENSG0000(	CHAC2	chr2	53767804	53775196	18.00673	7.336879	1.30841	4.92E-38
ENSG0000(	ARHGEF6	chrX	1.37E+08	1.37E+08	7.082843	3.357481	1.090241	1.56E-37
ENSG0000(	CHST11	chr12	1.04E+08	1.05E+08	11.76256	5.035452	1.238129	5.83E-37
ENSG0000(	ZBTB41	chr1	1.97E+08	1.97E+08	2.492917	5.417248	-1.10596	1.60E-36
ENSG0000(	WNT6	chr2	2.19E+08	2.19E+08	5.452619	15.97835	-1.53714	3.54E-36
ENSG0000(	CRELD2	chr22	49918167	49927540	33.01966	15.93107	1.065425	4.29E-36
ENSG0000(	KAZALD1	chr10	1.01E+08	1.01E+08	11.74223	25.42448	-1.10115	1.55E-34
ENSG0000(	TNIK	chr3	1.71E+08	1.71E+08	3.122276	7.739907	-1.29671	1.78E-34
ENSG0000(	PKHD1	chr6	51615299	52087613	0.299846	1.040104	-1.78008	2.03E-34
ENSG0000(	IL33	chr9	6215786	6257983	2.21382	0.058599	5.169279	2.06E-34
ENSG0000(	KIRREL3	chr11	1.26E+08	1.27E+08	3.330572	0.384874	3.108648	2.48E-34
ENSG0000(	RELL2	chr5	1.42E+08	1.42E+08	13.02778	5.936044	1.146725	4.68E-34
ENSG0000(	REST	chr4	56907876	56966808	2.990022	6.709864	-1.15207	5.68E-34
ENSG0000(	NFKBIE	chr6	44258166	44265788	14.4603	6.102076	1.256131	6.02E-34
ENSG0000(	CDKN1C	chr11	2883213	2885775	2.540666	8.448799	-1.71838	1.25E-32
ENSG0000(	ELMO1	chr7	36854361	37449249	10.33936	22.35619	-1.09923	2.40E-32
ENSG0000(	RPS6KL1	chr14	74903951	74923302	9.534141	3.860927	1.316211	4.67E-32
ENSG0000(	SULT1C4	chr2	1.08E+08	1.08E+08	2.259359	8.293478	-1.86087	6.53E-32
ENSG0000(	CYP21A2	chr6	32038327	32041644	1.898542	8.456865	-2.13793	1.30E-31
ENSG0000(	NDRG1	chr8	1.33E+08	1.33E+08	4.606419	15.85502	-1.76889	2.18E-31
ENSG0000(	ACVRL1	chr12	51906908	51923361	1.200004	5.333825	-2.13412	2.29E-31
ENSG0000(	C1QL1	chr17	44959693	44968303	1.84358	8.101088	-2.12061	4.56E-31
ENSG0000(	XPNPEP2	chrX	1.3E+08	1.3E+08	0.563644	3.159075	-2.46693	1.06E-30
ENSG0000(	ASIC1	chr12	50057548	50083611	7.83001	2.994152	1.398307	1.13E-30
ENSG0000(	TPBGL	chr11	75240774	75243704	11.82263	5.822103	1.034244	1.26E-30
ENSG0000(	KCTD12	chr13	76880175	76886405	1.68282	0.461186	1.875363	1.67E-30
ENSG0000(	TENM1	chrX	1.24E+08	1.25E+08	1.792866	0.584493	1.632074	2.11E-30
ENSG0000(	PGP	chr16	2211593	2214840	28.93658	14.41094	1.018382	2.21E-30
ENSG0000(	SLC16A2	chrX	74421493	74533917	1.594564	4.537705	-1.49346	3.01E-30
ENSG0000(	SYT16	chr14	61811974	62112550	3.823401	1.15082	1.742316	6.85E-30
ENSG0000(	IGFBP4	chr17	40443450	40457725	6.637931	13.54953	-1.01566	7.15E-30
ENSG0000(	NKX3-1	chr8	23678697	23682938	2.463725	0.183	3.74738	7.31E-30
ENSG0000(	SMPDL3A	chr6	1.23E+08	1.23E+08	7.146133	19.08671	-1.40281	1.27E-29
ENSG0000(	CDC42EP1	chr22	37560480	37569405	2.015493	8.284575	-2.02292	3.50E-29
ENSG0000(	NEK6	chr9	1.24E+08	1.24E+08	1.352023	5.628316	-2.04341	3.64E-29
ENSG0000(	MEGF10	chr5	1.27E+08	1.27E+08	2.764302	0.481241	2.52281	4.11E-29
ENSG0000(	RELT	chr11	73376399	73397474	10.80017	5.257088	1.051573	8.18E-29
ENSG0000(	LRIG1	chr3	66378797	66501263	1.808702	6.295867	-1.78449	1.55E-28
ENSG0000(	TEX36	chr10	1.26E+08	1.26E+08	2.37414	10.58213	-2.13844	1.58E-28
ENSG0000(	HINT3	chr6	1.26E+08	1.26E+08	4.924089	10.39976	-1.06477	1.98E-28
ENSG0000(	COL14A1	chr8	1.2E+08	1.2E+08	2.346462	0.557816	2.07873	2.62E-28
ENSG0000(	SCRT1	chr8	1.44E+08	1.44E+08	4.765459	2.004973	1.259914	3.44E-28
ENSG0000(	PTPRG	chr3	61561569	62297609	3.645256	7.888144	-1.10069	4.67E-28
ENSG0000(	HCN1	chr5	45254948	45696498	5.586153	2.551033	1.142002	1.02E-27
ENSG0000(	TMEM19	chr12	71686082	71705047	5.073482	12.05894	-1.23431	1.66E-27
ENSG0000(	ABAT	chr16	8674596	8784575	6.390459	14.15318	-1.13384	1.92E-27
ENSG0000(	CACNG4	chr17	66964707	67033398	2.339817	5.350146	-1.17987	3.01E-27
ENSG0000(	AHNAK2	chr14	1.05E+08	1.05E+08	0.438132	1.274268	-1.5244	3.40E-27
ENSG0000(	PLEKHG5	chr1	6467122	6520074	1.964723	5.787475	-1.54378	3.41E-27
ENSG0000(	PLA2G6	chr22	38111495	38214778	7.336374	15.22886	-1.03967	5.06E-27
ENSG0000(	PLCG2	chr16	81739097	81962685	0.155345	2.625726	-4.02564	7.80E-27
ENSG0000(	FRMD3	chr9	83242990	83538546	8.535155	3.510733	1.29484	7.80E-27
ENSG0000(	MYRF	chr11	61752636	61788518	1.363481	4.067962	-1.56319	9.74E-27
ENSG0000(	KCNK10	chr14	88180103	88326907	0.913209	0.092218	3.298189	2.02E-26
ENSG0000(	FAM117A	chr17	49710332	49789180	8.137892	19.04972	-1.21279	3.10E-26
ENSG0000(	CHST15	chr10	1.24E+08	1.24E+08	5.176987	1.720682	1.598168	3.91E-26
ENSG0000(	PKNOX2	chr11	1.25E+08	1.25E+08	0.628609	4.164513	-2.70525	5.10E-26

ENSG0000(	NTRK1	chr1	1.57E+08	1.57E+08	11.29281	5.11064	1.156834	5.53E-26
ENSG0000(	MME	chr3	1.55E+08	1.55E+08	4.411271	1.221718	1.85929	6.52E-26
ENSG0000(	SLCO4A1	chr20	62642503	62685785	12.10001	5.159723	1.242227	7.61E-26
ENSG0000(	CXADR	chr21	17513043	17593579	4.998128	11.17895	-1.14663	2.08E-25
ENSG0000(	MRPL41	chr9	1.38E+08	1.38E+08	168.2159	75.86929	1.162347	2.53E-25
ENSG0000(	KDM4C	chr9	6720863	7175648	6.274198	12.72047	-1.00573	3.05E-25
ENSG0000(	DBX1	chr11	20156155	20160475	2.895646	0.250073	3.515974	3.42E-25
ENSG0000(	RTN2	chr19	45485294	45497055	16.54967	7.426347	1.170424	3.58E-25
ENSG0000(	ABHD17C	chr15	80679684	80755621	14.0803	6.769926	1.068056	4.00E-25
ENSG0000(	EPHX2	chr8	27490781	27545564	7.930766	16.29655	-1.02518	6.14E-25
ENSG0000(	RREB1	chr6	7107597	7251980	0.835879	2.43366	-1.52639	1.77E-24
ENSG0000(	AHR	chr7	16916359	17346152	1.35874	0.139469	3.287779	2.01E-24
ENSG0000(	NEO1	chr15	73051710	73305205	4.716933	9.615864	-1.01458	2.13E-24
ENSG0000(	MFSD2A	chr1	39955112	39969968	8.753182	2.871358	1.620763	2.66E-24
ENSG0000(	GPM6B	chrX	13770939	13938638	7.207488	14.94265	-1.0375	3.25E-24
ENSG0000(	COQ10B	chr2	1.97E+08	1.97E+08	15.54518	6.42942	1.283961	3.28E-24
ENSG0000(	ABHD4	chr14	22598237	22613215	4.026421	12.47071	-1.61705	5.48E-24
ENSG0000(	ASNS	chr7	97851677	97872542	7.336965	15.43212	-1.05842	5.79E-24
ENSG0000(	TSPOAP1	chr17	58301228	58328795	0.746031	2.820612	-1.90034	1.07E-23
ENSG0000(	CDO1	chr5	1.16E+08	1.16E+08	7.403262	1.553215	2.262735	1.21E-23
ENSG0000(	ZBTB38	chr3	1.41E+08	1.41E+08	2.978935	7.312406	-1.28075	1.25E-23
ENSG0000(	PUDP	chrX	6667865	7148158	20.64302	10.4124	1.000306	1.82E-23
ENSG0000(	PCSK9	chr1	55039447	55064852	0.147284	1.617038	-3.41622	1.82E-23
ENSG0000(	FGFR2	chr10	1.21E+08	1.22E+08	2.22745	5.695705	-1.33997	2.47E-23
ENSG0000(	DUSP8	chr11	1554051	1572271	3.14984	0.820133	1.946895	4.37E-23
ENSG0000(	RELL1	chr4	37590800	37686376	8.895742	4.0192	1.158749	5.14E-23
ENSG0000(	AJAP1	chr1	4654609	4792534	0.965733	0.17374	2.479129	6.70E-23
ENSG0000(	IRAG1	chr11	10573091	10693988	1.12298	3.689116	-1.70137	7.11E-23
ENSG0000(	CACNB2	chr10	18140424	18543557	5.299791	2.170183	1.299251	7.97E-23
ENSG0000(	ALPK1	chr4	1.12E+08	1.12E+08	0.461971	2.476074	-2.39987	9.92E-23
ENSG0000(	TGFBR2	chr3	30606601	30694142	1.588857	3.986814	-1.31265	1.08E-22
ENSG0000(	FBLN2	chr3	13549125	13638422	0.264373	1.922465	-2.83399	1.37E-22
ENSG0000(	KALRN	chr3	1.24E+08	1.25E+08	5.393418	2.46977	1.140087	1.46E-22
ENSG0000(	GPR27	chr3	71753855	71756496	4.215852	1.53453	1.471765	1.74E-22
ENSG0000(	CERS4	chr19	8206736	8262421	1.393994	5.782574	-2.03562	3.18E-22
ENSG0000(	PDE4B	chr1	65792514	66374579	3.826314	0.89577	2.104932	3.39E-22
ENSG0000(	STK17A	chr7	43582758	43650713	13.40441	6.532881	1.049119	3.83E-22
ENSG0000(	MOK	chr14	1.02E+08	1.02E+08	5.149959	12.72345	-1.2913	4.46E-22
ENSG0000(	ERBB2	chr17	39687914	39730426	1.631312	5.637294	-1.77445	4.64E-22
ENSG0000(	GALNT7	chr4	1.73E+08	1.73E+08	0.773427	3.237838	-2.04876	7.06E-22
ENSG0000(	DSCAML1	chr11	1.17E+08	1.18E+08	0.600784	1.848585	-1.60587	1.77E-21
ENSG0000(	KLHL4	chrX	87517409	87670050	1.797662	0.508743	1.828979	1.98E-21
ENSG0000(	DHFR2	chr3	94047836	94063389	3.31928	7.444865	-1.15081	2.05E-21
ENSG0000(	LTBP1	chr2	32946953	33399509	4.094923	1.487162	1.472586	2.38E-21
ENSG0000(	CSF1	chr1	1.1E+08	1.1E+08	6.314531	13.6592	-1.09965	2.42E-21
ENSG0000(	MGST1	chr12	16347142	16609259	3.736579	11.66656	-1.62622	2.55E-21
ENSG0000(	ELF4	chrX	1.3E+08	1.3E+08	0.835534	2.651409	-1.6507	2.77E-21
ENSG0000(	ABTB1	chr3	1.28E+08	1.28E+08	2.166804	6.399255	-1.54644	3.26E-21
ENSG0000(	RAB3IP	chr12	69738860	69823204	2.72578	0.630053	2.124112	3.78E-21
ENSG0000(	P2RX7	chr12	1.21E+08	1.21E+08	2.469763	6.21267	-1.31596	3.79E-21
ENSG0000(	MGAT4A	chr2	98619106	98731132	0.448859	2.093941	-2.20315	1.03E-20
ENSG0000(	WT1	chr11	32387775	32435564	2.027733	0.293249	2.781002	1.07E-20
ENSG0000(	VIPR2	chr7	1.59E+08	1.59E+08	0.851175	3.354685	-1.96305	1.09E-20
ENSG0000(	FAM184B	chr4	17629306	17781621	0.31625	1.064301	-1.73518	1.19E-20
ENSG0000(	KCNK3	chr2	26692690	26733420	1.977146	4.145068	-1.05476	1.46E-20
ENSG0000(	TTYH2	chr17	74213571	74262020	1.904059	5.743505	-1.57732	1.59E-20
ENSG0000(	OPHN1	chrX	68042344	68433913	0.238034	1.928271	-2.98552	2.52E-20
ENSG0000(	ADAM23	chr2	2.06E+08	2.07E+08	5.902935	2.746863	1.115006	2.58E-20

ENSG0000(	CARF	chr2	2.03E+08	2.03E+08	5.630486	11.41477	-1.00517	3.08E-20
ENSG0000(	VSTM4	chr10	49014236	49115522	0.730298	2.60544	-1.81629	3.09E-20
ENSG0000(	LIX1	chr5	97091867	97142753	0.2953	1.848409	-2.6231	3.32E-20
ENSG0000(	LRP1B	chr2	1.4E+08	1.42E+08	0.73962	2.273697	-1.60676	3.35E-20
ENSG0000(	YPEL3	chr16	30092314	30096915	4.966374	12.62252	-1.33262	4.89E-20
ENSG0000(	LEPR	chr1	65420652	65641559	1.15331	3.010071	-1.37028	7.16E-20
ENSG0000(	SMARCA2	chr9	1980290	2193624	10.1672	5.051019	1.022375	7.93E-20
ENSG0000(	PPARG	chr3	12287368	12434356	4.307729	1.159923	1.904492	8.26E-20
ENSG0000(	GAS7	chr17	9910609	10198551	0.625587	2.683007	-2.08159	1.07E-19
ENSG0000(	WWC3	chrX	10015254	10144474	0.664171	2.138226	-1.67171	1.09E-19
ENSG0000(	FXYD2	chr11	1.18E+08	1.18E+08	0.273106	2.877454	-3.35296	1.26E-19
ENSG0000(	RIMKLA	chr1	42380792	42424232	2.995334	1.425425	1.085056	1.33E-19
ENSG0000(	PHLDA2	chr11	2928273	2929420	19.14433	7.685881	1.330269	1.39E-19
ENSG0000(	H2AC11	chr6	27133042	27135291	2.026415	4.937194	-1.27049	1.41E-19
ENSG0000(	PIGV	chr1	26787054	26800659	2.265213	4.72968	-1.04813	1.59E-19
ENSG0000(	NIPAL3	chr1	24415802	24472976	2.149295	5.094748	-1.2297	1.80E-19
ENSG0000(	PRSS23	chr11	86791059	86952910	4.666167	1.465123	1.678544	2.08E-19
ENSG0000(	HRH3	chr20	62214960	62220278	2.773199	0.545982	2.355176	2.32E-19
ENSG0000(	LYSMD2	chr15	51723011	51751585	10.65553	4.333457	1.308865	3.03E-19
ENSG0000(	CCDC181	chr1	1.69E+08	1.69E+08	8.625252	3.929573	1.145854	3.06E-19
ENSG0000(	EHHADH	chr3	1.85E+08	1.85E+08	4.670718	10.75931	-1.189	3.06E-19
ENSG0000(	CCN1	chr1	85580761	85584589	8.51113	3.931385	1.125329	3.10E-19
ENSG0000(	KCNB1	chr20	49293394	49484297	2.968698	6.688322	-1.15814	6.14E-19
ENSG0000(	FRMD4A	chr10	13643706	14462142	6.421966	16.03576	-1.30541	6.23E-19
ENSG0000(	C7orf31	chr7	25134692	25180356	1.426401	4.289439	-1.5714	7.22E-19
ENSG0000(	LMO2	chr11	33858576	33892076	3.268033	0.637414	2.367535	8.30E-19
ENSG0000(	BCL11B	chr14	99169287	99272197	0.451103	1.593921	-1.80764	8.59E-19
ENSG0000(	ABCB1	chr7	87503017	87713323	1.86026	0.233252	2.990379	8.84E-19
ENSG0000(	SLC6A11	chr3	10816201	10940714	0.349872	1.83709	-2.3747	1.00E-18
ENSG0000(	TAFA4	chr3	68731766	68953297	11.99476	5.188453	1.21928	1.08E-18
ENSG0000(	FUT10	chr8	33370824	33473146	5.213313	11.02014	-1.06679	1.10E-18
ENSG0000(	APOE	chr19	44905791	44909393	2.608254	8.680707	-1.71633	1.32E-18
ENSG0000(	ATP11A	chr13	1.13E+08	1.13E+08	3.040305	6.260348	-1.02882	1.78E-18
ENSG0000(	CYP2S1	chr19	41193210	41207539	3.029981	8.118859	-1.40732	1.93E-18
ENSG0000(	PBLD	chr10	68282660	68333049	3.493006	7.835658	-1.15245	2.79E-18
ENSG0000(	PAIP2B	chr2	71182738	71227103	1.001281	2.16217	-1.0972	4.83E-18
ENSG0000(	PXDNL	chr8	51319577	51809445	0.384533	1.806746	-2.21377	5.12E-18
ENSG0000(	ITPKB	chr1	2.27E+08	2.27E+08	0.356429	1.203071	-1.73873	7.90E-18
ENSG0000(	CD163L1	chr12	7346685	7479897	14.43813	7.129603	1.030193	9.13E-18
ENSG0000(	ZNF572	chr8	1.25E+08	1.25E+08	0.949075	2.88652	-1.58741	1.01E-17
ENSG0000(	PTPN14	chr1	2.14E+08	2.15E+08	1.293663	2.767153	-1.08265	1.07E-17
ENSG0000(	CACNA2D	chr3	54122547	55074557	4.185066	1.758002	1.263155	1.70E-17
ENSG0000(	HECA	chr6	1.39E+08	1.39E+08	1.43569	3.200275	-1.14189	2.26E-17
ENSG0000(	FOXN4	chr12	1.09E+08	1.09E+08	0.187975	1.376361	-2.846	2.45E-17
ENSG0000(	FERMT1	chr20	6074845	6123030	1.223925	3.081814	-1.3178	2.48E-17
ENSG0000(	XAF1	chr17	6755447	6775647	0.102264	2.176934	-4.31223	2.56E-17
ENSG0000(	SASH1	chr6	1.48E+08	1.49E+08	4.021866	8.535442	-1.07171	2.79E-17
ENSG0000(	SYTL4	chrX	1.01E+08	1.01E+08	0.203616	1.313373	-2.6568	3.70E-17
ENSG0000(	B3GALT5	chr21	39556442	39673137	0.150784	0.863788	-2.49093	3.86E-17
ENSG0000(	FAM78B	chr1	1.66E+08	1.66E+08	1.503856	0.327691	2.201361	5.36E-17
ENSG0000(	DAND5	chr19	12965159	12974762	0.562515	2.475553	-2.11937	6.01E-17
ENSG0000(	UPP1	chr7	48088628	48108736	7.933964	3.014813	1.410185	8.98E-17
ENSG0000(	PEG3	chr19	56810077	56840728	0.832966	1.930748	-1.19804	9.33E-17
ENSG0000(	PROKR1	chr2	68643579	68658251	0.07114	0.692238	-3.24489	1.63E-16
ENSG0000(	TMCC3	chr12	94567122	94650557	3.429861	7.234909	-1.06236	1.65E-16
ENSG0000(	NUDT10	chrX	51332231	51337525	6.064456	2.855498	1.098838	1.79E-16
ENSG0000(	PCGF5	chr10	91163012	91284337	2.666818	1.176014	1.191347	2.98E-16
ENSG0000(	ERV3-1	chr7	64990356	65006743	3.502272	7.823885	-1.14437	3.50E-16

ENSG0000(	MINAR1	chr15	79432336	79472304	0.492757	1.352053	-1.44225	3.65E-16
ENSG0000(	ANKRD33	chr5	10564070	10657816	0.390061	1.237582	-1.65201	4.57E-16
ENSG0000(	CCNA1	chr13	36431520	36442870	0.029401	1.01901	-4.94428	4.71E-16
ENSG0000(	INSYN1	chr15	73735431	73752747	1.786285	4.315867	-1.25658	8.49E-16
ENSG0000(	EIF4E3	chr3	71675414	71754773	4.946404	2.467829	1.016331	8.87E-16
ENSG0000(	EDNRA	chr4	1.47E+08	1.48E+08	4.323004	1.88415	1.211114	8.90E-16
ENSG0000(	PWP2	chr21	44107373	44131181	6.994786	3.383802	1.059154	1.01E-15
ENSG0000(	EPHB3	chr3	1.85E+08	1.85E+08	2.571432	6.097964	-1.23277	1.01E-15
ENSG0000(	FN3K	chr17	82735615	82751196	6.074794	2.221446	1.462008	1.06E-15
ENSG0000(	WIPI1	chr17	68420948	68457513	7.375922	2.811606	1.400552	1.18E-15
ENSG0000(	COPRS	chr17	31851871	31859291	23.7334	11.66201	1.03847	1.26E-15
ENSG0000(	FXYD5	chr19	35154730	35169881	0.236545	2.605184	-3.40829	1.29E-15
ENSG0000(	COL22A1	chr8	1.39E+08	1.39E+08	2.288196	4.828765	-1.06364	1.40E-15
ENSG0000(	GAREM1	chr18	32124877	32470882	0.724433	0.113828	2.66728	1.40E-15
ENSG0000(	ASIC4	chr2	2.2E+08	2.2E+08	1.537616	3.720681	-1.26116	1.87E-15
ENSG0000(	GREB1L	chr18	21242242	21525417	2.333296	5.293058	-1.16895	1.99E-15
ENSG0000(	MLKL	chr16	74671855	74700960	2.201788	5.253821	-1.23913	2.19E-15
ENSG0000(	GRAMD1E	chr11	1.23E+08	1.24E+08	0.993406	2.59629	-1.37031	2.45E-15
ENSG0000(	DCLK3	chr3	36712422	36764349	0.191769	0.942192	-2.27071	3.80E-15
ENSG0000(	ZNF727	chr7	64045434	64085339	1.195117	0.539228	1.161159	3.88E-15
ENSG0000(	BMF	chr15	40087890	40108892	4.532534	9.746444	-1.09095	3.89E-15
ENSG0000(	TP73	chr1	3652516	3736201	0.281757	1.308409	-2.1936	4.04E-15
ENSG0000(	PCDH15	chr10	53802771	55627942	0.118233	0.620916	-2.3663	4.59E-15
ENSG0000(	ZP3	chr7	76397518	76442071	4.46683	10.42665	-1.20854	4.80E-15
ENSG0000(	MYO15B	chr17	75588058	75626849	0.765627	2.737182	-1.81954	5.99E-15
ENSG0000(	MOB3B	chr9	27325209	27529814	3.403361	1.62741	1.075044	6.24E-15
ENSG0000(	P4HA2	chr5	1.32E+08	1.32E+08	6.317195	2.313788	1.463967	8.73E-15
ENSG0000(	SNAI1	chr20	49982980	49988886	2.573053	6.449064	-1.31262	1.04E-14
ENSG0000(	IL15	chr4	1.42E+08	1.42E+08	1.958917	0.501541	1.971048	1.11E-14
ENSG0000(	DUSP10	chr1	2.22E+08	2.22E+08	7.492818	3.587223	1.076845	1.38E-14
ENSG0000(	LRRC4	chr7	1.28E+08	1.28E+08	8.051413	3.822944	1.088094	1.53E-14
ENSG0000(	GADD45G	chr9	89605012	89606555	4.715341	10.37849	-1.12342	1.76E-14
ENSG0000(	DBP	chr19	48630030	48637379	1.74067	4.08728	-1.21569	1.77E-14
ENSG0000(	PARD3B	chr2	2.05E+08	2.06E+08	0.906714	2.208632	-1.26906	1.92E-14
ENSG0000(	ITGA2	chr5	52989340	53094779	1.425068	2.918169	-1.02087	2.09E-14
ENSG0000(	TNFRSF12	chr16	3018445	3022383	6.026501	1.921647	1.660499	2.57E-14
ENSG0000(	RNF217	chr6	1.25E+08	1.25E+08	0.571058	1.835041	-1.67002	4.04E-14
ENSG0000(	IGFBP3	chr7	45912245	45921874	7.509344	3.483877	1.120373	4.37E-14
ENSG0000(	ENHO	chr9	34521043	34522990	8.527024	3.367924	1.354716	4.82E-14
ENSG0000(	TAFA3	chr1	1.13E+08	1.13E+08	0.80107	3.221095	-1.98555	5.16E-14
ENSG0000(	CA9	chr9	35673918	35681159	0.563369	2.994597	-2.38206	5.89E-14
ENSG0000(	PGAM2	chr7	44062727	44065567	1.484485	5.37046	-1.837	5.96E-14
ENSG0000(	TENT5C	chr1	1.18E+08	1.18E+08	0.326741	0.02771	3.528799	6.55E-14
ENSG0000(	KLRG2	chr7	1.39E+08	1.39E+08	0.855735	3.111116	-1.84093	6.92E-14
ENSG0000(	SLC2A10	chr20	46709649	46736347	0.571683	2.198891	-1.92237	7.26E-14
ENSG0000(	GRIK3	chr1	36795527	37034515	3.376268	1.683535	1.015953	8.52E-14
ENSG0000(	ERMAP	chr1	42817122	42844991	2.722748	5.752264	-1.06494	1.02E-13
ENSG0000(	ANO4	chr12	1.01E+08	1.01E+08	2.881801	1.041224	1.477709	1.03E-13
ENSG0000(	SLIT1	chr10	96998038	97185959	0.38353	1.518508	-1.96826	1.11E-13
ENSG0000(	LDLRAD4	chr18	13217498	13652755	0.626114	1.947712	-1.61877	1.23E-13
ENSG0000(	FAM241A	chr4	1.12E+08	1.12E+08	0.240077	0.017507	3.730155	1.27E-13
ENSG0000(	LCA5	chr6	79484991	79537458	1.322225	3.08222	-1.20543	1.29E-13
ENSG0000(	ZBED8	chr5	1.6E+08	1.6E+08	1.281246	3.101579	-1.25896	1.33E-13
ENSG0000(	NUPR1	chr16	28532708	28539008	1.333725	3.831934	-1.50602	1.58E-13
ENSG0000(	AGBL1	chr15	86079973	87029052	0.272996	1.044634	-1.91433	1.64E-13
ENSG0000(	CREBRF	chr5	1.73E+08	1.73E+08	1.570511	3.487254	-1.13566	1.67E-13
ENSG0000(	MT1F	chr16	56657731	56660698	8.674621	3.126046	1.487046	1.94E-13
ENSG0000(	SMTNL2	chr17	4583999	4608319	0.417202	2.243339	-2.39953	2.05E-13

ENSG0000(	NFATC1	chr18	79395856	79529325	0.681202	2.092463	-1.60272	2.26E-13
ENSG0000(	FUT9	chr6	96015974	96215612	0.232388	0.801759	-1.76831	2.39E-13
ENSG0000(	METTL24	chr6	1.1E+08	1.1E+08	1.551661	5.012561	-1.67544	2.56E-13
ENSG0000(	VRK2	chr2	57907629	58159920	0.738953	2.51605	-1.75031	3.07E-13
ENSG0000(	SEMA3E	chr7	83363238	83649139	0.998061	2.449021	-1.27906	3.18E-13
ENSG0000(	GRIP2	chr3	14489107	14556075	0.345847	1.061609	-1.60258	3.35E-13
ENSG0000(	CDK5R2	chr2	2.19E+08	2.19E+08	5.082844	2.432889	1.073478	3.78E-13
ENSG0000(	CEP112	chr17	65635537	66192133	0.992304	3.700983	-1.87936	3.80E-13
ENSG0000(	SLC27A2	chr15	50182196	50236385	1.386557	0.225096	2.621022	4.05E-13
ENSG0000(	RHBG	chr1	1.56E+08	1.56E+08	0.278548	1.669213	-2.54721	4.12E-13
ENSG0000(	PRLR	chr5	35048756	35230487	0.136847	1.015541	-2.85322	6.85E-13
ENSG0000(	ARG2	chr14	67619920	67651708	14.21604	7.140498	1.008237	8.40E-13
ENSG0000(	SMOC2	chr6	1.68E+08	1.69E+08	0.063481	1.009931	-3.89326	9.01E-13
ENSG0000(	TMEM229	chr7	1.24E+08	1.24E+08	3.233217	1.237127	1.400475	1.01E-12
ENSG0000(	SALL4	chr20	51782331	51802521	2.789287	1.004594	1.48107	1.05E-12
ENSG0000(	PYROXD2	chr10	98383565	98415182	3.185601	6.983444	-1.11693	1.23E-12
ENSG0000(	COLGALT:	chr1	1.84E+08	1.84E+08	1.1995	0.298979	2.001013	1.31E-12
ENSG0000(	SDK2	chr17	73334384	73644445	2.12441	1.069404	1.003203	1.66E-12
ENSG0000(	HSPA2	chr14	64535905	64546173	4.353493	2.135301	1.0383	1.68E-12
ENSG0000(	VEGFC	chr4	1.77E+08	1.77E+08	6.442398	3.234145	1.00714	2.79E-12
ENSG0000(	AK5	chr1	77282019	77559966	9.883872	4.8587	1.037969	3.05E-12
ENSG0000(	EPB41L2	chr6	1.31E+08	1.31E+08	2.224152	4.724563	-1.07367	3.14E-12
ENSG0000(	YBEY	chr21	46286342	46297751	7.438182	15.18424	-1.01409	3.75E-12
ENSG0000(	TMBIM1	chr2	2.18E+08	2.18E+08	3.64836	8.097937	-1.13649	4.89E-12
ENSG0000(	ADGRG6	chr6	1.42E+08	1.42E+08	1.45031	0.421621	1.79366	5.67E-12
ENSG0000(	RAP1GAP	chr1	21596215	21669363	1.653878	3.523608	-1.0769	6.03E-12
ENSG0000(	INHBA	chr7	41667168	41705834	0.091314	0.761491	-3.00819	7.08E-12
ENSG0000(	TNFRSF25	chr1	6460786	6466175	0.649277	3.663644	-2.47466	7.76E-12
ENSG0000(	ARHGEF2(	chr3	1.54E+08	1.54E+08	1.830507	0.838044	1.137712	9.44E-12
ENSG0000(	GUCY1B1	chr4	1.56E+08	1.56E+08	4.279143	2.121042	1.026505	1.06E-11
ENSG0000(	GAS1	chr9	86944362	86947506	1.377329	0.470338	1.55893	1.08E-11
ENSG0000(	CHODL	chr21	17901263	18267373	1.738829	0.544971	1.680472	1.12E-11
ENSG0000(	TIGAR	chr12	4307763	4360028	1.316741	0.406571	1.706031	1.49E-11
ENSG0000(	ANGPTL4	chr19	8363289	8374370	2.30507	0.496906	2.215832	1.59E-11
ENSG0000(	LRRN4	chr20	6040546	6054060	0.508023	0.026691	4.165285	1.82E-11
ENSG0000(	PSEN2	chr1	2.27E+08	2.27E+08	5.87212	2.770271	1.095398	1.87E-11
ENSG0000(	ARFGEF3	chr6	1.38E+08	1.38E+08	0.463715	0.202161	1.206543	2.17E-11
ENSG0000(	SHROOM4	chrX	50591647	50814302	0.283388	0.024776	3.450753	2.23E-11
ENSG0000(	KCNK2	chr1	2.15E+08	2.15E+08	3.341999	7.246143	-1.10197	2.55E-11
ENSG0000(	SENP8	chr15	72114258	72143692	2.630376	5.521204	-1.05601	2.58E-11
ENSG0000(	WDR86	chr7	1.51E+08	1.51E+08	0.611226	2.293792	-1.88608	3.23E-11
ENSG0000(	CAVIN1	chr17	42402449	42423256	0.907677	1.984469	-1.11336	3.31E-11
ENSG0000(	DACH1	chr13	71437966	71867204	0.068796	0.446958	-2.65979	3.51E-11
ENSG0000(	CCDC184	chr12	48183644	48185926	1.730587	0.570143	1.61421	4.22E-11
ENSG0000(	PAG1	chr8	80967810	81112068	3.038958	1.530545	1.00394	4.50E-11
ENSG0000(	TGFBR3L	chr19	7916145	7919097	3.275838	0.79072	2.051184	4.53E-11
ENSG0000(	CBLN1	chr16	49277917	49281838	7.862527	3.484016	1.183762	4.99E-11
ENSG0000(	KCNJ12	chr17	21376357	21419870	0.611166	1.496028	-1.27608	5.78E-11
ENSG0000(	PPP1R3C	chr10	91628442	91633071	1.247131	2.945501	-1.22503	6.67E-11
ENSG0000(	AMOTL2	chr3	1.34E+08	1.34E+08	2.519465	0.90642	1.483355	7.84E-11
ENSG0000(	HS3ST5	chr6	1.14E+08	1.14E+08	3.763384	1.652443	1.195693	7.99E-11
ENSG0000(	RHOJ	chr14	63204114	63293508	1.350435	3.48015	-1.35052	1.03E-10
ENSG0000(	PCDH17	chr13	57631744	57729311	2.879348	1.388557	1.064848	1.08E-10
ENSG0000(	CCSER1	chr4	90127535	91601913	0.466073	1.815327	-1.93658	1.11E-10
ENSG0000(	SORBS2	chr4	1.86E+08	1.86E+08	4.789852	9.894767	-1.03413	1.25E-10
ENSG0000(	ARMCX2	chrX	1.02E+08	1.02E+08	3.390694	6.990472	-1.03013	1.37E-10
ENSG0000(	S100A4	chr1	1.54E+08	1.54E+08	5.072953	11.76527	-1.20007	1.58E-10
ENSG0000(	ITGA11	chr15	68296532	68432163	0.906971	0.263936	1.789671	1.63E-10

ENSG0000(	KCTD19	chr16	67289428	67326760	0.155921	0.973011	-2.6022	1.65E-10
ENSG0000(	ASCL1	chr12	1.03E+08	1.03E+08	0.969615	2.389685	-1.2841	1.94E-10
ENSG0000(	SLC13A4	chr7	1.36E+08	1.36E+08	0.050656	0.656672	-3.61288	2.32E-10
ENSG0000(	CYRIA	chr2	16549459	16666331	0.179249	1.191106	-2.68517	2.36E-10
ENSG0000(	PCDH8	chr13	52842889	52848641	0.891806	0.243741	1.870332	2.48E-10
ENSG0000(	FNDC4	chr2	27491883	27495200	2.83849	5.759145	-1.00629	2.51E-10
ENSG0000(	HIF3A	chr19	46297046	46343433	0.511859	2.081976	-2.00024	2.70E-10
ENSG0000(	ATP8B3	chr19	1782075	1812276	0.358812	1.200114	-1.71953	2.95E-10
ENSG0000(	ADAMTS1	chr19	8580240	8610735	0.331026	1.449413	-2.10239	3.16E-10
ENSG0000(	OTUB2	chr14	94026340	94048930	1.325798	2.958909	-1.14149	3.25E-10
ENSG0000(	C14orf39	chr14	60396469	60515543	4.243813	1.727319	1.308269	3.36E-10
ENSG0000(	SEZ6	chr17	28954901	29006440	2.009224	0.790167	1.356281	3.55E-10
ENSG0000(	AQP1	chr7	30911853	30925517	1.086395	2.99358	-1.44646	3.64E-10
ENSG0000(	ADCYAP1	chr18	904871	912172	0.59087	0.056976	3.320816	3.70E-10
ENSG0000(	NLRC5	chr16	56989485	57083531	2.983189	1.359803	1.142748	3.80E-10
ENSG0000(	LGI1	chr10	93757840	93806272	0.933847	0.096185	3.252427	5.12E-10
ENSG0000(	GPR176	chr15	39799008	39920266	1.001516	2.414202	-1.25497	5.86E-10
ENSG0000(	BDKRB2	chr14	96204679	96244166	0.755362	0.20638	1.880018	6.10E-10
ENSG0000(	CDKN2D	chr19	10566460	10569059	5.951556	2.826775	1.084114	7.77E-10
ENSG0000(	IGSF21	chr1	18107798	18378483	1.422921	0.275253	2.359797	7.90E-10
ENSG0000(	DPF3	chr14	72619296	72894116	0.335043	1.305478	-1.93737	1.17E-09
ENSG0000(	PAPLN	chr14	73237497	73274640	0.06564	0.469171	-2.79402	1.29E-09
ENSG0000(	TEX19	chr17	82359247	82363775	0.707469	2.034893	-1.50951	1.48E-09
ENSG0000(	COL26A1	chr7	1.01E+08	1.02E+08	0.598175	0.060814	3.264812	1.63E-09
ENSG0000(	RBPMS2	chr15	64739891	64775589	4.895668	2.433369	1.018294	1.66E-09
ENSG0000(	CNGB1	chr16	57882340	57971128	4.052229	1.988344	1.036928	1.78E-09
ENSG0000(	CCDC9B	chr15	40331452	40340967	2.849099	1.431747	1.003579	2.12E-09
ENSG0000(	FGD4	chr12	32399529	32646050	0.878839	2.136739	-1.26671	2.13E-09
ENSG0000(	C6orf223	chr6	44000580	44007612	0.337985	1.047651	-1.61279	2.25E-09
ENSG0000(	ETNK2	chr1	2.04E+08	2.04E+08	2.335731	5.615841	-1.24779	2.72E-09
ENSG0000(	PPP2R2C	chr4	6320578	6563600	0.420798	1.35238	-1.66413	2.72E-09
ENSG0000(	ZNF844	chr19	12064731	12081565	0.748766	1.699375	-1.16829	3.19E-09
ENSG0000(	LYPD6	chr2	1.49E+08	1.49E+08	1.393255	2.94414	-1.06469	3.20E-09
ENSG0000(	TCP11L2	chr12	1.06E+08	1.06E+08	0.591011	2.283356	-1.92837	3.59E-09
ENSG0000(	EPHB2	chr1	22710839	22921500	0.355675	0.983779	-1.45024	3.79E-09
ENSG0000(	PM20D1	chr1	2.06E+08	2.06E+08	0.0177	0.646614	-4.87673	4.37E-09
ENSG0000(	SLC30A10	chr1	2.2E+08	2.2E+08	0.443986	0.077898	2.492102	4.56E-09
ENSG0000(	TUBA4A	chr2	2.19E+08	2.19E+08	2.964691	0.976005	1.605967	4.72E-09
ENSG0000(	ARL17B	chr17	46274784	46361797	0.22988	2.056389	-3.12955	4.84E-09
ENSG0000(	GYPE	chr4	1.44E+08	1.44E+08	2.073789	0.610312	1.763747	4.89E-09
ENSG0000(	CDNF	chr10	14819245	14838575	2.469299	5.721477	-1.19447	4.95E-09
ENSG0000(	EFNA1	chr1	1.55E+08	1.55E+08	1.813182	4.367833	-1.25501	5.10E-09
ENSG0000(	ZSCAN23	chr6	28431930	28443502	2.271813	4.600761	-1.00192	5.42E-09
ENSG0000(	TSPAN8	chr12	71125085	71441898	0.63581	2.613664	-2.00975	5.76E-09
ENSG0000(	SERPINB8	chr18	63970029	64019779	0.906593	0.163029	2.469082	6.05E-09
ENSG0000(	SPATA2L	chr16	89696357	89701705	4.269795	2.012554	1.096128	6.35E-09
ENSG0000(	AKAP3	chr12	4615508	4649051	0.221219	1.187362	-2.38728	6.41E-09
ENSG0000(	BCL6B	chr17	7023050	7030290	0.661848	2.423593	-1.84992	6.48E-09
ENSG0000(	TMEM200	chr1	29119429	29123903	0.020171	0.411696	-4.17993	7.18E-09
ENSG0000(	PRR22	chr19	5782960	5784746	1.61466	3.596821	-1.14181	7.44E-09
ENSG0000(	H2AC6	chr6	26124145	26139116	2.145808	4.426893	-1.03104	7.83E-09
ENSG0000(	ST18	chr8	52110839	52460959	0.738924	0.066189	3.391432	7.88E-09
ENSG0000(	DNAH1	chr3	52316319	52400491	0.332253	0.866646	-1.36371	8.96E-09
ENSG0000(	KLLN	chr10	87859158	87863437	0.402346	1.03941	-1.35072	9.44E-09
ENSG0000(	SYT7	chr11	61513714	61581148	0.746658	1.774694	-1.23293	9.95E-09
ENSG0000(	GABRG3	chr15	26971181	27541984	1.40827	0.420795	1.757278	1.05E-08
ENSG0000(	SIX1	chr14	60643421	60658259	0.479831	0.036608	3.644428	1.16E-08
ENSG0000(	FAM72C	chr1	1.44E+08	1.44E+08	1.536166	3.109679	-1.00245	1.24E-08

ENSG0000(	HTR3A	chr11	1.14E+08	1.14E+08	2.271072	0.918912	1.32067	1.31E-08
ENSG0000(	THBS4	chr5	79991311	80083287	2.167701	4.464069	-1.02675	1.85E-08
ENSG0000(	CSKMT	chr11	62665309	62668496	1.140934	2.747326	-1.25022	1.85E-08
ENSG0000(	JAK3	chr19	17824780	17848071	0.587122	1.745806	-1.55562	1.87E-08
ENSG0000(	ALPL	chr1	21509397	21578410	1.477053	3.224004	-1.11058	1.97E-08
ENSG0000(	COL16A1	chr1	31652263	31704319	1.003897	2.663083	-1.3919	2.10E-08
ENSG0000(	DOCK10	chr2	2.25E+08	2.25E+08	0.212641	0.7965	-1.8797	2.14E-08
ENSG0000(	ZCWPW1	chr7	1E+08	1E+08	0.452404	1.60478	-1.80377	2.25E-08
ENSG0000(	RIT2	chr18	42743227	43115691	2.216241	4.978382	-1.15124	2.28E-08
ENSG0000(	WDR31	chr9	1.13E+08	1.13E+08	1.123984	2.51106	-1.14698	2.69E-08
ENSG0000(	CCDC191	chr3	1.14E+08	1.14E+08	0.862904	2.181668	-1.32056	2.95E-08
ENSG0000(	HOGA1	chr10	97584323	97612802	0.437175	1.417438	-1.67327	2.98E-08
ENSG0000(	HTR1A	chr5	63957874	63962507	0.374759	0.019898	4.066336	3.02E-08
ENSG0000(	ID4	chr6	19837370	19842197	0.692722	0.185773	1.891716	3.08E-08
ENSG0000(	PSD2	chr5	1.4E+08	1.4E+08	0.247125	0.898603	-1.8359	3.28E-08
ENSG0000(	METTL7B	chr12	55681678	55684611	0.470547	1.901845	-1.98557	3.41E-08
ENSG0000(	NXPH2	chr2	1.39E+08	1.39E+08	1.654423	0.647541	1.357288	3.79E-08
ENSG0000(	GPR146	chr7	1044546	1059261	0.896155	2.854104	-1.64996	3.98E-08
ENSG0000(	H2BC6	chr6	26172059	26184655	0.89449	2.275923	-1.32932	4.13E-08
ENSG0000(	METTL27	chr7	73834590	73842516	3.232913	7.375426	-1.17511	4.13E-08
ENSG0000(	CACNA1H	chr16	1153106	1221771	0.373209	0.865781	-1.19631	4.54E-08
ENSG0000(	BHLHE41	chr12	26120030	26125037	1.120416	3.260296	-1.52267	4.93E-08
ENSG0000(	CDC42BP(	chr11	64823052	64844653	0.573308	1.633701	-1.49183	4.99E-08
ENSG0000(	FAM161B	chr14	73931501	73950414	0.950459	1.93316	-1.00801	5.07E-08
ENSG0000(	ZNF391	chr6	27374615	27403904	1.811951	3.827953	-1.06187	5.60E-08
ENSG0000(	RAB6B	chr3	1.34E+08	1.34E+08	1.466981	0.517545	1.508306	5.64E-08
ENSG0000(	MAGEE1	chrX	76427710	76431342	1.129435	0.493411	1.206231	5.67E-08
ENSG0000(	KANK4	chr1	62236165	62319434	0.095341	0.524844	-2.41539	5.77E-08
ENSG0000(	PTH2R	chr2	2.08E+08	2.09E+08	0.881258	2.185702	-1.2939	5.89E-08
ENSG0000(	SH2D3C	chr9	1.28E+08	1.28E+08	2.868869	1.318039	1.131268	5.93E-08
ENSG0000(	GPR158	chr10	25174802	25602229	1.175225	0.592649	1.001577	6.96E-08
ENSG0000(	F10	chr13	1.13E+08	1.13E+08	1.259586	3.12405	-1.29395	7.34E-08
ENSG0000(	GLRX	chr5	95751319	95822726	3.77185	1.596219	1.247842	7.41E-08
ENSG0000(	TAS2R4	chr7	1.42E+08	1.42E+08	0.257127	0.662538	-1.34586	8.62E-08
ENSG0000(	MATN2	chr8	97868840	98036724	1.298454	3.033289	-1.21034	8.69E-08
ENSG0000(	ADRA2A	chr10	1.11E+08	1.11E+08	0.522282	0.13947	1.896152	8.83E-08
ENSG0000(	IQSEC3	chr12	66767	178455	0.079189	0.699082	-3.0931	1.04E-07
ENSG0000(	NFAM1	chr22	42380407	42432403	0.281923	0.869411	-1.60144	1.05E-07
ENSG0000(	TMSB15B	chrX	1.04E+08	1.04E+08	1.986751	0.300759	2.709977	1.06E-07
ENSG0000(	SLC4A4	chr4	71062667	71572087	0.426143	0.107299	1.98361	1.12E-07
ENSG0000(	COLEC12	chr18	316737	500722	0.048584	0.361323	-2.84052	1.17E-07
ENSG0000(	FOSL1	chr11	65892049	65900573	2.576553	1.007264	1.36153	1.20E-07
ENSG0000(	TRIM29	chr11	1.2E+08	1.2E+08	0.104052	1.065635	-3.26569	1.29E-07
ENSG0000(	APLN	chrX	1.3E+08	1.3E+08	0.425287	1.229224	-1.51714	1.30E-07
ENSG0000(	CCDC149	chr4	24806117	24980204	0.644649	2.044939	-1.65027	1.31E-07
ENSG0000(	GCHFR	chr15	40764068	40767708	3.465577	7.561862	-1.10902	1.67E-07
ENSG0000(	TCTE1	chr6	44278734	44297698	1.792353	0.73076	1.306459	2.36E-07
ENSG0000(	HSD17B14	chr19	48813018	48836510	2.817899	5.838103	-1.03576	2.37E-07
ENSG0000(	CCDC110	chr4	1.85E+08	1.85E+08	0.473758	1.538511	-1.68068	2.39E-07
ENSG0000(	DYSF	chr2	71453722	71686768	0.017818	0.212258	-3.45981	2.81E-07
ENSG0000(	ARHGAP2	chr4	85475150	86002668	0.799729	2.022224	-1.32237	2.87E-07
ENSG0000(	TMEM74	chr8	1.09E+08	1.09E+08	0.840316	0.322129	1.385954	2.94E-07
ENSG0000(	FAM156A	chrX	52926402	52995472	3.244985	0.604639	2.427382	3.03E-07
ENSG0000(	LAMA1	chr18	6941742	7117797	0.090966	0.449296	-2.2761	3.05E-07
ENSG0000(	SH2D2A	chr1	1.57E+08	1.57E+08	0.396435	1.671951	-2.04765	3.23E-07
ENSG0000(	GYG2	chrX	2828822	2882820	1.294845	2.63982	-1.01243	3.43E-07
ENSG0000(	CFAP70	chr10	73253759	73358859	0.077296	0.465327	-2.53949	3.47E-07
ENSG0000(	PAPPA2	chr1	1.76E+08	1.77E+08	0.082146	0.543755	-2.67647	3.63E-07

ENSG0000(	SORCS2	chr4	7192538	7742836	0.303292	0.828343	-1.43108	3.75E-07
ENSG0000(	RAMP2	chr17	42758447	42763041	4.247641	8.719084	-1.02069	3.80E-07
ENSG0000(	AZIN2	chr1	33081104	33123492	0.340114	1.166751	-1.75412	4.02E-07
ENSG0000(	C1GALT1(	chr2	43675151	43676429	2.157205	0.774861	1.49091	4.12E-07
ENSG0000(	PARP11	chr12	3791047	3873448	0.671474	1.528767	-1.16849	4.38E-07
ENSG0000(	SLC41A2	chr12	1.05E+08	1.05E+08	2.241677	0.731539	1.623178	4.51E-07
ENSG0000(	SCX	chr8	1.44E+08	1.44E+08	0.641773	1.992975	-1.61498	4.51E-07
ENSG0000(	METRNL	chr17	83079609	83095122	2.372744	0.87359	1.449048	5.26E-07
ENSG0000(	ZNF19	chr16	71464555	71565089	1.684693	3.417129	-1.00457	5.94E-07
ENSG0000(	CYP2J2	chr1	59893308	59926773	1.739248	0.727202	1.261814	6.03E-07
ENSG0000(	FEZ1	chr11	1.25E+08	1.26E+08	1.269088	3.319795	-1.36721	6.94E-07
ENSG0000(	TMEM132	chr17	34579487	34639318	0.367523	0.076311	2.252028	7.21E-07
ENSG0000(	SCN2B	chr11	1.18E+08	1.18E+08	0.030088	0.360612	-3.46238	7.67E-07
ENSG0000(	ATOH8	chr2	85751344	85791383	1.501984	0.666853	1.181626	7.77E-07
ENSG0000(	ZNF843	chr16	31432593	31443160	0.111867	0.552727	-2.26537	7.87E-07
ENSG0000(	LAT2	chr7	74199652	74229834	1.713307	0.570705	1.588789	8.01E-07
ENSG0000(	CNTNAP5	chr2	1.24E+08	1.25E+08	0.233734	0.866707	-1.86807	8.51E-07
ENSG0000(	TTC34	chr1	2635976	2801717	0.387292	0.803676	-1.03747	8.51E-07
ENSG0000(	ACSF2	chr17	50426158	50474845	1.942218	4.382719	-1.15547	8.58E-07
ENSG0000(	MEIOB	chr16	1833983	1884294	0.143801	1.015719	-2.76026	1.03E-06
ENSG0000(	ADGRD1	chr12	1.31E+08	1.31E+08	0.424572	1.18755	-1.46113	1.05E-06
ENSG0000(	SLC27A6	chr5	1.29E+08	1.29E+08	2.103213	0.918051	1.204781	1.11E-06
ENSG0000(	NT5C1A	chr1	39659121	39672038	3.477865	1.640671	1.094013	1.12E-06
ENSG0000(	TRPM6	chr9	74722495	74888094	0.315192	0.086073	1.861106	1.13E-06
ENSG0000(	MYOZ1	chr10	73631612	73641474	0.175182	0.982352	-2.4364	1.32E-06
ENSG0000(	ADAMTS1	chr10	70672506	70762441	0.36027	0.763452	-1.06633	1.33E-06
ENSG0000(	CRACDL	chr2	98793846	98936259	0.14374	0.884225	-2.56443	1.35E-06
ENSG0000(	CUX2	chr12	1.11E+08	1.11E+08	0.8864	0.295907	1.587012	1.45E-06
ENSG0000(	C17orf113	chr17	42038232	42050601	0.603521	1.225131	-1.0044	1.46E-06
ENSG0000(	ADARB2	chr10	1177313	1737525	0.01191	0.318853	-4.41143	1.48E-06
ENSG0000(	H2AC15	chr6	27837880	27838375	1.428859	4.995195	-1.7797	1.50E-06
ENSG0000(	C11orf65	chr11	1.08E+08	1.08E+08	0.951911	2.375379	-1.29895	1.52E-06
ENSG0000(	CACNA1G	chr17	50560715	50627474	0.080184	0.307346	-1.90639	1.59E-06
ENSG0000(	MOCOS	chr18	36187497	36272157	0.19932	0.007989	4.372727	1.60E-06
ENSG0000(	INKA1	chr3	49803261	49805030	2.450464	0.954936	1.367927	1.62E-06
ENSG0000(	GABRB1	chr4	46993723	47426447	1.902009	0.5747	1.730672	1.72E-06
ENSG0000(	ITPR3	chr6	33620365	33696574	0.208167	0.453829	-1.10866	1.78E-06
ENSG0000(	DNASE2B	chr1	84398532	84415018	0.042207	0.625431	-3.7203	1.79E-06
ENSG0000(	ITGA7	chr12	55684568	55716043	0.821788	2.080195	-1.32597	1.83E-06
ENSG0000(	HBEGF	chr5	1.4E+08	1.4E+08	2.944245	1.337369	1.154257	2.07E-06
ENSG0000(	ITGA2B	chr17	44372180	44389649	1.080258	2.547472	-1.21752	2.15E-06
ENSG0000(	DENND1C	chr19	6467207	6482557	0.151144	0.876159	-2.47892	2.17E-06
ENSG0000(	TGM2	chr20	38127385	38166578	1.274785	0.545446	1.23643	2.19E-06
ENSG0000(	LTBP2	chr14	74498183	74612378	0.060535	0.323624	-2.37525	2.22E-06
ENSG0000(	TEX22	chr14	1.05E+08	1.05E+08	0.958285	2.256677	-1.22199	2.32E-06
ENSG0000(	SPATA25	chr20	45886489	45887635	0.970385	2.873253	-1.54726	2.40E-06
ENSG0000(	ADAMTSL	chr9	1.34E+08	1.34E+08	0.464224	0.970504	-1.04873	2.46E-06
ENSG0000(	FGF5	chr4	80266599	80336680	0.584234	0.167661	1.794668	2.47E-06
ENSG0000(	OTOG	chr11	17547373	17647150	0.005421	0.14473	-4.41004	2.56E-06
ENSG0000(	RAB26	chr16	2140803	2154165	0.515067	1.696418	-1.6973	2.60E-06
ENSG0000(	HOXD10	chr2	1.76E+08	1.76E+08	0.411027	1.426763	-1.77366	2.70E-06
ENSG0000(	CRYBG3	chr3	97822011	97944984	0.964214	0.481536	1.012286	2.83E-06
ENSG0000(	FRAT1	chr10	97319271	97321915	0.154289	0.743564	-2.2263	2.84E-06
ENSG0000(	RPH3AL	chr17	212389	386254	0.32853	1.52142	-2.17075	3.12E-06
ENSG0000(	PAK5	chr20	9537389	9839041	0.624571	0.212811	1.558871	3.15E-06
ENSG0000(	MARCHF3	chr5	1.27E+08	1.27E+08	1.247309	0.386764	1.688298	3.38E-06
ENSG0000(	H4C3	chr6	26103933	26104337	4.819635	10.40411	-1.0936	3.40E-06
ENSG0000(	SYNGR4	chr19	48364367	48376377	0.760501	2.793821	-1.84915	3.41E-06

ENSG0000(	COL9A3	chr20	62816244	62841159	0.613441	2.064751	-1.72841	3.78E-06
ENSG0000(	NSUN7	chr4	40749925	40811184	0.083228	0.540048	-2.63559	3.81E-06
ENSG0000(	IL1RAPL1	chrX	28587446	29956718	0.565262	0.148182	1.932834	3.86E-06
ENSG0000(	SERPING1	chr11	57597387	57614848	0.404792	1.394578	-1.76032	3.95E-06
ENSG0000(	DLGAP2	chr8	737628	1708476	0.14222	0.619997	-2.0839	4.14E-06
ENSG0000(	CHDH	chr3	53812335	53846419	0.025579	0.262833	-3.23972	4.33E-06
ENSG0000(	HAPLN3	chr15	88877294	88895597	0.842282	1.885064	-1.14597	4.34E-06
ENSG0000(	SNX22	chr15	64151715	64157481	1.10758	2.695947	-1.26983	4.70E-06
ENSG0000(	ZNF165	chr6	28080568	28089563	0.503519	1.204649	-1.24318	4.80E-06
ENSG0000(	GABRE	chrX	1.52E+08	1.52E+08	0.326731	1.097912	-1.72511	4.82E-06
ENSG0000(	SLC28A3	chr9	84275457	84340758	0.009315	0.226874	-4.29678	4.86E-06
ENSG0000(	IFIT2	chr10	89283694	89309271	0.487023	1.029488	-1.06299	4.86E-06
ENSG0000(	STING1	chr5	1.39E+08	1.39E+08	1.065638	2.282821	-1.08235	5.03E-06
ENSG0000(	DTX1	chr12	1.13E+08	1.13E+08	0.050522	0.541161	-3.28878	5.17E-06
ENSG0000(	MRGPRF	chr11	69004395	69013382	0.134128	0.668982	-2.27936	5.25E-06
ENSG0000(	CD40	chr20	46118278	46129863	1.15876	2.564199	-1.12707	5.39E-06
ENSG0000(	LRRIQ1	chr12	85036314	85264457	1.361377	0.578105	1.241971	5.47E-06
ENSG0000(	ICAM4	chr19	10286955	10288522	0.07883	0.669161	-2.99071	5.87E-06
ENSG0000(	TP53TG3D	chr16	32252719	32255922	0.248698	1.13792	-2.15164	5.89E-06
ENSG0000(	CD72	chr9	35609979	35646810	0.634995	1.952198	-1.5967	5.91E-06
ENSG0000(	OSGIN1	chr16	83931311	83966332	1.149354	0.355937	1.693915	6.55E-06
ENSG0000(	LRRC32	chr11	76657524	76670747	0.030364	0.322517	-3.28891	6.61E-06
ENSG0000(	PDE9A	chr21	42653621	42775509	0.0156	0.382739	-4.2994	6.73E-06
ENSG0000(	GRHL1	chr2	9951693	10002277	1.27997	0.529481	1.287181	6.82E-06
ENSG0000(	RAB38	chr11	88113251	88175443	1.509806	3.337396	-1.12489	7.01E-06
ENSG0000(	C4orf48	chr4	2041993	2043970	19.72504	8.791696	1.175526	7.17E-06
ENSG0000(	H2AJ	chr12	14774383	14778002	1.273866	2.666328	-1.04877	7.17E-06
ENSG0000(	ADAMTSL	chr1	1.51E+08	1.51E+08	0.369253	0.905996	-1.27511	7.25E-06
ENSG0000(	MAPK15	chr8	1.44E+08	1.44E+08	0.846384	2.227056	-1.37768	7.32E-06
ENSG0000(	ESPNL	chr2	2.38E+08	2.38E+08	0.306984	0.776553	-1.31851	7.51E-06
ENSG0000(	ACAD11	chr3	1.33E+08	1.33E+08	0.390943	1.10817	-1.47925	9.80E-06
ENSG0000(	SLC12A5	chr20	46021690	46060150	1.945241	0.866132	1.1743	1.03E-05
ENSG0000(	CDH23	chr10	71396920	71815947	0.29338	0.692947	-1.22572	1.08E-05
ENSG0000(	NKD1	chr16	50548396	50649249	0.228693	0.508397	-1.13307	1.16E-05
ENSG0000(	ADRB2	chr5	1.49E+08	1.49E+08	0.941368	0.438905	1.115621	1.23E-05
ENSG0000(	RIPOR3	chr20	50586108	50691542	0.37559	1.256712	-1.72574	1.25E-05
ENSG0000(	ADAP1	chr7	897900	955407	2.357771	0.972983	1.285381	1.31E-05
ENSG0000(	TPBG	chr6	82363206	82367420	0.564102	0.194749	1.532357	1.38E-05
ENSG0000(	PMFBP1	chr16	72112157	72176878	1.347175	2.835855	-1.06058	1.39E-05
ENSG0000(	PNMA8C	chr19	46424697	46428951	0.106959	0.360275	-1.72137	1.42E-05
ENSG0000(	GBGT1	chr9	1.33E+08	1.33E+08	0.482012	1.370292	-1.4832	1.45E-05
ENSG0000(	TPD52L1	chr6	1.25E+08	1.25E+08	0	0.398379	-6.27512	1.51E-05
ENSG0000(	BACH2	chr6	89926528	90296908	1.205454	0.559112	1.118488	1.52E-05
ENSG0000(	MAGED4	chrX	52184823	52192268	1.503529	0.349958	2.105187	1.59E-05
ENSG0000(	TRANK1	chr3	36826819	36945057	0.040704	0.163554	-1.96939	1.60E-05
ENSG0000(	TMCO4	chr1	19682213	19799945	0.772296	1.890006	-1.27399	1.60E-05
ENSG0000(	GAS2	chr11	22625509	22813055	1.022028	2.413862	-1.22081	1.72E-05
ENSG0000(	CAMK2A	chr5	1.5E+08	1.5E+08	0.040566	0.310764	-2.85263	1.75E-05
ENSG0000(	SMIM11A	chr21	34375480	34416961	0.440737	1.901626	-2.08973	1.76E-05
ENSG0000(	NEK11	chr3	1.31E+08	1.31E+08	0.474586	1.28443	-1.41211	1.79E-05
ENSG0000(	NKX2-5	chr5	1.73E+08	1.73E+08	0.092848	0.911755	-3.19908	1.81E-05
ENSG0000(	DLX4	chr17	49968970	49974959	0.081102	0.624992	-2.85354	1.98E-05
ENSG0000(	DLX5	chr7	97020396	97024950	1.839094	0.696351	1.39926	2.07E-05
ENSG0000(	AC007325	chrKI2707	138082	161852	0.057854	0.377513	-2.63986	2.25E-05
ENSG0000(	PDZD2	chr5	31639131	32110932	0.033794	0.314228	-3.08946	2.43E-05
ENSG0000(	DNAH6	chr2	84516455	84819589	0.083525	0.316889	-1.88678	2.49E-05
ENSG0000(	RAD51AP2	chr2	17510584	17518439	0.136297	0.432929	-1.64245	2.50E-05
ENSG0000(	MYRFL	chr12	69825227	69959097	0.377574	0.051075	2.811222	2.50E-05

ENSG0000(	CHN2	chr7	29146569	29514328	0.217859	1.012596	-2.17719	2.59E-05
ENSG0000(	CA14	chr1	1.5E+08	1.5E+08	0	0.364077	-6.20326	2.64E-05
ENSG0000(	VIPR1	chr3	42489299	42537573	0.771116	0.198568	1.953622	2.68E-05
ENSG0000(	WHRN	chr9	1.14E+08	1.15E+08	0.136315	0.591772	-2.08351	2.78E-05
ENSG0000(	WBP2NL	chr22	41998725	42058456	0.513687	1.510731	-1.5393	2.88E-05
ENSG0000(	NEUROD1	chr2	1.82E+08	1.82E+08	0	0.287009	-6.11925	3.16E-05
ENSG0000(	MUC4	chr3	1.96E+08	1.96E+08	0.204369	0.417498	-1.0178	3.20E-05
ENSG0000(	RALYL	chr8	84182787	84921844	0.030737	0.381943	-3.45206	3.21E-05
ENSG0000(	ZNF396	chr18	35366694	35377337	0.327734	0.926887	-1.47716	3.25E-05
ENSG0000(	ODAM	chr4	70196496	70204576	3.761668	1.737232	1.118424	3.34E-05
ENSG0000(	ARMC3	chr10	22928024	23038523	0.171021	0.732341	-2.06402	3.48E-05
ENSG0000(	C1orf158	chr1	12746200	12763699	0.152245	0.668986	-2.09723	3.51E-05
ENSG0000(	KCNA3	chr1	1.11E+08	1.11E+08	0.193525	0	6.253075	3.58E-05
ENSG0000(	TNNT1	chr19	55132698	55149206	0.331347	1.471042	-2.10078	3.65E-05
ENSG0000(	RILP	chr17	1646145	1649866	1.14701	0.337586	1.766923	3.66E-05
ENSG0000(	KIF17	chr1	20664014	20718017	0.597567	1.369747	-1.17986	3.68E-05
ENSG0000(	TMEM81	chr1	2.05E+08	2.05E+08	1.712723	0.720053	1.255466	3.85E-05
ENSG0000(	STEAP1B	chr7	22419444	22727613	1.034067	2.244019	-1.09772	3.92E-05
ENSG0000(	KCNK6	chr19	38319845	38332076	0.122284	0.426763	-1.77062	3.99E-05
ENSG0000(	APOL4	chr22	36189124	36204840	0.088921	0.63231	-2.76344	4.08E-05
ENSG0000(	PDZD7	chr10	1.01E+08	1.01E+08	0.483517	1.147248	-1.22784	4.28E-05
ENSG0000(	DCST2	chr1	1.55E+08	1.55E+08	0.374933	1.108557	-1.53784	4.32E-05
ENSG0000(	TESK2	chr1	45343883	45491163	0.089333	0.442223	-2.26932	4.35E-05
ENSG0000(	PTPRE	chr10	1.28E+08	1.28E+08	1.03644	0.428948	1.286079	4.39E-05
ENSG0000(	INAFM1	chr19	47274885	47275723	8.460081	3.718056	1.194896	4.47E-05
ENSG0000(	GRM7	chr3	6770001	7741533	1.06653	0.463326	1.215785	4.59E-05
ENSG0000(	NATD1	chr17	21238870	21253410	0.174394	0.426516	-1.2686	4.63E-05
ENSG0000(	OSBPL10	chr3	31657890	32077580	0.35998	1.182016	-1.68948	4.72E-05
ENSG0000(	GALNT18	chr11	11270877	11622005	0.627756	1.39457	-1.13073	5.04E-05
ENSG0000(	TOX3	chr16	52436417	52547802	0.23093	0.659226	-1.48876	5.06E-05
ENSG0000(	PLAAT1	chr3	1.93E+08	1.93E+08	0.420997	1.475921	-1.78095	5.13E-05
ENSG0000(	TSGA10	chr2	98997261	99154964	0.670803	1.545823	-1.18659	5.25E-05
ENSG0000(	PTGIR	chr19	46620468	46625089	1.350196	2.753528	-1.00952	5.30E-05
ENSG0000(	PPP1R9A	chr7	94907202	95296415	0.242013	0.084445	1.526331	5.32E-05
ENSG0000(	FFAR2	chr19	35443907	35451767	2.110268	1.054051	1.010483	5.43E-05
ENSG0000(	KCNJ9	chr1	1.6E+08	1.6E+08	0.012053	0.168224	-3.62573	5.49E-05
ENSG0000(	TAGAP	chr6	1.59E+08	1.59E+08	0.03485	0.25666	-2.8016	5.81E-05
ENSG0000(	MRPS28	chr8	79918717	80030289	5.264339	2.388913	1.148264	5.89E-05
ENSG0000(	NTN3	chr16	2471297	2474145	0.521414	1.33469	-1.33616	6.02E-05
ENSG0000(	SLC12A8	chr3	1.25E+08	1.25E+08	0.378359	1.107655	-1.52551	6.20E-05
ENSG0000(	DTNA	chr18	34493290	34891844	0.422761	0.081772	2.345347	6.44E-05
ENSG0000(	VIP	chr6	1.53E+08	1.53E+08	0.619166	0.141611	2.113683	6.81E-05
ENSG0000(	SYT13	chr11	45240302	45286341	0.030598	0.262559	-2.9797	7.02E-05
ENSG0000(	CMPK2	chr2	6840570	6866635	0.936264	0.313194	1.576812	7.06E-05
ENSG0000(	NPIPB2	chr16	11927259	11976643	0.284687	1.168577	-1.99493	7.17E-05
ENSG0000(	NPPC	chr2	2.32E+08	2.32E+08	1.235516	0.30089	2.025698	7.60E-05
ENSG0000(	GALNT13	chr2	1.54E+08	1.54E+08	1.642541	0.739578	1.159144	7.92E-05
ENSG0000(	TPPP	chr5	659862	693352	0.173565	0.044032	1.984919	8.48E-05
ENSG0000(	ZNF418	chr19	57921882	57935402	0.459522	1.16863	-1.32623	8.67E-05
ENSG0000(	CCDC146	chr7	77122434	77329533	0.165113	0.819487	-2.26792	8.80E-05
ENSG0000(	LOXL4	chr10	98247690	98268194	0.084384	0.31071	-1.84222	9.09E-05
ENSG0000(	ICAM5	chr19	10289952	10296778	0.278045	0.870263	-1.62032	9.10E-05
ENSG0000(	HLA-DOA	chr6	33004182	33009591	0.289156	1.102642	-1.89589	9.31E-05
ENSG0000(	KCNH6	chr17	63523334	63548977	0.675298	0.274492	1.309607	9.47E-05
ENSG0000(	CD200R1	chr3	1.13E+08	1.13E+08	0.363874	0.0601	2.550956	9.61E-05
ENSG0000(	CRISPLD2	chr16	84819985	84920768	0.620817	0.15574	1.987524	0.000105
ENSG0000(	LATS2	chr13	20973036	21061586	0.242919	0.650017	-1.40188	0.000112
ENSG0000(	JAG1	chr20	10637684	10673999	1.188632	0.541098	1.143293	0.000112

ENSG0000(	MINAR2	chr5	1.3E+08	1.3E+08	0.260001	0.033099	2.878823	0.000115
ENSG0000(	TLE6	chr19	2977538	2995179	1.404834	3.008391	-1.07976	0.000123
ENSG0000(	EYS	chr6	63719980	65707226	0.217096	0.038571	2.45564	0.00013
ENSG0000(	NRG2	chr5	1.4E+08	1.4E+08	0.177945	0.559323	-1.62178	0.000132
ENSG0000(	PHACTR1	chr6	12716554	13287843	1.017477	0.439795	1.219391	0.000136
ENSG0000(	POU2F2	chr19	42086110	42196585	0.296401	0.862348	-1.51992	0.000139
ENSG0000(	CEL	chr9	1.33E+08	1.33E+08	0.286733	0.729364	-1.32524	0.00014
ENSG0000(	BCL2L15	chr1	1.14E+08	1.14E+08	0.467388	0.13206	1.799934	0.000149
ENSG0000(	IDUA	chr4	986997	1004564	0.237118	0.921919	-1.92114	0.00015
ENSG0000(	TOGARAN	chr2	28956611	29061373	0.382858	0.782865	-1.0178	0.000151
ENSG0000(	STK32A	chr5	1.47E+08	1.47E+08	0.199536	0.674817	-1.72163	0.000159
ENSG0000(	ANKRD23	chr2	96824526	96857934	0.351912	0.902579	-1.3359	0.000171
ENSG0000(	BMP7	chr20	57168753	57266641	0.304383	0.997142	-1.6826	0.000174
ENSG0000(	PKD1L1	chr7	47740202	47948466	1.064959	0.505275	1.083245	0.000175
ENSG0000(	EVPL	chr17	76004502	76027306	0.248005	0.576201	-1.20076	0.000176
ENSG0000(	PHKG1	chr7	56080283	56092996	0.886449	0.202411	2.112508	0.000177
ENSG0000(	PPARGC1/	chr4	23755041	23904089	1.660043	0.805947	1.051936	0.000177
ENSG0000(	AC099489	chr16	11359845	11527245	0.084112	0.368138	-2.076	0.000182
ENSG0000(	DOCK3	chr3	50674927	51384198	0.064245	0.008638	2.817493	0.000189
ENSG0000(	AKAP4	chrX	50190777	50201007	0.469105	0.114052	2.031504	0.000202
ENSG0000(	VWF	chr12	5948877	6124770	0.442695	1.15994	-1.3739	0.000202
ENSG0000(	ZNF467	chr7	1.5E+08	1.5E+08	0.337212	0.872059	-1.34718	0.000212
ENSG0000(	ZNF385C	chr17	42025576	42098479	0.118553	0.540132	-2.13919	0.000213
ENSG0000(	RIBC1	chrX	53422690	53431120	0.866645	1.891121	-1.10527	0.000214
ENSG0000(	ENPP3	chr6	1.32E+08	1.32E+08	0.027633	0.293567	-3.25749	0.00022
ENSG0000(	CBLN4	chr20	55997357	56005519	0.112294	0	5.821816	0.00022
ENSG0000(	GABRA4	chr4	46918900	46993581	0.548915	0.236227	1.221448	0.000235
ENSG0000(	GDF15	chr19	18374731	18389176	2.718024	1.206569	1.181044	0.000244
ENSG0000(	SLC16A13	chr17	7036015	7040117	0.365167	1.010733	-1.44356	0.000259
ENSG0000(	HTR1D	chr1	23191895	23194729	0.017738	0.183458	-3.1915	0.000259
ENSG0000(	MT1X	chr16	56682470	56684196	1.983915	0.806757	1.303155	0.000261
ENSG0000(	CNTN3	chr3	74262568	74521140	0.183645	0.018852	3.14876	0.00027
ENSG0000(	ADAM28	chr8	24294069	24359014	0.327989	0.033073	3.149418	0.000284
ENSG0000(	TEKT2	chr1	36084094	36088275	0.515671	1.272589	-1.28185	0.000292
ENSG0000(	DNAH7	chr2	1.96E+08	1.96E+08	0.068157	0.270093	-1.95044	0.00031
ENSG0000(	ADRA2B	chr2	96112876	96116571	0.14374	0.386422	-1.4007	0.000327
ENSG0000(	TAS2R14	chr12	10937408	11171573	1.297695	0.602542	1.109481	0.000336
ENSG0000(	GRM2	chr3	51707068	51718613	0.470112	0.160316	1.547657	0.00034
ENSG0000(	NOXRED1	chr14	77394021	77423517	0.115377	0.553105	-2.21495	0.000342
ENSG0000(	CFAP69	chr7	90245174	90311063	0.247367	0.710853	-1.49141	0.000357
ENSG0000(	RFTN2	chr2	1.98E+08	1.98E+08	0.161407	0.552321	-1.73876	0.000361
ENSG0000(	PPM1J	chr1	1.13E+08	1.13E+08	0.115527	0.526011	-2.13084	0.000363
ENSG0000(	SIK1	chr21	43414483	43427131	0.492225	0.065608	2.816625	0.000382
ENSG0000(	GCOM1	chr15	57591908	57714745	0.078254	0.344134	-2.08262	0.000398
ENSG0000(	SLC12A1	chr15	48178438	48304078	0.111713	0.334808	-1.56083	0.000403
ENSG0000(	NEBL	chr10	20779973	21293011	0.309486	0.768525	-1.29095	0.000412
ENSG0000(	H3C3	chr6	26045384	26045869	0.104215	1.013919	-3.11739	0.000413
ENSG0000(	BRINP3	chr1	1.9E+08	1.9E+08	0.31725	0	5.716661	0.000428
ENSG0000(	FGD3	chr9	92947523	93036236	0.397502	0.847998	-1.07326	0.00043
ENSG0000(	RBFOX1	chr16	5239802	7713340	0.222009	0.03728	2.492067	0.000432
ENSG0000(	LRRC2	chr3	46515385	46580099	0.025578	0.251111	-3.11816	0.000434
ENSG0000(	POSTN	chr13	37562583	37598844	0.144125	0	5.717692	0.00044
ENSG0000(	OLFML2A	chr9	1.25E+08	1.25E+08	0.0668	0.235708	-1.77608	0.000452
ENSG0000(	B4GALT6	chr18	31622247	31685836	0.568308	0.226479	1.330203	0.000458
ENSG0000(	LRRC56	chr11	537527	554912	0.311269	0.686191	-1.11921	0.000473
ENSG0000(	ANKDD1B	chr5	75611182	75681773	1.409352	0.657668	1.109846	0.000478
ENSG0000(	CPNE4	chr3	1.32E+08	1.32E+08	0.735815	0.304702	1.277919	0.000478
ENSG0000(	SLC7A8	chr14	23125295	23183674	0.095291	0.418336	-2.083	0.000478

ENSG0000(	RASD1	chr17	17494437	17496395	1.445027	0.723581	1.006246	0.000483
ENSG0000(	PRICKLE3	chrX	49174802	49186528	0.212231	0.733549	-1.74944	0.000485
ENSG0000(	OGN	chr9	92383268	92404696	0.078679	0.407582	-2.30087	0.00049
ENSG0000(	ZDHHC22	chr14	77131270	77142734	0.560037	1.27088	-1.16489	0.000498
ENSG0000(	YES1	chr18	721588	812546	0.195742	0.497884	-1.32217	0.000505
ENSG0000(	LPAR5	chr12	6618835	6635960	0.39507	0.87999	-1.13803	0.000506
ENSG0000(	RTL1	chr14	1.01E+08	1.01E+08	0.448785	0.221248	1.025827	0.00051
ENSG0000(	OPRL1	chr20	64080082	64100643	0.155268	0.413255	-1.38908	0.000517
ENSG0000(	TEPP	chr16	57976435	57988116	0.3547	1.073196	-1.567	0.000526
ENSG0000(	LCN12	chr9	1.37E+08	1.37E+08	0.435887	1.507111	-1.74936	0.000527
ENSG0000(	ERVFRD-1	chr6	11102489	11111725	0.127097	0.375722	-1.53198	0.000535
ENSG0000(	CFAP58	chr10	1.04E+08	1.04E+08	0.20402	0.611781	-1.55786	0.000539
ENSG0000(	EVA1C	chr21	32412006	32515397	0.324966	0.922558	-1.4758	0.000544
ENSG0000(	NRTN	chr19	5823802	5828324	0.227698	0.818856	-1.80394	0.000548
ENSG0000(	ELOVL7	chr5	60751791	60844274	0.556711	0.205431	1.432317	0.000556
ENSG0000(	FAM178B	chr2	96875882	96986580	0.024602	0.408205	-3.7218	0.000563
ENSG0000(	TSPAN10	chr17	81637171	81648749	0.181426	0.605621	-1.70269	0.000577
ENSG0000(	SCARA5	chr8	27869883	27992673	0.030695	0.2188	-2.72868	0.000611
ENSG0000(	BCHE	chr3	1.66E+08	1.66E+08	0.441388	0.117253	1.899482	0.000614
ENSG0000(	FER1L5	chr2	96642737	96704887	0.01707	0.101052	-2.47349	0.000619
ENSG0000(	BSPRY	chr9	1.13E+08	1.13E+08	0.167665	0.528243	-1.62448	0.000638
ENSG0000(	ALOX12B	chr17	8072636	8087716	0.35418	1.039986	-1.52552	0.000644
ENSG0000(	NUAK2	chr1	2.05E+08	2.05E+08	0.074467	0.268614	-1.80497	0.000649
ENSG0000(	SLC44A5	chr1	75202131	75611116	0.050393	0.232858	-2.14593	0.000656
ENSG0000(	NLRP1	chr17	5499427	5619424	0.024971	0.147001	-2.47351	0.000658
ENSG0000(	USH2A	chr1	2.16E+08	2.16E+08	0.130627	0.303399	-1.20014	0.000661
ENSG0000(	MMRN2	chr10	86935540	86969481	0.021361	0.345551	-3.71752	0.000663
ENSG0000(	CHRNE	chr17	4897771	4934438	0.013639	0.220635	-3.71752	0.000664
ENSG0000(	DENND1B	chr1	1.98E+08	1.98E+08	0.35282	0.086658	1.991301	0.000664
ENSG0000(	TXNDC5	chr6	7881517	7910788	0.660434	0.237116	1.481553	0.000683
ENSG0000(	TRPM8	chr2	2.34E+08	2.34E+08	0.93304	0.416432	1.169827	0.000688
ENSG0000(	ISG20	chr15	88636153	88656483	1.153121	0.529153	1.13048	0.000713
ENSG0000(	SYTL2	chr11	85694224	85811159	0.144917	0.458845	-1.62541	0.000734
ENSG0000(	CATSPERE	chr1	2.44E+08	2.45E+08	0.101775	0.433974	-2.0351	0.000736
ENSG0000(	NLGN3	chrX	71144831	71171201	0.075531	0.270141	-1.80236	0.000746
ENSG0000(	C6	chr5	41142116	41261438	0.785539	1.594999	-1.00181	0.000763
ENSG0000(	NHLRC1	chr6	18120440	18122677	0.237869	0.634878	-1.39785	0.000765
ENSG0000(	C19orf71	chr19	3539154	3544030	1.313172	2.936698	-1.13741	0.000768
ENSG0000(	SH2D6	chr2	85418931	85437029	0.221877	0.723105	-1.66555	0.00077
ENSG0000(	ANKRD62	chr18	12093843	12129764	0.08237	0.253229	-1.58915	0.00078
ENSG0000(	SYT17	chr16	19167971	19268332	0.381267	1.021675	-1.39603	0.000787
ENSG0000(	YJEFN3	chr19	19528861	19537581	0.590719	1.290599	-1.11244	0.000802
ENSG0000(	MYCBPAP	chr17	50508384	50531501	0.089179	0.452867	-2.28602	0.000818
ENSG0000(	SPATA17	chr1	2.18E+08	2.18E+08	0.570128	1.182192	-1.03531	0.000831
ENSG0000(	ACTG2	chr2	73892314	73919865	0.089964	0.578794	-2.5966	0.000839
ENSG0000(	CFAP157	chr9	1.28E+08	1.28E+08	0	0.147252	-5.62391	0.00086
ENSG0000(	LHX6	chr9	1.22E+08	1.22E+08	0.214495	0.6077	-1.47617	0.000868
ENSG0000(	TMEM232	chr5	1.1E+08	1.11E+08	0.533501	1.219555	-1.17382	0.000877
ENSG0000(	SCGB1D2	chr11	62242239	62244812	0	0.68868	-5.62746	0.000878
ENSG0000(	TIGD4	chr4	1.53E+08	1.53E+08	0.300711	0.693046	-1.18041	0.000883
ENSG0000(	DUSP2	chr2	96143169	96145440	0.617575	1.391072	-1.15248	0.000913
ENSG0000(	EAF2	chr3	1.22E+08	1.22E+08	2.328619	1.150285	1.029728	0.000919
ENSG0000(	TSTD1	chr1	1.61E+08	1.61E+08	0.562726	1.731582	-1.58904	0.000984
ENSG0000(	LONRF3	chrX	1.19E+08	1.19E+08	0.064211	0.274818	-2.03531	0.000988
ENSG0000(	KCNN3	chr1	1.55E+08	1.55E+08	0.083431	0.2325	-1.44691	0.000989
ENSG0000(	CACNA1A	chr19	13206442	13633025	0.007401	0.119463	-3.71525	0.001025
ENSG0000(	CARNS1	chr11	67414968	67425607	0.046028	0.226465	-2.22881	0.001025
ENSG0000(	OAZ3	chr1	1.52E+08	1.52E+08	1.702478	0.781257	1.131053	0.001072

ENSG0000(	SPNS2	chr17	4498881	4539035	0.162494	0.62131	-1.89491	0.00108
ENSG0000(	PRR29	chr17	63998351	64004305	0.08633	0.423584	-2.22919	0.001109
ENSG0000(	CSPG4	chr15	75674322	75712848	0.06426	0.162388	-1.31867	0.001121
ENSG0000(	TMIE	chr3	46694528	46710886	0.18798	0.61276	-1.66903	0.001145
ENSG0000(	CYP4F11	chr19	15912367	15934867	0.043986	0.214867	-2.22785	0.001161
ENSG0000(	ASPRV1	chr2	69960089	69962265	0.186454	0.526358	-1.46803	0.001192
ENSG0000(	GLIPR1L2	chr12	75391089	75432688	0.134455	0.522788	-1.91033	0.001223
ENSG0000(	PTGIS	chr20	49503874	49568137	0.029407	0.164404	-2.40773	0.001225
ENSG0000(	LIMS2	chr2	1.28E+08	1.28E+08	0.026226	0.227129	-2.95934	0.001272
ENSG0000(	AK7	chr14	96392128	96489427	0.685416	1.44613	-1.05786	0.001287
ENSG0000(	BATF3	chr1	2.13E+08	2.13E+08	1.161621	0.511532	1.184981	0.001294
ENSG0000(	LRRC39	chr1	1E+08	1E+08	0.581371	0.217887	1.413737	0.001349
ENSG0000(	MFSD4A	chr1	2.06E+08	2.06E+08	0.334976	0.841574	-1.30424	0.001367
ENSG0000(	SPON1	chr11	13962723	14268133	0.225676	0.52081	-1.18903	0.001382
ENSG0000(	KRT80	chr12	52168996	52192014	0.074952	0	5.481304	0.001456
ENSG0000(	PILRA	chr7	1E+08	1E+08	0.683849	1.605114	-1.20467	0.001518
ENSG0000(	CST6	chr11	66012008	66013505	0.042471	0.613899	-3.53087	0.001571
ENSG0000(	GOLGA8T	chr15	30135149	30145567	0.225999	0	5.481741	0.001574
ENSG0000(	UGT8	chr4	1.15E+08	1.15E+08	0.121089	0	5.481742	0.001575
ENSG0000(	MYLK4	chr6	2663629	2750922	0.031246	0.124002	-1.93231	0.00158
ENSG0000(	MOGAT1	chr2	2.23E+08	2.23E+08	0.172273	0.642105	-1.8486	0.0016
ENSG0000(	CRACR2A	chr12	3606633	3764819	0.008028	0.116331	-3.53156	0.001627
ENSG0000(	ATOH1	chr4	93828753	93830964	0.160116	0.011943	3.49062	0.001629
ENSG0000(	ISG15	chr1	1001138	1014540	2.15057	0.905674	1.263148	0.001635
ENSG0000(	CRPPA	chr7	16087525	16502504	0.655004	0.299261	1.133785	0.001714
ENSG0000(	UBASH3B	chr11	1.23E+08	1.23E+08	0.475092	0.118125	1.991097	0.001727
ENSG0000(	YAP1	chr11	1.02E+08	1.02E+08	0.052601	0.234372	-2.09127	0.001796
ENSG0000(	WNT4	chr1	22117313	22143969	0.119111	0.440512	-1.84723	0.00181
ENSG0000(	AC007325	chrKI2707	131494	137392	0.645852	1.470211	-1.16822	0.001821
ENSG0000(	RPP21	chr6	30345131	30346884	2.150363	0.983999	1.133804	0.001832
ENSG0000(	SPAG8	chr9	35808045	35812272	0.565665	1.265112	-1.14231	0.001936
ENSG0000(	CMYA5	chr5	79689836	79800240	0.092377	0.219145	-1.22533	0.001937
ENSG0000(	PCDHGB3	chr5	1.41E+08	1.42E+08	0.500899	0.245511	1.035611	0.001954
ENSG0000(	RAD9B	chr12	1.11E+08	1.11E+08	0.158547	0.472742	-1.54617	0.002004
ENSG0000(	REEP1	chr2	86213993	86338083	0.753118	0.335169	1.173305	0.002005
ENSG0000(	COL1A2	chr7	94394895	94431227	0.414228	0.117969	1.803917	0.002127
ENSG0000(	RTL9	chrX	1.1E+08	1.1E+08	0.013489	0.111413	-2.87348	0.002161
ENSG0000(	SLC23A3	chr2	2.19E+08	2.19E+08	0	0.232372	-5.49795	0.002172
ENSG0000(	TBX4	chr17	61452404	61485110	0.035011	0.284439	-2.8723	0.002203
ENSG0000(	RASGRP2	chr11	64726911	64745456	0.10652	0.505712	-2.17057	0.00224
ENSG0000(	SPATS1	chr6	44342650	44380179	0.457769	0.140296	1.688449	0.002288
ENSG0000(	CYP1A1	chr15	74719542	74725536	0.524138	0.196944	1.403236	0.0023
ENSG0000(	COL19A1	chr6	69866556	70212468	0.152629	0.011339	3.492078	0.002302
ENSG0000(	KIF25	chr6	1.68E+08	1.68E+08	0.123659	0.4697	-1.87748	0.002338
ENSG0000(	GRIN2D	chr19	48394875	48444931	0.029763	0.122329	-1.97783	0.002493
ENSG0000(	EFCAB8	chr20	32858923	32961609	0.053857	0.329156	-2.50928	0.002632
ENSG0000(	AGBL2	chr11	47659591	47715389	0.347341	0.855077	-1.27434	0.00266
ENSG0000(	DPYS	chr8	1.04E+08	1.04E+08	0.306453	0.976061	-1.63017	0.002714
ENSG0000(	FAM180A	chr7	1.36E+08	1.36E+08	0	0.191298	-5.36843	0.002719
ENSG0000(	CTSS	chr1	1.51E+08	1.51E+08	0.116244	0.479187	-1.97873	0.00274
ENSG0000(	FNDC11	chr20	63547891	63556695	0.3744	0.909661	-1.25525	0.002749
ENSG0000(	FOLR1	chr11	72189558	72196323	0.116847	0.53014	-2.10572	0.002773
ENSG0000(	NOTCH2N	chr1	1.46E+08	1.46E+08	0	0.190293	-5.36927	0.002814
ENSG0000(	ZG16B	chr16	2830169	2839585	0.031961	0.42347	-3.42587	0.002817
ENSG0000(	HS3ST2	chr16	22814162	22916338	0.494722	0.192639	1.367096	0.002817
ENSG0000(	WDR93	chr15	89690811	89743638	0.014257	0.188893	-3.42587	0.002818
ENSG0000(	RBKS	chr2	27781379	27890681	1.150936	2.323305	-1.00051	0.002822
ENSG0000(	GLT8D2	chr12	1.04E+08	1.04E+08	0.285191	0.811506	-1.47465	0.002904

ENSG0000(	YWHAH	chr22	31944522	31957603	98.06753	48.49844	1.075662	5.09E-14
ENSG0000(	PGM5	chr9	68328308	68531061	0.013817	0.183558	-3.42666	0.003006
ENSG0000(	DMD	chrX	31097677	33339441	0.115832	0.294215	-1.31489	0.003019
ENSG0000(	TMEFF2	chr2	1.92E+08	1.92E+08	0.228426	0.044342	2.300049	0.003039
ENSG0000(	CDX1	chr5	1.5E+08	1.5E+08	0.3569	0.834328	-1.19822	0.003069
ENSG0000(	MCMDC2	chr8	66870749	66922048	0.147881	0.409958	-1.44487	0.003093
ENSG0000(	GLP1R	chr6	39048781	39091303	0.034136	0.112319	-1.68173	0.003102
ENSG0000(	SMIM10L2	chrX	1.35E+08	1.35E+08	0.048282	0.154308	-1.63134	0.003131
ENSG0000(	ATP2B3	chrX	1.54E+08	1.54E+08	0.149454	0.345061	-1.18869	0.003146
ENSG0000(	SLC16A11	chr17	7041621	7044092	0.045113	0.292516	-2.58381	0.003486
ENSG0000(	CGA	chr6	87085498	87095106	0.486185	0.037554	3.38808	0.00349
ENSG0000(	ZNF98	chr19	22391019	22532485	1.808063	0.831571	1.117242	0.003532
ENSG0000(	TNFSF18	chr1	1.73E+08	1.73E+08	0.227551	0.038097	2.470964	0.003541
ENSG0000(	GNAT2	chr1	1.1E+08	1.1E+08	0.220279	0.757875	-1.74454	0.003555
ENSG0000(	APOBEC3I	chr22	39021113	39033277	0.104633	0.383967	-1.82198	0.003665
ENSG0000(	NPIPA9	chr16	18358086	18379331	1.489835	3.729451	-1.30394	0.00367
ENSG0000(	FAM166C	chr2	26562585	26579532	0.44442	1.134587	-1.32033	0.003707
ENSG0000(	TNK1	chr17	7380534	7389774	0.042186	0.326282	-2.78156	0.003707
ENSG0000(	GPR37	chr7	1.25E+08	1.25E+08	0.071665	0.009626	2.740751	0.003766
ENSG0000(	NBPF14	chr1	1.49E+08	1.49E+08	0.081922	0.226807	-1.43407	0.003787
ENSG0000(	GCGR	chr17	81804132	81814008	0	0.237823	-5.37421	0.003791
ENSG0000(	MSH4	chr1	75796882	75913242	0.046466	0.182728	-1.91706	0.003809
ENSG0000(	NOX4	chr11	89324353	89498187	0.195223	0.027194	2.738916	0.003946
ENSG0000(	VDR	chr12	47841537	47943048	0.078316	0.272584	-1.74644	0.003984
ENSG0000(	SHANK1	chr19	50661827	50719450	0.112873	0.293522	-1.34895	0.004
ENSG0000(	DUSP15	chr20	31847637	31870747	0.066106	0.383477	-2.42933	0.004036
ENSG0000(	PTPN6	chr12	6946468	6961316	0.430262	0.07734	2.387452	0.004056
ENSG0000(	SOHLH1	chr9	1.36E+08	1.36E+08	0.059868	0.30902	-2.27693	0.004083
ENSG0000(	TEX12	chr11	1.12E+08	1.12E+08	0.39417	0.053746	2.741056	0.004303
ENSG0000(	TMEM273	chr10	49154725	49188585	0.123734	0.488078	-1.91805	0.004323
ENSG0000(	MYH7B	chr20	34955810	35002437	0.165241	0.454515	-1.43305	0.004503
ENSG0000(	KCNK12	chr2	47509290	47570985	0.211517	0.097041	1.125546	0.004535
ENSG0000(	ACSS3	chr12	80936414	81261210	0.202223	0.485447	-1.24252	0.004538
ENSG0000(	APOC1	chr19	44914247	44919349	1.146656	2.638144	-1.17544	0.004548
ENSG0000(	ACSM3	chr16	20610243	20797581	0.094405	0.457526	-2.1993	0.004609
ENSG0000(	EOMES	chr3	27715949	27722711	0.00864	0.117075	-3.43087	0.004649
ENSG0000(	KCNS1	chr20	45091214	45101127	0.126907	0.276198	-1.09909	0.004658
ENSG0000(	CXCR4	chr2	1.36E+08	1.36E+08	0.166271	0.5363	-1.66339	0.004675
ENSG0000(	CROCC2	chr2	2.41E+08	2.41E+08	0.138398	0.388166	-1.46131	0.004683
ENSG0000(	CITED4	chr1	40861054	40862363	1.043067	0.51522	1.025119	0.004717
ENSG0000(	GASK1B	chr4	1.58E+08	1.58E+08	0.250945	0.067252	1.872951	0.004789
ENSG0000(	SLC25A41	chr19	6426037	6433779	0.365812	0.819356	-1.13909	0.004939
ENSG0000(	IL17B	chr5	1.49E+08	1.49E+08	0.124883	0.726833	-2.42805	0.004974
ENSG0000(	DPT	chr1	1.69E+08	1.69E+08	0.014627	0.181751	-3.3134	0.005022
ENSG0000(	SOCS1	chr16	11254408	11256204	0.567956	0.222908	1.348202	0.005043
ENSG0000(	TAS2R3	chr7	1.42E+08	1.42E+08	0.252847	0.728824	-1.49506	0.005056
ENSG0000(	FSTL5	chr4	1.61E+08	1.62E+08	0.08489	0	5.198954	0.005056
ENSG0000(	RASGEF1C	chr5	1.8E+08	1.8E+08	0.132636	0	5.19861	0.005058
ENSG0000(	TPRG1	chr3	1.89E+08	1.89E+08	0.356185	0.927085	-1.34962	0.005064
ENSG0000(	ZFP92	chrX	1.53E+08	1.53E+08	0.033125	0.110254	-1.69101	0.005077
ENSG0000(	ANXA1	chr9	73151865	73170393	0.7828	0.306651	1.348239	0.005232
ENSG0000(	SLC25A27	chr6	46652915	46678190	0.542661	1.184943	-1.10232	0.005288
ENSG0000(	B3GALT4	chr6	33277123	33284832	0.809078	0.352114	1.200661	0.005303
ENSG0000(	ECE2	chr3	1.84E+08	1.84E+08	0	0.119165	-5.21894	0.005351
ENSG0000(	TBXA2R	chr19	3594507	3606875	0.015665	0.192941	-3.31104	0.005405
ENSG0000(	TMEM255	chr13	1.14E+08	1.14E+08	0.157342	0	5.199444	0.005464
ENSG0000(	CHRDL1	chrX	1.11E+08	1.11E+08	0.047291	0.203272	-2.03687	0.005521
ENSG0000(	CCDC187	chr9	1.36E+08	1.36E+08	0.056176	0.171734	-1.58077	0.00553

ENSG0000(	SPATA18	chr4	52051304	52097299	0.500585	0.177075	1.490904	0.005601
ENSG0000(	GSTA3	chr6	52896639	52909698	0.944345	0.300161	1.647613	0.005691
ENSG0000(	DRC3	chr17	17972813	18016889	0.363452	0.877071	-1.24459	0.005778
ENSG0000(	UCN	chr2	27307400	27308445	0.319454	0.947517	-1.53441	0.005883
ENSG0000(	TNFSF13	chr17	7558292	7561608	0.237239	0.646958	-1.41805	0.006209
ENSG0000(	H3-5	chr12	31791185	31792298	0.136721	0.511008	-1.85234	0.006276
ENSG0000(	AMDHD1	chr12	95943331	95968720	0.098068	0.401534	-1.96889	0.006278
ENSG0000(	TMEM130	chr7	98846488	98870771	0.123904	0.381587	-1.5807	0.006309
ENSG0000(	HSBP1L1	chr18	79964582	79970822	1.072047	2.20621	-1.01826	0.006432
ENSG0000(	MECOM	chr3	1.69E+08	1.7E+08	0.144917	0.021619	2.641238	0.006488
ENSG0000(	TCHH	chr1	1.52E+08	1.52E+08	0.05108	0.007605	2.64185	0.006514
ENSG0000(	ZC3H12B	chrX	65366638	65507887	0.01707	0.093547	-2.34317	0.006538
ENSG0000(	HAVCR2	chr5	1.57E+08	1.57E+08	0.444717	0.101304	2.081107	0.006595
ENSG0000(	PLIN1	chr15	89664367	89679427	0.06793	0.27685	-1.96817	0.006658
ENSG0000(	C10orf90	chr10	1.26E+08	1.27E+08	0.139782	0	5.200466	0.006691
ENSG0000(	MAGEL2	chr15	23643549	23647867	0	0.053562	-5.21669	0.006691
ENSG0000(	AK8	chr9	1.33E+08	1.33E+08	0.224439	0.615548	-1.41933	0.006754
ENSG0000(	ARHGAP9	chr12	57472264	57488814	0.215283	0.641795	-1.53586	0.006953
ENSG0000(	SCGB2B2	chr19	34593329	34675699	0.106771	0.434378	-1.96756	0.007029
ENSG0000(	SOX5	chr12	23529504	24562544	0.263322	0.051001	2.29997	0.007131
ENSG0000(	AHRR	chr5	321759	435110	0.229651	0.019894	3.274079	0.007159
ENSG0000(	MVP	chr16	29820394	29848039	1.048772	2.135321	-1.01263	0.007208
ENSG0000(	TMEM31	chrX	1.04E+08	1.04E+08	0.277262	0.885956	-1.63515	0.007252
ENSG0000(	SKAP2	chr7	26667068	26995239	0.852961	1.824408	-1.07838	0.007685
ENSG0000(	RAB40A	chrX	1.03E+08	1.04E+08	0.169173	0.396432	-1.20723	0.007827
ENSG0000(	FAM167B	chr1	32247222	32248856	0.34936	0.932008	-1.39094	0.007858
ENSG0000(	MYMX	chr6	44216926	44218234	0.269295	0.857748	-1.63448	0.007921
ENSG0000(	PIK3CD	chr1	9651731	9729114	0.089802	0.251783	-1.4498	0.007942
ENSG0000(	ORAI3	chr16	30949068	30956461	0.292341	0.784081	-1.38996	0.00798
ENSG0000(	NELL2	chr12	44508275	44921848	0.15798	0.454009	-1.48469	0.008064
ENSG0000(	ZDHHC11	chr5	795606	850986	0.313746	0.770696	-1.27044	0.008141
ENSG0000(	CEBPA	chr19	33299934	33302534	0.07803	0.250395	-1.63637	0.00816
ENSG0000(	CENPS	chr1	10430433	10442808	1.193856	0.455398	1.376496	0.008205
ENSG0000(	GRM1	chr6	1.46E+08	1.46E+08	0.01127	0.080831	-2.68129	0.008289
ENSG0000(	CCIN	chr9	36169388	36171334	0.039113	0.214744	-2.34525	0.008323
ENSG0000(	SOX7	chr8	10723768	10730511	0.18882	0.395216	-1.04401	0.008339
ENSG0000(	SLC24A5	chr15	48120990	48142672	0.099691	0.336202	-1.70331	0.008352
ENSG0000(	SP7	chr12	53326575	53345315	0.129361	0.360175	-1.44739	0.008372
ENSG0000(	PIPOX	chr17	28950513	29057216	0.633691	1.29931	-1.01567	0.008458
ENSG0000(	LDB3	chr10	86668507	86736072	0.083716	0.266843	-1.63392	0.008511
ENSG0000(	C19orf38	chr19	10836575	10869790	0.025709	0.291504	-3.18943	0.008599
ENSG0000(	TMEM179	chr14	1.04E+08	1.05E+08	0.016031	0.179768	-3.18977	0.008627
ENSG0000(	OCA2	chr15	27754875	28099315	0.012823	0.143796	-3.18977	0.008631
ENSG0000(	SLPI	chr20	45252239	45254564	0.042186	0.479884	-3.19014	0.008636
ENSG0000(	RASL11A	chr13	27270830	27275192	0.657457	0.244444	1.406738	0.008733
ENSG0000(	INHBC	chr12	57434784	57452062	0.174221	0.37293	-1.07739	0.008738
ENSG0000(	ADRA1A	chr8	26748150	26867278	0.145484	0.013309	3.150926	0.008757
ENSG0000(	ASPN	chr9	92456205	92482506	0.22381	0.58234	-1.34964	0.008794
ENSG0000(	FCMR	chr1	2.07E+08	2.07E+08	0.600496	0.199651	1.590347	0.008937
ENSG0000(	TF	chr3	1.34E+08	1.34E+08	0.01074	0.122567	-3.19107	0.009121
ENSG0000(	IL20RB	chr3	1.37E+08	1.37E+08	0.724323	0.335136	1.118645	0.009253
ENSG0000(	KIAA0319	chr6	24544104	24646191	0.072724	0.166102	-1.16374	0.009385
ENSG0000(	BICDL1	chr12	1.2E+08	1.2E+08	0.776022	0.246825	1.636453	0.009514
ENSG0000(	TTC9	chr14	70641916	70675366	0.044778	0.132136	-1.52574	0.009538
ENSG0000(	IL17D	chr13	20702127	20723098	1.051265	0.481731	1.121269	0.009664
ENSG0000(	SMPDL3B	chr1	27935000	27959157	0.159095	0.470653	-1.52661	0.009687
ENSG0000(	MARCOL	chr5	1.48E+08	1.48E+08	0.151189	0	5.033759	0.009767
ENSG0000(	GPR39	chr2	1.32E+08	1.33E+08	0	0.11298	-5.05432	0.009883

ENSG0000(	KCNJ14	chr19	48455574	48466980	0.238953	0.106605	1.174086	0.009897
ENSG0000(	PCSK1	chr5	96390333	96434143	0.39812	0.177615	1.174084	0.009907
ENSG0000(	CILP2	chr19	19538248	19546659	0.211065	0.431859	-1.01411	0.010053
ENSG0000(	CD177	chr19	43353686	43363172	0.143533	0	5.034196	0.010097
ENSG0000(	ACKR1	chr1	1.59E+08	1.59E+08	0.646156	0.284145	1.186928	0.010201
ENSG0000(	CFAP46	chr10	1.33E+08	1.33E+08	0.129396	0.38192	-1.52545	0.010204
ENSG0000(	ADGRG1	chr16	57610652	57665580	0.505757	1.169665	-1.17976	0.010313
ENSG0000(	C6orf132	chr6	42101119	42142620	0.138746	0.30704	-1.1177	0.01033
ENSG0000(	APOBR	chr16	28494643	28498970	0.013403	0.095197	-2.67858	0.010472
ENSG0000(	H3C7	chr6	26250142	26250635	0.461738	1.408583	-1.57695	0.010577
ENSG0000(	BHLHA15	chr7	98211427	98212979	0.438126	0.070357	2.536428	0.010716
ENSG0000(	MBL2	chr10	52765380	52772784	0.021338	0.109232	-2.25153	0.010729
ENSG0000(	NOS1	chr12	1.17E+08	1.17E+08	0.0134	0.068599	-2.25153	0.010735
ENSG0000(	LRRC55	chr11	57181747	57191717	0.052671	0.004979	3.15093	0.010791
ENSG0000(	BDKRB1	chr14	96256210	96268967	0.250198	0.023652	3.15093	0.0108
ENSG0000(	ASB9	chrX	15235288	15270467	0.331916	0.068417	2.209086	0.010803
ENSG0000(	TMEM144	chr4	1.58E+08	1.58E+08	0.236284	0.037324	2.536898	0.010824
ENSG0000(	IPO4	chr14	24180219	24188869	0.245755	0.0592	2.000179	0.010847
ENSG0000(	GNMT	chr6	42960754	42963880	0.633606	0.216703	1.535844	0.010932
ENSG0000(	CDHR2	chr5	1.77E+08	1.77E+08	0.058895	0.230155	-1.89722	0.011074
ENSG0000(	ESRP2	chr16	68229033	68238102	0.034325	0.229413	-2.57798	0.011086
ENSG0000(	ATP8B4	chr15	49858238	50182817	0.150974	0.024271	2.535958	0.011212
ENSG0000(	ANKRD1	chr10	90912096	90921087	0.623303	0.303778	1.037205	0.01124
ENSG0000(	EZHIP	chrX	51406948	51408843	0.173923	0.026898	2.538833	0.011307
ENSG0000(	NPTX1	chr17	80466834	80477843	0.233501	0.080043	1.533809	0.011448
ENSG0000(	CFD	chr19	859664	863641	0.27766	0.750517	-1.39986	0.01159
ENSG0000(	WSCD2	chr12	1.08E+08	1.08E+08	0.472026	0.162487	1.536276	0.011682
ENSG0000(	ESRRB	chr14	76310712	76501837	0	0.087833	-5.05074	0.011946
ENSG0000(	BLK	chr8	11486894	11564599	0.130953	0	5.031027	0.011946
ENSG0000(	ST14	chr11	1.3E+08	1.3E+08	0.128743	0	5.032398	0.011946
ENSG0000(	STPG2	chr4	97184093	98143476	0	0.094875	-5.05976	0.011946
ENSG0000(	NEXN	chr1	77888513	77943895	0.391409	0.160016	1.2827	0.011965
ENSG0000(	GRIA2	chr4	1.57E+08	1.57E+08	0.034334	0.174521	-2.24947	0.011999
ENSG0000(	IL21R	chr16	27402174	27452042	0.212043	0.456574	-1.08319	0.01212
ENSG0000(	ATP13A4	chr3	1.93E+08	1.94E+08	0.03086	0.159482	-2.25348	0.012331
ENSG0000(	PRR5L	chr11	36296288	36465204	0.234148	0.646851	-1.43169	0.012359
ENSG0000(	GJC3	chr7	99923266	99929620	0.227282	0.646272	-1.4812	0.012665
ENSG0000(	KRT17	chr17	41619442	41624842	0.185221	0.626249	-1.70561	0.013469
ENSG0000(	UNC13D	chr17	75827225	75844552	0.382911	0.823949	-1.08545	0.013638
ENSG0000(	EDN2	chr1	41478775	41484683	0.83599	0.276296	1.594487	0.013913
ENSG0000(	ZCWPW2	chr3	28348721	28538122	0.276458	0.683174	-1.27372	0.013914
ENSG0000(	RPGRIP1	chr14	21287939	21351301	0.065922	0.257358	-1.89809	0.013985
ENSG0000(	GGN	chr19	38384267	38388082	0.401654	0.095737	2.002246	0.014389
ENSG0000(	PSD4	chr2	1.13E+08	1.13E+08	0.099447	0.243629	-1.26003	0.014409
ENSG0000(	SPON2	chr4	1166932	1208962	1.028705	0.431105	1.259671	0.014683
ENSG0000(	HS3ST3B1	chr17	14301081	14349404	0.124042	0.038316	1.674392	0.014738
ENSG0000(	ARHGAP2	chr17	45393902	45434421	0.2894	0.604748	-1.04608	0.014868
ENSG0000(	CHSY3	chr5	1.3E+08	1.3E+08	0.281675	0.104998	1.425804	0.015003
ENSG0000(	RAB19	chr7	1.4E+08	1.4E+08	0.014433	0.149019	-3.05547	0.015191
ENSG0000(	SPINT1	chr15	40844018	40858207	0.364688	0.811934	-1.13181	0.015197
ENSG0000(	DENND2C	chr1	1.15E+08	1.15E+08	0.006708	0.069261	-3.05547	0.01521
ENSG0000(	CCDC190	chr1	1.63E+08	1.63E+08	0.199469	0.521507	-1.35022	0.015266
ENSG0000(	IRAK3	chr12	66189195	66254622	0.051682	0.152429	-1.51661	0.015428
ENSG0000(	C12orf40	chr12	39626167	39908300	0.01021	0.103552	-3.05427	0.015493
ENSG0000(	EMID1	chr22	29205896	29259597	0.124842	0.423299	-1.7156	0.015624
ENSG0000(	IL7R	chr5	35852695	35879603	0.450844	0.139763	1.673042	0.015905
ENSG0000(	CACNG5	chr17	66835117	66894751	0.07378	0.20903	-1.47076	0.016051
ENSG0000(	PCDHA1	chr5	1.41E+08	1.41E+08	0.2792	0.134984	1.046317	0.016174

ENSG0000(	C3orf80	chr3	1.6E+08	1.6E+08	0.211696	0.054251	1.913667	0.016316
ENSG0000(	HYAL1	chr3	50299890	50312381	0.202352	0.502184	-1.27774	0.016865
ENSG0000(	CLCNKB	chr1	16043736	16057308	0.015011	0.153882	-3.05354	0.016881
ENSG0000(	KIF6	chr6	39329990	39725408	0.074677	0.199016	-1.37861	0.016881
ENSG0000(	TSPAN19	chr12	85014311	85036277	0.398057	0.103005	1.912798	0.016886
ENSG0000(	NME5	chr5	1.38E+08	1.38E+08	0.840996	1.719504	-1.01285	0.017168
ENSG0000(	RAI2	chrX	17800049	17861337	0.025749	0.158368	-2.46546	0.017248
ENSG0000(	ELL3	chr15	43772605	43777315	0.053653	0.329989	-2.46546	0.017256
ENSG0000(	GRAP	chr17	19020656	19047011	0.050097	0.307211	-2.46478	0.017507
ENSG0000(	GASK1A	chr3	42979267	43060211	0.503037	0.250558	1.006264	0.017734
ENSG0000(	FSTL4	chr5	1.33E+08	1.34E+08	0.095209	0.29284	-1.57293	0.017757
ENSG0000(	SLC19A3	chr2	2.28E+08	2.28E+08	0.048515	0.20036	-1.95688	0.017817
ENSG0000(	CALCB	chr11	14904997	15082342	0.253845	0.072364	1.776632	0.017887
ENSG0000(	PLEK	chr2	68365282	68397453	0.028719	0.177379	-2.46512	0.018011
ENSG0000(	PLP1	chrX	1.04E+08	1.04E+08	0.054246	0.337033	-2.46663	0.01811
ENSG0000(	TAS2R5	chr7	1.42E+08	1.42E+08	0.286182	0.726508	-1.30846	0.018302
ENSG0000(	SVOPL	chr7	1.39E+08	1.39E+08	0.21246	0.478418	-1.13999	0.018442
ENSG0000(	TULP2	chr19	48880967	48898744	0.097011	0.460623	-2.15201	0.018447
ENSG0000(	GATM	chr15	45361124	45402327	0.177506	0.437849	-1.27623	0.018711
ENSG0000(	COL2A1	chr12	47972967	48004554	0.039806	0.191842	-2.15515	0.018735
ENSG0000(	CSF3R	chr1	36466043	36483278	0.038808	0.187029	-2.15515	0.018743
ENSG0000(	FAM240C	chr2	2.42E+08	2.42E+08	0	0.336114	-4.86921	0.01887
ENSG0000(	POTEF	chr2	1.3E+08	1.3E+08	0	0.061504	-4.86921	0.018878
ENSG0000(	GALNT12	chr9	98807670	98850081	0	0.153605	-4.86826	0.018899
ENSG0000(	WNT5A	chr3	55465715	55490539	0	0.078567	-4.86826	0.018907
ENSG0000(	PROK2	chr3	71771655	71785206	0.114336	0	4.84762	0.019081
ENSG0000(	PPP1R36	chr14	64549920	64589381	0.361257	0.813771	-1.1461	0.019143
ENSG0000(	C10orf82	chr10	1.17E+08	1.17E+08	0.038644	0.237386	-2.46683	0.019646
ENSG0000(	SLC22A15	chr1	1.16E+08	1.16E+08	0.023814	0.146288	-2.46683	0.019653
ENSG0000(	ANO3	chr11	26188842	26663289	0.269641	0.121449	1.155481	0.01973
ENSG0000(	TM4SF1	chr3	1.49E+08	1.49E+08	0.302389	0.637069	-1.04983	0.019773
ENSG0000(	P2RX3	chr11	57338352	57372396	0.015832	0.16342	-3.06064	0.019981
ENSG0000(	LIMS4	chr2	1.1E+08	1.1E+08	0	0.155428	-4.87075	0.020524
ENSG0000(	APCDD1	chr18	10454635	10489949	0	0.123628	-4.87076	0.020543
ENSG0000(	NPFFR1	chr10	70247329	70284004	0	0.019498	-4.86674	0.020621
ENSG0000(	SGCD	chr5	1.56E+08	1.57E+08	0	0.053385	-4.86673	0.020642
ENSG0000(	ATAD3C	chr1	1449689	1470163	0.185541	0.462693	-1.27927	0.021037
ENSG0000(	AQP5	chr12	49961872	49965682	0.600569	0.251549	1.254013	0.02108
ENSG0000(	PRR35	chr16	560394	565529	0.07872	0	4.84641	0.021092
ENSG0000(	LDLRAD2	chr1	21812265	21825225	0.185309	0.417777	-1.14604	0.0212
ENSG0000(	DLL4	chr15	40929340	40939073	0	0.089938	-4.87264	0.021587
ENSG0000(	AC010325	chr19	50776141	50793142	0.462791	0	4.849162	0.021587
ENSG0000(	MAP3K8	chr10	30434021	30461833	0.568799	0.284824	1.006928	0.021598
ENSG0000(	UGT3A2	chr5	36035021	36071358	0.110828	0.392593	-1.77885	0.02191
ENSG0000(	SHROOM:	chrX	9786429	9949443	0.046446	0.158486	-1.711	0.022221
ENSG0000(	NCAN	chr19	19211958	19252233	0.195247	0.063084	1.602061	0.022297
ENSG0000(	TXLNB	chr6	1.39E+08	1.39E+08	0.020219	0.125457	-2.46906	0.02245
ENSG0000(	HTR4	chr5	1.48E+08	1.49E+08	0.516435	0.259109	1.006548	0.022878
ENSG0000(	TBC1D8B	chrX	1.07E+08	1.07E+08	0.019931	0.122375	-2.46257	0.022967
ENSG0000(	MSX1	chr4	4859665	4863936	0.557426	0.231226	1.25531	0.023085
ENSG0000(	RAG2	chr11	36575574	36598279	0.350012	0.793295	-1.1546	0.023617
ENSG0000(	ZBED6	chr1	2.04E+08	2.04E+08	0.026305	0.062618	-1.21453	0.024008
ENSG0000(	TAF7L	chrX	1.01E+08	1.01E+08	0.326838	0.129672	1.332879	0.024091

padj	level
0	up
9.3657060	down
#######	down
#######	up
#######	up
#######	down
#######	down
#######	up
#######	down
#######	up
#######	down
#######	down
#######	down
#######	up
#######	up
#######	down
#######	down
#######	up
#######	down
#######	up
#######	down
#######	up
#######	up
#######	down
#######	down
#######	up
#######	down
#######	down
#######	down
#######	down
#######	up
#######	down
#######	down
#######	up
#######	down
#######	up
#######	up
#######	up
#######	up
#######	down
#######	up
#######	up
#######	down
#######	up
#######	down
#######	up
2.13E-98	up
3.78E-98	down
6.12E-97	up
1.23E-95	down
1.30E-92	down
3.89E-90	down
1.19E-89	down
9.06E-89	down
1.31E-88	up
2.90E-87	up
3.10E-87	up

1.69E-84 down
3.42E-84 up
2.23E-83 down
3.58E-83 up
1.49E-82 down
2.62E-81 down
2.66E-81 up
2.71E-81 down
6.58E-81 up
3.04E-80 up
1.43E-79 down
1.24E-77 down
2.16E-77 down
5.17E-77 down
9.74E-77 down
9.93E-77 up
2.04E-74 down
3.68E-74 down
6.20E-74 down
1.13E-73 up
2.12E-73 down
2.38E-72 down
9.11E-72 up
9.21E-72 up
7.12E-71 down
1.11E-70 up
2.69E-70 down
9.06E-67 up
5.07E-66 down
4.96E-65 down
2.11E-64 up
1.30E-63 down
4.57E-63 down
6.98E-62 up
1.38E-61 up
1.14E-60 down
1.21E-60 up
1.62E-59 down
5.23E-59 down
9.15E-59 down
3.74E-57 up
4.52E-57 down
1.35E-56 down
1.87E-56 down
1.91E-56 up
2.34E-56 up
9.26E-56 down
1.09E-55 down
1.81E-55 down
7.39E-55 down
8.60E-55 down
1.05E-54 up
1.17E-54 up
8.64E-54 down
2.28E-53 up
2.87E-53 up
8.62E-52 down
2.04E-51 up

7.17E-51 down
6.53E-50 up
1.48E-49 up
3.58E-49 up
3.71E-49 down
5.05E-49 down
1.23E-48 up
1.86E-48 down
2.73E-48 down
2.77E-48 up
3.59E-47 down
5.57E-47 down
2.02E-46 up
2.36E-46 up
2.96E-46 up
4.09E-46 up
6.48E-46 down
1.08E-45 up
1.11E-45 up
1.41E-45 up
2.40E-45 down
3.38E-45 down
1.20E-44 up
1.48E-44 up
1.76E-44 up
1.83E-44 up
2.71E-44 down
3.24E-44 up
3.44E-44 down
6.81E-44 down
1.80E-43 up
1.32E-42 up
1.40E-42 up
1.51E-42 down
3.12E-42 down
1.87E-41 down
2.12E-41 down
3.82E-41 down
5.66E-41 down
2.28E-40 up
2.34E-40 up
7.19E-40 down
7.20E-40 up
8.79E-40 down
3.04E-39 down
4.29E-39 up
4.40E-39 up
4.50E-39 up
8.48E-39 down
9.40E-39 down
1.12E-38 down
2.02E-38 up
3.88E-38 down
6.19E-38 up
1.31E-37 up
7.75E-37 up
1.00E-36 up
1.04E-36 down

2.14E-36 up
2.21E-36 up
6.96E-36 up
2.51E-35 up
6.74E-35 down
1.45E-34 down
1.74E-34 up
5.97E-33 down
6.79E-33 down
7.73E-33 down
7.82E-33 up
9.32E-33 up
1.73E-32 up
2.09E-32 down
2.21E-32 up
4.42E-31 down
8.40E-31 down
1.59E-30 up
2.19E-30 down
4.34E-30 down
7.20E-30 down
7.55E-30 down
1.48E-29 down
3.39E-29 down
3.61E-29 up
3.98E-29 up
5.29E-29 up
6.65E-29 up
6.94E-29 up
9.37E-29 down
2.11E-28 up
2.19E-28 down
2.24E-28 up
3.83E-28 down
1.04E-27 down
1.08E-27 down
1.21E-27 up
2.38E-27 up
4.46E-27 down
4.54E-27 down
5.62E-27 down
7.37E-27 up
9.64E-27 up
1.30E-26 down
2.83E-26 up
4.52E-26 down
5.23E-26 down
8.02E-26 down
9.03E-26 down
9.04E-26 down
1.33E-25 down
2.03E-25 down
2.03E-25 up
2.51E-25 down
5.12E-25 up
7.76E-25 down
9.75E-25 up
1.26E-24 down

1.36E-24 up
1.61E-24 up
1.87E-24 up
4.98E-24 down
6.05E-24 up
7.22E-24 down
8.07E-24 up
8.44E-24 up
9.38E-24 up
1.42E-23 down
3.97E-23 down
4.50E-23 up
4.76E-23 down
5.87E-23 up
7.14E-23 down
7.20E-23 up
1.20E-22 down
1.26E-22 down
2.27E-22 down
2.56E-22 up
2.62E-22 down
3.79E-22 up
3.80E-22 down
5.09E-22 down
8.88E-22 up
1.04E-21 up
1.34E-21 up
1.42E-21 down
1.59E-21 up
1.96E-21 down
2.13E-21 down
2.66E-21 down
2.85E-21 up
3.37E-21 up
6.04E-21 down
6.43E-21 up
7.24E-21 up
8.33E-21 down
8.65E-21 down
1.29E-20 down
3.17E-20 down
3.54E-20 up
3.65E-20 down
4.22E-20 up
4.29E-20 down
4.50E-20 down
4.88E-20 down
5.73E-20 down
6.62E-20 up
6.63E-20 down
1.75E-19 down
1.81E-19 up
1.83E-19 down
2.00E-19 down
2.44E-19 down
2.64E-19 down
4.12E-19 down
4.22E-19 up

5.01E-19 down
5.02E-19 down
5.39E-19 down
5.43E-19 down
7.81E-19 down
1.14E-18 down
1.25E-18 up
1.30E-18 up
1.68E-18 down
1.71E-18 down
1.96E-18 down
2.06E-18 up
2.15E-18 up
2.17E-18 down
2.45E-18 down
2.75E-18 down
3.16E-18 up
3.50E-18 up
4.52E-18 up
4.57E-18 up
4.57E-18 down
4.63E-18 up
8.96E-18 down
9.08E-18 down
1.05E-17 down
1.20E-17 up
1.24E-17 down
1.27E-17 up
1.44E-17 down
1.54E-17 up
1.57E-17 down
1.87E-17 down
2.50E-17 down
2.71E-17 down
3.86E-17 down
6.58E-17 down
6.94E-17 down
1.06E-16 down
1.22E-16 up
1.34E-16 down
1.41E-16 down
2.23E-16 up
2.95E-16 down
3.20E-16 down
3.22E-16 down
3.33E-16 down
3.62E-16 down
4.75E-16 down
4.95E-16 down
6.81E-16 up
7.61E-16 down
1.12E-15 up
1.16E-15 down
2.00E-15 down
2.02E-15 down
2.19E-15 up
3.58E-15 up
4.18E-15 down

4.35E-15 down
5.38E-15 down
5.53E-15 down
9.79E-15 down
1.02E-14 up
1.02E-14 up
1.16E-14 up
1.16E-14 down
1.21E-14 up
1.34E-14 up
1.42E-14 up
1.45E-14 down
1.57E-14 down
1.57E-14 up
2.08E-14 down
2.21E-14 down
2.42E-14 down
2.70E-14 down
4.13E-14 down
4.21E-14 up
4.21E-14 down
4.37E-14 down
4.94E-14 down
5.16E-14 down
6.37E-14 down
6.62E-14 up
9.13E-14 up
1.08E-13 down
1.15E-13 up
1.42E-13 up
1.57E-13 up
1.79E-13 down
1.81E-13 down
1.95E-13 down
2.12E-13 down
2.59E-13 up
4.01E-13 down
4.32E-13 up
4.75E-13 up
5.05E-13 down
5.73E-13 down
5.80E-13 down
6.34E-13 up
6.69E-13 down
7.01E-13 down
8.19E-13 up
9.72E-13 down
9.86E-13 up
1.05E-12 down
1.16E-12 down
1.20E-12 up
1.22E-12 down
1.25E-12 down
1.48E-12 down
1.53E-12 down
1.56E-12 down
1.80E-12 up
1.90E-12 down

2.09E-12 down
2.20E-12 down
2.34E-12 down
2.80E-12 down
2.90E-12 down
3.04E-12 down
3.41E-12 up
3.42E-12 down
3.64E-12 up
3.69E-12 down
6.00E-12 down
7.32E-12 up
7.81E-12 down
8.72E-12 up
9.03E-12 up
1.06E-11 down
1.12E-11 up
1.40E-11 up
1.42E-11 up
2.32E-11 up
2.53E-11 up
2.60E-11 down
3.09E-11 down
3.99E-11 down
4.60E-11 up
4.87E-11 down
5.68E-11 down
6.21E-11 down
7.50E-11 up
8.37E-11 up
8.52E-11 up
8.82E-11 up
1.16E-10 up
1.23E-10 up
1.40E-10 up
1.43E-10 up
1.66E-10 up
1.69E-10 up
1.93E-10 down
1.95E-10 down
2.42E-10 down
2.48E-10 down
2.62E-10 down
3.12E-10 up
3.32E-10 up
3.34E-10 up
3.67E-10 up
4.21E-10 down
4.86E-10 down
5.67E-10 up
5.77E-10 up
7.33E-10 down
7.70E-10 up
7.88E-10 down
8.87E-10 down
9.69E-10 down
1.11E-09 down
1.14E-09 up

1.15E-09 down
1.34E-09 down
1.59E-09 down
1.61E-09 down
1.69E-09 up
1.71E-09 down
1.83E-09 down
1.99E-09 down
2.13E-09 down
2.19E-09 down
2.26E-09 up
2.37E-09 up
2.44E-09 down
2.47E-09 up
2.53E-09 up
3.37E-09 up
3.83E-09 down
3.98E-09 up
5.04E-09 up
5.12E-09 up
7.47E-09 down
8.20E-09 down
9.39E-09 down
1.03E-08 up
1.05E-08 up
1.11E-08 up
1.32E-08 up
1.32E-08 down
1.39E-08 down
1.67E-08 down
1.67E-08 down
1.94E-08 down
1.94E-08 down
2.17E-08 down
2.28E-08 down
2.61E-08 down
2.72E-08 up
2.81E-08 up
2.88E-08 down
2.91E-08 up
2.94E-08 down
3.02E-08 down
3.20E-08 down
3.39E-08 down
3.56E-08 up
3.72E-08 up
3.76E-08 down
3.79E-08 down
4.19E-08 down
4.34E-08 down
4.55E-08 down
4.58E-08 up
5.18E-08 down
5.45E-08 down
5.73E-08 down
6.05E-08 up
6.64E-08 up
7.04E-08 down

7.44E-08 up
1.03E-07 down
1.03E-07 down
1.04E-07 down
1.09E-07 down
1.16E-07 down
1.18E-07 down
1.24E-07 down
1.26E-07 down
1.47E-07 down
1.61E-07 down
1.62E-07 down
1.64E-07 up
1.67E-07 up
1.78E-07 down
1.84E-07 down
2.03E-07 up
2.13E-07 down
2.20E-07 down
2.20E-07 down
2.41E-07 down
2.61E-07 down
2.64E-07 down
2.68E-07 down
2.94E-07 down
2.96E-07 up
2.97E-07 up
3.03E-07 down
3.08E-07 down
3.10E-07 up
3.62E-07 up
3.81E-07 down
3.85E-07 up
4.44E-07 down
4.48E-07 down
4.55E-07 up
5.30E-07 down
5.36E-07 down
5.39E-07 up
5.69E-07 up
5.94E-07 down
6.06E-07 up
6.48E-07 down
6.52E-07 down
6.59E-07 down
8.29E-07 down
1.15E-06 up
1.15E-06 down
1.16E-06 down
1.35E-06 down
1.38E-06 down
1.41E-06 up
1.45E-06 up
1.46E-06 down
1.55E-06 down
1.64E-06 down
1.65E-06 down
1.73E-06 down

1.78E-06 down
1.80E-06 down
1.90E-06 down
1.94E-06 up
2.06E-06 down
2.11E-06 up
2.11E-06 down
2.45E-06 up
2.75E-06 down
2.79E-06 up
3.18E-06 down
3.29E-06 up
3.48E-06 down
3.52E-06 up
3.57E-06 down
3.63E-06 up
3.84E-06 down
3.84E-06 down
3.87E-06 down
4.57E-06 down
4.68E-06 down
4.93E-06 up
4.97E-06 up
5.00E-06 up
5.80E-06 down
5.83E-06 down
5.92E-06 down
6.36E-06 up
6.38E-06 down
6.47E-06 down
6.55E-06 down
6.61E-06 down
6.93E-06 down
6.97E-06 up
7.03E-06 up
7.45E-06 up
7.71E-06 down
7.75E-06 down
7.88E-06 down
8.87E-06 up
9.19E-06 down
9.25E-06 down
9.32E-06 up
9.46E-06 down
9.85E-06 down
1.02E-05 down
1.04E-05 down
1.04E-05 up
1.08E-05 down
1.10E-05 down
1.14E-05 down
1.19E-05 up
1.19E-05 down
1.30E-05 down
1.32E-05 up
1.41E-05 up
1.42E-05 down
1.42E-05 down

1.56E-05 down
1.57E-05 down
1.59E-05 up
1.63E-05 down
1.70E-05 down
1.78E-05 down
1.79E-05 down
1.92E-05 down
1.96E-05 down
1.96E-05 down
1.98E-05 down
1.98E-05 down
2.04E-05 down
2.09E-05 down
2.13E-05 down
2.18E-05 down
2.21E-05 up
2.36E-05 down
2.37E-05 down
2.37E-05 down
2.61E-05 up
2.63E-05 down
2.68E-05 down
2.71E-05 up
2.78E-05 down
2.84E-05 up
2.84E-05 down
2.87E-05 down
2.89E-05 down
2.96E-05 down
3.80E-05 down
3.97E-05 up
4.17E-05 down
4.42E-05 down
4.70E-05 up
4.75E-05 down
4.96E-05 up
5.23E-05 up
5.27E-05 down
5.38E-05 down
5.47E-05 down
5.69E-05 down
5.71E-05 up
5.97E-05 up
5.99E-05 down
6.01E-05 down
6.41E-05 down
6.53E-05 down
6.54E-05 down
6.65E-05 down
6.74E-05 down
7.32E-05 down
7.63E-05 up
8.26E-05 down
8.90E-05 down
9.09E-05 down
9.12E-05 down
9.14E-05 up

9.42E-05	down
9.62E-05	down
9.76E-05	up
0.000101	down
0.000104	down
0.000114	down
0.000115	down
0.000115	down
0.000117	down
0.000119	up
0.000124	down
0.000125	down
0.000128	up
0.00013	down
0.00013	up
0.000131	down
0.000136	up
0.000139	down
0.000141	down
0.000144	down
0.00015	down
0.000152	down
0.000153	down
0.000154	up
0.000156	up
0.00016	up
0.000162	down
0.000165	down
0.000175	down
0.000176	down
0.000178	down
0.000182	down
0.000183	down
0.000184	up
0.000188	up
0.000189	down
0.0002	down
0.000202	up
0.000206	down
0.000212	down
0.00022	up
0.000231	up
0.000238	down
0.000239	up
0.000243	down
0.000256	up
0.000266	up
0.000284	up
0.00029	down
0.000294	down
0.000303	down
0.000303	down
0.00031	down
0.000315	up
0.000319	up
0.000346	up
0.000368	down
0.000368	up

0.000376 up
0.0004 down
0.000423 up
0.000426 down
0.00044 up
0.000449 down
0.000453 down
0.000479 up
0.000482 down
0.000483 down
0.000509 down
0.000546 down
0.000556 down
0.000559 up
0.00056 down
0.000564 up
0.000564 up
0.000578 down
0.000598 up
0.000638 up
0.000638 down
0.000668 down
0.000671 down
0.000674 down
0.000688 down
0.000688 up
0.000733 up
0.00076 up
0.000805 down
0.000806 down
0.000809 up
0.000836 up
0.000877 up
0.000897 down
0.000948 down
0.000997 down
0.00102 up
0.001034 up
0.001037 down
0.001082 down
0.001094 down
0.001098 down
0.001152 up
0.001196 down
0.001211 down
0.001237 down
0.001239 down
0.00128 up
0.001286 down
0.001292 up
0.001295 down
0.001311 up
0.001343 down
0.001362 up
0.0014 down
0.001413 up
0.001413 up
0.001413 down

0.001428 up
0.001432 down
0.001447 down
0.001467 down
0.001488 down
0.00149 down
0.001498 up
0.001519 down
0.001543 down
0.001543 down
0.001564 down
0.001576 down
0.001588 down
0.001599 down
0.00162 up
0.001638 down
0.001676 down
0.001767 down
0.001774 up
0.001787 down
0.001836 down
0.001852 down
0.001865 down
0.001884 down
0.001889 down
0.001899 down
0.001905 down
0.001905 down
0.001905 up
0.001957 up
0.001968 up
0.002034 up
0.002091 down
0.002095 down
0.002122 down
0.002166 down
0.002171 down
0.002178 down
0.002181 down
0.00221 down
0.002227 down
0.002266 down
0.002306 down
0.002342 down
0.002364 down
0.00242 down
0.002441 down
0.002462 down
0.002466 down
0.002478 down
0.002558 down
0.002572 up
0.002739 down
0.00275 down
0.002751 down
0.002842 down
0.002842 down
0.00296 up

0.002978 down
0.003052 down
0.003082 down
0.00314 down
0.003182 down
0.003265 down
0.003341 down
0.003346 down
0.003461 down
0.003497 down
0.003513 up
0.003652 up
0.003699 down
0.003738 down
0.003918 up
0.004071 down
0.004199 down
0.004207 up
0.004209 up
0.004222 down
0.004271 down
0.004334 down
0.004338 up
0.004352 up
0.004545 up
0.004577 up
0.004746 down
0.004779 down
0.004805 down
0.00483 up
0.005084 down
0.005086 down
0.005126 up
0.005252 down
0.005253 up
0.005545 up
0.005626 down
0.005652 down
0.005728 down
0.005815 down
0.005926 up
0.005956 up
0.005959 up
0.006046 down
0.006411 down
0.006743 down
0.00681 down
0.006933 down
0.006944 down
0.00699 down
0.007009 down
0.007067 down
0.007163 down
0.007167 down
0.007167 up
0.007168 down
0.007176 down
0.007368 down

4.99E-13	up
0.007607	down
0.007639	down
0.007686	up
0.007751	down
0.007808	down
0.007826	down
0.007887	down
0.007921	down
0.008701	down
0.008708	up
0.008808	up
0.008828	up
0.00885	down
0.009104	down
0.009115	down
0.0092	down
0.0092	down
0.009334	up
0.009382	down
0.009389	down
0.009428	down
0.009742	up
0.009831	down
0.009863	down
0.009948	down
0.009992	up
0.010052	down
0.010541	up
0.01058	down
0.010984	down
0.011049	up
0.011053	down
0.011071	down
0.011203	down
0.011296	down
0.011315	down
0.011353	down
0.011369	down
0.011442	up
0.011596	up
0.011928	down
0.012005	down
0.012108	down
0.012152	up
0.012177	down
0.012177	up
0.01218	up
0.012188	down
0.012215	down
0.012556	up
0.012684	down
0.012714	up
0.012815	down
0.012935	down
0.013059	up
0.013187	down
0.013206	down

0.013366	up
0.013555	up
0.013741	down
0.013976	down
0.014683	down
0.014829	down
0.014832	down
0.014897	down
0.015152	down
0.015274	up
0.015327	up
0.01538	down
0.015502	up
0.015643	down
0.015712	up
0.015712	down
0.015849	down
0.016275	down
0.016439	down
0.016665	up
0.016724	up
0.016825	down
0.01691	down
0.017844	down
0.01814	down
0.018202	down
0.018333	down
0.01837	down
0.018446	down
0.018632	down
0.018795	down
0.018828	down
0.018926	up
0.0191	down
0.019169	down
0.019204	down
0.019226	down
0.019261	down
0.019442	down
0.019544	down
0.019724	down
0.01978	down
0.019785	down
0.019795	down
0.019992	up
0.019999	down
0.020035	up
0.020106	down
0.020416	up
0.020792	down
0.02107	up
0.021356	down
0.021615	up
0.021661	down
0.021921	up
0.021971	down
0.022136	up
0.022379	down

0.022409 up
0.022424 up
0.022738 down
0.022836 up
0.023054 up
0.023057 down
0.023276 down
0.023309 down
0.023596 down
0.023812 down
0.024089 up
0.024116 down
0.024124 down
0.024231 up
0.024244 up
0.024248 up
0.024288 up
0.024333 up
0.024503 up
0.024803 down
0.024826 down
0.025069 up
0.025125 up
0.02527 up
0.025564 up
0.025849 down
0.026039 up
0.026541 down
0.026541 up
0.026541 up
0.026541 down
0.026572 up
0.026636 down
0.026883 down
0.027289 down
0.027333 down
0.02797 down
0.02954 down
0.029868 down
0.03041 up
0.03041 down
0.030543 down
0.031347 up
0.031388 down
0.031896 up
0.032006 up
0.032267 down
0.032528 up
0.032895 down
0.032903 down
0.032922 down
0.033026 down
0.033341 down
0.033449 down
0.033694 down
0.034244 up
0.034517 down
0.034748 up

0.035014 up
0.036065 down
0.036091 down
0.036091 down
0.036095 up
0.03665 down
0.03679 down
0.036802 down
0.037262 down
0.037704 up
0.037738 down
0.03786 down
0.037988 up
0.038226 down
0.038417 down
0.038792 down
0.039031 down
0.039036 down
0.039558 down
0.039604 down
0.039615 down
0.039847 down
0.039847 down
0.039885 down
0.039897 down
0.040228 up
0.040342 down
0.041324 down
0.041333 down
0.041479 up
0.041563 down
0.041929 down
0.042966 down
0.042994 down
0.043128 down
0.043167 down
0.043918 down
0.043978 up
0.043998 up
0.044199 down
0.044917 down
0.044917 up
0.044933 up
0.045511 down
0.046078 down
0.046224 up
0.046505 down
0.047297 up
0.047463 down
0.047666 up
0.048666 down
0.049377 down
0.049514 up

Supplementary Table 14

Sample	TSS_10kb	TSS_5kb	TSS_1kb	5'UTR	CDS	3'UTR	TES_1kb	TES_5kb
F1_IP	67776	77566	101461	2971529	36591071	4013637	65556	609618
F1_Input	70836	89209	130611	2551943	34560443	4334232	70256	1261645
F2_IP	60623	69748	88751	2493804	31181199	3538559	57110	500555
F2_Input	72034	88481	131712	2630888	34926965	4355997	71826	1181482
c141_IP	58530	67930	89990	2700118	34838766	3659831	53178	558606
c141_Inpu	80774	102003	139157	3091140	42760685	5046329	75634	1600871
c142_IP	64875	75506	98331	2934374	38118945	4032408	58267	653156
c142_Inpu	79134	98289	137671	3050444	42697592	4956236	75800	1603627
ctrl1_IP	83758	93799	134152	4017049	50591012	5616855	77859	830157
ctrl1_Input	64753	80297	127845	2662943	36826403	4372704	63481	1261219
ctrl2_IP	56956	62887	90397	2789640	35275088	3730010	52335	578229
ctrl2_Input	65670	81937	126834	2690621	36588247	4374231	64348	1384234

TES_10kb	Intergenic	Intron
250987	1877662	3347533
377869	2694718	3762027
215836	1658501	2970067
366720	2618381	3789493
238439	1616329	2821709
468391	3064029	4065943
269055	1816306	3176708
479987	3004113	4119838
350794	2465166	4068580
384110	2619912	3344019
232300	1669504	2751140
405491	2671965	3364778

Supplementary Table 15

gene_id	gene_nam	chr	Start	End	M142_FPK	C_FPKM	log2FoldC	pvalue
ENSG0000(	TNC	chr9	1.15E+08	1.15E+08	30.12559	7.974353	1.929979	#######
ENSG0000(	SEMA6D	chr15	47184101	47774228	22.09428	49.2183	-1.14226	#######
ENSG0000(	SYT9	chr11	7238778	7469043	12.20281	3.287035	1.904523	1.48E-82
ENSG0000(	SERINC5	chr5	80111651	80256048	20.92562	42.45465	-1.00732	7.23E-82
ENSG0000(	ZNF33B	chr10	42574185	42638570	13.98044	29.99942	-1.08772	9.30E-57
ENSG0000(	COL6A6	chr3	1.31E+08	1.31E+08	5.10083	1.682948	1.612518	2.63E-55
ENSG0000(	SVIP	chr11	22813799	22830299	32.82461	15.54824	1.090905	3.29E-48
ENSG0000(	PPARA	chr22	46150521	46243756	2.574361	8.399001	-1.69111	5.35E-46
ENSG0000(	B4GALNT:	chr12	459939	563509	3.083466	8.802122	-1.50016	3.28E-43
ENSG0000(	FHAD1	chr1	15247272	15400283	1.788627	8.379063	-2.21422	1.38E-41
ENSG0000(	IL33	chr9	6215786	6257983	2.21382	0.058599	5.169279	2.06E-34
ENSG0000(	REST	chr4	56907876	56966808	2.990022	6.709864	-1.15207	5.68E-34
ENSG0000(	ABAT	chr16	8674596	8784575	6.390459	14.15318	-1.13384	1.92E-27
ENSG0000(	ABHD17C	chr15	80679684	80755621	14.0803	6.769926	1.068056	4.00E-25
ENSG0000(	RREB1	chr6	7107597	7251980	0.835879	2.43366	-1.52639	1.77E-24
ENSG0000(	CACNB2	chr10	18140424	18543557	5.299791	2.170183	1.299251	7.97E-23
ENSG0000(	TGFBR2	chr3	30606601	30694142	1.588857	3.986814	-1.31265	1.08E-22
ENSG0000(	GALNT7	chr4	1.73E+08	1.73E+08	0.773427	3.237838	-2.04876	7.06E-22
ENSG0000(	CSF1	chr1	1.1E+08	1.1E+08	6.314531	13.6592	-1.09965	2.42E-21
ENSG0000(	KCNK3	chr2	26692690	26733420	1.977146	4.145068	-1.05476	1.46E-20
ENSG0000(	CARF	chr2	2.03E+08	2.03E+08	5.630486	11.41477	-1.00517	3.08E-20
ENSG0000(	LIX1	chr5	97091867	97142753	0.2953	1.848409	-2.6231	3.32E-20
ENSG0000(	RIMKLA	chr1	42380792	42424232	2.995334	1.425425	1.085056	1.33E-19
ENSG0000(	B3GALT5	chr21	39556442	39673137	0.150784	0.863788	-2.49093	3.86E-17
ENSG0000(	ANKRD33	chr5	10564070	10657816	0.390061	1.237582	-1.65201	4.57E-16
ENSG0000(	DCLK3	chr3	36712422	36764349	0.191769	0.942192	-2.27071	3.80E-15
ENSG0000(	PARD3B	chr2	2.05E+08	2.06E+08	0.906714	2.208632	-1.26906	1.92E-14
ENSG0000(	SLC2A10	chr20	46709649	46736347	0.571683	2.198891	-1.92237	7.26E-14
ENSG0000(	LDLRAD4	chr18	13217498	13652755	0.626114	1.947712	-1.61877	1.23E-13
ENSG0000(	LCA5	chr6	79484991	79537458	1.322225	3.08222	-1.20543	1.29E-13
ENSG0000(	CDK5R2	chr2	2.19E+08	2.19E+08	5.082844	2.432889	1.073478	3.78E-13
ENSG0000(	COLGALT:	chr1	1.84E+08	1.84E+08	1.1995	0.298979	2.001013	1.31E-12
ENSG0000(	ARFGEF3	chr6	1.38E+08	1.38E+08	0.463715	0.202161	1.206543	2.17E-11
ENSG0000(	CCSER1	chr4	90127535	91601913	0.466073	1.815327	-1.93658	1.11E-10
ENSG0000(	DNAH1	chr3	52316319	52400491	0.332253	0.866646	-1.36371	8.96E-09
ENSG0000(	KLLN	chr10	87859158	87863437	0.402346	1.03941	-1.35072	9.44E-09
ENSG0000(	ZCWPW1	chr7	1E+08	1E+08	0.452404	1.60478	-1.80377	2.25E-08
ENSG0000(	CCDC191	chr3	1.14E+08	1.14E+08	0.862904	2.181668	-1.32056	2.95E-08
ENSG0000(	CDC42BP(	chr11	64823052	64844653	0.573308	1.633701	-1.49183	4.99E-08
ENSG0000(	FAM161B	chr14	73931501	73950414	0.950459	1.93316	-1.00801	5.07E-08
ENSG0000(	MAGEE1	chrX	76427710	76431342	1.129435	0.493411	1.206231	5.67E-08
ENSG0000(	KANK4	chr1	62236165	62319434	0.095341	0.524844	-2.41539	5.77E-08
ENSG0000(	TRIM29	chr11	1.2E+08	1.2E+08	0.104052	1.065635	-3.26569	1.29E-07
ENSG0000(	TCTE1	chr6	44278734	44297698	1.792353	0.73076	1.306459	2.36E-07
ENSG0000(	ZNF19	chr16	71464555	71565089	1.684693	3.417129	-1.00457	5.94E-07
ENSG0000(	ITPR3	chr6	33620365	33696574	0.208167	0.453829	-1.10866	1.78E-06
ENSG0000(	HOXD10	chr2	1.76E+08	1.76E+08	0.411027	1.426763	-1.77366	2.70E-06
ENSG0000(	IL1RAPL1	chrX	28587446	29956718	0.565262	0.148182	1.932834	3.86E-06
ENSG0000(	DLGAP2	chr8	737628	1708476	0.14222	0.619997	-2.0839	4.14E-06
ENSG0000(	ADAMTSL	chr1	1.51E+08	1.51E+08	0.369253	0.905996	-1.27511	7.25E-06
ENSG0000(	GBGT1	chr9	1.33E+08	1.33E+08	0.482012	1.370292	-1.4832	1.45E-05
ENSG0000(	TRANK1	chr3	36826819	36945057	0.040704	0.163554	-1.96939	1.60E-05
ENSG0000(	PDZD7	chr10	1.01E+08	1.01E+08	0.483517	1.147248	-1.22784	4.28E-05
ENSG0000(	PPP1R9A	chr7	94907202	95296415	0.242013	0.084445	1.526331	5.32E-05
ENSG0000(	STK32A	chr5	1.47E+08	1.47E+08	0.199536	0.674817	-1.72163	0.000159
ENSG0000(	EVPL	chr17	76004502	76027306	0.248005	0.576201	-1.20076	0.000176
ENSG0000(	NEBL	chr10	20779973	21293011	0.309486	0.768525	-1.29095	0.000412

ENSG0000(	FGD3	chr9	92947523	93036236	0.397502	0.847998	-1.07326	0.00043
ENSG0000(	RASD1	chr17	17494437	17496395	1.445027	0.723581	1.006246	0.000483
ENSG0000(	RTL1	chr14	1.01E+08	1.01E+08	0.448785	0.221248	1.025827	0.00051
ENSG0000(	CFAP58	chr10	1.04E+08	1.04E+08	0.20402	0.611781	-1.55786	0.000539
ENSG0000(	BCHE	chr3	1.66E+08	1.66E+08	0.441388	0.117253	1.899482	0.000614
ENSG0000(	SPATA17	chr1	2.18E+08	2.18E+08	0.570128	1.182192	-1.03531	0.000831
ENSG0000(	LONRF3	chrX	1.19E+08	1.19E+08	0.064211	0.274818	-2.03531	0.000988
ENSG0000(	REEP1	chr2	86213993	86338083	0.753118	0.335169	1.173305	0.002005
ENSG0000(	CYP1A1	chr15	74719542	74725536	0.524138	0.196944	1.403236	0.0023
ENSG0000(	YWHAH	chr22	31944522	31957603	98.06753	48.49844	1.075662	5.09E-14
ENSG0000(	ATP2B3	chrX	1.54E+08	1.54E+08	0.149454	0.345061	-1.18869	0.003146
ENSG0000(	TCHH	chr1	1.52E+08	1.52E+08	0.05108	0.007605	2.64185	0.006514
ENSG0000(	ZDHHC11	chr5	795606	850986	0.313746	0.770696	-1.27044	0.008141
ENSG0000(	EDN2	chr1	41478775	41484683	0.83599	0.276296	1.594487	0.013913
ENSG0000(	PCDHA1	chr5	1.41E+08	1.41E+08	0.2792	0.134984	1.046317	0.016174
ENSG0000(	RAI2	chrX	17800049	17861337	0.025749	0.158368	-2.46546	0.017248

padj	level
#######	up
#######	down
3.04E-80	up
1.43E-79	down
8.60E-55	down
2.28E-53	up
2.36E-46	up
3.44E-44	down
1.87E-41	down
7.19E-40	down
7.82E-33	up
2.09E-32	down
5.23E-26	down
9.38E-24	up
3.97E-23	down
1.59E-21	up
2.13E-21	down
1.29E-20	down
4.29E-20	down
2.44E-19	down
5.01E-19	down
5.39E-19	down
2.06E-18	up
4.95E-16	down
5.38E-15	down
4.13E-14	down
1.95E-13	down
7.01E-13	down
1.16E-12	down
1.22E-12	down
3.41E-12	up
1.12E-11	up
1.66E-10	up
7.88E-10	down
5.18E-08	down
5.45E-08	down
1.24E-07	down
1.61E-07	down
2.64E-07	down
2.68E-07	down
2.97E-07	up
3.03E-07	down
6.48E-07	down
1.15E-06	up
2.75E-06	down
7.71E-06	down
1.14E-05	down
1.59E-05	up
1.70E-05	down
2.87E-05	down
5.47E-05	down
5.99E-05	down
0.00015	down
0.000184	up
0.000509	down
0.00056	down
0.001237	down

0.001286	down
0.001428	up
0.001498	up
0.001576	down
0.001774	up
0.002342	down
0.00275	down
0.005253	up
0.005956	up
4.99E-13	up
0.007921	down
0.015327	up
0.018795	down
0.03041	up
0.034748	up
0.03679	down

Supplementary Table 16

gene_id	gene_nam	chr	Start	End	M142_FPK	C_FPKM	log2FoldC	pvalue
ENSG0000(	YWHAH	chr22	31944522	31957603	98.06753	48.49844	1.075662	5.09E-14
ENSG0000(	SEMA6D	chr15	47184101	47774228	22.09428	49.2183	-1.14226	6.4E-122
ENSG0000(	SYT9	chr11	7238778	7469043	12.20281	3.287035	1.904523	1.48E-82
ENSG0000(	COL6A6	chr3	1.31E+08	1.31E+08	5.10083	1.682948	1.612518	2.63E-55
ENSG0000(	ABHD17C	chr15	80679684	80755621	14.0803	6.769926	1.068056	4E-25
ENSG0000(	CDK5R2	chr2	2.19E+08	2.19E+08	5.082844	2.432889	1.073478	3.78E-13
ENSG0000(	SERINC5	chr5	80111651	80256048	20.92562	42.45465	-1.00732	7.23E-82
ENSG0000(	ZNF33B	chr10	42574185	42638570	13.98044	29.99942	-1.08772	9.3E-57
ENSG0000(	PPARA	chr22	46150521	46243756	2.574361	8.399001	-1.69111	5.35E-46
ENSG0000(	B4GALNT:	chr12	459939	563509	3.083466	8.802122	-1.50016	3.28E-43
ENSG0000(	FHAD1	chr1	15247272	15400283	1.788627	8.379063	-2.21422	1.38E-41
ENSG0000(	REST	chr4	56907876	56966808	2.990022	6.709864	-1.15207	5.68E-34
ENSG0000(	ABAT	chr16	8674596	8784575	6.390459	14.15318	-1.13384	1.92E-27
ENSG0000(	TGFBR2	chr3	30606601	30694142	1.588857	3.986814	-1.31265	1.08E-22
ENSG0000(	CARF	chr2	2.03E+08	2.03E+08	5.630486	11.41477	-1.00517	3.08E-20
ENSG0000(	ZCWPW1	chr7	1E+08	1E+08	0.452404	1.60478	-1.80377	2.25E-08
ENSG0000(	CCDC191	chr3	1.14E+08	1.14E+08	0.862904	2.181668	-1.32056	2.95E-08
ENSG0000(	CDC42BP(	chr11	64823052	64844653	0.573308	1.633701	-1.49183	4.99E-08
ENSG0000(	TRIM29	chr11	1.2E+08	1.2E+08	0.104052	1.065635	-3.26569	1.29E-07
ENSG0000(	ZNF19	chr16	71464555	71565089	1.684693	3.417129	-1.00457	5.94E-07

padj	level
4.99E-13	up
3.2E-119	down
3.04E-80	up
2.28E-53	up
9.38E-24	up
3.41E-12	up
1.43E-79	down
8.6E-55	down
3.44E-44	down
1.87E-41	down
7.19E-40	down
2.09E-32	down
5.23E-26	down
2.13E-21	down
5.01E-19	down
1.24E-07	down
1.61E-07	down
2.64E-07	down
6.48E-07	down
2.75E-06	down

Supplementary Table 17

GSE45547	rownames	Type	values	GSE49710	rownames	Type	values
	GSM1108	G1(n=149	14.46432		GSM1205	G1(n=120	14.30853
	GSM1108	G1(n=149	14.45192		GSM1205	G1(n=120	14.50889
	GSM1108	G1(n=149	13.38725		GSM1205	G1(n=120	13.47687
	GSM1108	G1(n=149	13.6972		GSM1205	G1(n=120	14.96758
	GSM1108	G1(n=149	13.06268		GSM1205	G1(n=120	14.83379
	GSM1108	G1(n=149	14.19212		GSM1205	G1(n=120	15.1751
	GSM1108	G1(n=149	14.84131		GSM1205	G1(n=120	14.31722
	GSM1108	G1(n=149	14.39921		GSM1205	G1(n=120	14.75037
	GSM1108	G1(n=149	15.2584		GSM1205	G1(n=120	14.63144
	GSM1108	G1(n=149	14.72948		GSM1205	G1(n=120	13.90918
	GSM1108	G1(n=149	13.9137		GSM1205	G1(n=120	14.34382
	GSM1108	G1(n=149	14.00794		GSM1205	G1(n=120	14.73118
	GSM1108	G1(n=149	13.82665		GSM1205	G1(n=120	14.89565
	GSM1108	G1(n=149	14.42494		GSM1205	G1(n=120	14.26254
	GSM1108	G1(n=149	14.25796		GSM1205	G1(n=120	14.38383
	GSM1108	G1(n=149	14.22207		GSM1205	G1(n=120	14.45969
	GSM1108	G1(n=149	14.25555		GSM1205	G1(n=120	13.50969
	GSM1108	G1(n=149	14.21379		GSM1205	G1(n=120	13.64541
	GSM1108	G1(n=149	14.21979		GSM1205	G1(n=120	14.59278
	GSM1108	G1(n=149	13.89187		GSM1205	G1(n=120	14.43895
	GSM1108	G1(n=149	13.65815		GSM1205	G1(n=120	14.30648
	GSM1108	G1(n=149	14.97169		GSM1205	G1(n=120	13.98949
	GSM1108	G1(n=149	12.87565		GSM1205	G1(n=120	13.91067
	GSM1108	G1(n=149	14.09351		GSM1205	G1(n=120	13.8394
	GSM1108	G1(n=149	13.85383		GSM1205	G1(n=120	14.63986
	GSM1108	G1(n=149	14.78902		GSM1205	G1(n=120	14.75365
	GSM1108	G1(n=149	14.10148		GSM1205	G1(n=120	13.99442
	GSM1108	G1(n=149	14.13056		GSM1205	G1(n=120	13.57215
	GSM1108	G1(n=149	14.69314		GSM1205	G1(n=120	14.54147
	GSM1108	G1(n=149	14.37204		GSM1205	G1(n=120	14.16061
	GSM1108	G1(n=149	14.18795		GSM1205	G1(n=120	13.62218
	GSM1108	G1(n=149	13.77918		GSM1205	G1(n=120	14.79133
	GSM1108	G1(n=149	13.96875		GSM1205	G1(n=120	14.29069
	GSM1108	G1(n=149	14.17949		GSM1205	G1(n=120	14.2145
	GSM1108	G1(n=149	14.46197		GSM1205	G1(n=120	14.74892
	GSM1108	G1(n=149	13.7659		GSM1205	G1(n=120	14.17876
	GSM1108	G1(n=149	14.08384		GSM1205	G1(n=120	13.94912
	GSM1108	G1(n=149	14.8322		GSM1205	G1(n=120	14.43569
	GSM1108	G1(n=149	14.64361		GSM1205	G1(n=120	14.26381
	GSM1108	G1(n=149	14.2072		GSM1205	G1(n=120	14.80036
	GSM1108	G1(n=149	13.97573		GSM1205	G1(n=120	13.86907
	GSM1108	G1(n=149	13.87724		GSM1205	G1(n=120	14.05519
	GSM1108	G1(n=149	13.66975		GSM1205	G1(n=120	14.53396
	GSM1108	G1(n=149	14.53406		GSM1205	G1(n=120	14.3976
	GSM1108	G1(n=149	14.27855		GSM1205	G1(n=120	14.60499
	GSM1108	G1(n=149	14.54409		GSM1205	G1(n=120	14.8928
	GSM1108	G1(n=149	14.08003		GSM1205	G1(n=120	14.35882
	GSM1108	G1(n=149	14.22609		GSM1205	G1(n=120	14.84107
	GSM1108	G1(n=149	14.54198		GSM1205	G1(n=120	13.43511
	GSM1108	G1(n=149	13.27643		GSM1205	G1(n=120	14.24796
	GSM1108	G1(n=149	14.20041		GSM1205	G1(n=120	14.60197
	GSM1108	G1(n=149	14.28928		GSM1205	G1(n=120	14.16331
	GSM1108	G1(n=149	14.66275		GSM1205	G1(n=120	14.30195
	GSM1108	G1(n=149	14.9294		GSM1205	G1(n=120	14.99844
	GSM1108	G1(n=149	14.84506		GSM1205	G1(n=120	14.61503
	GSM1108	G1(n=149	14.56348		GSM1205	G1(n=120	14.27633
	GSM1108	G1(n=149	14.43008		GSM1205	G1(n=120	14.21262

GSM1108(	G1(n=149	13.80675	GSM1205;	G1(n=120	14.10372
GSM1108(	G1(n=149	13.48833	GSM1205;	G1(n=120	15.29065
GSM1108(	G1(n=149	14.19858	GSM1205;	G1(n=120	15.48077
GSM1108(	G1(n=149	13.95089	GSM1205;	G1(n=120	14.4161
GSM1108'	G1(n=149	14.24508	GSM1205;	G1(n=120	14.50339
GSM1108'	G1(n=149	14.68745	GSM1205‹	G1(n=120	14.49489
GSM1108'	G1(n=149	14.5838	GSM1205‹	G1(n=120	14.57636
GSM1108'	G1(n=149	13.91876	GSM1205‹	G1(n=120	13.6687
GSM1108'	G1(n=149	14.52275	GSM1205‹	G1(n=120	13.48721
GSM1108'	G1(n=149	14.33869	GSM1205‹	G1(n=120	14.29482
GSM1108'	G1(n=149	14.69314	GSM1205‹	G1(n=120	13.39003
GSM1108'	G1(n=149	13.31141	GSM1205‹	G1(n=120	13.37631
GSM1108'	G1(n=149	13.9137	GSM1205‹	G1(n=120	13.99856
GSM1108'	G1(n=149	13.39212	GSM1205‹	G1(n=120	14.09398
GSM1108'	G1(n=149	14.92627	GSM1205‹	G1(n=120	14.26014
GSM1108'	G1(n=149	14.78594	GSM1205‹	G1(n=120	14.23433
GSM1108'	G1(n=149	15.13144	GSM1205‹	G1(n=120	14.29886
GSM1108'	G1(n=149	14.23485	GSM1205‹	G1(n=120	14.20805
GSM1108'	G1(n=149	13.83279	GSM1205‹	G1(n=120	13.29337
GSM1108'	G1(n=149	14.27165	GSM1205‹	G1(n=120	14.47223
GSM1108'	G1(n=149	14.66817	GSM1205‹	G1(n=120	13.91376
GSM1108'	G1(n=149	14.84848	GSM1205‹	G1(n=120	14.09697
GSM1108'	G1(n=149	14.17949	GSM1205‹	G1(n=120	14.89565
GSM1108'	G1(n=149	14.32042	GSM1205‹	G1(n=120	14.52226
GSM1108'	G1(n=149	14.38995	GSM1205‹	G1(n=120	14.49043
GSM1108'	G1(n=149	13.55384	GSM1205‹	G1(n=120	14.09244
GSM1108'	G1(n=149	13.40598	GSM1205‹	G1(n=120	14.19318
GSM1108'	G1(n=149	14.73236	GSM1205‹	G1(n=120	14.31822
GSM1108'	G1(n=149	14.47767	GSM1205‹	G1(n=120	14.37823
GSM1108'	G1(n=149	13.53491	GSM1205‹	G1(n=120	14.33023
GSM1108'	G1(n=149	14.36342	GSM1205‹	G1(n=120	13.56525
GSM1108'	G1(n=149	14.23082	GSM1205‹	G1(n=120	13.7927
GSM1108'	G1(n=149	13.6541	GSM1205!	G1(n=120	14.50224
GSM1108'	G1(n=149	13.63594	GSM1205!	G1(n=120	13.74101
GSM1108'	G1(n=149	13.92945	GSM1205!	G1(n=120	14.30438
GSM1108'	G1(n=149	15.15466	GSM1205!	G1(n=120	14.13839
GSM1108'	G1(n=149	14.02077	GSM1205!	G1(n=120	14.52566
GSM1108'	G1(n=149	13.92945	GSM1205!	G1(n=120	14.07214
GSM1108'	G1(n=149	14.53674	GSM1205!	G1(n=120	13.40843
GSM1108'	G1(n=149	14.58113	GSM1205!	G1(n=120	14.17112
GSM1108{	G1(n=149	14.38756	GSM1205!	G1(n=120	13.97602
GSM1108{	G1(n=149	14.41111	GSM1205!	G1(n=120	14.29972
GSM1108{	G1(n=149	14.54409	GSM1205!	G1(n=120	14.32914
GSM1108{	G1(n=149	13.61323	GSM1205!	G1(n=120	14.35978
GSM1108{	G1(n=149	13.85725	GSM1205(	G1(n=120	14.17305
GSM1108{	G1(n=149	14.13238	GSM1205(	G1(n=120	14.27094
GSM1108{	G1(n=149	14.43478	GSM1205(	G1(n=120	13.76311
GSM1108{	G1(n=149	13.30351	GSM1205(	G1(n=120	14.10642
GSM1108{	G1(n=149	14.42241	GSM1205(	G1(n=120	14.92128
GSM1108{	G1(n=149	14.5101	GSM1205(	G1(n=120	14.47023
GSM1108{	G1(n=149	14.05903	GSM1205'	G1(n=120	13.78604
GSM1108{	G1(n=149	14.01503	GSM1205'	G1(n=120	14.46385
GSM1108{	G1(n=149	13.48133	GSM1205'	G1(n=120	14.64245
GSM1108{	G1(n=149	14.02839	GSM1205'	G1(n=120	13.77492
GSM1108{	G1(n=149	14.64112	GSM1205'	G1(n=120	13.94524
GSM1108{	G1(n=149	14.3323	GSM1205'	G1(n=120	14.43895
GSM1108{	G1(n=149	14.22407	GSM1205'	G1(n=120	13.57106
GSM1108{	G1(n=149	14.01889	GSM1205'	G1(n=120	14.8928

GSM11088	G1(n=149	14.14089
GSM11088	G1(n=149	13.1743
GSM11088	G1(n=149	13.57896
GSM11088	G1(n=149	14.15268
GSM11088	G1(n=149	14.44436
GSM11088	G1(n=149	15.45039
GSM11088	G1(n=149	14.21979
GSM11088	G1(n=149	14.30219
GSM11088	G1(n=149	14.11361
GSM11088	G1(n=149	14.45192
GSM11089	G1(n=149	14.07811
GSM11089	G1(n=149	13.67131
GSM11089	G1(n=149	14.17358
GSM11089	G1(n=149	13.2796
GSM11089	G1(n=149	14.00036
GSM11089	G1(n=149	12.84246
GSM11089	G1(n=149	13.23117
GSM11089	G1(n=149	14.85907
GSM11089	G1(n=149	14.66544
GSM11089	G1(n=149	14.89844
GSM11089	G1(n=149	13.81342
GSM11089	G1(n=149	14.38756
GSM11089	G1(n=149	14.75534
GSM11089	G1(n=149	13.66243
GSM11089	G1(n=149	14.64618
GSM11089	G1(n=149	13.98664
GSM11090	G1(n=149	13.90848
GSM11090	G1(n=149	14.00605
GSM11090	G1(n=149	14.2072
GSM11090	G1(n=149	14.25796
GSM11090	G1(n=149	14.77356
GSM11090	G1(n=149	14.52843
GSM11090	G1(n=149	13.11777
GSM11090	G1(n=149	13.94023
GSM11084	G2(n=83)	13.6306
GSM11084	G2(n=83)	14.63627
GSM11084	G2(n=83)	13.515
GSM11084	G2(n=83)	14.31144
GSM11084	G2(n=83)	13.22452
GSM11084	G2(n=83)	14.614
GSM11084	G2(n=83)	14.75669
GSM11084	G2(n=83)	14.72445
GSM11084	G2(n=83)	13.42912
GSM11084	G2(n=83)	13.93283
GSM11084	G2(n=83)	14.50421
GSM11085	G2(n=83)	14.03908
GSM11085	G2(n=83)	13.95876
GSM11085	G2(n=83)	13.6067
GSM11085	G2(n=83)	14.02968
GSM11085	G2(n=83)	14.1548
GSM11085	G2(n=83)	13.76274
GSM11085	G2(n=83)	13.59486
GSM11085	G2(n=83)	13.25048
GSM11085	G2(n=83)	14.29251
GSM11085	G2(n=83)	13.49894
GSM11085	G2(n=83)	14.09408
GSM11085	G2(n=83)	13.61166
GSM11085	G2(n=83)	14.22334

GSM12057	G1(n=120	14.69244
GSM12057	G1(n=120	14.27827
GSM12057	G1(n=120	14.05519
GSM12057	G1(n=120	13.97313
GSM12057	G1(n=120	13.76106
GSM12052	G2(n=62)	13.426
GSM12052	G2(n=62)	13.58118
GSM12053	G2(n=62)	14.4798
GSM12053	G2(n=62)	14.65273
GSM12053	G2(n=62)	13.49758
GSM12053	G2(n=62)	13.61001
GSM12053	G2(n=62)	14.11341
GSM12053	G2(n=62)	14.32949
GSM12053	G2(n=62)	14.21266
GSM12053	G2(n=62)	13.78727
GSM12053	G2(n=62)	14.73364
GSM12053	G2(n=62)	13.81061
GSM12053	G2(n=62)	14.37911
GSM12053	G2(n=62)	13.95778
GSM12053	G2(n=62)	15.02364
GSM12053	G2(n=62)	13.33423
GSM12053	G2(n=62)	12.98878
GSM12053	G2(n=62)	14.27405
GSM12053	G2(n=62)	14.07215
GSM12053	G2(n=62)	14.04616
GSM12053	G2(n=62)	14.49441
GSM12053	G2(n=62)	14.48461
GSM12053	G2(n=62)	14.25566
GSM12053	G2(n=62)	13.85284
GSM12053	G2(n=62)	13.72372
GSM12053	G2(n=62)	13.20772
GSM12053	G2(n=62)	13.15704
GSM12053	G2(n=62)	13.45996
GSM12053	G2(n=62)	14.54977
GSM12053	G2(n=62)	13.96453
GSM12053	G2(n=62)	14.67992
GSM12053	G2(n=62)	13.17601
GSM12054	G2(n=62)	13.30873
GSM12054	G2(n=62)	14.7138
GSM12054	G2(n=62)	13.0222
GSM12054	G2(n=62)	14.55208
GSM12054	G2(n=62)	13.91479
GSM12054	G2(n=62)	14.62607
GSM12054	G2(n=62)	13.4925
GSM12054	G2(n=62)	14.29453
GSM12054	G2(n=62)	13.85777
GSM12054	G2(n=62)	13.40091
GSM12054	G2(n=62)	13.04984
GSM12054	G2(n=62)	13.58034
GSM12054	G2(n=62)	13.53062
GSM12054	G2(n=62)	14.43473
GSM12054	G2(n=62)	13.84071
GSM12054	G2(n=62)	13.56608
GSM12054	G2(n=62)	13.19108
GSM12054	G2(n=62)	13.47371
GSM12054	G2(n=62)	13.75462
GSM12054	G2(n=62)	12.85067
GSM12054	G2(n=62)	13.15246

GSM1108	G2(n=83)	14.05026
GSM1108	G2(n=83)	13.17896
GSM1108	G2(n=83)	14.51144
GSM1108	G2(n=83)	14.51472
GSM1108	G2(n=83)	13.86624
GSM1108	G2(n=83)	13.22877
GSM1108	G2(n=83)	14.28582
GSM1108	G2(n=83)	13.754
GSM1108	G2(n=83)	14.82306
GSM1108	G2(n=83)	14.67377
GSM1108	G2(n=83)	13.53133
GSM1108	G2(n=83)	14.39584
GSM1108	G2(n=83)	13.68196
GSM1108	G2(n=83)	13.64122
GSM1108	G2(n=83)	14.13596
GSM1108	G2(n=83)	14.35472
GSM1108	G2(n=83)	14.57202
GSM1108	G2(n=83)	13.98755
GSM1108	G2(n=83)	13.22252
GSM1108	G2(n=83)	13.37632
GSM1108	G2(n=83)	14.24242
GSM1108	G2(n=83)	13.46435
GSM1108	G2(n=83)	13.82294
GSM1108	G2(n=83)	13.9894
GSM1108	G2(n=83)	15.03878
GSM1108	G2(n=83)	13.81242
GSM1108	G2(n=83)	14.75392
GSM1108	G2(n=83)	13.03391
GSM1108	G2(n=83)	14.50421
GSM1108	G2(n=83)	13.84298
GSM1108	G2(n=83)	14.53431
GSM1108	G2(n=83)	13.5027
GSM1108	G2(n=83)	13.77685
GSM1108	G2(n=83)	13.5697
GSM1108	G2(n=83)	13.58916
GSM1108	G2(n=83)	13.3349
GSM1108	G2(n=83)	14.57202
GSM1108	G2(n=83)	13.04672
GSM1108	G2(n=83)	13.49894
GSM1108	G2(n=83)	13.18
GSM1108	G2(n=83)	13.21166
GSM1108	G2(n=83)	12.86737
GSM1108	G2(n=83)	13.87399
GSM1108	G2(n=83)	12.89495
GSM1108	G2(n=83)	12.90991
GSM1108	G2(n=83)	12.9547
GSM1108	G2(n=83)	13.03734
GSM1108	G2(n=83)	13.45726
GSM1108	G2(n=83)	13.23721
GSM1108	G2(n=83)	15.06222
GSM1108	G2(n=83)	15.14458
GSM1108	G2(n=83)	14.64183
GSM1109	G2(n=83)	14.04831
GSM1109	G2(n=83)	14.34721
GSM1109	G2(n=83)	13.701
GSM1109	G2(n=83)	13.45372
GSM1109	G2(n=83)	14.60853
GSM1109	G2(n=83)	14.25801

GSM1205	G2(n=62)	13.73624
GSM1205	G2(n=62)	14.14303
GSM1205	G2(n=62)	13.56262
GSM1205	G2(n=62)	14.4798
GSM1205	G2(n=62)	12.89184
GSM1205	G2(n=62)	14.3364
GSM1205	G2(n=62)	15.3845
GSM1205	G2(n=62)	14.12355
GSM1205	G2(n=62)	12.3402
GSM1205	G3(n=233	13.17697
GSM1205	G3(n=233	13.94588
GSM1205	G3(n=233	13.19618
GSM1205	G3(n=233	13.35477
GSM1205	G3(n=233	13.08838
GSM1205	G3(n=233	13.63251
GSM1205	G3(n=233	13.72399
GSM1205	G3(n=233	13.01852
GSM1205	G3(n=233	13.24308
GSM1205	G3(n=233	13.83598
GSM1205	G3(n=233	13.2758
GSM1205	G3(n=233	13.41393
GSM1205	G3(n=233	14.34023
GSM1205	G3(n=233	13.46818
GSM1205	G3(n=233	14.08509
GSM1205	G3(n=233	13.20054
GSM1205	G3(n=233	14.07131
GSM1205	G3(n=233	14.31044
GSM1205	G3(n=233	13.64879
GSM1205	G3(n=233	13.11084
GSM1205	G3(n=233	13.43931
GSM1205	G3(n=233	13.40215
GSM1205	G3(n=233	13.28255
GSM1205	G3(n=233	13.93111
GSM1205	G3(n=233	13.72339
GSM1205	G3(n=233	13.13123
GSM1205	G3(n=233	14.53554
GSM1205	G3(n=233	13.02245
GSM1205	G3(n=233	13.42215
GSM1205	G3(n=233	12.17253
GSM1205	G3(n=233	13.65435
GSM1205	G3(n=233	13.20187
GSM1205	G3(n=233	13.5612
GSM1205	G3(n=233	14.01653
GSM1205	G3(n=233	12.5781
GSM1205	G3(n=233	13.89127
GSM1205	G3(n=233	14.7794
GSM1205	G3(n=233	13.84572
GSM1205	G3(n=233	13.76754
GSM1205	G3(n=233	13.17073
GSM1205	G3(n=233	12.96901
GSM1205	G3(n=233	14.40887
GSM1205	G3(n=233	13.07931
GSM1205	G3(n=233	14.47757
GSM1205	G3(n=233	13.62846
GSM1205	G3(n=233	13.33994
GSM1205	G3(n=233	13.80223
GSM1205	G3(n=233	13.75197
GSM1205	G3(n=233	13.41525

GSM1109(	G2(n=83)	14.04487
GSM1108‹	G3(n=271	13.90718
GSM1108‹	G3(n=271	13.2992
GSM1108‹	G3(n=271	14.00451
GSM1108‹	G3(n=271	14.61623
GSM1108‹	G3(n=271	13.64333
GSM1108‹	G3(n=271	13.21494
GSM1108‹	G3(n=271	13.99896
GSM1108‹	G3(n=271	13.75316
GSM1108‹	G3(n=271	14.38606
GSM1108‹	G3(n=271	13.88532
GSM1108‹	G3(n=271	13.45278
GSM1108‹	G3(n=271	14.30209
GSM1108‹	G3(n=271	14.48391
GSM1108‹	G3(n=271	13.45978
GSM1108‹	G3(n=271	14.1433
GSM1108‹	G3(n=271	14.32591
GSM1108‹	G3(n=271	14.14523
GSM1108‹	G3(n=271	13.9971
GSM1108‹	G3(n=271	13.67458
GSM1108‹	G3(n=271	13.62587
GSM1108‹	G3(n=271	13.0477
GSM1108‹	G3(n=271	13.71046
GSM1108‹	G3(n=271	14.17504
GSM1108‹	G3(n=271	13.70187
GSM1108‹	G3(n=271	13.75023
GSM1108‹	G3(n=271	14.77451
GSM1108‹	G3(n=271	13.52622
GSM1108‹	G3(n=271	13.89178
GSM1108‹	G3(n=271	13.42021
GSM1108‹	G3(n=271	13.66076
GSM1108‹	G3(n=271	13.46882
GSM1108‹	G3(n=271	13.9056
GSM1108‹	G3(n=271	13.49901
GSM1108‹	G3(n=271	14.29108
GSM1108‹	G3(n=271	13.77506
GSM1108‹	G3(n=271	13.24785
GSM1108‹	G3(n=271	13.98051
GSM1108‹	G3(n=271	13.67972
GSM1108‹	G3(n=271	13.35771
GSM1108‹	G3(n=271	14.01459
GSM1108‹	G3(n=271	13.81923
GSM1108‹	G3(n=271	12.96691
GSM1108‹	G3(n=271	13.07685
GSM1108‹	G3(n=271	12.30383
GSM1108‹	G3(n=271	14.08837
GSM1108‹	G3(n=271	14.13568
GSM1108‹	G3(n=271	13.32403
GSM1108‹	G3(n=271	14.15298
GSM1108‹	G3(n=271	14.00774
GSM1108!	G3(n=271	13.81923
GSM1108!	G3(n=271	13.89776
GSM1108!	G3(n=271	13.25611
GSM1108!	G3(n=271	13.96163
GSM1108!	G3(n=271	13.92939
GSM1108!	G3(n=271	13.56606
GSM1108!	G3(n=271	13.69924
GSM1108!	G3(n=271	13.71879

GSM1205‹	G3(n=233	13.61696
GSM1205‹	G3(n=233	12.7984
GSM1205‹	G3(n=233	13.39581
GSM1205‹	G3(n=233	13.16596
GSM1205‹	G3(n=233	13.00922
GSM1205‹	G3(n=233	13.50992
GSM1205!	G3(n=233	14.25075
GSM1205!	G3(n=233	13.14422
GSM1205!	G3(n=233	12.95349
GSM1205!	G3(n=233	13.01118
GSM1205!	G3(n=233	13.13601
GSM1205!	G3(n=233	13.19013
GSM1205!	G3(n=233	12.94047
GSM1205!	G3(n=233	13.56796
GSM1205!	G3(n=233	13.61148
GSM1205!	G3(n=233	13.19117
GSM1205!	G3(n=233	14.47501
GSM1205!	G3(n=233	13.53176
GSM1205!	G3(n=233	13.91454
GSM1205!	G3(n=233	13.50582
GSM1205!	G3(n=233	13.98165
GSM1205!	G3(n=233	13.06293
GSM1205!	G3(n=233	14.50125
GSM1205!	G3(n=233	13.94808
GSM1205!	G3(n=233	14.91083
GSM1205!	G3(n=233	13.04082
GSM1205!	G3(n=233	13.66845
GSM1205!	G3(n=233	13.46456
GSM1205!	G3(n=233	13.91654
GSM1205!	G3(n=233	13.75742
GSM1205!	G3(n=233	13.52333
GSM1205!	G3(n=233	13.03445
GSM1205!	G3(n=233	14.01931
GSM1205!	G3(n=233	12.95645
GSM1205!	G3(n=233	12.76344
GSM1205!	G3(n=233	13.13016
GSM1205!	G3(n=233	14.00422
GSM1205!	G3(n=233	14.61654
GSM1205!	G3(n=233	13.61832
GSM1205!	G3(n=233	13.19355
GSM1205!	G3(n=233	13.32555
GSM1205!	G3(n=233	12.93772
GSM1205!	G3(n=233	14.31913
GSM1205!	G3(n=233	14.04716
GSM1205!	G3(n=233	13.73896
GSM1205!	G3(n=233	13.75197
GSM1205!	G3(n=233	13.81915
GSM1205!	G3(n=233	14.23268
GSM1205!	G3(n=233	14.06768
GSM1205!	G3(n=233	14.54343
GSM1205!	G3(n=233	13.65685
GSM1205!	G3(n=233	13.67519
GSM1205!	G3(n=233	13.42554
GSM1205!	G3(n=233	13.72399
GSM1205!	G3(n=233	12.83357
GSM1205!	G3(n=233	13.43471
GSM1205!	G3(n=233	13.79188
GSM1205!	G3(n=233	13.33871

GSM1108!	G3(n=271	13.52364
GSM1108!	G3(n=271	13.63638
GSM1108!	G3(n=271	13.52364
GSM1108!	G3(n=271	13.38114
GSM1108!	G3(n=271	12.79514
GSM1108!	G3(n=271	13.26699
GSM1108!	G3(n=271	12.96503
GSM1108!	G3(n=271	13.68106
GSM1108!	G3(n=271	13.82539
GSM1108!	G3(n=271	13.98051
GSM1108!	G3(n=271	13.65948
GSM1108!	G3(n=271	12.84721
GSM1108!	G3(n=271	13.08346
GSM1108!	G3(n=271	13.18412
GSM1108!	G3(n=271	13.71729
GSM1108!	G3(n=271	13.06065
GSM1108!	G3(n=271	13.76764
GSM1108!	G3(n=271	13.55481
GSM1108!	G3(n=271	14.00617
GSM1108!	G3(n=271	13.57747
GSM1108!	G3(n=271	13.83619
GSM1108!	G3(n=271	13.20061
GSM1108!	G3(n=271	14.59738
GSM1108!	G3(n=271	13.14713
GSM1108!	G3(n=271	13.61649
GSM1108!	G3(n=271	13.79442
GSM1108!	G3(n=271	13.91514
GSM1108!	G3(n=271	13.36347
GSM1108!	G3(n=271	13.8108
GSM1108!	G3(n=271	13.74748
GSM1108!	G3(n=271	13.30251
GSM1108!	G3(n=271	13.71729
GSM1108!	G3(n=271	13.64862
GSM1108!	G3(n=271	13.0796
GSM1108(	G3(n=271	13.29808
GSM1108(	G3(n=271	13.51137
GSM1108(	G3(n=271	13.25611
GSM1108(	G3(n=271	13.45278
GSM1108(	G3(n=271	13.21088
GSM1108(	G3(n=271	13.96163
GSM1108(	G3(n=271	13.4365
GSM1108(	G3(n=271	13.51883
GSM1108(	G3(n=271	14.10352
GSM1108(	G3(n=271	13.06443
GSM1108(	G3(n=271	14.5526
GSM1108(	G3(n=271	14.05168
GSM1108(	G3(n=271	13.49784
GSM1108(	G3(n=271	14.96512
GSM1108(	G3(n=271	13.41563
GSM1108(	G3(n=271	13.38657
GSM1108(	G3(n=271	13.5139
GSM1108(	G3(n=271	14.54517
GSM1108(	G3(n=271	13.63638
GSM1108(	G3(n=271	13.9971
GSM1108(	G3(n=271	13.37447
GSM1108(	G3(n=271	12.83615
GSM1108(	G3(n=271	14.41609
GSM1108(	G3(n=271	13.73449

GSM1205!	G3(n=233	13.62951
GSM1205!	G3(n=233	13.81576
GSM1205!	G3(n=233	13.44187
GSM1205!	G3(n=233	13.18584
GSM1205!	G3(n=233	13.92563
GSM1205!	G3(n=233	13.30674
GSM1205!	G3(n=233	12.94096
GSM1205!	G3(n=233	14.09367
GSM1205!	G3(n=233	12.66445
GSM1205!	G3(n=233	13.16361
GSM1205!	G3(n=233	13.3731
GSM1205!	G3(n=233	13.81326
GSM1205!	G3(n=233	13.41088
GSM1205!	G3(n=233	13.47395
GSM1205!	G3(n=233	13.97893
GSM1205!	G3(n=233	12.83086
GSM1205!	G3(n=233	13.40884
GSM1205!	G3(n=233	13.44841
GSM1205!	G3(n=233	13.7373
GSM1205!	G3(n=233	13.90288
GSM1205!	G3(n=233	13.53423
GSM1205!	G3(n=233	13.70696
GSM1205!	G3(n=233	13.12056
GSM1205!	G3(n=233	13.01019
GSM1205!	G3(n=233	12.70513
GSM1205(	G3(n=233	13.32214
GSM1205(	G3(n=233	13.7507
GSM1205(	G3(n=233	13.59606
GSM1205(	G3(n=233	13.61979
GSM1205(	G3(n=233	13.17804
GSM1205(	G3(n=233	13.62484
GSM1205(	G3(n=233	13.57075
GSM1205(	G3(n=233	14.21687
GSM1205(	G3(n=233	13.08139
GSM1205(	G3(n=233	13.14475
GSM1205(	G3(n=233	12.79413
GSM1205(	G3(n=233	13.93779
GSM1205(	G3(n=233	13.11282
GSM1205(	G3(n=233	13.56059
GSM1205(	G3(n=233	13.90448
GSM1205(	G3(n=233	12.84138
GSM1205(	G3(n=233	13.51364
GSM1205(	G3(n=233	12.71389
GSM1205(	G3(n=233	13.18058
GSM1205(	G3(n=233	13.10395
GSM1205(	G3(n=233	14.90553
GSM1205(	G3(n=233	13.32214
GSM1205(	G3(n=233	13.019
GSM1205(	G3(n=233	13.56796
GSM1205(	G3(n=233	13.89936
GSM1205(	G3(n=233	13.94122
GSM1205(	G3(n=233	13.67125
GSM1205(	G3(n=233	13.68968
GSM1205(	G3(n=233	13.30946
GSM1205(	G3(n=233	13.3984
GSM1205(	G3(n=233	13.96971
GSM1205(	G3(n=233	13.7682
GSM1205(	G3(n=233	12.71201

GSM1108(	G3(n=271	13.57617
GSM1108(	G3(n=271	13.69924
GSM1108(	G3(n=271	14.16497
GSM1108(	G3(n=271	13.71729
GSM1108(	G3(n=271	14.57162
GSM1108(	G3(n=271	13.31963
GSM1108(	G3(n=271	13.83139
GSM1108(	G3(n=271	14.56338
GSM1108(	G3(n=271	14.0377
GSM1108(	G3(n=271	14.02701
GSM1108(	G3(n=271	14.85148
GSM1108(	G3(n=271	13.24886
GSM1108(	G3(n=271	13.15414
GSM1108(	G3(n=271	12.88206
GSM1108ˉ	G3(n=271	13.4365
GSM1108ˉ	G3(n=271	13.12606
GSM1108ˉ	G3(n=271	14.15298
GSM1108ˉ	G3(n=271	13.28738
GSM1108ˉ	G3(n=271	14.94812
GSM1108ˉ	G3(n=271	13.13111
GSM1108ˉ	G3(n=271	14.33061
GSM1108ˉ	G3(n=271	13.23752
GSM1108ˉ	G3(n=271	13.17709
GSM1108ˉ	G3(n=271	13.85497
GSM1108ˉ	G3(n=271	13.59697
GSM1108ˉ	G3(n=271	13.53718
GSM1108ˉ	G3(n=271	13.50664
GSM1108ˉ	G3(n=271	14.37917
GSM1108ˉ	G3(n=271	14.09401
GSM1108ˉ	G3(n=271	13.28
GSM1108ˉ	G3(n=271	13.75596
GSM1108ˉ	G3(n=271	13.21802
GSM1108ˉ	G3(n=271	14.01273
GSM1108ˉ	G3(n=271	13.40562
GSM1108ˉ	G3(n=271	13.14408
GSM1108ˉ	G3(n=271	14.04285
GSM1108ˉ	G3(n=271	12.51317
GSM1108ˉ	G3(n=271	12.91716
GSM1108ˉ	G3(n=271	13.78538
GSM1108ˉ	G3(n=271	13.41563
GSM1108ˉ	G3(n=271	14.01997
GSM1108ˉ	G3(n=271	14.34422
GSM1108ˉ	G3(n=271	13.80772
GSM1108ˉ	G3(n=271	13.40881
GSM1108{	G3(n=271	14.2615
GSM1108{	G3(n=271	13.43062
GSM1108{	G3(n=271	13.73879
GSM1108{	G3(n=271	13.096
GSM1108{	G3(n=271	13.52495
GSM1108{	G3(n=271	13.68662
GSM1108{	G3(n=271	14.68518
GSM1108{	G3(n=271	12.92431
GSM1108{	G3(n=271	13.84089
GSM1108{	G3(n=271	13.0364
GSM1108{	G3(n=271	13.8298
GSM1108{	G3(n=271	13.52248
GSM1108{	G3(n=271	13.76902
GSM1108{	G3(n=271	13.9568
GSM1205(	G3(n=233	14.32338
GSM1205(	G3(n=233	13.48917
GSM1205(	G3(n=233	14.00568
GSM1205(	G3(n=233	12.38113
GSM1205(	G3(n=233	12.75023
GSM1205(	G3(n=233	13.93589
GSM1205(	G3(n=233	13.42964
GSM1205(	G3(n=233	14.12258
GSM1205(	G3(n=233	13.23665
GSM1205(	G3(n=233	12.80865
GSM1205(	G3(n=233	14.64605
GSM1205(	G3(n=233	12.9621
GSM1205(	G3(n=233	12.89911
GSM1205(	G3(n=233	13.18058
GSM1205(	G3(n=233	13.89461
GSM1205(	G3(n=233	13.01274
GSM1205(	G3(n=233	12.86157
GSM1205(	G3(n=233	13.13123
GSM1205(	G3(n=233	12.01775
GSM1205(	G3(n=233	13.68317
GSM1205(	G3(n=233	13.65868
GSM1205(	G3(n=233	14.27512
GSM1205(	G3(n=233	12.99053
GSM1205(	G3(n=233	13.29788
GSM1205(	G3(n=233	14.18078
GSM1205(	G3(n=233	13.8008
GSM1205(	G3(n=233	14.32243
GSM1205(	G3(n=233	13.70265
GSM1205(	G3(n=233	14.32338
GSM1205(	G3(n=233	13.99283
GSM1205(	G3(n=233	12.9485
GSM1205(	G3(n=233	14.32937
GSM1205(	G3(n=233	13.75807
GSM1205(	G3(n=233	13.55707
GSM1205(	G3(n=233	13.85784
GSM1205(	G3(n=233	12.63952
GSM1205(	G3(n=233	14.64605
GSM1205(	G3(n=233	14.09658
GSM1205(	G3(n=233	13.41957
GSM1205(	G3(n=233	13.40276
GSM1205(	G3(n=233	13.29127
GSM1205(	G3(n=233	14.15335
GSM1205(	G3(n=233	14.29421
GSM1205(	G3(n=233	13.9565
GSM1205(	G3(n=233	13.41896
GSM1205(	G3(n=233	13.73428
GSM1205(	G3(n=233	14.16348
GSM1205(	G3(n=233	13.37788
GSM1205(	G3(n=233	13.31861
GSM1205(	G3(n=233	13.08955
GSM1205(	G3(n=233	13.44248
GSM1205(	G3(n=233	14.75632
GSM1205ˉ	G3(n=233	12.83828
GSM1205ˉ	G3(n=233	13.14422
GSM1205ˉ	G3(n=233	13.69949
GSM1205ˉ	G3(n=233	12.42005
GSM1205ˉ	G3(n=233	13.95732
GSM1205ˉ	G3(n=233	12.81052

GSM11088	G3(n=271	13.82539
GSM11088	G3(n=271	13.90246
GSM11088	G3(n=271	13.30027
GSM11088	G3(n=271	14.15908
GSM11088	G3(n=271	13.41224
GSM11088	G3(n=271	13.24886
GSM11088	G3(n=271	14.08262
GSM11088	G3(n=271	13.61512
GSM11088	G3(n=271	13.23752
GSM11088	G3(n=271	13.52745
GSM11088	G3(n=271	14.63257
GSM11088	G3(n=271	13.07486
GSM11088	G3(n=271	13.29808
GSM11088	G3(n=271	13.13015
GSM11088	G3(n=271	13.27882
GSM11088	G3(n=271	13.43995
GSM11088	G3(n=271	13.17915
GSM11088	G3(n=271	13.24165
GSM11088	G3(n=271	13.58146
GSM11088	G3(n=271	14.05361
GSM11089	G3(n=271	12.96858
GSM11089	G3(n=271	12.91895
GSM11089	G3(n=271	14.00934
GSM11089	G3(n=271	13.6459
GSM11089	G3(n=271	13.51022
GSM11089	G3(n=271	13.22398
GSM11089	G3(n=271	12.34835
GSM11089	G3(n=271	12.98735
GSM11089	G3(n=271	13.15317
GSM11089	G3(n=271	13.28633
GSM11089	G3(n=271	13.33781
GSM11089	G3(n=271	13.14192
GSM11089	G3(n=271	13.38326
GSM11089	G3(n=271	13.41344
GSM11089	G3(n=271	13.27243
GSM11089	G3(n=271	12.71379
GSM11089	G3(n=271	11.98439
GSM11089	G3(n=271	12.49073
GSM11089	G3(n=271	11.90529
GSM11089	G3(n=271	12.77763
GSM11089	G3(n=271	12.84126
GSM11089	G3(n=271	13.09981
GSM11089	G3(n=271	14.22538
GSM11089	G3(n=271	13.48561
GSM11089	G3(n=271	14.33061
GSM11089	G3(n=271	14.03931
GSM11089	G3(n=271	13.39868
GSM11089	G3(n=271	14.39106
GSM11089	G3(n=271	14.29521
GSM11089	G3(n=271	13.91676
GSM11089	G3(n=271	13.80925
GSM11089	G3(n=271	12.75626
GSM11089	G3(n=271	14.89403
GSM11089	G3(n=271	14.68518
GSM11089	G3(n=271	14.16121
GSM11089	G3(n=271	13.50776
GSM11089	G3(n=271	13.49784
GSM11089	G3(n=271	13.3611

GSM12057	G3(n=233	13.05129
GSM12057	G3(n=233	13.04958
GSM12057	G3(n=233	12.70642
GSM12057	G3(n=233	13.5325
GSM12057	G3(n=233	13.81257
GSM12057	G3(n=233	13.49037
GSM12057	G3(n=233	13.26455
GSM12057	G3(n=233	13.14082
GSM12057	G3(n=233	13.89646
GSM12057	G3(n=233	13.14579

GSM1108!	G3(n=271	14.22538
GSM1108!	G3(n=271	14.34648
GSM1108!	G3(n=271	12.95601
GSM1108!	G3(n=271	14.02512
GSM1108!	G3(n=271	13.75023
GSM1108!	G3(n=271	15.36926
GSM1108!	G3(n=271	13.02899
GSM1108!	G3(n=271	14.07886
GSM1108!	G3(n=271	13.28841
GSM1108!	G3(n=271	13.84252
GSM1108!	G3(n=271	13.97349
GSM1108!	G3(n=271	15.06362
GSM1108!	G3(n=271	13.57747
GSM1108!	G3(n=271	13.24886
GSM1108!	G3(n=271	13.8949
GSM1108!	G3(n=271	13.5918
GSM1108!	G3(n=271	14.77148
GSM1108!	G3(n=271	14.38606
GSM1108!	G3(n=271	13.82389
GSM1108!	G3(n=271	14.07713
GSM1108!	G3(n=271	13.73879
GSM1109(	G3(n=271	14.05537
GSM1109(	G3(n=271	13.05886
GSM1109(	G3(n=271	13.9971
GSM1109(	G3(n=271	13.81632
GSM1109(	G3(n=271	13.65403
GSM1109(	G3(n=271	14.41393
GSM1109(	G3(n=271	14.10159
GSM1109(	G3(n=271	13.89178
GSM1109(	G3(n=271	14.56338
GSM1109(	G3(n=271	13.24785
GSM1109(	G3(n=271	14.57678
GSM1109(	G3(n=271	14.38606
GSM1109(	G3(n=271	13.84089
GSM1109(	G3(n=271	13.52495
GSM1109(	G3(n=271	13.3611
GSM1109(	G3(n=271	13.87239
GSM1109(	G3(n=271	14.26973
GSM1109(	G3(n=271	12.98999
GSM1109(	G3(n=271	12.80895

Supplementary Table 9

Genes	GSM1108	GSM1108	GSM1108	GSM1108	GSM1108	GSM1108	GSM1108	GSM1108
METTL14	10.88683	11.37748	11.56596	11.65323	11.36871	11.01975	11.02882	10.93507
YWHAH	14.46432	14.45192	13.38725	13.6972	13.06268	14.19212	14.84131	14.39921

GSM1108	GSM1108	GSM1108	GSM1108	GSM1108	GSM1108	GSM1108	GSM1108	GSM1108
11.18782	10.92972	10.68483	11.81629	11.21791	11.40163	11.57672	11.45421	11.27436
15.2584	14.72948	13.9137	14.00794	13.82665	14.42494	14.25796	14.22207	14.25555

GSM1108!	GSM1108!	GSM1108!	GSM1108!	GSM1108!	GSM1108!	GSM1108!	GSM1108!	GSM1108!
11.72647	11.09494	11.70289	11.40073	11.12059	10.91367	11.16431	11.33961	10.98529
14.21379	14.21979	13.89187	13.65815	14.97169	12.87565	14.09351	13.85383	14.78902

GSM1108[illegible]	GSM1108[illegible]	GSM1108[illegible]	GSM1108[illegible]	GSM1108[illegible]	GSM1108[illegible]	GSM1108[illegible]	GSM1108[illegible]	GSM1108[illegible]
11.1962	11.06617	11.35334	11.39886	10.90615	11.00561	11.13305	11.303	11.09135
14.10148	14.13056	14.69314	14.37204	14.18795	13.77918	13.96875	14.17949	14.46197

GSM1108[illegible]	GSM1108[illegible]	GSM1108[illegible]	GSM1108[illegible]	GSM1108[illegible]	GSM1108[illegible]	GSM1108[illegible]	GSM1108[illegible]	GSM1108[illegible]
11.35104	11.30932	11.7019	10.57513	10.92336	11.03372	11.34002	11.03246	10.92413
13.7659	14.08384	14.8322	14.64361	14.2072	13.97573	13.87724	13.66975	14.53406

GSM1108	GSM1108	GSM1108	GSM1108	GSM1108	GSM1108	GSM1108	GSM1108	GSM11086
11.06885	11.31874	10.88088	11.75395	10.89071	11.57875	11.0587	10.78577	11.72386
14.27855	14.54409	14.08003	14.22609	14.54198	13.27643	14.20041	14.28928	14.66275

GSM1108(	GSM1108(	GSM1108(	GSM1108(	GSM1108(	GSM1108(	GSM1108(	GSM1108(	GSM1108
10.48695	11.31727	11.16755	10.93699	10.91287	11.19381	11.38484	11.54186	10.86123
14.9294	14.84506	14.56348	14.43008	13.80675	13.48833	14.19858	13.95089	14.24508

GSM1108	GSM1108	GSM1108	GSM1108	GSM1108	GSM1108	GSM1108	GSM1108	GSM1108
11.12819	11.19015	11.16755	10.88643	10.88125	10.84312	11.13623	11.11925	10.61288
14.68745	14.5838	13.91876	14.52275	14.33869	14.69314	13.31141	13.9137	13.39212

GSM1108	GSM1108	GSM1108	GSM1108	GSM1108	GSM1108	GSM1108	GSM1108	GSM1108
10.95736	10.81068	10.94211	11.25809	11.43849	11.65893	10.83342	10.66696	10.98163
14.92627	14.78594	15.13144	14.23485	13.83279	14.27165	14.66817	14.84848	14.17949

GSM1108	GSM1108	GSM1108	GSM1108	GSM1108	GSM1108	GSM1108	GSM1108	GSM1108
10.97952	11.64988	11.032	11.30494	10.7449	11.27436	11.05228	10.79083	10.87908
14.32042	14.38995	13.55384	13.40598	14.73236	14.47767	13.53491	14.36342	14.23082

GSM1108	GSM1108	GSM1108	GSM1108	GSM1108	GSM1108	GSM1108	GSM1108	GSM1108
11.10848	11.28683	11.4682	10.74998	11.08647	11.05779	11.12535	10.91367	11.33594
13.6541	13.63594	13.92945	15.15466	14.02077	13.92945	14.53674	14.58113	14.38756

GSM1108(	GSM1108(	GSM1108(	GSM1108(	GSM1108(	GSM1108(	GSM1108(	GSM1108(	GSM1108(
10.57182	11.25993	11.37252	10.96042	10.38406	10.66609	11.30121	11.18377	10.43798
14.41111	14.54409	13.61323	13.85725	14.13238	14.43478	13.30351	14.42241	14.5101

GSM1108	GSM1108	GSM1108	GSM1108	GSM1108	GSM1108	GSM1108	GSM1108	GSM1108
11.97809	10.44408	11.12189	11.25484	10.66291	11.14689	11.32499	11.91278	11.74062
14.05903	14.01503	13.48133	14.02839	14.64112	14.3323	14.22407	14.01889	14.14089

GSM1108	GSM1108	GSM1108	GSM1108	GSM1108	GSM1108	GSM1108	GSM1108	GSM1108
11.48066	11.27626	11.53328	11.22669	11.02191	10.87647	11.05367	11.38574	11.32636
13.1743	13.57896	14.15268	14.44436	15.45039	14.21979	14.30219	14.11361	14.45192

GSM1108[illegible]	GSM1108[illegible]	GSM1108[illegible]	GSM1108[illegible]	GSM1108[illegible]	GSM1108[illegible]	GSM1108[illegible]	GSM1108[illegible]	GSM1108[illegible]
11.52782	11.88006	11.56492	11.81036	11.23742	8.221717	9.941414	11.17745	11.07655
14.07811	13.67131	14.17358	13.2796	14.00036	12.84246	13.23117	14.85907	14.66544

GSM1108	GSM1108	GSM1108	GSM1108	GSM1108	GSM1108	GSM1108	GSM1109	GSM1109
11.21157	11.51726	11.6812	10.84682	11.49191	11.20516	10.42885	11.70139	11.00476
14.89844	13.81342	14.38756	14.75534	13.66243	14.64618	13.98664	13.90848	14.00605

GSM1109(	GSM1109(	GSM1109(	GSM1109(	GSM1109(	GSM1109(	GSM1108⁄	GSM1108⁄	GSM1108⁄
10.75339	11.39984	11.52633	11.2047	11.69366	10.27384	11.23861	11.13816	10.80231
14.2072	14.25796	14.77356	14.52843	13.11777	13.94023	14.80858	13.78877	14.54811

GSM1108	GSM1108	GSM1108	GSM1108	GSM1108	GSM1108	GSM1108	GSM1108	GSM1108
11.25233	11.31138	10.81259	11.40444	10.82951	11.34531	11.47434	11.44444	10.94976
13.947	14.65504	14.84808	14.39312	14.64968	13.89873	14.51202	15.35113	13.7695

GSM1108	GSM1108	GSM1108	GSM1108	GSM1108	GSM1108	GSM1108	GSM1108	GSM1108
11.0976	10.91774	10.54905	10.54177	11.38033	10.84612	10.83508	11.39605	11.27986
14.36983	14.75038	13.7348	14.89346	14.10765	14.2821	14.4347	13.9154	14.11728

GSM1108!	GSM1108!	GSM1108!	GSM1108!	GSM1108!	GSM1108!	GSM1108!	GSM1108!	GSM1108!
10.54627	11.76512	11.76132	10.81999	11.57061	11.21441	11.39504	11.33263	11.5711
14.21821	13.03067	13.39892	14.11914	14.13037	14.42254	14.2384	14.01898	14.14679

GSM11085	GSM11085	GSM11085	GSM11085	GSM11085	GSM11086	GSM11086	GSM11086	GSM11086
11.14192	11.19603	10.96251	11.72475	11.43467	11.065	11.06095	11.20021	11.0029
14.24681	14.00441	14.4822	12.32983	13.6905	14.23224	14.49432	13.63886	14.84201

GSM1108(	GSM1108(	GSM1108(	GSM1108(	GSM1108(	GSM1108(	GSM1108(	GSM1108(	GSM1108(
11.45334	11.02763	11.18489	10.77956	11.17279	11.25141	11.37896	11.32027	11.49089
13.70361	14.18164	14.11159	14.87034	14.13435	14.09636	13.85542	15.11919	13.3467

GSM1108	GSM1108	GSM1108	GSM1108	GSM1108	GSM1108	GSM1108	GSM1108	GSM1108
11.1973	10.89151	10.82649	10.6904	10.97328	10.67067	11.38266	10.85619	11.17104
14.37432	14.47494	14.04918	14.25121	13.75752	14.79925	14.49189	15.20436	14.62833

GSM1108	GSM1108	GSM1108	GSM1108	GSM1108	GSM1108	GSM1108	GSM1108	GSM1108
11.04698	11.17765	11.12407	11.29419	11.39605	11.42272	11.13994	10.37528	11.60877
13.64835	13.88098	14.12075	14.77472	13.69762	13.91367	14.21324	13.96506	12.99038

GSM1108[illegible]	GSM1108[illegible]	GSM1108[illegible]	GSM1108[illegible]	GSM1108[illegible]	GSM1108[illegible]	GSM1108[illegible]	GSM1108[illegible]	GSM1108[illegible]
11.31836	11.22224	10.69794	11.42571	11.15202	11.45139	11.44587	11.66652	11.52348
13.70645	13.05194	14.82991	14.45484	14.34767	13.95391	13.82759	14.25346	14.02039

GSM1108[illegible]	GSM1108[illegible]	GSM1108[illegible]	GSM1108[illegible]	GSM1108[illegible]	GSM1108[illegible]	GSM1108[illegible]	GSM1108[illegible]	GSM1108[illegible]
11.40337	10.78901	11.16729	10.83166	10.82649	10.92114	11.75188	11.72979	11.16045
13.95936	14.00276	13.28675	15.00961	14.29715	14.32735	14.27343	14.2238	13.30123

GSM1108[illegible]	GSM1108[illegible]	GSM1108[illegible]	GSM1108[illegible]	GSM1108[illegible]	GSM1108[illegible]	GSM1108[illegible]	GSM1108[illegible]	GSM1108[illegible]
12.22978	11.13908	11.32576	11.17804	10.94699	8.824183	9.22018	11.70813	11.36657
14.10021	13.94033	14.1136	13.50001	13.55679	13.4745	13.5351	14.63913	14.1136

GSM1108[illegible]	GSM1108[illegible]	GSM1108[illegible]	GSM1108[illegible]	GSM1108[illegible]	GSM1108[illegible]	GSM1108[illegible]	GSM1109[illegible]	GSM1109[illegible]
10.79372	11.47668	12.15319	11.1226	11.98229	11.45445	11.25788	10.09088	10.59652
13.91208	13.41728	14.34537	14.02383	12.53838	14.71216	13.59612	13.94883	14.29943

GSM1109(	GSM1109(	GSM1109(	GSM1109(	GSM1109(	GSM1109(	GSM1109(	GSM1109(	GSM1109(
10.69252	10.15312	10.91475	11.64413	11.16451	11.97836	9.93121	10.5932	10.69124
13.60669	13.23929	15.09939	13.61996	14.60153	15.10333	14.84201	14.56793	13.50344

GSM1109	GSM1108	GSM1108	GSM1108	GSM1108	GSM1108	GSM1108	GSM1108	GSM1108
11.10627	11.59877	11.60915	11.39162	11.63532	11.29211	10.89006	11.46027	11.25011
14.09075	13.6306	14.63627	13.515	14.31144	13.22452	14.614	14.75669	14.72445

GSM1108[illegible]	GSM1108[illegible]	GSM1108[illegible]	GSM1108[illegible]	GSM1108[illegible]	GSM1108[illegible]	GSM1108[illegible]	GSM1108[illegible]	GSM1108[illegible]
11.5723	11.24729	11.67532	11.44335	11.58782	11.93204	10.88788	11.03716	10.93992
13.42912	13.93283	14.50421	14.03908	13.95876	13.6067	14.02968	14.1548	13.76274

GSM1108!	GSM1108!	GSM1108!	GSM1108!	GSM1108!	GSM1108!	GSM1108!	GSM1108!	GSM1108!
10.9023	11.55633	11.03538	11.80163	11.1989	12.09316	11.01811	11.35556	11.62728
13.59486	13.25048	14.29251	13.49894	14.09408	13.61166	14.22334	14.05026	13.17896

GSM1108[illegible]	GSM1108[illegible]	GSM1108[illegible]	GSM1108[illegible]	GSM1108[illegible]	GSM1108[illegible]	GSM1108[illegible]	GSM1108[illegible]	GSM1108[illegible]
11.05566	10.85056	11.21052	11.73942	11.15188	11.40975	11.04873	10.2109	11.13518
14.51144	14.51472	13.86624	13.22877	14.28582	13.754	14.82306	14.67377	13.53133

GSM1108[illegible]	GSM1108[illegible]	GSM1108[illegible]	GSM1108[illegible]	GSM1108[illegible]	GSM1108[illegible]	GSM1108[illegible]	GSM1108[illegible]	GSM1108[illegible]
11.29493	11.8654	11.99739	11.30562	10.92334	11.0302	10.88823	11.1754	11.62682
14.39584	13.68196	13.64122	14.13596	14.35472	14.57202	13.98755	13.22252	13.37632

GSM1108	GSM1108	GSM1108	GSM1108	GSM1108	GSM1108	GSM1108	GSM1108	GSM1108
11.22103	11.85285	11.4178	11.49768	11.2865	11.6188	10.80823	11.90889	11.07199
14.24242	13.46435	13.82294	13.9894	15.03878	13.81242	14.75392	13.03391	14.50421

GSM1108[illegible]	GSM1108[illegible]	GSM1108[illegible]	GSM1108[illegible]	GSM1108[illegible]	GSM1108[illegible]	GSM1108[illegible]	GSM1108[illegible]	GSM1108[illegible]
10.95572	10.75957	11.08041	10.62309	11.96717	11.34003	11.45411	11.47373	11.92634
13.84298	14.53431	13.5027	13.77685	13.5697	13.58916	13.3349	14.57202	13.04672

GSM1108[illegible]	GSM1108[illegible]	GSM1108[illegible]	GSM1108[illegible]	GSM1108[illegible]	GSM1108[illegible]	GSM1108[illegible]	GSM1108[illegible]	GSM1108[illegible]
11.4005	11.82938	10.90892	11.98813	11.04345	11.24395	10.00397	9.673136	9.00905
13.49894	13.18	13.21166	12.86737	13.87399	12.89495	12.90991	12.9547	13.03734

GSM11089	GSM11089	GSM11089	GSM11089	GSM11089	GSM11090	GSM11090	GSM11090	GSM11090
11.43766	12.03456	11.31138	11.23828	11.94786	11.45411	11.29493	10.10851	9.541605
13.45726	13.23721	15.06222	15.14458	14.64183	14.04831	14.34721	13.701	13.45372

GSM1109(	GSM1109(	GSM1109(	GSM1108ʻ	GSM1108ʻ	GSM1108ʻ	GSM1108ʻ	GSM1108ʻ	GSM1108ʻ
11.41885	11.13157	10.21574	11.94028	12.0502	11.94028	11.11665	10.86374	11.7591
14.60853	14.25801	14.04487	13.90718	13.2992	14.00451	14.61623	13.64333	13.21494

GSM1108	GSM1108	GSM1108	GSM1108	GSM1108	GSM1108	GSM1108	GSM1108	GSM1108
11.49214	11.3996	11.14912	12.21143	11.8209	11.46379	11.71655	11.61329	11.88736
13.99896	13.75316	14.38606	13.88532	13.45278	14.30209	14.48391	13.45978	14.1433

GSM1108	GSM1108	GSM1108	GSM1108	GSM1108	GSM1108	GSM1108	GSM1108	GSM1108
11.13965	11.63952	11.92877	11.98206	11.1832	11.27192	11.63165	11.817	11.22075
14.32591	14.14523	13.9971	13.67458	13.62587	13.0477	13.71046	14.17504	13.70187

GSM1108⁴	GSM1108⁴	GSM1108⁴	GSM1108⁴	GSM1108⁴	GSM1108⁴	GSM1108⁴	GSM1108⁴	GSM1108⁴
11.62737	11.4618	11.43279	11.60071	11.48188	11.54973	11.28131	11.32378	11.81181
13.75023	14.77451	13.52622	13.89178	13.42021	13.66076	13.46882	13.9056	13.49901

GSM1108	GSM1108	GSM1108	GSM1108	GSM1108	GSM1108	GSM1108	GSM1108	GSM1108
11.72839	11.63843	10.99012	11.50535	11.8521	11.28412	11.76338	11.20205	11.04027
14.29108	13.77506	13.24785	13.98051	13.67972	13.35771	14.01459	13.81923	12.96691

GSM1108	GSM1108	GSM1108	GSM1108	GSM1108	GSM1108	GSM1108	GSM1108	GSM1108
11.45222	11.4701	11.44637	11.38137	11.14778	11.51918	10.98002	11.15561	11.83719
13.07685	12.30383	14.08837	14.13568	13.32403	14.15298	14.00774	13.81923	13.89776

GSM1108!	GSM1108!	GSM1108!	GSM1108!	GSM1108!	GSM1108!	GSM1108!	GSM1108!	GSM1108!
11.91663	11.59756	10.99789	11.77432	11.79967	11.31808	11.72518	11.57011	12.01996
13.25611	13.96163	13.92939	13.56606	13.69924	13.71879	13.52364	13.63638	13.52364

GSM1108!	GSM1108!	GSM1108!	GSM1108!	GSM1108!	GSM1108!	GSM1108!	GSM1108!	GSM1108!
11.81523	11.49409	11.6159	11.42834	11.00012	11.10334	11.01477	11.03444	10.44159
13.38114	12.79514	13.26699	12.96503	13.68106	13.82539	13.98051	13.65948	12.84721

GSM1108!	GSM1108!	GSM1108!	GSM1108!	GSM1108!	GSM1108!	GSM1108!	GSM1108!	GSM1108!
11.4785	11.59756	10.74037	11.34625	11.11489	11.24071	11.10699	11.3866	11.3866
13.08346	13.18412	13.71729	13.06065	13.76764	13.55481	14.00617	13.57747	13.83619

GSM1108!	GSM1108!	GSM1108!	GSM1108!	GSM1108!	GSM1108!	GSM1108!	GSM1108!	GSM1108!
11.5675	11.06381	11.2546	11.48872	11.64369	11.85669	11.36082	11.35755	11.62737
13.20061	14.59738	13.14713	13.61649	13.79442	13.91514	13.36347	13.8108	13.74748

GSM1108[illegible]	GSM1108[illegible]	GSM1108[illegible]	GSM1108[illegible]	GSM1108[illegible]	GSM1108[illegible]	GSM1108[illegible]	GSM1108[illegible]	GSM1108[illegible]
11.51818	11.69738	11.39476	11.25653	12.05622	11.9785	11.69104	11.57863	11.32662
13.30251	13.71729	13.64862	13.0796	13.29808	13.51137	13.25611	13.45278	13.21088

GSM1108(	GSM1108(	GSM1108(	GSM1108(	GSM1108(	GSM1108(	GSM1108(	GSM1108(	GSM1108(
11.63952	11.60124	11.84746	11.12995	11.59441	11.27288	11.36898	11.26868	11.0135
13.96163	13.4365	13.51883	14.10352	13.06443	14.5526	14.05168	13.49784	14.96512

GSM1108(	GSM1108(	GSM1108(	GSM1108(	GSM1108(	GSM1108(	GSM1108(	GSM1108(	GSM1108(
11.5696	11.49925	12.09399	11.33146	11.28224	11.78515	11.64159	11.95654	11.41067
13.41563	13.38657	13.5139	14.54517	13.63638	13.9971	13.37447	12.83615	14.41609

GSM1108(	GSM1108(	GSM1108(	GSM1108(	GSM1108(	GSM1108(	GSM1108(	GSM1108(	GSM1108(
12.32805	11.01027	11.20387	11.79912	11.86337	10.73121	11.49769	11.99965	11.03571
13.73449	13.57617	13.69924	14.16497	13.71729	14.57162	13.31963	13.83139	14.56338

GSM1108	GSM1108	GSM1108	GSM1108	GSM1108	GSM1108	GSM1108	GSM1108	GSM1108
10.99962	11.27102	11.20474	11.82199	10.99612	11.13965	11.21398	12.77192	11.62002
14.0377	14.02701	14.85148	13.24886	13.15414	12.88206	13.4365	13.12606	14.15298

GSM1108	GSM1108	GSM1108	GSM1108	GSM1108	GSM1108	GSM1108	GSM1108	GSM1108
11.60494	10.75789	11.80134	11.20474	11.28551	11.72355	11.51672	10.90272	11.39282
13.28738	14.94812	13.13111	14.33061	13.23752	13.17709	13.85497	13.59697	13.53718

GSM1108	GSM1108	GSM1108	GSM1108	GSM1108	GSM1108	GSM1108	GSM1108	GSM1108
11.6747	11.09447	11.46128	11.56063	10.87573	11.52018	11.87016	10.23136	11.75306
13.50664	14.37917	14.09401	13.28	13.75596	13.21802	14.01273	13.40562	13.14408

GSM1108	GSM1108	GSM1108	GSM1108	GSM1108	GSM1108	GSM1108	GSM1108	GSM1108
11.27528	11.45311	11.49613	11.83999	11.3996	10.85053	11.23117	10.32436	11.34483
14.04285	12.51317	12.91716	13.78538	13.41563	14.01997	14.34422	13.80772	13.40881

GSM1108(	GSM1108(	GSM1108(	GSM1108(	GSM1108(	GSM1108(	GSM1108(	GSM1108(	GSM1108(
11.66992	11.53485	11.51274	11.16025	11.78966	11.31238	10.95499	11.96305	10.6997
14.2615	13.43062	13.73879	13.096	13.52495	13.68662	14.68518	12.92431	13.84089

GSM1108	GSM1108	GSM1108	GSM1108	GSM1108	GSM1108	GSM1108	GSM1108	GSM1108
11.08521	11.37993	10.95756	11.2919	11.74599	11.20161	11.19146	11.64724	10.87145
13.0364	13.8298	13.52248	13.76902	13.9568	13.82539	13.90246	13.30027	14.15908

GSM1108	GSM1108	GSM1108	GSM1108	GSM1108	GSM1108	GSM1108	GSM1108	GSM1108
10.76882	11.34483	11.90122	11.88506	11.47654	11.56453	11.71226	12.03738	11.75149
13.41224	13.24886	14.08262	13.61512	13.23752	13.52745	14.63257	13.07486	13.29808

GSM1108[illegible]	GSM1108[illegible]	GSM1108[illegible]	GSM1108[illegible]	GSM1108[illegible]	GSM1108[illegible]	GSM1108[illegible]	GSM1108[illegible]	GSM1108[illegible]
11.83445	12.01646	11.27054	11.53835	12.05993	10.95756	11.7208	12.18562	11.19612
13.13015	13.27882	13.43995	13.17915	13.24165	13.58146	14.05361	12.96858	12.91895

GSM1108[illegible]	GSM1108[illegible]	GSM1108[illegible]	GSM1108[illegible]	GSM1108[illegible]	GSM1108[illegible]	GSM1108[illegible]	GSM1108[illegible]	GSM1108[illegible]
11.41828	11.55068	11.62996	11.84289	11.45802	11.68788	11.61847	11.73529	11.00804
14.00934	13.6459	13.51022	13.22398	12.34835	12.98735	13.15317	13.28633	13.33781

GSM11089	GSM11089	GSM11089	GSM11089	GSM11089	GSM11089	GSM11089	GSM11089	GSM11089
10.70992	11.93114	11.58632	11.98902	9.168611	8.715668	8.549839	8.44013	8.782311
13.14192	13.38326	13.41344	13.27243	12.71379	11.98439	12.49073	11.90529	12.77763

GSM11089	GSM11089	GSM11089	GSM11089	GSM11089	GSM11089	GSM11089	GSM11089	GSM11089
8.272711	8.822164	11.29521	11.60699	11.40869	10.88803	11.86461	11.00458	10.82551
12.84126	13.09981	14.22538	13.48561	14.33061	14.03931	13.39868	14.39106	14.29521

GSM1108!	GSM1108!	GSM1108!	GSM1108!	GSM1108!	GSM1108!	GSM1108!	GSM1108!	GSM1108!
11.35419	11.48474	11.61641	11.47996	11.14189	11.5443	12.34569	11.75525	11.75692
13.91676	13.80925	12.75626	14.89403	14.68518	14.16121	13.50776	13.49784	13.3611

GSM1108	GSM1108	GSM1108	GSM1108	GSM1108	GSM1108	GSM1108	GSM1108	GSM1108
11.68371	11.46278	11.34048	11.20161	10.84751	11.03796	11.13965	11.71976	11.89663
14.22538	14.34648	12.95601	14.02512	13.75023	15.36926	13.02899	14.07886	13.28841

GSM1108!	GSM1108!	GSM1108!	GSM1108!	GSM1108!	GSM1108!	GSM1108!	GSM1108!	GSM1108!
12.61567	11.79572	11.38085	11.51432	11.78249	12.3199	11.15001	11.27528	11.61222
13.84252	13.97349	15.06362	13.57747	13.24886	13.8949	13.5918	14.77148	14.38606

GSM1108!	GSM1108!	GSM1108!	GSM1109(	GSM1109(	GSM1109(	GSM1109(	GSM1109(	GSM1109(
11.53537	11.66992	11.58473	11.16785	11.71815	11.20115	11.07143	11.50725	11.87355
13.82389	14.07713	13.73879	14.05537	13.05886	13.9971	13.81632	13.65403	14.41393

GSM1109(	GSM1109(	GSM1109(	GSM1109(	GSM1109(	GSM1109(	GSM1109(	GSM1109(	GSM1109(
11.51578	10.18693	11.14727	10.45065	11.03571	11.88797	11.47213	11.2897	11.62267
14.10159	13.89178	14.56338	13.24785	14.57678	14.38606	13.84089	13.52495	13.3611

GSM1109(	GSM1109(	GSM1109(	GSM1109048
10.32598	11.61901	11.52983	12.05749
13.87239	14.26973	12.98999	12.80895

Supplementary Table 19

Genes	GSM1205:	GSM1205:	GSM1205:	GSM1205:	GSM1205:	GSM1205:	GSM1205:	GSM1205:
YWHAH	14.30853	14.50889	13.47687	14.96758	14.83379	15.1751	14.31722	14.75037
YTHDF1	11.54751	11.47375	11.33203	10.75247	11.06305	11.11374	11.2027	11.21758

GSM1205:	GSM1205:	GSM1205:	GSM1205:	GSM1205:	GSM1205:	GSM1205:	GSM1205:	GSM1205:
14.63144	13.90918	14.34382	14.73118	14.89565	14.26254	14.38383	14.45969	13.50969
11.76382	11.36973	11.26167	11.2037	11.20181	10.97254	11.29488	10.98178	11.30347

GSM1205:	GSM1205:	GSM1205:	GSM1205:	GSM1205:	GSM1205:	GSM1205:	GSM1205:	GSM1205:
13.64541	14.59278	14.43895	14.30648	13.98949	13.91067	13.8394	14.63986	14.75365
11.42637	10.93044	11.14105	11.29848	11.31694	11.73078	11.63697	11.09965	11.1049

GSM1205[illegible]	GSM1205[illegible]	GSM1205[illegible]	GSM1205[illegible]	GSM1205[illegible]	GSM1205[illegible]	GSM1205[illegible]	GSM1205[illegible]	GSM1205[illegible]
13.99442	13.57215	14.54147	14.16061	13.62218	14.79133	14.29069	14.2145	14.74892
11.33888	11.5161	10.93971	11.48894	11.4973	11.20663	11.4129	11.44633	10.62618

GSM1205:	GSM1205:	GSM1205:	GSM1205:	GSM1205:	GSM1205:	GSM1205:	GSM1205:	GSM1205:
14.17876	13.94912	14.43569	14.26381	14.80036	13.86907	14.05519	14.53396	14.3976
11.05696	10.84119	11.18869	11.38845	10.74564	11.26919	10.91954	11.11352	11.29758

GSM1205[illegible]	GSM1205[illegible]	GSM1205[illegible]	GSM1205[illegible]	GSM1205[illegible]	GSM1205[illegible]	GSM1205[illegible]	GSM1205[illegible]	GSM1205[illegible]
14.60499	14.8928	14.35882	14.84107	13.43511	14.24796	14.60197	14.16331	14.30195
11.16289	11.59539	11.61522	10.89958	11.36105	11.28409	11.53635	11.55312	11.54887

GSM1205[illegible]	GSM1205[illegible]	GSM1205[illegible]	GSM1205[illegible]	GSM1205[illegible]	GSM1205[illegible]	GSM1205[illegible]	GSM1205[illegible]	GSM1205[illegible]
14.99844	14.61503	14.27633	14.21262	14.10372	15.29065	15.48077	14.4161	14.50339
11.11732	11.33857	11.06853	11.24847	11.47805	11.37771	11.52442	11.38605	11.06923

GSM1205⁴	GSM1205⁴	GSM1205⁴	GSM1205⁴	GSM1205⁴	GSM1205⁴	GSM1205⁴	GSM1205⁴	GSM1205⁴
14.49489	14.57636	13.6687	13.48721	14.29482	13.39003	13.37631	13.99856	14.09398
11.26655	11.3098	11.49665	11.19874	11.56795	11.54478	11.70054	11.28239	11.41826

GSM1205‹	GSM1205‹	GSM1205‹	GSM1205‹	GSM1205‹	GSM1205‹	GSM1205‹	GSM1205‹	GSM1205‹
14.26014	14.23433	14.29886	14.20805	13.29337	14.47223	13.91376	14.09697	14.89565
11.59317	11.56292	11.31886	11.38557	11.51541	11.48849	11.23963	11.50197	11.29167

GSM1205	GSM1205	GSM1205	GSM1205	GSM1205	GSM1205	GSM1205	GSM1205	GSM1205
14.52226	14.49043	14.09244	14.19318	14.31822	14.37823	14.33023	13.56525	13.7927
11.49712	11.47805	11.54212	11.28674	12.11381	11.38787	11.79699	11.64566	10.73513

GSM1205[illegible]	GSM1205[illegible]	GSM1205[illegible]	GSM1205[illegible]	GSM1205[illegible]	GSM1205[illegible]	GSM1205[illegible]	GSM1205[illegible]	GSM1205[illegible]
14.50224	13.74101	14.30438	14.13839	14.52566	14.07214	13.40843	14.17112	13.97602
11.1742	11.4379	11.68506	11.28573	11.8662	11.34429	11.5077	11.34662	11.55827

GSM12055	GSM12055	GSM12055	GSM12056	GSM12056	GSM12056	GSM12056	GSM12056	GSM12056
14.29972	14.32914	14.35978	14.17305	14.27094	13.76311	14.10642	14.92128	14.47023
11.63306	11.46459	11.2037	11.5235	11.32293	11.58224	11.53523	12.10531	11.57863

GSM1205	GSM1205	GSM1205	GSM1205	GSM1205	GSM1205	GSM1205	GSM1205	GSM1205
13.78604	14.46385	14.64245	13.77492	13.94524	14.43895	13.57106	14.8928	14.69244
11.8256	11.38021	11.34624	11.14569	11.45157	11.61445	11.3458	11.43258	11.02634

GSM1205	GSM1205	GSM1205	GSM1205	GSM1205	GSM1205	GSM1205	GSM1205	GSM1205
14.27827	14.05519	13.97313	13.76106	14.18581	14.42114	15.14548	14.14897	14.54804
11.51994	11.70017	11.23398	11.48059	11.20828	11.398	10.78449	11.61	11.32353

GSM1205[illegible]	GSM1205[illegible]	GSM1205[illegible]	GSM1205[illegible]	GSM1205[illegible]	GSM1205[illegible]	GSM1205[illegible]	GSM1205[illegible]	GSM1205[illegible]
13.40928	13.7963	14.53887	15.23364	14.67428	13.70243	13.93611	14.15418	14.81431
11.16445	11.53516	11.40888	10.96819	11.13717	11.81489	11.06715	11.49081	11.24284

GSM12052	GSM12052	GSM12052	GSM12052	GSM12053	GSM12053	GSM12053	GSM12053	GSM12053
14.11286	13.90769	14.30531	13.84187	14.84304	13.73267	13.95747	14.27042	14.46499
11.12556	10.98964	11.23002	11.2556	10.94752	11.48126	11.32431	11.58608	11.16052

GSM1205	GSM1205	GSM1205	GSM1205	GSM1205	GSM1205	GSM1205	GSM1205	GSM1205
14.29178	14.79164	14.0713	14.1955	14.30297	14.0647	12.40919	14.53674	13.7573
11.12351	11.08533	10.90762	11.46329	11.35493	10.86066	11.18464	11.31052	11.23563

GSM1205[illegible]	GSM1205[illegible]	GSM1205[illegible]	GSM1205[illegible]	GSM1205[illegible]	GSM1205[illegible]	GSM1205[illegible]	GSM1205[illegible]	GSM1205[illegible]
14.2872	14.54088	13.70836	14.23176	14.88167	13.75293	13.36166	14.18163	14.90691
11.21791	11.30137	11.49251	11.22435	11.1062	11.87029	11.25152	11.2255	11.06292

GSM1205	GSM1205	GSM1205	GSM1205	GSM1205	GSM1205	GSM1205	GSM1205	GSM1205
14.41255	14.19935	13.36267	14.69653	14.61458	14.38162	14.56202	14.31998	15.36284
11.22076	11.53307	11.66039	11.36961	11.29464	11.2813	11.46059	11.45846	11.15979

GSM1205	GSM1205	GSM1205	GSM1205	GSM1205	GSM1205	GSM1205	GSM1205	GSM1205
14.00369	14.1921	14.69653	14.34599	14.06294	13.62127	13.99238	13.56561	13.76293
11.37022	11.33203	10.86257	11.34193	11.38953	11.32521	11.05729	11.05446	10.73217

GSM12054	GSM12054	GSM12054	GSM12054	GSM12054	GSM12055	GSM12055	GSM12055	GSM12055
14.15189	14.16428	14.92301	14.16165	14.28967	13.84951	15.03678	13.09937	14.30297
11.80093	11.58888	11.07561	11.27068	11.67857	11.26853	11.19642	11.70456	11.46677

GSM1205	GSM1205	GSM1205	GSM1205	GSM1205	GSM1205	GSM1205	GSM1205	GSM1205
14.17401	13.95825	13.04896	15.01347	14.70969	14.17282	13.96345	13.49193	12.34865
11.30149	11.31267	11.27316	11.72693	11.62543	11.32617	11.35349	11.36911	11.94805

GSM1205	GSM1205	GSM1205	GSM1205	GSM1205	GSM1205	GSM1205	GSM1205	GSM1205
13.5608	13.426	13.58118	14.4798	14.65273	13.49758	13.61001	14.11341	14.32949
11.41518	11.54062	11.47663	11.07582	10.70382	11.43958	11.1079	10.923	10.85423

GSM1205	GSM1205	GSM1205	GSM1205	GSM1205	GSM1205	GSM1205	GSM1205	GSM1205
14.21266	13.78727	14.73364	13.81061	14.37911	13.95778	15.02364	13.33423	12.98878
11.14747	11.4832	11.09626	11.08159	10.93652	11.3955	11.24299	11.52197	11.59108

GSM1205:	GSM1205:	GSM1205:	GSM1205:	GSM1205:	GSM1205:	GSM1205:	GSM1205:	GSM1205:
14.27405	14.07215	14.04616	14.49441	14.48461	14.25566	13.85284	13.72372	13.20772
10.61796	11.17229	11.47122	11.20912	11.07438	11.44444	10.97278	10.64105	11.50992

GSM1205:	GSM1205:	GSM1205:	GSM1205:	GSM1205:	GSM1205:	GSM1205	GSM1205	GSM1205
13.15704	13.45996	14.54977	13.96453	14.67992	13.17601	13.30873	14.7138	13.0222
11.31584	11.58647	11.38431	11.00743	11.13962	10.73652	10.87294	11.66475	11.19176

GSM1205	GSM1205	GSM1205	GSM1205	GSM1205	GSM1205	GSM1205	GSM1205	GSM1205
14.55208	13.91479	14.62607	13.4925	14.29453	13.85777	13.40091	13.04984	13.58034
11.08582	11.09416	11.14443	11.7881	11.18808	11.20877	11.68594	11.59725	11.65313

GSM1205[illegible]	GSM1205[illegible]	GSM1205[illegible]	GSM1205[illegible]	GSM1205[illegible]	GSM1205[illegible]	GSM1205[illegible]	GSM1205[illegible]	GSM1205[illegible]
13.53062	14.43473	13.84071	13.56608	13.19108	13.47371	13.75462	12.85067	13.15246
11.6343	11.22456	10.99362	11.78116	11.21351	11.54129	11.60825	11.6141	11.4787

GSM12055	GSM12055	GSM12055	GSM12055	GSM12056	GSM12057	GSM12057	GSM12057	GSM12057
13.73624	14.14303	13.56262	14.4798	12.89184	14.3364	15.3845	14.12355	12.3402
11.52919	11.48983	11.40323	10.98678	11.7665	11.54507	11.39921	11.74087	11.5379

GSM1205:	GSM1205:	GSM1205:	GSM1205:	GSM1205:	GSM1205:	GSM1205:	GSM1205:	GSM1205:
13.17697	13.94588	13.19618	13.35477	13.08838	13.63251	13.72399	13.01852	13.24308
11.32669	11.48677	11.38966	11.43093	11.20292	11.17157	11.36045	10.73571	10.3998

GSM1205[illegible]	GSM1205[illegible]	GSM1205[illegible]	GSM1205[illegible]	GSM1205[illegible]	GSM1205[illegible]	GSM1205[illegible]	GSM1205[illegible]	GSM1205[illegible]
13.83598	13.2758	13.41393	14.34023	13.46818	14.08509	13.20054	14.07131	14.31044
11.06038	11.1614	11.82605	10.92953	11.11796	10.91498	11.18703	11.07596	11.23334

GSM1205:	GSM1205:	GSM1205:	GSM1205:	GSM1205:	GSM1205:	GSM1205:	GSM1205:	GSM1205:
13.64879	13.11084	13.43931	13.40215	13.28255	13.93111	13.72339	13.13123	14.53554
11.17755	11.15672	11.11819	10.99752	11.08078	11.12301	10.939	11.23402	11.35637

GSM12053	GSM12054	GSM12054	GSM12054	GSM12054	GSM12054	GSM12054	GSM12054	GSM12054
13.02245	13.42215	12.17253	13.65435	13.20187	13.5612	14.01653	12.5781	13.89127
11.0983	11.34158	11.00859	10.92346	10.37965	10.96064	11.36471	11.60367	10.92996

GSM1205	GSM1205	GSM1205	GSM1205	GSM1205	GSM1205	GSM1205	GSM1205	GSM1205
14.7794	13.84572	13.76754	13.17073	12.96901	14.40887	13.07931	14.47757	13.62846
10.9808	11.1862	11.47549	11.93795	11.39891	10.92811	11.50143	11.25557	11.56165

GSM1205	GSM1205	GSM1205	GSM1205	GSM1205	GSM1205	GSM1205	GSM1205	GSM1205
13.33994	13.80223	13.75197	13.41525	13.61696	12.7984	13.39581	13.16596	13.00922
11.26774	11.68528	11.26151	11.44785	11.8304	11.29143	11.41505	11.14885	11.11022

GSM1205	GSM1205	GSM1205	GSM1205	GSM1205	GSM1205	GSM1205	GSM1205	GSM1205
13.50992	14.25075	13.14422	12.95349	13.01118	13.13601	13.19013	12.94047	13.56796
11.64932	10.96895	11.75046	11.24049	11.30847	11.608	11.53763	11.0327	11.2879

GSM1205[illegible]	GSM1205[illegible]	GSM1205[illegible]	GSM1205[illegible]	GSM1205[illegible]	GSM1205[illegible]	GSM1205[illegible]	GSM1205[illegible]	GSM1205[illegible]
13.61148	13.19117	14.47501	13.53176	13.91454	13.50582	13.98165	13.06293	14.50125
11.3879	11.87012	11.00023	10.85751	11.0137	10.90852	11.1727	11.58125	10.69021

GSM1205!	GSM1205!	GSM1205!	GSM1205!	GSM1205!	GSM1205!	GSM1205!	GSM1205!	GSM1205!
13.94808	14.91083	13.04082	13.66845	13.46456	13.91654	13.75742	13.52333	13.03445
11.1787	10.99078	11.08022	10.84116	11.23009	10.55117	11.14578	11.00792	11.07852

GSM1205[illegible]	GSM1205[illegible]	GSM1205[illegible]	GSM1205[illegible]	GSM1205[illegible]	GSM1205[illegible]	GSM1205[illegible]	GSM1205[illegible]	GSM1205[illegible]
14.01931	12.95645	12.76344	13.13016	14.00422	14.61654	13.61832	13.19355	13.32555
11.04987	11.39801	11.02384	11.44895	10.84903	11.14956	10.99054	11.03055	11.25649

GSM12055	GSM12055	GSM12055	GSM12055	GSM12055	GSM12055	GSM12055	GSM12055	GSM12055
12.93772	14.31913	14.04716	13.73896	13.75197	13.81915	14.23268	14.06768	14.54343
11.04492	10.88627	10.91088	10.97991	11.08874	11.09304	11.23853	10.88149	11.23186

GSM1205!	GSM1205!	GSM1205!	GSM1205!	GSM1205!	GSM1205!	GSM1205!	GSM1205!	GSM1205!
13.65685	13.67519	13.42554	13.72399	12.83357	13.43471	13.79188	13.33871	13.62951
11.25624	11.58264	11.29695	11.09885	10.8619	11.6039	11.406	11.53712	11.55959

GSM12055	GSM12055	GSM12055	GSM12055	GSM12055	GSM12055	GSM12055	GSM12055	GSM12055
13.81576	13.44187	13.18584	13.92563	13.30674	12.94096	14.09367	12.66445	13.16361
10.95012	11.23358	11.56991	11.1291	11.49768	10.64329	11.26122	11.29905	11.35498

GSM1205!	GSM1205!	GSM1205!	GSM1205!	GSM1205!	GSM1205!	GSM1205!	GSM1205!	GSM1205!
13.3731	13.81326	13.41088	13.47395	13.97893	12.83086	13.40884	13.44841	13.7373
11.13775	11.64709	11.50229	11.01851	10.904	11.56246	11.47187	11.41717	11.43665

GSM1205!	GSM1205!	GSM1205!	GSM1205!	GSM1205!	GSM1205!	GSM1205(	GSM1205(	GSM1205(
13.90288	13.53423	13.70696	13.12056	13.01019	12.70513	13.32214	13.7507	13.59606
11.22338	10.97284	11.12688	11.39104	11.6796	11.28225	10.89898	11.25704	11.01141

GSM1205(	GSM1205(	GSM1205(	GSM1205(	GSM1205(	GSM1205(	GSM1205(	GSM1205(	GSM1205(
13.61979	13.17804	13.62484	13.57075	14.21687	13.08139	13.14475	12.79413	13.93779
11.39312	11.2673	11.53661	11.22778	11.3111	11.37078	11.30931	10.85932	11.46601

GSM1205(	GSM1205(	GSM1205(	GSM1205(	GSM1205(	GSM1205(	GSM1205(	GSM1205(	GSM1205(
13.11282	13.56059	13.90448	12.84138	13.51364	12.71389	13.18058	13.10395	14.90553
11.6243	11.33564	11.11071	11.09906	11.52208	11.27728	11.3108	11.04294	11.15017

GSM1205(	GSM1205(	GSM1205(	GSM1205(	GSM1205(	GSM1205(	GSM1205(	GSM1205(	GSM1205(
13.32214	13.019	13.56796	13.89936	13.94122	13.67125	13.68968	13.30946	13.3984
11.19672	11.41685	12.00209	11.13125	11.03916	11.30744	11.1626	11.54423	11.34635

GSM1205(	GSM1205(	GSM1205(	GSM1205(	GSM1205(	GSM1205(	GSM1205(	GSM1205(	GSM1205(
13.96971	13.7682	12.71201	14.32338	13.48917	14.00568	12.38113	12.75023	13.93589
10.95277	10.68622	10.99347	11.10314	11.33131	11.4195	11.19672	11.51694	10.95439

GSM1205(	GSM1205(	GSM1205(	GSM1205(	GSM1205(	GSM1205(	GSM1205(	GSM1205(	GSM12056
13.42964	14.12258	13.23665	12.80865	14.64605	12.9621	12.89911	13.18058	13.89461
11.37591	10.9778	11.56191	11.17807	11.09773	11.3422	11.34451	11.38497	10.35878

GSM1205(	GSM1205(	GSM1205(	GSM1205(	GSM1205(	GSM1205(	GSM1205(	GSM1205(	GSM1205(
13.01274	12.86157	13.13123	12.01775	13.68317	13.65868	14.27512	12.99053	13.29788
11.68528	11.69522	11.33485	10.96425	11.46797	11.4711	11.42523	11.31316	11.46546

GSM1205(	GSM1205(	GSM1205(	GSM1205(	GSM1205(	GSM1205(	GSM1205(	GSM1205(	GSM1205(
14.18078	13.8008	14.32243	13.70265	14.32338	13.99283	12.9485	14.32937	13.75807
10.93622	11.28922	11.68424	11.66267	11.00023	11.48625	11.29578	11.74162	11.5574

GSM1205(	GSM1205(	GSM1205(	GSM1205(	GSM1205(	GSM1205(	GSM1205(	GSM1205(	GSM1205(
13.55707	13.85784	12.63952	14.64605	14.09658	13.41957	13.40276	13.29127	14.15335
11.74568	11.36446	10.56001	11.64111	11.60466	11.55188	12.89195	11.51914	11.56519

GSM1205	GSM1205	GSM1205	GSM1205	GSM1205	GSM1205	GSM1205	GSM1205	GSM1205
14.29421	13.9565	13.41896	13.73428	14.16348	13.37788	13.31861	13.08955	13.44248
11.96014	11.43684	11.82675	11.27854	11.2303	11.50505	11.45476	11.5041	11.35567

GSM1205(	GSM1205	GSM1205	GSM1205	GSM1205	GSM1205	GSM1205	GSM1205	GSM1205
14.75632	12.83828	13.14422	13.69949	12.42005	13.95732	12.81052	13.05129	13.04958
10.61637	10.78729	11.57167	11.12486	11.2306	10.96102	11.21779	11.23792	11.25624

GSM1205	GSM1205	GSM1205	GSM1205	GSM1205	GSM1205	GSM1205	GSM1205729
12.70642	13.5325	13.81257	13.49037	13.26455	13.14082	13.89646	13.14579
11.75206	10.67281	11.13199	10.69271	11.48677	11.15756	11.69372	11.29951

Supplementary Table 20

Depmap II	METTL14 I	YWHAH Ic	Primary Di	Cell Line N	Lineage
ACH-0011	4.464668	5.899176	Non-Smal	LC1SQSF	Lung
ACH-0012	4.97728	6.573344	Embryona	COGAR35	CNS/Brain
ACH-0013	4.257011	6.874428	Melanoma	COLO794	Skin
ACH-0015	4.277985	5.334497	Intraducta	KKU213	Biliary Tract
ACH-0002	3.83289	6.855741	Bladder U	RT4	Bladder/Urinary Tract
ACH-0007	3.121015	6.415826	Colorectal	SNU283	Bowel
ACH-0003	2.493135	5.27538	Non-Smal	NCIH1395	Lung
ACH-0002	4.405312	6.749936	Mature T a	DEL	Lymphoid
ACH-0004	3.407353	6.954778	Intraducta	SNU1196	Biliary Tract
ACH-0007	2.992768	6.096557	Non-Smal	LC1F	Lung
ACH-0017	4.608218	6.889839	Liposarcor	93T449	Soft Tissue
ACH-0020	4.763943	6.620586	Ampullary	TGBC18TK	Ampulla of Vater
ACH-0005	3.827819	5.919102	Non-Smal	ABC1	Lung
ACH-0016	2.83996	6.202418	Uterine Sa	SKN	Uterus
ACH-0001	4.31034	6.377471	Mature B-	KE97	Lymphoid
ACH-0007	3.288359	6.652056	Renal Cell	BFTC909	Kidney
ACH-0010	4.604664	6.355615	Pancreatic	KCIMOH1	Pancreas
ACH-0005	3.970854	5.925287	Diffuse Gli	YKG1	CNS/Brain
ACH-0003	4.642702	6.50462	Esophago	MKN1	Esophagus/Stomach
ACH-0020	4.175525	5.88923	Mucosal M	HMVII	Vulva/Vagina
ACH-0007	3.039138	6.412104	Non-Smal	LK2	Lung
ACH-0001	4.1152	7.242317	Non-Canc	HS888T	Fibroblast
ACH-0008	3.627607	6.60422	Invasive Br	ZR7530	Breast
ACH-0004	4.432291	6.210817	Colorectal	SW837	Bowel
ACH-0007	4.252476	7.339405	Melanoma	WM983B	Skin
ACH-0013	4.727376	6.269594	Pancreatic	PACADD1	Pancreas
ACH-0000	3.295723	6.811856	Melanoma	A101D	Skin
ACH-0018	4.19928	6.900263	Osteosarc	OS252	Bone
ACH-0008	4.144862	6.727512	Lung Neur	NCIH446	Lung
ACH-0006	2.698218	6.908212	Head and	PECAPJ34	Head and Neck
ACH-0003	2.684819	7.124018	Renal Cell	TUHR14TK	Kidney
ACH-0001	3.742006	6.953847	Ovarian Ep	COV434	Ovary/Fallopian Tube
ACH-0007	2.330558	5.569248	Head and	YD10B	Head and Neck
ACH-0020	4.569248	7.269127	Nerve She	HSSCH2	Peripheral Nervous System
ACH-0015	2.961623	6.034744	Intraducta	KKU100	Biliary Tract
ACH-0031	5.294621	7.550208	Non-Canc	ABMT0023	Unknown
ACH-0003	4.667324	7.57107	Renal Cell	KMRC3	Kidney
ACH-0020	4.77926	5.795715	Glassy Cel	HOKUG	Cervix
ACH-0007	4.440288	6.722056	B-Lympho	KASUMI2	Lymphoid
ACH-0007	5.486393	6.796494	Non-Smal	NCIH2882	Lung
ACH-0008	3.339137	6.900142	Esophagea	KYSE140	Esophagus/Stomach
ACH-0006	3.340562	6.530289	Esophago	MKN7	Esophagus/Stomach
ACH-0000	4.434962	5.822475	Acute Mye	MONOMA	Myeloid
ACH-0020	3.915521	5.608514	Head and	SAS	Head and Neck
ACH-0013	4.942984	6.997631	Pancreatic	PACADD1	Pancreas
ACH-0007	3.566815	7.230549	Hodgkin L	L428	Lymphoid
ACH-0004	3.544733	6.984248	Melanoma	SKMEL1	Skin
ACH-0001	3.729009	6.647602	Pancreatic	CFPAC1	Pancreas
ACH-0018	4.135042	4.9241	Intraducta	YSCCC	Biliary Tract
ACH-0003	3.365972	6.794026	Esophago	OE33	Esophagus/Stomach
ACH-0015	4.189825	5.927422	Pleural Me	LO68	Pleura
ACH-0009	3.139142	6.764606	Esophagea	TE4	Esophagus/Stomach
ACH-0024	5.040892	6.170326	Osteosarc	OS052	Bone
ACH-0009	4.66562	7.630959	Endometri	HEC6	Uterus
ACH-0014	4.521679	6.579995	Bladder U	UMUC16	Bladder/Urinary Tract
ACH-0017	2.375735	5.058316	Mature B-	WAOSEL	Lymphoid
ACH-0001	3.173127	6.149341	Invasive Br	EFM192A	Breast
ACH-0009	3.708187	6.69808	Colorectal	SNU1040	Bowel

ACH-0006	3.346248	7.084064	Esophago	FU97	Esophagus/Stomach
ACH-0005	3.168321	6.370687	Ovarian E	59M	Ovary/Fallopian Tube
ACH-0001	3.07382	6.606442	Non-Smal	LOUNH91	Lung
ACH-0000	3.705978	6.288359	Invasive B	SKBR3	Breast
ACH-0000	4.313971	6.793376	B-Lympho	MHHCALL	Lymphoid
ACH-0013	3.042644	6.272583	Head and	RPMI2650	Head and Neck
ACH-0005	3.564378	7.943218	Non-Smal	NCIH1975	Lung
ACH-0007	4.321207	6.250393	Non-Smal	NCIH2126	Lung
ACH-0005	4.330558	5.445594	Invasive B	UACC893	Breast
ACH-0013	3.813525	6.092546	Renal Cell	SW156	Kidney
ACH-0019	4.663914	6.696968	Ovarian E	NZOV9	Ovary/Fallopian Tube
ACH-0013	2.974529	5.400196	Head and	BICR78	Head and Neck
ACH-0005	3.38405	7.593727	Non-Smal	EPLC272H	Lung
ACH-0005	3.365972	6.958611	Melanoma	IGR39	Skin
ACH-0006	4.120186	5.209453	Non-Smal	LCLC103H	Lung
ACH-0007	2.803227	7.370862	Ovarian E	OAW42	Ovary/Fallopian Tube
ACH-0002	4.658783	7.171527	Invasive B	HCC38	Breast
ACH-0000	4.011675	7.29094	Diffuse Gli	U87MG	CNS/Brain
ACH-0008	4.356848	5.647602	Invasive B	BT483	Breast
ACH-0016	2.503349	5.039577	Mature B-	OCILY18	Lymphoid
ACH-0006	4.555816	7.909533	Hepatocel	HEP3B217	Liver
ACH-0009	3.187451	6.606886	Endometri	HEC1B	Uterus
ACH-0003	2.815575	6.689858	Non-Smal	CALU3	Lung
ACH-0002	3.933573	7.030667	Diffuse Gli	AM38	CNS/Brain
ACH-0016	3.491853	6.785681	Embryona	TASK1	CNS/Brain
ACH-0031	4.651913	7.193476	Non-Canc	ABMT002	Unknown
ACH-0008	3.827819	6.448405	Invasive B	CAL51	Breast
ACH-0026	3.047887	5.597233	Ocular Me	WM3772F	Eye
ACH-0011	3.82171	5.5157	Mature T	MAC2A	Lymphoid
ACH-0031	4.887525	7.68629	Non-Canc	ABMT000	Unknown
ACH-0003	3.930737	6.99876	Diffuse Gli	SF295	CNS/Brain
ACH-0006	3.361768	5.782409	Adenosqu	L33	Pancreas
ACH-0007	4.479619	7.646091	Lung Neu	NCIH1963	Lung
ACH-0010	5.166314	6.662348	Ewing Sar	CHLA9	Bone
ACH-0012	4.485427	5.692929	Ewing Sar	TC106	Bone
ACH-0008	4.189034	7.44667	Esophago	AGS	Esophagus/Stomach
ACH-0000	3.654206	6.759023	Non-Smal	NCIH1650	Lung
ACH-0012	3.508429	7.288635	Rhabdoid	STM9101	Kidney
ACH-0001	4.040892	8.202124	Non-Canc	HS934T	Fibroblast
ACH-0003	3.545968	7.165209	Melanoma	HT144	Skin
ACH-0005	3.257011	6.243364	Colorectal	HT29	Bowel
ACH-0025	4.341986	6.790642	Melanoma	WM3211	Skin
ACH-0002	3.182692	6.502076	Pancreatic	ASPC1	Pancreas
ACH-0002	4.968552	6.362996	Neuroblas	SKNAS	Peripheral Nervous System
ACH-0016	3.104337	7.148731	Cervical S	SKGII	Cervix
ACH-0006	2.182692	6.394891	Ovarian E	COV644	Ovary/Fallopian Tube
ACH-0005	2.963474	6.664198	Colorectal	LS123	Bowel
ACH-0006	4.510962	6.607626	Mature T	HUT102	Lymphoid
ACH-0011	4.408032	6.044176	Rhabdom	SCMCRM2	Soft Tissue
ACH-0016	2.035624	5.19101	Renal Cell	UMRC7	Kidney
ACH-0018	3.456806	4.894333	Intraducta	ICC12	Biliary Tract
ACH-0006	4.128458	6.378165	Non-Smal	NCIH441	Lung
ACH-0018	4.44427	6.982081	Intraducta	ICC6	Biliary Tract
ACH-0007	3.438293	6.168722	Colorectal	CL40	Bowel
ACH-0013	4.751142	6.338603	Medullary	TT	Thyroid
ACH-0008	3.047887	8.428988	Non-Smal	NCIH1869	Lung
ACH-0015	4.721919	7.141392	Pleural Me	MERO82	Pleura
ACH-0003	2.950468	5.295723	Esophago	SNU668	Esophagus/Stomach
ACH-0002	3.672425	6.716442	Invasive B	HCC1937	Breast

ACH-0000	3.571677	6.311975	Pancreatic	PANC0213	Pancreas
ACH-0000	3.125982	6.076388	Acute Mye	PLB985	Myeloid
ACH-0018	3.833902	5.279471	Intrachole	TGBC1TKB	Biliary Tract
ACH-0007	3.917432	6.710944	Rhabdomy	TE441T	Soft Tissue
ACH-0009	3.81455	7.470293	Endometri	HEC265	Uterus
ACH-0005	3.949535	6.37417	Non-Smal	LCLC97TM	Lung
ACH-0006	3.82171	6.944273	Melanoma	HS944T	Skin
ACH-0009	3.824768	7.485024	Endometri	EN	Uterus
ACH-0003	4.399171	6.364397	Pancreatic	YAPC	Pancreas
ACH-0018	4.762349	6.697385	Intraducta	ICC15	Biliary Tract
ACH-0001	4.672991	7.680113	Lung Neur	NCIH1618	Lung
ACH-0008	2.618239	6.340206	Diffuse Gli	SF172	CNS/Brain
ACH-0003	3.568032	7.735658	Diffuse Gli	SNU1105	CNS/Brain
ACH-0002	4.235727	7.831371	Non-Canc	HS600T	Fibroblast
ACH-0005	4.239551	7.431874	Non-Smal	HCC1171	Lung
ACH-0016	4.983222	7.6094	Diffuse Gli	NP8	CNS/Brain
ACH-0001	3.969933	7.9331	Non-Canc	HS940T	Fibroblast
ACH-0004	3.675816	5.925525	Acute Mye	U937	Myeloid
ACH-0015	4.159468	5.226894	Mature B-	KMS28PE	Lymphoid
ACH-0009	4.05398	7.23477	Colorectal	CW2	Bowel
ACH-0004	3.647315	5.707635	Melanoma	SH4	Skin
ACH-0009	4.273516	6.737146	B-Lympho	REH	Lymphoid
ACH-0000	3.382667	7.261061	Diffuse Gli	GAMG	CNS/Brain
ACH-0016	2.867896	6.380071	Cervical Sc	SKGI	Cervix
ACH-0014	2.778209	5.888987	Colorectal	C125PM	Bowel
ACH-0013	2.715893	6.040673	Cervical Sc	CASKI	Cervix
ACH-0002	3.906891	7.229107	Non-Canc	HS840T	Fibroblast
ACH-0009	4.172327	7.474193	Colorectal	HCT116	Bowel
ACH-0014	3.956986	6.845615	Colorectal	C10	Bowel
ACH-0009	3.051372	6.052242	Colorectal	HT55	Bowel
ACH-0000	3.795975	6.657211	Non-Smal	NCIH1819	Lung
ACH-0005	3.313246	5.071248	Mature B-	KMS27	Lymphoid
ACH-0000	3.963474	5.818902	Invasive Br	MCF7	Breast
ACH-0015	2.641546	6.208868	Acute Mye	MUTZ8	Myeloid
ACH-0003	3.759156	5.405652	Invasive Br	EFM19	Breast
ACH-0008	3.77926	7.519322	Hepatocel	JHH7	Liver
ACH-0004	3.571677	6.594549	Non-Smal	NCIH2170	Lung
ACH-0015	1.895303	6.758356	Endometri	HEC1	Uterus
ACH-0003	4.065228	6.670585	Mature T a	SR786	Lymphoid
ACH-0019	4.224966	6.889595	Intraducta	CCLP1	Biliary Tract
ACH-0014	4.505256	5.182692	Bladder Ur	UMUC6	Bladder/Urinary Tract
ACH-0008	3.725741	6.585714	Lung Neur	NCIH510	Lung
ACH-0014	4.181898	6.79012	Ovarian Ep	TO14	Ovary/Fallopian Tube
ACH-0008	3.395063	5.730368	Melanoma	COLO679	Skin
ACH-0011	4.139961	6.552131	Non-Canc	NHAHTDD	CNS/Brain
ACH-0015	4.501439	6.229203	Ocular Me	MEL202	Eye
ACH-0008	3.502076	6.706807	Esophago	HGC27	Esophagus/Stomach
ACH-0015	4.157852	6.103078	Intraducta	KKU055	Biliary Tract
ACH-0012	4.176323	7.135761	Non-Canc	TIG3TD	Fibroblast
ACH-0018	5.079805	6.665052	Non-Canc	MMNK1	Biliary Tract
ACH-0008	3.212569	6.327148	Head and	CAL27	Head and Neck
ACH-0019	3.601697	6.235918	Melanoma	MM576	Skin
ACH-0004	3.987321	7.144556	Renal Cell	UO31	Kidney
ACH-0013	4.029453	6.185272	Pancreatic	PACADD1	Pancreas
ACH-0013	4.769772	6.954545	Neuroblas	COGN305	Peripheral Nervous System
ACH-0006	3.542258	7.507081	Non-Smal	HCC2814	Lung
ACH-0011	2.427606	6.264349	T-Lympho	MYLA	Lymphoid
ACH-0009	4.705978	6.70141	Mature B-	HT	Lymphoid
ACH-0008	2.9241	5.423242	Non-Smal	KNS62	Lung

ACH-0014	5.588265	6.827184	Retinoblas	WERIRB1	Eye
ACH-0005	2.448901	5.218006	Esophagea	OE21	Esophagus/Stomach
ACH-0007	3.800123	5.334139	Breast Duc	HCC202	Breast
ACH-0005	3.377124	6.886062	Bladder Ur	SW1710	Bladder/Urinary Tract
ACH-0007	3.87578	7.228049	Diffuse Gli	LNZ308	CNS/Brain
ACH-0017	4.203984	5.54566	Rhabdomy	RHJT	Soft Tissue
ACH-0007	3.634593	6.687761	Non-Smal	LXF289	Lung
ACH-0007	2.24184	6.20594	Lung Neur	DMS273	Lung
ACH-0016	4.485427	6.958727	Head and	UPCISCC0	Head and Neck
ACH-0013	3.968091	6.143026	Cutaneous	A431	Skin
ACH-0010	3.307429	5.800382	Mature T a	FEPD	Lymphoid
ACH-0003	3.997292	7.963821	Lung Neur	NCIH69	Lung
ACH-0016	4.344118	6.636335	Non-Semi	TERA2	Testis
ACH-0016	4.676944	6.976364	Esophago	SKGT4	Esophagus/Stomach
ACH-0015	4.227279	6.843356	Cutaneous	HKA1	Skin
ACH-0002	2.682573	5.177519	Intraducta	SNU1079	Biliary Tract
ACH-0015	2.475085	4.825277	B-Lympho	HG3	Lymphoid
ACH-0002	3.334854	6.717265	Pancreatic	HUPT4	Pancreas
ACH-0016	4.169925	6.538849	Diffuse Gli	NP5	CNS/Brain
ACH-0014	4.786074	6.444104	Urethral C	UMUC14	Bladder/Urinary Tract
ACH-0009	4.9241	6.133399	Colorectal	SNU81	Bowel
ACH-0014	5.293885	6.252476	Bladder Ur	UMUC10	Bladder/Urinary Tract
ACH-0003	4.19456	7.375039	Pleural Me	ISTMES2	Pleura
ACH-0014	4.370862	6.159064	Bladder Ur	UMUC13	Bladder/Urinary Tract
ACH-0005	4.286142	7.266131	Melanoma	C32	Skin
ACH-0005	4.572283	7.197806	Lung Neur	DMS153	Lung
ACH-0006	3.30305	6.858976	Renal Cell	KMRC1	Kidney
ACH-0007	4	6.825022	Non-Canc	DM3	Fibroblast
ACH-0008	2.397803	6.647602	Non-Smal	HCC1195	Lung
ACH-0007	2.618239	6.257011	Head and	PECAPJ41	Head and Neck
ACH-0014	4.907371	6.379725	Neuroblas	CHLA90	Peripheral Nervous System
ACH-0004	3.056584	5.150153	Renal Cell	A704	Kidney
ACH-0003	4.308885	6.81596	Neuroblas	IMR32	Peripheral Nervous System
ACH-0024	4.68594	6.340384	Myeloprol	HAP1	Myeloid
ACH-0006	2.990955	6.302867	Invasive Br	HDQP1	Breast
ACH-0002	3.64501	7.655566	Renal Cell	CAKI2	Kidney
ACH-0014	4.316146	5.737416	Mature B-	FARAGE	Lymphoid
ACH-0008	3.738768	6.812113	Bladder Ur	647V	Bladder/Urinary Tract
ACH-0002	5.131343	7.601177	Neuroblas	NH6	Peripheral Nervous System
ACH-0016	3.858976	6.740523	Intrachole	NOZ	Biliary Tract
ACH-0014	3.704872	5.330917	Ocular Me	921	Eye
ACH-0001	3.910733	7.002027	Acute Mye	SIGM5	Myeloid
ACH-0008	4.435629	5.618239	B-Lympho	RS411	Lymphoid
ACH-0001	2.93546	6.209648	Non-Smal	NCIH2444	Lung
ACH-0027	4.417515	6.102658	Ewing Sarc	NCCES1C1	Bone
ACH-0006	3.554589	6.523719	Non-Smal	SW1573	Lung
ACH-0000	3.69265	6.640245	Embryona	D283MED	CNS/Brain
ACH-0004	3.329124	6.015025	Esophago	GSU	Esophagus/Stomach
ACH-0006	4.045268	5.768714	Non-Smal	IALM	Lung
ACH-0002	3.948601	6.646595	Mature B-	NUDUL1	Lymphoid
ACH-0007	2.99458	5.831117	Esophagea	KYSE70	Esophagus/Stomach
ACH-0005	3.747387	6.508904	Lung Neur	DMS114	Lung
ACH-0020	3.049631	6.597382	Colorectal	ECC4	Bowel
ACH-0003	3.195348	6.349967	Mature B-	KMS12BM	Lymphoid
ACH-0017	4.970394	6.880073	Mature T a	CTV1DM	Lymphoid
ACH-0014	1.130931	3.82273	Squamous	SW954	Vulva/Vagina
ACH-0010	4.339137	5.405652	Embryona	D458	CNS/Brain
ACH-0000	3.690417	7.16088	Colorectal	C2BBE1	Bowel
ACH-0004	4.54782	6.429616	Colorectal	NCIH716	Bowel

ACH-0017	2.182692	4.630522	Mature B-	VAL	Lymphoid
ACH-0000	3.244887	6.272956	Acute Mye	HL60	Myeloid
ACH-0015	2.104337	5.511278	Melanoma	MM386	Skin
ACH-0009	3.599318	6.858105	Endometri	JHUEM7	Uterus
ACH-0026	4.573496	6.60585	Diffuse Gli	170MGBA	CNS/Brain
ACH-0008	4.416164	5.417853	Mature B-	EB1	Lymphoid
ACH-0007	3.393691	6.184875	Invasive Br	JIMT1	Breast
ACH-0015	4.497612	7.107688	Cervical Sc	HCS2	Cervix
ACH-0028	4.255501	6.65435	Melanoma	YUHOIN	Skin
ACH-0007	4.518535	6.559186	Hodgkin L	L1236	Lymphoid
ACH-0010	4.256256	6.824895	Embryona	BT16	CNS/Brain
ACH-0006	3.738768	7.304511	Renal Cell	SLR26	Kidney
ACH-0003	3.165108	6.776104	Pancreatic	CAPAN1	Pancreas
ACH-0000	3.920293	6.891784	Non-Smal	HCC827	Lung
ACH-0006	3.190615	6.883987	Rhabdomy	RH18	Soft Tissue
ACH-0025	4.702658	6.629357	Renal Cell	SNU482	Kidney
ACH-0004	4.002703	6.849249	Renal Cell	TUHR10TK	Kidney
ACH-0014	4.639811	6.888743	Bladder Ur	UMUC11	Bladder/Urinary Tract
ACH-0006	3.41007	7.503508	Hepatocel	SNU878	Liver
ACH-0003	2.784504	6.43179	Colorectal	CL14	Bowel
ACH-0002	4.218781	7.174426	Diffuse Gli	U178	CNS/Brain
ACH-0003	3.040892	6.170125	Colorectal	NCIH508	Bowel
ACH-0002	3.275007	6.269781	Colorectal	CL11	Bowel
ACH-0013	4.487358	6.469235	Esophago	OACM51	Esophagus/Stomach
ACH-0002	3.672425	7.422485	Diffuse Gli	KALS1	CNS/Brain
ACH-0009	3.536053	5.961623	Non-Smal	NCIH650	Lung
ACH-0006	2.675816	6.063287	Non-Smal	CORL23	Lung
ACH-0024	4.365972	5.469235	Melanoma	HT144SKIN	Skin
ACH-0013	2.163499	6.004052	Pancreatic	PACADD1	Pancreas
ACH-0010	4.890447	6.68902	Ewing Sarc	CHLA10	Bone
ACH-0002	4.190615	6.283551	Invasive Br	AU565	Breast
ACH-0009	3.732269	5.226509	Colorectal	RKO	Bowel
ACH-0007	3.620586	7.080871	Acute Mye	OCIM1	Myeloid
ACH-0016	1.056584	3.978196	Head and	UPCISCC1	Head and Neck
ACH-0010	4.581351	7.824959	Diffuse Gli	BECKER	CNS/Brain
ACH-0002	4.380591	6.487197	Non-Smal	NCIH1755	Lung
ACH-0002	4.221104	7.228145	Non-Smal	RERFLCAI	Lung
ACH-0001	3.467279	7.134837	Pancreatic	PANC1	Pancreas
ACH-0017	4.69488	6.524346	Liposarcor	LPS27	Soft Tissue
ACH-0002	3.476382	7.735387	Renal Cell	UOK101	Kidney
ACH-0001	3.771886	6.501758	Mature B-	OCILY3	Lymphoid
ACH-0007	3.813525	6.715344	Mature T a	KIJK	Lymphoid
ACH-0007	3.24184	6.840086	Colorectal	SNUC1	Bowel
ACH-0008	3.063503	6.943101	Invasive Br	CAL851	Breast
ACH-0011	3.764474	6.756623	Acute Mye	MONOMA	Myeloid
ACH-0016	3.358959	6.107269	Intrachole	OCUG1	Biliary Tract
ACH-0001	3.961623	8.007924	Non-Canc	HS343T	Fibroblast
ACH-0003	3.82273	7.984931	Diffuse Gli	CCFSTTG1	CNS/Brain
ACH-0015	3.835924	5.048323	Melanoma	MM415	Skin
ACH-0008	4.027685	7.35808	Melanoma	HS939T	Skin
ACH-0017	2.827819	5.097189	Head and	UPCISCC2	Head and Neck
ACH-0001	4.020591	6.267161	Mature T a	TALL1	Lymphoid
ACH-0005	4.399171	5.644722	Bladder Ur	VMCUB1	Bladder/Urinary Tract
ACH-0009	4.588565	6.385086	Esophago	TGBC11TK	Esophagus/Stomach
ACH-0001	3.269033	6.061776	Mature B-	OCILY19	Lymphoid
ACH-0005	3.790772	6.025029	Esophago	ECC10	Esophagus/Stomach
ACH-0008	3.909773	7.140676	Well-Diffe	FTC238	Thyroid
ACH-0016	4.028569	5.679199	Head and	OSC20	Head and Neck
ACH-0006	2.857981	6.385949	Head and	PECAPJ15	Head and Neck

ACH-0019	4.209453	4.400538	Melanoma	MM485	Skin
ACH-0013	3.458119	5.381283	Ovarian Ep	COV413A	Ovary/Fallopian Tube
ACH-0005	3.061776	5.182295	Head and	HSC4	Head and Neck
ACH-0017	4.028569	6.241459	Mature B-	WSUNHL	Lymphoid
ACH-0020	3.639232	6.26922	Ocular Me	OMM25	Eye
ACH-0001	4.109361	7.50311	Pleural Me	NCIH2052	Pleura
ACH-0005	3.856986	7.932746	Non-Canc	HS821T	Fibroblast
ACH-0001	3.626439	6.377991	Ovarian Ep	OAW28	Ovary/Fallopian Tube
ACH-0008	4.030336	5.65621	Hodgkin L	KMH2	Lymphoid
ACH-0012	3.873813	7.282347	Synovial S	127399	Soft Tissue
ACH-0025	4.605257	6.933573	Hepatocel	SNU739	Liver
ACH-0003	3.658783	8.053274	Ovarian Ep	JHOC5	Ovary/Fallopian Tube
ACH-0015	4.170726	6.254745	Pleural Me	MERO84	Pleura
ACH-0008	2.788686	6.117903	Colorectal	SW480	Bowel
ACH-0008	3.92505	6.247928	Esophago	SNU719	Esophagus/Stomach
ACH-0005	4.399855	5.442943	Pancreatic	SNU410	Pancreas
ACH-0014	5.126395	6.351911	Bladder U	UMUC7	Bladder/Urinary Tract
ACH-0006	3.176323	6.607182	Non-Smal	A549	Lung
ACH-0013	5.268659	7.855305	Non-Canc	HA1E	Kidney
ACH-0000	3.377124	6.666331	B-Lympho	697	Lymphoid
ACH-0004	3.775051	6.433627	Renal Cell	VMRCRCV	Kidney
ACH-0001	3.49057	6.428946	Adenosqu	KP3	Pancreas
ACH-0008	3.107688	7.282347	Intraducta	HUH28	Biliary Tract
ACH-0006	3.667892	6.256445	Mature B-	SUDHL8	Lymphoid
ACH-0001	4.170726	6.703211	Acute Mye	KASUMI6	Myeloid
ACH-0009	4.0268	7.708808	Colorectal	HCT15	Bowel
ACH-0008	4.936873	7.345982	Lung Neu	NCIH1048	Lung
ACH-0008	4.577731	7.091277	Diffuse Gli	LN18	CNS/Brain
ACH-0002	4.128458	6.629794	Mature T a	SUPM2	Lymphoid
ACH-0000	4.330558	6.898934	Ewing Sarc	SKES1	Bone
ACH-0003	4.307429	7.923743	Non-Canc	HS870T	Fibroblast
ACH-0020	3.838952	6.533408	Melanoma	SKMEL19	Skin
ACH-0007	4.511595	6.805937	Esophago	MKN74	Esophagus/Stomach
ACH-0001	3.008989	7.513333	Invasive Br	HCC1599	Breast
ACH-0031	4.877744	7.307611	Non-Canc	ABMT0551	Muscle
ACH-0002	4.212569	6.258519	Invasive Br	DU4475	Breast
ACH-0001	4.035624	7.598648	Head and	SW579	Head and Neck
ACH-0000	4.177918	6.812883	Myeloprol	NCO2	Myeloid
ACH-0005	4.778734	7.633068	Mature T a	HUT78	Lymphoid
ACH-0011	4.900625	7.18933	Ewing Sarc	SKPNDW	Bone
ACH-0005	3.711495	6.851749	Diffuse Gli	T98G	CNS/Brain
ACH-0003	3.908813	6.376777	Ewing Sarc	MHHES1	Bone
ACH-0002	3.434295	6.127221	Endometri	KLE	Uterus
ACH-0031	5.115616	7.331454	Non-Canc	ABMT0292	CNS/Brain
ACH-0005	3.399171	5.992768	Colorectal	SNU61	Bowel
ACH-0005	3.277985	6.465158	Ovarian Ep	JHOS4	Ovary/Fallopian Tube
ACH-0016	2.841973	5.706254	Head and	UPCISCC0	Head and Neck
ACH-0005	4.887525	6.948134	Lung Neu	NCIH1876	Lung
ACH-0009	4.094236	6.759555	Mature B-	NAMALW.	Lymphoid
ACH-0031	4.560715	6.346248	Non-Canc	ABMT0782	Muscle
ACH-0005	3.635754	5.651339	Esophage	TDOTT	Esophagus/Stomach
ACH-0016	2.952334	6.0054	Prostate A	SHMAC4	Prostate
ACH-0003	3.434295	6.350497	Lung Neu	NCIH2081	Lung
ACH-0009	3.775051	7.774458	Endometri	HEC1A	Uterus
ACH-0002	4.309613	6.26922	Mature B-	LP1	Lymphoid
ACH-0001	3.099295	5.796234	Embryona	BT12	CNS/Brain
ACH-0009	4.487358	7.042316	Colorectal	SNU175	Bowel
ACH-0013	3.212569	4.954662	Head and	H157	Head and Neck
ACH-0006	3.255501	7.299025	Invasive Br	HCC2157	Breast

ACH-0002	3.182692	6.810443	Intraducta	SNU245	Biliary Tract
ACH-0001	4.263034	7.827756	Non-Canc	HS863T	Fibroblast
ACH-0003	4.000901	6.927659	Non-Smal	NCIH1781	Lung
ACH-0013	4.272023	6.324991	Mixed Cer	DOTC2451	Cervix
ACH-0007	3.681449	6.868884	Mature B-	KMS11	Lymphoid
ACH-0009	4.934045	6.979911	T-Lymphc	PF382	Lymphoid
ACH-0007	3.569248	6.738227	Mature B-	MM1S	Lymphoid
ACH-0028	4.017031	6.119564	Osteosarc	OS384	Bone
ACH-0016	4.231893	5.868143	Intraducta	TFK1	Biliary Tract
ACH-0003	4.263786	6.103288	Myeloprol	JURLMK1	Myeloid
ACH-0003	3.759156	6.434628	Renal Cell	SLR25	Kidney
ACH-0009	3.675816	6.644866	Non-Smal	HCC1359	Lung
ACH-0008	3.077243	6.675393	Lung Neu	NCIH524	Lung
ACH-0006	4.356144	6.815319	Acute Mye	CMK	Myeloid
ACH-0005	3.841973	7.586465	Non-Smal	CALU1	Lung
ACH-0020	5.754353	6.437128	Lung Neu	LU165	Lung
ACH-0005	3.364572	6.712871	Non-Hod	MC116	Lymphoid
ACH-0008	3.998196	7.134118	Head and	YD15	Head and Neck
ACH-0009	5.150966	6.723559	Non-Smal	HCC2450	Lung
ACH-0001	4.147307	7.269314	Non-Smal	NCIH3255	Lung
ACH-0004	3.037382	6.238405	Colorectal	HCC56	Bowel
ACH-0007	3.301588	6.17971	Non-Smal	HCC1833	Lung
ACH-0006	3.117695	6.264724	Hepatobla	HUH6	Liver
ACH-0005	4.450881	6.498889	Lung Neu	NCIH1184	Lung
ACH-0008	3.805292	7.086083	Endometri	COLO684	Uterus
ACH-0007	3.867896	5.862203	Esophagea	TE14	Esophagus/Stomach
ACH-0001	4.336283	7.431539	Neuroblas	CHP126	Peripheral Nervous System
ACH-0020	3.753818	6.136479	Osteosarc	NOS1	Bone
ACH-0004	4.209453	6.3875	Mature B-	CA46	Lymphoid
ACH-0025	5.061344	5.846995	Colorectal	SNU1460	Bowel
ACH-0014	2.480265	6.503985	Colorectal	C99	Bowel
ACH-0008	4.077243	7.234578	Lung Neu	SW1271	Lung
ACH-0016	3.424922	5.887525	Head and	UPCISCC0	Head and Neck
ACH-0000	4.219556	6.153603	Diffuse Gli	GOS3	CNS/Brain
ACH-0005	3.80426	6.96763	Non-Smal	HCC78	Lung
ACH-0007	4.249445	7.725809	Osteosarc	SJSA1	Bone
ACH-0016	3.395063	5.949301	B-Lymphc	TANOUE	Lymphoid
ACH-0008	3.864929	6.0974	Bladder U	UMUC1	Bladder/Urinary Tract
ACH-0005	3.360364	6.595295	Colorectal	RCM1	Bowel
ACH-0004	3.519793	7.255501	Uterine Sa	MESSA	Uterus
ACH-0003	3.817623	5.789468	Lung Neu	NCIH2196	Lung
ACH-0020	4.041769	5.852748	Neuroblas	TN2	Peripheral Nervous System
ACH-0007	4.736605	7.04756	Acute Mye	P31FUJ	Myeloid
ACH-0007	2.786596	7.171727	Colorectal	NCIH854	Bowel
ACH-0005	3.737687	6.0568	Esophago	KE39	Esophagus/Stomach
ACH-0000	3.81455	7.087993	Non-Smal	HCC4006	Lung
ACH-0000	3.368768	6.340562	Pancreatic	PATU8988	Pancreas
ACH-0016	2.411426	6.104546	Non-Semi	SUSA	Testis
ACH-0003	3.776104	6.467606	Mature B-	DB	Lymphoid
ACH-0003	2.83996	8.113378	Invasive B	HCC1143	Breast
ACH-0005	5.098032	7.020702	T-Lymphc	PEER	Lymphoid
ACH-0009	4.145677	6.66775	Esophago	SNU520	Esophagus/Stomach
ACH-0020	4.278728	4.192983	Melanoma	A375SKIN	Skin
ACH-0005	2.987321	6.161686	Ovarian Ep	FUOV1	Ovary/Fallopian Tube
ACH-0001	3.399171	8.343719	Invasive B	HS578T	Breast
ACH-0024	4.033863	6.964283	Melanoma	RVH421SK	Skin
ACH-0006	2.127633	4.682011	Ovarian Ep	OVMANA	Ovary/Fallopian Tube
ACH-0003	4.066089	7.224773	Non-Smal	HCC2108	Lung
ACH-0017	2.553361	6.097189	Invasive B	VP267	Breast

ACH-0000	4.8089	7.806453	Diffuse Gli	U118MG	CNS/Brain
ACH-0010	4.489928	6.166515	Mature T a	DERL2	Lymphoid
ACH-0004	3.605257	6.57198	Ovarian Ep	TYKNU	Ovary/Fallopian Tube
ACH-0010	4.154616	6.327328	Melanoma	CHL1DM	Skin
ACH-0019	4.111866	5.917193	Pleural Me	NO36	Pleura
ACH-0003	4.264536	6.467932	Diffuse Gli	SNU626	CNS/Brain
ACH-0016	2.353323	6.492013	Ovarian Ep	PEO1	Ovary/Fallopian Tube
ACH-0004	4.636335	7.5462	Diffuse Gli	SW1088	CNS/Brain
ACH-0004	4.446256	5.991862	Acute Mye	KO52	Myeloid
ACH-0013	1.500802	5.084915	Invasive Br	SUM185PE	Breast
ACH-0005	2.160275	6.284107	Head and	BICR16	Head and Neck
ACH-0003	5.319762	6.627899	Diffuse Gli	LN215	CNS/Brain
ACH-0004	3.683696	6.807226	Colorectal	NCIH747	Bowel
ACH-0004	3.155425	6.087675	Colorectal	SKCO1	Bowel
ACH-0002	3.596935	7.414051	Non-Canc	HS819T	Fibroblast
ACH-0003	3.468583	6.391458	Esophagea	TE10	Esophagus/Stomach
ACH-0014	4.533563	6.146492	Ewing Sarc	TC138	Bone
ACH-0009	3.518535	6.83023	Endometri	JHUEM1	Uterus
ACH-0008	3.397803	6.842853	Non-Smal	NCIH1651	Lung
ACH-0008	4.307429	6.897966	Mature B-	KMM1	Lymphoid
ACH-0007	3.596935	6.982195	Melanoma	HS695T	Skin
ACH-0016	4.735522	6.694184	Diffuse Gli	ONDA8	CNS/Brain
ACH-0007	3.81455	6.803744	Esophago	SNU601	Esophagus/Stomach
ACH-0008	3.624101	6.364923	Invasive Br	MDAMB41	Breast
ACH-0012	4.8089	6.611763	Embryona	UW228	CNS/Brain
ACH-0016	4.235727	6.325171	Acute Mye	OCIM2	Myeloid
ACH-0026	3.874797	5.561021	Non-Smal	ETCC016	Lung
ACH-0002	4.297191	7.22062	Pancreatic	PANC0403	Pancreas
ACH-0003	3.350497	6.392661	Colorectal	T84	Bowel
ACH-0019	5.15866	5.673839	B-Lympho	NALM16	Lymphoid
ACH-0013	5.898692	6.569096	Neuroblas	COGN278	Peripheral Nervous System
ACH-0008	2.715893	5.891419	Mature B-	OCIMY5	Lymphoid
ACH-0001	3.145677	6.787641	Lung Neur	CORL311	Lung
ACH-0005	3.253989	5.734168	Diffuse Gli	SNU489	CNS/Brain
ACH-0005	3.670161	6.599913	Pleural Me	ISTMES1	Pleura
ACH-0006	3.198494	7.720005	Invasive Br	HCC70	Breast
ACH-0001	4.177121	8.320349	Non-Canc	HS274T	Fibroblast
ACH-0009	3.85997	6.653777	Non-Smal	NCIH2172	Lung
ACH-0014	3.762349	6.303781	Mature B-	BLUE1	Lymphoid
ACH-0002	3.575312	5.847496	Colorectal	OUMS23	Bowel
ACH-0027	4.844486	5.64789	Chondrosa	NCCDCS1	Bone
ACH-0007	3.269033	7.340295	Ovarian Ep	CAOV3	Ovary/Fallopian Tube
ACH-0013	2.488001	5.782933	Cervical Sc	MS751	Cervix
ACH-0029	4.502076	6.150966	Invasive Br	BCK4	Breast
ACH-0002	3.581351	6.799217	Diffuse Gli	SF539	CNS/Brain
ACH-0017	5.209063	6.942045	Osteosarc	CAL72	Bone
ACH-0016	4.777683	7.303324	Mature B-	OCILY7	Lymphoid
ACH-0006	3.300124	6.131754	Lung Neur	NCIH211	Lung
ACH-0005	2.748461	6.454176	Non-Smal	NCIH2073	Lung
ACH-0005	4.214902	6.642702	Non-Smal	SQ1	Lung
ACH-0000	4.726286	6.373648	Acute Mye	MUTZ3	Myeloid
ACH-0000	3.201634	6.390943	Pancreatic	HPAFII	Pancreas
ACH-0003	3.462052	6.71452	Hepatocel	SKHEP1	Liver
ACH-0007	3.344828	5.970624	Ovarian Ep	RMGI	Ovary/Fallopian Tube
ACH-0013	2.704872	4.139142	Invasive Br	SUM52PE	Breast
ACH-0018	3.41684	6.54782	Intraducta	ICC8	Biliary Tract
ACH-0003	4.028569	7.36247	Non-Smal	HCC461	Lung
ACH-0020	4.400538	5.412443	Rhabdomy	RMSYM	Soft Tissue
ACH-0002	4.002703	6.113742	Acute Mye	PL21	Myeloid

ACH-0024	4.90352	6.766993	Ovarian Ep	MESOV	Ovary/Fallopian Tube
ACH-0007	4.148934	6.6401	Non-Smal	NCIH810	Lung
ACH-0004	3.8885	7.665336	Non-Canc	HS739T	Fibroblast
ACH-0001	4.250962	8.106432	Non-Canc	TO175T	Fibroblast
ACH-0001	4.125155	7.898994	Non-Canc	HS839T	Fibroblast
ACH-0002	3.960697	7.28568	Diffuse Gli	LN382	CNS/Brain
ACH-0010	4.169925	6.495695	Osteosarc	143B	Bone
ACH-0004	3.163499	6.208478	Endometri	SNU685	Uterus
ACH-0021	5.053111	5.071248	Lung Neu	NCIH1688	Lung
ACH-0020	2.811471	5.258142	Endometri	HOUAI	Uterus
ACH-0011	4.921246	6.94825	T-Lympho	MTA	Lymphoid
ACH-0012	4.888987	7.134632	Ovarian Ep	BIN67	Ovary/Fallopian Tube
ACH-0000	4.666188	6.968436	B-Lympho	MHHCALL	Lymphoid
ACH-0014	1.014355	6.44576	Invasive B	VP229	Breast
ACH-0004	4.178715	6.81954	Non-Canc	OELE	Ovary/Fallopian Tube
ACH-0008	4.339137	7.135453	Esophagea	KYSE450	Esophagus/Stomach
ACH-0004	3.408712	5.714246	Acute Mye	ME1	Myeloid
ACH-0015	4.738227	5.977051	Cutaneous	HSC1	Skin
ACH-0000	3.914565	6.128252	Mature B-	OPM2	Lymphoid
ACH-0006	3.077243	5.27165	Esophago	NUGC4	Esophagus/Stomach
ACH-0006	3.276497	5.863938	Lung Neu	SBC5	Lung
ACH-0005	2.650765	6.911572	Pancreatic	PATU8902	Pancreas
ACH-0002	4.292782	6.317413	Acute Mye	KASUMI1	Myeloid
ACH-0003	3.538538	7.051046	Esophago	SNU5	Esophagus/Stomach
ACH-0015	4.758623	5.339137	Merkel Ce	MCC142	Skin
ACH-0007	3.620586	7.686781	Esophago	KATOIII	Esophagus/Stomach
ACH-0025	5.219169	6.635609	Melanoma	M140325	Skin
ACH-0002	2.565597	6.275193	Colorectal	SNU1033	Bowel
ACH-0002	3.975447	7.697871	Renal Cell	KMRC20	Kidney
ACH-0001	3.467279	6.246788	Pancreatic	PANC0327	Pancreas
ACH-0017	4.249445	5.852249	B-Lympho	SEMK2	Lymphoid
ACH-0013	4.267536	6.282996	Invasive B	SUM1315I	Breast
ACH-0011	3.714795	6.112283	Bladder U	RT11284	Bladder/Urinary Tract
ACH-0005	3.647315	6.159871	Myeloprol	K562	Myeloid
ACH-0011	4.695994	5.426265	Renal Cell	CCLFPEDS	Kidney
ACH-0000	2.533563	6.988571	Sarcoma, I	S117	Soft Tissue
ACH-0007	3.237258	6.587365	Non-Smal	NCIH1623	Lung
ACH-0004	3.31034	7.459186	Hepatocel	SNU449	Liver
ACH-0003	3.211012	5.561021	Acute Mye	OCIAML3	Myeloid
ACH-0004	3.627607	6.250393	Bladder U	KU1919	Bladder/Urinary Tract
ACH-0006	3.671293	6.662063	Esophagea	OE19	Esophagus/Stomach
ACH-0015	4.596339	6.280956	Pleural Me	MERO41	Pleura
ACH-0002	3.539779	7.177619	Esophago	OCUM1	Esophagus/Stomach
ACH-0009	3.681449	6.874797	Ovarian Ep	OVK18	Ovary/Fallopian Tube
ACH-0008	3.377124	7.039248	Diffuse Gli	SNU738	CNS/Brain
ACH-0004	3.214125	7.161988	Hepatocel	JHH4	Liver
ACH-0005	3.523562	7.22033	Lung Neu	NCIH2171	Lung
ACH-0031	4.448901	6.482203	Non-Canc	ABMT0795	Lung
ACH-0011	4.405312	6.6094	Mature B-	OCILY132	Lymphoid
ACH-0008	3.201634	7.019924	Non-Smal	NCIH661	Lung
ACH-0000	3.97728	7.900021	Non-Canc	HS281T	Fibroblast
ACH-0004	3.593354	6.268846	Diffuse Gli	KNS81	CNS/Brain
ACH-0002	3.003602	6.96463	Non-Canc	HS742T	Fibroblast
ACH-0016	4.297191	6.809929	Esophago	SNU638	Esophagus/Stomach
ACH-0009	3.940167	6.290756	Colorectal	SNU407	Bowel
ACH-0000	3.736605	7.035734	Non-Canc	HEKTE	Kidney
ACH-0006	4.294253	7.474112	Non-Smal	NCIH1734	Lung
ACH-0001	3.819668	7.243364	Non-Canc	HS255T	Fibroblast
ACH-0001	3.273516	6.057667	Mature B-	KARPAS62	Lymphoid

ACH-0005	4.050502	6.907972	Small Bow	HUTU80	Bowel
ACH-0019	4.059182	4.969933	Melanoma	NZM7	Skin
ACH-0014	4.44824	6.2804	Non-Semi	1777NRPN	Testis
ACH-0016	4.079805	5.584061	Acute Mye	SHI1	Myeloid
ACH-0020	2.996389	4.44427	Head and	CA922	Head and Neck
ACH-0016	3.755956	5.6401	Prostate A	SHMAC5	Prostate
ACH-0001	3.119356	6.934399	Pancreatic	SW1990	Pancreas
ACH-0002	3.320485	5.945327	Pancreatic	HPAC	Pancreas
ACH-0006	3.72792	5.778997	Colorectal	SNU503	Bowel
ACH-0020	4.371559	5.419539	Ocular Me	OMM1	Eye
ACH-0006	3.176323	6.976364	Diffuse Gli	SF268	CNS/Brain
ACH-0014	3.292782	5.060047	Endometri	EMTOKA	Uterus
ACH-0001	2.438293	6.12143	Head and	SCC25	Head and Neck
ACH-0000	3.641546	6.139961	Acute Mye	BDCM	Myeloid
ACH-0001	4.31034	6.781622	Acute Mye	THP1	Myeloid
ACH-0004	2.192194	4.892877	Head and	BICR6	Head and Neck
ACH-0015	4.662775	6.652917	Endometri	HEC116	Uterus
ACH-0008	4.501439	7.050611	Non-Smal	HCC1438	Lung
ACH-0016	3.609991	6.835545	Head and	OSC19	Head and Neck
ACH-0008	3.464668	7.392833	Colorectal	CL34	Bowel
ACH-0008	3.221877	5.880196	Non-Smal	MORCPR	Lung
ACH-0008	3.636915	6.616328	Esophage	KYSE510	Esophagus/Stomach
ACH-0005	3.01078	6.87172	Lung Neu	NCIH146	Lung
ACH-0005	3.88655	7.215484	Non-Canc	HS606T	Fibroblast
ACH-0000	3.431623	7.383877	Non-Hod	HH	Lymphoid
ACH-0001	3.770829	7.422233	Non-Canc	HS737T	Fibroblast
ACH-0005	2.589763	4.792855	Invasive B	UACC812	Breast
ACH-0007	3.95977	7.034084	Mature B-	P3HR1	Lymphoid
ACH-0006	3.058316	5.627315	Non-Smal	BEN	Lung
ACH-0009	4.353323	6.791293	Myeloprol	KCL22	Myeloid
ACH-0005	3.226509	5.90665	Mature B-	KMS34	Lymphoid
ACH-0006	3.192194	6.555663	Colorectal	SW948	Bowel
ACH-0024	4.519164	5.434628	Melanoma	HT144SKIN	Skin
ACH-0001	4.1127	6.687761	Invasive B	HCC1187	Breast
ACH-0024	4.631686	6.658354	Non-Canc	RPE1SS77	Eye
ACH-0003	4.012569	6.92505	Acute Mye	MOLM16	Myeloid
ACH-0006	4.109361	6.561479	Head and	SNU1041	Head and Neck
ACH-0004	3.346248	6.050284	Colorectal	SW1463	Bowel
ACH-0031	5.028127	7.135863	Non-Canc	ABMT078:	CNS/Brain
ACH-0020	4.825786	5.627023	Pancreatic	PK8	Pancreas
ACH-0001	3.600508	5.876271	Pancreatic	HUPT3	Pancreas
ACH-0006	3.885574	6.486393	Melanoma	RVH421	Skin
ACH-0013	5.099295	6.358256	Neuroblas	CHP134	Peripheral Nervous System
ACH-0004	3.578939	5.968552	Myeloprol	BV173	Myeloid
ACH-0005	4.92505	7.029895	Cervical Sc	SIHA	Cervix
ACH-0018	4.393691	6.09043	Liposarcor	LPS510	Soft Tissue
ACH-0013	5.430285	7.919221	Lung Neu	SCLC22H	Lung
ACH-0010	5.211012	7.106118	Mature T a	DL40	Lymphoid
ACH-0015	3.577731	5.221104	Cervical A	HT3	Cervix
ACH-0015	3.439623	5.720005	Mature B-	KML1	Lymphoid
ACH-0000	3.986411	5.987776	Melanoma	HS294T	Skin
ACH-0027	4.544733	6.981396	Leiomyosa	NCCLMS1	Soft Tissue
ACH-0006	3.62293	6.007644	Esophage	KYSE520	Esophagus/Stomach
ACH-0026	4.720826	5.727376	Nerve She	S462	Peripheral Nervous System
ACH-0018	2.735522	5.709291	Intraducta	OZ	Biliary Tract
ACH-0004	4.453518	4.665052	Diffuse Gli	LN428	CNS/Brain
ACH-0003	3.842979	5.521679	Diffuse Gli	H4	CNS/Brain
ACH-0027	4.760221	5.731183	Nerve She	MPNST72	Peripheral Nervous System
ACH-0001	3.725741	6.911092	Lung Neu	NCIH1341	Lung

ACH-0004	3.395063	6.634738	Cervical So	SF767	Cervix
ACH-0004	2.565597	5.33985	Non-Smal	NCIH1915	Lung
ACH-0010	3.316146	6.482203	Cervical A	HELA	Cervix
ACH-0020	4.263786	5.393004	Head and	T3M5	Head and Neck
ACH-0005	4.82985	7.157549	Rhabdoid	TTC709	Kidney
ACH-0007	3.787641	7.179412	Diffuse Gli	GI1	CNS/Brain
ACH-0003	3.840967	7.154008	Bladder U	J82	Bladder/Urinary Tract
ACH-0031	5.079805	6.789729	Non-Canc	ABMT0781	CNS/Brain
ACH-0000	3.812498	7.394634	Colorectal	NCIH684	Bowel
ACH-0003	3.989139	6.589913	Mature B-	RL	Lymphoid
ACH-0002	3.295723	6.930501	Pancreatic	KP4	Pancreas
ACH-0001	2.833902	6.110823	Ovarian Ep	JHOS2	Ovary/Fallopian Tube
ACH-0002	3.541019	6.562853	Esophago	JHESOAD1	Esophagus/Stomach
ACH-0006	4.159468	6.660495	Osteosarc	HOS	Bone
ACH-0008	3.956057	6.364923	Esophagea	KYSE150	Esophagus/Stomach
ACH-0009	2.99458	6.142005	Ovarian Ep	ES2	Ovary/Fallopian Tube
ACH-0003	4.167519	6.297375	Melanoma	RPMI7951	Skin
ACH-0011	1.035624	4.06695	T-Lympho	KHYG1	Lymphoid
ACH-0002	3.351911	6.386466	Head and	SCC4	Head and Neck
ACH-0002	3.70044	6.372778	Ovarian Ep	JHOM1	Ovary/Fallopian Tube
ACH-0003	3.560715	7.934575	Hepatocel	HLF	Liver
ACH-0006	3.529821	6.447579	Diffuse Gli	SNU201	CNS/Brain
ACH-0003	3.68818	7.214805	Osteosarc	MG63	Bone
ACH-0010	2.528571	5.338781	Embryona	CHLA06AT	CNS/Brain
ACH-0006	4.293518	7.199574	Invasive Br	MDAMB15	Breast
ACH-0016	3.485427	6.088099	Head and	SAT	Head and Neck
ACH-0000	3.292782	6.358431	Mature B-	DOHH2	Lymphoid
ACH-0004	3.898208	6.763013	Hepatocel	SNU182	Liver
ACH-0005	4.276497	6.931447	Lung Neur	NCIH1092	Lung
ACH-0000	4.497612	7.223519	Rhabdomy	TE617T	Soft Tissue
ACH-0000	4.196922	7.018367	Non-Canc	HS895T	Fibroblast
ACH-0009	3.493135	6.351911	Ovarian Ep	EFO27	Ovary/Fallopian Tube
ACH-0007	3.647315	6.886428	Well-Diffe	TT2609C0	Thyroid
ACH-0009	3.833902	6.881665	Endometri	MFE319	Uterus
ACH-0009	4.290572	6.14995	Prostate A	MDAPCA2	Prostate
ACH-0013	3.07382	4.887038	Anaplastic	HTCC3	Thyroid
ACH-0000	3.800123	6.207307	Acute Mye	OCIAML5	Myeloid
ACH-0006	4.330558	7.321116	Diffuse Gli	SF126	CNS/Brain
ACH-0014	4.159468	5.562853	Bladder Sc	UMUC5	Bladder/Urinary Tract
ACH-0005	4.017031	7.958379	Diffuse Gli	SNB75	CNS/Brain
ACH-0009	4.012569	6.622638	Prostate A	22RV1	Prostate
ACH-0017	4.563768	6.320304	Osteosarc	HS860T	Bone
ACH-0006	4.571677	6.746985	Pancreatic	SUIT2	Pancreas
ACH-0004	4.153805	6.517118	Non-Smal	NCIH1944	Lung
ACH-0004	2.885574	5.819157	Head and	HSC2	Head and Neck
ACH-0000	3.446256	6.784635	Colorectal	LS513	Bowel
ACH-0004	2.914565	6.197905	Neuroblas	KPNSI9S	Peripheral Nervous System
ACH-0003	4.740388	6.81455	Acute Mye	KG1	Myeloid
ACH-0018	3.810443	7.25484	Intrachole	G415	Biliary Tract
ACH-0007	2.952334	5.617063	Breast Duc	HCC2218	Breast
ACH-0000	3.405992	5.488644	Invasive Br	KPL1	Breast
ACH-0006	3.904002	6.646883	Non-Smal	SKMES1	Lung
ACH-0016	4.451541	6.346602	Mature B-	U2973	Lymphoid
ACH-0000	4.663345	6.068671	Neuroblas	MHHNB11	Peripheral Nervous System
ACH-0020	4.627607	6.343052	Endometri	HTMMT	Uterus
ACH-0003	3.635754	6.263034	Endometri	TEN	Uterus
ACH-0031	4.771886	7.072427	Non-Canc	ABMT0725	Embryonal
ACH-0002	3.806324	7.375735	Non-Canc	HS822T	Fibroblast
ACH-0029	2.827819	5.463034	Ocular Me	UPMM3	Eye

ACH-0005	3.856986	6.890447	Head and	CAL33	Head and Neck
ACH-0015	3.371559	6.061128	Melanoma	MM370	Skin
ACH-0002	3.938286	6.292598	Colorectal	LS1034	Bowel
ACH-0005	3.642702	7.353412	Melanoma	G361	Skin
ACH-0016	4.863443	7.068241	Diffuse Gli	NO10	CNS/Brain
ACH-0025	5.193772	6.296641	Non-Smal	SNU2292	Lung
ACH-0002	4.039138	6.509062	Invasive Br	HCC1419	Breast
ACH-0012	3.581351	6.527164	Melanoma	WM2664	Skin
ACH-0007	3.894333	6.60807	Mature B-	DAUDI	Lymphoid
ACH-0008	3.214125	7.184677	Lung Neur	COLO668	Lung
ACH-0019	4.06953	5.153805	Melanoma	MM253	Skin
ACH-0020	5.027243	7.062964	Ovarian Ca	HSKTC	Ovary/Fallopian Tube
ACH-0005	2.972693	6.847245	Hepatocel	SNU761	Liver
ACH-0028	4.276497	7.000451	Diffuse Gli	TB096	CNS/Brain
ACH-0004	4.094236	5.512543	Mature B-	KMS28BM	Lymphoid
ACH-0007	4.266037	6.644	Bladder Ur	HT1376	Bladder/Urinary Tract
ACH-0005	4.280214	6.632268	Bladder Ur	UMUC3	Bladder/Urinary Tract
ACH-0015	3.952334	7.597755	Lung Neur	LU135	Lung
ACH-0007	3.769772	6.923506	Non-Smal	EKVX	Lung
ACH-0007	3.806324	6.941576	Non-Smal	NCIH1385	Lung
ACH-0005	3.775051	6.961044	Head and	BHY	Head and Neck
ACH-0018	3.377124	6.569248	Intraducta	ICC137	Biliary Tract
ACH-0017	3.763412	6.258896	Liposarcor	LPS141	Soft Tissue
ACH-0015	2.827819	5.198494	Myeloprol	MOLM1	Myeloid
ACH-0004	2.277985	6.053329	Melanoma	UACC62	Skin
ACH-0003	3.575312	7.6858	Renal Cell	G402	Kidney
ACH-0007	4.086614	7.913727	Bladder Ur	JMSU1	Bladder/Urinary Tract
ACH-0002	3.764474	7.735522	Non-Canc	HS834T	Fibroblast
ACH-0007	3.014355	7.197217	Head and	PECAPJ49	Head and Neck
ACH-0006	3.734439	6.875043	Mature B-	RAJI	Lymphoid
ACH-0010	4.649041	7.233332	Meningotl	F5	CNS/Brain
ACH-0001	4.329124	5.809414	Non-Canc	PRECLH	Prostate
ACH-0013	3.65764	5.608218	Invasive Br	SUM229PI	Breast
ACH-0004	3.01078	6.579995	Melanoma	K029AX	Skin
ACH-0008	3.502076	6.950235	Melanoma	IGR1	Skin
ACH-0015	2.427606	4.236493	Head and	H357	Head and Neck
ACH-0002	2.922198	6.539159	Ewing Sarc	CADOES1	Bone
ACH-0002	2.887525	6.368594	Head and	SCC15	Head and Neck
ACH-0006	2.797013	6.397974	Myeloprol	KYO1	Myeloid
ACH-0000	2.899176	5.911931	Prostate A	PC3	Prostate
ACH-0001	4.536053	6.615593	Acute Mye	SET2	Myeloid
ACH-0017	1.464668	6.307975	Liposarcor	LPS6	Soft Tissue
ACH-0016	4.274262	6.039796	Cervical A	SISO	Cervix
ACH-0006	2.944858	5.627607	Esophagea	TE6	Esophagus/Stomach
ACH-0004	4.363872	5.698218	Melanoma	CJM	Skin
ACH-0020	3.927896	7.223229	Melanoma	MMAC	Skin
ACH-0000	3.836934	5.78031	Acute Mye	HEL	Myeloid
ACH-0002	3.179511	7.509379	Non-Smal	NCIH2887	Lung
ACH-0002	3.710393	6.714383	Myeloprol	JK1	Myeloid
ACH-0011	4.386811	6.947783	Renal Cell	SKRC31	Kidney
ACH-0020	3.518535	4.355439	Mature B-	TL1	Lymphoid
ACH-0014	4.458119	6.384223	Anaplastic	ASH3	Thyroid
ACH-0006	2.289834	7.436212	Ovarian Ep	OV7	Ovary/Fallopian Tube
ACH-0004	3.809414	6.237067	Mature B-	MEC1	Lymphoid
ACH-0008	2.632268	5.985045	Mature B-	EJM	Lymphoid
ACH-0016	5.161485	6.477192	Mature B-	U2904	Lymphoid
ACH-0025	4.07382	5.938051	Non-Smal	SNU1327	Lung
ACH-0016	3.258519	5.661636	Prostate A	P4E6	Prostate
ACH-0009	3.914565	6.886184	Non-Smal	NCIH2342	Lung

ACH-0010	4.785551	6.002928	Acute Mye	CMK115	Myeloid
ACH-0005	3.275007	6.209063	Bladder U	HT1197	Bladder/Urinary Tract
ACH-0008	3.587365	6.68832	Non-Smal	CAL12T	Lung
ACH-0019	3.825786	5.321928	Melanoma	NZM42	Skin
ACH-0011	2.944858	6.020147	Mature T a	OCILY12	Lymphoid
ACH-0001	4.051372	6.9331	Mature B-	GA10	Lymphoid
ACH-0031	4.422233	7.979168	Non-Canc	ABMT0457	Muscle
ACH-0017	4.045268	6.300307	Rhabdomy	RH36	Soft Tissue
ACH-0000	3.704872	7.835671	Bladder U	T24	Bladder/Urinary Tract
ACH-0007	2.709291	6.336462	Invasive B	HMC18	Breast
ACH-0000	3.828835	7.081617	Colorectal	CACO2	Bowel
ACH-0000	3.329124	6.828708	Pleural Me	NCIH2452	Pleura
ACH-0006	3.550901	7.22978	Non-Smal	SW900	Lung
ACH-0010	3.802193	7.173127	Rhabdoid	KD	Kidney
ACH-0006	2.889474	7.419033	Pleural Me	NCIH28	Pleura
ACH-0009	3.887525	6.929909	Colorectal	LOVO	Bowel
ACH-0000	3.432959	5.001352	Mature B-	NCIH929	Lymphoid
ACH-0011	3.643856	7.067488	B-Lymphc	KOPN8	Lymphoid
ACH-0020	4.162693	5.940167	Lung Neu	LU134A	Lung
ACH-0003	3.719183	6.013909	Pleural Me	NCIH226	Pleura
ACH-0005	2.192194	6.004277	Non-Smal	NCIH1437	Lung
ACH-0005	2.929791	5.570463	Mature B-	KHM1B	Lymphoid
ACH-0015	4.33985	6.442943	Melanoma	HMY1	Skin
ACH-0008	3.488001	7.141188	Endometri	HEC50B	Uterus
ACH-0004	3.714795	6.730368	Ewing Sar	TC71	Bone
ACH-0004	3.228049	5.214902	Ovarian Ep	OVSAHO	Ovary/Fallopian Tube
ACH-0006	3.849999	6.764739	Esophago	HS746T	Esophagus/Stomach
ACH-0006	4.324811	6.966592	Non-Smal	NCIH596	Lung
ACH-0005	3.017922	5.620293	Mature B-	INA6	Lymphoid
ACH-0002	3.488001	5.84147	Diffuse Gli	U251MG	CNS/Brain
ACH-0008	3.701549	6.188638	Colorectal	SW403	Bowel
ACH-0012	4.885086	6.212958	Retinoblas	Y79	Eye
ACH-0011	3.095924	6.060264	Undifferer	CCLFPEDS	Soft Tissue
ACH-0008	3.936402	6.984134	Hodgkin L	L540	Lymphoid
ACH-0025	2.773996	6.137708	Ovarian Ep	SNU251	Ovary/Fallopian Tube
ACH-0006	3.434295	6.42425	Pleural Me	JL1	Pleura
ACH-0000	3.963474	6.766728	Neuroblas	SIMA	Peripheral Nervous System
ACH-0009	3.368768	5.948134	Invasive B	CAL148	Breast
ACH-0006	3.400538	8.083054	Renal Cell	786O	Kidney
ACH-0000	3.200065	6.956754	Pleural Me	ACCMESC	Pleura
ACH-0001	4.519164	7.179909	Neuroblas	SKNSH	Peripheral Nervous System
ACH-0001	3.452859	7.133605	Bladder U	CAL29	Bladder/Urinary Tract
ACH-0025	3.674687	5.247548	Colorectal	SNU254	Bowel
ACH-0002	4.102658	7.051698	Neuroblas	KPNYN	Peripheral Nervous System
ACH-0006	3.536053	5.456806	Mature B-	A3KAW	Lymphoid
ACH-0009	4.14323	7.531693	T-Lymphc	HPBALL	Lymphoid
ACH-0002	3.651913	6.391802	Non-Smal	CALU6	Lung
ACH-0008	3.129283	6.557042	Esophagea	KYSE410	Esophagus/Stomach
ACH-0000	4.31252	5.000451	Pancreatic	PANC0504	Pancreas
ACH-0001	3.508429	6.554435	Rhabdomy	RH41	Soft Tissue
ACH-0000	3.305971	6.280028	Pancreatic	PANC1005	Pancreas
ACH-0000	4.061776	6.960581	Ovarian Ep	NIHOVCA	Ovary/Fallopian Tube
ACH-0009	3.602884	6.860963	Colorectal	LS180	Bowel
ACH-0006	2.863938	6.982195	Melanoma	IGR37	Skin
ACH-0010	4.399171	6.051807	Rhabdomy	JR	Soft Tissue
ACH-0027	4.524816	5.931919	Nerve She	NCCMPNS	Peripheral Nervous System
ACH-0002	3.232661	6.171127	Esophago	HUG1N	Esophagus/Stomach
ACH-0007	3.177918	6.569704	Head and	YD38	Head and Neck
ACH-0009	3.258519	6.833017	B-Lymphc	RCHACV	Lymphoid

ACH-0027	4.639232	7.463034	Undifferen	NCCUPS2(	Soft Tissue
ACH-0008	4.169925	7.844612	Breast Duc	HCC1954	Breast
ACH-0009	3.889474	6.75141	Endometri	HEC108	Uterus
ACH-0004	4.008092	7.061992	Osteosarc	SAOS2	Bone
ACH-0019	4.557655	6.177918	Melanoma	MM466	Skin
ACH-0009	3.66562	6.400709	Invasive Br	BT474	Breast
ACH-0018	3.589763	5.345538	Hepatocel	KMCH1	Liver
ACH-0006	4.607626	6.608661	Esophage	TE9	Esophagus/Stomach
ACH-0009	3.051372	7.264724	Colorectal	MDST8	Bowel
ACH-0009	4.498251	6.736064	T-Lympho	MOLT16	Lymphoid
ACH-0007	2.835924	7.468502	Esophago	NUGC2	Esophagus/Stomach
ACH-0015	4.428276	6.986752	Melanoma	MM127	Skin
ACH-0009	3.237258	6.913488	Endometri	SNGM	Uterus
ACH-0002	3.385431	7.041221	Lung Neur	NCIH889	Lung
ACH-0017	4.207893	7.374779	Ovarian Ep	OCIC4P	Ovary/Fallopian Tube
ACH-0002	2.972693	7.607774	Pancreatic	DANG	Pancreas
ACH-0014	3.261531	5.659925	Ovarian Ep	UWB1289	Ovary/Fallopian Tube
ACH-0003	3.651913	6.321026	Acute Mye	MOLM13	Myeloid
ACH-0008	3.916477	6.414474	Non-Smal	HARA	Lung
ACH-0008	3.918386	6.773601	Non-Smal	HCC366	Lung
ACH-0018	4.279471	5.255878	Intraducta	HKGZCC	Biliary Tract
ACH-0015	4.453518	5.728737	Well-Diffe	IHH4	Thyroid
ACH-0016	3.365972	6.941106	Acute Mye	SKNO1	Myeloid
ACH-0000	3.938286	6.344651	Renal Cell	SLR21	Kidney
ACH-0001	4.249445	8.247738	Non-Canc	HS751T	Fibroblast
ACH-0014	3.554589	5.995032	Colorectal	C84	Bowel
ACH-0005	5.197315	6.868884	Diffuse Gli	TM31	CNS/Brain
ACH-0011	4.812498	6.423242	Ewing Sarc	SKNEP1	Bone
ACH-0000	3.249445	6.51428	Acute Mye	MV411	Myeloid
ACH-0007	3.764474	6.753284	Bladder Ur	UBLC1	Bladder/Urinary Tract
ACH-0009	4.489928	6.986183	Lung Neur	NCIH2286	Lung
ACH-0005	4.273516	7.894636	Non-Canc	T173	Fibroblast
ACH-0005	4.676944	6.535897	Lung Neur	CORL88	Lung
ACH-0005	3.489286	7.744498	Hepatocel	JHH2	Liver
ACH-0007	4.052242	7.207014	Invasive Br	MDAMB23	Breast
ACH-0006	4.313971	6.156639	T-Lympho	RPMI8402	Lymphoid
ACH-0006	3.134221	5.860715	Non-Smal	HCC44	Lung
ACH-0009	3.260026	7.719183	Bladder Ur	5637	Bladder/Urinary Tract
ACH-0014	2.969012	5.427271	Intraducta	EGI1	Biliary Tract
ACH-0003	4.079805	6.737281	Acute Mye	TF1	Myeloid
ACH-0025	3.292782	6.006971	Melanoma	M040416	Skin
ACH-0013	4.563768	6.676944	Neuroblas	NB1643	Peripheral Nervous System
ACH-0000	3.473787	6.717951	Bladder Ur	253JBV	Bladder/Urinary Tract
ACH-0008	3.206331	5.547203	Mature B-	AMO1	Lymphoid
ACH-0011	5.004501	6.150763	Diffuse Gli	U251MGD	CNS/Brain
ACH-0008	3.707083	6.337176	Melanoma	SKMEL24	Skin
ACH-0005	3.681449	7.408967	Non-Canc	RS5	Fibroblast
ACH-0003	3.921246	6.8212	Breast Duc	HCC1500	Breast
ACH-0005	2.726831	5.464342	Ovarian Ep	OVISE	Ovary/Fallopian Tube
ACH-0002	3.754888	6.489607	Ewing Sarc	EWS502	Bone
ACH-0005	5.574102	6.977967	Chondros	CAL78	Bone
ACH-0008	3.611172	7.170326	Melanoma	COLO783	Skin
ACH-0031	4.957915	7.068241	Non-Canc	ABMT082	Lung
ACH-0013	4.90641	6.667608	Neuroblas	CHLA15	Peripheral Nervous System
ACH-0005	3.331992	5.414812	Ovarian Ep	KURAMO(	Ovary/Fallopian Tube
ACH-0006	3.673556	6.302319	Mature B-	SUDHL6	Lymphoid
ACH-0004	3.765535	8.023921	Renal Cell	769P	Kidney
ACH-0007	3.629939	8.219265	Non-Smal	NCIH2291	Lung
ACH-0009	2.980025	7.086508	Well-Diffe	FTC133	Thyroid

ACH-0020	4.74685	7.710118	Gestationa	T3M3	Uterus
ACH-0004	3.253989	6.49041	Renal Cell	CAKI1	Kidney
ACH-0004	3.72792	6.33539	Ewing Sarc	EW8	Bone
ACH-0002	2.91265	6.181103	Ovarian Ep	COV362	Ovary/Fallopian Tube
ACH-0002	3.72465	6.305788	Colorectal	COLO320	Bowel
ACH-0017	4.493775	5.828581	B-Lympho	HB1119	Lymphoid
ACH-0013	3.891419	6.460579	Pancreatic	PACADD1	Pancreas
ACH-0020	4.712045	6.678635	Meningoth	HKBMM	CNS/Brain
ACH-0001	4.029453	6.564683	Diffuse Gli	LN319	CNS/Brain
ACH-0006	4.077243	7.698496	Ovarian Ep	OVCAR8	Ovary/Fallopian Tube
ACH-0000	3.914565	6.683275	Ewing Sarc	RDES	Bone
ACH-0002	3.858976	6.95245	Non-Canc	HS675T	Fibroblast
ACH-0007	3.634593	7.564683	Non-Smal	NCIH1703	Lung
ACH-0013	3.682573	7.068456	Anaplastic	8505C	Thyroid
ACH-0003	4.810443	7.060372	Neuroblas	SKNFI	Peripheral Nervous System
ACH-0017	5.463688	6.814166	Rhabdomy	RH28	Soft Tissue
ACH-0004	3.560715	7.305332	Pancreatic	PANC0813	Pancreas
ACH-0013	2.627607	5.249066	Invasive Br	SUM190PT	Breast
ACH-0001	4.244126	7.570918	Neuroblas	CHP212	Peripheral Nervous System
ACH-0016	5.104337	5.874059	Head and	UPCISCC0	Head and Neck
ACH-0015	2.825786	6.084277	Head and	KON	Head and Neck
ACH-0025	5.355087	6.580748	Melanoma	WM4235	Skin
ACH-0004	3.775051	7.05159	Non-Smal	LU65	Lung
ACH-0013	4.519793	6.119979	Neuroblas	NGP	Peripheral Nervous System
ACH-0006	3.553361	6.337354	Mature B-	JJN3	Lymphoid
ACH-0008	2.961623	6.771357	Bladder Sc	SCABER	Bladder/Urinary Tract
ACH-0006	4.01078	8.284014	Diffuse Gli	LN443	CNS/Brain
ACH-0015	3.831877	4.347666	Cutaneous	HSC5	Skin
ACH-0000	4.255501	6.775314	Hodgkin L	HS611T	Lymphoid
ACH-0031	4.659925	6.792725	Non-Canc	ABMT0558	Muscle
ACH-0003	3.754888	7.517591	Diffuse Gli	42MGBA	CNS/Brain
ACH-0000	3.559492	6.393862	Non-Smal	NCIH1693	Lung
ACH-0018	3.719183	6.75007	Intraducta	TKKK	Biliary Tract
ACH-0009	4.320485	8.302365	Non-Smal	NCIH1155	Lung
ACH-0001	3.641546	6.418021	Mature B-	JVM2	Lymphoid
ACH-0031	4.509696	7.010668	Non-Canc	ABMT079[illegible]	Lung
ACH-0007	3.730096	6.485588	Hepatocel	JHH5	Liver
ACH-0008	3.720278	6.464668	Non-Smal	NCIH1373	Lung
ACH-0019	3.716991	5.784766	Melanoma	NZM3	Skin
ACH-0007	3.593354	6.987207	Melanoma	SKMEL5	Skin
ACH-0013	4.508429	6.201634	Cervical Sc	C4I	Cervix
ACH-0006	4.025915	4.750607	Lung Neu	DMS53	Lung
ACH-0008	3.65764	5.882888	Bladder U	BFTC905	Bladder/Urinary Tract
ACH-0018	4.517276	6.20183	Intraducta	ICC108	Biliary Tract
ACH-0005	4.58376	6.082788	Invasive Br	MDAMB43	Breast
ACH-0016	3.581351	6.265287	Head and	UPCISCC1	Head and Neck
ACH-0009	3.340562	6.912291	Colorectal	CCK81	Bowel
ACH-0015	3.656496	6.350144	Melanoma	MM383	Skin
ACH-0016	3.876762	6.054848	Esophago	SKGT2	Esophagus/Stomach
ACH-0008	3.346248	5.74443	Non-Smal	NCIH1435	Lung
ACH-0018	4.235727	6.761684	Ampullary	TGBC52TK	Ampulla of Vater
ACH-0031	4.147307	6.468909	Non-Canc	ABMT0794	Lung
ACH-0006	4.035624	7.692302	Melanoma	WM1799	Skin
ACH-0015	4.297191	7.159569	Esophago	FLO1	Esophagus/Stomach
ACH-0011	5.066089	5.294253	Melanoma	SKMEL2	Skin
ACH-0024	4.604664	7.309976	Non-Canc	RPE1SS51	Eye
ACH-0003	4.203984	7.198101	Osteosarc	U2OS	Bone
ACH-0004	3.135863	5.225738	Non-Smal	NCIH1666	Lung
ACH-0009	4.055716	6.574404	Esophago	SNU1	Esophagus/Stomach

ACH-0007	3.845992	6.818518	Invasive Br	CAMA1	Breast
ACH-0020	4.438958	5.729281	Head and	HSQ89	Head and Neck
ACH-0003	3.773996	6.281327	Myeloprol	LAMA84	Myeloid
ACH-0024	4.357552	5.479295	Melanoma	HT144SKIN	Skin
ACH-0009	2.636915	7.386035	Intraducta	HUCCT1	Biliary Tract
ACH-0015	4.824768	7.060696	Cervical A	HCSC1	Cervix
ACH-0013	5.177519	5.990274	Neuroblas	LAN2	Peripheral Nervous System
ACH-0016	4.290572	5.378165	Melanoma	SEKI	Skin
ACH-0031	4.234961	7.51025	Non-Canc	ABMT028(	CNS/Brain
ACH-0008	4.328406	6.275193	Non-Smal	CHAGOK1	Lung
ACH-0003	4.422906	6.306882	Melanoma	WM115	Skin
ACH-0020	4.537917	6.121637	Osteosarc	HSOS1	Bone
ACH-0009	4.314697	5.98823	Melanoma	HMCB	Skin
ACH-0013	5.035624	7.137401	Ovarian G	PA1	Ovary/Fallopian Tube
ACH-0003	2.678072	5.965553	Endometri	SNU1077	Uterus
ACH-0003	3.443607	5.878971	Esophagea	ECGI10	Esophagus/Stomach
ACH-0000	3.564378	5.795715	Myeloprol	MEG01	Myeloid
ACH-0007	3.815575	6.612942	Non-Smal	NCIH1648	Lung
ACH-0005	3.927896	7.482687	Rhabdoid	NCIH2004	Kidney
ACH-0003	3.708187	6.874059	Non-Smal	NCIH522	Lung
ACH-0014	2.575312	6.683977	Non-Semi	1618K	Testis
ACH-0001	3.782409	7.006522	Renal Cell	VMRCRCZ	Kidney
ACH-0001	3.791814	6.84549	Endometri	JHUEM3	Uterus
ACH-0005	3.040892	6.341452	Pancreatic	BXPC3	Pancreas
ACH-0007	3.028569	6.411257	Non-Smal	RERFLCAD	Lung
ACH-0001	2.91265	6.509854	Pancreatic	HS766T	Pancreas
ACH-0013	2.942984	7.06415	Anaplastic	8305C	Thyroid
ACH-0009	4.076388	6.717539	Endometri	HEC59	Uterus
ACH-0011	4.782933	6.034304	Neuroblas	SHSY5Y	Peripheral Nervous System
ACH-0003	4.080658	6.811856	T-Lympho	P12ICHIKA	Lymphoid
ACH-0008	4.201634	6.716442	Neuroblas	NB1	Peripheral Nervous System
ACH-0000	3.058316	5.961855	Bladder U	253J	Bladder/Urinary Tract
ACH-0007	3.921246	7.403182	Non-Smal	NCIH1838	Lung
ACH-0016	4.257011	5.635754	Neuroblas	NH12	Peripheral Nervous System
ACH-0008	4.201634	6.90135	Mature B-	HUNS1	Lymphoid
ACH-0001	4.154616	6.467443	Intrachole	SNU308	Biliary Tract
ACH-0008	3.215679	7.053872	Melanoma	SKMEL30	Skin
ACH-0011	4.219556	7.618753	Ovarian Ep	OC316	Ovary/Fallopian Tube
ACH-0007	3.68818	6.398487	Esophagea	COLO680N	Esophagus/Stomach
ACH-0007	3.524816	7.533408	Diffuse Gli	GB1	CNS/Brain
ACH-0018	3.893362	5.999098	Intraducta	ICC3	Biliary Tract
ACH-0026	4.296457	6.340028	Colorectal	JVE253	Bowel
ACH-0007	3.451541	6.79831	Lung Neur	NCIH1105	Lung
ACH-0015	5.254745	6.543651	Neuroblas	LS	Peripheral Nervous System
ACH-0007	3.918386	6.790642	Invasive Br	MDAMB17	Breast
ACH-0015	4.319762	6.450386	Pleural Me	MERO14	Pleura
ACH-0013	3.390943	5.856239	Ovarian Ep	OCIC5X	Ovary/Fallopian Tube
ACH-0009	3.877744	6.063503	Colorectal	KM12	Bowel
ACH-0008	3.058316	5.338068	Esophagea	KYSE270	Esophagus/Stomach
ACH-0012	4.563768	6.402415	Synovial S	SYO1	Soft Tissue
ACH-0026	4.66562	6.117903	Colorectal	JVE015	Bowel
ACH-0009	4.31034	6.61574	Non-Smal	NCIH157D	Lung
ACH-0013	4.214902	5.150966	Ovarian Ep	OV17R	Ovary/Fallopian Tube
ACH-0028	4.536053	6.064797	Nerve She	NCCMPNS	Peripheral Nervous System
ACH-0010	4.478972	6.438293	Ewing Sarc	CBAGPN	Bone
ACH-0000	4.090853	8.270155	Osteosarc	G292CLON	Bone
ACH-0004	2.316146	6.586915	Esophagea	TE11	Esophagus/Stomach
ACH-0007	3.157044	6.627607	Lung Neur	SHP77	Lung
ACH-0001	4.162693	6.502076	Rhabdoid	TM87	Kidney

ACH-0006	4.145677	7.608588	Esophagea	TE1	Esophagus/Stomach
ACH-0006	4.211791	7.137504	Acute Mye	M07E	Myeloid
ACH-0001	4.498251	6.614415	B-Lympho	MHHCALL	Lymphoid
ACH-0020	4.270529	4.637494	Melanoma	A375SKIN	Skin
ACH-0003	4.739848	5.978882	Mature B-	KARPAS42	Lymphoid
ACH-0024	2.608809	6.319943	Esophago	CCLFUPGI	Esophagus/Stomach
ACH-0002	3.190615	7.01402	Rhabdoid	A204	Kidney
ACH-0004	2.260026	5.887769	Endometri	EFE184	Uterus
ACH-0009	4.187451	6.85437	Endometri	RL952	Uterus
ACH-0015	5.372604	7.224773	Merkel Ce	MCC13	Skin
ACH-0020	4.776104	7.138835	Endometri	HHUA	Uterus
ACH-0008	3.921246	7.193279	Diffuse Gli	SW1783	CNS/Brain
ACH-0004	3.678072	5.834913	Non-Smal	RERFLCKJ	Lung
ACH-0004	3.49057	6.781884	Melanoma	MELHO	Skin
ACH-0026	5.368768	6.532784	Pancreatic	MAPACHS	Pancreas
ACH-0008	3.275007	6.531069	Undifferer	GCT	Soft Tissue
ACH-0018	4.244126	4.557042	Liposarcor	LPS853	Soft Tissue
ACH-0006	3.858976	7.058316	Meningotl	IOMMLEE	CNS/Brain
ACH-0007	4.300124	6.627899	Head and	SNU1214	Head and Neck
ACH-0008	4.329124	6.770961	Lung Neu	NCIH1930	Lung
ACH-0009	3.620586	6.809414	Invasive B	MDAMB45	Breast
ACH-0007	3.157044	6.53512	Non-Smal	PC9	Lung
ACH-0001	3.499527	6.405141	Mature B-	PFEIFFER	Lymphoid
ACH-0011	4.64501	6.164907	Rhabdomy	SMSCTR	Soft Tissue
ACH-0014	4.293518	5.320485	B-Lympho	CI	Lymphoid
ACH-0006	3.157044	6.398145	Esophagea	KYSE180	Esophagus/Stomach
ACH-0008	3.742006	7.862513	Ovarian Ep	SKOV3	Ovary/Fallopian Tube
ACH-0004	3.62527	7.147815	Melanoma	SKMEL3	Skin
ACH-0002	4.350497	7.516488	Lung Neu	NCIH209	Lung
ACH-0018	4.074677	6.56514	Intraducta	ICC7	Biliary Tract
ACH-0004	4.281698	6.618092	B-Lympho	MUTZ5	Lymphoid
ACH-0015	4.065228	6.441284	Chondrosa	HEMCSS	Bone
ACH-0001	4.118526	5.543805	Esophago	NCCSTCK1	Esophagus/Stomach
ACH-0014	3.587365	6.431623	Esophago	ESO51	Esophagus/Stomach
ACH-0001	4.580145	7.57342	Diffuse Gli	GMS10	CNS/Brain
ACH-0008	3.068671	5.763146	Mature B-	RPMI8226	Lymphoid
ACH-0015	5.040454	7.432124	Pleural Me	MERO25	Pleura
ACH-0009	4.200065	5.992542	Mature T a	MOLT3	Lymphoid
ACH-0003	3.809414	6.486232	Non-Smal	NCIH647	Lung
ACH-0009	4.311794	6.876762	Uterine Sa	ESS1	Uterus
ACH-0005	3.982765	8.373735	Non-Smal	HCC364	Lung
ACH-0006	3.911692	6.729281	Diffuse Gli	LN340	CNS/Brain
ACH-0022	3.447579	6.451376	Non-Semi	NTERA2CL	Testis
ACH-0013	4.732812	6.966707	Synovial S	CME1	Soft Tissue
ACH-0007	4.189034	6.433794	Lung Neu	NCIH526	Lung
ACH-0016	2.214125	4.078097	Non-Hodg	SCC3	Lymphoid
ACH-0015	4.265287	6.489125	Merkel Ce	MCC26	Skin
ACH-0004	3.344828	6.958147	Non-Smal	NCIH2085	Lung
ACH-0008	3.58376	7.307702	Endometri	MFE296	Uterus
ACH-0018	4.478325	5.862947	Intraducta	ICC2	Biliary Tract
ACH-0010	4.366672	5.690976	Ewing Sarc	CHLA32	Bone
ACH-0009	4.144046	7.276962	Colorectal	GP2D	Bowel
ACH-0016	2.849999	6.509062	Mature B-	RAMOS	Lymphoid
ACH-0001	2.881665	7.666402	Renal Cell	RCC10RGE	Kidney
ACH-0004	3.582556	6.517118	Non-Smal	HCC95	Lung
ACH-0001	4.242603	8.033698	Non-Canc	HS229T	Fibroblast
ACH-0006	4.293518	6.672567	Mature B-	SUDHL5	Lymphoid
ACH-0011	5.480588	6.527321	Non-Hodg	SMZ1	Lymphoid
ACH-0009	3.709291	7.258802	Melanoma	IPC298	Skin

ACH-0004	3.646163	7.080977	Hepatocel	HUH7	Liver
ACH-0007	2.987321	5.615887	Mature B-	MOLP8	Lymphoid
ACH-0001	3.670161	6.290019	Non-Smal	CORL105	Lung
ACH-0003	3.680324	6.273143	Non-Smal	NCIH520	Lung
ACH-0014	2.510962	5.592158	Colorectal	C80	Bowel
ACH-0009	4.185867	6.14323	Non-Smal	NCIH1573	Lung
ACH-0016	4.646163	6.779128	Diffuse Gli	NP3	CNS/Brain
ACH-0019	3.87578	6.528415	Intraducta	CCSW1	Biliary Tract
ACH-0018	2.865919	6.78398	Intraducta	RBE	Biliary Tract
ACH-0031	4.363872	7.382667	Non-Canc	ABMT922(	Muscle
ACH-0002	4.512859	6.552439	Myeloprol	EM2	Myeloid
ACH-0014	3.582556	5.421223	B-Lymphc	Cll	Lymphoid
ACH-0002	3.892391	6.538538	Lung Neu	NCIH841	Lung
ACH-0001	4.123501	5.964399	Diffuse Gli	8MGBA	CNS/Brain
ACH-0003	3.925999	5.558574	Ovarian Ep	EFO21	Ovary/Fallopian Tube
ACH-0004	3.545968	7.315421	Diffuse Gli	KNS60	CNS/Brain
ACH-0012	3.305971	6.617798	Head and	UPCISCC1	Head and Neck
ACH-0001	4.176323	6.124328	Esophago	RERFGC1E	Esophagus/Stomach
ACH-0008	3.766595	6.374866	Non-Smal	NCIH322	Lung
ACH-0000	4.092546	6.400367	Ovarian Ep	TOV112D	Ovary/Fallopian Tube
ACH-0004	2.835924	6.287066	Non-Smal	NCIH460	Lung
ACH-0008	3.871844	7.407013	Melanoma	MELJUSO	Skin
ACH-0003	2.577731	6.060264	Mature B-	NUDHL1	Lymphoid
ACH-0019	4.963012	6.705978	Pleural Me	ONE58	Pleura
ACH-0001	3.456806	5.694602	Invasive Br	T47D	Breast
ACH-0009	3.794936	7.491853	Invasive Br	HCC1569	Breast
ACH-0008	4.534186	6.704595	Melanoma	WM88	Skin
ACH-0003	4.431623	7.189429	Neuroblas	SKNDZ	Peripheral Nervous System
ACH-0015	3.691534	6.527164	Osteosarc	HUO9	Bone
ACH-0000	4.528571	5.720005	Non-Canc	SALE	Lung
ACH-0013	4.854494	6.310522	Diffuse Gli	ANGMCSS	CNS/Brain
ACH-0004	3.720278	7.312883	Chondros	SW1353	Bone
ACH-0012	5.201242	6.506526	Synovial S	SCS214	Soft Tissue
ACH-0029	4.336997	6.161888	Neuroblas	SKNMM	Peripheral Nervous System
ACH-0008	4.132577	5.613826	Non-Smal	HOP62	Lung
ACH-0006	3.171527	5.812755	Head and	SNU899	Head and Neck
ACH-0001	4.08151	6.769243	Pancreatic	CAPAN2	Pancreas
ACH-0001	4.413459	7.280864	Rhabdomy	HS729	Soft Tissue
ACH-0003	3.984589	7.778997	Non-Canc	HS688AT	Fibroblast
ACH-0015	4.832384	8.11369	Gestationa	JEG3	Uterus
ACH-0006	2.887525	6.96359	Colorectal	SW620	Bowel
ACH-0005	3.684819	7.079378	Non-Smal	EBC1	Lung
ACH-0000	3.840967	6.924694	Well-Diffe	ML1	Thyroid
ACH-0003	4.785027	7.73098	Neuroblas	KPNRTBM	Peripheral Nervous System
ACH-0003	4.475733	7.068026	Hepatocel	SNU886	Liver
ACH-0008	3.934517	6.297742	Melanoma	MDAMB43	Skin
ACH-0002	2.903038	7.302776	Diffuse Gli	SNU466	CNS/Brain
ACH-0014	3.244887	7.297558	Cervical Sc	BOKU	Cervix
ACH-0003	3.746313	6.239551	Lung Neu	NCIH82	Lung
ACH-0029	4.502076	4.792335	Ocular Me	UPMD1	Eye
ACH-0009	3.367371	6.501758	Ovarian Ep	OC314	Ovary/Fallopian Tube
ACH-0004	3.229588	6.65764	Bladder Ur	RT112	Bladder/Urinary Tract
ACH-0001	3.905928	6.195544	Endometri	MFE280	Uterus
ACH-0016	3.806324	6.764606	Diffuse Gli	ONDA9	CNS/Brain
ACH-0018	4.975905	6.703765	Liposarcor	LPS067	Soft Tissue
ACH-0024	3.785551	6.516173	Non-Canc	RPE1SS11	Eye
ACH-0013	4.8166	5.28762	Neuroblas	GIMEN	Peripheral Nervous System
ACH-0007	3.800123	6.583158	Non-Smal	A427	Lung
ACH-0000	4.22265	7.2538	Fibrosarcc	HT1080	Soft Tissue

ACH-0013	3.938286	7.133707	Hepatocel	PLCPRF5	Liver
ACH-0006	2.226509	6.456806	Pancreatic	MIAPACA2	Pancreas
ACH-0006	3.486714	6.682995	Ovarian Ep	SNU119	Ovary/Fallopian Tube
ACH-0018	3.353323	7.417431	Intraducta	SG231	Biliary Tract
ACH-0009	3.726831	7.650908	Ovarian Ep	IGROV1	Ovary/Fallopian Tube
ACH-0006	4.484783	6.81724	Head and	YD8	Head and Neck
ACH-0002	4.030336	6.903882	Esophago	ECC12	Esophagus/Stomach
ACH-0006	3.879706	6.907732	Ovarian Ep	A2780	Ovary/Fallopian Tube
ACH-0006	3.056584	5.175924	Breast Duc	HCC1806	Breast
ACH-0006	4.709842	7.657354	Invasive Br	HCC1395	Breast
ACH-0009	4.018812	6.1637	Mature B-	BCP1	Lymphoid
ACH-0006	3.834913	7.015694	Non-Hodg	CI1	Lymphoid
ACH-0000	4.331275	5.611763	Invasive Br	MDAMB13	Breast
ACH-0007	3.206331	6.946497	Head and	A253	Head and Neck
ACH-0004	4.702658	6.111657	Non-Smal	NCIH2228	Lung
ACH-0001	4.260026	5.365623	Mature T a	KE37	Lymphoid
ACH-0014	4.327687	7.052025	Bladder Ur	UMUC9	Bladder/Urinary Tract
ACH-0003	4.211012	7.078524	Pleural Me	MPP89	Pleura
ACH-0001	4.262283	6.317232	Myeloprol	EOL1	Myeloid
ACH-0002	3.01078	5.847997	Ovarian Ep	SNU840	Ovary/Fallopian Tube
ACH-0001	5.171927	7.089689	Mature T a	LOUCY	Lymphoid
ACH-0018	3.738768	4.707635	Intraducta	ICC106	Biliary Tract
ACH-0008	3.918386	7.299025	Non-Smal	NCIH1563	Lung
ACH-0004	3.800123	7.20134	Hepatocel	SNU423	Liver
ACH-0000	3.432959	5.693208	Pancreatic	PANC0203	Pancreas
ACH-0020	3.815575	5.815319	Head and	HO1U1	Head and Neck
ACH-0002	4.022368	6.78725	Hepatocel	SNU398	Liver
ACH-0004	4.105175	7.401903	Melanoma	MALME3M	Skin
ACH-0007	3.69488	6.724241	Esophago	GSS	Esophagus/Stomach
ACH-0003	4.060047	5.627899	Esophago	MKN45	Esophagus/Stomach
ACH-0004	3.763412	7.059399	Renal Cell	CAL54	Kidney
ACH-0009	4.516015	7.581653	Non-Smal	NCIH2106	Lung
ACH-0031	4.934517	7.247928	Non-Canc	ABMT9233	Hair
ACH-0002	4.257765	7.1469	Diffuse Gli	DKMG	CNS/Brain
ACH-0006	4.145677	6.630231	Lung Neur	NCIH2227	Lung
ACH-0000	4.260778	6.744834	Acute Mye	GDM1	Myeloid
ACH-0031	4.734439	7.733829	Non-Canc	ABMT9430	Pancreas
ACH-0002	3.463361	7.204669	Esophago	LMSU	Esophagus/Stomach
ACH-0010	4.715344	6.211207	Ewing Sarc	COGE352	Bone
ACH-0003	3.987321	5.434295	Invasive Br	HCC1428	Breast
ACH-0020	4.891419	7.009661	Ocular Me	MEL290	Eye
ACH-0004	3.710393	5.854494	Non-Smal	NCIH838	Lung
ACH-0007	4.228049	7.870981	Lung Neur	NCIH196	Lung
ACH-0008	4.318317	6.990728	Bladder Ur	KMBC2	Bladder/Urinary Tract
ACH-0002	3.22033	6.504779	Colorectal	SW1417	Bowel
ACH-0004	3.280956	7.344296	Melanoma	COLO800	Skin
ACH-0007	3.498251	6.970163	Non-Smal	HCC2279	Lung
ACH-0008	4.572283	7.403012	Non-Smal	NCIH2347	Lung
ACH-0006	3.18428	6.579542	Mature B-	U266B1	Lymphoid
ACH-0005	3.307429	7.207893	Bladder Ur	BC3C	Bladder/Urinary Tract
ACH-0001	4.034744	6.053763	Ampullary	SNU869	Ampulla of Vater
ACH-0004	3.864929	7.543419	Non-Smal	RERFLCSQ	Lung
ACH-0001	3.496974	7.543882	Renal Cell	OSRC2	Kidney
ACH-0009	3.542258	6.366672	Renal Cell	SNU349	Kidney
ACH-0001	3.246408	5.884109	Anaplastic	BHT101	Thyroid
ACH-0002	2.718088	6.197118	Pancreatic	PK59	Pancreas
ACH-0001	3.179511	5.874059	Ovarian Ep	CAOV4	Ovary/Fallopian Tube
ACH-0007	3.107688	5.62819	Hepatobla	HEPG2	Liver
ACH-0015	4.925525	6.482364	Pleural Me	MERO48A	Pleura

ACH-0002	5.31252	6.43062	T-Lymphc	C8166	Lymphoid
ACH-0008	3.618239	6.638653	Non-Smal	NCIH2087	Lung
ACH-0006	3.277985	7.183586	Melanoma	SKMEL28	Skin
ACH-0016	4.590961	5.804002	Acute Mye	OCIAML4	Myeloid
ACH-0016	3.482848	5.899901	Diffuse Gli	NP2	CNS/Brain
ACH-0008	3.593354	8.448777	Melanoma	HS936T	Skin
ACH-0005	3.89724	7.42156	Non-Canc	HS618T	Fibroblast
ACH-0004	3.099295	6.730368	Diffuse Gli	CAS1	CNS/Brain
ACH-0006	3.50716	7.376169	Diffuse Gli	KNS42	CNS/Brain
ACH-0004	4.174726	6.680746	Esophagea	TE8	Esophagus/Stomach
ACH-0013	2.260026	6.043957	Invasive Br	SUM149PT	Breast
ACH-0014	3.998196	5.793116	Adrenocor	SW13	Adrenal Gland
ACH-0003	3.15056	6.189232	Mature B-	SUDHL4	Lymphoid
ACH-0014	4.445594	5.924813	Ewing Sarc	TC205	Bone
ACH-0004	3.160275	6.187847	Hepatocel	HUH1	Liver
ACH-0008	4.680887	7.054306	Non-Smal	NCIH358	Lung
ACH-0000	4.655352	6.361242	Myeloprol	KU812	Myeloid
ACH-0003	3.317594	7.583534	Ampullary	SNU478	Ampulla of Vater
ACH-0015	5.180307	5.098874	Pleural Me	MERO95	Pleura
ACH-0016	4.171527	6.362821	Head and	UPCISCC0	Head and Neck
ACH-0031	4.635754	7.423746	Non-Canc	ABMT0168	Unknown
ACH-0002	4.181103	7.570159	Neuroblas	KELLY	Peripheral Nervous System
ACH-0028	4.96116	6.379898	Nerve She	NCCMPNS	Peripheral Nervous System
ACH-0013	0.62293	1.757023	Invasive Br	SUM102PT	Breast
ACH-0014	4.934988	6.388362	Bladder Ur	UMUC4	Bladder/Urinary Tract
ACH-0020	3.773996	6.541948	Colorectal	TT1TKB	Bowel
ACH-0007	4.070389	7.051263	Bladder Ur	TCCSUP	Bladder/Urinary Tract
ACH-0000	4.794416	5.837691	Embryona	D341Med	CNS/Brain
ACH-0013	4.241077	7.043738	Colorectal	GP5D	Bowel
ACH-0005	4.125155	7.010668	Diffuse Gli	A172	CNS/Brain
ACH-0006	4.446918	5.34802	Mature T a	SUDHL1	Lymphoid
ACH-0001	3.898208	6.699607	Prostate S	NCIH660	Prostate
ACH-0003	4.078097	6.356496	Pleural Me	MSTO211H	Pleura
ACH-0009	4.121844	6.101818	Endometri	AN3CA	Uterus
ACH-0031	4.571677	7.036174	Non-Canc	ABMT9249	Hair
ACH-0006	4.090007	6.559492	Diffuse Gli	KS1	CNS/Brain
ACH-0002	3.480265	7.162492	Head and	DETROIT5	Head and Neck
ACH-0007	3.381283	7.088417	Esophago	SH10TC	Esophagus/Stomach
ACH-0009	4.372952	6.438792	T-Lymphc	JURKAT	Lymphoid
ACH-0003	3.817623	6.735251	Acute Mye	SKM1	Myeloid
ACH-0009	5.454176	6.927659	T-Lymphc	SUPT1	Lymphoid
ACH-0007	3.258519	6.555049	Renal Cell	KMRC2	Kidney
ACH-0008	4.149747	5.960465	Head and	FADU	Head and Neck
ACH-0017	4.402586	6.034524	Epithelioic	VAESBJ	Soft Tissue
ACH-0003	3.947666	6.759156	Ovarian Ep	JHOM2B	Ovary/Fallopian Tube
ACH-0020	5.698774	5.941106	B-Lymphc	P30OHK	Lymphoid
ACH-0009	3.569248	6.698218	Non-Smal	NCIH2110	Lung
ACH-0013	2.107688	4.927896	Invasive Br	SUM44PE	Breast
ACH-0009	3.697107	7.012569	Esophago	2313287	Esophagus/Stomach
ACH-0020	4.702103	5.546586	Ocular Me	MEL285	Eye
ACH-0009	3.992768	6.930619	T-Lymphc	DND41	Lymphoid
ACH-0006	2.596935	6.98823	Ovarian Ep	OVCAR4	Ovary/Fallopian Tube
ACH-0003	3.102658	5.337354	Non-Smal	NCIH2122	Lung
ACH-0012	3.936402	7.533797	Rhabdoid	TTC1240	Kidney
ACH-0018	4.185867	7.142924	Intraducta	ICC10	Biliary Tract
ACH-0031	4.330558	7.438293	Non-Canc	ABMT0616	Kidney
ACH-0003	3.675816	5.741467	Non-Smal	LUDLU1	Lung
ACH-0004	4.42156	7.281791	Pancreatic	PK45H	Pancreas
ACH-0010	4.717539	6.784635	Colorectal	DLD1	Bowel

ACH-0000	4.191405	7.270529	Non-Hodg	MJ	Lymphoid
ACH-0004	3.911692	4.543496	Poorly Diff	BCPAP	Thyroid
ACH-0004	3.655352	6.780048	Non-Smal	NCIH1792	Lung
ACH-0025	4.464015	6.489447	Melanoma	MM16011	Skin
ACH-0000	3.476382	6.399171	Non-Smal	RERFLCMS	Lung
ACH-0024	4.937344	6.763412	Non-Canc	RPE1SS6	Eye
ACH-0000	4.820179	7.621979	Non-Canc	HS172T	Fibroblast
ACH-0005	4.860963	7.021369	Invasive Br	BT20	Breast
ACH-0000	4.208673	8.080764	Non-Canc	HS706T	Fibroblast
ACH-0020	4.512227	4.760221	Melanoma	UACC62SI	Skin
ACH-0008	2.891419	6.196725	Non-Smal	HCC15	Lung
ACH-0013	4.169925	5.320124	Pancreatic	PACADD1	Pancreas
ACH-0020	4.172327	6.041331	Myeloprol	P2URK562	Myeloid
ACH-0003	3.491853	6.186065	Renal Cell	SKRC52	Kidney
ACH-0004	3.308885	7.170125	Colorectal	SNU1197	Bowel
ACH-0004	3.646163	6.480588	Esophago	NCIN87	Esophagus/Stomach
ACH-0009	3.952334	6.608809	B-Lympho	NALM6	Lymphoid
ACH-0004	4.431623	6.516646	Myeloprol	NALM1	Myeloid
ACH-0009	4.148934	8.24522	Uterine Sa	SKUT1	Uterus
ACH-0003	3.72792	7.577504	Esophago	SNU620	Esophagus/Stomach
ACH-0005	4.689299	6.933573	Lung Neu	HCC33	Lung
ACH-0010	5.003152	6.388706	Embryona	CHLA57	CNS/Brain
ACH-0009	3.710393	6.641835	Esophago	IM95	Esophagus/Stomach
ACH-0008	3.498251	6.875903	Diffuse Gli	DBTRG05M	CNS/Brain
ACH-0006	4.141596	5.433293	Non-Canc	HMEL	Breast
ACH-0005	3.244887	7.03166	Esophago	SNU16	Esophagus/Stomach
ACH-0010	4.446918	6.618239	Rhabdomy	CW9019	Soft Tissue
ACH-0018	4.739848	6.375213	Osteosarc	C396	Bone
ACH-0013	2.508429	6.820945	Well-Diffe	RO82W1	Thyroid
ACH-0007	3.764474	6.900384	Non-Canc	HLFA	Fibroblast
ACH-0002	4.324811	6.946848	Mature B-	MINO	Lymphoid
ACH-0015	2.737687	5.712871	Mature B-	KARPAS17	Lymphoid
ACH-0015	4.833396	5.795455	Head and	H314	Head and Neck
ACH-0008	3.704872	7.082043	Melanoma	WM793	Skin
ACH-0009	4.311794	6.485427	Endometri	ISHIKAWA	Uterus
ACH-0007	3.420887	7.015917	Head and	HSC3	Head and Neck
ACH-0008	3.028569	6.229396	Non-Smal	HOP92	Lung
ACH-0007	3.639232	7.735996	Lung Neu	CORL95	Lung
ACH-0001	3.61471	6.228241	Acute Mye	HDMYZ	Myeloid
ACH-0018	4.123501	6.10706	Invasive Br	MFM223	Breast
ACH-0001	4.145677	6.180904	Mature B-	A4FUK	Lymphoid
ACH-0017	3.856986	7.123501	Colorectal	SNU1544	Bowel
ACH-0002	3.803227	7.400965	Colorectal	COLO201	Bowel
ACH-0013	2.456806	5.480588	Pancreatic	PACADD1	Pancreas
ACH-0009	4.459432	6.560256	Colorectal	SW48	Bowel
ACH-0002	3.01614	6.882521	Head and	BICR31	Head and Neck
ACH-0002	3.909773	7.127943	Pancreatic	SNU213	Pancreas
ACH-0017	5.011675	6.774787	Rhabdomy	TTC442	Soft Tissue
ACH-0001	3.523562	6.654206	Anaplastic	CAL62	Thyroid
ACH-0002	3.669027	7.467524	Mature B-	BL41	Lymphoid
ACH-0012	5.030336	6.098874	Ovarian Ep	SCCOHT1	Ovary/Fallopian Tube
ACH-0031	4.24184	5.749802	Non-Canc	ABMT073	Unknown
ACH-0006	3.491853	6.750874	Hepatocel	JHH1	Liver
ACH-0008	3.808385	5.911212	Non-Smal	T3M10	Lung
ACH-0015	4.050502	5.05398	Cervical A	HCA1	Cervix
ACH-0001	3.990047	6.476868	Acute Mye	NOMO1	Myeloid
ACH-0002	3.200065	7.14425	Lung Neu	CORL279	Lung
ACH-0002	3.200065	7.088417	Embryona	DAOY	CNS/Brain
ACH-0002	3.568032	7.023255	Diffuse Gli	A1207	CNS/Brain

ACH-0020	4.550901	5.189825	Anaplastic	HOTHC	Thyroid
ACH-0009	3.971773	6.589164	Melanoma	MEWO	Skin
ACH-0006	3.609991	5.82222	Head and	SNU1066	Head and Neck
ACH-0000	4.177918	5.92077	Mature B-	GRANTA5	Lymphoid
ACH-0002	3.510962	5.944156	Invasive Br	CAL120	Breast
ACH-0000	3.509696	7.412443	Rhabdoid	G401	Kidney
ACH-0000	4.244887	7.700648	Non-Canc	HS616T	Fibroblast
ACH-0019	4.072963	5.770565	Pancreatic	9505BIK	Pancreas
ACH-0007	3.153805	5.415488	Embryona	ONS76	CNS/Brain
ACH-0005	3.565597	6.932392	Non-Smal	NCIH2030	Lung
ACH-0002	5.072963	6.113325	Acute Mye	HNT34	Myeloid
ACH-0000	3.431623	6.333066	Meningotl	CH157MN	CNS/Brain
ACH-0005	3.788686	6.91265	Diffuse Gli	LN235	CNS/Brain
ACH-0017	4.656496	6.364748	Neuroblas	GOTO	Peripheral Nervous System
ACH-0008	3.454176	7.151575	Rhabdomy	RH30	Soft Tissue
ACH-0016	5.313246	6.600656	Ovarian Ep	PEA1	Ovary/Fallopian Tube
ACH-0003	3.386811	6.753952	Mature B-	RI1	Lymphoid
ACH-0002	3.896272	6.871597	Melanoma	HS852T	Skin
ACH-0013	2.757023	5.333424	Ovarian Ep	OCIP5X	Ovary/Fallopian Tube
ACH-0000	4.172327	5.658497	Non-Smal	HCC827GI	Lung
ACH-0018	3.040892	5.768184	Intraducta	ICC9	Biliary Tract
ACH-0006	3.972693	6.001352	Melanoma	COLO829	Skin
ACH-0014	4.669027	5.098874	Prostate A	WPE1NA2	Prostate
ACH-0005	3.588565	7.613753	Mature B-	KMS26	Lymphoid
ACH-0008	3.850999	7.970163	Invasive Br	MDAMB46	Breast
ACH-0002	3.537296	5.64299	Melanoma	A375	Skin
ACH-0001	4.020591	6.979797	Non-Smal	HCC2935	Lung
ACH-0018	3.833902	6.367546	Intraducta	ICC5	Biliary Tract
ACH-0003	4.040892	5.749802	Bladder Ur	SW780	Bladder/Urinary Tract
ACH-0010	4.198494	4.517276	Embryona	D425	CNS/Brain
ACH-0012	5.489607	6.083639	Synovial S	SW982	Soft Tissue
ACH-0000	3.485427	6.836934	Ovarian Ep	OV56	Ovary/Fallopian Tube
ACH-0020	4.286881	4.517276	Ocular Me	MEL270	Eye
ACH-0016	2.014355	5.780835	Mature B-	PGA1	Lymphoid
ACH-0025	4.947666	5.822475	Non-Smal	SNU2535	Lung
ACH-0004	3.776104	6.226894	Renal Cell	TUHR4TKE	Kidney
ACH-0009	4.045268	6.704595	Esophago	NUGC3	Esophagus/Stomach
ACH-0000	3.458119	6.561326	Ovarian Ep	ONCODG1	Ovary/Fallopian Tube
ACH-0007	3.206331	5.375387	Esophagea	KYSE30	Esophagus/Stomach
ACH-0017	4.145677	5.67638	Rhabdomy	RH4	Soft Tissue
ACH-0003	4.24184	7.388792	Neuroblas	SKNBE2	Peripheral Nervous System
ACH-0000	3.81455	5.346248	Pancreatic	T3M4	Pancreas
ACH-0004	2.488001	4.193772	Mature B-	OCIMY7	Lymphoid
ACH-0008	3.874797	6.452694	Non-Smal	NCIH1568	Lung
ACH-0002	4.459432	7.106327	Hodgkin L	HDLM2	Lymphoid
ACH-0004	1.807355	5.802969	Colorectal	SW1116	Bowel
ACH-0004	3.894333	6.898813	Lung Neur	NCIH1694	Lung
ACH-0001	4.111866	7.563234	Diffuse Gli	M059K	CNS/Brain
ACH-0009	4.058316	6.95977	Endometri	JHUEM2	Uterus
ACH-0005	3.065228	5.839708	Mature B-	WSUDLCL	Lymphoid
ACH-0005	3.327687	6.317955	Melanoma	COLO741	Skin
ACH-0004	3.189034	6.096768	Ovarian Ep	SNU8	Ovary/Fallopian Tube
ACH-0004	3.949535	5.76288	Ovarian Ep	OVKATE	Ovary/Fallopian Tube
ACH-0015	4.603478	6.422065	Melanoma	MM426	Skin
ACH-0009	2.680324	6.276311	Colorectal	HT115	Bowel
ACH-0013	4.038261	6.923268	Cervical Sc	C4II	Cervix
ACH-0000	3.818646	6.311249	Pancreatic	PATU8988	Pancreas
ACH-0020	4.331275	5.313609	Mature B-	SLVL	Lymphoid
ACH-0024	4.74685	7.097084	Non-Canc	RPE1SS48	Eye

ACH-0001	4.50716	6.876885	B-Lympho	JM1	Lymphoid
ACH-0012	4.148934	6.348905	Synovial S	YAMATO	Soft Tissue
ACH-0006	3.956986	7.039577	Rhabdomy	KYM1	Soft Tissue
ACH-0026	3.91934	6.32355	Intraducta	CCC5	Biliary Tract
ACH-0007	4.423578	6.716168	Lung Neu	DMS79	Lung
ACH-0012	4.441616	5.491853	Rhabdoid	TTC549	Kidney
ACH-0031	3.799087	7.725741	Non-Canc	ABMT079(	Lung
ACH-0015	3.364572	5.304146	Head and	H413	Head and Neck
ACH-0001	4.221104	6.171927	Acute Mye	OCIAML2	Myeloid
ACH-0003	4.616475	5.954662	Non-Smal	NCIH3122	Lung
ACH-0005	3.797013	6.007644	Mature B-	KMS21BM	Lymphoid
ACH-0000	4.122673	6.313609	Mature B-	OPM1	Lymphoid
ACH-0002	3.547203	6.680746	Pancreatic	KP2	Pancreas
ACH-0003	3.301588	5.817879	Myeloprol	MOLM6	Myeloid
ACH-0009	3.533563	6.956986	Colorectal	LS411N	Bowel
ACH-0004	4.095924	6.803485	Non-Smal	LU99	Lung
ACH-0003	3.519793	5.957218	Pancreatic	QGP1	Pancreas
ACH-0013	3.786596	7.079591	Colorectal	SW626	Bowel
ACH-0010	1.735522	5.250962	Mature T a	KARPAS38	Lymphoid
ACH-0001	3.612352	7.109987	Bladder Ur	SLR20	Bladder/Urinary Tract
ACH-0019	5.084064	6.648178	Intrachole	GB2	Biliary Tract
ACH-0017	4.608809	6.785681	Liposarcor	95T1000	Soft Tissue
ACH-0003	3.746313	7.454998	Pancreatic	PK1	Pancreas
ACH-0010	4.890447	5.212958	Ewing Sarc	CHLA218	Bone
ACH-0024	5.043519	6.547203	Mature B-	MAVER1	Lymphoid
ACH-0005	3.805292	7.622198	Diffuse Gli	LN229	CNS/Brain
ACH-0015	2.427606	4.144862	Liposarcor	KMLS1	Soft Tissue
ACH-0002	3.968091	7.22207	Lung Neu	NCIH2029	Lung
ACH-0007	2.946731	5.440288	Head and	BICR56	Head and Neck
ACH-0005	3.852998	6.663772	Head and	SNU46	Head and Neck
ACH-0003	3.825786	6.890325	Esophagea	TE15	Esophagus/Stomach
ACH-0003	3.261531	5.787903	Mature B-	SKMM2	Lymphoid
ACH-0003	4.140779	4.950468	Colorectal	COLO678	Bowel
ACH-0000	3.83289	6.848373	Mature T a	KARPAS29	Lymphoid
ACH-0015	3.537296	5.676944	Head and	KOSC2	Head and Neck
ACH-0006	3.190615	7.273982	Ovarian Ep	OVTOKO	Ovary/Fallopian Tube
ACH-0003	3.485427	5.506843	Mature B-	JVM3	Lymphoid
ACH-0009	4.283922	6.859348	Colorectal	SNUC5	Bowel
ACH-0013	5.110614	6.655066	Cervical Sc	C33A	Cervix
ACH-0007	2.295723	5.717813	Head and	BICR22	Head and Neck
ACH-0000	4.476382	7.357816	Ewing Sarc	SKNMC	Bone
ACH-0004	3.62527	6.755555	Hepatocel	LI7	Liver
ACH-0008	4.265287	7.756423	Non-Canc	HS698T	Fibroblast
ACH-0002	2.853996	6.440952	Ovarian Ep	OV90	Ovary/Fallopian Tube
ACH-0014	4.420887	5.585864	Non-Canc	BPH1	Prostate
ACH-0014	3.370164	6.363697	Colorectal	C75	Bowel
ACH-0008	3.49057	7.138425	Non-Smal	NCIH2009	Lung
ACH-0008	3.836934	6.690696	Non-Smal	NCIH1793	Lung
ACH-0006	3.842979	7.987605	Lung Neu	SCLC21H	Lung
ACH-0000	3.0268	6.762615	Non-Smal	PC14	Lung
ACH-0012	4.632268	5.63285	Embryona	SUMB002	CNS/Brain
ACH-0002	3.276497	5.994353	Mature B-	SUDHL10	Lymphoid
ACH-0005	3.543496	5.458776	Head and	SNU1076	Head and Neck
ACH-0005	3.435629	6.277427	Renal Cell	A498	Kidney
ACH-0004	2.976364	7.246123	Hepatocel	SNU387	Liver
ACH-0007	3.693766	6.516488	Lung Neu	NCIH727	Lung
ACH-0000	3.743084	5.841973	B-Lympho	EHEB	Lymphoid
ACH-0011	4.089159	7.88923	Rhabdoid	KPMRTRY	Kidney
ACH-0014	4.05398	6.301222	Cutaneous	A388	Skin

ACH-0005	4.380591	6.541329	Lung Neu	NCIH1836	Lung
ACH-0005	1.790772	6.471838	Non-Smal	NCIH1299	Lung
ACH-0020	4.040892	6.359135	Nerve She	HSPSS	Peripheral Nervous System
ACH-0006	3.609991	7.160376	Diffuse Gli	LN464	CNS/Brain
ACH-0026	3.646163	5.761551	Colorectal	JVE127	Bowel
ACH-0008	2.867896	5.3875	Lung Neu	DMS454	Lung
ACH-0027	4.769772	6.387156	Rhabdomy	NCCPRMS	Soft Tissue
ACH-0024	5.410748	6.040454	Non-Smal	NCIH920	Lung
ACH-0009	3.626439	7.019257	Non-Smal	DV90	Lung
ACH-0024	4.62527	6.68636	Non-Canc	RPE1SS11	Eye
ACH-0002	2.819668	6.347488	Renal Cell	SLR24	Kidney
ACH-0010	3.635754	6.005625	Ewing Sar	CHLA99	Bone
ACH-0005	3.277985	6.677085	Renal Cell	SNU1272	Kidney
ACH-0010	4.460087	6.851999	Embryona	DL	CNS/Brain
ACH-0009	4.309613	7.305515	Colorectal	SNUC4	Bowel
ACH-0001	4.207112	6.815447	Rhabdomy	RD	Soft Tissue
ACH-0014	4.700994	6.649759	Cervical Sc	SW756	Cervix
ACH-0007	3.032101	6.802322	Ovarian Ep	MCAS	Ovary/Fallopian Tube
ACH-0001	6.313064	6.554435	Non-Smal	HCC2429	Lung
ACH-0000	3.851999	6.871597	Diffuse Gli	HS683	CNS/Brain
ACH-0006	3.414136	6.72615	Melanoma	SKMEL31	Skin
ACH-0016	4.896272	7.116448	Neuroblas	TGW	Peripheral Nervous System
ACH-0016	2.124328	4.024142	Head and	UPCISCC1	Head and Neck
ACH-0020	4.372256	5.508112	Melanoma	A375SKIN	Skin
ACH-0020	4.208673	5.798569	Ocular Me	MP46	Eye
ACH-0009	4.755956	6.539624	Colorectal	SNUC2A	Bowel
ACH-0004	4.485427	7.015582	Diffuse Gli	YH13	CNS/Brain
ACH-0013	3.969933	5.89603	Pancreatic	JOPACA1	Pancreas
ACH-0015	2.613532	5.351911	Acute Mye	MOLM14	Myeloid
ACH-0000	3.661065	6.250014	Invasive Br	ZR751	Breast
ACH-0002	2.983678	5.625855	Ovarian Ep	COV318	Ovary/Fallopian Tube
ACH-0009	2.970854	5.233428	Prostate A	LNCAPCL(	Prostate
ACH-0009	3.715893	6.71122	Endometri	HEC151	Uterus
ACH-0013	5.357552	7.118422	Neuroblas	NMB	Peripheral Nervous System
ACH-0003	3.121015	7.381975	Lung Neu	CORL24	Lung
ACH-0005	2.765535	6.491372	Melanoma	UACC257	Skin
ACH-0020	4.929318	6.886428	Non-Smal	LCAM1	Lung
ACH-0015	4.092546	6.862699	Rhabdoid	JMURTK2	Kidney
ACH-0006	3.1127	7.476382	Lung Neu	CORL47	Lung
ACH-0017	4.617063	7.272303	Liposarco	94T778	Soft Tissue
ACH-0015	1.250962	5.358256	Head and	H376	Head and Neck
ACH-0005	3.66562	5.951634	Pancreatic	TCCPAN2	Pancreas
ACH-0001	4.201634	6.456478	Mature B-	L363	Lymphoid
ACH-0004	3.751678	5.264536	Esophage	TE5	Esophagus/Stomach
ACH-0014	4.879706	5.856986	Esophago	ESO26	Esophagus/Stomach
ACH-0007	3.767655	6.765004	Melanoma	A2058	Skin
ACH-0016	2.629939	6.35931	Diffuse Gli	ONDA7	CNS/Brain
ACH-0004	3.447579	6.60733	Esophago	SNU216	Esophagus/Stomach
ACH-0015	4.768714	6.026136	Pleural Me	MERO83	Pleura
ACH-0000	3.57289	6.384395	Acute Mye	HEL9217	Myeloid
ACH-0016	1.505891	4.106851	Renal Cell	UMRC3	Kidney
ACH-0011	4.198494	6.546894	Ovarian Ep	OVCAR5	Ovary/Fallopian Tube
ACH-0007	3.137504	7.036613	Ovarian Ep	RMUGS	Ovary/Fallopian Tube
ACH-0005	3.49057	6.375735	Ovarian Ep	HEYA8	Ovary/Fallopian Tube
ACH-0001	4.503349	6.493615	Diffuse Gli	KG1C	CNS/Brain
ACH-0016	5.352617	6.163297	Hodgkin L	UHO1	Lymphoid
ACH-0009	3.722466	6.544424	Pancreatic	SNU324	Pancreas
ACH-0000	3.276497	5.797791	Mature B-	REC1	Lymphoid
ACH-0000	3.019702	5.982309	Non-Smal	NCIH1581	Lung

ACH-0011	2.367371	6.090642	Mature T a	L82	Lymphoid
ACH-0004	3.001802	6.595295	Myelodysp	F36P	Myeloid
ACH-0001	4.687621	5.771886	Mature T a	SUPT11	Lymphoid
ACH-0002	3.757023	7.214902	Invasive Br	BT549	Breast
ACH-0018	4.106013	7.398231	Breast Neo	COLO824	Breast
ACH-0007	4.599318	6.641835	B-Lympho	SEM	Lymphoid
ACH-0016	1.510962	5.0387	Ovarian Ep	PEO4	Ovary/Fallopian Tube
ACH-0003	3.770829	5.08534	Mature B-	JEKO1	Lymphoid
ACH-0000	5.534186	7.497213	Non-Canc	TE125T	Fibroblast
ACH-0010	4.878725	6.17971	Embryona	CHLA266	CNS/Brain
ACH-0010	5.037382	5.233428	Mature B-	EB2	Lymphoid
ACH-0010	4.153805	6.450386	Ovarian Ep	COV504	Ovary/Fallopian Tube
ACH-0004	3.41684	4.562548	Mature B-	MOLP2	Lymphoid
ACH-0031	4.495056	6.898813	Non-Canc	ABMT0789	Liver
ACH-0007	4.38819	7.385949	Non-Smal	RERFLCAD	Lung
ACH-0005	4.241077	7.23678	Mature B-	ST486	Lymphoid
ACH-0013	4.453518	6.305059	Invasive Br	SUM159PT	Breast
ACH-0010	3.870858	5.729825	Non-Smal	NCIH292	Lung
ACH-0001	4.275007	7.052351	T-Lympho	ALLSIL	Lymphoid
ACH-0005	4.324091	6.990955	Acute Mye	AML193	Myeloid
ACH-0001	3.949535	7.368943	Non-Canc	TE159T	Fibroblast
ACH-0013	5.260026	6.801805	Anaplastic	MB1	Thyroid
ACH-0018	4.100978	6.727648	Intraducta	ICC4	Biliary Tract
ACH-0013	3.819668	4.795455	Head and	H103	Head and Neck
ACH-0008	3.505891	7.435462	Ovarian Ep	TOV21G	Ovary/Fallopian Tube
ACH-0009	2.427606	6.311794	Non-Smal	NCIH23	Lung
ACH-0006	4.361768	7.145168	Non-Smal	NCIH1355	Lung
ACH-0002	3.168321	7.132577	Hepatocel	JHH6	Liver
ACH-0008	3.937344	5.818646	Non-Smal	HCC515	Lung
ACH-0020	4.393004	6.293702	Cervical Sc	SKGIIIA	Cervix
ACH-0016	4.544114	4.617651	Mature B-	RCK8	Lymphoid
ACH-0012	4.217231	5.203201	Head and	UPCISCC1	Head and Neck
ACH-0001	3.619413	5.481557	Head and	SCC9	Head and Neck
ACH-0001	3.901108	5.615593	Prostate A	VCAP	Prostate
ACH-0001	4.482203	6.838448	B-Lympho	NALM19	Lymphoid
ACH-0001	4.06264	5.97728	Non-Smal	NCIH2405	Lung
ACH-0017	3.732269	6.19101	Embryona	PFSK1	CNS/Brain
ACH-0009	3.803227	6.604516	Invasive Br	MDAMB36	Breast
ACH-0009	4.185074	6.493775	Endometri	HEC251	Uterus
ACH-0015	3.564378	8.023144	Gestationa	JAR	Uterus
ACH-0017	5.043957	6.054414	Rhabdomy	RC2	Soft Tissue
ACH-0003	3.229588	5.915521	Pancreatic	PSN1	Pancreas
ACH-0005	3.998196	7.06953	Leiomyosa	RKN	Soft Tissue
ACH-0007	4.066089	7.093919	Non-Smal	NCIH2023	Lung
ACH-0000	4.596935	6.556736	B-Lympho	SUPB15	Lymphoid
ACH-0009	4.173927	6.855741	Myeloprol	CMLT1	Myeloid
ACH-0002	4.232661	6.843104	Acute Mye	NB4	Myeloid
ACH-0004	4.07382	7.428611	Hepatocel	SNU475	Liver
ACH-0002	4.151372	7.374779	Diffuse Gli	NMCG1	CNS/Brain
ACH-0000	3.641546	7.32562	Renal Cell	ACHN	Kidney
ACH-0009	3.122673	8.133399	Melanoma	COLO792	Skin
ACH-0008	4.289834	7.304146	Lung Neur	NCIH1436	Lung
ACH-0001	3.680324	5.501121	Pancreatic	SU8686	Pancreas
ACH-0004	4.108524	6.96786	Mature B-	BL70	Lymphoid
ACH-0015	4.523562	6.122259	Non-Semi	NCCIT	Testis
ACH-0000	3.358959	5.621759	Diffuse Gli	U343	CNS/Brain
ACH-0009	3.903038	7.781622	Urethral C	639V	Bladder/Urinary Tract
ACH-0011	4.521679	7.320394	Rhabdoid	MON	Kidney
ACH-0007	3.300124	5.872583	Melanoma	LOXIMVI	Skin

ACH-0002	5.314334	6.488804	Mature B-	TOLEDO	Lymphoid
ACH-0026	4.047015	5.814038	Colorectal	KP363T	Bowel
ACH-0018	3.870858	5.768714	Intraducta	SSP25	Biliary Tract
ACH-0019	4.635754	5.334497	Intraducta	ECC2	Biliary Tract
ACH-0000	3.968091	6.889474	Ewing Sarc	A673	Bone

Supplementary Table 21

GSE19274

Genes	GSM4781!	GSM4781!	GSM4781!	GSM4781!	GSM4781!	GSM4781!	GSM4781!	GSM4781!
METTL14	11.63421	8.948652	10.05927	10.46081	10.70091	9.303444	9.667921	9.439313
YWHAH	554.8763	491.5355	536.288	471.582	263.0551	1675.624	416.3153	854.695

GSE49710

Genes	GSM1205:	GSM1205:	GSM1205:	GSM1205:	GSM1205:	GSM1205:	GSM1205:	GSM1205:
YWHAH	14.30853	14.50889	13.47687	14.96758	14.83379	15.1751	14.31722	14.75037
YTHDF1	11.54751	11.47375	11.33203	10.75247	11.06305	11.11374	11.2027	11.21758

GSE120559

Genes	GSM3402!	GSM3402!	GSM3402!	GSM3402!	GSM3402!	GSM3402!	GSM3402!	GSM3402!
METTL14	12.0068	11.59989	11.62275	11.58594	11.42061	11.70437	11.37421	11.48734
YWHAH	13.37435	14.22491	13.37948	14.05681	13.03749	13.31562	13.8467	14.74702

GSE73517

Genes	GSM1897(	GSM1897(	GSM1897(	GSM1897(	GSM1897(	GSM1897(	GSM1897(	GSM1897(
METTL14	11.55735	12.15436	11.21213	11.71882	11.47526	10.42091	11.06083	11.60221
YWHAH	12.35854	13.54811	14.1468	13.53798	13.93189	13.83222	14.51181	14.1797

GSE120572

Genes	GSM3402!	GSM3402!	GSM3402!	GSM3402!	GSM3402!	GSM3402!	GSM3402!	GSM3402!
METTL14	12.00805	11.6013	11.6244	11.58714	11.4226	11.70583	11.37622	11.48884
YWHAH	13.37429	14.2255	13.37948	14.05593	13.03739	13.31542	13.84627	14.75022

GSE45547

Genes	GSM1108-	GSM1108-	GSM1108-	GSM1108-	GSM1108-	GSM1108-	GSM1108-	GSM1108-
YWHAH	14.46432	14.45192	13.38725	13.6972	13.06268	14.19212	14.84131	14.39921
YTHDF1	10.93738	11.09895	11.06564	10.6244	11.4558	11.46282	11.20516	11.38088

GSM4781	GSM4781	GSM4781	GSM4781	GSM4781	GSM4781	GSM4781	GSM4781	GSM4781
10.99202	9.242698	10.62637	10.24313	9.846884	10.65711	10.39085	10.45897	9.73706
458.408	483.9344	744.2792	713.8342	744.2792	824.0131	546.9007	941.9667	977.5551

GSM1205	GSM1205	GSM1205	GSM1205	GSM1205	GSM1205	GSM1205	GSM1205	GSM1205
14.63144	13.90918	14.34382	14.73118	14.89565	14.26254	14.38383	14.45969	13.50969
11.76382	11.36973	11.26167	11.2037	11.20181	10.97254	11.29488	10.98178	11.30347

GSM3402	GSM3402	GSM3402	GSM3402	GSM3402	GSM3402	GSM3402	GSM3402	GSM3402
11.7971	11.67537	11.86871	11.04353	11.83779	11.39564	10.78855	11.35447	11.52665
14.55052	13.69123	13.60058	13.59328	13.204	13.76148	13.91695	13.51486	13.9331

GSM1897	GSM1897	GSM1897	GSM1897	GSM1897	GSM1897	GSM1897	GSM1897	GSM1897
11.72355	11.23757	11.48584	11.62512	11.58465	11.21921	11.67152	11.30406	11.69936
13.18245	12.92759	14.09558	13.44308	13.88035	14.52563	13.32442	14.28082	13.35154

GSM3402	GSM3402	GSM3402	GSM3402	GSM3402	GSM3402	GSM3402	GSM3402	GSM3402
11.79902	11.67673	11.86955	11.04522	11.83885	11.39749	10.79092	11.35655	11.52791
14.55283	13.69138	13.60097	13.59359	13.20323	13.76198	13.91711	13.51427	13.93295

GSM1108	GSM1108	GSM1108	GSM1108	GSM1108	GSM1108	GSM1108	GSM1108	GSM1108
15.2584	14.72948	13.9137	14.00794	13.82665	14.42494	14.25796	14.22207	14.25555
11.22098	10.5993	11.15401	11.29084	11.10622	11.34677	11.67558	11.45076	11.36912

GSM4781(	GSM4781(	GSM4781'	GSM4781'	GSM4781'	GSM4781'	GSM4781'	GSM4781'	GSM4781'
9.385812	9.350512	10.87321	12.10219	11.15782	10.57392	9.184424	12.08957	9.100547
836.5618	381.0517	441.0938	515.6624	1310.603	1017.624	1326.76	566.1576	810.6402

GSM1205:	GSM1205:	GSM1205:	GSM1205:	GSM1205:	GSM1205:	GSM1205:	GSM1205:	GSM1205:
13.64541	14.59278	14.43895	14.30648	13.98949	13.91067	13.8394	14.63986	14.75365
11.42637	10.93044	11.14105	11.29848	11.31694	11.73078	11.63697	11.09965	11.1049

GSM3403(	GSM3403(	GSM3403(	GSM3403(	GSM3403(	GSM3403(	GSM3403(	GSM3403(	GSM3403(
11.07801	11.91408	11.95005	11.78973	12.04266	11.61578	11.64598	11.50325	10.2893
13.32095	13.20214	14.48017	14.69411	13.55516	13.50656	13.60181	13.3678	13.96356

GSM1897(	GSM1897(	GSM1897(	GSM1897(	GSM1897(	GSM1897(	GSM1897(	GSM1897(	GSM1897(
11.26117	11.3297	11.10925	11.33617	11.7061	12.01427	10.3475	11.90812	12.80509
14.2986	14.0583	13.27894	13.10723	13.11296	12.88365	13.42269	13.49117	13.15686

GSM3403(	GSM3403(	GSM3403(	GSM3403(	GSM3403(	GSM3403(	GSM3403(	GSM3403(	GSM3403(
11.07986	11.91503	11.9511	11.79166	12.04401	11.61727	11.64745	11.50473	10.29115
13.32082	13.20132	14.48266	14.69781	13.55498	13.50603	13.60221	13.36782	13.96288

GSM1108!	GSM1108!	GSM1108!	GSM1108!	GSM1108!	GSM1108!	GSM1108!	GSM1108!	GSM1108!
14.21379	14.21979	13.89187	13.65815	14.97169	12.87565	14.09351	13.85383	14.78902
11.57875	11.60349	11.43449	11.27253	11.15897	11.18923	11.18333	10.74282	10.77711

GSM4781	GSM4781	GSM4781	GSM4781	GSM4781	GSM4781	GSM4781	GSM4781	GSM4781
10.70251	10.73861	10.34352	9.312765	9.321731	9.377868	11.03954	9.725748	10.92109
810.6402	888.595	1249.04	977.5551	399.3953	762.357	1349.281	458.408	936.4029

GSM1205	GSM1205	GSM1205	GSM1205	GSM1205	GSM1205	GSM1205	GSM1205	GSM1205
13.99442	13.57215	14.54147	14.16061	13.62218	14.79133	14.29069	14.2145	14.74892
11.33888	11.5161	10.93971	11.48894	11.4973	11.20663	11.4129	11.44633	10.62618

GSM3403	GSM3403	GSM3403	GSM3403	GSM3403	GSM3403	GSM3403	GSM3403	GSM3403
11.82615	11.57707	11.16703	11.78973	11.46234	11.82268	12.39471	11.70437	10.92958
14.05681	12.98717	14.36572	12.87509	15.12271	13.18726	13.96947	13.43604	13.37639

GSM1897	GSM1897	GSM1897	GSM1897	GSM1897	GSM1897	GSM1897	GSM1897	GSM1897
11.48982	12.37114	10.91852	11.76943	11.61487	11.74257	11.14208	11.47243	11.42833
13.69036	13.76395	14.92515	13.29028	13.34867	14.01067	14.89579	13.82663	14.2356

GSM3403	GSM3403	GSM3403	GSM3403	GSM3403	GSM3403	GSM3403	GSM3403	GSM3403
11.82746	11.57823	11.16877	11.79166	11.46389	11.82419	12.39459	11.70583	10.93167
14.05593	12.98692	14.36617	12.87516	15.12739	13.18682	13.96899	13.43578	13.37633

GSM1108	GSM1108	GSM1108	GSM1108	GSM1108	GSM1108	GSM1108	GSM1108	GSM1108
14.10148	14.13056	14.69314	14.37204	14.18795	13.77918	13.96875	14.17949	14.46197
10.9395	11.32777	10.50671	11.03372	11.25167	11.1523	10.76268	11.16246	10.97016

GSM47818	GSM47818	GSM47818	GSM47818	GSM47819	GSM47819	GSM47819	GSM47819	GSM47819
10.52192	9.921548	9.442753	9.84065	9.159186	10.51073	9.225894	9.94564	8.808973
1280.632	754.9122	579.969	1502.568	836.5618	638.1857	1530.772	503.7968	1194.605

GSM12053	GSM12053	GSM12053	GSM12053	GSM12053	GSM12053	GSM12053	GSM12053	GSM12053
14.17876	13.94912	14.43569	14.26381	14.80036	13.86907	14.05519	14.53396	14.3976
11.05696	10.84119	11.18869	11.38845	10.74564	11.26919	10.91954	11.11352	11.29758

GSM34030	GSM34030	GSM34030	GSM34030	GSM34030	GSM34030	GSM34030	GSM34030	GSM34030
11.0784	12.13149	11.512	10.42316	11.53602	11.64196	11.81662	11.18341	11.64795
13.04715	12.88036	13.32302	13.94706	14.44966	14.18384	12.98454	14.27767	13.54909

GSM18970	GSM18970	GSM18970	GSM18970	GSM18970	GSM18970	GSM18970	GSM18970	GSM18970
11.48212	11.04263	11.16375	11.09322	11.21213	11.36584	12.10507	10.99955	11.93179
14.16777	14.25551	14.5633	14.53553	14.269	14.00227	13.63019	14.82261	13.84649

GSM34030	GSM34030	GSM34030	GSM34030	GSM34030	GSM34030	GSM34030	GSM34030	GSM34030
11.08028	12.13191	11.51329	10.42522	11.5371	11.64343	11.81828	11.18492	11.64944
13.04706	12.88037	13.32282	13.94697	14.45116	14.18347	12.98428	14.27892	13.5488

GSM11085	GSM11086	GSM11086	GSM11086	GSM11086	GSM11086	GSM11086	GSM11086	GSM11086
13.7659	14.08384	14.8322	14.64361	14.2072	13.97573	13.87724	13.66975	14.53406
11.50549	11.37396	11.3558	10.88088	11.4079	11.58031	11.1268	11.36057	11.06564

GSM4781	GSM4781	GSM4781	GSM4781	GSM4781	GSM4782	GSM4782	GSM4782	GSM4782
11.01258	10.65817	10.70668	9.897212	8.667008	10.39198	7.422933	9.664863	9.365
744.2792	433.6404	416.3153	309.0781	471.582	446.2161	520.7099	351.0495	460.9723

GSM1205	GSM1205	GSM1205	GSM1205	GSM1205	GSM1205	GSM1205	GSM1205	GSM1205
14.60499	14.8928	14.35882	14.84107	13.43511	14.24796	14.60197	14.16331	14.30195
11.16289	11.59539	11.61522	10.89958	11.36105	11.28409	11.53635	11.55312	11.54887

GSM3403	GSM3403	GSM3403	GSM3403	GSM3403	GSM3403	GSM3403	GSM3403	GSM3403
12.27443	12.12068	12.32957	11.41647	11.65294	10.91549	12.42867	11.58353	10.35409
13.13562	13.79375	13.30743	12.20523	12.59067	13.33536	14.24115	13.19711	12.92281

GSM1897	GSM1897	GSM1897	GSM1897	GSM1897	GSM1897	GSM1897	GSM1897	GSM1897
10.95046	11.54055	11.32689	11.40327	11.3807	10.85257	11.70335	9.899286	11.12094
14.78497	14.37572	13.97035	14.09808	14.07881	14.33533	13.45907	13.75397	13.24984

GSM3403	GSM3403	GSM3403	GSM3403	GSM3403	GSM3403	GSM3403	GSM3403	GSM3403
12.27417	12.12107	12.32923	11.41835	11.65449	10.91757	12.42869	11.58477	10.35616
13.13517	13.79401	13.30706	12.20524	12.59053	13.33549	14.24153	13.19655	12.92245

GSM1108	GSM1108	GSM1108	GSM1108	GSM1108	GSM1108	GSM1108	GSM1108	GSM1108
14.27855	14.54409	14.08003	14.22609	14.54198	13.27643	14.20041	14.28928	14.66275
11.54714	11.40935	11.43197	11.50549	11.26342	11.57204	10.9693	11.09266	11.28733

GSM4782(	GSM4782(	GSM4782(	GSM4782(	GSM4782(	GSM4782(	GSM4782:	GSM4782:	GSM4782:
9.786778	10.84548	9.623515	10.93759	10.83314	10.61264	8.807317	9.262375	10.9474
501.1454	375.3699	503.7968	677.6239	443.7552	633.6994	446.2161	515.6624	612.0553

GSM1205:	GSM1205:	GSM1205:	GSM1205:	GSM1205:	GSM1205:	GSM1205:	GSM1205:	GSM1205:
14.99844	14.61503	14.27633	14.21262	14.10372	15.29065	15.48077	14.4161	14.50339
11.11732	11.33857	11.06853	11.24847	11.47805	11.37771	11.52442	11.38605	11.06923

GSM3403(	GSM3403(	GSM3403(	GSM3403(	GSM3403(	GSM3403(	GSM3403(	GSM3403(	GSM3403(
11.26306	11.97342	11.4936	11.26837	11.61136	11.73643	11.16662	11.41185	11.65615
13.25428	13.36229	12.97708	13.57652	13.59197	13.18269	14.09194	13.18369	13.54909

GSM1897:	GSM1897:	GSM1897:	GSM1897:	GSM1897:	GSM1897:	GSM1897:	GSM1897:	GSM1897:
10.89652	11.8967	11.14757	10.77383	10.83615	11.21879	11.27369	10.65507	11.58029
13.11186	12.66376	14.54347	14.5647	14.07511	13.62133	13.18907	11.97427	13.84227

GSM3403(	GSM3403(	GSM3403(	GSM3403(	GSM3403(	GSM3403(	GSM3403(	GSM3403(	GSM3403(
11.26533	11.97422	11.49513	11.27069	11.61289	11.73851	11.16835	11.41368	11.65764
13.25372	13.36252	12.97679	13.57658	13.59232	13.18199	14.09135	13.18301	13.5488

GSM1108(	GSM1108(	GSM1108(	GSM1108(	GSM1108(	GSM1108(	GSM1108(	GSM1108(	GSM1108
14.9294	14.84506	14.56348	14.43008	13.80675	13.48833	14.19858	13.95089	14.24508
11.04867	11.5414	10.93302	11.31157	11.58281	11.33828	11.27758	11.5919	11.02274

GSM4782	GSM4782	GSM4782	GSM4782	GSM4782	GSM4782	GSM4782	GSM4782	GSM4782
11.34872	9.718369	9.642691	10.78002	10.24883	10.5993	11.81703	8.586998	10.95383
295.1962	373.7306	373.7306	501.1454	545.1992	532.3986	356.246	726.6864	471.582

GSM1205	GSM1205	GSM1205	GSM1205	GSM1205	GSM1205	GSM1205	GSM1205	GSM1205
14.49489	14.57636	13.6687	13.48721	14.29482	13.39003	13.37631	13.99856	14.09398
11.26655	11.3098	11.49665	11.19874	11.56795	11.54478	11.70054	11.28239	11.41826

GSM3403	GSM3403	GSM3403	GSM3403	GSM3403	GSM3403	GSM3403	GSM3403	GSM3403
11.69401	11.71752	11.36255	11.63897	11.41884	11.27432	11.44603	11.61136	11.44555
14.45157	14.2076	14.19119	13.65668	14.02731	14.09373	13.50656	13.05925	13.75446

GSM1897	GSM1897	GSM1897	GSM1897	GSM1897	GSM1897	GSM1897	GSM1897	GSM1897
11.618	11.63895	11.6104	11.3341	11.61097	12.04518	11.64148	11.1653	11.76142
13.09827	13.84754	13.18397	13.39341	13.37871	13.74817	13.29156	13.45317	13.46724

GSM3403	GSM3403	GSM3403	GSM3403	GSM3403	GSM3403	GSM3403	GSM3403	GSM3403
11.69508	11.71942	11.3647	11.64052	11.42075	11.27657	11.44781	11.61289	11.44736
14.45323	14.2082	14.19081	13.65719	14.02611	14.09323	13.50603	13.05892	13.75478

GSM1108	GSM1108	GSM1108	GSM1108	GSM1108	GSM1108	GSM1108	GSM1108	GSM1108
14.68745	14.5838	13.91876	14.52275	14.33869	14.69314	13.31141	13.9137	13.39212
11.05599	11.53328	11.13489	10.75215	11.11526	10.89377	11.35438	11.13889	11.10444

GSM4782	GSM4782	GSM4782	GSM4782	GSM4782	GSM4782	GSM4782	GSM4782	GSM4782
10.54435	11.52406	11.06979	9.914944	12.2143	9.459172	8.854977	9.722487	10.55387
666.7751	527.9284	485.7714	646.7069	362.6378	563.3881	422.2206	494.3153	509.2973

GSM1205	GSM1205	GSM1205	GSM1205	GSM1205	GSM1205	GSM1205	GSM1205	GSM1205
14.26014	14.23433	14.29886	14.20805	13.29337	14.47223	13.91376	14.09697	14.89565
11.59317	11.56292	11.31886	11.38557	11.51541	11.48849	11.23963	11.50197	11.29167

GSM3403	GSM3403	GSM3403	GSM3403	GSM3403	GSM3403	GSM3403	GSM3403	GSM3403
12.08973	11.75156	12.00984	11.22424	11.2692	11.94884	11.99153	11.81883	11.38342
13.35185	13.89733	13.30225	13.9951	13.25527	12.55008	13.32095	13.53112	14.14133

GSM1897	GSM1897	GSM1897	GSM1897	GSM1897	GSM1897	GSM1897	GSM1897	GSM1897
11.26416	10.82801	10.95658	11.71278	11.28602	10.91842	10.75997	11.4378	10.49727
12.33969	14.27273	13.44081	13.13087	14.70305	14.06828	13.3775	13.60071	13.70118

GSM3403	GSM3403	GSM3403	GSM3403	GSM3403	GSM3403	GSM3403	GSM3403	GSM3403
12.09039	11.75376	12.01101	11.22617	11.27156	11.94997	11.99249	11.82046	11.38532
13.35193	13.89726	13.30182	13.99419	13.25473	12.5501	13.32082	13.53072	14.14107

GSM1108	GSM1108	GSM1108	GSM1108	GSM1108	GSM1108	GSM1108	GSM1108	GSM1108
14.92627	14.78594	15.13144	14.23485	13.83279	14.27165	14.66817	14.84848	14.17949
10.56251	10.85925	10.93912	11.38768	11.17429	11.08962	10.96126	11.00253	10.76674

GSM4782	GSM4782	GSM4782	GSM4782	GSM4782	GSM4782	GSM4782	GSM4782	GSM4782
11.37645	9.995326	10.2589	10.78954	12.35304	9.222436	8.116305	10.81344	10.80222
354.6367	399.3953	460.9723	433.6404	488.6265	623.3023	713.8342	441.0938	450.6238

GSM1205	GSM1205	GSM1205	GSM1205	GSM1205	GSM1205	GSM1205	GSM1205	GSM1205
14.52226	14.49043	14.09244	14.19318	14.31822	14.37823	14.33023	13.56525	13.7927
11.49712	11.47805	11.54212	11.28674	12.11381	11.38787	11.79699	11.64566	10.73513

GSM3403	GSM3403	GSM3403	GSM3403	GSM3403	GSM3403	GSM3403	GSM3403	GSM3403
11.41272	11.61529	11.95745	11.54716	11.89969	11.49064	12.32219	12.2219	12.03817
14.55052	13.25428	13.0555	14.79265	13.47608	12.71489	13.71846	14.10952	12.99513

GSM1897	GSM1897	GSM1897	GSM1897	GSM1897	GSM1897	GSM1897	GSM1897	GSM1897
11.07945	11.79624	11.38555	11.3094	11.65042	11.12573	11.37413	11.30691	12.04453
13.29156	13.41713	13.37215	12.75304	13.63806	13.38239	12.84379	12.7871	12.90828

GSM3403	GSM3403	GSM3403	GSM3403	GSM3403	GSM3403	GSM3403	GSM3403	GSM3403
11.41459	11.61679	11.95839	11.54819	11.90077	11.49224	12.32206	12.22176	12.03931
14.55283	13.25372	13.05525	14.79576	13.47584	12.71502	13.71949	14.10932	12.99499

GSM1108	GSM1108	GSM1108	GSM1108	GSM1108	GSM1108	GSM1108	GSM1108	GSM1108
14.32042	14.38995	13.55384	13.40598	14.73236	14.47767	13.53491	14.36342	14.23082
11.07607	10.76887	11.25484	11.11568	10.97684	10.72636	11.34915	10.9799	11.13393

GSM4782	GSM4782	GSM4782	GSM4782	GSM4782	GSM4782	GSM4782	GSM4782	GSM4782
11.092	10.46404	10.86499	10.26088	10.09118	10.18868	10.62416	10.251	11.04598
437.348	427.1558	424.7199	356.246	867.2391	1017.624	1468.464	406.6067	460.9723

GSM1205	GSM1205	GSM1205	GSM1205	GSM1205	GSM1205	GSM1205	GSM1205	GSM1205
14.50224	13.74101	14.30438	14.13839	14.52566	14.07214	13.40843	14.17112	13.97602
11.1742	11.4379	11.68506	11.28573	11.8662	11.34429	11.5077	11.34662	11.55827

GSM3403	GSM3403	GSM3403	GSM3403	GSM3403	GSM3403	GSM3403	GSM3403	GSM3403
11.84215	11.30318	11.28933	11.75376	10.89422	11.51097	11.22471	11.68822	11.72496
12.75142	12.36879	13.03573	13.16863	14.43132	14.22111	13.69356	14.03694	12.22321

GSM1897	GSM1897	GSM1897	GSM1897	GSM1897	GSM1897	GSM1897	GSM1897	GSM1897
11.4116	11.53402	11.33548	11.03518	11.82602	11.34656	10.89141	11.59857	11.65537
12.36934	12.65613	12.99159	13.40722	13.11186	13.44215	13.8606	13.86421	13.51604

GSM3403	GSM3403	GSM3403	GSM3403	GSM3403	GSM3403	GSM3403	GSM3403	GSM3403
11.84309	11.30535	11.29161	11.75591	10.89646	11.51228	11.2266	11.6894	11.72687
12.75191	12.36872	13.03565	13.16801	14.43287	14.22179	13.69384	14.03589	12.22308

GSM1108	GSM1108	GSM1108	GSM1108	GSM1108	GSM1108	GSM1108	GSM1108	GSM1108
13.6541	13.63594	13.92945	15.15466	14.02077	13.92945	14.53674	14.58113	14.38756
11.25716	11.42853	10.92455	11.26713	11.26342	11.40348	10.96524	11.18782	11.24306

GSM47824	GSM47825	GSM47825	GSM47825	GSM47825	GSM47825	GSM47825	GSM47825	GSM47825
12.60314	10.56843	11.50605	10.84017	10.8293	12.70915	10.68589	12.00605	11.93508
378.2257	918.8298	906.1242	1113.071	351.0495	659.6473	220.8886	387.2163	316.0811

GSM12055	GSM12055	GSM12055	GSM12056	GSM12056	GSM12056	GSM12056	GSM12056	GSM12056
14.29972	14.32914	14.35978	14.17305	14.27094	13.76311	14.10642	14.92128	14.47023
11.63306	11.46459	11.2037	11.5235	11.32293	11.58224	11.53523	12.10531	11.57863

GSM34031	GSM34031	GSM34031	GSM34031	GSM34031	GSM34031	GSM34031	GSM34031	GSM34031
11.2388	12.08416	11.76363	11.8432	11.65197	11.77519	11.87674	11.62275	11.55053
14.61715	13.33755	13.73675	13.40565	13.48654	12.15129	13.77399	13.58862	13.00892

GSM18971	GSM18971	GSM18971	GSM18971	GSM18971	GSM18971	GSM1897157
10.823	11.87758	11.65042	10.51597	10.77165	11.47723	11.27047
12.78322	13.13574	13.5602	13.57039	13.89261	13.45994	13.1466

GSM34031	GSM34031	GSM34031	GSM34031	GSM34031	GSM34031	GSM34031	GSM34031	GSM34031
11.24083	12.08496	11.76583	11.84416	11.65345	11.77745	11.87772	11.6244	11.55159
14.61973	13.33753	13.73728	13.40568	13.48628	12.15171	13.77441	13.58876	13.00881

GSM11088	GSM11088	GSM11088	GSM11088	GSM11088	GSM11088	GSM11088	GSM11088	GSM11088
14.41111	14.54409	13.61323	13.85725	14.13238	14.43478	13.30351	14.42241	14.5101
11.19799	10.69869	11.51077	11.35778	11.24442	10.93785	11.40452	11.16519	11.23359

GSM4782	GSM4782	GSM4782	GSM4782	GSM4782	GSM4782	GSM4782	GSM4782	GSM4782
10.70017	12.80712	9.426411	14.21356	8.839662	12.9687	10.93386	12.51638	11.75805
293.0368	319.7049	392.553	433.6404	383.5746	375.3699	349.5643	362.6378	292.1661

GSM1205	GSM1205	GSM1205	GSM1205	GSM1205	GSM1205	GSM1205	GSM1205	GSM1205
13.78604	14.46385	14.64245	13.77492	13.94524	14.43895	13.57106	14.8928	14.69244
11.8256	11.38021	11.34624	11.14569	11.45157	11.61445	11.3458	11.43258	11.02634

GSM3403	GSM3403	GSM3403	GSM3403	GSM3403	GSM3403	GSM3403	GSM3403	GSM3403
12.03297	10.74035	11.83003	10.9374	12.169	11.8048	11.58208	11.25885	11.39564
12.90576	12.93805	13.37206	14.16647	13.58622	13.48417	13.46059	14.89559	13.37744

GSM3403	GSM3403	GSM3403	GSM3403	GSM3403	GSM3403	GSM3403	GSM3403	GSM3403
12.03406	10.74305	11.83122	10.93932	12.1693	11.80663	11.58334	11.26095	11.39749
12.90559	12.93775	13.37209	14.16672	13.58628	13.48393	13.4602	14.89896	13.37741

GSM1108	GSM1108	GSM1108	GSM1108	GSM1108	GSM1108	GSM1108	GSM1108	GSM1108
14.05903	14.01503	13.48133	14.02839	14.64112	14.3323	14.22407	14.01889	14.14089
11.13069	11.3458	11.22386	11.37748	10.76803	11.29892	12.03822	11.44622	11.11781

GSM4782	GSM4782	GSM4782	GSM4782	GSM4782	GSM4782	GSM4782	GSM4782	GSM4782
11.73308	9.408767	11.06538	11.76561	9.101913	10.42712	11.71512	10.67854	12.08232
458.408	399.3953	422.2206	532.3986	383.5746	460.9723	381.0517	301.0514	239.5847

GSM1205	GSM1205	GSM1205	GSM1205	GSM1205	GSM1205	GSM1205	GSM1205	GSM1205
14.27827	14.05519	13.97313	13.76106	14.18581	14.42114	15.14548	14.14897	14.54804
11.51994	11.70017	11.23398	11.48059	11.20828	11.398	10.78449	11.61	11.32353

GSM3403	GSM3403	GSM3403	GSM3402	GSM3402	GSM3402	GSM3402	GSM3403	GSM3403
11.35113	12.84418	10.42123	11.61119	11.39958	11.50717	11.18883	11.44824	11.80138
13.57264	13.19993	13.88414	14.1158	13.69477	14.20958	14.4274	14.31502	14.47856

GSM3403	GSM3403	GSM3403	GSM3403	GSM3403	GSM3403	GSM3403	GSM3403	GSM3403
11.35323	12.84414	10.4233	11.64991	11.92568	11.35472	12.05674	11.72091	11.60035
13.57271	13.19928	13.88411	13.27274	13.59232	14.09672	13.58392	14.03757	13.37532

GSM1108	GSM1108	GSM1108	GSM1108	GSM1108	GSM1108	GSM1108	GSM1108	GSM1108
13.1743	13.57896	14.15268	14.44436	15.45039	14.21979	14.30219	14.11361	14.45192
11.39194	11.38233	11.45421	11.40025	11.43554	11.20976	11.26481	11.15981	11.7782

GSM4782	GSM4782	GSM4782	GSM4782	GSM4782	GSM4782	GSM4782	GSM4782	GSM4782
11.01787	12.15631	12.84448	12.77154	11.86041	8.422947	11.51548	11.33442	12.03471
284.6726	546.9007	297.1919	362.6378	383.5746	340.9235	289.0219	303.2604	257.6922

GSM1205	GSM1205	GSM1205	GSM1205	GSM1205	GSM1205	GSM1205	GSM1205	GSM1205
13.40928	13.7963	14.53887	15.23364	14.67428	13.70243	13.93611	14.15418	14.81431
11.16445	11.53516	11.40888	10.96819	11.13717	11.81489	11.06715	11.49081	11.24284

GSM3403	GSM3403	GSM3403	GSM3403	GSM3403	GSM3403	GSM3403	GSM3403	GSM3403
11.61336	11.37342	10.39447	12.01753	10.62986	11.09147	11.60134	11.15804	11.31573
13.7782	13.79861	15.63801	14.43986	15.09123	14.09678	14.22832	15.73175	14.81522

GSM3403	GSM3403	GSM3403	GSM3403	GSM3403	GSM3403	GSM3403	GSM3403	GSM3403
11.72635	11.72091	11.54723	11.63207	11.57437	11.1178	11.48263	12.11435	11.55989
13.86959	13.85073	14.37019	14.44413	14.07217	14.04419	13.82727	13.14655	13.31029

GSM1108	GSM1108	GSM1108	GSM1108	GSM1108	GSM1108	GSM1108	GSM1108	GSM1108
14.07811	13.67131	14.17358	13.2796	14.00036	12.84246	13.23117	14.85907	14.66544
11.42002	11.47017	11.23407	11.42947	11.20877	11.73889	10.77182	12.04605	11.44972

GSM4782(	GSM4782(	GSM4782(	GSM478288
10.2059	11.11546	14.38514	11.82963
274.0473	281.7133	264.9075	351.0495

GSM1205:	GSM1205:	GSM1205:	GSM1205:	GSM1205:	GSM1205:	GSM1205:	GSM1205:	GSM1205:
14.11286	13.90769	14.30531	13.84187	14.84304	13.73267	13.95747	14.27042	14.46499
11.12556	10.98964	11.23002	11.2556	10.94752	11.48126	11.32431	11.58608	11.16052

GSM3403(	GSM3403(	GSM3403(	GSM3403:	GSM3403:	GSM3403:	GSM3403:	GSM3403:	GSM3403:
11.56529	11.99697	11.34881	11.52999	11.05102	11.26823	11.24779	11.52581	11.7731
14.06136	13.90794	14.49512	13.46291	15.18096	14.88545	13.7902	14.07486	15.12073

GSM3403:	GSM3403:	GSM3403:	GSM3403:	GSM3403:	GSM3403:	GSM3403:	GSM3403:	GSM3403:
11.96574	11.98072	11.91781	11.59745	11.42829	11.87772	11.37392	11.59305	11.35123
13.69138	14.15569	13.86226	13.648	13.48393	14.04782	13.59596	13.61799	13.22057

GSM1108(	GSM1108(	GSM1108(	GSM1108(	GSM1108(	GSM1108(	GSM1108(	GSM1109(	GSM1109(
14.89844	13.81342	14.38756	14.75534	13.66243	14.64618	13.98664	13.90848	14.00605
11.30981	11.44431	11.51521	11.45076	11.03895	11.58586	11.53177	11.6868	11.66804

GSM1205	GSM1205	GSM1205	GSM1205	GSM1205	GSM1205	GSM1205	GSM1205	GSM1205
14.29178	14.79164	14.0713	14.1955	14.30297	14.0647	12.40919	14.53674	13.7573
11.12351	11.08533	10.90762	11.46329	11.35493	10.86066	11.18464	11.31052	11.23563

GSM3403	GSM3403	GSM3403	GSM3403	GSM3402	GSM3402	GSM3402	GSM3403	GSM3403
11.69742	11.33755	10.91507	11.34549	10.98996	11.44171	11.92114	11.42085	11.51302
13.39366	13.57515	14.37854	13.93809	14.79441	13.69324	13.27603	13.67623	14.22707

GSM3403	GSM3403	GSM3403	GSM3403	GSM3403	GSM3403	GSM3403	GSM3403	GSM3403
10.4233	11.47931	11.75267	12.07256	11.43441	11.53522	11.41882	12.40813	11.68284
13.69258	13.42443	13.93777	13.30499	13.92815	14.4374	13.69384	15.57053	13.9644

GSM1109	GSM1109	GSM1109	GSM1109	GSM1109	GSM1109	GSM1108	GSM1108	GSM1108
14.2072	14.25796	14.77356	14.52843	13.11777	13.94023	14.80858	13.78877	14.54811
11.28077	10.84439	11.17291	11.04027	10.95831	10.87828	11.39079	11.1973	11.24626

GSM1205	GSM1205	GSM1205	GSM1205	GSM1205	GSM1205	GSM1205	GSM1205	GSM1205
14.2872	14.54088	13.70836	14.23176	14.88167	13.75293	13.36166	14.18163	14.90691
11.21791	11.30137	11.49251	11.22435	11.1062	11.87029	11.25152	11.2255	11.06292

GSM3403	GSM3403	GSM3403	GSM3403	GSM3403	GSM3403	GSM3403	GSM3403	GSM3403
11.86198	10.33227	11.21247	11.56197	11.97257	12.42155	10.6994	11.25693	11.17693
13.50897	14.14084	14.76933	13.66279	12.44699	13.13991	13.92181	15.0787	14.78383

GSM3403	GSM3403	GSM3403	GSM3403	GSM3403	GSM3403	GSM3403	GSM3403	GSM3403
11.49801	11.95839	11.5376	11.42682	11.68736	11.88156	11.93188	11.4784	11.08569
14.3179	13.35526	12.58536	13.32183	13.35945	13.20323	13.75629	13.72353	14.10932

GSM1108	GSM1108	GSM1108	GSM1108	GSM1108	GSM1108	GSM1108	GSM1108	GSM1108
13.947	14.65504	14.84808	14.39312	14.64968	13.89873	14.51202	15.35113	13.7695
11.30937	10.81115	10.61458	11.35954	11.28258	11.20305	11.37941	11.08467	11.13816

GSM1205	GSM1205	GSM1205	GSM1205	GSM1205	GSM1205	GSM1205	GSM1205	GSM1205
14.41255	14.19935	13.36267	14.69653	14.61458	14.38162	14.56202	14.31998	15.36284
11.22076	11.53307	11.66039	11.36961	11.29464	11.2813	11.46059	11.45846	11.15979

GSM3403	GSM3403	GSM3403	GSM3403	GSM3403	GSM3403	GSM3403	GSM3403	GSM3403
11.28326	11.81213	11.35466	11.05968	11.4532	12.02174	11.80964	10.86688	11.35383
14.23081	14.94798	13.27359	13.9433	13.42123	13.14527	13.82669	14.61944	13.03286

GSM3403	GSM3403	GSM3403	GSM3403	GSM3403	GSM3403	GSM3403	GSM3403	GSM3403
12.40161	11.99191	12.68755	10.54767	12.23689	11.49128	12.85655	11.28978	11.09901
13.81008	12.75567	13.91876	13.32183	13.55739	13.75339	12.1777	15.2005	13.65053

GSM1108	GSM1108	GSM1108	GSM1108	GSM1108	GSM1108	GSM1108	GSM1108	GSM1108
14.36983	14.75038	13.7348	14.89346	14.10765	14.2821	14.4347	13.9154	14.11728
11.14042	10.99659	10.63425	10.98158	11.20782	11.12589	11.03646	11.03688	11.28825

GSM1205[illegible]	GSM1205[illegible]	GSM1205[illegible]	GSM1205[illegible]	GSM1205[illegible]	GSM1205[illegible]	GSM1205[illegible]	GSM1205[illegible]	GSM1205[illegible]
14.00369	14.1921	14.69653	14.34599	14.06294	13.62127	13.99238	13.56561	13.76293
11.37022	11.33203	10.86257	11.34193	11.38953	11.32521	11.05729	11.05446	10.73217

GSM3403[illegible]	GSM3403[illegible]	GSM3403175
11.08174	11.72434	11.26283
14.01647	13.47679	14.19428

GSM3403[illegible]	GSM3403[illegible]	GSM3403[illegible]	GSM3403[illegible]	GSM3403[illegible]	GSM3403[illegible]	GSM3403[illegible]	GSM3403[illegible]	GSM3403[illegible]
11.11608	12.01385	11.41093	10.85105	10.96697	11.35697	10.33448	11.70028	11.86732
14.92983	13.90179	13.28335	15.01386	13.83313	14.61973	13.47942	14.22363	14.0689

GSM1108[illegible]	GSM1108[illegible]	GSM1108[illegible]	GSM1108[illegible]	GSM1108[illegible]	GSM1108[illegible]	GSM1108[illegible]	GSM1108[illegible]	GSM1108[illegible]
14.21821	13.03067	13.39892	14.11914	14.13037	14.42254	14.2384	14.01898	14.14679
10.84741	11.60272	11.8948	11.22547	11.1107	11.05648	11.04698	10.80572	11.37582

GSM1205	GSM1205	GSM1205	GSM1205	GSM1205	GSM1205	GSM1205	GSM1205	GSM1205
14.15189	14.16428	14.92301	14.16165	14.28967	13.84951	15.03678	13.09937	14.30297
11.80093	11.58888	11.07561	11.27068	11.67857	11.26853	11.19642	11.70456	11.46677

GSM3403	GSM3403	GSM3403	GSM3403	GSM3403	GSM3403	GSM3403	GSM3403	GSM3403
11.75484	11.58226	11.18225	11.08487	11.46389	10.83352	11.12199	11.3684	11.36978
13.58269	13.39019	14.44871	14.00356	14.21012	13.79401	14.62475	13.43114	13.30809

GSM1108	GSM1108	GSM1108	GSM1108	GSM1108	GSM1108	GSM1108	GSM1108	GSM1108
14.24681	14.00441	14.4822	12.32983	13.6905	14.23224	14.49432	13.63886	14.84201
11.27623	10.77709	11.22916	11.10218	11.15202	11.14372	11.23951	11.42731	11.03732

GSM1205	GSM1205	GSM1205	GSM1205	GSM1205	GSM1205	GSM1205	GSM1205	GSM1205
14.17401	13.95825	13.04896	15.01347	14.70969	14.17282	13.96345	13.49193	12.34865
11.30149	11.31267	11.27316	11.72693	11.62543	11.32617	11.35349	11.36911	11.94805

GSM3403	GSM3403	GSM3403	GSM3403	GSM3403	GSM3403	GSM3403	GSM3403	GSM3403
11.20097	11.6439	11.42976	11.36749	11.75111	11.28978	11.47008	11.32773	11.84529
13.84041	13.35088	13.13239	13.71301	14.3346	13.77441	13.65174	13.6269	14.08961

GSM1108	GSM1108	GSM1108	GSM1108	GSM1108	GSM1108	GSM1108	GSM1108	GSM1108
13.70361	14.18164	14.11159	14.87034	14.13435	14.09636	13.85542	15.11919	13.3467
11.79686	11.15031	11.15242	11.01506	11.49957	11.48901	10.83639	10.62215	11.05995

GSM1205[illegible]	GSM1205[illegible]	GSM1205[illegible]	GSM1205[illegible]	GSM1205[illegible]	GSM1205[illegible]	GSM1205[illegible]	GSM1205[illegible]	GSM1205[illegible]
13.5608	13.426	13.58118	14.4798	14.65273	13.49758	13.61001	14.11341	14.32949
11.41518	11.54062	11.47663	11.07582	10.70382	11.43958	11.1079	10.923	10.85423

GSM3403[illegible]	GSM3403[illegible]	GSM3403[illegible]	GSM3403[illegible]	GSM3403[illegible]	GSM3403[illegible]	GSM3403[illegible]	GSM3403[illegible]	GSM3403[illegible]
11.71204	11.44421	11.93413	11.77856	11.72924	11.09949	11.24758	11.6439	11.59596
13.20835	13.43356	13.98942	13.79104	13.37322	13.30282	13.16714	13.31652	13.81589

GSM1108[illegible]	GSM1108[illegible]	GSM1108[illegible]	GSM1108[illegible]	GSM1108[illegible]	GSM1108[illegible]	GSM1108[illegible]	GSM1108[illegible]	GSM1108[illegible]
14.37432	14.47494	14.04918	14.25121	13.75752	14.79925	14.49189	15.20436	14.62833
11.25557	11.29419	11.00155	11.11156	11.38694	10.78214	11.17279	10.80734	11.00059

GSM1205	GSM1205	GSM1205	GSM1205	GSM1205	GSM1205	GSM1205	GSM1205	GSM1205
14.21266	13.78727	14.73364	13.81061	14.37911	13.95778	15.02364	13.33423	12.98878
11.14747	11.4832	11.09626	11.08159	10.93652	11.3955	11.24299	11.52197	11.59108

GSM3403	GSM3403	GSM3403	GSM3403	GSM3403	GSM3403	GSM3402	GSM3402	GSM3402
11.37796	11.80554	11.46486	11.67673	11.5539	11.87938	10.98894	11.43902	11.91718
13.37429	13.25052	13.90485	13.13602	12.37727	14.23754	14.78827	13.67845	13.26787

GSM1108	GSM1108	GSM1108	GSM1108	GSM1108	GSM1108	GSM1108	GSM1108	GSM1108
13.64835	13.88098	14.12075	14.77472	13.69762	13.91367	14.21324	13.96506	12.99038
11.73151	10.91615	11.31783	11.08911	11.30458	11.20305	11.47862	11.34329	11.15458

GSM1205	GSM1205	GSM1205	GSM1205	GSM1205	GSM1205	GSM1205	GSM1205	GSM1205
14.27405	14.07215	14.04616	14.49441	14.48461	14.25566	13.85284	13.72372	13.20772
10.61796	11.17229	11.47122	11.20912	11.07438	11.44444	10.97278	10.64105	11.50992

GSM3403	GSM3403	GSM3403	GSM3403	GSM3403	GSM3403	GSM3403	GSM3403	GSM3403
11.41803	11.50954	11.85828	11.20999	11.55938	11.96951	12.4163	10.69646	11.25379
13.66202	14.22139	13.49414	14.76282	13.64913	12.44218	13.12997	13.90857	15.07215

GSM1108	GSM1108	GSM1108	GSM1108	GSM1108	GSM1108	GSM1108	GSM1108	GSM1108
13.70645	13.05194	14.82991	14.45484	14.34767	13.95391	13.82759	14.25346	14.02039
11.16972	11.05691	11.17013	11.31271	11.27449	11.23314	10.95945	11.24011	11.23267

GSM12053	GSM12053	GSM12053	GSM12053	GSM12053	GSM12053	GSM12054	GSM12054	GSM12054
13.15704	13.45996	14.54977	13.96453	14.67992	13.17601	13.30873	14.7138	13.0222
11.31584	11.58647	11.38431	11.00743	11.13962	10.73652	10.87294	11.66475	11.19176

GSM34030	GSM34030	GSM34031	GSM34031	GSM34031	GSM34031	GSM34031	GSM34031	GSM34031
11.17447	11.28071	11.80895	11.35171	11.05821	11.45028	12.01834	11.34531	11.80636
14.77691	14.22538	14.94678	13.26503	13.92938	13.40873	13.13565	14.02129	13.81228

GSM11088	GSM11088	GSM11088	GSM11088	GSM11088	GSM11088	GSM11088	GSM11088	GSM11088
13.95936	14.00276	13.28675	15.00961	14.29715	14.32735	14.27343	14.2238	13.30123
11.39794	11.33359	11.17677	11.13319	11.29763	11.24579	11.40337	11.64688	11.57902

GSM1205	GSM1205	GSM1205	GSM1205	GSM1205	GSM1205	GSM1205	GSM1205	GSM1205
14.55208	13.91479	14.62607	13.4925	14.29453	13.85777	13.40091	13.04984	13.58034
11.08582	11.09416	11.14443	11.7881	11.18808	11.20877	11.68594	11.59725	11.65313

GSM3403	GSM3403	GSM3403	GSM3403	GSM3403	GSM3403	GSM3403	GSM3403	GSM3403
10.86455	11.35091	11.08011	11.72213	11.25974	11.75006	11.70249	12.05912	11.41122
14.61403	13.02537	14.00471	13.46209	14.18766	13.17355	14.71606	13.6569	13.61526

GSM1108	GSM1108	GSM1108	GSM1108	GSM1108	GSM1108	GSM1108	GSM1108	GSM1108
14.10021	13.94033	14.1136	13.50001	13.55679	13.4745	13.5351	14.63913	14.1136
11.73564	10.97831	11.53676	10.9573	11.2449	11.82481	11.31271	11.60433	11.25742

GSM1205	GSM1205	GSM1205	GSM1205	GSM1205	GSM1205	GSM1205	GSM1205	GSM1205
13.53062	14.43473	13.84071	13.56608	13.19108	13.47371	13.75462	12.85067	13.15246
11.6343	11.22456	10.99362	11.78116	11.21351	11.54129	11.60825	11.6141	11.4787

GSM3403	GSM3403	GSM3403	GSM3403	GSM3403	GSM3403	GSM3403	GSM3403	GSM3403
11.08616	12.01953	11.2745	11.40898	11.68902	11.13439	11.66735	11.38444	11.51354
14.13703	14.90815	13.31133	15.13671	13.42806	14.37622	13.51912	15.11284	13.90857

GSM1108	GSM1108	GSM1108	GSM1108	GSM1108	GSM1108	GSM1108	GSM1109	GSM1109
13.91208	13.41728	14.34537	14.02383	12.53838	14.71216	13.59612	13.94883	14.29943
11.27177	11.28121	11.61736	10.78573	11.02258	11.92337	11.67606	12.16966	11.69355

GSM12055	GSM12055	GSM12055	GSM12055	GSM12056	GSM12057	GSM12057	GSM12057	GSM12057
13.73624	14.14303	13.56262	14.4798	12.89184	14.3364	15.3845	14.12355	12.3402
11.52919	11.48983	11.40323	10.98678	11.7665	11.54507	11.39921	11.74087	11.5379

GSM34033	GSM34033	GSM34033	GSM34033	GSM34033	GSM34034	GSM34034	GSM34034	GSM34034
11.40125	11.39393	12.08838	11.9997	11.29656	11.48835	11.55025	10.9122	11.49719
14.22329	14.47631	13.73145	13.12221	14.17828	13.60534	14.13539	14.83119	13.43172

GSM11090	GSM11090	GSM11090	GSM11090	GSM11090	GSM11090	GSM11090	GSM11090	GSM11090
13.60669	13.23929	15.09939	13.61996	14.60153	15.10333	14.84201	14.56793	13.50344
11.25328	11.02299	11.02439	11.4494	10.60194	11.46828	11.83857	10.96592	10.88278

GSM1205	GSM1205	GSM1205	GSM1205	GSM1205	GSM1205	GSM1205	GSM1205	GSM1205
13.17697	13.94588	13.19618	13.35477	13.08838	13.63251	13.72399	13.01852	13.24308
11.32669	11.48677	11.38966	11.43093	11.20292	11.17157	11.36045	10.73571	10.3998

GSM3403	GSM3403	GSM3403	GSM3403	GSM3403	GSM3403	GSM3403	GSM3403	GSM3402
11.55025	11.23509	11.59295	11.25217	10.32575	11.71319	11.17184	11.31779	11.59802
13.42275	13.61797	14.07457	14.3674	14.74916	13.89971	14.58725	14.31863	14.10933

GSM1109	GSM1108	GSM1108	GSM1108	GSM1108	GSM1108	GSM1108	GSM1108	GSM1108
14.09075	13.6306	14.63627	13.515	14.31144	13.22452	14.614	14.75669	14.72445
10.88063	11.25823	11.1966	11.81042	11.23738	11.53372	11.16088	10.66261	11.76277

GSM12052	GSM12053	GSM12053	GSM12053	GSM12053	GSM12053	GSM12053	GSM12053	GSM12053
13.83598	13.2758	13.41393	14.34023	13.46818	14.08509	13.20054	14.07131	14.31044
11.06038	11.1614	11.82605	10.92953	11.11796	10.91498	11.18703	11.07596	11.23334

GSM34029	GSM34029	GSM34029	GSM34030	GSM34030	GSM34030	GSM34030	GSM34030	GSM34030
11.38543	11.49437	11.17397	11.4345	11.78827	11.60009	11.35877	10.37938	12.00651
13.67868	14.20306	14.42285	14.31189	14.47517	13.76037	13.78047	15.6272	14.436

GSM11084	GSM11084	GSM11084	GSM11085	GSM11085	GSM11085	GSM11085	GSM11085	GSM11085
13.42912	13.93283	14.50421	14.03908	13.95876	13.6067	14.02968	14.1548	13.76274
11.68189	11.12641	11.00139	11.32299	11.41548	11.68863	11.40547	11.56381	11.56127

GSM1205	GSM1205	GSM1205	GSM1205	GSM1205	GSM1205	GSM1205	GSM1205	GSM1205
13.64879	13.11084	13.43931	13.40215	13.28255	13.93111	13.72339	13.13123	14.53554
11.17755	11.15672	11.11819	10.99752	11.08078	11.12301	10.939	11.23402	11.35637

GSM3403	GSM3403	GSM3403	GSM3403	GSM3403	GSM3403	GSM3403	GSM3403	GSM3403
10.61435	11.07612	11.58836	11.14299	11.30076	11.55261	11.98518	11.33442	11.51761
15.08466	14.08973	14.22382	15.72063	14.81152	14.05147	13.89305	14.49144	13.44523

GSM1108	GSM1108	GSM1108	GSM1108	GSM1108	GSM1108	GSM1108	GSM1108	GSM1108
13.59486	13.25048	14.29251	13.49894	14.09408	13.61166	14.22334	14.05026	13.17896
11.39435	11.76629	10.59476	11.53328	11.20589	11.502	11.14422	11.51979	11.30897

GSM12053	GSM12054	GSM12054	GSM12054	GSM12054	GSM12054	GSM12054	GSM12054	GSM12054
13.02245	13.42215	12.17253	13.65435	13.20187	13.5612	14.01653	12.5781	13.89127
11.0983	11.34158	11.00859	10.92346	10.37965	10.96064	11.36471	11.60367	10.92996

GSM34031	GSM34031	GSM34031	GSM34031	GSM34031	GSM34031	GSM34031	GSM34031	GSM34031
11.03577	11.25438	11.23326	11.51352	11.76014	11.68462	11.32301	10.89981	11.33094
15.17325	14.88091	13.77164	14.06548	15.11336	13.37655	13.55933	14.37389	13.92343

GSM11085	GSM11086	GSM11086	GSM11086	GSM11086	GSM11086	GSM11086	GSM11086	GSM11086
14.51144	14.51472	13.86624	13.22877	14.28582	13.754	14.82306	14.67377	13.53133
11.23156	11.10518	10.99335	11.55448	11.48162	10.66185	11.63115	10.75873	11.42303

GSM1205	GSM1205	GSM1205	GSM1205	GSM1205	GSM1205	GSM1205	GSM1205	GSM1205
14.7794	13.84572	13.76754	13.17073	12.96901	14.40887	13.07931	14.47757	13.62846
10.9808	11.1862	11.47549	11.93795	11.39891	10.92811	11.50143	11.25557	11.56165

GSM3403	GSM3403	GSM3403	GSM3403	GSM3403	GSM3403	GSM3403	GSM3403	GSM3403
11.76068	11.15635	11.90492	11.87488	11.60158	11.09494	11.09622	11.03028	11.41609
13.79489	14.2797	16.33642	14.08804	13.94523	14.39887	14.2652	13.90385	13.50436

GSM1108	GSM1108	GSM1108	GSM1108	GSM1108	GSM1108	GSM1108	GSM1108	GSM1108
14.39584	13.68196	13.64122	14.13596	14.35472	14.57202	13.98755	13.22252	13.37632
10.92495	11.3168	11.04873	10.90363	10.81176	11.38859	11.02708	10.71155	11.49128

GSM1205	GSM1205	GSM1205	GSM1205	GSM1205	GSM1205	GSM1205	GSM1205	GSM1205
13.33994	13.80223	13.75197	13.41525	13.61696	12.7984	13.39581	13.16596	13.00922
11.26774	11.68528	11.26151	11.44785	11.8304	11.29143	11.41505	11.14885	11.11022

GSM3403	GSM3403	GSM3403	GSM3403	GSM3403	GSM3403	GSM3403	GSM3403	GSM3403
11.54929	11.30363	11.76565	11.16376	10.97778	11.65228	11.41943	11.30748	10.7293
13.93202	14.67129	14.35686	13.63798	14.33304	13.46386	14.17634	13.58851	13.49308

GSM1108	GSM1108	GSM1108	GSM1108	GSM1108	GSM1108	GSM1108	GSM1108	GSM1108
14.24242	13.46435	13.82294	13.9894	15.03878	13.81242	14.75392	13.03391	14.50421
11.13975	11.51282	11.08713	11.40318	11.20452	11.45755	11.05798	11.49768	11.04652

GSM12054	GSM12055	GSM12055	GSM12055	GSM12055	GSM12055	GSM12055	GSM12055	GSM12055
13.50992	14.25075	13.14422	12.95349	13.01118	13.13601	13.19013	12.94047	13.56796
11.64932	10.96895	11.75046	11.24049	11.30847	11.608	11.53763	11.0327	11.2879

GSM34033	GSM34033	GSM34033	GSM34033	GSM34033	GSM34033	GSM34034	GSM3403426
10.95886	11.02363	11.05213	11.28029	11.14747	11.05682	11.75622	11.00003
14.77545	14.27778	14.5156	14.64948	13.77017	15.22159	14.47737	14.61307

GSM11087	GSM11087	GSM11088	GSM11088	GSM11088	GSM11088	GSM11088	GSM11088	GSM11088
13.84298	14.53431	13.5027	13.77685	13.5697	13.58916	13.3349	14.57202	13.04672
11.55448	11.17587	10.95939	11.62999	11.64806	11.83289	10.90681	11.13285	11.21922

GSM1205!	GSM1205!	GSM1205!	GSM1205!	GSM1205!	GSM1205!	GSM1205!	GSM1205!	GSM1205!
13.61148	13.19117	14.47501	13.53176	13.91454	13.50582	13.98165	13.06293	14.50125
11.3879	11.87012	11.00023	10.85751	11.0137	10.90852	11.1727	11.58125	10.69021

GSM1108(	GSM1108(	GSM1108(	GSM1108(	GSM1108(	GSM1108(	GSM1108(	GSM1108(	GSM1108(
13.49894	13.18	13.21166	12.86737	13.87399	12.89495	12.90991	12.9547	13.03734
11.5708	11.48247	11.25823	11.63167	11.01321	11.07516	11.22385	11.02369	11.29858

GSM1205!	GSM1205!	GSM1205!	GSM1205!	GSM1205!	GSM1205!	GSM1205!	GSM1205!	GSM1205!
13.94808	14.91083	13.04082	13.66845	13.46456	13.91654	13.75742	13.52333	13.03445
11.1787	10.99078	11.08022	10.84116	11.23009	10.55117	11.14578	11.00792	11.07852

GSM1108!	GSM1108!	GSM1108!	GSM1108!	GSM1108!	GSM1109(	GSM1109(	GSM1109(	GSM1109(
13.45726	13.23721	15.06222	15.14458	14.64183	14.04831	14.34721	13.701	13.45372
11.19797	11.17166	10.48061	11.21376	10.98917	11.43217	11.64534	12.01515	11.52974

GSM1205!	GSM1205!	GSM1205!	GSM1205!	GSM1205!	GSM1205!	GSM1205!	GSM1205!	GSM1205!
14.01931	12.95645	12.76344	13.13016	14.00422	14.61654	13.61832	13.19355	13.32555
11.04987	11.39801	11.02384	11.44895	10.84903	11.14956	10.99054	11.03055	11.25649

GSM1109(	GSM1109(	GSM1109(	GSM1108	GSM1108	GSM1108	GSM1108	GSM1108	GSM1108
14.60853	14.25801	14.04487	13.90718	13.2992	14.00451	14.61623	13.64333	13.21494
11.23972	10.92294	11.63839	11.1353	11.70005	11.3176	11.60699	10.79435	11.37896

GSM1205	GSM1205	GSM1205	GSM1205	GSM1205	GSM1205	GSM1205	GSM1205	GSM1205
12.93772	14.31913	14.04716	13.73896	13.75197	13.81915	14.23268	14.06768	14.54343
11.04492	10.88627	10.91088	10.97991	11.08874	11.09304	11.23853	10.88149	11.23186

GSM1108	GSM1108	GSM1108	GSM1108	GSM1108	GSM1108	GSM1108	GSM1108	GSM1108
13.99896	13.75316	14.38606	13.88532	13.45278	14.30209	14.48391	13.45978	14.1433
11.22935	11.45896	11.07366	11.84518	11.66735	11.43808	11.09891	11.29761	11.87592

GSM1205!	GSM1205!	GSM1205!	GSM1205!	GSM1205!	GSM1205!	GSM1205!	GSM1205!	GSM1205!
13.65685	13.67519	13.42554	13.72399	12.83357	13.43471	13.79188	13.33871	13.62951
11.25624	11.58264	11.29695	11.09885	10.8619	11.6039	11.406	11.53712	11.55959

GSM1108	GSM1108	GSM1108	GSM1108	GSM1108	GSM1108	GSM1108	GSM1108	GSM1108
14.32591	14.14523	13.9971	13.67458	13.62587	13.0477	13.71046	14.17504	13.70187
11.0629	11.45513	11.26819	11.74657	11.43808	10.84711	11.67786	11.45311	11.16153

GSM1205	GSM1205	GSM1205	GSM1205	GSM1205	GSM1205	GSM1205	GSM1205	GSM1205
13.81576	13.44187	13.18584	13.92563	13.30674	12.94096	14.09367	12.66445	13.16361
10.95012	11.23358	11.56991	11.1291	11.49768	10.64329	11.26122	11.29905	11.35498

GSM1108	GSM1108	GSM1108	GSM1108	GSM1108	GSM1108	GSM1108	GSM1108	GSM1108
13.75023	14.77451	13.52622	13.89178	13.42021	13.66076	13.46882	13.9056	13.49901
11.38085	11.26819	11.35617	11.54638	11.40671	11.42255	11.26205	11.78089	11.66992

GSM1205[illegible]	GSM1205[illegible]	GSM1205[illegible]	GSM1205[illegible]	GSM1205[illegible]	GSM1205[illegible]	GSM1205[illegible]	GSM1205[illegible]	GSM1205[illegible]
13.3731	13.81326	13.41088	13.47395	13.97893	12.83086	13.40884	13.44841	13.7373
11.13775	11.64709	11.50229	11.01851	10.904	11.56246	11.47187	11.41717	11.43665

GSM1108[illegible]	GSM1108[illegible]	GSM1108[illegible]	GSM1108[illegible]	GSM1108[illegible]	GSM1108[illegible]	GSM1108[illegible]	GSM1108[illegible]	GSM1108[illegible]
14.29108	13.77506	13.24785	13.98051	13.67972	13.35771	14.01459	13.81923	12.96691
11.4785	11.27054	11.37512	11.27288	11.41207	11.25701	11.14543	11.06062	10.94578

GSM1205!	GSM1205!	GSM1205!	GSM1205!	GSM1205!	GSM1205!	GSM1205(	GSM1205(	GSM1205(
13.90288	13.53423	13.70696	13.12056	13.01019	12.70513	13.32214	13.7507	13.59606
11.22338	10.97284	11.12688	11.39104	11.6796	11.28225	10.89898	11.25704	11.01141

GSM1108·	GSM1108·	GSM1108·	GSM1108·	GSM1108·	GSM1108·	GSM1108·	GSM1108!	GSM1108!
13.07685	12.30383	14.08837	14.13568	13.32403	14.15298	14.00774	13.81923	13.89776
11.23163	11.13965	11.02388	11.0781	10.84795	11.02828	10.85561	11.8008	11.81411

GSM1205[illegible]	GSM1205[illegible]	GSM1205[illegible]	GSM1205[illegible]	GSM1205[illegible]	GSM1205[illegible]	GSM1205[illegible]	GSM1205[illegible]	GSM1205[illegible]
13.61979	13.17804	13.62484	13.57075	14.21687	13.08139	13.14475	12.79413	13.93779
11.39312	11.2673	11.53661	11.22778	11.3111	11.37078	11.30931	10.85932	11.46601

GSM1108[illegible]	GSM1108[illegible]	GSM1108[illegible]	GSM1108[illegible]	GSM1108[illegible]	GSM1108[illegible]	GSM1108[illegible]	GSM1108[illegible]	GSM1108[illegible]
13.25611	13.96163	13.92939	13.56606	13.69924	13.71879	13.52364	13.63638	13.52364
11.75096	11.47262	11.31059	11.37512	11.99852	11.72675	11.94207	11.11983	11.49613

GSM1205(	GSM1205(	GSM1205(	GSM1205(	GSM1205(	GSM1205(	GSM1205(	GSM1205(	GSM1205(
13.11282	13.56059	13.90448	12.84138	13.51364	12.71389	13.18058	13.10395	14.90553
11.6243	11.33564	11.11071	11.09906	11.52208	11.27728	11.3108	11.04294	11.15017

GSM1108!	GSM1108!	GSM1108!	GSM1108!	GSM1108!	GSM1108!	GSM1108!	GSM1108!	GSM1108!
13.38114	12.79514	13.26699	12.96503	13.68106	13.82539	13.98051	13.65948	12.84721
11.83162	11.42255	11.5078	11.2546	11.11576	11.57711	11.36661	11.58017	11.35944

GSM1205(	GSM1205(	GSM1205(	GSM1205(	GSM1205(	GSM1205(	GSM1205(	GSM1205(	GSM1205(
13.32214	13.019	13.56796	13.89936	13.94122	13.67125	13.68968	13.30946	13.3984
11.19672	11.41685	12.00209	11.13125	11.03916	11.30744	11.1626	11.54423	11.34635

GSM1108!	GSM1108!	GSM1108!	GSM1108!	GSM1108!	GSM1108!	GSM1108!	GSM1108!	GSM1108!
13.08346	13.18412	13.71729	13.06065	13.76764	13.55481	14.00617	13.57747	13.83619
11.61847	11.6562	11.51672	11.14543	10.97137	11.35236	10.67855	11.43958	11.30971

GSM1205(	GSM1205(	GSM1205(	GSM1205(	GSM1205(	GSM1205(	GSM1205(	GSM1205(	GSM1205(
13.96971	13.7682	12.71201	14.32338	13.48917	14.00568	12.38113	12.75023	13.93589
10.95277	10.68622	10.99347	11.10314	11.33131	11.4195	11.19672	11.51694	10.95439

GSM1108!	GSM1108!	GSM1108!	GSM1108!	GSM1108!	GSM1108!	GSM1108!	GSM1108!	GSM1108!
13.20061	14.59738	13.14713	13.61649	13.79442	13.91514	13.36347	13.8108	13.74748
11.6442	11.5017	11.2093	11.15794	11.24812	11.24812	10.52758	11.25608	11.0629

GSM1205(	GSM1205(	GSM1205(	GSM1205(	GSM1205(	GSM1205(	GSM1205(	GSM1205(	GSM1205(
13.42964	14.12258	13.23665	12.80865	14.64605	12.9621	12.89911	13.18058	13.89461
11.37591	10.9778	11.56191	11.17807	11.09773	11.3422	11.34451	11.38497	10.35878

GSM1108!	GSM1108!	GSM1108!	GSM1108!	GSM1108(	GSM1108(	GSM1108(	GSM1108(	GSM1108(
13.30251	13.71729	13.64862	13.0796	13.29808	13.51137	13.25611	13.45278	13.21088
10.55306	11.70322	11.11489	11.32855	11.661	11.57062	11.86962	11.56063	11.17097

GSM1205(	GSM1205(	GSM1205(	GSM1205(	GSM1205(	GSM1205(	GSM1205(	GSM1205(	GSM1205(
13.01274	12.86157	13.13123	12.01775	13.68317	13.65868	14.27512	12.99053	13.29788
11.68528	11.69522	11.33485	10.96425	11.46797	11.4711	11.42523	11.31316	11.46546

GSM1108(	GSM1108(	GSM1108(	GSM1108(	GSM1108(	GSM1108(	GSM1108(	GSM1108(	GSM1108(
13.96163	13.4365	13.51883	14.10352	13.06443	14.5526	14.05168	13.49784	14.96512
11.11893	11.32002	11.59441	11.21305	11.54732	11.40964	11.15286	11.49666	11.23023

GSM1205(	GSM1205(	GSM1205(	GSM1205(	GSM1205(	GSM1205(	GSM1205(	GSM1205(	GSM1205(
14.18078	13.8008	14.32243	13.70265	14.32338	13.99283	12.9485	14.32937	13.75807
10.93622	11.28922	11.68424	11.66267	11.00023	11.48625	11.29578	11.74162	11.5574

GSM1108(	GSM1108(	GSM1108(	GSM1108(	GSM1108(	GSM1108(	GSM1108(	GSM1108(	GSM1108(
13.41563	13.38657	13.5139	14.54517	13.63638	13.9971	13.37447	12.83615	14.41609
11.61434	11.25886	11.93901	11.0794	10.96013	11.14189	11.25277	11.36661	11.0362

GSM1205(	GSM1205(	GSM1205(	GSM1205(	GSM1205(	GSM1205(	GSM1205(	GSM1205(	GSM1205(
13.55707	13.85784	12.63952	14.64605	14.09658	13.41957	13.40276	13.29127	14.15335
11.74568	11.36446	10.56001	11.64111	11.60466	11.55188	12.89195	11.51914	11.56519

GSM1108(	GSM1108(	GSM1108(	GSM1108(	GSM1108(	GSM1108(	GSM1108(	GSM1108(	GSM1108(
13.73449	13.57617	13.69924	14.16497	13.71729	14.57162	13.31963	13.83139	14.56338
11.26765	11.17097	11.90866	11.04159	11.46713	11.55419	11.44829	11.44442	10.77698

GSM1205	GSM1205	GSM1205	GSM1205	GSM1205	GSM1205	GSM1205	GSM1205	GSM1205
14.29421	13.9565	13.41896	13.73428	14.16348	13.37788	13.31861	13.08955	13.44248
11.96014	11.43684	11.82675	11.27854	11.2303	11.50505	11.45476	11.5041	11.35567

GSM1108	GSM1108	GSM1108	GSM1108	GSM1108	GSM1108	GSM1108	GSM1108	GSM1108
14.0377	14.02701	14.85148	13.24886	13.15414	12.88206	13.4365	13.12606	14.15298
11.29949	11.21067	11.49214	11.731	11.20298	11.15143	11.01437	11.20387	11.18867

GSM1205(	GSM1205`	GSM1205`	GSM1205`	GSM1205`	GSM1205`	GSM1205`	GSM1205`	GSM1205`
14.75632	12.83828	13.14422	13.69949	12.42005	13.95732	12.81052	13.05129	13.04958
10.61637	10.78729	11.57167	11.12486	11.2306	10.96102	11.21779	11.23792	11.25624

GSM1108`	GSM1108`	GSM1108`	GSM1108`	GSM1108`	GSM1108`	GSM1108`	GSM1108`	GSM1108`
13.28738	14.94812	13.13111	14.33061	13.23752	13.17709	13.85497	13.59697	13.53718
11.40192	11.25936	10.85176	11.33146	11.20696	11.63899	11.33241	11.00548	11.19706

GSM1205	GSM1205	GSM1205	GSM1205	GSM1205	GSM1205	GSM1205	GSM1205729
12.70642	13.5325	13.81257	13.49037	13.26455	13.14082	13.89646	13.14579
11.75206	10.67281	11.13199	10.69271	11.48677	11.15756	11.69372	11.29951

GSM1108	GSM1108	GSM1108	GSM1108	GSM1108	GSM1108	GSM1108	GSM1108	GSM1108
13.50664	14.37917	14.09401	13.28	13.75596	13.21802	14.01273	13.40562	13.14408
11.07143	11.33855	11.26111	11.42598	11.223	11.27528	11.5696	11.16605	11.5133

GSM1108	GSM1108	GSM1108	GSM1108	GSM1108	GSM1108	GSM1108	GSM1108	GSM1108
14.04285	12.51317	12.91716	13.78538	13.41563	14.01997	14.34422	13.80772	13.40881
11.32993	11.34432	11.32614	11.59549	11.22798	11.29708	11.57659	11.72573	11.52576

GSM1108	GSM1108	GSM1108	GSM1108	GSM1108	GSM1108	GSM1108	GSM1108	GSM1108
14.2615	13.43062	13.73879	13.096	13.52495	13.68662	14.68518	12.92431	13.84089
11.14682	11.43229	11.66945	11.44591	11.75364	11.17914	11.3329	11.41259	11.25748

GSM1108	GSM1108	GSM1108	GSM1108	GSM1108	GSM1108	GSM1108	GSM1108	GSM1108
13.0364	13.8298	13.52248	13.76902	13.9568	13.82539	13.90246	13.30027	14.15908
11.1957	11.43662	11.47704	11.7305	11.731	11.11983	11.25886	11.16285	11.11799

GSM1108{	GSM1108{	GSM1108{	GSM1108{	GSM1108{	GSM1108{	GSM1108{	GSM1108{	GSM1108{
13.41224	13.24886	14.08262	13.61512	13.23752	13.52745	14.63257	13.07486	13.29808
11.56112	11.43468	11.55369	11.77486	11.57455	11.4907	11.38995	11.53835	12.01352

GSM1108	GSM1108	GSM1108	GSM1108	GSM1108	GSM1108	GSM1108	GSM1108	GSM1108
13.13015	13.27882	13.43995	13.17915	13.24165	13.58146	14.05361	12.96858	12.91895
11.80408	11.28502	11.41491	11.52881	11.45802	11.14727	11.07143	11.66735	10.95539

GSM1108	GSM1108	GSM1108	GSM1108	GSM1108	GSM1108	GSM1108	GSM1108	GSM1108
14.00934	13.6459	13.51022	13.22398	12.34835	12.98735	13.15317	13.28633	13.33781
11.58735	11.5133	11.5736	11.75042	11.29708	11.80018	11.36365	11.39865	11.78966

GSM1108!	GSM1108!	GSM1108!	GSM1108!	GSM1108!	GSM1108!	GSM1108!	GSM1108!	GSM1108!
13.14192	13.38326	13.41344	13.27243	12.71379	11.98439	12.49073	11.90529	12.77763
11.03665	11.5691	11.74493	12.06055	11.62787	11.38708	11.69588	11.7417	11.61329

GSM1108	GSM1108	GSM1108	GSM1108	GSM1108	GSM1108	GSM1108	GSM1108	GSM1108
12.84126	13.09981	14.22538	13.48561	14.33061	14.03931	13.39868	14.39106	14.29521
11.32424	11.31808	11.40964	11.62477	11.22075	11.46082	11.6359	11.2043	11.5428

GSM1108[illegible]	GSM1108[illegible]	GSM1108[illegible]	GSM1108[illegible]	GSM1108[illegible]	GSM1108[illegible]	GSM1108[illegible]	GSM1108[illegible]	GSM1108[illegible]
13.91676	13.80925	12.75626	14.89403	14.68518	14.16121	13.50776	13.49784	13.3611
11.5443	12.12551	10.73162	11.29761	11.83392	11.78407	11.72839	12.96769	11.71976

GSM1108!	GSM1108!	GSM1108!	GSM1108!	GSM1108!	GSM1108!	GSM1108!	GSM1108!	GSM1108!
14.22538	14.34648	12.95601	14.02512	13.75023	15.36926	13.02899	14.07886	13.28841
11.7444	12.12918	11.53735	11.6039	11.12269	11.35328	11.74657	11.04598	11.53133

GSM1108	GSM1108	GSM1108	GSM1108	GSM1108	GSM1108	GSM1108	GSM1108	GSM1108
13.84252	13.97349	15.06362	13.57747	13.24886	13.8949	13.5918	14.77148	14.38606
10.85433	11.86397	11.44487	11.50636	11.47508	11.33146	10.84711	11.24071	11.46379

GSM1108	GSM1108	GSM1108	GSM1109	GSM1109	GSM1109	GSM1109	GSM1109	GSM1109
13.82389	14.07713	13.73879	14.05537	13.05886	13.9971	13.81632	13.65403	14.41393
11.77274	12.07409	11.41639	11.64369	11.43516	11.80674	11.43906	12.12171	11.81124

GSM1109(	GSM1109(	GSM1109(	GSM1109(	GSM1109(	GSM1109(	GSM1109(	GSM1109(	GSM1109(
14.10159	13.89178	14.56338	13.24785	14.57678	14.38606	13.84089	13.52495	13.3611
11.58125	11.32901	11.60494	11.30321	11.54638	11.91046	11.81235	11.54535	11.66205

GSM1109(	GSM1109(	GSM1109(	GSM1109048
13.87239	14.26973	12.98999	12.80895
11.09491	11.31428	11.54029	11.17545
